# Characterization of the Fecal Microbiome from Non-Human Wild Primates Reveals Species Specific Microbial Communities

**DOI:** 10.1371/journal.pone.0013963

**Published:** 2010-11-12

**Authors:** Suleyman Yildirim, Carl J. Yeoman, Maksim Sipos, Manolito Torralba, Brenda A. Wilson, Tony L. Goldberg, Rebecca M. Stumpf, Steven R. Leigh, Bryan A. White, Karen E. Nelson

**Affiliations:** 1 The Institute for Genomic Biology, University of Illinois, Urbana, Illinois, United States of America; 2 Department of Physics, University of Illinois, Urbana, Illinois, United States of America; 3 The J. Craig Venter Institute, Rockville, Maryland, United States of America; 4 Department of Microbiology, University of Illinois, Urbana, Illinois, United States of America; 5 Department of Pathobiological Sciences, University of Wisconsin, Madison, Wisconsin, United States of America; 6 Department of Anthropology, University of Illinois, Urbana, Illinois, United States of America; 7 Department of Animal Sciences, University of Illinois, Urbana, Illinois, United States of America; East Carolina University School of Medicine, United States of America

## Abstract

**Background:**

Host-associated microbes comprise an integral part of animal digestive systems and these interactions have a long evolutionary history. It has been hypothesized that the gastrointestinal microbiome of humans and other non-human primates may have played significant roles in host evolution by facilitating a range of dietary adaptations. We have undertaken a comparative sequencing survey of the gastrointestinal microbiomes of several non-human primate species, with the goal of better understanding how these microbiomes relate to the evolution of non-human primate diversity. Here we present a comparative analysis of gastrointestinal microbial communities from three different species of Old World wild monkeys.

**Methodology/Principal Findings:**

We analyzed fecal samples from three different wild non-human primate species (black-and-white colobus [*Colubus guereza*], red colobus [*Piliocolobus tephrosceles*], and red-tailed guenon [*Cercopithecus ascanius*]). Three samples from each species were subjected to small subunit rRNA tag pyrosequencing. *Firmicutes* comprised the vast majority of the phyla in each sample. Other phyla represented were *Bacterioidetes, Proteobacteria, Spirochaetes, Actinobacteria, Verrucomicrobia, Lentisphaerae, Tenericutes, Planctomycetes, Fibrobacateres*, and TM7. Bray-Curtis similarity analysis of these microbiomes indicated that microbial community composition within the same primate species are more similar to each other than to those of different primate species. Comparison of fecal microbiota from non-human primates with microbiota of human stool samples obtained in previous studies revealed that the gut microbiota of these primates are distinct and reflect host phylogeny.

**Conclusion/Significance:**

Our analysis provides evidence that the fecal microbiomes of wild primates co-vary with their hosts, and that this is manifested in higher intraspecies similarity among wild primate species, perhaps reflecting species specificity of the microbiome in addition to dietary influences. These results contribute to the limited body of primate microbiome studies and provide a framework for comparative microbiome analysis between human and non-human primates as well as a comparative evolutionary understanding of the human microbiome.

## Introduction

Host-microbe interactions can be commensal, mutualistic, pathogenic or parasitic, and these interactions are known to influence the metabolic, developmental, and immunological status of hosts [1]–[4]. The interactions between animals and microbes have a long evolutionary history and therefore have a profound influence in shaping life on earth. Dietary adaptation is a fundamental driving force for the evolution of all species, including human and non-human primates (NHPs) [5]. Indeed, major evolutionary events separating humans from NHPs probably are centered on dietary adaptations [6]–[7]. Not surprisingly, differences in the anatomy of vertebrate digestive tracts are often correlated with the quality and abundance of food. Although the mechanism of digestion is evolutionarily conserved across vertebrate taxa, the digestive systems of individual species have evolved in response to structural and nutritional qualities of specific diets, which is a fundamental component of a species' overall ecology. Our current understanding of how dietary adaptations impact primate evolution and diversity is based on ecological, morphological, and behavioral data, as well as correlations between encephalization and high quality diets [8]–[9]. Because gut microbes play a critical role in species adaptation to a particular diet by providing the host with critical metabolic pathways, it is expected that microbes might influence host evolution. However, their role in the evolution of primate diets has not yet been extensively explored.

There is a strong body of evidence that obligate mutualist and commensal bacteria associated with insects are crucial to host survival by not only provisioning the host essential nutrients but also protecting it from hostile competitors [10]–[11]. Moreover, normal development in a variety of animals and plants requires the presence of symbiotic bacteria, as in the example of the *Vibrio fisheri*-induced light organ in the squid [12] or the association of nitrogen-fixing soil bacteria (Rhizobia, Actinomycetes) with plants [13]. The same is true in mammalian systems. Mazmanian et al. (2005) [14] for example demonstrated that the *Bacteroides fragilis* polysaccharide directs the cellular and physical maturation of the developing immune system of the mouse host, including correcting systemic T cell deficiencies and T(h)1/T(h)2 imbalances and directing lymphoid organogenesis. Remarkably, molecular and experimental approaches in recent reports show that human metabolic phenotypes are strongly influenced by the gut microbiome [15]–[18]. Interestingly, \an obesity phenotype in mice was reportedly associated with changes in the microbiome that increase host capacity to harvest energy from the diet [17], [18]. This was also found to be a transmissible trait. Thus, a direct role of microbes in creating energy surpluses suggests that microbes may affect tissue trade-offs [8] and development. Conversely, host adaptation to a specific diet provides gut microbes with the opportunity to evolve and reciprocally adapt to the host gut environment and diet, resulting in host-driven diversification and co-evolution of both species [19], [20]. We have only limited knowledge of how the association with indigenous microorganisms has shaped the genomes, microbiomes, physiology or postnatal development of primates [3]. We have therefore adopted the overall goal of determining how the gastrointestinal microbiome of primates correlates with their phylogenetic distribution along with their ecological, morphological, and behavioral features.

Ley et al. [21], [22] performed pioneering studies by comparing the microbiomes of a range of mammalian species and hypothesized that comparing the human gastrointestinal microbiome with those of non-human primates and other mammals could reveal a core set of gastrointestinal microbial genes and microbial lineages that may be unique to humans. This study revealed a correlation between host diet and microbial community composition. However, although gastrointestinal microbes are presumed to play a crucial role in diet-driven speciation, this role has not been adequately explored. Wider surveys of primate gastrointestinal microbiomes, particularly of wild primates, combined with analyses of their microbiomes are necessary to better understand the overall influence of microbes on primate evolution.

To better understand how microbiomes relate to non-human primate ecological and evolutionary diversity, we have undertaken a comparative sequencing survey of the microbiomes of the gastrointestinal tracts of several NHPs. Herein, we present deep hypervariable 16S rRNA tag sequencing and comparative analysis of the gastrointestinal microbiomes associated with wild Old World monkeys from nine individuals belonging to three different species.

## Results

NHP species included in this study and their important characteristics are shown in Table 1. We used the Roche 454 GS-FLX Titanium system to obtain 136,750 high-quality sequences from three datasets targeting the V1-V3 hypervariable region of the 16S rRNA gene (62,638 sequences generated using the 27F primer, ranging from 4314-8778 reads per subject; 62,552 sequences using the 534R primer, ranging from 3759-9090 reads per subject; and 9,565 bi-directional, full-length sequences spanning the 27F primer to the 534R primer, ranging from 292–1579 reads per subject, Table 2; Sequence Data S1, S2, and S3, respectively). Median sequence lengths were 262 bp (range 223–316 nt) in the 27F primer generated data set, encompassing the V1-V2 hypervariable regions; 287 bp (range 271–338 nt) in the 534R data set, including the V3 hypervariable region, and 433 bp (range 415–482 nt) in 27F-534R data set, including V1, V2, and V3 hypervariable regions [23]. All three data sets were rigorously screened to remove poor quality reads (short reads <200 nt, zero-ambiguous sequences, and homopolymers <6) and to ensure that sequences were aligned over the same range of starting and ending nucleotides. As a result of applying these stringent criteria 41% of the total sequences in the 27F data set, 37% of 534R dataset and 22% of the 27F-534R dataset were excluded from the analysis. Even though the 27F and 534R datasets contained a comparable number of sequences per subject, the number of observed operational taxonomic units (OTUs) per subject in the 27F dataset was consistently more than that in the 534R dataset (Table 2). Whereas comparison of species richness estimates between different regions of 16S rRNA was out of the scope of this work, our findings are in accord with a recent report [24], showing that the V1-V2 region overestimated species richness unlike the V3 region, which underestimated the richness. Consequently, the true species richness in our analysis should be close to the average of the number of OTUs obtained in both data sets.

**Table 1:** Characteristics of non-human primate subjects in this study*.

Latin Name	Common Name	Habitat	General Distribution	Diet Preference	Fermentation
*Colobus guereza*	Black-and-White Colobus	Lowland rain forests, gallery, and montane forests	East and Central Africa	Mostly fresh leaves. Also, whole fruits, flowers, seeds	Foregut
*Piliocolobus tephrosceles*	Tephrosceles Red Colobus**	Lowland rain forests, gallery, and montane forests	Kenya and Uganda	Fresh leaves, flowers	Foregut
*Cercopithecus ascanius*	Red-Tailed Guenon	Lowland and montane forest, swamp forest, agricultural lands	Central Africa	Primarily fruit and insects, some leaves and flowers	Hindgut

*Fashing, P; Jaffe, KE, and Isbell, LA, in Primates in Perspective, Oxford University Press, 2^nd^ Edition.

**Grubb, P., T. M. Butynski, J. F. Oates, S. K. Bearder, T. R. Disotell, C. P. Groves, and T. T. Struhsaker. 2003. Assessment of the diversity of African primates. Int. J. Primatol. 24:1301–1357.

The latest 454-pyrosequencing GS FLX Titanium technology reagents are designed such that both ends of amplicons can be sequenced. Sequencing amplicons at both ends of the V1-V3 region in this study allowed us to verify our results on the microbial community composition and structure. We wanted to determine whether the two datasets compare favorably in terms of describing microbial diversity. We therefore employed ‘Analysis of Similarities’ or ANOSIM testing (using PRIMER-E, v6, [25]), a distribution independent analogue of one-way ANOVA [26], under the null hypothesis that there are no significant differences in the diversity described in the two datasets. This analysis showed that the datasets generated using the 27F and 534R primers did not generate significant differences in microbial diversity at 97, 95, and 90% OTU definition (*P*>0.05; *p* = 0.10, *R* = −0.229 at 97% similarity level; *p = *0.179, R = 0.083 at 95% similarity; *p* = 0.001, R = 0.396 at 90% similarity). Nevertheless, an unpaired t-test (Graphpad software online calculators, www.graphpad.com) showed evenness calculated for the same pair of subjects in both datasets were significantly different (*P*<0.05; *P* = 0.001), which may be attributed to the variable degree of sequence conservation in both regions [24], [27].

### Bacterial diversity in non-human primate fecal samples

Bacterial diversity varied significantly among the NHP species included in this study. The total number of OTUs at 97% sequence similarity ranged from 1063–2525 in the 27F dataset and 780–1582 in the 534R dataset. As expected, the 27F-534R dataset showed considerably lower OTUs (a range of 132–641) because of a relatively shallow sequencing depth (Table 2). On the other hand, rarefaction curves (Figures S1–S3) failed to plateau in any of the samples analyzed, suggesting that the actual number of OTUs in each sample is higher. Consistent with this observation, the Chao1 estimates of richness were substantially higher (39%–105%) than the observed number of OTUs as shown in Table 2. On average, observed bacterial richness was highest in the red colobus, followed by red-tailed guenon, and black-and-white colobus, though each subject showed variable numbers of OTUs. In terms of Shannon diversity estimate and Shannon estimator of evenness, red colobus monkeys were consistently among the highest in all three data sets. Thus, red colobus showed the highest level of bacterial diversity compared to the other two species in this study. Good's estimator showed approximately 90% coverage in all subjects (Table 2), indicating that additional OTUs would be found if sampling continued, in line with trends shown in rarefaction curves (Figures S1–S3).

**Table 2:** Number of OTUs and estimators of sequence diversity and richness.

	BWC1^1^	BWC2	BWC3	RC1^1^	RC2	RC3	RTG1^1^	RTG2	RTG3
**27F** 4									
# of Sequences	8664	4519	7618	6698	6116	8778	4314	8674	7257
# of OTUs^2^	1372	1072	1442	1697	1791	2522	1306	1867	1827
Chao1 (Richness)	2269	1677	2282	2792	3008	4119	2246	3121	3072
Shannon (Diversity, H)	6.20	6.14	6.24	6.63	6.88	7.22	6.56	6.63	6.75
Evenness (*E_H_*)^3^	0.36	0.44	0.36	0.44	0.54	0.54	0.54	0.41	0.46
Good's coverage	0.93	0.89	0.91	0.87	0.86	0.86	0.85	0.89	0.88
**534R** 4									
# of Sequences	9090	3785	7897	7199	6779	8687	3759	7294	8062
# of OTUs	919	787	1067	1207	1250	1592	776	1348	1369
Chao1 (Richness)	1331	1217	1636	1707	1713	2263	1073	2460	2271
Shannon (Diversity, *H*)	5.69	5.94	5.91	6.30	6.43	6.66	5.92	6.11	6.14
Evenness (*E_H_*)	0.32	0.48	0.34	0.45	0.50	0.49	0.48	0.33	0.34
Good's coverage	0.96	0.91	0.94	0.94	0.93	0.93	0.90	0.90	0.91
**27F-534R** 4									
# of Sequences	1449	292	1563	885	1067	1579	673	1373	684
# of OTUs	299	132	335	351	416	641	307	512	345
Chao1 (Richness)	458	251	552	638	628	1050	547	1329	710
Shannon (Diversity, *H*)	5.01	4.42	5.05	5.42	5.68	6.09	5.37	5.42	5.42
Evenness (*E_H_*)	0.50	0.63	0.47	0.64	0.70	0.69	0.70	0.44	0.65
Good's coverage	0.91	0.73	0.90	0.78	0.81	0.79	0.74	0.75	0.68

1 BWC-Black and White colobus; RC: Red colobus; RTG: Red-tailed guenon.

2 Operational Taxonomic Unit (OTUs) at 97% similarity.

3 (EH  =  Exp (H)/Sobs, where H is Shannon diversity index, Sobs is total number of OTUs).

4 Median pyrotag lengths were, 262, 287, and 433 base pairs in the datasets generated using the primer 27F, 534R, and 27F-534R, respectively.

### Community composition of NHP fecal bacteria

**Figure 1:**
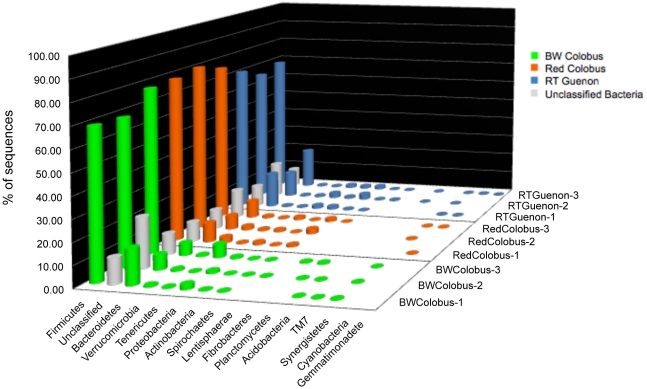
Relative abundance of phylum members of fecal bacteria from nine wild primate subjects. Ribosomal Database Project classifier (v.10.2; 70% confidence threshold) was used for sequence assignment. Sequence abundance from fecal samples of black-and-white colobus subjects are green; red colobus subjects are red; red-tailed guenon subjects are blue, and unclassified bacteria abundance are represented by grey color bars. The analysis is based on 534R dataset.

The RDP Classifier [28], v10.2, at 70% Bayesian bootstrap cut-off was used to assign sequences to phylotypes. We observed a common pattern of phyla distribution for very diverse communities across all three NHP species in that only a few phyla were in high abundance as shown in Figure 1 (and Figures S4–S5). Not surprisingly, Firmicutes occupied the largest portion (65–79%) of the genomic sequences, followed by Bacteroidetes (5.5–19.3%). Among the rare but common to all subjects were Verrucomicrobia (0.3–2.6%), Tenericutes (0.07–6.1%), Proteobacteria (0.6–2.2%), Actinobacteria (0.03–1.3%), and Spirochaetes (0.02–2.6%). In addition, sequences matching Lentisphere and Synergistates phylotypes were present in the majority of subjects, albeit in minor abundance. Notably, TM7 and Planctomycetes phyla were detected only in black-and-white colobus subjects. Despite having high bacterial diversity, red colobus subjects did not reveal any Fibrobacteres, Actinobacteria, and Planctomycetes phylotypes while they were identified in at least one black-and-white colobus and red-tailed guenon. The relative abundance of unclassified bacteria ranged from 8.5%–23.5%, the highest being in black-and-white colobus subject 2 (Figure 1).

**Figure 2:**
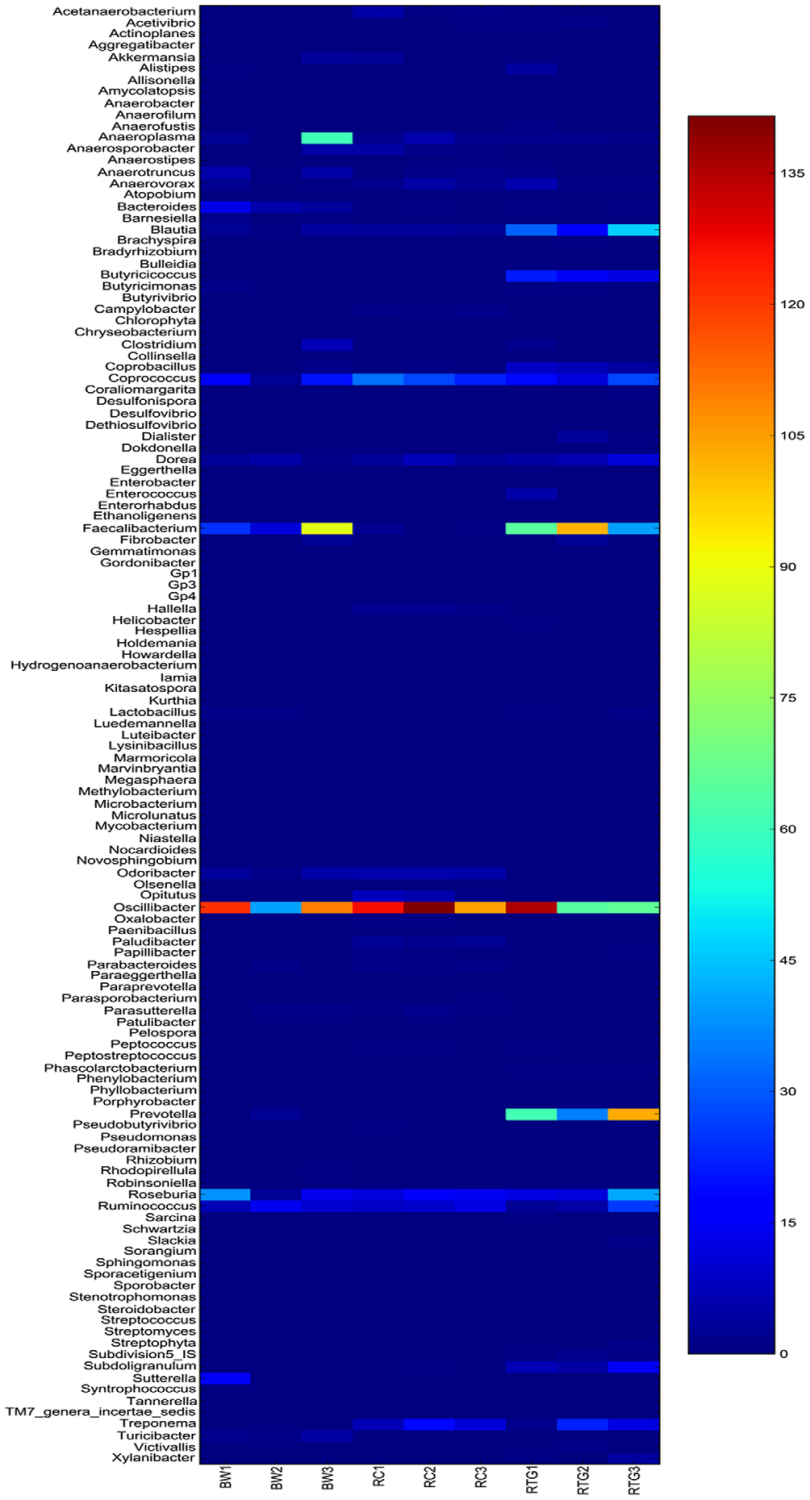
Heatmap display of the relative abundance of genera. (534R dataset). The color spectrum represents abundance of each genus per thousand sequences; dark blue representing minimum relative abundance and dark red representing maximum relative abundance. The genera are shown in alphabetical order. Each subject is shown at the bottom of the figure; BW1-BW3: Black and white colobus subjects; RC1-RC3; Red colobus subjects, and RTG1-RTG3: Red-tailed guenon subjects.

In addition to the phylum level analysis, we also looked at relative abundance of genera in each fecal sample using RDP Classifier at 70% Bayesian bootstrap cut-off (Figure 2; the heatmap standardized as per thousand sequences); *Ruminococcus, Roseburia, Oscillabacter, Anaerovorax, Novosphingobium, Caprococcus, Parabacteroides, Blautia, Faecalibacterium, Subdoligranulum, Anaerotruncus, Anaeroplasma,* and *Dorea* were present in all nine fecal samples, though in variable abundance. Of these genera *Oscillabacter, Roseburia, Ruminococcus*, *Faecalibacterium,* and *Caprococcus* showed the highest abundance (Figure 2 and Figures S6–S7). We observed the following genera in at least one subject of the three NHP species, but not in all nine subjects: *Hespellia, Paipillibacter, Alistipes, Acetivibrio, Victivallis, Butyricicoccus, Caprobacillus, Campylobacter, Anaeroflium, Prevotella, Butyrivibrio, Oxalobacter,* and *Treponema.* Also, the following genera were conspicuously absent in all three subjects of one NHP species though present in others; *Peptococcus* was not recovered from black-and-white samples, *Lactobacillus* from red colobus samples, and *Odoribacter* from red-tailed guenon (Figure 2).

### Human and non-human primate fecal microbiome is largely species-specific

**Figure 3:**
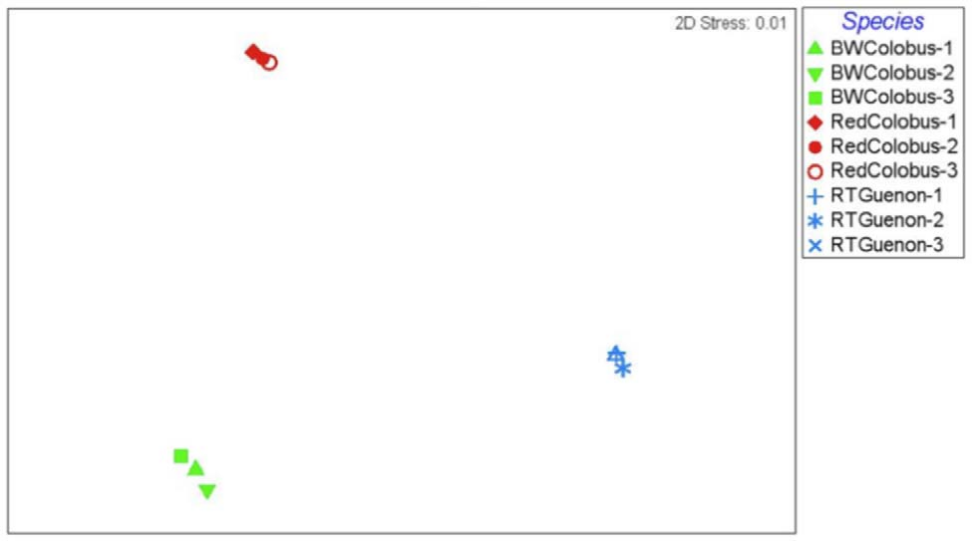
Multi Dimensional Scale analysis of the microbiomes based on the 534R dataset. non-metric Multi Dimensional Scale analysis is an ordination method used to visually assess variation in patterns of microbial diversity. Each data point represents 16S rRNA data from a single primate subject; Black and white colobus subjects, red-colobus subjects, and red-tailed guenon subjects are designated by symbols in green, red, and blue colors, respectively. The Bray-Curtis similarity was used to rank the distances calculated using the community data.

Bray-Curtis similarity indices calculated based on taxonomic clustering of sequences at 97% similarity from each sample revealed that different individuals of the same species harbor similar microbial communities (Figures 3–5). All three non-human primate species inhabit the same environment in Kibale National Park, Uganda. The observation that subjects from the same species clustered together and distinctly from other species with distinct microbiomes, suggests that while diet may play a role, species-specific factors are also important. Both of the colobine species are extremely folivorous (80–90%) and incorporate very little fruit into their diet, thus there is considerable overlap in their diets [29]. In contrast, the redtail guenons are frugivorous/omnivorous, incorporating a substantial proportion of fruit in their diet, supplemented with flowers, insects, seeds, and leaves [30]. If diet were the only determinant of microbial community compositions, then the red and black and white colobus should have overlapped considerably. However, this is not what we found.

**Figure 4 pone-0013963-g004:**
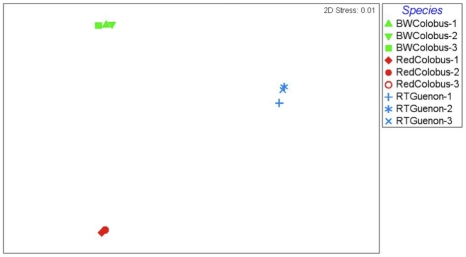
Multi Dimensional Scale analysis of the microbiomes based on the 27F dataset. Each data point represents 16S rRNA data from a single primate subject; Black and white colobus subjects, red-colobus subjects, and red-tailed guenon subjects are designated by symbols in green, red, and blue colors, respectively. The Bray-Curtis similarity was used to rank the distances calculated using the community data.

**Figure 5:**
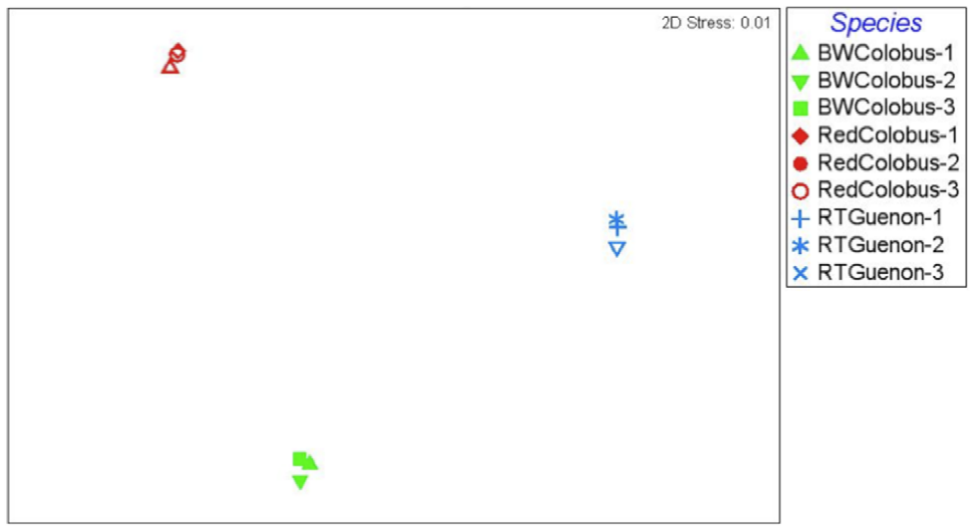
Multi Dimensional Scale analysis of the microbiomes based on the (27F-534R) dataset. Black and white colobus subjects, red-colobus subjects, and red-tailed guenon subjects are designated by symbols in green, red, and blue colors, respectively. The Bray-Curtis similarity was used to rank the distances calculated using the community data.

**Figure 6:**
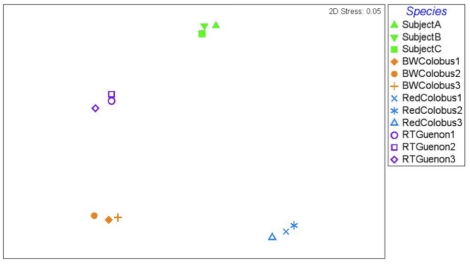
Multi Dimensional Scale analysis of the microbiomes based on the enriched (27F-534R) dataset. The analysis was based on the (27F-534R) dataset, which include human stool data. Human subjects (A, B, C) were previously described elsewhere [31]. Black and white colobus subjects, red-colobus subjects, red-tailed guenon subjects, and human subjects are designated by symbols in green, orange, blue, and purple colors, respectively. The Bray-Curtis similarity was used to rank the distances calculated using the community data.

In addition to the above analysis, we compared (Figure 6) the fecal microbial communities of the NHPs included in this study with microbial communities found in stool samples of healthy human subjects (n = 3), described in [31]. We trimmed the near full length 16S rRNA sequences generated from human stool samples [31] down to the 27F-534R regions before aligning with the 27F-534R dataset (median sequence length = 433 bp) generated in this study. We chose the 27F-534R dataset for comparison because the number of sequences per human subject (A, B, C, 617, 662, 1060, respectively) was comparable to the 27F-534R-dataset (292–1563, per subject). Unexpectedly, human subjects, like the NHP species, showed high within-species similarity and clustered together, indicating that human fecal microbiota are distinct from those of other primates.

Even though primate fecal microbiomes appear to be distinct and species specific per Bray-Curtis similarity index, we investigated how many taxa were actually shared between subjects and also the number of taxa that are unique to each subject. Using mothur (get.sharedseqs, list and group files at 0.03 similarity cutoff) we found shared and unique OTUs as listed in Table 3. Strikingly, 25–63% of OTUs clustered at 97% similarity cutoff are unique to subjects regardless of the species. This finding indicates that even though fecal microbiomes appear to be species-specific, each subject harbors a considerable portion of unique OTUs. Curiously, human subjects shared few OTUs with colobine monkeys, which are foregut fermenters and predominantly folivorous, while more OTUs were common between human and red-tailed guenon, both of which are hindgut fermenters and tend to eat a broader range of foods.

**Table 3:** Number of shared OTUs for each pairwise comparison.

	A	B	C	BWC1	BWC2	BWC3	RC1	RC2	RC3	RTG1	RTG2	RTG3
A	**(51/49)**	27	41	0	0	0	0	0	0	7	7	3
B		**(87/62)**	40	1	0	1	0	0	0	7	7	5
C			**(77/54)**	0	1	1	0	0	0	4	7	3
BWC1				**(137/44)**	76	140	3	1	5	14	17	9
BWC2					**(33/25)**	77	1	0	3	5	5	6
BWC3						**(169/48)**	4	5	12	14	12	8
RC1							**(131/36)**	158	190	0	0	0
RC2								**(146/35)**	233	0	0	1
RC3									**(334/51)**	0	0	0
RTG1										**(154/50)**	103	97
RTG2											**(312/63)**	119
RTG3												**(184/52)**
# OTUs	104	140	143	311	132	351	364	423	653	311	497	355

The number of OTUs unique to each subject and the corresponding percent values are given in parenthesis. (A, B, and C are Human subjects. BWC- Black and White Colobus, RC-Red Colobus, RTG-Red-tailed Guenon). The numbers below were calculated using ‘get.sharedseqs’ command in mothur software.

Furthermore, we compared bacterial communities found in NHP fecal samples with that of human stool samples at the genus level using RDP Classifier (Figure S8, relative abundance expressed as per thousand sequences). *Oscillibacter, Faecalibacterium, Ruminococcus, Roseburia, Coprococcus,* and *Blautia* were recovered from all NHP fecal samples and human stool samples. The genus *Bacteroides* was markedly overrepresented in all three samples from human subjects whereas this genus was hardly detected in NHP samples (Figure S8), which may be a result of comparatively shallow sequencing depth in the 27F-534R dataset. Similarly, *Catenibacterium, Escherichia/Shigella, Eubacterium, Lactococcus, Succiniclasticum,* and *Veillonella* were observed in human samples but not in NHP samples in this dataset. Notably, some genera in human samples were also present in high abundance in samples only from red-tailed guenon such as *Prevotella, Subdoligranulum,* and *Butyricicoccus.*

## Discussion

There are only a few previously reported culture-independent studies on fecal microbiota of non-human primates [21], [32]–[36] leading to only limited comparative data available on the intestinal microbiota of primates either in captivity or in the wild, and none of these other studies employed high-throughput pyrosequencing. A recent report [32] focused on fecal microbiota of wild chimpanzees from Tanzania using Terminal Restriction Fragment Length Polymorphism (T-RFLP); interestingly nine out of twelve subjects showed highly similar microbiomes. In terms of within-species similarity of fecal microbiota, our results agree with this finding. These authors [32] also reported the presence of Mollicutes in the fecal samples of chimpanzees. We observed *Anaeroplasma,* a genus in the class Mollicutes (a more recent addition to the phylum Tenericutes) in all NHP subjects, suggesting Mollicutes, including potential human pathogens, are commonly found in the primate gastrointestinal tract. In another study [34] the authors employed T-RFLP to characterize fecal microbiota from wild gorillas. Their studies did not recover members of the *Proteobacteria,* whereas all species in this study harbor OTUs belonging to the *Proteobacteria* taxa at relatively in high abundance. Fecal microbiome of wild and captive chimpanzees was characterized in recent study [32] using PCR-based methods such as temperature gradient gel electrophoresis and amplified ribosomal DNA restriction analyses (ARDRA) of the gene encoding the bacterial 16S rRNA. This study revealed that *Clostridium, Lactobacillus,* and *Bifidobacterium* are common intestinal bacteria for both captive and wild chimpanzees. Our analysis revealed no hits to the human probiotic bacterium, *Bifidobacterium*, while *Lactobacillus* was present in very low abundance. Since primates need to ferment dietary sugars, these sugar-fermenting bacterial species may have functional substitutes in the primate species targeted by our study. Notably, the majority (9/13) of chimpanzee subjects harbored a *Prevotella* like bacterium. Chimpanzees are omnivorous like humans and red-tailed guenons and may utilize proteolytic bacteria to digest proteins derived from other animals on which they prey.

Our results provide compelling evidence that, in addition to diet, primate gastrointestinal microbiomes are functionally linked to their vertebrate host taxa, and are perhaps species-specific or population-specific. Although the number of primate species included in this study is limited, the fact that all subjects originated from the same location (Kibale National Park, Uganda) strengthens our conclusion that primate microbiomes are host-specific, in that differences in microbiomes observed among species cannot be accounted for solely by habitat differences. Moreover, sampling primate species of different families, including two closely related colobine species and a cercopithecine (red-tailed guenon), suggests an evolutionary dimension. Our analyses of human samples offer additional evidence for species- or taxonomic-specificity of microbiomes.

On the other hand, we also observed a significant number of OTUs unique to each individual across all subjects (ranged 25–63% in NHP; 49–62% in human subjects, Table 3). Temporal variation of the microbial community found in fecal matter is expected and may partially account for the observed abundance of unique OTUs. Still, this argument does not negate the hypothesis that core sets of phylotypes exist and have either been adopted by hosts from the same habitat, or have evolved together with the host, as indicated by the high proportions of within-species microbial richness. In fact, in a recent report [37] on deep sequencing of fecal microbiomes from human twins identified common phylotypes shared by twins in spite of the lack of abundantly represented shared phylotypes.

Our results across all three datasets broadly define three categories of microbial populations associated with fecal samples of NHP: Firmicutes and Bacteriodes in high abundance, other phyla types that are in relatively low abundance, and unclassified bacteria. The abundance of Firmicutes and Bacteroidetes is concordant with the previously published results on fecal microbiomes of NHPs [32]–[36] and healthy humans [31], [38]. Interestingly, we observed large variations in the abundance of Bacteroidetes phylotypes (ranged 5.6–19.3%) across all species of NHP as shown in Figure 1. Large variation in the abundance of Bacteroidetes was previously reported for human and non-human primates [31], [37], which suggests colonization dynamics of the members of this particular phylum is wide-ranging among hosts. Unclassified bacterial abundance in this study ranged 8.9% to 23.5% in 534R-generated dataset (Figure 1) while the range was higher in the 27F dataset (12.5–32.9%, Figure S4). Multiple factors may play a role in this observation, at the technical level this may include the fact that the primer 27F is degenerate at two nucleotide positions or that pyrosequencing technology currently generates noisy data and is still evolving [24], [27], [39]

Overall, our results suggest that dietary preferences, gut habitat, and other host-specific factors contribute to shaping of the structure of NHP gastrointestinal microbial communities [40]. Colobines are distinct from red-tailed guenon in that both species are folivorous (primarily leaf eaters) foregut fermenters while the hindgut fermenting red-tailed guenon has a greater flexibility in its diet, which includes mostly fruits (frugivores) but also vertebrate and invertebrate prey [41]. Conceivably, some phyla could be shared between different host species with similar diets or digestive physiologies. Our analysis showed that human subjects shared several OTUs with red-tailed guenon (up to 7 OTUs) while only one or no OTUs were shared with colobines (Table 3). RDP Classifier analysis revealed *Prevotella, Subdoligranulum,* and *Butyricicoccus* were consistently overrepresented in all samples from human subjects and red-tailed guenon and across all datasets. Natural habitats of *Prevotella* sp. include the rumen and hindgut of cattle and sheep, and humans where they help the breakdown of protein [42] and carbohydrate foods [43] although some species of this genus are known to be opportunistic pathogens to humans [44]. *Subdoligranulum* is a recently characterized genus [45] in studies of human microbiota and is in the Ruminococcaceae family. *Butyricicoccus* is a butyrate-producing organism, which can ferment dietary polysaccharides. Both *Subdoligranulum* and *Butyricicoccus* are members of the *Clostridium leptum* supra-generic rRNA phylogenetic cluster.

In summary, we have demonstrated that the microbiomes of three NHP species and of humans show a much higher similarity within the same primate species than among different primate species, even when habitats overlap between NHP species. This surprising degree of host-specificity might suggest that patterns of primate evolution may have influenced the structure of primate microbiomes, although ecological similarlity between members of the same species could also account for this result. Further studies of the microbiomes of non-human primates in other locations should help determine the relative effects of phylogeny versus ecology in shaping the primate gastrointestinal microbiome. Nonetheless, our data significantly contributes to the understanding of the phylogenetic composition of primate microbiota, and thus provides a framework for comparative microbiome analysis between human and NHPs in the context of evolution.

## Materials and Methods

### Study species and their ecology

Individual NHP species (n = 3) included in this study, their distribution, habitat, and diet preferences are shown in Table 1. All samples (n = 9) were collected from Kibale National Park, Uganda as described elsewhere [46]. The fecal samples (n = 9) from the 3 species were collected as part of a larger study during behavioral observations in June and July 2005. Kibale National Park (795 km^2^) is located in western Uganda near the foothills of the Rwenzori Mountains and known for its high species diversity and density of primates. It consists primarily of moist, semi deciduous and evergreen forest and interspersed with grassland, woodland, wetlands, and colonizing forest [46]. Primates were sampled in core protected areas of Kibale National Park, relatively free from human influences. To avoid environmental contamination, only portions of primate feces not in direct contact with the ground were sampled. Samples were placed in sterile tubes and transported to the field laboratory within six hours and kept frozen (−80 C). Concurrent, detailed dietary studies were conducted on all three species in this location (Wasserman and Chapman 2003, Lambert 2002).

### Genomic DNA extraction

The frozen samples were first thawed on ice and homogenized in cold, sterile phosphate buffered saline. Subsequent steps included lysozyme incubation (20 mM Tris-HCl at pH 7.4, 100 mM EDTA, 50 mM NaCl, 0.2% Tween), freeze-thaw cycling, proteinase-K incubation, addition of 10% SDS, protein precipitation using 5M NaCl and incubation on ice, RNAse treatment, and phenol-chloroform extraction. DNA concentration and purity was estimated by gel electrophoresis and spectrometry (NanoDrop, ND-2000, ThermoScientific, DE).

### Bacterial 16S rRNA PCR amplification and Titanium Pyrosequencing

The 16S rRNA genes were amplified in four replicates using bar-coded primers (27F: 5′-AGAGTTTGAT**YM**TGGCTCAG-3′, and 534R: 5′-ATTACCGCGGCTGCTGG-3′). Each 25-µL PCR reaction contained platinum super mix (Invitrogen Cat. No. 11306-016), 3.0 mM MgCl_2,_ 200 µM of each dNTP (Promega, Madison, WI), 0.2 µM of each primer (Integrated DNA Technologies, Coralville, IA). PCR was performed on a Bio-Red thermocycler (DNA Engine) and included the following cycling steps: 3 min at 95°C, 20–25 cycles of 40 sec at 94°C, 30 sec at 60°C, 45 sec at 72°C; and a final 10 min extension. No template controls were included in all steps of the process. PCR products from each extracted DNA sample were pooled, run on 1.5% agarose gel, excised and purified using Qiagen gel extraction kit columns (Qiagen, Valencia, CA). The purified PCR products were then sent to J. Craig Venter Institute (JCVI) for pyrosequencing using GS FLX Titanium series reagents (454 Life Sciences, Roche Diagnostics, Branford, CT). The amplicons generated from all 9 fecal samples (samples collected from 3 different subjects per species) were each sequenced in both directions using the primers 27F and 534R, which resulted in three data sets; the 27F dataset, 534R dataset, and the dataset including bi-directional spanning 27F-534R.

### Data analysis

Bioinformaticians at the JCVI performed preliminary quality checks, sorting, and trimming of the sequences. We then used in-house python scripts to bin sequences generated with each primer, which also included sequences harboring both primers. Sequences without any primers were excluded from the analysis. We then concatenated sequences from nine files and transferred to a directory for analysis using mothur software [47]. A cluster computer (housed in the Institute for Genomic Biology) was used for all Linux platform applications. Preprocessing of unaligned sequences included removing short sequences <200 nt, all sequences containing ambiguous characters, and sequences with more than 6 homopolymers. We subsequently aligned the sequences using the align.seqs command embedded in mothur [48] against Greengenes' Core Set, which is used by NAST alignment tool [49]. We further removed sequences that did not align over the same span of nucleotide positions. Potential chimeric sequences were detected and removed by using chimera.slayer incorporated into mothur software (http://www.mothur.org/wiki/Chimera.slayer). Chimera analysis showed 2.1% and 3.0% of all sequences in the alignment of 27F and 534R datasets were potential chimeric sequences. The analysis did not detect any chimeras in the 27F-534R dataset. All community diversity parameters were then calculated as described in the software manual (www.mothur.org). To observe the distribution of phylotypes across each primate sample pyrosequencing reads were run through RDP classifier (v10.2, 70% bootstrap threshold). We used in-house python scripts to process Classifier output and to create heat maps. Primer V6 (PRIMER-E, Plymouth) was used to calculate Bray-Curtis similarities, to perform non-metric multi dimensional analysis (nMDS), and Analysis of Similarities (ANOSIM).

### Ethics Statement

No human subjects were included in this study. We included data from a published work [31], whose authors declared in the supplemental file of their publication that “The study was approved by the Research Ethics Board of the University of Manitoba and the Stanford University Administrative Panel on Human Subjects in Medical Research, and all participants signed informed consent.” The use of non-human primates in research: Prior to sample collection, all animal work was approved by the Institutional Animal Care and Use Committee of the University of Illinois (IACUC approval number 07229). Because samples were collected entirely non-invasively from free-living primates, steps to ameliorate suffering in accordance with the recommendations of the Weatherall report, “The use of non-human primates in research,” were not relevant to this study.

## Supporting Information

Figure S1 Rarefaction analysis of 16S rRNA sequences generated using 27F primer. Operational Taxonomical Units (OTUs) were defined at 97% sequence similarity. Blue, red, and green color curves show black and white colobus, red colobus, and red-tailed guenon subjects, respectively.
(0.11 MB TIF)

Figure S2 Rarefaction analysis of 16S rRNA sequences generated using 534R primer. Operational Taxonomical Units (OTUs) were defined at 97% sequence similarity. Blue, red, and green color curves show black and white colobus, red colobus, and red-tailed guenon subjects, respectively.
(2.51 MB TIF)

Figure S3 Rarefaction analysis of 16S rRNA sequences generated in 27F-534R data set. Operational Taxonomical Units (OTUs) were defined at 97% sequence similarity. Blue, red, and green color curves show black and white colobus, red colobus, and red-tailed guenon subjects, respectively.
(2.79 MB TIF)

Figure S4 Relative abundance of phylum members of fecal bacteria in 27F-534R data set. Ribosomal Database Project classifier (v.10.2; 70% confidence threshold) was used for sequence assignment. Sequence abundance from fecal samples of black-and-white colobus subjects are green, red colobus subjects are red,red-tailed guenon subjects are blue, and unclassified bacteria abundance are represented by grey color bars.
(2.38 MB TIF)

Figure S5 Relative abundance of phylum members of fecal bacteria in 27F data set. Ribosomal Database Project classifier (v.10.2; 70% confidence threshold) was used for sequence assignment. Sequence abundance from fecal samples of black-and-white colobus subjects are green, red colobus subjects are red, red-tailed guenon subjects are blue, and unclassified bacteria abundance are represented by grey color bars.
(2.20 MB TIF)

Figure S6 Heatmap display of the relative abundance of genera in each of the nine wild primate fecal samples (27F data set). The color spectrum represents abundance of each genus per thousand sequences. The genera are shown in alphabetical order. BW-Black-and-white colobus; RC:Red colobus, RTG:Red-tailed guenon.
(2.06 MB TIF)

Figure S7 Heatmap display of the relative abundance of genera in 27F-534R data set. The color spectrum represents abundance of each genus per thousand sequences. The genera are shown in alphabetical order. BW-Black-and-white colobus; RC:Red colobus, RTG:Red-tailed guenon.
(1.19 MB TIF)

Figure S8 Heatmap display of the relative abundance of genera in each of the nine wild primate fecal samples and human subjects as described in Eckburg et al. (2005). The color spectrum represents abundance of each genus per thousand sequences. The genera are shown in alphabetical order. BW-Black-and-white colobus; RC:Red colobus, RTG:Red-tailed guenon; A,B,C-human subjects.
(1.17 MB TIF)

Sequence Data S1 Representative sequences selected from the data set generated using the 27F primer.
(4.05 MB RTF)

Sequence Data S2 Representative sequences selected from the data set generated using the 534R primer.
(2.43 MB TXT)

Sequence Data S3 Representative sequences selected from the 27F-534R data set.
(1.14 MB TXT)
