# Supplementary figures and images for: Characterization of the Fecal Microbiome from Non-Human Wild Primates Reveals Species Specific Microbial Communities
Source: PLoS One. 2010 Nov 12;5(11):e13963. doi: 10.1371/journal.pone.0013963 (PMC2980488; doi:10.1371/journal.pone.0013963)

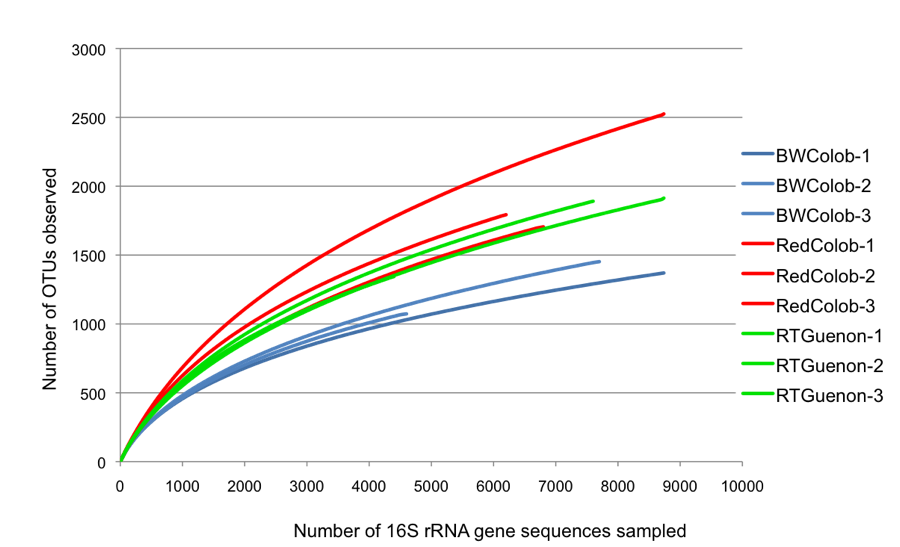

Supplement: Figure S1 — Rarefaction analysis of 16S rRNA sequences generated using 27F primer. Operational Taxonomical Units (OTUs) were defined at 97% sequence similarity. Blue, red, and green color curves show black and white colobus, red colobus, and red-tailed guenon subjects, respectively. (0.11 MB TIF) [file pone.0013963.s001.tif]

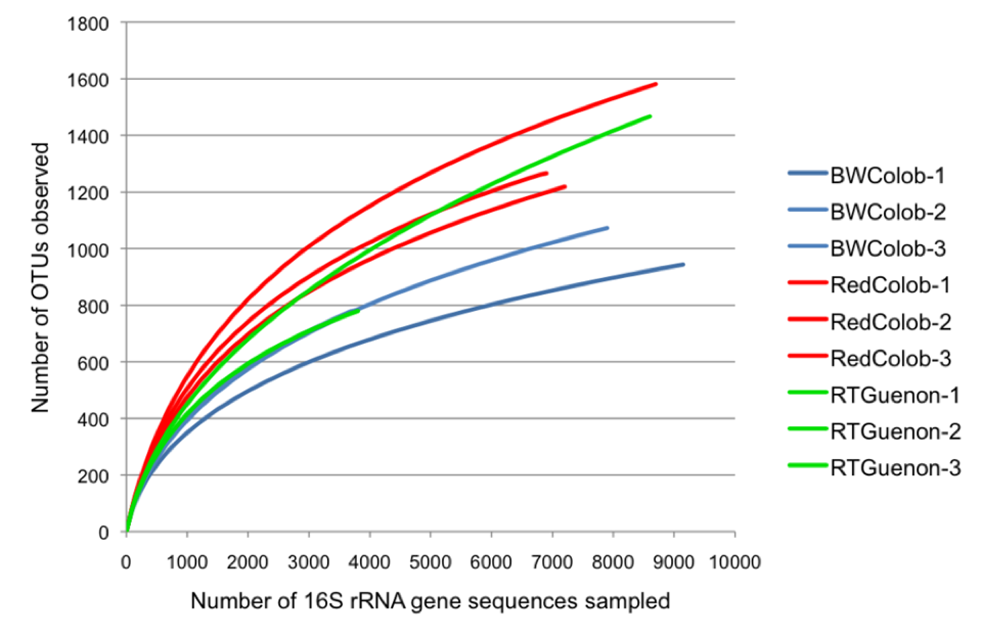

Supplement: Figure S2 — Rarefaction analysis of 16S rRNA sequences generated using 534R primer. Operational Taxonomical Units (OTUs) were defined at 97% sequence similarity. Blue, red, and green color curves show black and white colobus, red colobus, and red-tailed guenon subjects, respectively. (2.51 MB TIF) [file pone.0013963.s002.tif]

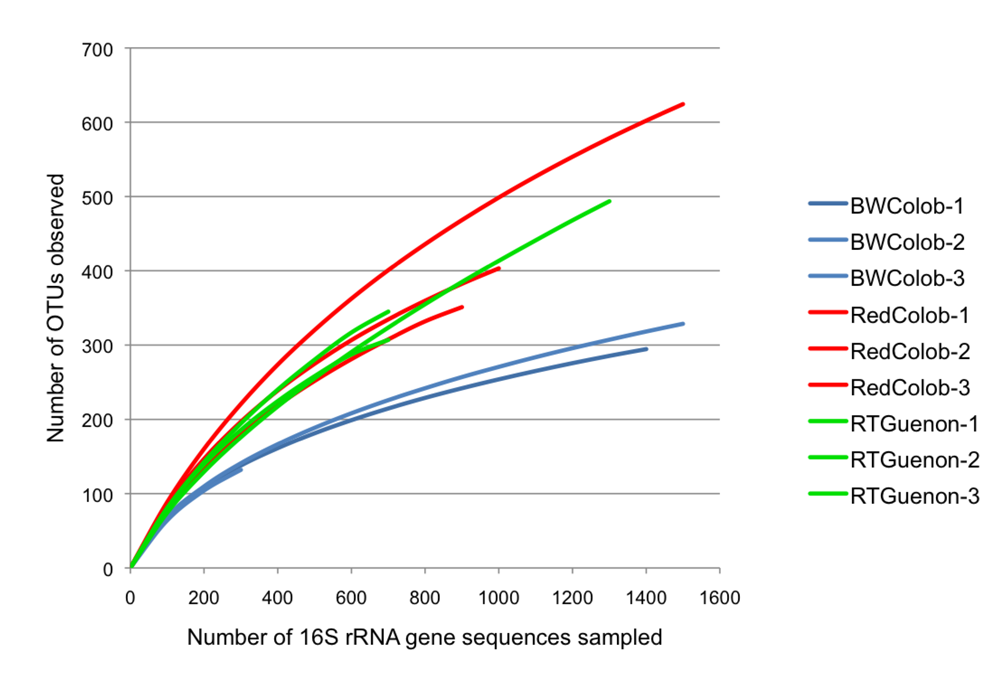

Supplement: Figure S3 — Rarefaction analysis of 16S rRNA sequences generated in 27F-534R data set. Operational Taxonomical Units (OTUs) were defined at 97% sequence similarity. Blue, red, and green color curves show black and white colobus, red colobus, and red-tailed guenon subjects, respectively. (2.79 MB TIF) [file pone.0013963.s003.tif]

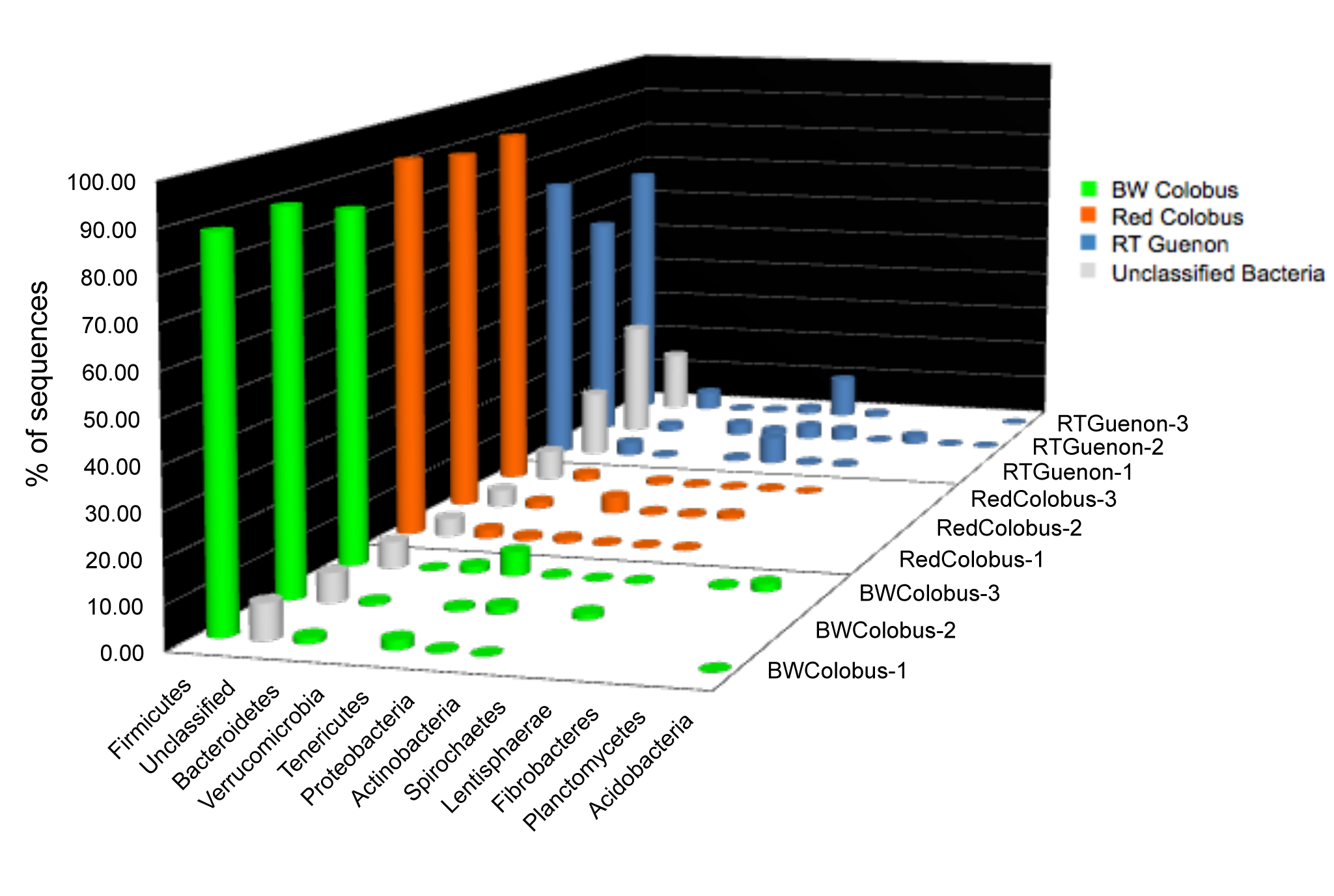

Supplement: Figure S4 — Relative abundance of phylum members of fecal bacteria in 27F-534R data set. Ribosomal Database Project classifier (v.10.2; 70% confidence threshold) was used for sequence assignment. Sequence abundance from fecal samples of black-and-white colobus subjects are green, red colobus subjects are red,red-tailed guenon subjects are blue, and unclassified bacteria abundance are represented by grey color bars. (2.38 MB TIF) [file pone.0013963.s004.tif]

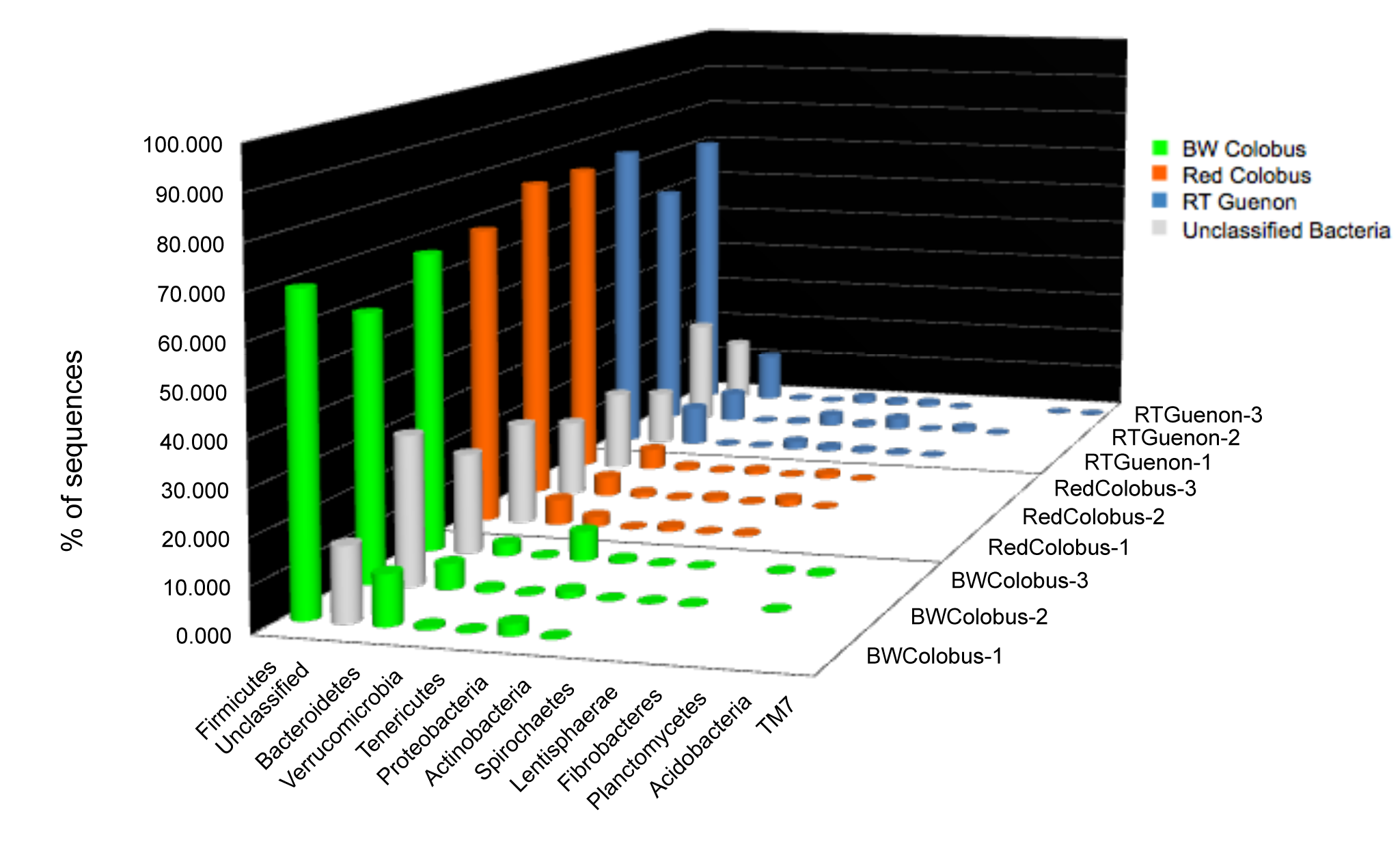

Supplement: Figure S5 — Relative abundance of phylum members of fecal bacteria in 27F data set. Ribosomal Database Project classifier (v.10.2; 70% confidence threshold) was used for sequence assignment. Sequence abundance from fecal samples of black-and-white colobus subjects are green, red colobus subjects are red, red-tailed guenon subjects are blue, and unclassified bacteria abundance are represented by grey color bars. (2.20 MB TIF) [file pone.0013963.s005.tif]

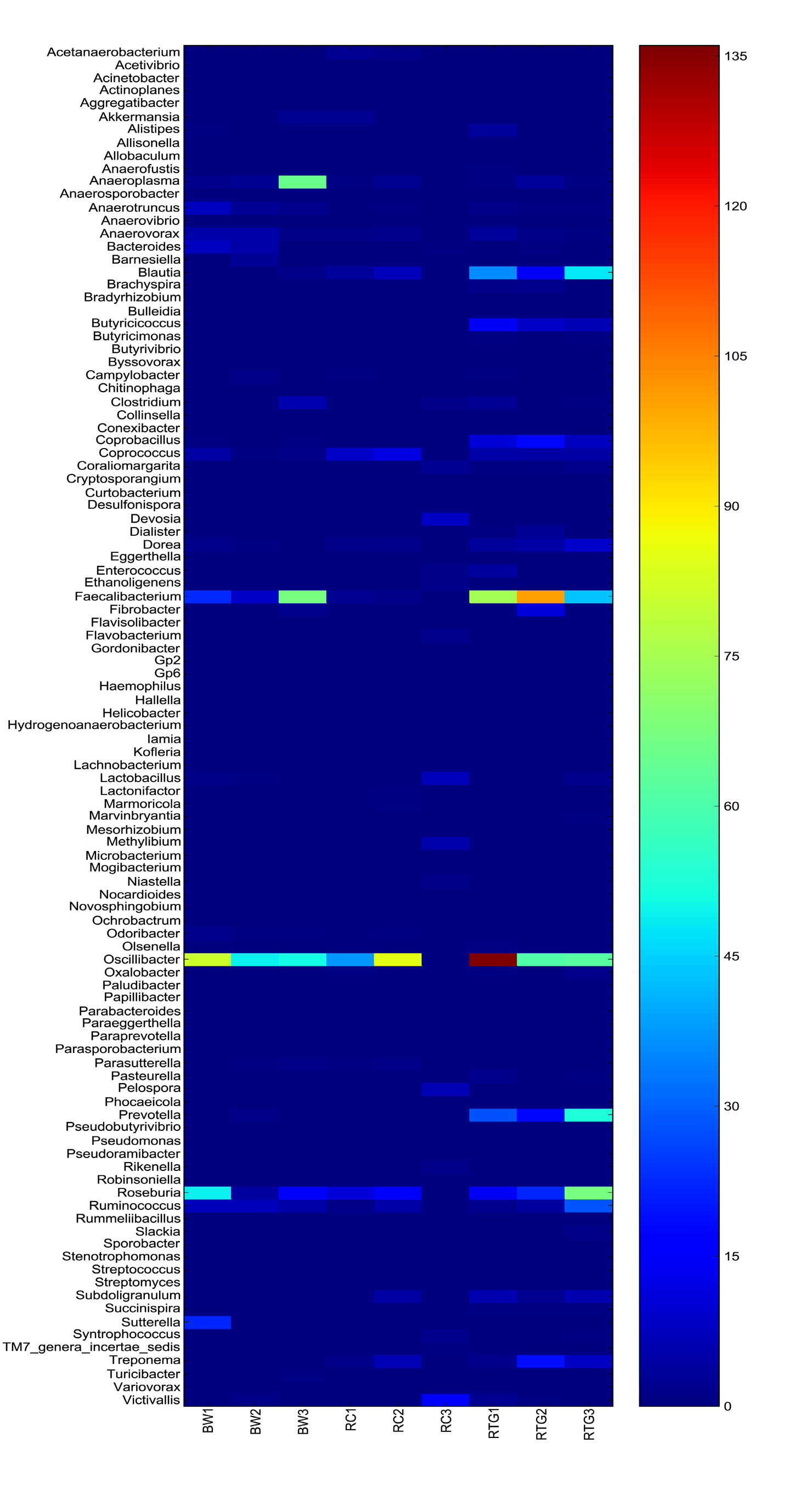

Supplement: Figure S6 — Heatmap display of the relative abundance of genera in each of the nine wild primate fecal samples (27F data set). The color spectrum represents abundance of each genus per thousand sequences. The genera are shown in alphabetical order. BW-Black-and-white colobus; RC:Red colobus, RTG:Red-tailed guenon. (2.06 MB TIF) [file pone.0013963.s006.tif]

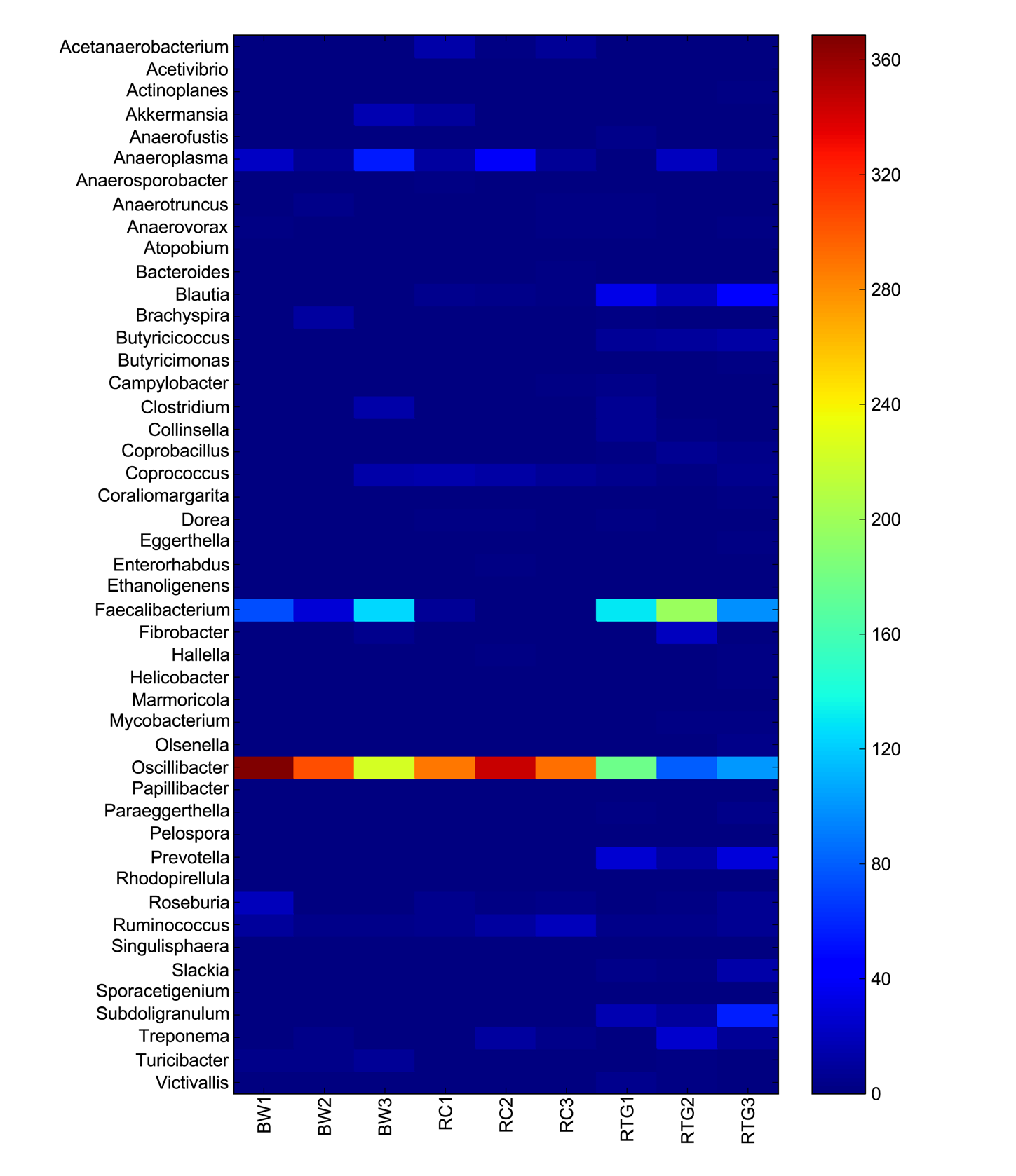

Supplement: Figure S7 — Heatmap display of the relative abundance of genera in 27F-534R data set. The color spectrum represents abundance of each genus per thousand sequences. The genera are shown in alphabetical order. BW-Black-and-white colobus; RC:Red colobus, RTG:Red-tailed guenon. (1.19 MB TIF) [file pone.0013963.s007.tif]

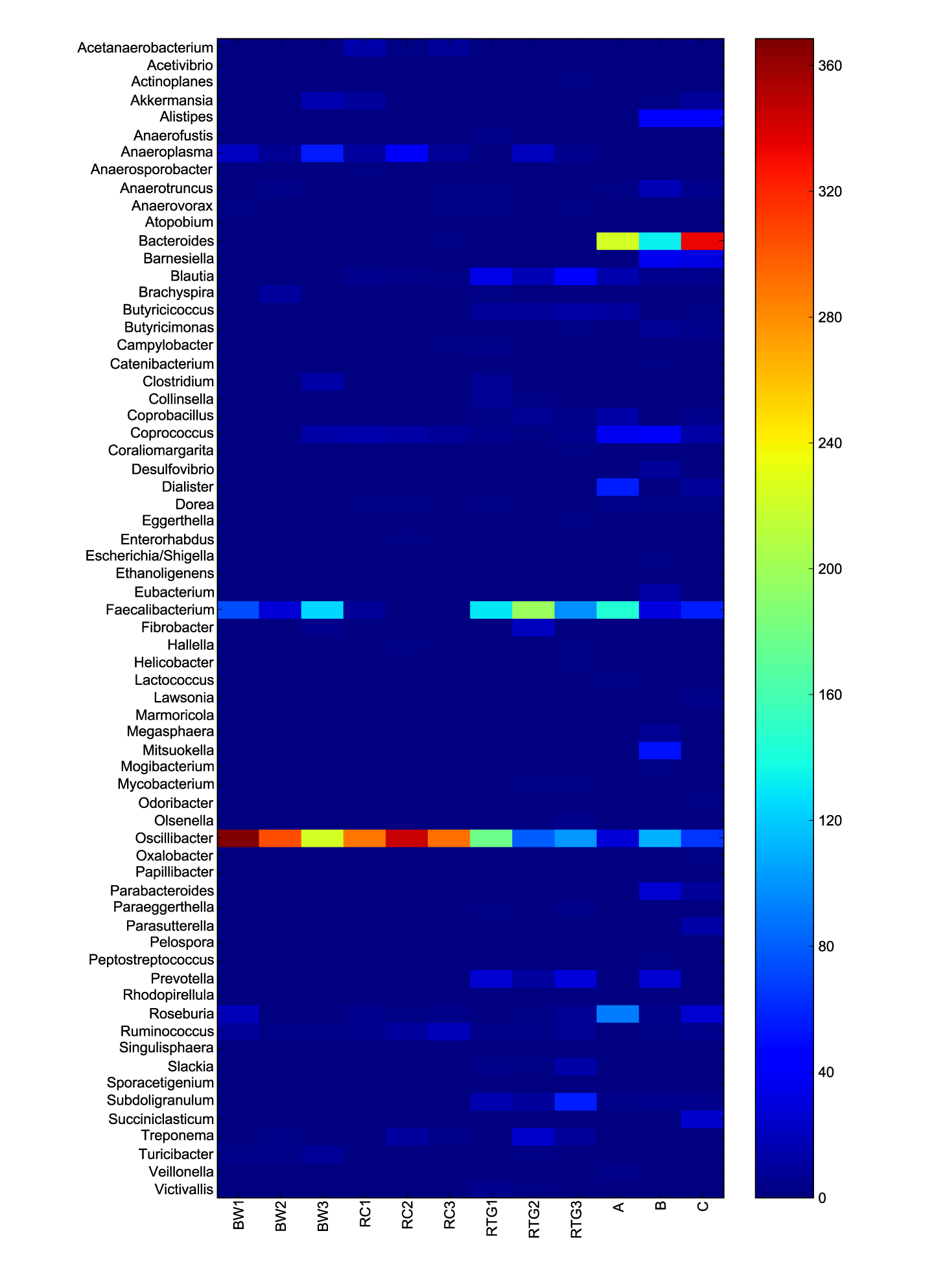

Supplement: Figure S8 — Heatmap display of the relative abundance of genera in each of the nine wild primate fecal samples and human subjects as described in Eckburg et al. (2005). The color spectrum represents abundance of each genus per thousand sequences. The genera are shown in alphabetical order. BW-Black-and-white colobus; RC:Red colobus, RTG:Red-tailed guenon; A,B,C-human subjects. (1.17 MB TIF) [file pone.0013963.s008.tif]
