# Supplementary material for: Characterization of the Fecal Microbiome from Non-Human Wild Primates Reveals Species Specific Microbial Communities
Source: PLoS One. 2010 Nov 12;5(11):e13963. doi: 10.1371/journal.pone.0013963 (PMC2980488; doi:10.1371/journal.pone.0013963)
Supplement: Sequence Data S1 — Representative sequences selected from the data set generated using the 27F primer. (4.05 MB RTF) [file pone.0013963.s009.rtf]

>FQH3XDB01BRD9P|1|1
GATGAACGCTGGCGGCATGCCTAAGACATGCAAGTCGAACGAAGGAACCCAAGGAGAATTATGGAAGTTTGGAGAGCTTGCTCAAAGAACGGAATAATCAAATTGGATCATTCCTTAGTGGCAAACGGGTGAGTAACACGTGGGTTACCTACCTTCAAGATGGGGATAACAGTTGGAAACGATTGCTAATACCGAATGTGATCTACGGATTAAAGAAGCTCCAAAAGCTTCGCTTGAAGATGGGCCTGCGGTGCATTAGCTAGTTGGTGGATAATGGCCTACAAGGCACGATGCATAGCCGAACTGAGAGGTCAAA
>FQH3XDB01EJHO5|2|2
GATGAACGCTGGCGGCATGCCTAAGACATGCAAGTCGAACGAAGGAACCCAAGGAGAATTATGGAAGTTTGGAGAGCTTGCTCAAAGAACGGAAATAAATCAAATTGGATCATTCCTTAGTGGCAAACGGGTGAGTAACACGTGGGTTACCTACCTTCAAGATGGGGATAACAGTTGGAAACGATTGCTAATACCGAATGTGATCTACGGATTAAAGAAGCTCCAAAGCTTCGCTTGAAGATGGGCCTGCGGTGCATTAGCTAGTTGGTGGGATAATGGCCTACAAGGCGACGATGCAT
>FQH3XDB01D4QVU|3|25
GACGAACGCTGGCGGCGTGCCTAACACATGCAAGTCGAACGGGAATTAAGAGCTTGCTTTTAATTTTAGTGGCGAACGGGTGAGTAAAGCGTGAGTAACCTGCCTAATGGAGGGGGATAACGTCCGGAAACGGATGCTAATACCGCATGGAGTGCGAGATCCGCATGGATGAGCGTACGAAAGGAGAGATCCGCCGATAGATGGGCTCACGTCCGATTAGTTAGTTGGTGGGGTAAAAGGCCTACCAAGGCAGCGATCGGTAGCCGGGCTGAGAGGCTGAACGGCCACATTGGGACTGAGACACGGCCCAGACTCCTA
>FQH3XDB01C0ISK|4|1
GACGAACGCTGGCGGCACGCCTAACACATGCAAGTCGAACGGAGAACCTTCGGGTTCTTAGTGGCGGACGGGTGAGTAACACGTGAGCAACCTGCCTCTCAGAGGGGGATAACGTTTGGAAACGAACGCTAATACCGCATAACATATTGAAGTCGCATGGCTTTGATATCAAAGGAGCAATCCGCTGAGAGATGGGCTCGCGTCCGATTAGATAGTTGGCGGGTAACGGCCGACCAAGTCGACGATCGGTAGCCGGACTGAGAGGTTGATCGGCCACATTGGGACTGAGACACGGCCCAGACTCCTACGGGAGGCAGCAGTGGGGGATATTGGCACAATGGGGGAAACCCTGATGCAGCGATGCCGCGTGAATGATGACGGTCTCGGATTGTAAAGTTCTGGTCGTTAGGGGACGATAATGACAGTACCC
>FQH3XDB01BGVJO|5|1
GACGAACGCTGGCGGCACGCCTAACACATGCAAGTCGAACGGAGAATTTAGGTTTACTTGAATTCTTAGTGGCGGACGGGTGAGTAACACGTGAGCAACCTGCCTTTGAGAGAGGAATAATTTCTGGAAACGGATTGCTAATACCGGATGTACTCTACGGAGTAAAGAAGCCTTTAAAGCTTCGCTTAGAGATGGGCCTGCGGTGCATTAGCTAGTTGGCAGGATAACGGCCTACCAAGGCGACGATGCATAGCCGAACTGAGAGGTTAATCGGCCACACTGGGACTGAGACACGGCCCAGACTCCTACGGGA
>FQH3XDB01CN7H5|6|1
GACGAACGCTGGCGGCACGCCTAACACATGCAAGTCGAACGGAGAATTTAGGTTTACTTGAATTCTTAGTGGCGGACGGGTGAGTAACACGTGAGCAACCTGCCTTTGAGAGAGGAATAATTTCTGGAAACGGATTGCCTAATACCGGATGTACTCTACGGAGTAAAGAGCCTTTAAAGCTTCGCTTAGAGATGGGCCTGCGGTGCATTAGCTAGTTGGCAGGATAACGGCCTACCAAGGCGACGATGCATAGCCGAACTGAGAGGTTAATC
>FQH3XDB01CXVGX|7|1
GACGAACGCTGGCGGCACGCCTAACACATGCAAGTCGAGCGGAGATTAAGAGCTTGCTCTTATGATCAGCGGCGGACGGGTGAGTAACACGTGAGCAACCTGCCTTTCAGAGGGGGACAACAGTTGGAAACGACTGCTAATACCGCATAAGCCCACGACCCGGCATCGGGTTGAGGGAAAAGGAGCAATCCGCTTTGAGATGGCCTCGCGTCCGATTAGCTAGTTGGTGAGGTAATGGCCCACCAAGGCGACGATCGGTAGCCGGACTGAGAGGTTGAACGGCCACATTGGGACTGAGACACGGCCCAGACTCCTACGGGAGGCAGCAGTGGGGAATATTGCACAATGGGGAAACCCTGATGCAGCGACGCCGCGTGGAGGAAGAAGGTCTTCGGATTGTAAACTCCGTGTTGTTGGAGGTAAGATAATGGACGGTA
>FQH3XDB01B7AN9|8|1
GACGAACGCTGGCGGCACGCTTAACACATGCAAGTCGAACGAGCGATGAGAGCTTGCTCTCAAAGCGAGTGGCGGACGGGTGAGTAACACGTGAGCAACCTGCCTTTAAGAGGGGAATAACGTTTGGAAATGATTGCTAATGCCGGATGAAAATGGGAAACGATACGTCATTTTTTATAAAAGGTGACCTTTAAAGCATCGCTTGAAGATGAGCCTGCGTCGTATTAGTTAGTGGTGGGTAACGCCTACCAAGACGACGATGCGTAGCCGACCTGAGAGGGTGATCGGCCACACTGGGACTGAGACACGGCCCAGACTCC
>FQH3XDB01AQKVP|9|1
GACGAACGCTGGCGGCACGCTTAACACATGCAAGTCGAACGAGCGATGAGAGCTTGCTCTCAAAGCGAGTGGCGGACGGGTGAGTAACACGTGAGCAACCTGCCTTTAAGAGGGGAATAACAGTTGGAAACGATTGCTAATACCGAATGTGCTCTACGGAGTAAAGAAGCCTTTAAAGCTTCGCTTGGAGATGGGCCTGCGGCGCATTAGCTAGTTGGTGGGTAATGGCCTACCAAGGCAACGATGCGTAGCCGAACTGAGAGGTTGATCGGCCACATTGGGACTGAG
>FQH3XDB01CU249|10|8
AACGAACGCTGGCGGCAGGCTTAACACATGCAAGTCAAGGGGGTGTAGCAATACACAACCGGCGCACGGGTGAGTAACGCGTGGGAATATGTCCATTTGTGGGGGATAGCTTCTGGAAACGGAAGGTAATACCGCATAAGCCCCTGAGGGGGAAAGATTTATCGCGAATGGAGTGGCCCGCGTTGGATTAGTTAGTTGGTTAGGTAAAGGCTGACCAAGGCGAAGATCCATAGCTGGTCTGAGAGGACGATCAGCCACATTGGGGACTGAGACACGGCCCAGACTCCTACGGGAGGCAGCAGTAAGGAATATTGGACAATGGGGGCAACCCTGATCCAGCCATGCCGCGTGAGTGAAGAAGGCCCTATGGGTTAGTAAAACTCTGTTGTAGTGGGAAGAACGGCCATATAAGGAAATGTATAGTGGAGTGACGGTACCATACC
>FQH3XDB01CQF09|11|1
GACGAACGCTGGCGGCATGCCTTACACATGCAAGTCGAACGGCAGCGCGGGAGCTTGCTCCTGGCGGCGAGTGGCGAACGGGTGAGTAATACATCGGAACGTGTCCGTTTGTGGGGGACAACCAGCCGAAAGGTTGGCTAATACCGCATAAGACCTGAGGGTGAAAGCCGGGACCGCAAGGCCTGGCGCAGACGGAGCGGCCGATGATTGATTAGCGTGGTTGGCGGGTAAGGCCACCAAGGCGACGATCAATAGCTGGTCTGAGAGGACGACCAGCCACACTGGAACTGAGACACGGT
>FQH3XDB01COZBJ|12|49
GACGAACGCTGGCGGCGCGCCTAACACATGCAAGTCGAACGAGCGATGGAGAGCTTGCTTTCCAGAGCGAGTGGCGAACGGGTGAGTAACGCGTGAGGAACCTGCCTCAAAGAGGGGGACAACAGTTGGAAACGACTGCTAATACCGCATAAGCCCACGACCCGGCATCGGGTTGAGGGAAAAGGAGTAATCCGCTTTGAGATGGCCTCGCGTCCGATTAGCTAGTTGGTGAGGTAACGGCCCACCAAGGCGACGATCGGTAGCCGGACTGAGAGGTTGAACGGCCACATTGGGACTGAGACACGGCCCAGACTCCTACGGGAGGCAGCAGTGGGGAATATTGCACAATGGGGGAAACCCTGATGCAGCGACGCCGCGTGGAGGAGAAGGTCTCGGATTGTAAACTCCTGTTGTTGGGAAG
>FQH3XDB01B8FQX|13|16
GACGAACGCTGGCGGCGCGCCTAACACATGCAAGTCGAACGAGAGACAGAGAGCTTGCTTTCTGAATCGAGTGGCGAACGGGTGAGTAACGCGTGAGGAACCTGCCTCAAAGAGGGGGACAACAGTTGGAAACGACTGCTAATACCGCATAAGCCCCACGACCCGGCATCGGGTTGAGGGAAAAGGAGCAATCCGCTTTGAGATGGCCTCGCGTCCGATTAGCTAGTTGGTGAGGTAACGGCCCACCAAGGCGACGATCGGTAGCCGGACTGAGAGGTTGAACGGCCACATTGGGACTGAGACACGGCCCAGACTCCTACGGGAGGCAGCAGTGGGGAATATTGCACAAGTGGGGGAAACCCTGATGCAGCGACGCC
>FQH3XDB01DMG9N|14|13
GACGAACGCTGGCGGCGCGCCTAACACATGCAAGTCGAACGAGTGACAGAGAGCTTGCTCTCTGAAGCGAGTGGCGAACGGGTGAGTAACGCGTGAGGAACCTGCCTCAAAGAGGGGGACAACAGTTGGAAACGACTGCTAATACCGCATAAGCCCACGGACCGGCATCGGTCTGAGGGAAAAGGAGCAATCCGCTTTGAGATGGCCTCGCGTCCGATTAGCTAGTTGGTGAGGTAACGGCCACCAAGGCGACGATCGGTAGCCGGACTGAGAGGTTGAACGGCCACATTGGGACTGAGACACGGCCCAGACTCCTACGGGAGGCAGCAGTGGGGAATATTGCACAATGGGGGAAACCCTGATGCAGCGACGCCGCGTGGAGGAAGAAGGTCTTCGGATTGTAAACTCCTAGTTGTTGAGAGATATGACGGTACTCAA
>FQH3XDB01C7RVS|15|23
GACGAACGCTGGCGGCGCGCCTAACACATGCAAGTCGAACGAGCGAGAGAGAGCTTGCTTTCTTGAGCGAGTGGCGAACGGGTGAGTAACGCGTGAGGAACCTGCCTCAAAGAGGGGGACAACAGTTGGAAACGACTGCTAATACCGCATAAGCCCACGACCCGGCATCGGGTTGAGGGAAAAGGAGCAATCCGCTTTGAGATGGCCTCGCGTCCGATTAGCTAGTTGGTGAGGTAATGGGCCCACCAAGGCGACGATCGGTAGCCGGACTGAGAGGTTGAACGGCCACATTGGGACTGAGACACGGCCCCAGACTCCTACGGGAGGCAGCAGTGGGGAATATTGCACAATGGGGGAAACCCTGATGCAGCGACGCCGCGTGGAGGAAGAAGGTCTTCGGGATTGGTAAAACTCCT
>FQH3XDB01CXFYN|16|5
GACGAACGCTGGCGGCGCGCCTAACACATGCAAGTCGAACGAGCGAGAGAGAGCTTGCTTTCTCAAGCGAGTGGCGAACGGGTGAGTAACGCGTGAGGAACCTGCCTCAAAGAGGGGGACAACAGTTGGAAACGACTGCTAATACCGCATAAGCCCACGGCTCGGCATCGAGCAGAGGGAAAAGGAGCAATCCGCTTTGAGATGGCCTCGCGTCCGATTAGCTAGTTGGTGAGGTAACGGCCCACCAAGGCGACGATCGGTAGCCGGACTGAGAGGTTGAACGGCCACATTGGGACTGAGACACGGCCCAGACTCCTACGGGAGGCAGCAGTGGGGAATATTGCACAATGGGGGAAACCCTGATGCAGCGACGCCGCGTGGAGGAAGAAGGTCTTCGGATTGGTAAAACTCCTGTTGTTGAGGAAGATAATGACGGTACTCAACAAGGAAGTGAACGGCTAACTACGTGCCAGCAGCC
>FQH3XDB01D04YT|17|2
GACGAACGCTGGCGGCGCGCCTAACACATGCAAGTCGAACGGAGCGAGAGAGAGCTTGCTTTCTCAAGCGAGTGGCGAACGGGTGAGTAACGCGTGAGGAACCTGCCTCAAAGAGGGGGACAACAGTTGGAAACGACTGCTAATACCGCATAAGCCCACGGGTCGGCATCGACCTGAGGGAAAAGAGCAATCCGCTTTGAGATGGCCTCGCGTCCGATTAGCTGGTTGGTGAGGTAACGGCCCACCAAGGCGACGATCGGTAGCCGGACTGAGAGGTTGAACGGCCACATTGGGACTGAGACACGGCCCAGACTCCTACGGGAGGCAGCAGTGGGGAATATTGCACAATGGGGGAAACCCTGATGCAGCGACGCCGC
>FQH3XDB01D97UI|18|4
GACGAACGCTGGCGGCGCGCCTAACACATGCAAGTCGAACGAGCGAGAGAGAGCTTGCTTTCTTGAGCGAGTGGCGAACGGGTGAGTAACGCGTGAGGAACCTGCCTCAAAGAGGGGGACAACAGTTGGAAACGACTGCTAATACCGCATAAGCCCACGATCCGGCATCGGATTGAGGGAAAAGGAGCAATCCGCTTTGAGATGGCCTCGCGTCCGATTAGCTGGTTGGTGAGGTAACGGCCCACCAAGGCGACGATCGGTAGCCGTACTGAGAGGTAGAA
>FQH3XDB01DAYPD|19|37
GACGAACGCTGGCGGCGCGCCTAACACATGCAAGTCGAACGAGCGATGGAGAGCTTGCTTTCCAAAGCGAGTGGCGAACGGGTGAGTAACGCGTGAGGAACCTGCCTCAAAGAGGGGGACAACAGTTGGAAACGACTGCTAATACCGCATAAGCCCACGACCCGGCATCGGGGTTGAGGGAAAAGGAGCAATCCGCTTTGAGATGGCCTCGCGTCCGATTAGCTAGTTGGTGAGGTAATGGCCCACCAAGGCGACGATCGGTAGCCGGACTGAGAGGTTGAACGGCCACATTGGGACTGAGACACGGCCCAGACTCCTACGGGAGGCAGCAGTGGGGAATATTGCACAAGTGGGGAAACCCTGATGCAGCGACGCC
>FQH3XDB01EDGSP|20|2
GACGAACGCTGGCGGCGCGCCTAACACATGCAAGTCGAACGAGCGATGGAGAGCTTGCTTTCCAGAGCGAGTGGCGAACGGGTGAGTAACGCGTGAGGAACCTGCCTCAAAGAGGGGGGACAACAGTTGGAAACGACTGCTAATACCGCATAAGCCCACGGGTCGGCATCGATCTGAGGGAAAAGGAGCAATCCGCTTTGAGATGGCCTCGCGTCCGATTAGCTAGTTGGTGAGGTAACGGCCCACCAAGGCGACGATCGGTAGCCGGACTGAGAAGTTGAACGGCCACATTGGGACTGAGACACGGCCCAGACTCCGTACGGGAGGCAGCAGGTGGGGAATATTGCACGAAGTCGGGGAAACCCTGATGCAGCGACGCCGCGTGGAGAGAGGT
>FQH3XDB01C8HVQ|21|8
GACGAACGCTGGCGGCGCGCCTAACACATGCAAGTCGAACGAGCGAGGAGGAGCTTGCTTCTCCGAGCGAGTGGCGAACGGGTGAGTAACGCGTGAGGAACCTGCCTCAAAGAGGGGGACAACAGTTGGAAACGACTGCTAATACCGCATAAGCCCACGACCCGGCATCGGGTTGAGGGAAAAGGAGCAATCCGCTTTGAGATGGCCTCGCGTCCGATTAGCTAGTTGGTGAGGTAACGGCCCACCAAGGCGACGATCGGTAGCCGGACTGAGAGGTTGAACGGCCACATTGGGACTGAGACACGGCCCCAGACTCCTACGGGAGGCAGCAGTGGGGAATATTGCACAATGGGGGAAAACCCTGATGCAGCGACGCCGCGTGGAGGAAGAAGGTCTTCGGATTGTAAACTCCTGTTGTTGGGGAAGATAATGACGGTACCAACAAGGAAGTGACGGCTAACTACGTGCCAGCAGCCGC
>FQH3XDB01AHHWA|22|1
GACGAACGCTGGCGGCGCGCCTAACACATGCAAGTCGAACGAGCGAGGAGAAGCTTGCTTCTCTAAGCGAGTGGCGAACGGGTGAGTAACGCGTGAGGGACCTGCCTCAAAGAGGGGGACAACAGTTGGAAACGACTGCTAATACCGCATAAGCCCACGACCCGGCATCGGGCTGAGGGAAAAGGAGCAATCCGCTTTGAGATGGCCTCGCGTCCGATTAGCTAGTTGGTGAGGTAACGGCCCACCAAGGCGACGATCGGTAGCCGGACTGAGAGGTTGAACGGCCACATTGGGACTGAGACACGGCCCAGACCTCCTACGGGAGGCAGCAGTGAGG
>FQH3XDB01CUNOT|23|1
GACGAACGCTGGCGGCGCGCCTAACACATGCAAGTCGAACGAGCGATGAGGAGCTTGCTTCTCAAAGCGAGTGGCGAACGGGTGAGTAACGCGTGAGGAACCTGCCTCAAAGAGGGGGACAACAGTTGGAAACGACTGCTAATACCGCATAAGCCCACGACCCGGCATCGGGTAGAGGGAAAAGAGCAATCCGCTTTGAGATGGCCTCGCGTCCGATTAGCTAGTTGGTGAGGTAACGGCCCACCAAGGCGACGATCGGTAGCCGGACTGAGAGGTTGAACGGCCACATTGGGACTGAGACA
>FQH3XDB01BYXWX|24|8
GACGAACGCTGGCGGCGCGCCTAACACATGCAAGTCGAACGAGCGAAGAGGAGCTTGCTTCTCTAAGCGAGTGGCGAACGGGTGAGTAACGCGTGAGGAACCTGCCTCAAAGAGGGGGACAACAGTTGGAAACGACTGCTAATACCGCATAAGCCCACGACCCGGCATCGGGGTTGAGGGAAAAGGAGTGATCCGCTTTGAGATGGCCTCGCGTCCGATTAGCTAGTTGGTGAGGTAATGGCCCACCAAGGCGACGATCGGTAGCCGGACTGAGAGGTTGAACGGCCACATTGGGACTGAGACACGGCCCAGACTCCTACGGGAGGCAGCAGTGGGGAATATTGCACAATGGGGGAAACCCTGATGCAGCGACGCCGCGTGGAGGAAGAAGGTCTTCGGATTGTAAACTCC
>FQH3XDB01BNQPY|25|36
GACGAACGCTGGCGGCGCGCCTAACACATGCAAGTCGAACGAGCGAAGAGGAGCTTGCTTCTCTGAGCGAGTGGCGAACGGGTGAGTAACGCGTGAGGAACCTGCCTCAAAGAGGGGGACAACAGTTGGAAACGACTGCTAATACCGCATAAGCCCCACGACCCGGCATCGGGTTGAGGGAAAAGGAGCAATCCGCTTTGAGATGGCCTCGCGTCCGATTAGCTAGTTGGTGAGGTAACGGCCCACCAAGGCGACGATCGGTAGCCGGACTGAGAGGTTGAACGGCCACATTGGGACTGAGACACGGCCCAGACTCCTACGGGAGGCAGCAGTGGGGAATATTGCACGAAGTGGGGGAAACCCTGATGCAGCGACGCCGCGTGAAGGAA
>FQH3XDB01A0LRN|26|1
GACGAACGCTGGCGGCGCGCCTAACACATGCAAGTCGAACGAGCGAAGAGGAGCTTGCTTCTCTGAGCGAGTGGCGAACGGGTGAGTAACGCGTGAGGAACCTGCCTCAAAGAGGGGACAACAGTTGGAACGACTGCTAATACCGCATAAGCCACGACCGGCATCGGGTTGAGGGAAAGGAGCGATCCGCTTTGAGATGGCCTCGCGTCCGATTAGCTAGTTGGTGAGGTAACGGCCACCAAGGCGACGATCGGTAGCCGGACTGAGAGGTTTGAACGGCCACATTGGGACTGAGGACACGGCCCAGGACTCCTACGGGAGGCAGCAGTGGGGAA
>FQH3XDB01CVTGM|27|1
GACGAACGCTGGCGGCGCGCCTAACACATGCAAGTCGAACGAGCGAAGAGGAGCTTGCTTCTCTGAGCGAGTGGCGAACGGGTGAGTAACGCGTGAGGAACCTGCCTCAAAGAGGGGGACAACAGTTGGAAACGACTGCTAATACCGCATAAGCCCACGGGATCGCATGATTCTGAGGGAAAAGGAGCAATCCGCTTTGAGATGGCCTCGCGTCCGATTAGCTAGTTGGTGAGGTAACGGCCCACCAAGGCGACGATCGGTAGCCGGACTGAGAGGTTGAACGGCCACATTGGGACTGAGACACGGCCCAGACCTCCGTACGGGGAGGCCAGCAGTGGGAATATTGCACGAAGTGGGGGAAACCCTGATGCAGCGAC
>FQH3XDB01DU4YS|28|14
GACGAACGCTGGCGGCGCGCCTAACACATGCAAGTCGAACGAGCGAAGAGAAGCTTGCTTTTCTGAGCGAGTGGCGAACGGGTGAGTAACGCGTGAGGAACCTGCCTCAAAGAGGGGGACAACAGTTGGAAACGACTGCTAATACCGCATAAGCCCACGGCCCGGCATCGGGTTGAGGGAAAAGGAGTGATCCGCTTTGAGATGGCCTCGCGTCCGATTAGCTAGTGGTGAGGTAACGGCCCACCAAGGCGACGATCGGTAGCCGGACTGAGAGGTTGAACGGCCACATTGGGACTGAGACACGGCCCAGACTCCTACGGGAGGCAGCAGTGGGGAATAGTTGCACAAGTGGGGGAAACCCTGATGCAGCGACG
>FQH3XDB01EQ6QL|29|2
GACGAACGCTGGCGGCGCGCCTAACACATGCAAGTCGAACGAGTGATGGGGAGCTTGCTCTCCAAAGCGAGTGGCGAACGGGTGAGTAACGCGTGAGGAACCTGCCTCAAAGAGGGGGACAACAGTTGGAAACGACTGCTAATACCGCATAAGCCCACGGACCGGCATCGGTCTGAGGGAAAAGGAGCAATCCGCTTTGAGATGGCCTCGCGTCCGATTAGCTAGTTGGTGAGGTAACGGCCCACCAAGGCGACGATCGGTAGCCGGACTGAGAGGTTGAACGGCCACATTGGGACTGAGACACGGCCCAGACTCCTACGGGAGGCAGCAGGTGGGAATATTGCACAAGTAGGGGGAAACCCTGATGCAGCGACGCG
>FQH3XDB01ARDOA|30|1
GACGAACGCTGGCGGCGCGCCTAACACATGCAAGTCGAACGGAGTTGGGAGGAGCTTGCTCTTCTTAACTTAGTGGCGAACGGGTGAGTAACGCGTGAGTAACCTGCCCTAGAGTGGGGGACAACAGTTGGAAACGACTGCTAATACCGCATAAGCCCACGATCCGGCATCGGATTGAGGGAAAAGGATTTATTCGCTTTAGGATGGACTCGCGTCCAATTAGCTAGTTGGTGAGGTAACGGCCCACCAAGGCGACGATTGGTAGCCGGACTGAGAGGTTGAACGGCCACATTGGGGACTGAGACACGGCCCCAGGACTCCTACGGGAGGCAGCAG
>FQH3XDB01B7EGS|31|1
GACGAACGCTGGCGGCGCGCCTAACACATGCAAGTCGAACGGAGTTTATGTTTCAGAACCCTTCGGGGGACGAAACATAAACTTAGTGGCGGACGGGTGAGTAACACGTGAGCAACCTGCCTTTCAGAGGGGAATAACGTTTGGAAACGAACGCTAATACCGCATAACGTATTTTGATGGCATCGTCGAAATACCAAAGGAGCAATCCGCTGAAAGATGGGCTCGCGTCCGATTAGATAGTTGGTGAGGTAACGGCCCACCAAGTCGACGATCGGTAGCCGGACTGAGAGGTTGAACGGCCACATTGGGACTGAGACACGGCCCAGACTCCTACGGGAGGCAGCAGTGAGGGATATTGGTCAAGTGGGGGAAAACCCTGAACCAGCGACGCCGCGTGAGGGAAGACGGTTTTCGGATTGTAAACCTCTGTC
>FQH3XDB01DSRIX|32|1
GACGAACGCTGGCGGCGCGCCTAACACATGCAAGTCGAAACGAGCGAGAGAGAGCTTGCTTTCTTGAGCGAGTGGCGAAACGGGTGAGTAACGCGTGAGGAACCTGCCTCAAAGAGGGGGACAACAGGTTTGGAAAACGACTGCTAATACCGCATAAGCCCACGATCCGGCATCGGATTGAGGGAAAAGGAGCAATCCGGCTTTGAGATGGCCTCGCGTCCGATTAGCTAGTTGGTGAGGTAACGGCCCACCAAGGCGACGATCGGTAGCCGGACTGAGAGGTT
>FQH3XDB01DNE4S|33|1
GACGAACGCTGGCGGCGTGCCTAACACATGCAAGTCGAACGAAGTTTTAAGGAGCTTGCTCTTTAAAACTTAGTGGCGGACGGGTGAGTAACGCGTGAGCAACCTGACTCTCAGAGGGGGGATAACGTTTTGAAAAGACGCTAATACCGCATTAATATATCGGAAACCGCATGATTCTGATATCAAAAGGAGCAATCCGCTGAGAGATGGGGCTCGCGTCCGATTAGTTAGTTGGTGAGGTAACGGCTCACCAAGACTACGATCGGTAGCCGGACTGAGAGGTTGATCGGCCACATTGGGGACTGAGACACGGGTCCCCAGG
>FQH3XDB01EF8A8|34|48
GACGAACGCTGGCGGCGTGCCTAACACATGCAAGTCGAACGAAGTTTTAAGGAGCTTGCTCTTTAAAACTTAGTGGCGGACGGGTGAGTAACGCGTGAGCAACCTGCCTCTCAGAGGGGGATAACGTTTTGAAAAGAACGCTAATACCGCATAACATATCGGAACCGCATGATTCTGATATCAAAGGAGCAATCCGCTGAGAGATGGGCTCGCGTCCGATTAGTTAGTTGGTGAGGTAACGGCTCACCAAGACTACGATCGGTAGCCGGACTGAGAGGTTGATCGGCCACATTGGGACTGAGACACGGCCCAGACTCCTACGGGAGGCAGCAGTGGGGATATTGCGCAATGGGGGAAAACCCCTGACGCAGCAACGCCGCGTGAAGG
>FQH3XDB01DPN19|35|9
GACGAACGCTGGCGGCGTGCCTAACACATGCAAGTCGAACGAAGTTTTAAGGAGCTTGCTCTTTAAAACTTAGTGGCGGACGGGTGAGTAACGCGTGAGCAACCTGCCTCTCAGAGGGGGATAACGTTTTGAAAAGAACGCTAATACCGCATAACATATCGGAACCGCATGATTCTGATATCAAAAGGAGCAATCCGCTGAGAGATGGGCTCGCGTCCGATTAGTTAGTTGGTGAGGTAACGGCTCACCAAGACTACGATCGGTAGCCGGACTGAGAGGTTGATCGGCCACATTGGGACTGAGACACGGCCCAGACTCCTACGGGAGGCAGCAGTGGGGATATTGCGCAAGTGGGGGAAACCCTGACGCAGCAAC
>FQH3XDB01A4721|36|6
GACGAACGCTGGCGGCGTGCCTAACACATGCAAGTCGAACGGAGTTATGAAGAAGCTTGCTTCAGATTAACTTAGTGGCGGACGGGTGAGTAACGCGTGAGCAACCTGCCTTTCAGAGGGGAATAACACAGTGAAAACTGTACTAATACCGCATAACGTGTTTAGTTCGCATGAACTGAACACCAAAGGAGCAATCCGCTGAAAGATGGGCTCGTGTCCGATTAGATAGTTGGTGAGGTAACGGCCCACCAAGTCTGCGATCGGTAGCCGGACTGGAGAGGTTGAACGGCCACATTGGGGACTGAGATACGGCCCAGACTCCTACGGGAGCAGCAGTGAGGGATATTA
>FQH3XDB01DBBRO|37|4
GACGAACGCTGGCGGCGTGCCTAACACATGCAAGTCGAACGAAGTGTTGAGAGCTTGCTTTTAACACTTAGTGGCGGACGGGTGAGTAACGCGTGAGCAACCTGCCTTTCAGAGGGGGATAACGTTTGGAAACGAACGCTAATACCGCATGATATATTTAAACCGCATGGTTTGGATATCAAAGGAGCAATCCGCTGAAAGATGGGCTCGCGTCCGATTAGCTAGTTGGCGGATAACAGCCCACCAAGGCGACGATCGGTAGCCGGACTGAGAGGTCGAACGGCCACAGTTGGGACTGAGACACGGCCCAGA
>FQH3XDB01BH90P|38|11
GACGAACGCTGGCGGCGTGCCTAACACATGCAAGTCGAACGAAGAATCGGATTCGTCCGGTTCTTAGTGGCGGACGGGTGAGTAACGCGTGAGAAACCTGCCTTTCAGAGGGGGATAACGTTTGGAAACGAACGCTAATACCGCATGACGCAGCGGATTCGCATGAATCTGCTGCCAAAGGAGCGATCCGCTGAAAGATGGTCTCGCGTCCGATTAGCTAGTTGGTGAGATAACAGCCCACCAAGGCGACGATCGGTAGCCGGACTGAGAGGTCGAACGGCCACATTGGGACTGAGACACGGCCCAGACTCCTACGGGAGGCAGCAGTGGGGGATATTGGCACAATGGAGGAAACTCAGATGCAGCAACGCCGCGTGAGGGAAGAAGGATTTCGGTTGTAAACCTCTGTCTTCGGTGACGATAATGACGGTAGCC
>FQH3XDB01BSSUI|39|13
GACGAACGCTGGCGGCGTGCCTAACACATGCAAGTCGAACGAAGCTTTTACGGAATGCGACTTCGGTCAAGTGAAGTTTAAGCTTAGTGGCGGACGGGTGAGTAACGCGTGAGCAACCTGCCTTTCAGAGGGGGATAACGTTTGGAAACGAACGCTAATACCGCATAACATTGTTTTCCCGCATGAGAGAACAATCAAAGGAGCAATCCGCTGAAAGATGGGCTCGCGTCCGATTAGGTAGTTGGTGGGGTAACGGCCGTACCAAGCCGACGATCGGTAGCCGGACTGAGAGGTTGAACGGCCGACATTGGGACTGAGACACGGCCCAGACTCCTACGGGAGGCAGC
>FQH3XDB01DFJNE|40|82
GACGAACGCTGGCGGCGTGCCTAACACATGCAAGTCGAACGAAGCTTTTACGGAATGAGACTTCGGTCAAGTGAAGTTTAAGCTTAGTGGCGGACGGGTGAGTAACGCGTGAGCAACCTGCCTTTCAGAGGGGGATAACGTTTGGAAACGAACGCTAATACCGCATAACATTGTTTCCCGCATGAGAGAACAATCAAAGGAGCAATCCGCTGAAAGATGGGCTCGCGTCCGATTAGGTAGTTGGTGGGGTAACGGCCTACCAAGCCGACGATCGGTAGCCGGACTGAGAGGTTGAACGGCCACATTGGGACTGAGACACGGCCCAGACTCCTACGGGAGGCAGCAGTGGGGAATATTGCACAATGGGGGAAACCCTGATGCAGCAACGCCGCGTGAGGGAAGACGGTTTTCGGATTGG
>FQH3XDB01AX7FS|41|1
GACGAACGCTGGCGGCGTGCCTAACACATGCAAGTCGAACGAAGCTTTTACGGAATGAGACTTCGGTCAAGTGAAGTTTAAGCTTAGTGGCGGACGGGTGAGTAACGCGTGAGCAACCTGCCTTTCAGAGGGGGATAACGTTTGGAAACGAACGCTAATACCGCATAACATTGTTTACCCGCATGAGAGAACAATCAAAGGAGCAATCCGCTGAAAGATGGGCTCGCGTCCGATTAGGTAGTTGGTGGGGTAAACGGCCTACCAAGTCCGACGATCGGTAGCCGGACTGAGAGGTTGAACG
>FQH3XDB01DGOPY|42|2
GACGAACGCTGGCGGCGTGCCTAACACATGCAAGTCGAACGAAGCTTTTACGGAATGCGACTTCGGTCAAGTGAAGTTTAAGCTTAGTGGCGGACGGGTGAGTAACGCGTGAGCAACCTGCCTTTCAGAGGGGGGATAACGTTTGGAAACGAACGCTAATACCGCATGACATTGTTTTCCCGCATGAGAGAACAATCAAAGGAGCAATCCGCTGAAAGATGGGCTCGCGTCCGATTGGAGTTGGTGGGTAACGGCCTACAAGCCGACGATCGGTAAGCCGGACTGAGAGGTTGAACGGCCACATTGGGACTGA
>FQH3XDB01E203D|43|1
GACGAACGCTGGCGGCGTGCCTAACACATGCAAGTCGAACGAAGTTACTTTTCTTAATCCTTCGGGAAGCGGAGATTTAACTTAGTGGCGGACGGGTGAGTAACACGTGAGCAACCTGCCTCTCAGAGGGGGATACCGTTTGGAAACGAACGTTAATACCGCATAACGCAGCGGGATCGCATGATTCTGCTGCCAAAGGAGTAATCCGCTGAGAGATGGGCTCGCGTCTGATTAGGTAGTTGGTGAGGTAACGGCCCACCAAGTCGACGATCAGTAGCCGGACTGAGAGGTTGATCGGCCACATTGGGACTGAGACACGGCCCCAGACTCCTACGGGAGGCAGCAGTGGGGGATATTGCGCAATGGGGGAAACCCTGACGCAGCAACGCCGCGTGTGGGAAGAAGGTCTTCGGATTGTAAACCATTGTTCCAGGGG
>FQH3XDB01AMDIW|44|1
GACGAACGCTGGCGGCGTGCCTAACACATGCAAGTCGAACGGAGCATTGAGAGCTTGCTTTTGATGCTTAGTGGCGGACGGGTGAGTAACGCGTGAGCAACCTGCCTTTCAGAGGGGGATAACGTTTGGAAACGAACGCTAATACCGCATAACATATTTCAGTCGCATGAATGAGATATCAAAGGAGCAATCCGCTGAAAGATGGGCTCGCGTCCGATTAGCTAGTTGGCGGGATAACAGCCCACCAAGGCGACGATCGGTAGCCGGACTGAGAGGTCGATCGGCCACATTGGGACTGAGACACGGCCCAGACTCCTACGGGAGCAGCAGTGGGGGATATTGCACAATGGAGGAAACTCTGATGCAGC
>FQH3XDB01BSJQM|45|1
GACGAACGCTGGCGGCGTGCCTAACACATGCAAGTCGAACGGAAGTTAGAAGCTTGCTTTTAACTTTAGTGGCGGACGGGTGAGTAACGCGTGAGCAACCTGCCTATCAGAGGGGGATAACTGAGGGAAACCTTAGCTAATACCGCATAAAGTACAGAATTCGCATGGAAATTGTATGAAAGGAGAAATCCGCTGATAGATGGGCTCGCGTCCGATTAGCCAGTTGGTGAGGTAACGGCTCACCAAAGCGACGATCGGTAGCCGGGCTGAGAGGCTGAACGGCCACATTGGGACTGAGACACGGCCCAGACTCCTACGGGAGGCAGCAGTGGGGGATATTGCACAATGGGGGAACCCTGATGCAGCAACGCCGCGTGAGGGAAGAAGGTTTTCGGATTGGTAAAACCTCTGTCCTCAGAGAAGAAAAGAATGACGGTATCTGAGGAGGAAGCTCCGGCTAACTACGTGCCAGCAGCC
>FQH3XDB01BV46V|46|1
GACGAACGCTGGCGGCGTGCCTAACACATGCAAGTCGAACGGAGTGAAGCGCTGAAATGATGTTAGTTTACTAATGGATTTTTCTTGTTTCACTTAGTGGCGGACGGGTGAGTAACGCGTGAGGAACCTGCCCTTCAGAGGGGGACAACAGTTGGAAACGACTGCTAATACCGCATGAAGTACTTTGGGGCATCCCTAGAGTACCAAAGGAGCAATCCGCTGAAGGATGGCCTCGCGTCCGATTAGGTAGTTGGTGAGGTAACGGCTCACCAAGCCGACGATCGGTAGCCGGACTGAGAGGTTGAACGGCCACATTGGGACTGAGACACGGCCCAGACTCCTAC
>FQH3XDB01D6IIG|47|3
GACGAACGCTGGCGGCGTGCCTAACACATGCAAGTCGAACGGAGTGAAGCGCTGAAAATGATGTTAGTTTACTAATGGATTTTTCTTGTTTCACTTAGTGGCGGACGGGTGAGTAACGCGTGAGGAACCTGCCCTTCAGAGGGGGACAACAGTTGGAAACGACTGCTAATACCGCATGAAGTACTTTTGGGGCATCCCTAGAGTACCAAAGGAGCAATCCGCTGAAGGATGGCCTCGCGTCCGATTAGGTAGTTGGTGAGGTAACGGCTCACCAAGCCGACGATCGGTAGCCGGACTGAGAGGTTGAACGGCCACAGTTGGACTGAGACACGGCCCAGACCTCCGTAGGGA
>FQH3XDB01ATU41|48|11
GACGAACGCTGGCGGCGTGCCTAACACATGCAAGTCGAACGGAGCTTGATGGAATGCGACTTCGGTCAAATGAATTCAAGCTTAGTGGCGGACGGGTGAGTAACGCGTGAGCAACCTGCCTTTCAGAGGGGATAACGTTTGGAAACGAACGCTAATACCGCATGAGACTACAGAGCCGCATGGCTCAGTGGTCAAAGGAGCAATCCGCTGAAAGATGGGCTCGCGTCCGATTAGGTAGTTGGTGAGGTAGAGGCTCACCAAGCCGACGATCGGTAGCCGGACTGAGAGGTTGAACGGCCACATTGGGACTGAGACACGGCCCAGACTCCGTACGGGAGCAGCAGTGAGGG
>FQH3XDB01A4Y2I|49|3
GACGAACGCTGGCGGCGTGCCTAACACATGCAAGTCGAACGGAGCTAGAGAGCTTGCTTTCTAGCTTAGTGGCGGACGGGTGAGTAATGCGTGAGCAACCTGCCTATCAGTGGGGGATAACTTCCGGAAACGGACGCTAATACCGCATATAGTACATAATCCGCATGGATAAGTGTATGAAAAGGAGCAATCCGCTGATAGATGGGCTCACGTCCGATTAGTTAGTTGGTGAGGTTAACGGCTCACCAAGACCGCGATCGGTAGCCGGGCTGAGAGGCTGAACGGCCACATTGGGACTGAGACACGGCCCCAGACTCCTACGGGAGGCAGCAGTGGGGATACTT
>FQH3XDB01BHC47|50|5
GACGAACGCTGGCGGCGTGCCTAACACATGCAAGTCGAACGGAGTTGGAGAGCTTGCTTTTCAACTTAGTGGCGGACGGGTGAGTAAAGCGTGAGCAACCTGCCTATCAGTGGGGGATAACTTCCGGAAACGGATGCTAATACCGCATATAGTACATAATTCGCATGGATAAGTGTATGAAAGGAGCAATTTGCTGGTAGATGGGCTCACGTCCGATTAGTTAGTTGGTGGGGTAAAGGCTTACCAAGACCGCGATCGGTAGCCGGGCTGAGAGGCTGAACGGCACATTGGGACTGAGACACGGCCCAGACT
>FQH3XDB01C85W4|51|13
GACGAACGCTGGCGGCGTGCCTAACGCATGCAAGTCGAACGGAGTATTGCGCTGAACAGATGATAGCTTGCTATAGGAAGTTCTTGTGATACTTAGTGGCGGACGGGTGAGTAACGCGTGAGCAACCTGCCTTCGAGAGGGGAATAACGTTCTGAAAAGGACGCTAATACCGCATAACGTATAGAAGTCACATGGCATTTATACCAAAGATTTATCGCTCGAAGATGGGCTCGCGTCCGATTAGGTAGTTGGTGAGGTAACGGCTCACCAAGCCGACGATCGGTAGCCGGACTGAGAGGTTGAACGGCCACATTGGGACTGAGACACGGCCCAGACTCC
>FQH3XDB01ASNZB|52|4
GACGAACGCTGGCGGCGTGCCTAACACATGCAAGTCGAACGGAGTTTGTTGGAACAAGGCTTCGGCCAAGGGGAATTCAAACTTAGTGGCGGACGGGTGAGTAACGCGTGAGCAACCTGCCTTTCAGAGGGGGATAACGTTTGGAAACGAACGCTAATACCGCATGAGACTACAGAACCGCATGGTTCAGCAGTCAAAGGAGCAATCCGCTGAAAGATGGGCTCGCGTCCGATTAGGTAGTTGGTGAGGTAGAGGCTCACCAAGCCGACGATCGGTAGCCGGACTGAGAGGTTGAACGGCCACATTGGGACTGAG
>FQH3XDB01A9YQC|53|1
GACGAACGCTGGCGGCGTGCCTAACACATGCAAGTCGAACGGGAATTGAGAGCTTGCTTTTAATTCTAGTGGCGAACGGGTGAGTAAAGCGTGAGTAACCTGCCTAACGGAGGGGGATAACGTCCGGAAACGGATGCTAATACCGCATAAAGTACGGATTCCGCATGGAGAGACGTATGAAAGGAGCGATCCGCCGATAGATGGGCTCACGTCCGATTAGCTAGTTGGTGAGGTAAAGGCTCACCAAGGCAACGATCGGTAGCCGGGCTGAGAGGCTGAACGGCCACATTGGGACTGAGACACGGCCCAGACTCCTACGGGAGCAGCAGTGGGGATATTGCAC
>FQH3XDB01EYLJN|54|2
GACGAACGCTGGCGGCGTGCCTAACACATGCAAGTCGAACGGGAGATTAAAGCTTGCTTTAATCTTTAGTGGCGGACGGGTGAGTAAAGCGTGAGCAACCTGCCTATTAGAGGGGGATAACTTCCGGAAACGGATGCTAATACCGCATATAGTACATAATTCGCATGAATAAGTGTATGAAAGGAGCAATCCGCTGATAGATGGGCTCACGTCCGATTAGTTAGTTGGTGAGGTAACGGCTCACCAAGACTGCGATCGGTAGCCGGGCTGAGAGGCTGAACGGCCACATTGGGACTGAGATACGGCCCAGACTCCTACGGGAGGCAGCAGTGGGGATATTGCACAAGTGGGGGAAACCCTGATGCAGCAACGCC
>FQH3XDB01EY7O8|55|9
GACGAACGCTGGCGGCGTGCCTAACACATGCAAGTCGAACGGGGTTGGAAAGCTTGCTTTTCAACCTAGTGGCGGACGGGTGAGTAATGCGTGAGCAACCTGCCTGTCAGTGGGGGATAACTTCCGGAAACGGACGCTAATACCGCATATTGTACATAATCCGCATGGATAAGTGTATGAAAGGAGTAATCCGCTGACAGATGGGCTCACGTCCGATTAGTAGTTGGTGGGGTAATGGCTCACCAAGACCGCGATCGGTAGCCGGGCTGAGAGGCTGATCGGCCACATTGGGACTGAGACACGGCCCCAGACTCC
>FQH3XDB01B66OP|56|1
GACGAACGCTGGCGGCGTGCCTAACACATGCAAGTCGAACGGAACTTGTTGGAACGAAGCTTCGGCCGAGGGAATTCAAGTTTAGTGGCGGACGGGTGAGTAACGCGTGAGCAACCTGCCTTTCAGAGGGGGATAACGTTTGGAAACGAACGCTAATACCGCATGAGACTACAGAGCCGCATGGCTCAGTGATCAAAGGAGCAATCCGCTGAAAGATGGGCTCGCGTCCGATTAGGTAGTTGGTGAGGTAGAGGCTCACCAAGCCGACGATCGGTAGCCGGACTGAGAGGTTGAACGGCCACATTGGGACTGAGACACGGCCCAGACTCCTACGGGAGCAGCAGTGAGGGATAG
>FQH3XDB01ELR7D|57|3
GACGAACGCTGGCGGCGTGCCTAACACATGCAAGTCGAACGGAGCTAAAGAGCTTGCTTTTAGCTTAGTGGCGGACGGGTGAGTAATGCGTGAGCAACCTGCCTGTCAGTGGGGGATAACTTCCGGAAACGGACGCTAATACCGCATACAGTACATAATCCGCATGGATAAGTGTATGAAAGGAGCAATCCGCTGATAGATGGGCTCACGTCCGATTAGTTAGTTGGTGGGGTAATGGCCTACCAAGACCGCGATCGGTAGCCGGGCTGAGAGGCTGAACGGCCACATTGGGACTGAGACACGGCCCAGACTCCTAC
>FQH3XDB01CBP1D|58|1
GACGAACGCTGGCGGCGTGCCTAACACATGCAAGTCGAACGGAGCTAAAGAGCTTGCTTTTTAGCTTAGTGGCGGACGGGTGAGTAATGCGTGAGCAACCTGCCTGTCAGTGGGGATAACTTCCGGAAAACGGACGCTAATACCGCATACAGTTACATAATCCGCATGGATAAGTGTATGAAAGGAGCAATCCGCTGATAGATGGGCTCACGTCCGATTAGTTAGTTGGTGGGTAATGGCCTACCAAGACCGCGATCGGTAGCCGGGCTGAGAGGCTGAACGGCCACATTGGGACTGAGACGCGGCCCA
>FQH3XDB01AOYR4|59|13
GACGAACGCTGGCGGCGTGCCTAACACATGCAAGTCGAACGGAGCTAAAGAGCTTGCTTTTTAGCTTAGTGGCGGACGGGTGAGTAATGCGTGAGCAACCTGCCTGTCAGTGGGGGATAACTTCCGGAAACGGACGCTAATACCGCATACAGTACATAATCCGCATGGATAAGTGTATGAAAGGAGCAAATCCGCTGATAGATGGGCTCACGTCCGATTAGTTAGTTGGTGGGGTAATGGCCTACCAAGACCGCGATCGGTAGCCGGGCTGAGAGGCTGAAC
>FQH3XDB01DNCIL|60|45
GACGAACGCTGGCGGCGTGCCTAACACATGCAAGTCGAACGAAGCTTTTACGGAATGAGACTTCGGTCAAGTGAAGTTTAAGCTTAGTGGCGGACGGGTGAGTAACGCGTGAGCAACCTGCCTTTCAGAGGGGGGATAACGTTTGGAAACGAACGCTAATACCGCATAACATTGTTTTCCCGCATGAGAGAACAATCAAAGGAGCAATCCGCTGAAAGATGGGCTCGCGTCCGATTAGGTAGTTGGTGGGGTAACGGCCTACCAAGCCGACGATCGGTAGCCGGACTGAGAGGTTGAACGGCCACATTGGGACTGAGACACGGCCC
>FQH3XDB01EYFIS|61|1
GACGAACGCTGGCGGCGTGCCTAACACATGCAAGTCGAACGGAGTTAAGCTCTTCGGAACTTAACTTAGTGGCGAACGGGTGAGTAACGCGTGAGGAACCTGCCTTTCAGTGGGGGACAACAGTTGGAAACGACTGCTAATACCGCATGATGCTTTTGGGAGACATCTCCTGGAAGCCAAAGCTTTATGTGCTGAAAGATGAGCCTGCGGCGTATTAGCTAGTTGGTAAGGTAATGGCTTACCAAGGCAACGATGCGTAGCCGAACTGAGAGGTTGATCGGCCACATTGGGACTGAGACACGGCCCAAAACTCCTAATGGGAGACAGCAGTTAGGAATATTCGTCAAGTGGGGGAAACCCTGAACGAGCAATGCCGCGTGAGTGATGACGGTC
>FQH3XDB01C5RL7|62|1
GACGAACGCTGGCGGCGTGCCTAACACATGCAAGTCGAACGGAGTTAAGCTCTTCGGAACTTAACTTAGTGGCGAACGGGTGAGTAACGCGTGAGGAACCTGCCTTTCAGTGGGGGACAACAGTTGGAAACGACTGCTAATACCGCATGATGCTTTTGGGAGACATCTCCTGGAAGCCAAAAGCTTTATGTGCTGAAAGATGAGCCTGCGGCGTATTAGCTAGTTGGTAAGGTAATGGCTTACCAAGGCAACGATGCGTAGCCGAACTGAGAGGTTGATCGGCCACATTGGGACTGAGACACGGCCCAAAACTCCTATGGGAGACAGCAGTTAGGAATATTCGTCAATGGGGGAAAACCCTGAACGAGCAATGCCGCGTGAGTGATGACGGTCTTGGATTGTAAAACTCTTTTATAGGGACGAACGACTAGTATAGGAAATGATACTAGAGTGACGGTACTTTGAATAAGCCCCGGACTAACTACGTGCCAGCAG
>FQH3XDB01DSVYW|63|1
GACGAACGCTGGCGGCGTGCCTAACACATGCAAGTCGAACGGAGATATGTTTTGGAAGGGCTTCGGCCCGGAAGAGATATATTTTAGTGGCGGACGGGTGAGTAACGCGTGGGCAATCTGCCTTGTAGAAAGGGATAACAGGCCGAAAGGCTTGCTAATACCGGATGAGCTTATCGGGGTCGCATGATCGGATAAGGAAAGGGGAAACCCGCTACAAGATGAGCCTGCGTCCATTAGCTAGTTGGTGAGATAACAGCCCACCAAGGCGGCGATGGGTAGCCGGCCTGAGAGGGTGACCGGCCACATTGGAACTGAG
>FQH3XDB01AJ81J|64|2
GACGAACGCTGGCGGCGTGCCTAACACATGCAAGTCGAACGGGGTATAAGGAGCTTGCTTTTTATACCTAGTGGCGGACGGGTGAGTAAAGCGTGAGCAACCTGCCTATCAGAGGGGGATAACGTTCGGAAACGGATGCTAATACCGCATATAGTACATAATTCGCATGGATAGATGTATGAAAGGAGAGATCCGCTGATAGATGGGCTCACGTCCGATTAGTTAGTTGGTGAGGTAACGGCTCACCAAGGCAGCGATCGGTAGCCGGGCTGAGAGGCTGGACGGCCACATTGGGACTGAGATACGGCCCAGACTCCTACGGGAGCAGCAGGTGGGGATATTGCACAAGTGGGGAAA
>FQH3XDB01APGNL|65|1
GACGAACGCTGGCGGCGTGCCTAACACATGCAAGTCGAGCGAAGCATTTATGAAAGCTTGCTTTTAGAAATGACTTAGCGGCGGACGGGTGAGTAACGCGTGAGCAACCTGGCCTTCACAGGGGGATAACAGTTGGGAACGACTGCTAATACCGCATGACACTTTTAATATCGCATGATATAGAAGTCAAAGATTTATTGGGTGAAAGGGATTGGGGCTCGCGTCCCATTAGCTAGTTGGCAGGGTAACAGCCTACCAAGGCAACGATGGGGTA
>FQH3XDB01B5J43|66|1
GACGAACGCTGGCGGCGTGCCTAACACATGCAAGTCGAGCGGGAAATTCCGAAATGAGGCTTCGGCGGATTTTCGGAATGGATAGCGGCGGACGGGTGAGTAACGCGTGGGTAATCTACCTTCGAGTCTGGAATAACAGTTAGAAATGATTGCTAATGCCGGATGAAATGGGAAACGATACGTCATTTTTATTAAAAGGTGCCTTTAAAGCATCGCTTGAAGATGAGCCTGCGTCGTATTAGTTAGTTGGTGGGGTAACGCCTACCAAGACGACGATGCGTAGCCGACCTGAGAGGGTGATCGGCCACACTGGGACTGAGACACGGCCCAGACTCCTACGGGAGACAGCAGTTAGGAA
>FQH3XDB01A1O19|67|5
GACGAACGCTGGCGGCGTGCCTAACACATGCAAGTCGAACGGAGATCCACGCTGAAAATGAGATTAGTTTACTAAAGGAATTTTCTTGTAGATCTTAGTGGCGGACGGGTGAGTAACGCGTGAGGAACCTGCCCTTTAGAGGGGACAACAGTTGGAAACGACTGCTAATACCGCATAACATACCGAAAGGACATCCTTTTGGTATCAAAGGAGCAATCCGCTGAAGGATGGCCTCGCGTCCGATTAGATAGTTGGTGAGGTAACGGCCCACCAAGTCGACGATCGGTAGCCGGACTGAGAGGTTGAACGGCCACATTGGGACTGAGACACGGCCCAGACTCCTACGGGAGGCAGCAGTGGGGATATTGCACAATGGGGGAAACCCTGATGCAGCAACGCCGCGTGAAGGAAGAAGGTCTTC
>FQH3XDB01BDMPE|68|4
GACGAACGCTGGCGGCGTGCCTAACACATGCAAGTCGAACGGAGATCCACGCTGAAAATGAGATTAGTTTACTAAAGGAATTTTCTTGTAGATCTTAGTGGCGGACGGGTGAGTAACGCGTGAGGAACCTGCCCTTCAGAGGGGGACAACAGTTGGAAACGACTGCTAATACCGCATAACATACCGAAAGGACATCTTTTTGGTATCAAAAGGAGCAATCCGCTGAAGGATGGCCTCGCGTCCGATTAGATAGTTGGTGAGGTAACGGCCCACCAAGTCGACGATCGGTAGCCGGACTGAGAGGTTGAACGGCCACATTGGGACTGAGACACGGCCCAGACTCCTACGGGAGGCAGCAGTGGGGATATTGCACAATGGGGAAACCCTGATGCAGCAACGCCGCGTGAAGGAAGAAGGTCTTCGGATTGTAAACTTCTGTTTTAGTG
>FQH3XDB01C4A8N|69|4
GACGAACGCTGGCGGCGTGCCTAACACATGCAAGTCGAACGGAGTGGAACGCTGAAATTGATGTTAGTTTACTAATGGATTTTTCTTGTTTCACTTAGTGGCGGACGGGTGAGTAACGCGTGAGGAACCTGCCCTTCAGAGGGGGACAACAGTTGGAAACGACTGCTAATACCGCATAACGTATTGAAAGGACATCCTTTTAATACCAAAAGGAGCAATCCGCTGAAGGATGGCCTCGCGTCCGATTAGGTAGTTGGTGAGGTAACGGCTCACCAAGCCGACGATCGGTAGCCGGACTGAGAGGTTGAACGGCCACATTGGGACTGAGACACGGCCCAGACTCCTACGGGAGCAGCAGTGGGGATATTGCACAATGGGGGAAA
>FQH3XDB01AXIM7|70|1
GACGAACGCTGGCGGCGTGCCTAACACATGCAAGTCGAACGGAGTGAAGCGCTGAAAATGATGTTAGTTTACTAATGGATTTTTCTTGTTTCACTTAGTGGCGGACGGGTGAGTAACGCGTGAGGAACCTGCCCTTCAGAGGGGGACAACAGTTGGAAACGACTGCTAATACCGCATAACGTATTGAAAGGACATCCTTTTAATACCAAAGGAGCAATCCGCTGAAGGATGGCCTCGCGTCCGATTAGGTAGTTGGTGAGGTAACGGCTCACCAAGCCGACGATCGGTAGCCGGACTGAGAGGTTGAACGGCCACATTGGGACTGAGACACGGCCCAGACTCCTACGGGAGGCAGCAGTGGGGATATTGCACAATGGGGAAACCCTGATGCAGCAAC
>FQH3XDB01EBN3G|71|2
GACGAACGCTGGCGGCGTGCCTAACACATGCAAGTCGAACGGAGTGGAACGCTGAAATTGATGTTAGTTTACTAATGGATTTTCTTGTTTCACTTAGTGGCGGACGGGTGAGTAACGCGTGAGGAACCTGCCCTTCAGAGGGGGACAACAGTTGGAAACGACTGCTAATACCGCATAACGTATTGAAAGGACATCCTTTTAATACCAAAGGAGCAATCCGCTGAAGGATGGCCTCGCGTCCGATTAGGTAGTTGGTGAGGTAACGGCTCACCAAGCCGACGATCGGTAGCCGGACTGAGAGGTTGAACGGCCACATTGGGACTGAGACACGGCCCCAGACTCCTACGGGAGGCAGCAGTGGGGATATTGCACAATGGGGGAAACCCTGATGCAGCAACGCCGCGTGAAGGAAGAAGGTCTTCGGATTGTAAAACTTCTGTTTTTAAGTGTACGAAACAATGAC
>FQH3XDB01A10A3|72|1
GACGAACGCTGGCGGCGTGCCTAACACATGCAAGTCGAGCGGTTTTTAGAGGCAAGAATCGAGTGGCAAAAGAGGATTTGATTCTGTTATTTGCAACTGGGTTCTTGTTTCTGGAGATAGCGGCGGACGGGTGAGTAACGCGTGAGCAACCTGCCTTCTTGAGGGGGATAACGTCTGGAAACGGACGCTAATACCGCATAAAATACTTAAGTCGCATGGTTTAAGTATCAAAAGGAGCAATCCGCAGGAAGATGGGCTCGCGTCCGATTAGCTAGTTGGAGGGTAAAGGCCCACCAAGGCAACGATCGGTAGCCGGACTGAGAGGTTGAACGGCCACATTGGGACTGAGACACGGCCCAGACTCCTACGGGAGGCAGCAGTGAGGGATATTGCACGAATGGGGGGAACCCTGATG
>FQH3XDB01C8CA3|73|2
GACGAACGCTGGCGGCGTGCTTAACACATGCAAGTCGAACGGGGAAGTAAAGCTTGCTTTGCTTTCTAGTGGCGGACGGGTGAGTAAAGCGTGAGCAACCTGCCTATCAGTGGGGGATAACTTCCGGAAACGGATGCTAATACCGCATATAGTACATAATTCGCATGAATAAGTGTATGAAAGGAGAGATTCGCTGGTAGATGGGCTCACGTCCGATTAGTTAGTTGGTGGGTAAAGGCCTACCAAGACCGCGATCGGTAGCCGGGCTGAGAGGCTGAACGGCCACATTGGGACTGAGACACGGCCCAGACTCCGTACGGGAGGCAGCAGGTGGGGGATATTGCACAAGTGGGGAAACCCTGATGCAGCAACGCCGCGT
>FQH3XDB01A8PLC|74|2
GACGAACGCTGGCGGCGTGCCTGACACATGCAAGTCGAACGGAGTTTCATTCGAAACGGATGGGAGCTTGCTCCTTGAAGTTTTGGATGCAACTTAGTGGCGGACGGGTGAGTAACGCGTGAGCAACCTGCCTTACAGAGGGGGACAACATCGGGAAACTGATGCTAATACCGCATGACATTGTTTTGCCGCATGACAGAACAATCAAAGATTTATCGCTGTAAGATGGGCTCGCGTCCGATTAGGTAGTTGGTGAGGTAACGGCTCACCAAGCCGACGATCGGTAGCCGGACTGAGAGGTTGAACGGCCACATTGGGACTGAGACACGGCCCAGA
>FQH3XDB01DV92T|75|1
GACGAACGCTGGCGGCGTGCTTAACACATGCAAGTCGAACGGACTGAATTTGGTGCTTGCACCGAATGAAAGTTAGTGGCGGACGGGTGAGTAACACGTGAGCAACCTACCCTTCAGAGGGGAATAACGGACGGAAACGTTCGCTAATACCGCATAACATATCTTCGCCGCATGACGGAGGTATCAAAAGATTTTATCGCTGAGGGATGGGCTCGCGGCCGATTAGGGTAGTTGGTGAGGTAACGGCTCACCAAGCCGACGATCGGTAGCCGGACTGAGAGGTTGACCGGCCACATTGGGACTGAGACACGGCCCAGACTCCTACGGGAGGCAGCAGTGGGGAATATTGCGCAATGGGGGAAACCCTGATGCAGCGACGCCGCGTGAGCGAAGAAGGTCTTCGGATCGTAAAGCTCTGTCTTAGGGACGAAAATGACGGTA
>FQH3XDB01AQU0H|76|1
GACGAACGCTGGCGGCGTGCTTAACACATGCAAGTCGAACGGTGATGTCAGGGCTTGCTCTGGCTGATCAGTGGCGAACGGGTGAGTAACACGTGAGTAACCTGCCCCTGTCTCTGGGATAACCATTGGAAACGGTGGCTAATACCGGATATGAGATGTACAGGCATCTGTTGCGTCTGGAAAGATTTATCGGACAGGGATGGACTCGCGGCCTATCAGCTTGTTGGTGAGGTAATGGCTCACCAAGGCGACGACGGGTAGCCGGCCTGAGAGGGCGACCGGCCACACTGGGACTGAGACACGGCCCAGACTCCTACGGGAGGCAGCAGTGGGGAATATTGCACAATGGGCGAAAGCCTGATGCAGCGACGCCGCGTGAGGGAT
>FQH3XDB01DM1S1|77|1
GACGAACGCTGGCGGCGTGCTTAACACATGCAAGTCGAACGAGAATCTGCTGAAAGAGGATTCGTCCAATGGAAGCAGAGGAAAGTGGCGGACGGGTGAGTAACGCGTGGGTAATGTGCCTCTTCGCTGGGGATACCTATTCGAAAGGATAGCTAATACCGAATGAACTTGTTGAACGGCATCGTTTGACTCGTAAACCTCCGGGGCGAAGAGATCAGCTTGCGTACTATCAGCTAGTAGGTGGGGTAACGGCCTACCTAGGCGACGACGGTTAGCCGGTCTGAGAGGATGATCGGCCACATTGGAACTGAAACACGGTCCAGACCTCCTACGGGAGGCAGCAGTGAGGAATCTTGCGCAATGGGCGAAAGCCTGACGCAGCGACACCGC
>FQH3XDB01BIRYZ|78|2
GACGAACGCTGGCGGCGTGCTTAAGACATGCAAGTCGAGCGGGGCATTGAGGAGCTTGCTTCAAGATGCTTAGCGGCGGACGGTGAGTAACGCGTGAGCAACCTGCCCTATACAGCGGGATAACGTTTGGAAACGAACGCTAATACCGCATATGACCACAACCCGCATGGGGAAGAGGTGAAAAGGGTTTACTGGTATAGGATGGGCTCGCGTCTGATTAGCTTGTTGGTGAGGTAACGGCTCACCAAGGCGACGATCAGTAGCCGACCTGAGAGGGTGATCGGCCACATTGGAACTGAGATACGGTCCAGACTC
>FQH3XDB01CKXW5|79|6
GACGAACGCTGGCGGCGTGCTTAAGACATGCAAGTCGAGCGGAGCATTGAGGAGCTTGCTTCAAGATGCTTAGCGGCGGACGGGTGAGTAACGCGTGAGCAACCTGCCCTATACAGCGGGATAACGTTTGGAAACGAACGCTAATACCGCATATGACCACAACCCCGCATGGGGAAGGGGTGAAAGGGTTTACTGGTATAGGATGGGCTCGCGTCTGATTAGCTTGTTGGTGAGGTAACGGCTCACCAAGGCGACGATCAGTAGCCGACCTGAGAGGGTGATCGGCCACATTGGAACTGAGATACGGTCCAGACTCCTACGGGAGCAGCAGTCGGAATATTGGGCAATGGAGGAAACTCTGACCCAGCAACGCCGCGTGAAGGATGAAGGT
>FQH3XDB01CRZ20|80|1
GACGAACGCTGGCGGCGTGCTTAAGACATGCAAGTCGAGCGGAGCATTGAGGAGCTTGCTTCAAGATGCTTAGCGGCGGACGGGTGAGTAACGCGTGAGCAACCTGCCCTATACAGCGGGATAACGTTTGGAAACGAACGCTAATACCGCATATGACCACAACCCCGCATGGGGAAGAGGTTGAAAAGGGGTTTTACTGGGTAATAGGGATGGGCTCGCGTCTGATTAGCTTGTTGGTGAGGTAACGGCTCACCAAGGCGACGATCAGTAGCCGACCTGAGAGGGTGATCGGCCACATTGGAACTGAGATACGGTCCAGACTCCTACGGGGAGGCAGCAGTCGGAATATTGGGCAATGGAGGAAACTCTGACCCAGCAACGCCGCGTGAAGGATGAAGGTCCTCGGATTGTAAACTTCTGTCCTAGGGGAAAATAAGTGATGGTACCCTAGAA
>FQH3XDB01DBOHP|81|1
GACGAACGCTGGCGGCGTGCTTCAAACATGCAAGTCGAACGGAGTTTGAGGGAGCTTGCTCCTGAGAACTTAGTGGCGGACGGGTGAGTAACGCGTGAGCAATCTGGCTATTACAGGGGGATAACAGTTGGAAACGACTGCTAATACCGCATATGACCACAACCTGACATCGGGAAGGGGTGAAAGGAGAAATCCGGTAATAGATGAGCTTGCGTCTCATTAGCTAGTGGTGAGGTAACGCCACCAAGGCGACGATGAGTAGCCGACCTGAGAGGGTGATCGGCCACACTGGTATTG
>FQH3XDB01D3UP8|82|1
GACGAACGCTGGCGGTATGCTTAAGACATGCAAGTCGAGCGGGATTTGTTTGAAGCTTGCTTTGAACAAATCTAGCGGCGGACGGGTGAGTAACGCGTGAGCAACCTGCCTCTTTCATCGGGATACCATTTGGAAACGAATGTTAATACCGAATATGACCACAGCACCACATGGTGCAGGGGTGAAAGGGTTTACTGGAAAGAGATGGGCTCGCGTCTGATTAGCTTGTTGGTGGGTAACGGCCTACCAAGGCGATGATCAGTAGCCGACCTGAGAGGGTGATCGGCCACATTGGAACTGAGAACGGTCCAGACTCCTACGGGAGGCAGCAGTCGGGAATATTGGGGCAAATGGAGGAAACTCTGACCCAGCAATACCGCGTGAAGGATGAAGGTCTTCGGATTGTAAAACTTCTGTTAATAAAAGGAAGAAAACAAATG
>FQH3XDB01DHZVO|83|2
GACGAACGCTGGCGGTATGCTTAAGACATGCAAGTCGAGCGGGGTTTATTGAGAGCTTGCTTTTGATAAACCTAGCGGCGGACGGGTGAGTAACGCGTGAGCAACCTGCCTCTTTCATTGGGATACCAACTGGAAAACGGTTGTTAATACCAAATATGACCACAGCATCGCATGGTGCAGGGGTGAAAGGGTTTACTGGAAAAGAGATGGGCTCGCGTCTGATTAGCTTGTTGGTGAGGTAACGGCCACCAAGGCGACGATCAGTAGCCGACCTGAGAGGGTGATCGGCCACATTGGGGCTGAGAACGGCCCAGACTCCTACGGGAGGCAGCAGTCGGGAATATTGGGCAATGGAGGAAAACTCTGACCCAGCAATACCGCGTGAAGGATGAAGGTCTTCGGATTGTAAAACTTCT
>FQH3XDB01BOKJ9|84|227
GACTAACGCTGGCGGTGCGCCTAACACATGCAAGTCGTACGAGCCCTTCGGGGCGAGTGGCAGACGGGTGAGTAACGCGTAGGTAATGTGCCTCTTCGCTGGGGATACCTATTCGAAAGGATAGCTAATACCGAATGAACTTGTTGAACGGCATCGTTTGACTCGTAAACCTCCGGGGCGAAGAGATCAGCTTGCGTAACTATCAGCTAGTAGGTGGGTAACGGCCTACCTAGGCGACGACGGTTAGCCGGTCTGAGAGGATGATCGGCCACATTGGAACTGAAACACGGTCCAGACTCCTACGGGAGGCAGCAGTGAGGAATCTTGCGCAATGGGCGAAAGCCTGACGCAGCG
>FQH3XDB01EXM82|85|106
AACGAACGCTGGTGGAGTGTCTTATACATGCAAGTCGAGCGAGGACGTAGCGATACGAGCCGAGCGGCGAATGGGTGAGTAACGCGTAAGCAACCTGCCCCGCACACCGGAACAACCGTGCCAACGCGCGGCTAATGCCGGGAGCCGTGGTTCCCCGCATGGGGGATTGACGAAAGATTTATCGGTGCGGGATGGGCTTGCGTCCGATTAGCTAGTTGGCGGGGCAACGGCCACCAAGGCGACGATCGGTAGCCGGCCTGAGAGGGCGATCGGCCACATTGGGACTGAGAGACGGCCCAGACTCCTACGGGAGGCAGCAGTAGGGAATATTGCGCAATGGGGGCAACCCTGACGCAGCAACTCCACGTGTGGGATGAAGCATTTCGGTGT
>FQH3XDB01BQXLV|86|9
GATGAACGCTGGCGGCATGCCTAAGACATGCAAGTCGAACGGAGGGACCCAATGACATTTTATGAAGTTTTGCGAGCTTGCTCAAGAAACGGATTAAAATTGATTTGGATTATCCCTTAGTGGCAAACGGGTGAGTAACACGTGGGTTACCTACCTCCAAGTCGGGGACAACAGTTGGAAACGATTGCTAATACCGGATGTGGACTACGGTTTAAAGAAGCCTTTAAAGCTTCGCTTGGAGATGGGCCTGCGGTGCATTAGCTAGTTGGTGGGGCAATGGCCTACCAAGGCGACGATGCATAGCCGAACTGAGAGGTTAGTCGGCCACATTGGGACTGAGACACGGCCC
>FQH3XDB01CI6B2|87|26
GATAAACGCTGGCGGCATGCCTAATACATGCAAGTCAAACGGGAACATAGCAATATGTTCTAGTGGCGAACGGGTGAGTAACACGTAGGCAACCTGTCCATAAGTCGAGGATAACAGTTGGAAACGACTGATAATACTGGATAGTATAAGAGTTTGCATGAATTCTTATTTAAAGATCCGTTTGGATCTCTTATGGAGGGCCTGCGGTGCATTAGCTAGTTGGTGAGGTAACGGCCCACCAAGGCGACGATGCATAGCTGCGCTGAGAGGCGAAACAGCCACATTGGGACTGAGACACGGCCCAAAACTCCTACGGGAGGCAGCAGTAGGGAATTTTCGGCAATGGGGGAAACCCTGACCGAGCAATGCCGCGTGAATGATGAAGGTCTTCGGATCGTAAAGTTCTGTTGTTAAGGAAGAACGTATGGGATTAGGAAATGAGTTCCATTTGACGGTACTTAACTAGAAAGCCCCGGCTAACTATGTGCCAGCA
>FQH3XDB01D82K0|88|2
GATGAACGCTGGCGGCATGCCTAAGACATGCAAGTCGTACGAAGGGGCCCAATGAAAAGGATTGAAGTTTGAAGTGCTTGCACTGAGAATGGATTGAATTGGATTTGGATTTTCCCCTTAGTGGCAAACGGGTGAGTAACACGTGGGTTACCTGCCTCCAAGATGGGGATAACAGTTGGAAACGACTGATAATACCGAATGTGCTCTACGGAGTAAAGAAGCCCTTAAAGCTTCGCTTGGAGATGGGCCTGCGGCGTATTAGCTAGTTGGTGGGTAATGGCCTACCAAGGCAACGATGCGTAGCCGAACTGAG
>FQH3XDB01APVGR|89|2
GATGAACGCTGGCGGCATGCCTAAGACATGCAAGTCGTACGAAGGGGCCCAATGAAAGAATTGAAATTTGGAGTGCTTGCACAAAAACGGATTTTCTTGGATTTGGATTTTCCCCTTAGTGGCAAACGGGTGAGTAACACGTGGGTTACCTACCTCTAAGATGGGGATAACAATTGGAAACGACTGATAATACCGAATGTGCTCTACGGAGTAAAGAAGCCCTTAAAGCTTCGCTTAGAGATGGGCCTGCGGCGTATTAGCTAGTTGGTGGGGTAATGGCCTACCAAGGCGACGATGCGTAGCCGAACTGAGAGGTTGATCGGCCACACTGGGACTGAGACACGGCCCAGACTCCTAC
>FQH3XDB01CMUKC|90|2
GATGAACGCTGGCGGCATGCCTAAGACATGCAAGTCGTACGAAGGGGCCCAATGAAAAGAATTGAAATTTGGAGTGCTTGCACAAAAAATGGATTTTCTTGGATTTGGATTTTCCCCTTAGTGGCAAACGGGTGAGTAACACGTGGGTTACCTGCCTCCAAGATGGGGATAACAGTTGGAAACGACTGATAATACCGAATATGCTCTACGGAGTAAAGAGCCTTTAAAGCTTCGCTTGGAGATGGGCCTGCGGCGTATTAGCTAGTTGGTGGGTAATGGCCTACCAAGGCAACGATGCGTAGCCGAACTGAGAGGTTGATCGGCCACACTGGGACTGAGACACGGCCCAGC
>FQH3XDB01EEKSQ|91|1
GATGAACGCTGGCGGCATGCCTAAGACATGCAAGTCGTACGAAGGGGCCCAATGAAAAGAATTGAAATTTGGAGTGCTTGCACAAAGAACGGATTTTCTGGACTTGGATTTTCCCCTAGTGGCAAACGGGTGAGTAACACGTGGGTTACCTACCTCCAAGATGGGGATAACAGTTGGAAACGACTGATAATACCGAATGTGCTCTACGGAGTAAAGAAGCCTTTAAAAGCTTCGCTTGGAGATGGGCCTGCGGCGTATTAGCTAGTTGGTGGGTAATGGCCTACAAGGCGACGATGCGTAGCCGAACTGAGAGGTT
>FQH3XDB01CKCY8|92|1
GATGAACGCTGGCGGCATGCCTAAGACATGCAAGTCGTACGAAGGGGCCCAATGAAAGAATTGAAGTTTGAAGTGCTTGCACTGAGAATGGATTGAATTGGATTTGGATTTTCCCCTAGTGGCAAACGGGTGAGTAACACGTGGGTTACCTGCCTCCAAGATGGGGATAACAGTTGGAAACGACTGATAATACCGAATGTGCTCTACGGAGTAAAGAAGCCTTTAAAGCTTCGCTTGGAGATGGGCCTGCGGCGTATTAGCTAGTTGGTGGGGTAATGGCCTACCAAGGCAACGATGCGTAGCCGAACTGAGAGGTTGATCGGCCACACTGGGACTGAGACA
>FQH3XDB01AF2BO|93|19
GATGAACGCTGGCGGCATGCCTAAGACATGCAAGTCGAACGAAGTGGCCCAATGAAGAATGCGAGCTTGCTCAAATTCGGATTTGGTTTACCACTTAGTGGCGCAAGGGTGAGTAACACGTGGGTAATCTACCTTCGAGTCTGGAATAACAGTTAGAAATGATTGCTAATGCCGGATGAAATTGGGAACGAAATTTCATTCTTTATTAAAAGGAGCCTTTAAAGCTTCGCTTGAAGATGAGCCTGCGACGTATTAGCTAGTTGGTGGGGTAAAGGCCTACCAAGGCAACGATGCGTAGCCGACCTGAGAGGGTGATCGGCCACACTGGGACTGAGACACGGCCCCAGACTCCTACGGGGAGACAGCAGTTAGGAATATTCGTCAATGGGGAAACCCTGAACGAGCAATGCCGCGTGAAGGATGACGGTCCTATGGATTGTAAACTTCTGTTGTTAGGGAAGAACGACTAGTGTAGG
>FQH3XDB01CX703|94|2
GATGAACGCTAGCGGCAGGCTTAACACATGCAAGTCGAGGGGCAGCGCGGGTGGCAACACCTGGCGGCGACCGGCGGAAGGGTGCGTAACGCGTGAGCAACCTGCCCGTATCAGGGGGATAACCGGTGGAAACGCCGACTAATACCCCATAACAGTTGAAGCTGCATGGTTTCAACTTGAAAGCTCCGGCGGATACGGATGGGCTCGCGTGACATTAGCTAGTCGGCGGGGTAACGGCCCACCGAGGCGACGATGGTCTAGGGGTTCTGAGAGGAAGGTCCCCCA
>FQH3XDB01EMK7O|95|2
GATTGAACGCTAGCGGCAGGCTTAACACATGCAAGTCGAGGGGCAGCGCGGGTGGCAACACTTGGCGGCGACCGGCGGAAGGGTGCGTAACGCGTGAGCAACCTGCCCGTATCAGGGGGATAACCGGTGGAAACGCCGACTAATACCCCATAACAGTTGAAGCTGCATGGCTTTGACTTGAAAGCTCCGGCGGATACGGATGGGCTCGCGTGACATTAGCTAGTCGGCGGGTAACGCCACCGAGGCGACGATGTCTAGGGGTTCTGAGAGGAAGGTCCCCCA
>FQH3XDB01EMJLB|96|3
GATGAACGCTAGCGGCAGGCTTAACACATGCAAGTCGAGGGGCAGCGCGGGGTGGCAACACTCTGGCGGCGACCGGCGGAAGGGTGCGTAACACGTGAGCAACCTGCCCGTGTCCGGGACATAACCGGCGGAAACGCCGACTAATTTCCCATAACAGCACAGCCCGCATGGGGTGTGCTTGAAAGATGCGTCGGACACGGATGGGCTCGCGGGACATTAGCTGGTTGGAGTGGTAACGGCACACCAAGGCGACGATGTCTAGGGGTTCTGAG
>FQH3XDB01BL4YQ|97|1
GATGAACGCTAGCGGCAGGCTTAACACATGCAAGTCGAGGGGCAGCGCGCGGAGTCAGCAATGACACCGTGGCGGCGACCGGCGCAAGGGTGCGTAACGCGTGAGCAACCTGCCCGTCACGGGGGCATAACCGGTGGAAACGCCGACTAATTCCCCATAGCTCTCCGAGGGGGCATCCCCACGGAGATAAAGGCTTCGGCCGGTGACGGATGGGCTCGCGTGACATTAGGCAGTCGGCGGGGTAACGCCCACCGAACCACGATGTCTAGGGGTTC
>FQH3XDB01EE67D|98|1
GATGAACGCTAGCGGCAGGCTTAACACATGCAAGTCGAGGGGCAGCGCGTTGTAGCAATACGATGGCGGCGACCGGCGAAAGGGTGCGTAACACGTGAGCAACTTGCCCGTATCAGGGGAAAGCGATGGAAACGTCGTCTAATATCCCATAACAATGTTGGCTGCATGGTCGATATTTGAAAGATTCGTCGGATACGGATGGGCTCGCGGGACATTAGCTAGTTGGAGTGGTAACGGCACACCAAGGCGACGATGTCTAGGGGTTCTGAGAGGAA
>FQH3XDB01B8OQF|99|5
GATGAACGCTAGCGGCAGGCTTAACACATGCAAGTCGAGGGGCAGCGCGTTGTGGCAACACGATGGCGGCGACCGGCGGAAGGGTGCGTAACACGTGAGCAACCTGCCCGTGTCCGGGACATAACCGGCGGAAACGCCGACTAATTTCCCATAACATTTCCGGCCGCATGGCCGGGGATTGAAAGATGCGTCGGACACGGATGGGCTCGCGGGACATTAGCTGGTTGGAGTGGTAACGGCACACCAAGGCGACGATGTCGTAGGGGTTCTGAGAGGAA
>FQH3XDB01EPAHF|100|1
GATGAACGCTAGCGGCAGGCTTAACACATGCAAGTCGAGGGGCAGCGCGTGGAGTCAGCAATGACACCATGGCGGCGACCGGCGCAAGGGTGCGTAACGCGTGAGCAACCTGCCCGTCACGGGGGCATAACCGGTGGAAACGCCGACTAATTCCCCATAGCTTTCCCAGAGGGCATCCTCAGGGAAATAAAGGCTTCGGCCGGTGACGGATGGGCTCGCGTGACATTAGGCAGTCGGCGGGTAACGCCCACGAACCTACGATGTCTAGGGGTTCTGAGA
>FQH3XDB01C1029|101|1
GATGAACGCTAGCGACAGGCTTAACACATGCAAGTCGAGGGGCAGCGGGGCTGAAGCTTGCTTCAGCCGCCGGCGACCGGCGCACGGGTGAGTAACACGTATGCAACCTGCCCATGGCAGTGGAATAACCGGAAGAAATTCCGACTAATACCGCATGATACCTCTGGGAGACATCTCCCGGAGGTCAAAGGAGGCGACTCCGGCCATGGATGGGCATGCGCCGCATTAGCTAGGTCGGCGGGTAACGCCACGAGCGACGATGCGGTAGGGGTTC
>FQH3XDB01BQPFA|102|4
GATGAACGCTAGCGGCAGGCTTAACACATGCAAGTCGAGGGGCAGCGGGGCTGAAGCTTGCTTCAGCCGCCGGCGACCGGCGCACGGGTGAGTAACACGTATGCAACCTGCCCATGGCAGTGGGATAACCGGAAGAAATTCCGACTAATACCGCATGACACCTCCGGGAGACATCTTCCGGAGGTCAAAGGAGGCGACTCCGGCCATGGATGGGCATGCGCCGCATTAGCTAGTCGGCGGGTAACGCCCACGAGGCGACGATGCGTAGGG
>FQH3XDB01BS5KA|103|3
GATGAACGCTAGCGACAGGCTTAACACATGCAAGTCGAGGGGCAGCGTGGAGGCTGGCTTGCCAGCCTCTGACGGCGACCGGCGCACGGGTGAGTAACACGTATGCAACCTGCCCCCGGCCGGGGGATAACCCGGAGAAATCCGGACTAATACCGCATAACACCCCTGGGGGACATCCCTCGGGGGTCAAAGGAGAGATCCGGCCGAGGATGGGCATGCGCCGCATTAGGAAGTTGGCGGTGTAACGGACCACCAATCCGACGATGCGTAGGGGTTCTGAGAGGAAGGCCCCCCACACTGGTACTGAGACACGGACCAGACTCCTACGGGAGGCAGCAGTGAGGAATATTGGTCAAGTGGGCGAAGCCTGAACCAGCCAAGTCGCGTGAGGGAAGACGGTCCTATGGATTGTAAACCTCTTTTGCCGGGAGCAACGGCGGCCA
>FQH3XDB01BR2GN|104|4
GATGAACGCTAGCGACAGGCTTAACACATGCAAGTCGAGGGGCAGCGTGGAGGCTGGCTTGCCAGCCTCTGACGGCGACCGGCGCACGGGTGAGTAACACGTATGCAACCTGCCCCGGCCGGGGATAACCCGGAGAAATCCGGACTAATACCGCATAACACCCCTGGGGGACATCCCTCGGGGGTCAAAGGAGAGATCCGGCCGAGGATGGGCATGCGCCGCATTAGGAAGTTGGCGGTGTAACGGACCTCCAATCCGACGATGCGTAGGGTTCTGAGAGGAAGGCCCCCCACACTGGTACTGAGACACGGACCAGACTCCTACGGGAGGCAGCAGTGAGGAATATTGGTCAATGGGCGGAAGCCTGAACCAGCCAAGTCGCGTGAGGGAAGACGGTCCTATGGATTGTAAACCTCTTTGCCGGGAGCAACGGCGCCACGTG
>FQH3XDB01CI90S|105|9
GATGAACGCTAGCGACAGGCTTAACACATGCAAGTCGAGGGGCATCAGGGAAGGAAGCTTGCTTTCTTCCGCTGGCGACCGGCGCACGGGTGAGTAACACGTATGCAACCTGCCCTGTGCAGGGGGATAACCGGAAGAAATTCCGACTAATACCGCATAACACCACTTGGGGACATCCCCTGGTGGTCAAAGGAGGCGACTCCGGCACAGGATGGGCATGCGGCGCATTAGTTAGTTGGCGGGTAACGCCACAAGGCGACGATGCGTAGGGTTC
>FQH3XDB01BHFQH|106|2
GATGAACGCTAGCGACAGGCTTAACACATGCAAGTCGAGGGGCAGCACGGGGTAGCAATACCCCGGTGGCGACCGGCGCACGGGTGAGTAACACGTGTGCAACCAACCCCGTACCGGGAGATAACCCGCGGAAACGTGGACTAATACCCCATAACAGCTTTTCCCCGCATGGGGTAAGGCTTAAAACCCCGGTGGTACGGGACGGGCACGCGCGAACATTAGGTAGTTGGCCGGGGTAACGCCACAAGCCGACGATGTCTAGGGGTTCTGAGAGGAAGGCCCCCCACACTGGAACTGAGACACGG
>FQH3XDB01B8ILX|107|1
GATGAACGCTAGCGACAGGCTTAACACATGCAAGTCGAGGGGCAGCATGAAGTAGCAATACTTTGATGGCGACCGGCGCACGGGTGAGTAACGCGTATGTAACCTGTCCGATACAGGGGAATAGCCCATGGAAACGTGGATTAATGCCCCATATTGCTGCGGATACGCATGTATCTGCAGTGAAATCTACGGAGGTATCGGTTGGGCATGCGTCCTATTAGGTAGTTGGAGAGGTAACGGCTCCCAAGCCGATGATAGGTAGGGGTTCTGAGAGGAAGGTCCCCCACATTGGAACTGAGACACGGTCCAAACTCCTACGGGAGGCAGCAGTGAGGAATATTGGTCAATGGACGGAAGTCTGAACCA
>FQH3XDB01BRYBM|108|1
GATGAACGCTAGCGACAGGCTTAACACATGCAAGTCGAGGGGCAGCGGGGTAGAAAGCTTGCTTTCTACTGCCGGCGACCGGCGCACGGGTGAGTAACACGTATGCAACCTGGCCGTCACAGAGGGATAACCGGGAGAAATCCCGCCTAATACCGCATAATGCGGAGGGAGGCATCCTTCCTCCGCCAAAGGAGTGATCCGGTGACGGATGGGCATGCGCCGCATTAGTTAGTTGGGGGTGTAACGGACCACCAAGACGACGATGCGTAGGGGTTCT
>FQH3XDB01EDNH2|109|1
ATTACCGCGGGCTGCTGGCACGGAGTTAGCCGATGCTTATTCGTCAAGTACTGGCAAGATCGCACTCGTGCAATTTATTCTTCCTTGACAAAAGAGGTTTACAGGCCATAGACCATTCTTCCCTCACGCGACTTGGCTGGTTCAGGCTCTCGCCCATTGACCAATATTCCTCACTGCTGCCTCCCGTAGGAGTCTGGACCGTGTCTCAGTTCCAGTGTGGGGGACCTTCCTCTCAGAACCCCTAGACATCGTCGGCTTGGTGGGCCGTTACCCCGCCAACTACCTAATGTCGCGCGTGCCCGTCCCGTACCACCGGGGTTTTAAGCCTCTCCCCATGCGGAAAAAGCTCTTATGGGGTATTAGTCCACGTTTCCGCGGGTTATCTCCCGGTACGGGTTGGTTGCACACGTGTTACTCACCCGTGCGCCGGTCGCCGGCGGAGTATTGCTACTCCCCGCTGCCCCTCGACTTGCATGTGTTAAGCCTGTCGCTAGCGTTCATC
>FQH3XDB01ATZ1C|110|3
GATGAACGCTAGCGACAGGCTTAACACATGCAAGTCGAGGGGCAGCGGGGAGTAGCAATACTCCGCCGGCGACCGGCGCACGGGTGAGTAACACGTGTGCAACCAACCCCGTACCGGGAGATAACCCGCGGAAACGTGGACTAATACCCCATAATAGCTTTTCCCCGCATGGGGAGAGGCTTAAAACCCCGGTGGTACGGGACGGGCACGCGCGACATTAGGTAGTTGGCGGGTAACGCCACCAAGCCGACGATGTCTAGGGGTTCTGAGAGGAAGGCCCCCCACACTGGAACTGAGACACGGTCCAGACTCCTACGGGAGGCAGCAGTGAGGAATATTGGTCAA
>FQH3XDB01D5X15|111|5
GATGAACGCTAGCGACAGGCTTAACACATGCAAGTCGAGGGGCAGCGGGGAGTAGCGATACTCCGCCGGCGACCGGCGCACGGGTGAGTCACATGTGTGCAACCACCCCTTGCCGGGAGATAACCCGCGGAAACGCGGACTAACATCCCATAACACCGTATGATCGCATGGTCAGTCGGTTAAAATTCCGGTGGCAAGGGACGGGCACGCGCGACATTAGGTAGTTGGCGGGGTAACGGCCCACCAAGCCGACGATGTCCAGGGGTCCCGAGAGGGAGGTCCCCCACACTGGAACTGAGACACGGTCCAGACTCCTACGGGAGGCAGCAGTGAGGAACATTGGGTCAATGGGGCGAGAGCCTGAACCAGCCAAGTCGCGTGAGGGAAGAA
>FQH3XDB01CALBD|112|12
GATGAACGCTGGCGGCATGCCTAAGACATGCAAGTCGAACGAGAAGGCCCAAAGAAGACAGAGTGCTTGCACAAAGTTGGACTTGGATCACCTTCTAGTGGCAAACGGGTGAGTAACACGTGGGTTACCTACCTCTAAGACGGGGATAACAGTTGGAAACGATTGCTAATACCGGATGTGCTCTACGGAGTAAAGGAGCCTTTAAAAGCTTCGCTTAGAGATGGGCCTGCGGTGCATTAGCTAGTTGGCAGGATAACGGCCTACCAAGGCGACGATGCATAGCCGAACTGAGAGGTTAATCGGCCACACTGGGACTGAGACA
>FQH3XDB01B9R2I|113|1
GATGAACGCTAGCGGCGTGCCTAACACATGCAAGTCGAACGAAGCCAATCGACAGAAGCCTTCGGGCGGAAGACGAGGAAGCTTAGTGGCGGACGGGTGAGTAACGCGTGAGCAATCTGCCTTACAGAGGGGGATAACACATAGAAATGTGTGCTAATACCGCATAACATATATTTGCGGCATCGCAGGTATATCAAAGATTTATTGCTGTAAGATGAGCTCGCGTCTGATTAGTTAGTTGGTGGGTAACGCCTACCAAGACGACGATCAGTAGCCGGACTGAGAGGTTGAACGGCCACATTGGGACTGAGACACGGCCCAGACTCC
>FQH3XDB01EJFXO|114|1
GATGAACGCTAGCGGCGTGCCTAACACATGCAAGTCGAACGGAACACCCACGGACTTAACTTCGGTTAAAGAAGTGGAATGTTTAGTGGCGGACGGGTGAGTAACGCGTGAGCAATCTGCCTCACAGAGGGGGATAACACAGAGAAATCTGTGCTAATACCGCATGACATAATCGAATGGCATCATTTGATTATCAAAGATTTATCACTGTGAGATGAGCTCGCGTCTGATTAGTTAGTTGGCGGGTAACGCCACCAAGACGACGATCAGTAGCCGGACTGAGAGGTTGAACGGCCACATTGGGACTGAGACACGGCCCAGACTCCTACGGGAGCAGCAGTGGGGAATATTGCGCGAAGTCGGGGGAACCCTGACGCAGCAACG
>FQH3XDB01ETWDD|115|1
GATGAACGCTAGCGGCGTGCCTAACACATGCAAGTCGAACGGAGCCAATTGACGGAAGCCTTCGGGTGGAAGACAAGGAAGCTTAGTGGCGGACGGGTGAGTAACGCGTGAGCAATCTGCCTTACAGAGGGGGATAACACATAGAAATGTGTGCTAATACCGCATAACATATTAAAGTGGCATCACTATAATATCAAAGATTTATCACTGTAAGATGAGCTCGCGTCTGATTAGTTAGTTGGTGGGTAACGGCCTACCAAGACGACGATCAGTAGCCGGACTGAGAGGTTGAACGGCCACATTGGGACTGAGACACGGCCCAGACTCCTACGGGAGGCAGCAGTGGGGAATATTGCGCAATGGGGGAACCCTGACGCAGCAACGCCGCGTGAAGGAAGAAGGCCTTCGGGTTGTAAACTTTGTCTTAGTGAAAAGTAGGATGGTAGCTAAGGAGGAAGCCACGGCTAACTACGTGCCAGCAGCCG
>FQH3XDB01BBKFI|116|5
GATGAACGCTAGCGGCGTGCCTAACACATGCAAGTCGAACGGAGCCAATTTTCGGAAGTTTTCGGACGGAAGAAGAGGAAGCTTAGTGGCGGACGGGTGAGTAACGCGTGAGCAATCTGCCTTATGGAGGGGGATAACACAGAGAAATTTGTGCTAATACCGCATGAAATATTTAGATGGCATCATTTGAGTATCAAAGATTTATCGCCATAAGATGAGCTTGCGTCTGATTAGTTAGTTGGTGGGTAACGGCCTACCAAGACGACGATCAGTAGCCGGACTGAGAGGTTGAACGGCCACATTGGGACTGAGACACGGCCCAGACTCCTACGGGAGGCAGCAGTGGGGAATATTGCACAATGGGGGGAACCCTGATGCAGCAACGCCGCGTGAAGGAGAA
>FQH3XDB01CA6PO|117|1
GATGAACGCTAGCGGCGTGCCTAACACATGCAAGTCGAACGGAGCCAATTTTCGGAAGCCTTCGGGCAGAAGAAGAGGAAGCTTAGTGGCGGACGGGTGAGTAACGCGTGAGCAATCTGCCTTACAGAGGGGGATAACACATAGAAATGTGTGCTAATACCGCATAACATATTGAGGTGGCATCACTTTGATATCAAAGATTTATCACTGTAAGATGAGCTCGCGTCTGATTAGTTAGTTGGTGGGTAACGGCCTACCAAGACGACGATCAGTAGCCGGACTGAGAGGTTGAACGGCCACATTGGGACTGAGACACGGCCCCAGACTCCTACGGGAGGCAGCAGTGGGAATATTGCGCAAGTGGGGGAACCCTGACGCAGCAACGCCGCGTGAAGGAAGAAGGCCTTCGGGTTAG
>FQH3XDB01ETFGB|118|56
GATGAACGCTGGCGGTGTGCCTAATACATGCAAGTTGAGCGGGGCTCCAGCAATGGAGCCTAGCAGCGGACGGGTGAGTAACACGTAGGTAACCTGCCTTTAAGCCTGGAATACCCGAGAGAAATCTCGGCTAATGCCGGATATGTGATTGAGAGGCATCTCTCAGTCATGAAAGGGGCAATTGCTCCACTTTTAGATGGACCTGCGGCGCATTAGTTAGTTGGTGAGATAACAACCCACCAAGACGAGGATGCGTAGCCGACCTGAGAGGTGATCGGTCACAATGGAACTGAGACACGGTCCATACTCC
>FQH3XDB01ARULD|119|1
GATGAACGCTAGCTACAGGCTTAACACATGCAAGTCGAACGAGACAGCCCATTGATTATTGAGTGCTTGCACAAGATATGATTTGGATTCCTGTCTAGTGGCGCAAGGGTGAGTAACACGTAGGTAATCTACCTTCGAGTCTGGGATAACGGTTAGAAATGATCGCTAATACCGGATTATATATAAGACGATACGTCATCTTATATTAAAAGAGGCCTTTAAAGCCTCGCTTGAAGATGAGCCTGCGGCGTATTAGCTAGTTGGTGAGGTAATGGCTCACCAAGGCGACGATGCGTAGCCGACCTGAGAGGGTGATCGGCCACACTGGGACTGAGACA
>FQH3XDB01CLXJ1|120|5
GATGAACGCTAGCTACAGGCTTAACACATGCAAGTCGAGGGGCAGCATGTCGGTAGCTTGCTACCGATGATGGCGACCGGCGCACGGGTGAGTAACGCGTATCCAACCTGCCCTTGTCCACCGCACAGCCCGTCGAAAGGCGGATTAACGCGGTATGCGGTCCGAAGCGGGCATCTAATTCGGATGAAATGTGAAGGAGAAGGATGGGGATGCGTCTGGATTAGGTAGTCGGTGGGGTAACGGCCCACCGAGCCGACGATCAGTAGGGGTTCTGAGAGGAAGGTCCCCC
>FQH3XDB01CQDMN|121|1
GATGAACGCTAGCTACAGGCTTAACACATGCAAGTCGAGGGGCAGCATGTTCTTAGCTTGCTAAGAATGATGGCGACCGGCGCACGGGTGAGTAACGCGTATCCAACCTGCCCTTGTCCACTGTACAGCCCGTCGAAAGGCGGATTAACACAGTATGCGGTCCTATGCAGGCATCTAAGTAGGACGAAATGTGAAGGAGAAGGATGGGGATGCGTCTGATTAGGTAGTCGGCGGGGTAACGGCCCACCGAGCCGACGATCAGTAGGGGTTCTGAGAGGAA
>FQH3XDB01DUOK7|122|2
GATGAACGCTAGCTACAGGCTTAACACATGCAAGTCGAGGGGCAGCATGTACAGATGCTTGCATCAGTACGATGGCGACCGGCGGCACGGGTGAGTAACGCGTATCCAACCTGCCCATTACTCGGGTATGCCCAGTGAAAACTGGATTAACCCCCGATATATCTTTTGCAGGCATCTAAGAAGATGAAAGTTTACGGTAATGGATGGGGATGCGTCCGATTAGTTAGTTGGTGAGGTAACGGCTCACCAAGACGACGATCGGTAGGGGTTCTGAGAGGAAGGTCCCCCCAGATTGGAACTGAGACACGGTCCAAAC
>FQH3XDB01BJDA6|123|1
GATGAACGCTAGCTACAGGCTTAACACATGCAAGTCGAGGGGGCAGCATGTACAGATGCTTGCATCAGTACGATGGCGACCGGCGCACGGGTGAGTAACGCGTATCCAACCTACCCATTACTCGGGTATAGCCCAGTGAAAACTGGATTAACCCCCGATATATCTTTTGCAGGCATCTAACGAAGATGAAAGTTACGGTACTGGATGGGGATGCGTCCGATTAGCGTTGGTGGCGGGTACGCCACAGGCGACGAGTCGGTAGGGTTC
>FQH3XDB01C9YY9|124|1
GATGAACGCTAGCTACAGGCTTAACACATGCAAGTCGAGGGGAAACGGCATCGAGAGCTTGCTCTTGATGGACGTCGACCGGCGCACGGGTGAGTAACGCGTGAGGAACCTGCCTCAAAGAGGGGGACAACAGTTGGAAACGACTGCTAATACCGCATAAGCCCACGACCCGGCATCGGGTTGAGGGAAAAGGAGCAATCCGCTTTGAGATGGCCTCGCGTCCGATTAGCTAGTTGGTGAGGTAACGGCCCACCAAGGCGACGATCGGTAGCCGGACTGAGAGGTTGAACGGCCACATTGGGACTGAGACACGGCCCCAGACTCCGTACGGGAGGCAGCAGTGGGAATATTGCACAAGTGGGGAAACCCTGATGCAGCGACGCCGCGTGGAGGAGAAGGTCTTCGGATTGTAAACTCCTGTTGTGAGGAAGATAAGTGAC
>FQH3XDB01EB9ZN|125|1
GATGAACGCTGGCAGCATGCCTAAGACATGCAAGTCGAACGAAGTGACCTTTTGAAGTTGGAGTGCTTGCACAAAGATGGATTAAGATTTTCACTTAGTGGCGGACGGGTGAGTAACACGTGGGTGACCTACCTTTAAGTCTGGGATAACTATTAGAAATGATAGCTAATACCGGATTAAATGCAGTTTCGTTCTGCATTAAAAAGGAGCCTTTAAAAGCTTCGCTTATAGATGGGCCTGCGGCGTATTAGCTAGTGGTGGGTAATGGCCTACCAAGGCAACGATGCGTAGCCGAACTGAGAGGTTGATCGGCCACATTGGGACTGAGACACGG
>FQH3XDB01CX5WW|126|47
GATGAACGCTGGCGGCATGCCTAAGACATGCAAGTCGAACGAGGTGGCCTTTTGAAGACAGAGTACTTGTACAAAGTTGGAAAGAGAATTTCCACCTAGTGGCAGACGGGTGAGTAACACGTAAGTAATCTACCGTAAAGACTGGGATAACGTTTGGAAACGAACGCTAATACCGGATAATTCATATTTAGAAAACTAGATATGACTAAAAGGAGCTTCGGCTTCACTTTACGATGAGCTTGCGGCGTATTAACTAGTTGGTGAGGTAATGGCTCACCAAGGTGACGATGCGTAGCCGAACTGAGA
>FQH3XDB01EV0A7|127|6
GATGAACGCTGGCGGCGTGGCCTAATACATGCAAGTCGAACGAATCACCTTCGGGTGATTAGTGGCGAACGGGTGAGTAAAAGATAAGTAACCTGCCCTTGACAGGGGGATAACGACCGGAAACGGACGCTAAGACCGCATAGGTATAATTTACACATGTAGATTATATTAAATATCCTACGGGATAGGTGAAGGATGGACTTATCACGCATTAGCTAGTTGGAGGGTAACGCCCACCAAGGCGACGATGCGTAGCCGGCCTGAGAGGGCGGACGGCCACACTGGGACTGAGACACGGCCCAGACTCCTACGGGAGGCAGCAGTAGGGAATTTTCGGCAATGGGCGAAAGCCTGACCGAGCAACGCCGCGTGAACGAAGAAGGTCTTCGGATTGTAAAGTTCTGTTGTTGAGGAAGAACGGCCTATATAGGGAATGATATAGGAGTGACGG
>FQH3XDB01A7IO5|128|11
GATGAACGCTGGCGGCGTGCCTAATACATGCAAGTCGAACGAATCACCTTCGGGTGATTAGTGGCGAACGGGTGAGTAAAAGATAAGTAACCTGCCCTTGACAGGGGGATAACGACCGGAAACGGACGCTAAGACCGCATAGGTATAATAAACACATGTTTATTATATTAAATATCCTACGGGATAGGTGAAGGATGGACTTATCACGCATTAGCTAGTTGGAGGGTAACGGCCCACCAAGGCGACGATGCGTAGCCGGCCTGAGAGGGCGGACGGCCACACTGGGACTGAGACACGGCCCAGACTCCTACGGGAGGCAGCAGTAGGGAATTTTCGGCAATGGGCGAAAGCCTGACCGAGCAACGCCGCGTGAACGAAGAAGGTCTTCGGATTGTAAAGTTCTGTTGTTGAGGAAGAACGGCCTATATAGGGAATGATATAGGAGTGACGGTACTCAATTAGAAAGCCACGGCTAACGTACGTGCCAGCAGCCG
>FQH3XDB01CUZBS|129|2
GATGAACGCTGGCGGCGTGCCTAATACATGCAAGTCGAACGAATCACCTTCGGGTGATTAGTGGCGAACGGGTGAGTAAAAGATAAGTAACCTGCCCTTGACAGGGGGATAACGACCGGAAACGGACGCTAAGACCGCATAGGTATAATAAACACATGTTTATTATATTAAATATCCTACGGGGATAGGTGAAGGATGGACTTATCACGCATTAGCTAGTTGGAGGGTAACGCCACCAAGGCGACGATGCGTAGCCGGCCTGAGAGGGCGGACGGCCACACTGGGACTGAGACACGGCCCAGACTCCTACGGGAGGCAGCAGTAGGATTTTCGGCAATGGGCGAAAGCCTGACCGAGCAACGCCGCGTGAAC
>FQH3XDB01BUHF8|130|12
GATGAACGCTGGCGGCGTGCCTAATACATGCAAGTCGAACGGATTGTAGAAATACAGTCAGTGGCGAACGGGTGAGTAACACATAAATAATCTACCCTTAAGACCGGGATAACGTTTGGAAACGAGCGCTAATACCGGATAGGCTTATCGGAGGCATCTTAGATAAGTTAAAGCAGCTGCAAGGCTGTACTGAAGGATGAGCTTATGACGCATTAGTTAGTGGCAGAGTAAAAGCCTACCAAGACGATGATGCGTAGCCGATCTGAGAGGATGACCGGCCACATTGGGACTGAGACACGGCCCAGACTCCTACGGGAGCAGCAGTAGGGAATTTTCGGCAATGGGCGAAAGCCTGACCGAGCAACGCCGCGTGAACGAAGAAGGTCTTTGGATCGCAGAGTTCTGTTGCGGGGAAGAATGAGCAGTATAGGAAATGATACTG
>FQH3XDB01AL42I|131|1
GATGAACGCTGGCGGCGTGCCTAATACATGCAAGTCGAACGGATTGTAGAAATACAGTCAGTGGCGAACGGGTGAGTAACACATAAATAATCTACCCTTAAGACCGGGAGTAACGTTTTGGTAAACGAAGCGCTAATACCGGATAGGCTTATCGGAGGGCATCTTAGATAAGTTAAAGACAGCTGCAAGGCTGTACTGAAGGATGAGTTTATGACGCATTAGTTAGTTGGCAGAGTAAAAGACCTACCAAGACGATGATGCGTAGCCGATCTGAGAGGATGACCGGCCACATTGGGACTGAGACACGGCCCAGACTCCTACGGGAGGCAGCAGTAGGGAATTTTCGGCAATGGGCGAAAGCCTGACCGAGCAACGCCGCGTGAACGAAGAAGGTCTTTGGATCGCAGAGTTCTGTTGGCGGGGAAGAATGAGCAGT
>FQH3XDB01APAZ5|132|1
GATGAACGCTGGCGGCATGCCTAACACATGCAAGTCGAACGAAGCATTGTCAACGGAAGCTTCGGCCGAAGTTGACTTTGACTTAGTGGCGGACGGGTGAGTAACGCGTGAGTAATCTGCCTTAGAGAGGGGGATAACGTTCCGAAAGGGACGCTAATACCGCATAATATATTTGGGAGGCATCTTCTGGATATCAAAGATTTATCACTTTAAGATGAGCTCGCGTATGATTAGCTAGTTGGTGAGGTAACGGCTCACCAAGGCGACGATCATTAGCCGGACTGAGAGGTTGAACGGCCACATTGGGACTGAGACACGGCCCAGACTCCTACGGGAGGCAGCAGTGGGAATATTGCGCAAGTGGGGGAAACCCTGACGCAGCAATGCCGCGTGAAGGATGAAGGTTTTCGGATTGTAAACTT
>FQH3XDB01D21NT|133|3
GATGAACGCTGGCGGCATGCCTAAGACATGCAAGTCGAACGAAGAAGCCCAATGAAGATTGAGTGCTTGCACGAGATTGGAACTGGATACTTCTTAGTGGCAGACGGGTGAGTAACACGTGGGTAATCTACCTCAGAGACTGGGATAACGTTTGGAAACGAACGCTAATACCGGATAAATCATATTTGGATAACCAGATATGCTAAAAGGAGCTACGGCTTCACTTTGAGATGAGCTTGCGGTGTATTAGCTAGTTGGTGAGGTAATGGCTCACCAAGGCAACGATGCATATCCGAGCTGAGAGGCTGATCGGACACACTGGGGACTGAGACACGGCCAGACTCCTACGGAGACAGCAGTTAGGAATATTCGTCAATGGAGGAAACTCTGAACGAGCAATGCCGCGTGAGTGATGAAGGTCTTACAGATTGTAAAA
>FQH3XDB01AUO3U|134|2
GATGAACGCTGGCGGCATGCCTAAGACATGCAAGTCGAACGAAACAGCCTGTAGAAATTCAAGTGCTTGCACGAGAATGGAAATAGATACTGTTTAGTGGCGGACGGGTGAGTAACACGTGGGTGATCTACCTATAAGACTGGGATAACAATTAGAAATGATTGCTAATACCGGATGATATACATTTTCATTAATGTATTAAAAGTTGCCTTCAAGCAATACTTATAGATGAGCCTGCGGCATATTAGCTAGTTGGTAAGGTAAGAGCTTACCAAGGCGACGATGCGTAGCCGGACCGAGAGGTTGAACGGTCACACTGGAACTGAGACACGGTCCAGACTCCTACGGGAGACAGCAGTTAGGAATATTCGTCAAGTGGGGGAAACCCTGAACGAGCAATGCCGCGTGAAGGATGAAGGTCTTTAGATTGTAAACTTC
>FQH3XDB01EU3JD|135|1
GATGAACGCTGGCGGCATGCCTAAGACATGCAAGTCGAACGGAACAGCCCATAGAAGATTGAGTGCTTGCACAAGATTGGAAATGGTTTACTGTTTAGTGGCAGACGGGTGAGTAACACGTAGGTAACCTGCCCTAGAGACTGGGACAACAGTTAGAAATGACTGCTAATACCGGATAAATCATATTTAGAAAACTAGATATGCTAAAAGGAGCGTATGCTTCACTTTAGGATGGGCTTGCGGTGTATTAGCTAGTTGGTGGGGTAATGGCTCACCAAGGCAACGATGCATATCCGAGCTGAGAGGCTGATCGGACACACTGGGACTGAGACACGGCCCAGACTCCTACGGGAGACAGCAGTTAGGAATATTCGTCAAGTGGGGGAACCCTGAACGAGCAATGCCGCGTGAGTGATGAAGG
>FQH3XDB01EDXMF|136|1
GATGAACGCTGGCGGCATGCCTAAGACATGCAAGTCGAACGGAATGGCCCATTGAAGACGGAGTGCTTGCACGAAGTTGGATATGGATTTCCATTCAGTGGCAGACGGGTGAGTAACACGTGGGTAATCTACCTCAGAGACTGGGATAACGTTTGGAAACGAACGCTAATACCGGATAATTCATATTTAGAAAACTAGATATGCTAAAAGGAGCTACGGCTTCACTTTGAGATGAGCTTGCGGTGTATTAGCTAGTTGGTGAGGTAATGGCTCACCAAGGCAACGATGCATATCCGAGCTGAGAGGCTGATCGGACACACTGGGGACTGAGACACGGCCCAGACTCCTACGGGAGACAGCAGTTAGGAATATTCGTCAATGGAGGAAACTCTGAACGAGCAATGCC
>FQH3XDB01DGDVD|137|3
GATGAACGCTGGCGGCATGCCTAAGACATGCAAGTCGAACGGGACGGCCCAATGAAGATTGCGTGCTTGCACAAAATCAGATTTGGATTCCCGTCTAGTGGCGCAAGGGTGAGTAGCACGTGGGTAATCTGCCTTCGAGTCTGGAATAACAGTTAGAAATGATTGCTAATGCCGGATTATATATAGGAGATACACTTCTATATTAAAAGGAGCCTTTAAAGCTTCGCTTGAAGATGAGCCTGCGCCGTATTAGCTAGTTGGTGGGGTAATGGCCTACCAAGGCAACGATGCGTAGCCGACCTGAGAGGGCGATCGGCCACACTGGGACTGAGACACGGCCCAGACCTCCGTACGGGAGACAGCAGTTAGGAATATTCGTCAAGTGGGGAAACCCTGAACGA
>FQH3XDB01D3259|138|6
GATGAACGCTGGCGGCATGCCTAAGACATGCAAGTCGAACGAGAAGGCCTGTAGAAGACAGAGTGCTTGCACAAAGTTGGAAACAGATCACCTTCTAGTGGCAAACGGGTGAGTAACACGTGGGTTACCTACCTCTAAGACGGGGACAACAGTTGGAAACGATTGCTAATACCGGATGTGATCTACGGATTAAAGAAGCCTTTAAAAGCTTCGCTTAGAGATGGGCCTGCGGTGCATTAGCTAGTTGGTGGGTAACGCCTACCAAGGCGACGATGCATAGCCGAACTGAGAGGTTAATCGGCCACACTGGGACTGAGACACGGCCCAGACTCCTA
>FQH3XDB01CJTZC|139|1
GATGAACGCTGGCGGCATGCCTAAGACATGCAAGTCGAACGAGAAGGCCTATGGAAAACGGAGTGCTTGCACGAAGTTGGAAATAGATTCCCTTCTAGTGGCAAACGGGTGAGTAACACGTGGGTTACCTACCTCTAAGACGGGGACAACAGTTGGAAACGATTGCTAATACCGGATGTGCTCTACGGAGTAAAGAAGCCCTTAAAGCTTCGCTTAGAGATGGGCCTGCGGTGCATTAGCTAGTTGGTGGGTAATGGCCTACCAAGGCGACGATGCATAGCCGAACTGAGAGGTTAATCGGCCACACTGGGACTGAGACACGGCCCAGA
>FQH3XDB01BPAM8|140|2
GATGAACGCTGGCGGCATGCCTAAGACATGCAAGTCGAACGAGAAGGCCCAAAGAAGACAGAGTGCTTGCACAAAGTTGGACTTGGATCACCTTCTAGTGGCAAAACGGGTGAGTAACACGTGGGTTACCTACCTCTAAGACGGGGATAACAGTTGGAAACGATTGCTAATACCGGATGTGCTCTACGGAGTAAAGGAGCCTTTAAAAGCTTCGCTTAGAGATGGGCCTGCGGTGCATTAGCTAGTTGGCAGGATAACGGCTACAGGCACGATGCATAGCCGAACTGAGAG
>FQH3XDB01D9Y6C|141|3
GATGAACGCTGGCGGCATGCCTAAGACATGCAAGTCGAACGAGAAGGCCCAAAGAAGACAGAGTGCTTGCACAAAGTTGGACTTGGATCACCTTCTAGTGGCAAACGGGTGAGTAACACGTGGGTTACCTACCTCTAAGACGGGGATAACAGTTGGAAACGATTGCTAATACCGGATGTGCTCTACGGAGTAAAGGAGCCTTTTAAAAGCTTCGCTTAGAGATGGGCCTGCGGTGCATTAGCTAGTTGGCAGGATAACGGCCTACCAAGGCGACGATGCATAGCCGAACTGAGAGGTTAATCGGCCACACTGGGACTGAGACACGG
>FQH3XDB01A2B8P|142|1
GATGAACGCTGGCGGCATGCCTAAGACATGCAAGTCGAACGAGAAGGCCCAAAGAAGACAGAGTGCTTGCACAAAGTTGGACTTGGATCACCTTTCTAGTGGCAAAACGGGTGAGTAACACGTGGGTTACCTACCTCTAAGGACGGGGATAACAGTTGGAAACGATTGCTAATACCGGATGTGCTCTACGGAGTAAAGGAGCCTTTAAAGCTTCGCTTAGAGATGGGCCTGCGGTGCATTAGCTAGTTGGCAGGATAACGGCCTACCAAGGCACGATGGCA
>FQH3XDB01A390Q|143|15
GATGAACGCTGGCGGCATGCCTAAGACATGCAAGTCGAACGAGAAGGCCCAAAGAAGGCAGAGTGCTTGCACAAAGTTGGACTTGGATTACCTTCTAGTGGCAAACGGGTGAGTAACACGTGGGTTACCTACCTCTAAGACGGGGATAACAGTTGGAAACGATTGCTAATACCGGATGTACTCTACGGAGTAAAGAAGCCTTTAAAGCTTCGCTTAGAGATGGGCCTGCGGTGCATTAGCTAGTTGGCAGGATAACGGCCTACCAAGGCGACGATGCATAGCCGAACT
>FQH3XDB01B8POX|144|1
GATGAACGCTGGCGGCATGCCTAAGACATGCAAGTCGAACGAGAAGGCCCAAAGAAGACAGAGTGCTTGCACAAAGTTGGACTTGGATTACCTTCTAGTGGCAAACGGGTGAGTAACACGTGGGTTACCTACCTCTAAGACGGGGATAACAGTTGGAAACGATTGCTAATACCGGATGTGCTCTACGGAGTAAAGAAGCCTTTAAAGCTTCGCTTAGAGATGGGCCTGCGGTGCATTAGCTAGTTGGTGGGTAACGCCTACAAGGCGACGATGCATAGCCGAA
>FQH3XDB01B8DC1|145|1
GATGAACGCTGGCGGCATGCCTAAGACATGCAAGTCGAACGAGAAGGCCCAAAGAAGACAGAGTGCTTGCACAAAGTTGGACTTGGATTACCTTCTAGTGGCAAACGGGGTGAGTAACACGTGGGTCACCTACCTCTAAGACGGGGATAACAGTTGGAAACGATTGCTAATACCGGATGTGCTCTACGGAGTAAAGGAGCGAATTATCGCTTCGCTTAGAGATGGGCCTGCGGTGCATTAGCTAGTTGGTAGGTAACGCCTACCAAGGCGACGATGCATAGCCGAACTGAGAGG
>FQH3XDB01AOVX9|146|1
GATGAACGCTGGCGGCATGCCTAAGACATGCAAGTCGAACGAGAAGGCCCAATGATTATGGAGTGCTTGCACAAAGTATGATTTGGATTTCCTTCTAGTGGCAAACGGGTGAGTAACACGTGGGTTACCTACCTCTAAGACGGGGACAACAGTTGGAAACGATTGCTAATACCGGATGTGCTCTACGGAGTAAAGGAGCCTTAAAAGCTTCGCTTAGAGATGGGCCTGCGGTGCATTAGCTAGTTGGTGGGGTAATGGCCTACCAAGGCGACGATGCATAGCCGAACTGAGAGGTTAATCGGCCACACTGGGACTGAGACACGGCCCAGACTCCTACGGGAGACAGCAGTTAGGAATATTCGTCAATGGGGAAACCCTGAACGAGCAATGCCGCGTGAGTGATGACGGTCCTATGGATTGTAAAACTCTGTTGTTTGGAAAGAACTGTATAAGTAGGAAATGACTTATAC
>FQH3XDB01C6MT0|147|3
GATGAACGCTGGCGGCATGCCTAAGACATGCAAGTCGAACGAGAAGGCCCAATGAAAACGGAGTGCTTGCACAAAGTTGGACTTGGATCACCTTCTAGTGGCAAACGGGTGAGTAACACGTGGGTTACCTACCTCTAAGACTGGGATAACAGTTGGAAACGATTGCTAAATACCGGATGTGCTCTACGGAGTAAAGAAGCCTTTAAAGCTTCACTTAGAGATGGGCCTGCGGTGCATTAGCTAGTTGGTGGGGTAATGGCCTACCAAGGCGACGATGCATAGCCGAACTGAGAGGTTAATCGGCCACACTGGGACTGAGACACGGCCCAGACTCCGTACGGAGACAGCAGTTAGGAATATTCGTCAAGTGGGGAAACCCTGAACG
>FQH3XDB01BPH9P|148|2
GATGAACGCTGGCGGCATGCCTAAGACATGCAAGTCGAACGAGAAGGCCCATTGAAGACTGAGTGCTTGCACAAAGTTGGATATGGATTCCCTTCTAGTGGCAAACGGGTGAGTAACACGTGGGTTACCTGCCTCTAAGACGGGGATAACAGTTGGAAACGACTGCTAATACCGGATGTGATCTACGGATTAAAGGAGCCTTAAAGCTCCGCTTAGAGATGGGCCTGCGGTGCATTAGCTAGTTGGTGGGTAAGGCCTACCAAGGCGACGATGCATAGCCGAACTGAGAGGTTAA
>FQH3XDB01CMPNS|149|1
GATGAACGCTGGCGGCATGCCTAAGACATGCAAGTCGAACGAGAAGGCCCATTGATTACGGAGTGCTTGCACAAAGTATGAAATGGATTTCCTTCTAGTGGCAAACGGGTGAGTAACACGTGGGCTACCTACCTCTAAGACGGGGACAACAGTTGGAAACGATTGCTAATACCGGATGTGCTCTACGGAGTAAAGGAGCCTCAAAGCTTCGCTTAGAGATGGGCCTGCGGTGCATTAGCTAGTTGGTGGGTAATGGCCTACCAAGGCGACGATGCATAGCCGAACTGGAGAGGTTAATCGGCCACACTGGGACTGAG
>FQH3XDB01CJXX9|150|5
GATGAACGCTGGCGGCATGCCTAAGACATGCAAGTCGAACGAGACAGCCCATTGATTATTGAGTGCTCGCACAAGATATGATTTGGATTCCTGTCTAGTGGCGCAAGGGTGAGTAACACGTAGGTAATCTACCTTCGAGTCTGGGATAACGGTTAGAAATGATCGCTAATACCGGATTATATATAAGACGATACGTCATCTTATATTAAAAGAGGCCTTTAAAGCCTCGCTTGAAGATGAGCCTGCGGCGTATTAGCTAGTTGGTGAGGTAATGGCTCACCAAGGCGACGATGCGTAGCCGACCTGAGAGGGTGATCGGCCACACTGGGGACTGGGGACACGGCCCAGACTCCGTACGGGAGACAGCAGTTAGGAATATCGTCAAGTGGGGGAAACCCTGAACGAGCAA
>FQH3XDB01BRV0G|151|6
GATGAACGCTGGCGGCATGCCTAAGACATGCAAGTCGAACGAGACAGCCCATTGATTATTGAGTGCTTGCACAAGATATGATTTGGATTCCTGTCTAGTGGCGCAAGGGTGAGTAACACGTAGGTAATCTACCTTTGAGTCTGGGATAACGGTTAGAAATGATCGCTAATACCGGATTATATATGAGATGATACGTCATCTTATATTAAAAGAGGCCTTTAAAGCCTCGCTTGAAGATGAGCCTGCGGCGTATTAGCTTAGTTGGTGAGGTAATGGCTCACCAAGGCGACGATGCGTAGCCGACCTGAGAGGTGATCGGCCACGAC
>FQH3XDB01E066L|152|4
GATGAACGCTGGCGGCATGCCTAAGACATGCAAGTCGAACGAGAAGGCCCAAAGAAAACAGAGTGCTTGCACAAAGTTGGACTTGGATCACCTTCTAGTGGCAAACGGGTGAGTAACACGTGGGTTACCTACCTCTAAGACTGGGATAACAGTTGGAAACGATTGCTAATACCGGATGTGCTCTACGGAGTAAAGGAGCCTTTAAAGCTTCACTTAGAGATGGGCCTGCGGTGCATTAGCTAGTTGGTGGGGTAATGGCCTACCAAGGCGACGATGCATAGCCGAACTGAGAGGTTAATCGGCCACACTGGGACTGAGACACGGCCCAGACTCCGTACGGGAGACAGCAGCTAGGAATATTCGTCAAGTGGGGAAACCCTGAACGAGCAATGCCGCGTGA
>FQH3XDB01C78ST|153|1
GATGAACGCTGGCGGCATGCCTAAGACATGCAAGTCGAACGAGAAGGCCCATAGAAGATAGAGTGCTTGCACAAAGTTGGAAAATGGATTCCCTTCTAGTGGCAAACGGGTGAGTAACACGTGGGTTACCTACCTCTAAGACGGGGATAACAGTTGGAAACGATTGCTAATACCGGATGTGCTCTACGGAGTAAAGGAGCCTTTAAAAGCTTCGCTTAGAGATGGGCCTGCGGTGCATTAGCTAGGTTGGGTGGGGTTAATGGCCTACCGAAGGCGACGATGCATAGCCGAA
>FQH3XDB01DDMWF|154|11
GATGAACGCTGGCGGCATGCCTAAGACATGCAAGTCGAACGAGAAGGCCCATAGAAGATAGAGTGCTTGCACAAAGTTGGAAATGGATTCCCTTCTAGTGGCAAACGGGTGAGTAACACGTGGGTTACCTACCTCTAAGACGGGGATAACAGTTGGAAACGATTGCTAATACCGGATGTGCTCTACGGAGTAAAGGAGCCTTTAAAGCTTCGCTTAGAGATGGGCCTGCGGTGCATTAGCTAGTTGGTGGGTAATGGCCTACCAAGGCGACGATGCATAGCCGAACTGAGAGGTTAATCGGCCACACGT
>FQH3XDB01CGWZ7|155|1
GATGAACGCTGGCGGCATGCCTAAGACATGCAAGTCGAACGAGATGGCCCAATGAAGAATGCGTGCTTGCACAAATTTGGATTTGGATCACCATCTAGTGGCGCAAGGGTGAGTAATACGTGGGTAATCTGCCTTCGAGTTTGGAATAACAATTAGAAATGATTGCTAATGCCGAATTATATATTTAAAGATACGTCTTTGAATATTAAAAGGAGCCTTTAAAGCTTCGCTTGAAGATGAGCCCACGCCGTATTAGCTAGTTGGTGGGGTAATGGCCTACCAAGGCGACGATGCGTAGCCGACCTGAGAGGGTGATCGGCCACACTGGGACTGAGACACGGCCCAGA
>FQH3XDB01CD0IP|156|5
GATGAACGCTGGCGGCATGCCTAAGACATGCAAGTCGAACGAAACAGACTAATGAAATTCAAGTGCTTGCACGAGAATGGAAATAGAACCTGTTTAGTGGCGGACGGGTGAGTAACACGTGGGTGATCTGCCTTTAAGACTGGGATAACAATTAGAAATGATTGCTAATACCGGATGATATGTATATTCGGTTATACATTAAAAGTTGCTTTCGGGCAATACTTAAAGATGAGCCTGCGGCGTATTAGCTAGTTGGTAAGGTAATGGCTTACCAAGGCGACGATGCGTAGCCGGACCGAGAGGTTGAACGGTCACACTGGAACTGAGACACGGTCCAGACTCCTACGGGAGACAGCAGTTAGGAATATTCGTCAATGGGGAAACCCTGAACGAGCAATGCCGCGTGAAGGATGAAGGTCTTATAGATTGTAAACTTCTGTT
>FQH3XDB01AM1UN|157|1
GATGAACGCTGGCGGCATGCCTAAGACATGCAAGTCGAACGAAGCGGCCCGTTGAAACAGAGTGCCTGCACAAAGTAGATTCGGATTTCCGCTTAGTGGCGCAAGGGTGAGTAACACGTGGGTAATCTACCTCAGAGACAGGGATAACGGTTGGAAACGATCGCTAATACCTGATTAAATTTAAGAAGATACGTTTCTAAATTAAAGGGAGCCTTTAAAAGCTTCGCTTTGAGATGAGCTTGCGGCGTATTAGCTAGTTGGTGAGGTAATGGCTCACCAAGGCAACGATGCGTAGCCGACCTGAGAGGGTGATCGGCCACATTGGGACTGAGACACGGCCCAAAACTCCTACGGGAGACAGCAGTTAGGAATATTCGTCAATGGGGGAAACCCTGAACGAGCAATGCCGCGTGAGTGATGACGGTCCTTTGGATTGTAAAAACTCTGTTGTTAGGGAAGAACGACCTATGTAGG
>FQH3XDB01DCKK5|158|24
GATGAACGCTGGCGGCATGCCTAAGACATGCAAGTCGAACGAAGCGGCCCGTTGAAACAGAGTGCTTGCACAAAGTAGATTCGGATTTCCGCTTAGTGGCGCAAGGGTGAGTAACACGTGGGTAATCTACCTCAGAGGCAGGGATAACGGTTGGAAACGATCGCTAATACCTGATTAAATTTAAGAAGATACGTTTTCTTAAATTAAAAGGGAGCCTTTAAAGCTTCGCTTTGAGATGAGCTTGCGGCGTATTAGCTAGTTGGTGAGGTAATGGCTCACCAAGGCAACGATGCGTAGCCGACCTGAGAGGGTGATCGGCCACATTGGGACTGAGACACGGCCCAAAACTCCTACGGGAGACAGCAGTTAGGAATATTCGTCAAGTGGGGAAACCCTGAACGAGCAATGCCGCGTGAGTGATGAC
>FQH3XDB01BDQ0I|159|1
GATGAACGCTGGCGGCATGCCTAAGACATGCAAGTCGAACGAAGCGGCCCGTTGAAACAGAGTGCTTGCACAAAGTAGATTCGGATTTCCGCTTAGTGGCGCAAGGGTGAGTAACACGTGGGTAATCTACCTCAGAGACAGGGATAACGGTTGGAAACGATCGCTAATACCTGATTAAATTTAAGAAGATACGTTTTCTTAAATTAAAAGGGAGCCTTTAAAAGCTTCGCTTTGAGATGAGCTTGCGGCGTATTAGCTAGTTGGTGAGGTAATGGCTCACCAAGGCAACGATGCGTAGCCGACCTGAGGGGTGATCGGCCACATTGGGGACTGAGACACGGCCCAAAACTCCTAACGGGGAGACCAGCAGTTAGGAATATTCGTCAAGTGGGGGAACCCTGAACGAGCAATGCCGCGTGAGTGATGAC
>FQH3XDB01A7NVT|160|5
GATGAACGCTGGCGGCATGCCTAAGACATGCAAGTCGAACGAAGCGGCCCGTTGAAATGGAGTGCTTGCACAAAGTAGATTCGGATTTCCGCTTAGTGGCGCAAGGGTGAGTAACACGTGGGTAATCTACCTCAGAGACTGGGATAACGGTTGGAAACGATCGCTAATACCGGATTATATGTAAGAAGATACGTTTCTTAAATTAAAGGAAGCCTTTAAAGCTTCGCTTTGAGATGAGCTTGCGGCGTATTAGCTAGTTGGTAAGGTAATGGCTTACCAAGGCAACGATGCGTAGCCGACCTGAGAGGGTGATCGGCCACATTGGGACTGAGACACGGCCCAAAACTCCTACGGGAGACAGCAGTTAGGAATATTCGTCAAGTGGGGAAACCCTGAACGAGCAATGCCGCGTGAGTGATGACGGTCCTTGGATTGTAAAACTCTGTTGTTAGGAAGACGACCTGTGTAGGAAATGACAC
>FQH3XDB01C1OEO|161|3
GATGAACGCTGGCGGCATGCCTAAGACATGCAAGTCGAACGAAGCGGCCCGTTGAAATGGAGTGCTTGCACAAAGTAGATTCGGATTTCCGCTTAGTGGCGCAAGGGTGAGTAACACGTGGGTAATCTACCTCAGAGACTGGGATAACGGTTGGAAACGATCGCTAATACCGGATTATATGTAAGAAGATACGTTTTCTAAATTAAAAGGAAGCCTTTAAAAGCTTCGCTTTGAGATGAGCTTGCGGCGTATTAGCTAGTTGGTAAGGTAATGGCTTACCAAGGCAACGATGCGTAGCCGACCTGAGAGGTGATCGGCCACATTGGGACTGAGACACGGCCCAAAATCC
>FQH3XDB01AVF5C|162|1
GATGAACGCTGGCGGCATGCCTAAGACATGCAAGTCGAACGAAGTAGCCTAATGAAGATGGAGTGCTTGCACAAAATTGGAATTAGTTTACTACTTAGTGGCAGACGGGTGAGTAACACGTGGGTAACCTACCTCAGAGACTGGGATAACGTTTGGAAACGAACGCTAATACCGGATGAATTATAGAAAGATAACTTTTATACTAAAAGGAGCTTTCGGCTTCACTTTGAGATGGGCTTGCGGTGTATTAGCTAGTTGGTGGGTAATGGCCTACAAGGCAACGATGCATATCCGAGCTGAGAGGCTGATCGGACA
>FQH3XDB01D2X7C|163|2
GATGAACGCTGGCGGCATGCCTAAGACATGCAAGTCGAACGAAGTAGTCCAATGAGAGCGGAGTGCTTGCACAAAGCAGGATTTGGGTAGCTACTTAGTGGCGCAAGGGTGAGTAACACGTAGGTAACCTACCTTAGAGACTGGGATAACAGTTGGAAACGACTGCTAATACCGGATGATATGTATAAAGATACGTCTTTGTACATTAAAAGGAGCCTTTAAAAGCTTCACTTTAAGATGGGCCTGCGGCGTATTAGCTAGTTGGTGGGTAAGAGCCTACCAAGGCGACGATGCGTAGCCGACCTGAGAGGGTGATCGGCCACACTGGGGACTGAGACACGGCCCAGACTCCTACGGGAGACAGCAGTTAGGAATATTCGTCAAGTGGGGGAACCCTGAACGAGCAATGCCGCGTGAAGGAGAAG
>FQH3XDB01EBBC4|164|1
GATGAACGCTGGCGGCATGCCTAAGACATGCAAGTCGAACGAAGTAGTCCACTGATAACAGTGTGCTTGCACAATGTTGGATGTGGTTCGCTACTTAGTGGCAGACGGGTGAGTAACACGTGGGTAACCTACCCTTAAGACTGGGATAACTACTGGAAACGGTAGCTAATACCGGATAATTCCTATTTGGATAACCAGATAGGCTAAAAGAAGCTTTCGCTTCACTTTTGGATGGGCTTGCGTTGTATTAGCTAGTTGGTGAGGTAATGGCCCACCAAGGCAACGATGCATATCCGAGCTGAGAGGCTGATCGGACACATTGGGGACTGAGACACGGCCCAGACTCCTACGGGAGACAGCAGTTAGGAATATTCGTCAATGGGGGAAACCCTGAACGAGCAATGCCGCGTGAGCGATGAAGGTCTTATAGATCGTAAAGCTCTGTTGTAAAGGAAGAACA
>FQH3XDB01AVNJJ|165|5
GATGAACGCTGGCGGCATGCCTAAGACATGCAAGTCGAACGAAGTGAACCAATGAAAATGGAGTGCTTGCACAAAGTTGGATTTGGATTCTTCACTGAGTGGCAAACGGGTGAGTAACACGTGGGTTACCTACCTCTAAGACGGGGACAACAGTTGGAAACGATTGCTAATACCGGATGTGATCTATGGATTAAAGAAGCCTTTAAAGCTTCGCTTAGAGATGGGCCTGCGGTGCATTAGCTAGTTGGTAGGTAATGGCCTACAAGGCGACGATGCATAGCCGAA
>FQH3XDB01DREDO|166|2
GATGAACGCTGGCGGCATGCCTAAGACATGCAAGTCGAACGAAGTGAACCAATGAAAACGGAGTGCTTGCACAAAGTTAGATTTGGATTCTTCACTGAGTGGCAAACGGGTGAGTAACACGTGGGTTACCTACCTCTAAGACGGGGACAACAGTTGGAAACGATTGCTAATACCGGATGTGATCTATGGATTAAAGAAGCCTCTAAAAGCTTCGCTTAAAAGATGGGCCTGCGGTGCATTAGCTAGTTGGTGGGGTAATGGCCTACCAAGGCGACGATGCATAGCCGAACTGAGAGGTTAATCGGCCACACTGGGACTGAGACACGGCCCAGACTCCTACGGGAGACAGCAGTTAGAATATTCGTCAA
>FQH3XDB01CJ46C|167|2
GATGAACGCTGGCGGCATGCCTAAGACATGCAAGTCGAACGAAGTGAACCAATGAAAATGGAGTGCTTGCACAAAGTTGGATTTGGATTCTTCACTGAGTGGCAAACGGGTGAGTAACACGTGGGTTACCTACCTCTAAGACGGGGACAACAGTTGGAAACGATTGCTAATACCGGATGTGATCTATGGATTAAAGAAGCCTTTAAAGCTTCGCTTAGAGATGGGCCTGCGGTGCATTAGCTAGTTGGTAGGGTAATGGCCTACCAAGGCGACGATGCATAGCCGAACTGAGAGGTTAATCGGCCACACTGGGACTGAGACACGGCCCAGACTCC
>FQH3XDB01D11C9|168|2
GATGAACGTTGGCGGCATGCCTAAGACATGCAAGTCGAACGAAGTGGCCCATTGAAGATTGAGTGCTTGCACAAAATTGGATTTGGATTCCCACTTAGTGGCAGACGGGTGAGTAACACGTGGGTAATCTACCTCAGAGATTGGGATAACGTTTGGAAACGAACGCTAATACCGAATGATTTATATTTAGATAACTAGATATGCTAAAAGGAGCTTCGGCTCCGCTTTGAGATGAGCTTGCGGTGTATTAGCTAGTTGGTGGGTAATGGCTTACCAAGGCAACGATGCATAGCCGAGCTGAGAGGCTGATCGGCCACATTGGAACTGAGATACGGTCCAAAACTCCTACGGGAGGC
>FQH3XDB01CN511|169|1
GATGAACGCTGGCGGCATGCCTAAGACATGCAAGTCGAACGAAGTGGCCCATTGAAGATTGAGTGCTTGCACAATTTCGGATTTGGATTCCCACTTAGTGGCAGACGGGTGAGTAACACGTGGGTAACCTACCCTGAAGACTGGGATAACTACTGGAAACGGTAGCTAATACCGGATAATTCATAATTAGATAACTAGTTATGCTAAAAGGAGCGTTTGCTTCACTTCAGGATGGGCTTGCGTTGTATTAGCTAGTTGGTGGGGTAATGGCCTACCGAAGGCAACGATGCATATCCGAGCTGAGA
>FQH3XDB01CW786|170|1
GATGAACGCTGGCGGCATGCCTAAGACATGCAAGTCGAACGAAGTGGCCCATTGAAGATTGCGTGCTTGCACAAAATCGGATTTGGATTCCCACTTAGTGGCAGACGGGTGAGTAACACATGGGGTAACCTACCCCAGGGACTGGGATAACTATTGGAAACGATAGCTAATACCGGATGATTCATTGGTAGATAACTACTAATGCTAAAAGGAGCTTCGGCTTCACCTTGGGATGGGCTTGTGGCGTATTAGCTTGTTGGTGGGGTAATGGCCTACCAAGGCAATGATGCGTATCCGAGCTGAGAGGCTGATCGGACACATTGGAACTGAGACACGGTCCAAAACTCCTACGGGAGCAGCAGTTAGGAATATTCGTCAAGTGGGGAAACCCTGAACGAGCAATGCCGCGTGAGTGATGAAGGTC
>FQH3XDB01CGY34|171|4
GATGAACGCTGGCGGCATGCCTAAGACATGCAAGTCGAACGAAGTGGCCCATTGATGATGGAGTGCTTGCACAAAATCTGATTTGGATTTTCCACTTAGTGGCAGACGGGTGAGTAACACGTGGGTAACCTACCTCGTAGACCGGGATAACAGTTAGAAATGATTGCTAATACCGGATTATTCATATTTAGATAACTAGATATGCTAAAAGGGAGCTTTCGGCTTCGCTTCGAGATGGGCTTGCGGTGCATTAGCTAGTTGGTGAGGTAACGGCTCACCAAGGCGACGATGCATATCCGAGCTGAGAGGCTGATCGGACAC
>FQH3XDB01DB8HO|172|2
GATGAACGCTGGCGGCATGCCTAAGACATGCAAGTCGAACGAAGTGGCCTTTAGAAAGCTGAGTGCTTGCACAAAGCTGGAAAAAGATTTCCACTTAGTGGCAGACGGGTGAGTAACACGTGGGTAATCTACCCTAGGGACTGGGATAACGTTTGGAAACGAACGCTAATACCGGATGATACATATTTAGATAACTAGATATGTTAAAAGGAGCTTCGGCTTCACCTTAGGATGAGCTTGCGGTGTATTAGCTAGTTGGTGGGGTAATGGCCTACCAAGGCAACGATGCATAGCCGAGCTGAGAGGCTGATCGGCCACATTGGAACTGAGATA
>FQH3XDB01EB2B0|173|3
GATGAACGCTGGCGGCATGCCTAAGACATGCAAGTCGAACGAAGTGGTCCAAGGAAGATGGAGTGCTTGCACGAAATCTGAATTGGGTTACCACTTAGTGGCGAAAGGGTGAGTAACACGTAGGTTATCTACCTTTGAGACTGGAATAACAATTAGAAATGATTGCTAATACCGGATGATATATATAGTGATACGTCAATATATATTAAAAGGAGCCTTTTAAAAGCTTCACTTAAAGATGAGCCTGCGACGTATTAGCTAGTTGGTGGGTAAAGGCCTACCAAGGCGACGATGCGTAGCCGACCTGAGAGGGTGATCGGCCACACTGGGACTGAGACACGGCCCAGACTCCTACGGGAGACAGCAGTTAGGAATATTCGTCAATGGG
>FQH3XDB01C3WBK|174|1
GATGAACGCTGGCGGCATGCCTAAGACATGCAAGTCGAACGAAGTGGCCCAACAATAATGGAGTGCTTGCACAAAATTTGCGATGGAATTTCCACTTAGTGGCAGACGGGTGAGTAACACGTAAGTAATCTACCTTAAAGATTGGGATAATGTTTGGAAACGAACACTAATACCGGATAATTCATATTTAGATAACTAGATATGCTAAAAGGAGCGTTTGCTTCGCTTTAAGATGAGCTTGCGGCGTATTAACTAGTTGGTGGGGTAATGGCCTACCAAGGTGACGATGCGTAGCCGAACTGAGAGGTTGATCGGCCACATTGGGACTGAGACACGGCCCCAAAATCCTACGGGAGCAGCAGTTAGGATATTCGTCAATGGGGAAACCCTGAACGAGCAATGCCGCGTGAGTGATGACGGTCTTATGATTGTAAAACTCTGTTGTAAGGAACGAACCTTAGGATAGGAAATG
>FQH3XDB01AFYL6|175|5
GATGAACGCTGGCGGCGTGCCTAAGACATGCAAGTCGAACGAAGTGGCCCAACAATAACTGAGTGCTTGCACAAAGTTTGCGATGGAATTTCCACTTAGTGGCAGACGGGTGAGTAACACGTAAGTAATCTACCTTAGAGACTGGAATAATGTTTGGAAACGAGCACTAATACCGGATAATTCATATTTAGATAACTAGATATGCTAAAAGAAGCGTTTGCTTCACTTTAAGATGGGCTTGCGGCGTATTAACTAGTTGGTGAGGTAATGGCTCACCAAGGTGACGATGCGTAGCCGAACTGAGAGGTTGATCGGCCACATTGGGACTGAGACACGGCCCAAAA
>FQH3XDB01B9LB3|176|1
GATGAACGCTGGCGGCATGCCTAAGACATGCAAGTCGAACGAAGTGGCCCAACAATAACTGAGTGCTTGCACAAAGTTTGTCGATGGAATTTCCACTTAGTGGCAGACGGGTGAGTAACACGTAAGTAATCTACCTTAGAGACTGGAATAATGTTTGGAAACGAGCACTAATACCGGATAATTCATATTTAGATAACTAGATATGCTAAAAGAAGCGTTTGCTTCACTTTAAGATGGGCTTGCGGCGTATTAACTAGTTGGTGAGGTAATGGCTCACCAAGGTGACGATGCGTAGCCGAATGAGAGGTTGATCGGCCAGATTGGGACTGAGACACGGCCC
>FQH3XDB01DH7DL|177|3
GATGAACGCTGGCGGCATGCCTAAGACATGCAAGTCGAACGAAGTGGCCCAACAATAATTGAGTGCTTGCACGAAATTTGCGATGGAATTTCCACTTAGTGGCAGACGGGTGAGTAACACGTAAGTAACCTACCCTAAAGACTGGAATAATGTTTGGAAACGAACACTAATACCGGATAATTCATATTTAGATAACTAGATATGCTAAAAGGAGCGTTTGCTTCACTTTGGGATGGGCTTGCGGCGTATTAACTAGTTGGTGGGGTAATGGCCTACAAGGTGACGATGCGTAGCCGAACT
>FQH3XDB01BZ9G4|178|1
GATGAACGCTGGCGGCATGCCTAAGACATGCAAGTCGAACGAAGTGGCCCAACAATAGCTGCGTGCTTGCACAATGCTTGCGATGGAATTTCCACTTAGTGGCAGACGGGTGAGTAACACGTAAGTAACCTGCCTCGAAGACTGGAATAATGTCTGGAAACGGACACTAATACCGGATAATTCATATTTAGATAACTAGATATGCTAAAAGGAGCGTTTGCTCCACTTCGAGATGGGCTTGCGGCGTATTAACTAGTTGGTGGGGTAATGGCCTACCAAGGTGACGATGCGTAGCCGAACTGAGAGGTTGATCGGCCACATTGGGACTGAGACACGGCCCAAAATCCTACGGGAGGCAGCAGTTAGGAATATTCGTCAATGGGGAAACCCTGAACGAGCAATGCCGCGTGAGTGATGACGTCTTA
>FQH3XDB01CMXDL|179|1
GATGAACGCTGGCGGCATGCCTAAGACATGCAAGTCGAACGAAGTGGCCCAACAATAATGGAGTGCTTGCACAAAATTTGCGATGGAATTTTCCACTTAGTGGCAGACGGGTGAGTAACACGTAAGTAATCTACCTTAAAGATTGGGATAAGTGTTTGGAAACGAACACTAATACCGGATAATTCATATTTAGATAACTAGATATGCTGAAAGGAGCGTTTTGCTTTCGCTTTAAGATGAGCTTGCGGCGTATTAACTAGTTGGTGGGGTAATGGCCTACCAAGGTGACGATGCGTAGCCGAA
>FQH3XDB01AQIO5|180|3
GATGAACGCTGGCGGCATGCCTAAGACATGCAAGTCGAACGAAGTGGCCCAACAATGACTGAGTGCTTGCACAAAGTTTGCGATGGAATTTCCACTTAGTGGCAGACGGGTGAGTAACACGTAAGTAATCTACCTCGAAGACTGGAATAATGTTTGGAAACGAACACTAATACTGGATGATTCATATTTAGATAACTAGATATGCTAAAAGGAGCGTTTGCTCCACTTCGAGATGAGCTTGCGGCGTATTAACTAGTTGGTGGGGTAATGGCCTACCAAGGTGACGATGCGTAGCCGAACTGAGAGGTTGATCGGCCACATTGGGACTGAGACACGGCCCAAAATCCTACGGGAGGCAGCAGTTAGGAATATTCGTCAAGTGGGGGAAACCCTGAACGAGCAATGCCGCGTGAGTGATGACGGTCTTATGATTGTAAAACTCTGTTGTAAGG
>FQH3XDB01BILFZ|181|1
GATGAACGCTGGCGGCATGCCTAAGACATGCAAGTCGAACGAAGTGGCCCAATGAAGAATGCGAGCTTGCTCAAATTTAGATTTGGATAACCACTTAGTGGCGCAAGGGTGAGTAACGCGTAGGTAATCTACCTTCGAGTCTGGAATAACAGTTAGAAATGATTGCTAATACCGGATGAAATGGGAAACGATACGTCATTTTTATAAAAGGTGCCTTTAAAGCATCGCTTGAAGATGAGCCTGCGTCGTATTAGTTAGTTGGTGGGGTAATGGCCTACCAAGACAACGATGCGTAGCCGACCTGAGAGGGT
>FQH3XDB01EUFHA|182|3
GATGAACGCTGGCGGCATGCCTAAGACATGCAAGTCGAACGAAGTGGCCCAATGAAGAATGCGAGCTTGCTCAAATTTAGATTTGGATAACCACTTAGTGGCGCAAGGGTGAGTAACGCGTAGGTAATCTACCTTCGAGTCTGGAATAACAGTTAGAAATGATTGCTAATACCGGATGAAATGGGAAACGATACGTCTATTTTTATTAAAAGGTGCCTTTAAAGCATCGCTTGAAGATGAGCCTGCGTCGTATTAGTTAGTTGGTGGGGTAATGGCCTACCAAGACAACGATGCGTAGCCGACCTGAGAGGGTGATCGGCCAC
>FQH3XDB01B5EZH|183|3
GATGAACGCTGGCGGCATGCCTAAGACATGCAAGTCGAACGAAGTGGCCCAATGAAGAATGCGAGCTTGCTCAAATTTAGATTTGGATAACCACTTAGTGGCGCAAGGGTGAGTAACGCGTAGGTAATCTACCTTCGAGTCTGGAATAACAGTTAGAAATGATTGCTAATGCCGGATGAAATGGGAAACGATACGTCATTTTTATTAAAAGGTGCCTTTAAAGCATCGCTTGAAGATGAGCCTGCGTCGTATTAGTTAGTTGGTGGGGTAATGGCCTACCAAGACAACGATGCGTAGCCGACCTGAGAGGTGATCGGCCACACTGGGGACTGAGACACGGCCCAGACTCCTACGGGGAGACAGCAGTTAGGAATATTCGTC
>FQH3XDB01DD0R1|184|15
GATGAACGCTGGCGGCATGCCTAAGACATGCAAGTCGAACGAAGTGGCCCAATGAAGAATGCGAGCTTGCTCAAATTTAGATTTGGATTACCACTTAGTGGCGCAAGGGTGAGTAACGCGTGGGTAATCTACCTTCGAGTCTGGAATAACAGTTAGAAATGATTGCTAATGCCGGATGAAATGGGAAACGATACGTCATTTTTATTAAAAGGTGCCTTTAAAGCATCGCTTGAAGATGAGCCTGCGTCGTATTAGTTAGTTGGTGGGTAACGGCCTACCAAGACGACGATGCGTAGCCGACCTGAGAGGGTGATCGGCCACACTGGGACTGAGACACGGCCCAGACTCCTACGGGAGACCAGCAGTTAGGAATATTCGTCAAGTGGGGAAAACCCTGAACGAGCAATGCCGCGTGAGCGAAGAAGTATTCGGTATGTAAAGCTCTAT
>FQH3XDB01AEO6G|185|14
GATGAACGCTGGCGGCATGCCTAAGACATGCAAGTCGAACGAAGTGGCCCAATGAAGAATGCGAGCTTGCTCAAATTTAGATTTGGATTACCACTTAGTGGCGCAAGGGTGAGTAACGCGTGGGTAATCTACCTTCGAGTCTGGAATAACAGTTAGAAATGATTGCTAATGCCGGATGAAATGGGAAACGATACGTCATTTTTATAAAAGGTGCCTTTAAAGCATCGCTTGAAGATGAGCCTGCGTCGTATTAGTTAGTTGGTGGGTAACGCCTACCAAGACGACGATGCGTAGCCGA
>FQH3XDB01BL001|186|10
GATGAACGCTGGCGGCATGCCTAAGACATGCAAGTCGAACGAAGTGGCCCAATGAAGAATGCGAGCTTGCTCAAATTTAGATTTGGATTACCACTTAGTGGCGCAAGGGTGAGTAACGCGTGGGTAATCTACCTTCGAGTCTGGAATAACAGTTAGAAATGATTGCTAATGCCGGATGAAATGGGAAACGATACGTCATTTTTATAAAAGGTGCCTTTAAAGCATCGCTTGAAGATGAGCCTGCGTCGTATTAGTTAGTTGGTGGGGTAACGGCCTACCAAGCGACGATGCGTAGCCGACCTGAGAGGGTGATC
>FQH3XDB01C39SU|187|6
GATGAACGCTGGCGGCATGCCTAAGACATGCAAGTCGAACGAAGTGGCCCAATGAAGAATGCGAGCTTGCTCAAATTTAGATTTGGATTACCACTTAGTGGCGCAAGGGTGAGTAACGCGTGGGTAATCTACCTTCGAGTCTGGAATAACAGTTAGAAATGATTGCTAATGCCGGATGAAATGGGAAACGATACGTCATTTTTATTAAAAGGTGCCTTTAAAGCATCGCTTGAAGATGAGCCTGCGTCGTATTAGTTAGTTGGTGGGGTAACGGCCTACCAAGACGACGATGCGTAGCCGACCTGAGAGGGTGATCGGCCACACTGGGACTGAGACACGGCCCAGACTCCTA
>FQH3XDB01C9PBL|188|4
GATGAACGCTGGCGGCATGCCTAAGACATGCAAGTCGAACGAAGTGGCCCAATGAAGAATGCGAGCTTGCTCAAATTTAGATTTGGATTACCACTTAGTGGCGCAAGGGTGAGTAACGCGTGGGTAATCTACCTTCGAGTCTGGAATAACAGTTAGAAATGATTGCTAATGCCGGATGAAATGGGAAACGATACGTCATTTTTATTAAAAGGTGCCTTTAAAGCATCGCTTGAAGATGAGCCTGCGTCGTATTAGTTAGTTGGTGGGTAACGCCTACCAAGACGACGATGCGTAGCCGACCTGAGAGGGTGATCGGCCACACTGGGGACTGAGACACGGCCCAG
>FQH3XDB01B5HAP|189|4
GATGAACGCTGGCGGCATGCCTAAGACATGCAAGTCGAACGAAGTGGCCCAATGAAGAATGTGAGCTTGCTCAAATTTAGATTTGGATTACCACTTAGTGGCGCAAGGGTGAGTAACGCGTGGGTAATCTACCTTCGAGTCTGGAATAACAGTTAGAAATGATTGCTAATGCCGGATGAAATGGGAAACGATACGTCTATTTTTATTAAAAGGTGCCTTTAAAGCATCGCTTGAAGATGAGCCTGCGTCGTATTAGTTAGTTGGTGGGTAACGCCTACCAAGACGACGATGCGTAGCCGACCTGAGAGGGTGATCCGGCCACACTGGGACTGAGACACGG
>FQH3XDB01DZK34|190|13
GATGAACGCTGGCGGCATGCCTAAGACATGCAAGTCGAACGAAGTGGCCCAATGAAGAATGTGAGCTTGCTCAAATTTAGATTTGGATTACCACTTAGTGGCGCAAGGGTGAGTAACGCGTGGGTAATCTACCTTCGAGTCTGGAATAACAGTTAGAAATGATTGCTAATGCCGGATGAAATGGGAAACGATACGTCATTTTTATAAAAGGTGCCTTTAAAGCATCGCTTGAAGATGAGCCTGCGTCGTATTAGTTAGTTGGTGGGTAACGCCTACCAAGACGACGATGCGTAGCCGACTGAGA
>FQH3XDB01DLJT7|191|9
GATGAACGCTGGCGGCATGCCTAAGACATGCAAGTCGAACGAAGTGGCCCAATGAAGAATGTGAGCTTGCTCAAATTTAGATTTGGATTACCACTTAGTGGCGCAAGGGTGAGTAACGCGTGGGTAATCTACCTTCGAGTCTGGAATAACAGTTAGAAATGATTGCTAATGCCGGATGAAATGGGAAACGATACGTCATTTTTATAAAAGGTGCCTTTAAAGCATCGCTTGAAGATGAGCCTGCGTCGTATTAGTTAGTTGGTGGGGTAACGGCCTACCAAGACGACGATGCGTAGCCGACCTGAGAGGGTGATCGGCCACACTGGGACTGAGACACGGCCCAGACTCCTACGGGAGACAGCAGTTAGGAATATTCGTCAAGTGGGGGAAACCCTGAACGAGCAATGCCGCGTGAAGGATC
>FQH3XDB01AIKJM|192|4
GATGAACGCTGGCGGCATGCCTAAGACATGCAAGTCGAACGAAGTGGCCCAATGAAGAATGTGAGCTTGCTCAAATTTAGATTTGGATTACCACTTAGTGGCGCAAGGGTGAGTAACGCGTGGGTAATCTACCTTCGAGTCTGGGATAACAGTTAGAAATGATTGCTAATGCCGGATGAAATGGGAAACGATACGTCATTTTTATTAAAAGGTGCCTTTAAAGCATCGCTTGAAGATGAGCCTGCGTCGTATTAGTTAGTTGGTGGGTAACGGCCTACCAAGACGACGATGCGTAGCCGACCTGAGAGGGTGA
>FQH3XDB01C8U76|193|1
GATGAACGCTGGCGGCATGCCTAAGACATGCAAGTCGAACGAAATGGCCTAATGAAGATGGAGTGCTTGCACAAAGTTGGAATTAGATTTCCATTTAGTGGCAGACGGGTGAGTAACACGTGGGTAACCTACCTCAAAGACTGGGATAACGTTTAGAAATGAACGCTAATACCGGATAAATTATAGAAAGATAACTTTTATACTAAAAGGGGCTTCGGCCTTACTATGAGATGGGCTTGCGGTGTATTAGCTAGTTGGTGGGGTAATGGCCTACCAAGGCAACGATGCATATCCGAGCTGAGAGGCTGATCGGACACATTGGGACTGAGACACGGCCCAGACTCCTACGGGAGACAGCAGTTAGGAATATTCGTCAATGGGGAAACCCTGAACGAGCAATGCCGCGTGAGTGATGAAGGTC
>FQH3XDB01DWI0X|194|1
GATGAACGCTGGCGGCATGCCTAAGACATGCAAGTCGAACGAAGGGACCCAATGAAGACGGAGTGCTTGCACAAAGTTGGATTTGGATTTTCCCTTAGTGGCAAACGGGTGAGTAACGCGTGGGTTACCTACCTCTAAGTTGGGGATAACAGTTGGAAACGATTGCTAATACCGAATGTGTTCTACGGAATAAAGAAGCCCTTAAAGCTTCGCTTAGAGATGGGCCTGCGTTGTATTAGCTAGTTGGTGGGTAACGGCCTACCAAGGCAACGATGCATAGCCGAGCTGAGAGGTTAATCGGCCACACTGGGACTGAGACACGGCCCAGACTCCTACGGGAGACAGCAGTTAGGAATATTCGTCAATGGGGGAAACCCTGAACGAGCAATGCCGCGTGAGTGATGAAGGCCCTCTGGGTTGTAAAACTC
>FQH3XDB01CRL1N|195|1
GATGAACGCTGGCGGCATGCCTAAGACATGCAAGTCGAACGAAGGGACCCAATGAAAGTTGTGTGCTTGCACAAAACTGGATTTGGATCATCCCTTAGTGGCAAACGGGTGAGTAACACGTGGGTAACCTACCTTTAAGTTGGGGATAACAGTTGGAAACGATTGCTAATACCGAATGTGATCTACGGATTAAGAAGCCTTTAAAGCTTCGCTTGGAGATGGGCCTGCGGCGTATTAGCTTGTTGTGGGGTAAATGGCCTTACCAAGGCAACGATACGTAGCCGAACTGAGAGGTTAATCGGCCA
>FQH3XDB01BHT8T|196|1
GATGAACGCTGGCGGCATGCCTAAGACATGCAAGTCGAACGAAGCGGCCCGATGACATGGAGTGCTTGCACAAAATAGATTCGGATCTCCGCTTAGTGGCGCAAGGGTGAGTAACACGTGGGTAATCTACCTCAGAGACTGGGATAACAGTTGGAAACGATTGCTAATACCAGATGATATGTAAGAAGATACGTTTTCTTAAATTAAAAGGAAGCCTTTAAAGCTTCGCTTTGAGATGAGCCTGCGGCGTATTAGCTAGTTGGTAGGGTAATGGCCTACCAAGGCAACGATGCGTAGCCGACCTGAGAGGTGATCGGCCACATTGGGACTGAGACAC
>FQH3XDB01ET3LU|197|44
GATGAACGCTGGCGGCATGCCTAAGACATGCAAGTCGAACGAAGCGGCCCGATGACATGGAGTGCTTGCACAAAATAGATTCGGATTTCCGCTTAGTGGCGCAAGGGTGAGTAACACGTGGGTAATCTACCTCAGAGACTGGGATAACAGTTGGAAACGATTGCTAATACCAGATGATATGTAAGAAGATACGTTTTCTTAAATTAAAAGGAGCCTTTAAAGCTTCGCTTTGAGATGAGCCTGCGGCGTATTAGCTAGTTGGTAGGGTAATGGCCTACCAAGGCAACGATGCGTAGCCGACCTGAGAGGGTGATCGGCCACATTGGGACTGAGACACGGCCCAAACTCCTACGGGAGACAGCAGTTAGGGATATTCGTCAAGTGGGGAAACCCTGAACGAGCAATGCCGCGTGAGTGA
>FQH3XDB01DAMIF|198|9
GATGAACGCTGGCGGCATGCCTAAGACATGCAAGTCGAACGAAGCGGCCCGATGACATGGAGTGCTTGCACAAAATAGATTCGGATTTCCGCTTAGTGGCGCAAGGGTGAGTAACACGTGGGTAATCTACCTCAGAGACTGGGATAACAGTTGGAAACGATTGCTAATACCAGATGATATGTAAGAAGATACGTTTTCTTAAATTAAAAGGAAGCCTTTAAAGCTTCGCTTTGAGATGAGCCTGCGGCGTATTAGCTAGTTGGTAGGTAATGGCCTACCAAGGCAACGATGCGTAGCCGACCTGAGAGGGTGATCGGCCACATTGGGACTGAGACACGGCCCAAACTCCGTAACGGGAGACAGCAGTTAGGGATATTCGTCAAGTGGGGAAACCCTGAACGA
>FQH3XDB01DNBN4|199|14
GATGAACGCTGGCGGCATGCCTAAGACATGCAAGTCGAACGAAGCGGCCCGATGACATGGAGTGCTTGCACAAAATAGATTCGGATTTCCGCTTAGTGGCGCAAGGGTGAGTAACACGTGGGTAATCTACCTCAGAGACTGGGATAACAGTTGGAAACGATTGCTAATACCAGATGATATGTAAGAAGATACGTTTTCTTAAATTAAAGGAAGCCTTTAAAAGCTTCGCTTTGAGATGAGCCTGCGGCGTATTAGCTAGTTGGTAGGGTAATGGCCTACCAAGGCAACGATGCGTAGCCGACCTGAGAGGGTGATCGGCCACATTGGGACTGAGACACGGCCCAAAACTCCTACGGGAGACAGCGGTTAGGATATTCGTCAATGGGGAAACCCTGAACGAGCAATGCCGCGTGAGTGATGACGG
>FQH3XDB01EQHOC|200|35
GATGAACGCTGGCGGCATGCCTAAGACATGCAAGTCGAACGAAGCGGCCCGATGACATGGAGTGCTTGCACAAAGTAGATTCGGATTTCCGCTTAGTGGCGCAAGGGTGAGTAACACGTGGGTAATCTACCTCAGAGACTGGGATAACAGTTGGAAACGATTGCTAATACCAGATGATATGTAAGAAGATACGTTTTCTTAAATTAAAAGGGAGCCTTTTAAAAGCTTCGCTTTGAGATGAGCCTGCGGCGTATTAGCTAGTTGGTAGGGTAATGGCCTACCAAGGCAACGATGCGTAGCCGACCTGAGAGGGTGATCGGCCACATTGGGACTGAGACACGGCCCAAACTCCTACGGGAGACAGCAGTTAGGGATATCGTCAAGTGGGGAAACCCTGAACGAGCAATGCCGCGTGAGTG
>FQH3XDB01EMX91|201|5
GATGAACGCTGGCGGCATGCCTAAGACATGCAAGTCGAACGAAGCGGCCCGATGACATGGAGTGCTTGCACAAAATAGATTCGGATTTCCGCTTAGTGGCGCAAGGGTGAGTAACACGTGGGTAATCTACCTCAGAGACTGGGATAACAGTTGGAAACGATTGCTAATACCAGATGATATGTAAGAAGATACGTTTTCTAAATTAAAGGAAGCCTTTAAAGCTTCGCTTTGAGATGAGCCTGCGGCGTATTAGCTAGTTGGTAGGTAATGGCCTACCAAGGCAACGATGCGTAGCCGACCTGAGAGGGTGATCGGCCACATTGGGACTGAGACACGGCCCAAAACTCCTAACGGGAGACAGCAGTTAGGGATATTCGTCAATGGGGAAACCCTGAACGAGCAATGCCGCGTGAGTGATGACGGTCCTTTGGATTGTAAAACTCTGTT
>FQH3XDB01AUGC8|202|2
GATGAACGCTGGCGGCATGCCTAAGACATGCAAGTCGAACGAAGCGGCCCGATGACATGGAGTGCTTGCACAAAATAGATTCGGATTTCCGCTTAGTGGCGCAAGGGTGAGTAACACGTGGGGTAATCTACCTCAGAGACTGGGGATAACAGTTGGAAACGATTGCTAAATACCAGATGATATGTAAGAAGATTACGTTTTCTTAAAATTAAAAGGAAGCCTTTAAAAGCTTCGCTTTGAGATGAGCCTGCGGCGTATTAGCTAGTTGGTAGGGTAATGGCCTACCAAGGCAACGATGCGTAGCCGACCTGAGAGGGTGATCGGCCACATTGGGGACT
>FQH3XDB01BQD28|203|1
GATGAACGCTGGCGGCATGCCTAAGACATGCAAGTCGAACGAAGCGGCCCGATGACATGGAGTGCTTGCACAAAATAGATTCGGATTTCCGCTTAGTGGCGCAAGGGTGAGTAACACGTGGGTAATCTACCTCAGAGACTGGGATAACAGTTGGAAACGATTGCTAATACCAGATGATATGTAAAGAAGTATACGTTTTCTTAAATTAAAGGAAGCCTTTAAAGCTTCGCTTTGAGATGAGCCTGCGGCGTATTAGCTAGTTGGTAGGGTAATGGCCTACCAAGGCAACGATGCGTAGCCGA
>FQH3XDB01DLMAC|204|9
GATGAACGCTGGCGGCATGCCTAAGACATGCAAGTCGAACGAAGCGGCCCGATGACATGGAGTGCTTGCACAAAGTAGATTCGGATTTCCGCTTAGTGGCGCAAGGGTGAGTAACACGTGGGTAATCTACCTCAGAGACTGGGATAACAGTTGGAAACGATTGCTAATACCAGATGATATGTAAGAAGATACGTTTTACTTAAAATTAAAAGGGAGCCTTTAAAGCTTCGCTTTGAGATGAGCCTGCGGCGTATTAGCTAGTTGGTAGGTAATGGCCTACCAAGGCAACGATGCGTAGCCGACCTGAGAGGGTGATCGGCCACATTGGGACTGAGA
>FQH3XDB01EXU2A|205|1
GATGAACGCTGGCGGCATGCCTAAGACATGCAAGTCGAACGAAGTGGTCCAATGAAGAATGCGTACTTGTACAATTTTGGATTTGGGTTGCCACTTAGTGGCAGACGGGTGAGTAACACGTAGGTAATCTACCTCGGAGACTGGGATAACGTTTGGAAACGAACGCTAATACCGGATGATTCATAATTAGATAACTAGTTATGCTAAAAGGAGCTTCGGCTTCACTTCGAGATGAGCTTGCGGTGTATTAGTTAGTTGGTGGGTAATGGCCTACCAAGACAACGATGCATATCCGAGCTGAGAGGCTGATCGGAC
>FQH3XDB01BMN0D|206|2
GATGAACGCTGGCGGCATGCCTAAGACATGCAAGTCGAACGAAGTGGTCCAATGAAGATGGAGTGCTTGCACAATATTGGATTTGGTTCGCCACTTAGTGGCGGACGGGTGAGTAACACGTGGGTAACCTACCTCGAAGACTGGGATAACTACTGGAAACGGTAGCTAATACCGGATAATTCATATTTGGATAACCAGATATGCTAAAAGGAGCTACGGCTTCACTTCGAGATGGGCTTGCGTTGTATTAGCTAGTTGGTGAGGTAATGGCTCACCAAGGCAACGATGCATATCCGAGCTGAGAGGCTGAACGGACACATTGGGACTGAGACACGGCCCAGACTCC
>FQH3XDB01EASYT|207|5
GATGAACGCTGGCGGCATGCCTAAGACATGCAAGTCGAACGAAGTGGTCCAATGAAGATGGAGTGCTTGCACGAAATCAGATTTGGGTTGCCACTTAGTGGCGAAAGGGTGAGTAACACGTAGGTTATCTACCTTTAAGACTGGAATAACAATTAGAAATGATTGCTAATACCGGATGATATATAGAGTGATACGTCACTTTGTATTAAAAGGAGCCTTTAAAAGCTTCACTTAAAGATGAGCCTGCGACGTATTAGCTAGTTGGTGGGTAAAGGCTTACCAAGGCGACGATGCGTAGCCGACCTGAGAGGGTGATCGGCCACACTGGGACTGAGACACGGCCCAGACTCCTACGGGAGACAGCAGTTAGGAATATTCGTC
>FQH3XDB01EP77F|208|1
GATGAACGCTGGCGGCATGCCTAAGACATGCAAGTCGAACGAAGTGGTCCAATGAAGATGGAGTGCTTGCACAAAGTTGGATTTGGTTCGCCACTTAGTGGCAGACGGGTGAGTAACACGTGGGTAACCTACCTCGGAGACTGGGACAACAGTTGGAAACGACTGCTAATACCGGATAATTCATATTTAGATAACTAGATATGCTAAAAGGAGCTTCGGCTTCACTTCGAGATGGGCTTGCGGCGCATTAGCTAGTTGGTGGGTAATGGCCTACCAAGGCGACGATGCGTATCCGAGCTGAGAGGCTGATCGGACACACTGGGACTGAGACACGGCCCAGACTCCTA
>FQH3XDB01DFW09|209|6
GATGAACGCTGGCGGCATGCCTAAGACATGCAAGTCGAACGAGGTGGCCCAAAGAAGATTGAGTGCTTGCACAAGATTGGACTTGGATTACCACCTAGTGGCGGACGGGTGAGTAACACGTAGGTGACCTGCCTTTGAGTTTGGGATAACTATTAGAAATGATAGCTAATACCGGATTAAATATAGATTCGTTTCTATATTAAAAGGAGCCTTTAAAAGCTTCGCTTAAAGATGGGCCTGCGGCGTATTAGCTAGTTGGTGAGGTAATGGCTTACCAAGGCGACGATGCGTAGCCGAACTGAGAGGTTGATCGGCCACATTGGGACTGAGACACGGGCCCAAAATCCT
>FQH3XDB01CRXY7|210|7
GATGAACGCTGGCGGCATGCCTAAGACATGCAAGTCGAACGAGGTGGCCTTTTGAAGACTGCGTACTTGTACAAAGTTGGAAAGAGATTCTCCACCTAGTGGCAGACGGGTGAGTAACACGTAAGTAATCTACCGTAAAGACTGGGATAACGTTTGGAAACGAACGCTAATACCGGATAACTCATATTTAGATAACTAGATATGCTAAAAGGAGCCTCGGCTTCACTTTACGATGAGCTTGCGGCGTATTAACTAGTTGGTGAGGTAATGGCTCACCAAGGTGACGATGCGTAGCCGAACTGAGAGGTTGATCGGCCACATTGGGACTGAGACACGGCCCAAAATCCTACGGGAGCAGCAGTTAGGAATATT
>FQH3XDB01DX3B7|211|5
GATGAACGCTGGCGGCATGCCTAAGACATGCAAGTCGAACGAGGTGGCCTTTTGAAAGTTGCGTGCTTGCACAAAACCGGATAAAGATAACCACCTAGTGGCAGACGGGTGAGTAACACGTAGGTGACCTACCGTAAAGTTGGGGATAACAGTTGGAAACGATTGCTAATACCGAATAAGATATAATAGTCAATATTATATTGAAAAGGAGCCTTTAAAAGCTTCGCTTAATGATGGGCCTGCGGCGTATTAACTAGTTGGTGAGGTAATGGCTCACCAAGGTGACGATGCGTAGCCGGACTGAGAGGTTGATCGGCCACATTGGGACTGAGACACGGCCCAAAACTCCTACGGGGAGACCAGGCCAGTTAGGAATATCGTCAATGGAGGAAACTCTGAACGAGCAATGCCGCGTGAGTGATGAAGGTCTTTGGATTGTAAAACTCTGTTGTATGTGAAGAA
>FQH3XDB01CJQ4Z|212|1
GATGAACGCTGGCGGCATGCCTAAGACATGCAAGTCGAACGAGACGGCCCATTGAAGGATGAGTGCTTGCACGATTTTGGATTTGGATCACCGTCTAGTGGCGCAAGGGTGAGTAATACGTGGGTAATCTGCCTTCGAGTCTGGAATAACAGTTAGAAATGATTGCTAATGCCGGATTATATATTTTACGATACGTCGTTTAATATTAAAAGGAGCCTTTAAAAGCTTCGCTTGAAGATGAGCCCATGCCGTATTAGCTTGTTGGTGGGTAATGGCCTACCAAGGCGACGATGCGTAGCCGACCTGAGAGGGTGATCGGCCACACTGGGACTGAGACACGGCCCAGACTCCTACGGGAGACAGCAGTTAGGAATATTCGACAATGGAGGAAACTCTGATCGAGCAATGCCGCGTGAAGGATGACGGTC
>FQH3XDB01AVKMB|213|3
GATGAACGCTGGCGGCATGCCTAAGACATGCAAGTCGAACGAGACGGCCCATTGATTATTGAGTGCTTGCACAGGATATGATTTGGATTCCCGTCTAGTGGCGCAAGGGTGAGTAACACGTAGGTAATCTACCTTTGAGTCTGGGATAACGGTTAGAAATGATCGCTAATACCGGATTATATATAGGATGATACGTCATCTTATATTAAAAGGGGCCTTTAAAGCCTCGCTTGAAGATGAGCCTGCGGCGTATTAGCTAGTTGGTGAGGTAATGGCTCACCAAGGCGACGATGCGTAGCCGACCTGAGAGGGTCGATCGGGCCACACTGGGGACTGAGACACGGGCCCAGACTCCTACGGGAGACAGCAGTTAGGAATATTCGTCAATGGGGGAAACCCTGAACGAGCAATGCCGCGTG
>FQH3XDB01DI27C|214|6
GATGAACGCTGGCGGCATGCCTAAGACATGCAAGTCGAACGAGACGGCCCAATGAAGACTGCGTACTTGTACAAAGTTGGATTTGGATTCCCGTCTAGTGGCGCAAGGGTGAGTAACACGTGGGTAATCTACCTTCGAGTCTGGAATAACAGTTAGAAATGATTGCTAATGCCGGATTATATATAGTTAGATATTCTTTCTATATTAAAAGGAGCCTTTAAAGCTTCGCTTGAAGATGAGCCTGCGCCGTATTAGCTAGTTGGTGGGGTAAAGGCCTACCAAGGCGACGATGCGTAGCCGACCTGAGAGGGTGATCGGCCACACTGGGACTGAGACACCGGCCCAGACCTCCGTACGGGAGACAGCAGTTAGGAA
>FQH3XDB01EZI21|215|2
GATGAACGCTGGCGGCATGCCTAAGACATGCAAGTCGAACGAGACGGCCCAATGAAGACTGCGTACTTGTACAAAGTTGGATTTGGATTCCCGTCTAGTGGCGCAAGGGTGAGTAACACGTGGGTAATCTACCTTCGAGTCTGGAATAACAGTTAGAAATGATTGCTAATGCCGGATTATATATAGTTAGATATTCTTTCTATATTAAAAGGAGCCTTTAAAAGCTTCGCTTGAAGATGAGCCTGCGCCGTATTAGCTAGTTGGTGGGGTAAAGGCCTACCAAGGCGACGATGCGTAGCCGACCTGAGAGGGTGATCGGCCACACTGGGGACTGAGACACGGCCCAGACTTCCTACGGGGAGACAGCAGTTAGGAATATTCGTCAATGGGGGAAACCCTGAACGAGCAA
>FQH3XDB01EUGUT|216|1
GATGAACGCTGGCGGCATGCCTAAGACATGCAAGTCGAACGAGACGGCCCAATGAATACTGAGTGCTTGCACGATGTAGGATTTGGATTCCCGTCTAGTGGCGCAAGGGTGAGTAACACGTGGGTAATCTACCTTCGAGTCTGGAATAACAGTTAGAAATGATTGCTAATGCCGGATGATATATAAGACGATACGTCATCTTATATTAAAAGGAGCCTTTAAAGCTTCGCTTGAAGATGAGCCTGCGGCGTATTAGCTAGTTGGTGAGGTAATGGCTTACCAAGGCGACGATGCGTAGCCGACCTGAGAGGGTGATCGGTCACACTGGGACTGAGACACGGCCCAGACTCCTACGGAAGACAGCAGTTAGGAATATTCGT
>FQH3XDB01AOQJ2|217|1
GATGAACGCTGGCGGCATGCCTAAGACATGCAAGTCGAACGAGACGGCCCAATGAAGGTGGAGTGCTTGCACAAAGTTGGATTTGGATTCCCGTCTAGTGGCGCAAGGGTGAGTAACACGTAGGTAATCTACCTTTGAGTCTGGAATAACGATTAGAAATGATCGCTAATGCCGGATTATATATAGGATGATACGTCTTTCTATATTAAAAGGAGCCTTTAAAGCTTCGCTTAAAGATGAGCCTGCGGCGTATTAGCTAGTTGGTGGGGTAATGGCCTACCAAGGCGACGATGCGTAGCCGACCTGAGAGGGTGATCGGCCACACTGGGACTGAGACACGGCCCAGACTCCTACGGGAGACAGCAGTTAGGATATTCGTCAAGTGGGG
>FQH3XDB01DOJND|218|4
GATGAACGCTGGCGGCATGCCTAAGACATGCAAGTCGAACGAGACGGCCCAATGAAAACTGAGTGCTTGCACAAAGTTGGATTTGGTTCACCGTCTAGTGGCGCAAGGGTGAGTAACACGTGGGTAATCTGCCTTCGAGTCTGGAATAACAGTTAGAAATGATTGCTAATACCGGATTATATATAGAACGATACGTCATTCTATATTAAAAGAAGCCTTTAAAGCTTCGCTTGAAGATGAGCCTGCGCCGTATTAGCTAGTTGGTGGGGTAAAGGCCTACCAAGGCGACGATGCGTAGCCGACCTGAGAGGGTGATCGGCCACACTGGGACTGAGACACGGCCCAGACTCCGTACGGGAGACAGCAGTTAGGAATATT
>FQH3XDB01APPTC|219|5
GATGAACGCTGGCGGCATGCCTAAGACATGCAAGTCGAACGAGACGGCCCAATGAAAGTGGAGTGCTTGCACAATGCCGGATTTGGTTTACCGTCTAGTGGCGCAAGGGTGAGTAACACGTGGGTAGTCTACCTTCGAGTCTGGAATAACAGTTAGAAATGATTGCTAATGCCGGATTATATATGTTTAGATACGTCTTTACATATTAAAAGGAGCCTTTAAAAGCTTCGCTTGAAGATGAGCCTGCGGCGTATTAGCTAGTTGGTGGGGTAATGGCCTACCAAGGCAACGATGCGTAGCCGACCTGAGAGGTGATCGGCCACACTGGGACTGAGACACGGCCCAGGACTCCTACGGGGAGA
>FQH3XDB01EJAOK|220|13
GATGAACGCTGGCGGCATGCCTAAGACATGCAAGTCGAACGAGACGGCCCAATGAAAGTGGAGTGCTTGCACAATACCGGATTTGGTTTACCGTCTAGTGGCGCAAGGGTGAGTAACACGTGGGTAATCTACCTTCGAGTCTGGAATAACAGTTAGAAATGATTGCTAATGCCGGATTATATATGTTTAGATACGTCTTTACATATTAAAAGGAGCCTTTAAAGCTTCGCTTGAAGATGAGCCTGCGGCGTATTAGCTAGTTGGTGGGTAATGGCCTACCAAGGCAACGATGCGTAGCCGACCTGAGAGGGTGATCGGCCACACTGGG
>FQH3XDB01EGZ82|221|12
GATGAACGCTGGCGGCATGCCTAAGACATGCAAGTCGAACGAGACGGCCCAATGAATATTGAGTGCTTGCACGATGTAGGATTTGGATTCCCGTCTAGTGGCGCAAGGGTGAGTAACACGTGGGTAATCTACCTTCGAGTCTGGAATAACAGTTAGAAATGATTGCTAATGCCGGATTATATATAAGATGATACGTCATCATATATTAAAAGGAGCCTTTAAAGCTTCGCTTGAAGATGAGCCCGCGGCGTATTAGCTAGTTGGTGAGGTAATGGCTTACCAAGGCGACGATGCGTAGCCGACCTGAGAGGGTGATCGGCCACACTGGGACTGAG
>FQH3XDB01D4PND|222|4
GATGAACGCTGGCGGCATGCCTAAGACATGCAAGTCGAACGAGACGGCCCAATGAATATTGAGTGCTTGCACGATGTAGGATTTGGATTCCCGTCTAGTGGCGCAAGGGTGAGTAACACGTGGGTAATCTACCTTCGAGTCTGGAATAACAGTTAGAAATGATTGCTAATGCCGGATTATATATAAGATGATACGTCATCATATATTAAAAGGAGCCTTTAAAAGCTTCGCTTGAAGATGAGCCCGCGGCGTATTAGCTAGTTGGTGAGGTAATGGCTTACCAAGGCGACGATGCGTAGCCGACCTGAGAGGGTGATCGGCCACACTGGGACTGAGACACGGCCCAGACTCCTACGGGAGACAGCAGTTAGGAATATTCGTCAATGGAGGAAACTCTGAACGAGCAATGCCGCGTGAAGGATGACGGTCCTATGGATTGTAAACTTCTGTTGTTAGGGAACGAACGACC
>FQH3XDB01CAI2C|223|1
GATGAACGCTGGCGGCATGCCTAAGACATGCAAGTCGAACGAGACGGCCCAATGAAAATGGAGTGCTTGCACAAAGTTGGATTTGGTTCACCGTCTAGTGGCGCAAGGGTGAGTAACACGTGGGTAGTCTGCCTTCGAGTCTGGAATAACAGTTAGAAATGATTGCTAATGCCGGATTATATATAGGAAGACATACTTCTTATATTAAAAGGAGCCTTTAAAGCTTCGCTTGAAGATGAGCCTGCGGCGTATTAGCTAGTTGGTGGGGTAACGGCCTACCAAGGCAACGATGCGTAGCCGACCTGAGAGGGTGATCGGCCACACTGGGGACTGAGACACGGCCCCAGACTCCTACGGGAGACAGCAGTTAGGAATATTCGTCAATGGGGGAAACCCTGAACGAGCAATGCCGCGTGAAGGATGACGGTCCTATGGATTGTAAACTTCTGTTGTTAGGGAAGAACGACCTGTATAGGAAATGATACAGGAGTGACGTTACCGG
>FQH3XDB01AM8SH|224|5
GATGAACGCTGGCGGCATGCCTAAGACATGCAAGTCGAACGAGACGGCCCATTGAAGAATGAGTGCTTGCACGATTTCCGATTTGGATCACCGTCTAGTGGCGCAAGGGTGAGTAATACATGGGTAATCTGCCTTCGAGTTTGGAATAACAGTTAGAAATGATTGCTAATGCCGAATTATATATTTTATGATACGTCATTTAATATTAAAAGGAGCCTTTAAAAGCTTCGCTTGAAGATGAGCCCATGCCGTATTAGCTTGTTGGTGGGGTAATGGCCTACCAAGGCAACGATGCGTAGCCGACCTGAGAGGGTGATCGGCCACACTGGGGACTGAGACACGGCCCAGGACTTCCACGGGAGACAGCAGTTAGG
>FQH3XDB01A9HA3|225|8
GATGAACGCTGGCGGCATGCCTAAGACATGCAAGTCGAACGAGACGGCCCATTGAAGAATGAGTGCTTGCACGATTTTGGATTTGGATCACCGTCTAGTGGCGCAAGGGTGAGTAATACGTGGGTAATCTGCCTTCGAGTCTGGAATAACAGTTAGAAATGATTGCTAATGCCGGATTATATATTTTACGATACGTCGTTTAATATTAAAAAGGAGCCTTTAAAAGCTTCGCTTGAAGATGAGCCCATGCCGTATTAGCTTGTTGGTGGGGTAATGGCCTACCAAGGCGACGATGCGTAGCCGACCTGAGAGGGTGATCGGCCACACTGGGACTGAGACACGGCCCAGACTCCTACGGGAGACAGCAGTTAGGAATATTCGACAATGGAGGAAACTCTGATCGAGCAATG
>FQH3XDB01DALD9|226|1
GATGAACGCTGGCGGCATGCCTAAGACATGCAAGTCGAACGAGACGGCCCATTGAAGAATGAGTGCTTGCACGATTTTGGATTTGGATCACCGTCTAGTGGCGCAAGGGTGAGTAATACGTGGGTAATCTGCCTTCGAGTCTGGAATAACAGTTAGAAATGATTGCTAATGCCGGATTATATATTTTACGTATACGTCGTTTAATATAAAGAGCCTTTAAAGCTTCGTCTTGAAGATGAGCCCATGCCGTATTAGCTTGTTGGTGGGTAATGGCCTACCAAGGCGACGATGCGTAGCCGACCTGAGAGGGTGATCCGGCCACACTGGGACTGAGACACGGCCCAGACTCC
>FQH3XDB01B4XZD|227|1
GATGAACGCTGGCGGCATGCCTAAGACATGCAAGTCGAACGAGGCGGCCCAATGAAGAATTTGTACTTGTACAAATTTGGATTTGGATCCCCGCCTAGTGGCGCAAGGGTGAGTAACACGTGGGTAATCTGCCTTTGAGTCTGGAATAACAGTTAGAAATGATTGCTAATGCCGGATGATATATAGAATGATACGTCTTTCTATATTAAAAAGGAGCCTCAAAAACTTCGCTTAAAGATGAGCCTGCGTCGTATTAGCTAGTTGGTGGGGTAAAGGCCTACCAAGGCAACGATGCGTAGCCGACCTGAGAGGGTGATCGGCCACACTGGGGACTGAGACACGGCCCAGACCTCCTACGGGGAGACAGCAGTTAGGAATATTCGTCAAGTGGGGAAACCCTGAACGAGCAATGCCGCGTGAAGGATGACGGTCCTATGGATTGTAAACTTCTGTTGTTAGGAA
>FQH3XDB01A7P26|228|1
GATGAACGCTGGCGGCATGCCTAAGACATGCAAGTCGAACGGAGTGGCCCATTGAAGATTGAGTGCTTGCACAAAGTCGGATTTGGATTTCCACTTAGTGGCGGACGGGTGAGTAACACGTGGGTAATCTACCTCAAAGTTTGGGACAACAGTTAGAAATGACTGCTAATACCTAATGATTTGTAGAAAGATAACTTTTTATACTAAAAGGAGCTTCGGCTTCGCTTTGAGATGAGCTTGCGGTGTATTAGCTTGTTGGTGGGTAATGGCCTACCAAGGCAACGATGCATAGCCGAGCTGAGAGGCTGATCGGCCACACTGGG
>FQH3XDB01EM29X|229|29
GATGAACGCTGGCGGCATGCCTAAGACATGCAAGTCGAACGGAGCGGCCCATTGAAGATTGCGTGCTTGCACAAAATTGGATATGGATTCCCGCTCAGTGGCAAACGGGTGAGTAACACGTGGGTTACCTGCCTCTAAGTTGGGGATAACAGTTGGAAACGATTGCTAATACCGAATGTGCTCTACGGAGTAAAGAAGCCTTTAAAGCTTCGCTTGGAGATGGGCCTGCGGCGCATTAGCTAGGTTGGTGGGGTAATGGCCTACCAAGGCAACGATGCGTAGCCGAACTGAG
>FQH3XDB01CRKK5|230|3
GATGAACGCTGGCGGCATGCCTAAGACATGCAAGTCGAACGGAGCGGCCCATTGAAGATTGCGTGCTTGCACAAAATTGGATATGGATTCCCGCTCAGTGGCAAACGGGTGAGTAACACGTGGGTTACCTGCCTCTAAGTTGGGGATAACAGTTGGAAACGATTGCTAATACCGAATGTGCTCTACGGAGTAAAGAAGCCTTTAAAAGCTTCGCTTGGAGATGGGCCTGCGGCGCATTAGCTAGTTGGTGGGGTAATGGCCTACCAAGGCAACGATGCGTAGCCGAACTGAGAGGTTGATCGGCCACATTGGGACTGAGACACGGCCCAGACCTCCTACGGGAGACAGCAGTTAGGAATATTCGTCAATGGGGAAACCCTGAACGAGCAATGCCGCGTGAGTGATGACGGTCCTCTGGATTGTAAAACTCTGTTGTTTAGG
>FQH3XDB01CP5VN|231|1
GATGAACGCTGGCGGCATGCCTAAGACATGCAAGTCGTACGAGAAGGCCCAAAGAAAGCGGAGTGCTTGCACAAAGTTGGACTTGGATCACCTTCTAGTGGCAAACGGGTGAGTAACACGTGGGTTACCTACCTCTAAGACGGGGATAACAGTTGGAAACGATTGCTAATACCGGATGTGCTCTACGGAGTAAAGAAGCGAATTATCGCTTCGCTTAGAGATGGGCCTGCGGTGCATTAGCTAGTTGGTAGGTAAAGGCCTACCAAGGCGACGATGCATAGCCGAACTGAGAGGTTAATCGGCCACACT
>FQH3XDB01B7B6G|232|3
GATGAACGCTGGCGGCATGCCTAAGACATGCAAGTCGTACGAGAAGGCCCAAAGAAAGCGGAGTGCTTGCACAAAGCTGGACTTGGATCACCTTCTAGTGGCAAACGGGTGAGTAACACGTGGGTTACCTACCTCTAAGACGGGGATAACAGTTGGAAACGATTGCTAATACCGGATGTGCTCTACGGAGTAAAGAAGCGAATTATCGCTTCGCTTAGAGATGGGCCTGCGGTGCATTAGCTAGTTGGTAGGGTAAAGCCTACCAAGGCGACGATGCATAGCCGAACTGAGAGGTTAATCGGCCACACGTGGGACTGAGACACGGCCC
>FQH3XDB01CFR0V|233|5
GATGAACGCTGGCGGCATGCCTAAGACATGCAAGTCGTACGAGAAGGCCCAAAGAAAGCGGAGTGCTTGCACAAAGCTGGACTTGGATCACCTTCTAGTGGCAAACGGGTGAGTAACACGTGGGTTACCTACCTCTAAGACGGGGATAACAGTTGGAAACGATTGCTAATACCGGATGTGCTCTACGGAGTAAAGAAGCGAATTATCGCTTCGCTTAGAGATGGGCCTGCGGTGCATTAGCTAGTTGGTAGGTAAGCCTACCAAGGCGACGATGCATAGCCG
>FQH3XDB01CYR3E|234|1
GATGAACGCTGGCGGCATGCCTAAGACATGCAAGTCGTACGAGAAGGCCCAAAGAAAGTGGAGTGCTTGCACTAAGCTGGACTTGGATCACCTTCTAGTGGCAAACGGGTGAGTAACACGTGGGTTACCTACCTCTAAGACGGGGATAACAGTTGGAAACGATTGCTAATACCGGATGTGCTCTACGGAGTAAAGAAGCGAATTATCGCTTCGCTTAGAGATGGGCCTGCGGTGCATTAGCTAGTTGGTAGGTAAGCCTACCAAGGCGACGATGCATAGCCGAACTGAGAGGTTAATCGGCCACACGT
>FQH3XDB01DDIZN|235|1
GATGAACGCTGGCGGCATGCCTAAGACATGCAAGTCGTACGAGAAGGCCCAAAGAAAGTGGAGTGCTTGCACTAAGCTGGACTTGGATCACCTTCTAGTGGCAAACGGGTGAGTAACACGTGGGTTACCTACCTCTAAGACGGGGATAACAGTTGGAAACGATTGCTAATACCGGATGTGCTCTACGGAGTAAAGAAGCGAATTATCGCTTCGCTTAGAGATGGGCCTGCGGTGCATTAGCTAGTTGGTAGGTAAGCCTACAAGGCGACGATGCATAGCCG
>FQH3XDB01EBS11|236|2
GATGAACGCTGGCGGCATGCCTAAGACATGCAAGTCGTACGAGAAGGCCCAAAGAAAGTGGAGTGCTTGCACAAAGCTGGACTTGGATCACCTTCTAGTGGCAAACGGGTGAGTAACACGTGGGTTACCTACCTCTAAGACGGGGATAACAGTTGGAAACGATTGCTAATACCGGATGTGCTCTACGGAGTAAAGAAGCGGATTATCGCTTCGCTTAGAGATGGGCCTGCGGTGCATTAGCTAGTTGGTAGGTAAGCCTACCAAGGCGACGATGCATAGCCGAACTGAGAGGTTAATCGGCCACGACTGGGACTGAGA
>FQH3XDB01EC2O3|237|4
GATGAACGCTGGCGGCATGCCTAAGACATGCAAGTCGTACGAGAAGGCCCAAAGAAAGTGGAGTGCTTGCACAAAGCTGGACTTGGATCACCTTCTAGTGGCAAACGGGTGAGTAACACGTGGGTTACCTACCTCTAAGACGGGGATAACAGTTGGAAACGATTGCTAATACCGGATGTGCTCTACGGAGTAAAGAAGCGGATTATCGCTTCGCTTAGAGATGGGCCTGCGGTGCATTAGCTAGTTGGTAGGTAAGCCTACCAAGGCGACGATGCATAGCCGAACTGAGA
>FQH3XDB01BIL7A|238|2
GATGAACGCTGGCGGCATGCCTAAGACATGCAAGTCGTACGAAGAAGCCTATTGAAGATTGAGTGCTTGCACAAGATTGGATTTAGATACTTCTTAGTGGCAGACGGGTGAGTAACACGTAGGTAATCTACCTCGAAGACTGGGACAACGTTTGGAAACGAACGCTAATACCGGATAATTCGTAAACGGAAAACTGTTTATGCTAAAAGGTGCTTCGGCATCACTTCGAGATGAGCTTGCGGTGTATTAGCTAGTTGGTGAGGTAATGGCCCACCAAGGCAACGATGCATAGCCGGACTGAGAGGTCGATCGGCCACATTGGGGACTGGAGA
>FQH3XDB01BQ25E|239|1
GATGAACGCTGGCGGCATGCCTAAGACATGCAAGTCGTACGAAGTACCCCAAAGAAGTTTTGAGTGCTTGCACAAGAGACGGACTTGGATCAGTACTTAGTGGCAGACGGGTGAGTAACACGTGGGTAATCTACCTCGAAGACTGGGATAACAATTAGAAATGGTTGCTAATACCGGATAATTCGTAATCGGAAAACTGATTATGCTAAAAGGAGCTTCGGCTTCACTTCGAGATGAGCTTGCGGTGTATTAGCTAGGTTGGTGGGGCAAGTGGCCTACCGAAGGCAACGATGCAT
>FQH3XDB01CHGAB|240|1
GATGAACGCTGGCGGCATGCCTAAGACATGCAAGTCGTACGAAGTGGCCCGTTGAAATTTTGTGCTTGCACAGAATCGATTCGGATTCCCACTTAGTGGCGCAAGGGTGAGTAACACGTGGGTTATCTGCCTTAGAGACTGGGATAACAATTGGAAACGATTGCTAATACCGGATGATATATAAAACGATACGTTGTTTTATATTAAAAGGAGCCTTTAAAGCTTCACTTTAAGATGAGCCTGCGGCGTATTAGCTAGTTGGTAAGGTAATGGCTTACCAAGGCAACGATGCGTAGCCGACCTGAGAGGGTGATCGGCCACACTGGGACTGAGACACGGCCC
>FQH3XDB01BZKL3|241|2
GATGAACGCTGGCGGCATGCCTAAGACATGCAAGTCGTACGAAGTGGCCCATTGATATTTTGTGCTTGCACAAGATTGATTTGGATTCCCGCTTAGTGGCGCAAGGGTGAGTAACACGTGGGTTATCTACCTTGGAGACTGGGATAACAATTGGAAACGATTGCTAATACCGGATTATATGTAAGATGATACGTTGTCTTATATTAAAAGGAGCCTTTTAAAAGCTTCACTTCAAGATGAGCCTGCGGCGTATTAGCTAGTTGGTGGGGTAATGGCCTACCAAGGCAACGATGCGTAGCCGACCTGAGAGGGTGATCGGCCACACTGGGACTGAGACACGGCCCAGACTCCTACGGGAGACAGCA
>FQH3XDB01AKEZZ|242|1
GATGAACGCTGGCGGCATGCCTAAGACATGCAAGTCGTACGAAGTGGCCCATTGATGATGGAGTGCTTGCACAAGATTTGATTTGGATTCCCACTTAGTGGCGCAAGGGTGAGTAACGCGTAGGTTATCTGCCTTCGAGTTTGGAATAACAATTAGAAATGATTGCTAATGCCGGATTATATTTGAGATGATACGTTGTCTTAAATTAAAAGGAGCCTTTTAAAAGCTTCGCTTGAAGATGAGCCTGCGTCGTATTAGCTAGTTGGTAGGGTAATGGCCTACCAAGGCAACGATGCGTAGCCGACCTGAGAGGGTGATCGGCCACACTGGGACTGAGACACGGCCCAGACTCCTACGGGAGGCAGCAGTGGGAATATTGCACAATGGGCGAAAGCCTGATGCAGCAACGCCGCGTGAGTGAA
>FQH3XDB01AK0HY|243|19
GATGAACGCTGGCGGCATGCCTAAGACATGCAAGTCGTACGAAGTGGTCCATTGATAATAGCGTGCTTGCACAATATTTGATTTGGTTCGCCACTTAGTGGCAGACGGGTGAGTAACACGTGGGTAACCTACCCCGAAGACTGGGATAACTACTGGAAACGGTAGCTAATACCGGATAATTCATATTTAGATAACTAGATATGCTAAAAGGAGCTTCGGCTTCACTTCAGGATGGGGCTTGCGTTGTATTAGCTAGTTGGTGAGGTAATGGCTCACCAAGGCAACGATGCATATCCGAGCTGAGAGGCTGAACGGACACAGTTGGGACTGAGACACGGCCCAGACCTCCGTACGGGAGACAGC
>FQH3XDB01DSL97|244|1
GATGAACGCTGGCGGCATGCCTAAGACATGCAAGTCGTACGAAGTGGTCCATTGATAATAGCGTGCTTGCACAATATTTGATTTGGTTCGCTACTTAGTGGCAGACGGGTGAGTAACACGTGGGTAACCTACCCCGAAGACTGGGATAACTACTGGAAACGGTAGCTAATACCGGATAATTCATATTTAGATAACTAGATATGCTAAAAGGAGCTTCGGCTTCACTTCAGGATGGGCTTGCGTTGTATTAGCTAGTTGGTGAGGTAATGGCTCACCAAGGCAACGATGCATATCCGAGCTGAGAGGCTGAACCGGACACATTGGGGACTGAGACACGGCCCAGACTCCTACGGGAGACAGCAGTTAGGAATATTCGTCAAGTGGGGGAAACCCTGAACGAGCAATCGCCGCGTGAGTGATG
>FQH3XDB01BHP6X|245|1
GATGAACGCTGGCGGCATGCCTAAGACATGCAAGTCGTACGAAGTGGCCCATTGATATTTTGTGCTTGCACAAAGTTGATTTGGATTCCCACTTAGTGGCGCAAGGGTGAGTAACACGTGGGTTATCTACCTTGGAGACTGGGATAACAATTGGAAACGATTGCTAATACCGGATGATATATAAGACGATACGTTGTCTTATATTAAAAGGAGCCCTTTAAAAGCTTCACTTCAAGATGAGCCTGCGGCGTATTAGCTAGTTGGTGGGGTAATGGCCTACCAAGGCAACGATGCGTAGCCGACCTGAGAGGTGATCGGCCACACTGGGGACTGAGACACGGCCCAGACTCCTACGGGAGACAGCAGTTAGGGATATT
>FQH3XDB01DKCMF|246|1
GATGAACGCTGGCGGCATGCCTAAGACATGCAAGTCGTACGAGGTGGCCCCGTTGATTTTTGTGCTTGCACAGAAATGATTCGGTTTTCCACCTAGTGGCGCAAGGGTGAGTAACACGTGGGTTATCTGCCTCAGAGACTGGGATAACTATTGGAAACGATAGCTAATACCGGATGATATATAGAACGATACGTTGTTCTATAGTAAAAGGAGCCTTTAAAGCTTCACTTTGAGATGAGCCTGCGACGTATTAGCTAGTTGGTGGGTAAAGGCCTACCAAGGCGACGATGCGTAGCCGACCTGAGAGGGTGATCGGCCACACTGGGGACTGAGACACGGCCCAGACCTCCGTAACGGGAGACAGCAGTTAGGGATATT
>FQH3XDB01BRJEY|247|6
GATGAACGCTGGCGGCATGCCTAAGACATGCAAGTCGTACGAAGCGGCCCACTGAAGTCTGAGTGCTTGCACAAAGATGGATGTGGATTACCGCTTAGTGGCAAACGGGTGAGTAACACGTGGGTTACCTACCTCTAAGTTGGGGATAACAGTTGGAAACGACTGATAATACCGAATGTGCTCTATGGAGTAAAGAGCCCTTAAAGCTTCGCTTAGAGATGGGCCTGCGGCGCATTAGCTAGTTGGTGGGTAATGGCCTACCAAGGCAACGATGCGTAGCCGAA
>FQH3XDB01BGOHV|248|4
GATGAACGCTGGCGGCATGCCTAAGACATGCAAGTCGTACGAAGCGGCCCACTGAAGTCTGAGTGCTTGCACAAAGATGGATGTGGATTACCGCTTAGTGGCAAACGGGTGAGTAACACGTGGGTTACCTGCCTCTAAGTTGGGGATAACATTGGAAACGATTGATAATACCGAATGTGCTCTACGGAGTAAAGAAGCCCTTAAAGCTTCGCTTAGAGATGGGCCTGCGGCGCATTAGCTAGTTGGTAGGGTAATGGCCTACCAAGGCAACGATGCGTAGCCGAACTGAGAGGTTGATCGGCCACAGTTTGGGACTGAGA
>FQH3XDB01CDYJ8|249|4
GATGAACGCTGGCGGCATGCCTAAGACATGCAAGTCGTACGAAGCGGCCCACTGAAGTCTGAGTGCTTGCACAAAGATGGATGTGGATTACCGCTTAGTGGCAAACGGGTGAGTAACACGTGGGTTACCTGCCTCTAAGTTGGGGATAACAATTGGAAACGATTGATAATACCGAATGTGCTCTACGGAGTAAAGAAGCCCTTAAAGCTTCGCTTAGAGATGGGCCTGCGGCGCATTAGCTAGTTGGTAGGTAATGGCCTACCAAGGCAACGATGCGTAGCCGAACTGAGAGGTTGATCGGCCACATTGGGACTGAGACACGGCCCAGACCTCCGTACGGGA
>FQH3XDB01E198W|250|3
GATGAACGCTGGCGGCATGCCTAAGACATGCAAGTCGTACGAAGCGGCCCACTGAAGTCTGAGTGCTTGCACAAAGATGGACGTGGATTACCGCTTAGTGGCAAACGGGTGAGTAACACGTGGGTTACCTACCTCTAAGTTGGGGATAACAGTTGGAAACGACTGATAATACCGAATGTGCTCTACGGAGTAAAGAAGCCCTTAAAGCTTCGCTTAGAGATGGGCCTGCGGCGCATTAGCTAGTTGGTGGGCAATGGCCTACAAGGCAACGATGCGTAGCCGAA
>FQH3XDB01EIT3I|251|7
GATGAACGCTGGCGGCATGCCTAAGACATGCAAGTCGTACGAAGCGGCCCACTGAAGTCTGAGTGCTTGCACAAAGATGGACGTGGATTACCGCTTAGTGGCAAACGGGTGAGTAACACGTGGGTTACCTACCTCTAAGTTGGGGATAACAGTTGGAAACGACTGATAATACCGAATGTGCTCTATGGAGTAAAGAAGCCCTTAAAGCTTCGCTTAGAGATGGGCCTGCGGCGCATTAGCTAGTTGGTGGGGTAATGGCCTACCAAGGCAACGATGCGTAGCCGAACTGAGAGGTTGATCGGCCACATTGGGACTGAGACACGGCCC
>FQH3XDB01D9D9J|252|1
GATGAACGCTGGCGGCATGCCTAAGACATGCGAGTCGAACGAGAAGGCCCAAAGAAAACAGAGTGCTTGCACAAAGTTGGACTTGGATCACCTTCTAGTGGCAAACGGGTGAGTAACACGTGGGTTACCTACCTCTAAGACTGGGATAACAGTTGGAAACGATTGCTAATACCGGATGTGCTCTACGGAGTAAAGGAGCGAATTATCGCTTCACTTAGAGATGGGCCTGCGGTGCATTAGCTAGTTGGTGGGTAATGGCCTACCAAGGCGACGATGCATAGCCGAACTGAGAGGTTAATCGGCCACACTGGGACTGAGACACGGCCCAGACTCCTGCGGGAGACAGCAGTTAGGATATTCGTCAATGGGGAAACCCTGAACGAGCAATGCCGCGTGAGTGATGAA
>FQH3XDB01AJRB8|253|1
GATGAACGCTGGCGGCATGCCTAAGACATGCAAGTCGAACGAGACGGCCCAATGAAGATGGAGTGCTTGCACAAAGTTGGATTTGGATTCCCGTCTAGTGGCGCAAGGGTGAGTAACACGTAGGTAATCTACCTTTGAGTCTGGAATAACGATTAGAAATGATCGCTAATGCCGGATTATATATAGGATGATACGTCTTTCTATATTAAAAGGAGCCTTTAAAGCTTCGCTTAAAGATGAGCCTGCGGCGTATTAGCTAGTTGGTGGGGTAATGGCCTACCAAGGCGACGATGCGTAGCCGACCTGAGAGGGTGATCGGCCACACTGGGACTGAGACACGGCCCAGACTCCTACGGGAGACAGCAGTTAGGAATATTCGTCAATGGGGAAACCCTGAACGAGCAATGCCGCGTGAAGGATGACGGTCCTCTGGATTGTAAACTTCTGTTGTTAGGGAGAACGACCTATGTAGGAATGACATAGGA
>FQH3XDB01AX34C|254|1
GATGAACGCTGGCGGCATGCCTAAGACATGCAAGTCGAACGAGACGGCCCAATGAAGATGGAGTGCTTGCACAAAGTTGGATTTGGATTCCCGTCTAGTGGCGCAAGGGTGAGTAACACGTAGGTAATCTACCTTTGAGTCTGGAATAACGATTAGAAATGATCGCTAATGCCGGATTATATATAGGATGATACGTCTTTCTATATTAAAAGGAGCCTTTAAAGCTTCGCTTAAAGATGAGCCTGCGGCGTATTAGCTAGTTGGTGGGGTAATGGCCTACCAAGGCGACGATGCGTAGCCGACCTGAGAGGGTGATCGGCCACACTGGGACTGAGACACGGCCCAGACTCCGTACGGGAGACAGCAGTTAGGAATATT
>FQH3XDB01BM8I5|255|2
GATGAACGCTGGCGGCATGCCTAAGACATGCAAGTCGAACGAGACGGCCCAATGAAGATGGAGTGCTTGCACAAAGTTGGATTTGGATTCCCGTCTAGTGGCGCAAGGGTGAGTAACACGTGGGTAATCTACCTTTGAGTCTGGAATAACAGTTAGAAATGATTGCTAATGCCGGATTATATATAAGATGATACGTCTTCTTATATTAAAAGGAGCCTTTTAAAAGCTTCACTTAAAGATGAGCTTGCGTCGTATTAGCTAGTTGGTGGGTAAAGGCCTACCAAGGCGACGATGCGTAGCCGACCTGAGAGGGTGATCGGCCACACTGGGACTGAGACACGGCCCAGACTCCTACGGGAGACAGCAGTTAGGAATATTCGTCAATGGGGAAACCCTGAACGAGCAATGCCGCGTGAAGGATGACGGTCCTATGGATTGGTAAACTTCTGTTGTTAGGGAAGAACGACCTAGGTAGAATGACTAGGAGTGACGGTACTT
>FQH3XDB01DYU4C|256|7
GATGAACGCTGGCGGCATGCCTAAGACATGCAAGTCGAACGAGACGGCCCAATGAAGAATGAGTGCTTGCACAATTTTGGATTTGGATTCCCGTCTAGTGGCGCAAGGGTGAGTAACACGTGGGTAATCTACCTTTGAGTCTGGAATAACAGTTAGAAATGATTGCTAATGCCGGATTATATATAGGTTGGTATTCAACTTATATTAAAAGGAGCCTTTAAAAGCTTCGCTTGAAGATGAGCCTGCGGCGTATTAGCTAGTTGGTGAGGTAATGGCTTACCAAGGCGACGATGCGTAGCCGACCTGAGAGGTGATCGGCCACACTGGGGACTGAGACACGGCCCAGACTCCTACGGGAGACAGCAGTTAGGAATATTCGTCAATGGGGGAAACCCTGAACGAGCAATGCCGCGTGAAGGATGACGGTCCTCTGGATTGTAAACTT
>FQH3XDB01ASHPT|257|6
GATGAACGCTGGCGGCATGCCTAAGACATGCAAGTCGAACGAGACGGCCCAATGAAGAATGAGTGCTTGCACGATTTTGGATTTGGATTCCCGTCTAGTGGCGCAAGGGTGAGTAACACGTGGGTAATCTACCTTTGAGTCTGGAATAACAGTTAGAAATGATTGCTAATGCCGGATTATATATAGGTTGGCATTCAACTTATATTAAAAGGGGCCTTTAAAGCTTCGCTTGAAGATGAGCCTGCGGCGTATTAGCTAGTTGGTGAGGTAATGGCTTACCAAGGCGACGATGCGTAGCCGACCTGAGAGGGTGATCGGCCACACTGGGACTGAGACACGGCCCAGACCTCCTACGGGAGACAGCAGTTAGGAATATTCGTCAAGTGGGGGAAACCCTGAACGAGCAATCGCCGCGTGAAGGATGACGGTCCTCGTGGATTGTAAC
>FQH3XDB01EVA80|258|4
GATGAACGCTGGCGGCATGCCTAAGACATGCAAGTCGAACGAGACGGCCCAATGAAGAATGAGTGCTTGCACGATTTTGGATTTGGATTCCCGTCTAGTGGCGCAAGGGTGAGTAACACGTGGGTAATCTACCTTTGAGTCTGGAATAACAGTTAGAAATGATTGCTAATGCCGGATTATATATAGGTTGGCATTCAACTTATATTAAAAAGGAGCCTTTAAAAGCTTCGCTTGAAGATGAGCCTGCGGCGTATTAGCTAGTTGGTGAGGTAATGGGCTTTACCAAGGCGACGATGCGTAGCCGACCTGAGAGGGTGATCGGCCACACTGGGACTGAGACACGGCCCAGACTCCTACGGGAGACAGCAGTTAGGAATATTCGTCAATGGGGAAACCCTGAACGAGCAATGCCGCGTGAAGGATGACGGTCCTCTGGATT
>FQH3XDB01CBHF5|259|3
GATGAACGCTGGCGGCATGCCTAAGACATGCAAGTCGAACGAGACGGCCCAATGAAGATTGCGTGCTTGCACAAAATCAGATTTGGATTCCCGTCTAGTGGCGCAAGGGTGAGTAACACGTGGGTAATCTGCCTTCGAGTCTGGAATAACAGTTAGAAATGATTGCTAATGCCGGATTATATATAGGAGATACACTTCTATATTAAAAGGAGCCTTTAAAGCTTCGCTTGAAGATGAGCCTGCGCCGTATTAGCTAGTTGGTGGGGTAATGGCCTACCAAGACAACGATGCGTAGCCGACCTGAGAGGGTGATCGGCCACACTGGGACTGAGACACGGCCCTAGACTCCTACGGGAGACAG
>FQH3XDB01EN1H2|260|6
GATGAACGCTGGCGGCATGCCTAAGACATGCAAGTCGAACGAGACGGCCCAATGAAGATTGCGTGCTTGCACAAAATCAGATTTGGATTCCCGTCTAGTGGCGCAAGGGTGAGTAACACGTGGGTAATCTGCCTTCGAGTCTGGAATAACAGTTAGAAATGATTGCTAATGCCGGATTATATATAGGAGATACACTTCTATATTAAAAGGAGCCTTTAAAAGCTTCGCTTGAAGATGAGCCTGCGCCGTATTAGCTAGTTGGTGGGGTAATGGCCTACCAAGACAACGATGCGTAGCCGACCTGAGAGGGTGATCGGCCACACTGGGGACTGAGACACGGCCCAGACTCCTACGGGAGACAGCAGTTAGGAATATTCGTCAATGGGGAAACCCTGAACGAGCAATGCCGCGTGAAGGATGACGGTCCTATGGATTGTAAACTT
>FQH3XDB01BZ898|261|7
GATGAACGCTGGCGGCATGCCTAAGACATGCAAGTCGAACGAGACGGCCCAATGAAGACTGAGTGCTTGCACGAAGTTGGACTTGGTTTACCGTCTAGTGGCGCAAGGGTGAGTAACACGTGGGTAATCTGCCTTCGAGTCTGGAATAACAGTTAGAAATGATTGCTAATGCCGGATTATATTTAGAGAGATACGTCTTTCTAAATTAAAAGGAGCCTTTAAAAGCTTCGCTTGAAGATGAGCCTGCGCCGTATTAGCTAGTTGGTGGGTAAAGGCCTACCAAGGCGACGATGCGTAGCCGACCTGAGAGGGT
>FQH3XDB01B0QLX|262|1
GATGAACGCTGGCGGCATGCCTAAGACATGCAAGTCGAACGAGACGGCCCAATGAAGACTGAGTGCTTGCACGAAGTTGGACTTGGTTTACCGTCTAGTGGCGCAAGGGTGAGTAACACGTGGGTAATCTGCCTTCGAGTCTGGAATAACAGTTAGAAATGATTGCTAATGCCGGATTATATTTAGAGAGATACGTCTTTCTAAAATTAAAAGGAGCCTTTAAAGCTTCGCTTGAAGATGAGCCTGCGCCGTATTAGCTAGTTGGTGGGGTAAAGCCTACCAAGGCGACGATGCGTAGCCGACCTGAGAGGGTGATCGGCCACACTGGGACTGAGACACGGCCCAGACTCCTACGGGGAGACAGCAGTTAGGAATATTCGTCAATGGGGAAACCCTGAACGAGCAATGCCGC
>FQH3XDB01EUOCV|263|1
GATGAACGCTGGCGGCATGCCTAAGACATGCAAGTCGAACGAGACGGCCCAATGAAAACTGAGTGCTTGCACAAAGTTGGATTTGGTTCACCGTCTAGTGGCGCAAGGGTGAGTAACACGTGGGTAATCTGCCTTCGAGTCTGGAATAACAGTTAGAAATGATTGCTAATACCGGATTATATATAGAACCGATACGTCATTCTATATTAAAAGAAGCCTTTAAAGCTTCGCTTGAAGATGAGCCTGCGCCGTAATTGCTAGTTGGTGGGGTAAAAGCCTACCAAGGCGACGATTGCGTAGCCGACC
>FQH3XDB01EWIDT|264|2
GATGAACGCTGGCGGCATGCCTAAGACATGCAAGTCGAACGAGACGGCCCAATGAAAACAGAGTGCTTGCACGAAGTTGGATTTGGTTTCCCGTCTAGTGGCGCAAGGGTGAGTAACACGTGGGTAATCTACCTTTGAGTCTGGAATAACAGTTAGAAATGATTGCTAATGCCGGATGATATTTAGAAAGATACACTTACTAAATTAAAAGGAGCCTTTAAAAGCTTCGCTTAAAGATGAGCCTGCGCCGTATTAGTTAGTTGGTGGGGTAAAGGCCTACCAAGGCGACGATGCGTAGCCGACCTGAGAGGGTGATCGGCCACACTGGGACTGAGACACGGCCCAGACTCCTACGGGAGACAGCAGTTAGGAATATTCGTCAAGTGGGG
>FQH3XDB01DQWCD|265|15
GATGAACGCTGGCGGCATGCCTAAGACATGCAAGTCGGACGAGACGGCCCAATGAAAACGGAGTGCTTGCACAAAGTTGGATTTGGTTTCCCGTCTAGTGGCGCAAGGGTGAGTAACACGTGGGTAATCTGCCTTCGAGTCTGGAATAACAGTTAGAAATGATTGCTAATGCCGGATGATATTTAGATAGATACGTCTAACTAAATTAAAAGGAGCCTTTAAAGCTTCGCTTGAAGATGAGCCTGCGCCGTATTAGCTAGTTGGTGGGTAAAGCCTACCAAGGCAACGATGCGTAGCCGACCTGAGAGGTGATCGGCCACACTGGGACTGAGACACGG
>FQH3XDB01BSS9J|266|2
GATGAACGCTGGCGGCATGCCTAAGACATGCAAGTCGTACGAAGCGGCCCAATGAAGATTGCGTGCTTGCACAAAATTGGATTTGGATTTCCGCTTAGTGGCAAACGGGTGAGTAACACGTGGGTGTACCTACCTCTAAGATGGGGGATAACAATTGGAAACGATTGGTAATACCGAATGTGCTCTACGGAGTAAAGGAGCCTTTAAAGCTCCGCTTAGAGATGGGCCTGCGGCGCATTAGCTAGTTGGTGAGATAACGGCCACAAGGCGACGATGCGTAGCC
>FQH3XDB01CM9Z9|267|1
GATGAACGCTGGCGGCATGCCTAAGACATGCAAGTCGTACGAAGCGGCCCACTGATATTTTGTGCTTGCACAAGATTGATTTGGATTTCCGCTTAGTGGCGCAAGGGTGAGTAACACGTGGGTTATCTGCCTCAGAGACTGGGATAACAATTGGAAACGATTGCTAATACCGGATGATATATAAGACGATACGTTGTCTTGTATTAAAAGAAGCCTTTAAAGCTTTACTTTGAGATGAGCCTGCGGCGTATTAGCTAGTTGGTGGGGTAATGGCCTACCAAGGCAACGATGCGTAGCCGACCTGAGAGGGTGATCGGCCACACTGGGACTGAGACACGGCCCAGACCTCCGTACGGGAGACAGCA
>FQH3XDB01CH3J0|268|1
GATGAACGCTGGCGGCATGCCTAAGACATGCAAGTCGTACGAAGCGGCCCATTGATATTTTGTGCTTGCACAAGATTGATATGGATTCCCGCTTAGTGGCGCAAGGGTGAGTAACACGTGGGTTATCTGCCTTAGAGACTGGGATAACAATTGGAAACGATTGCTAATACCGGATGATATGTAAGACGATACGTTGTCTTATATTAAAAGGAGCCTTTAAAAGCTTCACTTTAAGATGAGCCTGCGGCGTATTAGCTAGTTGGTGGGTAATGGCCTACCAAGGCAACGATGCGTAGCCGACCTGAGAGGTGATCGGCCACACTGGGACTGAGACACGGCCCAGACCTCCGTACGGGAGACAGCAG
>FQH3XDB01D6AFI|269|1
GATGAACGCTGGCGGCATGCCTAAGACATGCAAGTCGTACGAAGCGGCCCACTGATATTTTGTGCTTGCACAAGATTGATTTGGATTTCCGCTTAGTGGCGCAAGGGTGAGTAACACGTGGGTTATCTGCCTCAGAGACTGGGATAACAATTGGAAACGATTGCTAATACCGGATGATATATAAGACGATACGTTGTCTTGTAATTAAAAGAAGCCTTTAAAGCTTTACTTTGAGATGAGCCTGCGGCGTATTAGCTAGTTGGTGGGGTAATGGCCTACCAAGGCAACGATGCGTAGCCGACCTGAGAGGGTGATCGGCCACACTGGGA
>FQH3XDB01COSWR|270|1
GATGAACGCTGGCGGCATGCCTAAGACATGCAAGTCGTACGAAGCGGCCCATTGATATTTTGTGCTTGCACAGAATTGATTTGGATTCCCGCTTAGTGGCGCAAGGGTGAGTAACACGTGGGTTATCTGCCTCAGAGACTGGGATAACAATTGGAAACGATTGCTAATACCGGATGATATGTAAGACGATACGTTGTCTTATATTAAAAGGAGCCTTTAAAGCTTCGCTTTGAGATGAGCCTGCGGCGTATTAGCTAGTTGGTGGGGTAATGGCCTACCAAGGCAACGATGCGTAGCCGAACT
>FQH3XDB01BFHS5|271|1
GATGAACGCTGGCGGCATGCCTAAGACATGCGAAGTCGAACGAAACAGACTAATGAAATTCGAGTGCTTGCACGAGAATGGAAATAGAACCTGTTTAGTGGCGGACGGGTGAGTAACACGTGGGTGATCTGCCTTTAAGACTGGGATAACAATTAGAAATGATTGCTAATACCGGATGATATGTATATTCGGTTATACATTAAAAGTTGCTTTCGGGCAATACTTAAAGATGAGCCTGCGGCGTATTAGCTAGTTGGTAAGGTAATGGCTTACCAAGGCGACGATGCGTAGCCGGACCGAGAGGTTGAAACGGTCACACTGGAACTGAGACA
>FQH3XDB01BL8G0|272|1
GATGAACGCTGGCGGCATGCCTAAGACATGCAAGTCGTACGAAGCGGCCCATTGATATTTTGTGCTTGCACAAGATTGATATGGATTCCCGCTTAGTGGCGCAAGGGTGAGTAACACGTGGGTTATCTGCCTTAGAGACTGGGATAACAATTGGAAACGATTGCTAATACCGGATGATATGTAAGACGATACGTTGTCTTATATTAAAAGGAGCCTTTAAAGCTTCACTTTAAGATGAGCCTGCGGCGTATTAGCTAGTTGGTGGGTAATGGCCTACCAAGGCAACGATGCGTAGCCGACCT
>FQH3XDB01DN22N|273|1
GATGAACGCTGGCGGCATGCCTAAGGCATGCAAGTCGAACGAAGTGGCCCAATGAAGAATGTGAGCTTGCTCAAATTTAGATTTGGATTACCACTTAGTGGCGCAAGGGTGAGTAACGCGTGGGTAATCTACCTTCGAGTCTGGAATAACAGTTAGAAATGATTGCTAATGCCGGATGAAAATGGGAAAACGATACGTCTATTTTTATAAAAGGTGCCTTTAAAGCATCGCTTGAAGATGAGCCTGCGTCGTATTAGTTAGTTGGTGGGTAACGCCTACCAAGACGACGATGCGTAGCCGACCTGAGAGGGTGATCGGCCACACTGGGACTGAGACACGGCCCA
>FQH3XDB01B9TAW|274|55
GATTAACGCTGGCGGCATGCCTAATACATGCAAGTTGAACGAGAAGTAGCAATACTTCTAGTAGCGAACGGGTGAGTAATACATAAGTAACCTGCCTTCTTGATGGGGATAACTGATCGAAAGATTGGCTAATACCGAATGAACTGATTGATATGCATATATTAATGAGGAAAGATGAGATCGCAAGGAGATGGGCTTATGGCGCATTAGCTAGTTGAGGGGTAATGGCCCGCCAAGGCGACGATGCGTAGCCGACCTGAGAGGGTGAACGGCCACACTGGAACTGAGACACGGTCCAGACTCCTAC
>FQH3XDB01AMSNO|275|1
GATGAACGCTGGCGGCATGCCTAAGGACATGCAAGTCGAACGAAGGGACCCAATGAAAATGGAGTGCTTGCACAAAGTTGGATTTGGATTTTCCCTTAGTGGCAAACGGGTGAGTAACGCGTGGGTTACCTACCTCTAAGTTGGGGATAACAGTTGGAAACGATTGCTAATACCGAATGTGTTCTACGGAATAAGAAGCCCTTAAAGCTTCGCTTAGAGATGGGTCTGCGTTGTATTAGCTAGTTGGTGGGTAACGGCCTACCAAGGCGACGATGCATAGCCGAGCTGAGAGGTTAATCGGCCACACTGGGACTGAGACACGGCCCAGACTCCGTACGGAGACAGCAGTTAGGATATCGTCAA
>FQH3XDB01CZ8YD|276|1
GATGAACGCTGGCGGCGTGCATAACACATTCAAGTCGAGCGGTGAAACTTCTTCGGAAGTGGATCAGTGGCGGACGGGTGAGTAACGCGTGAGCAACCTGCCTACGAATGGGGGATAACACAGGGAAACTTGTGCTAATACCGCATGACATATACTTTTCGCATGTAAGGTATATCAAAGGGAAACCGGTCGTAGATGGGCTCGCGTCTGATTAGGTAGTTGGTGAGGTAAGAGCCTACCAAGGCGACGATGCGTAGCCGAACTGAGAGGTTGATCGGCCACACTGGGACTGAGACACGGCCCAGACTCCTACGGGAGACAGCAGTTAGGAATATTCGTCAATGGGGGGAACCCTGAACGAGCAATGCCGCGTGAATGAAGAAGGCCCTATGGGTTGTAAAATTCTGTTGTTTGGAAAGAATGTAAAGTTAGGAAATGAACTTTACTTGACGGTACCATTCAA
>FQH3XDB01E2IQL|277|1
GATGAACGCTGGCGGCGTGCCTAACACATGCAAGTCGAACGGAGCACTGAGACTTCGGTTTTTGTGCTTAGTGGCGGACGGGTGAGTAACGCGTGAGCAATCTGCCTTTCAGAGGGGGATAACGATTGGAAACGATCGCTAATACCGCATAACACATTTGGATGGCATCTTCCGAATGTCAAAGGAGCAATCCGCTGAAAGATGAGCTCGCGTCTGATTAGATAGTTGGTGAGGTAACGGCCTACCAAGGCGACGATGCGTAGCCGAACTGAGAGGTTGATCGGCCACACTGGGACTGAGACACGGGCCCCAGGACTCCCTACGGGGAGACAGCAGTTAGGAATATTCGTCAATGGGGGGAACCCTGAACGAGCAATGCCGCGTGAATGAAGAAGGCCCTATGGGTTGTAAAATTCTGTTGTTTGGAAAGAAATGTAAAGTTAGGAAACTGAACTTTACTTGACGGTACCATTCAAGAAAGCCAACGGCTAACTACGTGCCAG
>FQH3XDB01A6A14|278|3
GATGAACGCTGGCGGCGTGCCTAACACATGCAAGTCGAACGGAGTTGCAGGGAGCGGAGATTTCGGTCAAAGCAATCTGTAACTTAGTGGCGGACGGGTGAGTAACGCGTGGATAACCTGCCGTATACAGGGGGATAACACTTAGAAATAGGTGCTAATACCGCATAAGCGCACAGCTTTGCATGAAGCAGTGTGAAAAAGCCGAGACGGTATACGATGGATCCGCGTCTGATTAGATAGTTGGCGGGTAAAGGCCCACCAAGTCGACGATCAGTAGCCGGCCTGAGAGGGTGGACGGCCACATTGGGACTGAGACACGGGCCCAAAACTCCTACGGGAGGCAGCAGTGGGGGATATTGCACAATGGGGGAAACCCTGATGCAGCGACGCCGCGTGAGTGAAGAAGTATTTCGGTATGTAAAGCTCTATCAGCAGGGAAGAAGAAATGACGGTACCTGA
>FQH3XDB01BNXTI|279|1
GATGAACGCTGGCGGCGTGCCTAACACATGCAAGTCGAACGAAGCGTCTCGATATTTTCGGATGGAGAGATGACTGAGTGGCGGACGGGTGAATAACACGTGGGTAACCTACCTCTAAGTTGGGGATAACAGTTGGAAACGACTGATAATACCGAATGAGCTCTACGGAGTAAAGAAGCCATCAAGCTTCGCTAAGAGATGGGCCTGCGGCGTATTAGCTAGTTGGTGGGGTAAGAGCCTACCAAGGCGACGATGCGTAGCCGAACTGAGAGGTTGATCGGCCACACTGGGACTGAGACACGGCCCAGACTCCTACGGGGAGACAGCAGTTAGGAATATCGTCAATGGGGGAACCCTGAACGAGCAATGCCGCGTGAAT
>FQH3XDB01BNY02|280|3
GATGAACGCTGGCGGCGTGCCTAACACATGCAAGTCGAACGGGTTTACTTTTTGAAGTTTTCGGATGAATAAAAAGCAAACTAGTGGCGGACGGGTGAGTAACGCGTGAACAATTTGCCTTAGAGAGGGGGATAACAAGTAGAAATATTTGCTAATACCGCATAAGACCACAACTCGGCATCGGGAAGGGGTAAAAGAGCAATCCGCTTTAAGATAAGTTTGCGTATCATTAGTTAGTTGGTGAGGTAATGGCCCACCAAGACTACGATGGTTAGCCGACCTGAGAGGGTGATCGGCCACATTGGAACTGAGAACGGTCCAGACTCCTACGGGAGGCAGCAGTGGGGAATATTGGGCAATGGAGGCAACTCTGACCCAGCAACGCCGCGTGAAGGATGAAGGTCTTCGGATTGTAAACTTCTTTATAGGGAAAAAAGAAGTGATGGTACCCTATGAATAAGCTACGGCAAACTATGTGCCAGCAG
>FQH3XDB01BDEAQ|281|2
GATGAACGCTGGCGGCGTGCCTAACACATTCAAGTCGAACGGTGAAATGCCTTCGGGCATGGAACAGTGGCGGACGGGTGAGTAACGCGTGAGCAATCTGCCTGTACGAGTGGAATAACAGCGGGAAACTGCTGCTAATACCGCATAATGCATGGGGCTTGCATGGGCCTTATGCCAAAGATTTATCGCGTACAGATGAGCTCGCGTCCGATTAGCCAGTTGGCGGGGTAAAAGCCCACAAAGCGACGATCGGTAGCCGGACTGAGAGGTTGAACGGCC
>FQH3XDB01C3I7T|282|8
GATGAACGCTGGCGGCGTGCCTAATACATGCAAGTCGAACGAGGTGTAGCAATACACCTAGTGGCGAACGGGTGAGTAACACGTAGGTAACCTGCCTTTCAGCTTGGGATACCCGAGGGAAACCTTGGCTAATACCGGATAACTGATTTAGAGGCATCTCTGAATCCTAAAAGGGGCTCCAAAGCCTCACTGAAAGATGGACTTGCGGCGCATTAGTTAGTTGGTGAGATAACAGCCCACCAAGGCGAGGATGCGTAGCCGACCTGAGAGGGTGATCGGCCACACTGGAACTGAGACACGGTCCAGACTCCTACGGGAGGCAGCAGTAGGGAGTTTTCGGCAAGTGGGGAAACCCTGACCGAGCAACGCC
>FQH3XDB01BZJSX|283|9
GATGAACGCTGGCGGCGTGCCTAATACATGCAAGTCGAACGGATGAGGTAGCAATACCGATTCAGTGGCGAACGGGTGAGTAACACGTAGGCAACCTGCCTCTACGAAGGGGATAACCGAGGGAAACTTTGGCTAATACCGTATATGTTTCTTCTAGGGCATCTTAGAAGAAGAAAAGGAGCGATCCGCGTAGAGATGGGCCTGCGGTGTATTAGTTAGTAGGTGAGGTAACGGCTCACCTAGACGATGATACACAGCCGACCTGAGAGGGTGATCGGCCACATTGGGACTGAGACACGGCCCAAAACTCCTACGGGAGGCAGCAGTAAGGAATTTTCGGCAATGGG
>FQH3XDB01AHAVU|284|35
GATGAACGCTGGCGGCGTGCCTAATACATGCAAGTCGAGCGAGAGACCCTTCGGGGAATCGAGCGGCGAACGGGTGAGTAACACGTAGGTAACCTGCCTTTTAGTTTGGGATACCCGATGGAAACGTCGGCTAATACCGGATACGTACTTTGGAGGCATCTCCGAAGTATAAAAGGGGCCTTAAAGCCTCGCTAATAGATGGACCTGCGGCGCATTAGTTAGTTGGTGAGATAACAGCCCCACCAAGACGAGGATGCGTAGCCGACCTGAGAGGGTGATCGGCCACATTGGGACTGAGACACGGCCCAGACTCCTACGGGAGGCAGCAGTAGGGAGTTTTCGGCAATGGGGGAAACCCTGACCGAGCAACGCCGCGTGAGTGATGAAGGTCTTCGGATCGTAAAGCTCTGTTGTGAAAGACGAATGACAGTACTCAACAAGGAAGTGAACGGCTAACTACGTGCCAGCAGCCG
>FQH3XDB01BS7MR|285|17
GATGAACGCTGGCGGCGTGCCTAATACATGCAAGTTGAACGAGTGAGGACTTCGGTCCAAGCTAGTAGCGAACGGGTGAGTAACACGTAGGCAACCTGCCCCATGGTGGGGATAACTAAGGGAACTTTAGCTAATACCGCATAGATGCAACCAGGCATCTGGTTGAGGAGAAAGGTCCTGCAAGGGACTGCGATGGGATGGGCCTGCGGCGCATTAACTAGTTGGTGAGGTAGAGGCTTACCAAGGTAATGATGCGTAGCCGATCTGAGAGGATGACCGGCACACTGGAACTGAGACACGGTCCAGACTCCTACGGGAGGCAGCAGTAGGAATTTTCGGCAATGGAGGGAACTCTGACCGAGCAAACGCCGCGTGAACG
>FQH3XDB01B2SA2|286|1
GATGAACGCTGGCGGCGTGCCTAATACATGCAAGTTGAACGAGTGAGGACTTCGGCCCAAGCTAGTAGCGAACGGGTGAGTAACACGTAGGCAACCTGCCCCATGGTGGGGATAACTAAGGGAACTTTAGCTAATACCGCATAGATGCAACCAGGCATCTGGTTGAGGAGAAAAGGTCCTGCAAGGGCTGCGATGGGATGGGCCTGCGGCGCATTAACCTAGTTGGTGAGGTAGAGGCTTACCAAGGTAATGATGCGTAGCCGATCTGAGAGGATGACCGGCCACACTGGAACTGAGACACGGTCCAGACTCCTACGGGAGGCAGCAG
>FQH3XDB01B7L40|287|51
ATTGAACGCTGGCGGCATGCCTTACACATGCAAGTCGAACGGCAGCGCGGGAGCTTGCTCCTGGCGGCGAGTGGCGAACGGGTGAGTAATACATCGGAACGTGTCCGTTTGTGGGGGACAACCAGCCGAAAGGTTGGCTAATACCGCATAAGACCTGAGGGTGAAAGCCGGGGATCGCAAGACCTGGCGCAGACGGAGCGGCCGATGATTGATTAGCTAGTTGGCGAGGTAAAGGCCCACCAAGGCGACGATCAATAGCTGGTCTGAGAGGACGACCAGCCACACTGGAACTGAGACACGGTCCAGACTCCTACGGGAGGCAGCAGTGGGGAATTTTGGACAATGGGGGCAACCCTGATCCAGCCATGCCGCGTGCGGGAAGAGGCCTTCGGGTTGTAAAGATCTTTAATCAGGGACGAAATAATGACGGTACCTGAAGAATAAGCTCCGGCTAACTACGTGCCAGCAGCC
>FQH3XDB01DK9UX|288|1
GATGAACGCTGGCGGCGTGCTTAACACATGGCAAGTCGAATGTAGTTTACTACATGGCGGACGGGTGAGTAACACGTGAGCAATCTGCCCATATCTGGGGGATAACCGTTGGAAACGACGGATAATACCGCATAATATCATTTGAAGGCATCTTCTTATGATCAAAGATTTATCGGATATGGATGAGCTCGCGTCTGATTAGGCTGTTGGTGAGGTAACGGCTCACCAAACCGCGATCAGTAGCCGACCTGAGAGGGTGATCGGCCACATTGGGACTGAG
>FQH3XDB01EURQN|289|37
GATGAACGCTGGCGGCGTGCTTAACACATGCAAGTCGAATGTAGTTTACTACATGGCGGACGGGTGAGTAACACGTGAGCAATCTGCCCATATCTGGGGGATAACCGTTGGAAACGACGGATAATACCGCATAATATCATTTGAAGGCATCTTCTTATGATCAAAAGATTTATCGGATATGGATGAGCTCGCGTCTGATTAGGCTGTTGGTGAGGTAACGGCTCACCAAACCGACGATCAGTAGCCGACCT
>FQH3XDB01CAS5D|290|138
GATGAACGCTGGCGGCGTGCTTAACACATGCAAGTCGAATGTAGTTTACTACATGGCGGACGGGTGAGTAACACGTGAGCAACCTGCCCATATCTGGGGGATAACCGTTGGAAACGACGGATAATACCGCATAATATTATTTGAAGGCATCTTCTTATAATCAAAGATTTATCGGATATGGATGGGCTCGCGTCTGATTAGGCTGTTGGTGAGGTAACGGCTCACCAAACCGCGATCAGTAGCCGACCTGAGAGGGTGATCGGCCACATTGGGACTGAGACACGGCCC
>FQH3XDB01EM616|291|7
GATGAACGCTGGCGGCGTGCTTAACACATGCAAGTCGAATGTAGTTTACTACATGGCGGACGGGTGAGTAACACGTGAGCAACCTGCCCATATCTGGGGGATAACCGTTGGAAACGACGGATAATACCGCATAATATTATTGAAGGCATCTTCTTATAATCAAAGATTTATCGGATATGGATGGGCTCGCGTCTGATTAGGCTGTTGGTGAGGTAACCGGCTCACCAAACCGACGATCAGTAGCCGACCTGAGAGGGTGATCGGCCACATTGGGACTGAGACACGGCCCAAAACTCCTACGGGAGGCAGCAGTGGGGAATATTGGTCAATGGAGGAAACTCTGAACC
>FQH3XDB01AUFJY|292|4
GATGAACGCTGGCGGCGTGCTTAACACATGCAAGTCGAATGTAGTTTACTACATGGCGGACGGGTGAGTAACACGTGAGCAATCTGCCCATATCTGGGGGATAACCGTTGGAAACGACGGATAATACCGCATAATATCATTTGAAGGCATCTTCTTATGATCAAAGATTTATCGGATATGGATGAGCTCGCGTCTGATTAGGGCTTGGTTGGTGAGGTAACGGCTCACCAAACCGACGATCAGTAGCCGA
>FQH3XDB01AWMVF|293|13
GATGAACGCTGGCGGCGTGCTTAACACATGCAAGTCGAATGTAGTTTACTACATGGCGGACGGGTGAGTAACGCGTGAGCAATCTGCCCATATCTGGGGGATAACCATTGGAAAACGACGGATAATACCGCATAATATCACGGGAAGGCATCTTCCTGTGATCAAAGATTTATCGGATATGGATGAGCTCGCGTCTGATTAGGCTGTTGGTGAGGTAACGGCTCACCAAACCGACGATCAGTAGCCGACCTGAGAGGGTGATCGGCCACATTGGGACTGAGACACGGCCCAAACTCCTACGGGAGGCAGCAGTGGGGAATATTGGGTCAATGGAGGAAACTCTGAACCAGCAACGCC
>FQH3XDB01DCF78|294|1
GATGAACGCTGGCGGCGTGCTTAACACATGCAAGTCGAATGTAGTTTTACTACATGGCGGACGGGTGAGTAACACGTGAGCAACCTGCCCATATCTGGGGGATAACCGTTGGAAACGACGGATAATACCGCATAATATTATTTGAAGGCATCTTCTTATAATCAAAAGATTTTATCGGATATGGATGGGCTCGCGTCTGATTAGGCTGTTGGTGAGGTAACGGCTCACCAAAACCGACGATCAGTAGCCGACCTGAGAGGGTGATCGGCCACATTTGGGGACCTGAG
>FQH3XDB01C3M6L|295|1
GATGAACGCTGGCGGCGTGCTTAACACATGCAAGTCGAACGGGCGTAGCAATACGTCAGTGGCGAACGGGTGAGTAACGCGTGAGCAATCTGCCCATATCTGGGGAATAGCGGTTGGAAACGACCGGTAATACCGCATGGCATCCTTTGGAGGCATCTCCGTTGGATTAAAGATTTATCGGATATGGATGAGCTCGCGTCCTATTAGGTAGTTGGTGAGGTAACGGCTCACCAAGCCGACGATGGGTAGCCGGCCTGAGAGGGTGATCGGCCACATTGGAACTGAGACACGGTCCAAAACTCCTACGGGAGGCAGCAGTGGGGAATATTGGGCAAGTGGGCGAAAGCCTGACCCAGCAACGCCGCGTGAGTGATGAAGGTCTTCGGATTGTAAAACTCTTAAGCAGGGAAGAAGAAGTGACGGTACCTGC
>FQH3XDB01EIN28|296|1
GATGAACGCTGGCGGCGTGCTTAACACATGCAAGTCGAACGGACTTTTAATGAAACCTGGTGATTTAAAAGTTAGTGGCGGACGGGTGAGTAACGCGTGGATAACCTGCCGTATACAGGGGGATAACACTTAGAAATAGGTGCTAATACCGCATAAGCGCACAGCTTTGCATGAAGCAGTGTGAAAAACTCCGGTGGTATACGATGGATCCGCGTCTGATTAGCTGGTTGGCGGGGTAACGGCCCACCAAGGCGACGATCAGTAGCCGGCCTGAGAGGGTGGACGGCCACATTGGGACTGAGACACGGCCCCAAACTCCTACGGGAGGCAGCAGTGGGGAATATTGCACAATGGGGGAAACCCTGATGCAGCGACGCCGCGTGAGTGAAGAGTATCGTGCGTAAGCTCTATCAGC
>FQH3XDB01DQMID|297|1
GATGAACGCTGGCGGCGTGCTTAACACATGCAAGTCGAACGAAGCACTTTTACGGAGACTCTTCGGAGAGGAAGTAAAAGTGACTGAGTGGCGGACGGGTGAGTAACGCGTGGGTAACCTGCCTTACACAGGGGGATAACAGTCGGAAACGATTGCTAATACCGCATAAGCGCACAGTGCTGCATGGCACAGTGTGAAAAACTGAGGTGGTGTAAGATGGACCCGCGTCTGATTAGGTAGTTGGTAGGGTAAAGGCCTACCAAGCCGACGATCAGTAGCCGGCCTGAGAGGGTGGACGGCCACATTGGGACTGAGACACGGCCCAGACTCCTGCGGGAGCAGCAGGTGGGGAATAGTTGCACAAGTGGGCGAAAGCCTGATGCAGCGAC
>FQH3XDB01EOPIU|298|16
ATTGAACGCTGGCGGCGTGCTTAACACATGCAAGTCGAACGCGAAAGTTCCTTCGGGGACGAGTAGAGTGGCGCACGGGTGAGTAACGCGTGGACAATCTGCCTTTCTGACGGGGATAACAGTTGGAAACGACTGCTAATACCGGATACGCTCATGTTGAACTATGTGAGGAAAGACGGCCTCTGCCTGCAAGCTGTCGCAGAAAGATGAGTCCGCGTCCCATTAGCTGGTTGGCGGGGTAACGGCCCACCAAGGCGACGATGGGTAGCCGATTTGAGAGGATGATCGGCCACACTGGAACTGAAACACGGTCCAGACTCCTACGGGAGGCAGCAGTGGGGAATAGTTGCGCAATGGGCGAAAGCCTGACGCAGCGACG
>FQH3XDB01DOQES|299|1
GATGAACGCTGGCGGCGTGCTTAACACATGCAAGTCGAACGGAGTTGAGGAGAGCTTGCTTTTCTTAACTTAGTGGCGAACGGGTGAGTAACGCGTGAGTAACCTGCCCTGGAGTGGGGGACAACAGTTGGAAACGACTGCTAATACCGCATAAGCCCACGGGTCCGCATGGACTTGAGGGAAAAGGATTTATTCGCTTCAGGATGGACTCGCGTCCAATTAGGTAGTTGGTGAGGTAATGGCCCACCAAGCCGACGATTGGTAGCCGGACTGAGAGGTTGAACGGCCACATTGGGACTGAGACACGGCCCAGACTCTCCTCTCCCGACCGTCG
>FQH3XDB01DHXQY|300|3
GATGAACGCTGGCGGCGTGCTTAACACATGCAAGTCGAACGAAGCACTTTATTACGATCTTTTCGGAGTGACGATTTAGTGACTTAGTGGCGGACGGGTGAGTAATGTATAAGAAACCTACCTCTGAGTGGGGAATAACAGTCCGAAAGGATTGCTAATACCCCATAACATCTTTAGGCGGCATCGTTTAGAGATCAAAAGATTTATCGCTTGGAGACGGTCTTATATTCTATCAGCTAGTTGGTGGGGTAACGGCCTACCAAGGCTACGACGGATAGCCGGCCTGAAAGGGCGACCGGCCACAAGGGCACTGAGACACGGGCCCTACTCCTACGGGAGGCAGCAGTGGGGAATTTTGGACAATGGGGCGAAAGCCTGATCCAGCAACGATGCGTGGAGGATGAAGGTTCTCGGATTGTAAACTCTTTGAGAGGGAAAGAAAAAATGAACGGTACCTCTCGAATAAGCCACGGCTAACTACGTGCCAGCAGCCGC
>FQH3XDB01A69RW|301|11
GATGAACGCTGGCGGCGTGCTTAATACATGCAAGTCGAGCGAGGTTCTTCGGAACCTAGCGGCGAACGGGTGAGTAACACGTAGGTAATCTGCCCTAAAGACCGGGATACCATGAGGAAACTTATGCTAATACCGGATATGAAGATGAGAGGCATCTTGAAACTTTGAAAGTTCCGAATGGAACACTATAGGATGAACCTGCGGCGCATTAGTTAGTTGGTGAGATAACAGCCCACCAAGACGAGGATGCGTAGCCGACCTGAGAGGGTGATCGGCCACAATGGAACTGAGACACGGTCCATACTCCTACGGGAGGCAGCAGTAAGGAGTTTTCGGCAAGTGGGGAAACCCTGACCGAGCAACGCCGCGTGAATGATGAA
>FQH3XDB01DBR28|302|1
GATGAACGCTGGCGGCGTGCTTAATACATGCAAGTCGAGCGAGGTTCTTCGGAACCTAGCGGCGAACGGGTGAGTAACACGTAGGTAATCTGCCCTAAAGCTGGGATACCATGAGGAAACTTATGCTAATACCGGATATGAAGATGAGAGGCATCTTGAAACTTTGAAATTCCGAATGGAACACTATAGGATGAACCCTGCGGCGCTATTAGTTAGTTGGTGAGATAACAGCCACCAAGACGAGGATGCGTAGCC
>FQH3XDB01CU10D|303|4
GATGAACGCTGGCGGCGTGCTTAATACATGCAAGTCGAGCGAGGGGTTTCGGCCCCTAGCGGCGAACGGGTGAGTAACACGTAGGTAATCTGCCCTATAGACTGGGATACCTTGAGGAGACTTAAGCTAATACCGGATATGAATAGGAAAGGCATCTTAGCTATTTGAAAGTTCCGGATGGAACACTATAGGATGAACCTGCGGCGCATTAGTTAGTTGGTGAGATAACAGCCCACCAAGACGAAGATGCGTAGCCGACCTGAGAGGGTGATCGGCCACAATGGAACTGAGACACGGTCCATACTCCTACGGGAGGCAGCAGTAAGGAGTTTTCGGCAAT
>FQH3XDB01AZAI7|304|1
GATGAACGCTGGCGGTGTGCCTAATACATGCAAGTCGAGCGAGTGAGCCCTTCGGGGCAAGCTAGCGGCGAACGGGTGAGTAACACGTAGGTAACCTGCCTATTAGCCTGGAATACCCCGAGAGAAATCTCGGCTAATGCCGGATACGTGTTTGGGAGGCATCTCCCAAAACATAAAAAGGGGGCAATTGCTCCACTAGTAGATGGACCTGCGGCGCATTAGTTAGTTGGTGAGATAACAGCCCACCAAGACGAGGATGCGTAGCCGACCTGAGAGGGTGATCGGCCACAATGGAACTGAGACACGGTCCATACTCCTACGGGAGGCAGCAGTAGGGAGTTTTCGGCAAGTGGGGGAAAACCCCTGGACCGAGCAACACCGCGTGAGTGATGAAGGT
>FQH3XDB01CHLPM|305|15
GATGAACGCTGGCGGTGTGCCTAATACATGCAAGTCGAGCGAGTGAGCCCTTCGGGGCGAGCTAGCGGCGAACGGGTGAGTAACACGTAGGTAACCTGCCTATTAGCCTGGAATACCCGAGAGAAATCTCGGCTAATGCCGGATACGTGTTTGGGAGGCATCTCCCAAACATAAAAAGGGGCAATTGCTCCACTAGTAGATGGACCTGCGGCGCATTAGTTAGTTGGTGAGATAACAGCCCACCAAGACGAGGATGCGTAGCCGACCTGAGAGGGTGATCGGCCACAATGGAACTGAGACACGGTCCATACTCCTACGGGAGCAGCAGTAGGGAG
>FQH3XDB01E112C|306|26
GATGAACGCTGGCGGTGTGCCTAATACATGCAAGTCGAGCGAGTGAGCCCTTCGGGGCGAGCTAGCGGCGAACGGGTGAGTAACACGTAGGTAACCTGCCTATTAGCCTGGAATACCCGAGAGAAATCTCGGCTAATGCCGGATACGTGTTTGGAAGGCATCTTCCAAACATAAAAGGGGCAATTGCTCCACTAGTAGATGGACCTGCGGCGCATTAGTAGTTGGTGAGATAATAGCCCACCAAGACGAGGATGCGTAGCCGACCTGAGAGGGTGATCGGCCACAATGGAACTGAGACACGGTCCATACTCCTACGGGAGCAGCAGTA
>FQH3XDB01AL7X3|307|10
GATGAACGCTGGCGGTGTGCCTAATACATGCAAGTCGAGCGAGTGAGCCCTTCGGGGCGAGCTAGCGGCGAACGGGTGAGTAACACGTAGGTAACCTGCCTATTAGCCTGGAATACCCCGAGAGAAATCTCGGCTAATGCCGGATACGTGTTTGGAAGGCATCTTCCAAACATAAAAGGGGCAATTGCTCCACTAGTAGATGGACCCTGCGGCGCATTAGTTAGTTGGTGAGATAATAGCCCACCAAGACGAGGATGCGTAGCCGACCTGAGAGGG
>FQH3XDB01BSVDN|308|1
GATGAACGCTGGCGGTGTGCCTAATACATGCAAGTTGAGCGGGGCTCCAGCAATGGAGCCTAGCAGCGGACGGGTGAGTAACACGTAGGTAACCTGCCTTTAAGCCTGGAATACCCGAGAGAAATCTCGGCTAATGCCGGATATGTGATTGAGAGGCATCTCTCAGTCATGAAAGGGGCAATTGCTCCTACTTTAGATGGACCTGCGGCGCATTAGTTAGTTGGTGAGATAACAGCCCACAAGACGAGGATGCGTAGCCGACCTGAGA
>FQH3XDB01EW357|309|1
GATGAACGCTGGCGGTGTGCCTAATACATGCAAGTTGAGCGGGGCTCCAGCAATGGAGCCTAGCAGCGGACGGGTGAGTAACACGTAGGTAACCTGCCTTTAAGCCTGGAATACCCCGAGAGAAATCTCGGCTAATGCCGGATATGTGATTGAGAGGCATCTCTCAGTCATGAAAGGGGCAATTGCTCCATTTTAGATGGACCTGCGGCGCATTAGTTAGTTGGTGAGATAACAGCCCACCAAGCGAGGATGCGTAGCCGACCTGAGAGGGT
>FQH3XDB01CTBOO|310|19
GATGAACGCTGGCGGTGTGCCTAATACATGCAAGTTGAGCGGGGCTCCAGCAATGGAGCCTAGCAGCGGACGGGTGAGTAACACGTAGGTAACCTGCCTTTAAGCCTGGAGTACCCGAGAGAAATCTCGGCTAATGCCGGATATGTGATTGAGAGGCATCTCTCAGTCATGAAAGGGGCAATTGCTCCACTTTTAGATGGACCTGCGGCGCATTAGTTAGTTGGTGAGATAACAGCCCACAAGACGAGGATGCGTAGCCGACCTGAGAGGGTGATCGGCCACAATGGAACTGAGACACGGTCCATACTCC
>FQH3XDB01ALTSQ|311|2
GATGAACGCTGGCGGTGTGCCTAATACATGCAAGTTGAGCGGGGCTTCAGCAATGAAGCCTAGCAGCGGACGGGTGAGTAACACGTAGGTAACCTGCCTTTAAGCCTGGAATACCCGAGAGAAATCTCGGCTAATGCCGGATATGTGATTGAGAGGCATCTCTCAGTCATGAAAGGGGCAATTGCTCCATTTTAGATGGACCTGCGGCGCATTAGTTAGTTGGTGAGATAACAGCCCACCAAGACGAGGATGCGTAGCCGACCTGAGAGGGTGATCGGCCACAATGGAACTGAGACACGGTCCATACTCCTACGGGAGGCAGCAGTAGGGAGTTTTCGGCAA
>FQH3XDB01A2TO3|312|21
GATGAACGCTGGCGGTGTGCCTAATACATGCAAGTTGAGCGGGGCTTCAGCAATGAAGCCTAGCAGCGGACGGGTGAGTAACACGTAGGTAACCTGCCTTTAAGCCTGGAATACCCGAGAGAAATCTCGGCTAATGCCGGATATGTGATTGAGAGGCATCTCTCAGTCATGAAAGGGGCAATTGCTCCACTTTTAGATGGACCTGCGGCGCATTAGTTAGTTGGTGAGATAACAGCCCACAAGACGAGGATGCGTAGCCGACCTGAGAGGGTGATCGGCCACATGGAACTGAGACACGGTCCATA
>FQH3XDB01DCULU|313|1
GATGAACGCTGGCGGCATGCCTAAGACATGCAAGTCGAACGGGATGGCCCAATGATTACTCGGAAAGTTTTTGTGCTTGCACGAGAACCGGAAGAGTAAGATTTGGATTTTCCATCCAGTGGCAAACGGGTGAGTAACACGTGGGTTACCTACCTCTAAGACGGGGACAACAGTTGGAAACGATTGCTAATACCGGATGTGCTCTACGGAGTAAAGAAGCCCTTAAAGCTTCGCTTAGAGATGGGCCTGCGGTGCATTAGCTAGTTGGTGGGTAAAGCCTACCAAGGCGACGATGCATA
>FQH3XDB01A6VF1|314|2
GATGAACGCTGGCGGCATGCCTAAGACATGCAAGTCGAACGGGATGGCCCAATGATTACTTGGAACTTTCTTGTGCTTGCACAAGGAAGGGAAAAGTAAGATTTGGATTTTCCATCCAGTGGCAAACGGGTGAGTAACACGTGGGTTACCTACCTCTAAGACGGGGACAACAGTTGGAAACGATTGCTAATACCGGATGTGCTCTACGGAGTAAAGGAGCCTTAAAGCTTCGCTTAGAGATGGGCCTGCGGTGCATTAGCTAGTTGGTGGGGTAATGGCCTACCAAGGCGACGATGCATAGCCGAACTGAGAGGTTAATCGGCCACACTGGGACTGAGACACGG
>FQH3XDB01DV152|315|8
GATGAACGCTGGCGGCATGCCTAAGACATGCAAGTCGTACGAAGCGGCCCAATGATTTATTTAGAAGCACTGAGAGCTTGCTCAAAGTGTGGACAAATAATGATTTGGATTTTCCGCTTAGTGGCAAACGGGTGAGTAACACGTGGGTTACCTACCTCCTAGATGGGGATACCAATTGGAAACGATTGTTAATACCGAATGTGATCTATGGATTAAAGAAGCCTTTAAAGCTTCGCTAGGAGATGGGCCTGCGGTGCATTAGATAGTTGGTGGGGTAATGGCCTACCAAGTCGACGATGCATAGCTGAACTGAGAGGTTGATCGGCCACATTGGGACTGAGA
>FQH3XDB01BP9EV|316|1
GATGAACGCTGGCGGCATGCCTAAGACATGCAAGTCGTACGAGATGGCCCATTGAGATTGATTGAAAATGGAGTGCTTGCACAAAATTGGATTTCTTTGATTTGGATTTTCCATCTAGTGGCAAAACGGGTGAATAACACGTGGGTTACCTACCTCTTTGTTGGGGATAACAATTGGAAACGATTGATAATACCGAATGTGCTCTACGGAGTAAAGAAGCTCTAAAGCTTCGCTAAGAGATGGGCCTGCGGCGTATTAGCTAGTTGGTGGGTATGGCCTACAGGCAACGATGCGTAGCC
>FQH3XDB01CCIUF|317|92
GACGAACGCTGGCGGCGCGCCTAACACATGCAAGTCGAACGAGCGATGAGGAGCTTGCTTCTCAAAGCGAGTGGCGAACGGGTGAGTAACGCGTGAGGAACCTGCCTCAAAGAGGGGGACAACAGTTGGAAACGACTGCTAATACCGCATAAGCCCACGGCTCGGCATCGAGCAGAGGGAAAAGGAGCAATCCGCTTTGAGATGGCCTCGCGTCCGATTAGCTAGTTGGTGAGGTAACGGCCACCAAGGCGACGATCGGTAGCCGGACTGAGAGGTTGAACGGCCACATTGGGACTGAGACACGGCCCAGACTCCTACGGGAGGCAGCAGTGGGAATATTGCACATGGGGAAACCCTGATGCAGCGACGCGCGTGGAGGAAGAAGGTC
>FQH3XDB01BKZ47|318|1
AACGAACGCTGGCGGCGCGTCTTAAGCATGCAAGTCGGGCGGGATCCATGCGCTTGCGCATGGTGAGAGCGGCGGACTGGCGAGTAACACGTGAGGAACCTGCCTCAAAGAGGGGGACAACAGTTGGAAACGCTGCTAATACCGCATAAGCCCAGACCTGGCATCGGGTTGAGGGAAAAGAGCAATCCGCTTTGAGATGGCCTCGCGTCCGATTAGCTGGTTGGTGAGGTAACGGCCCACCAAGGCGACGATCGGTAGCCCGGATGAGAGGTTGAACGGCCACATTGGGACTGAGACACGGCCCCAGACTTCCACGGGA
>FQH3XDB01EB40K|319|1
AACGAACGCTGGTGGAGTGTCTTATACATGCAAGTCGAGCGAGGACGTAGCGATACGAGCCGAGCGGCGAATGGGTGAGTAACGCGTAAGCAACCTGCCCCGCACACCGGAACAACCGTGCCAACGCGCGGCTAATGCCGGGAGCCGTGGTTCCCCGCATGGGGGATTGACGAAAGATTTATCGGTGCGGGATGGGCTTGCGTCCGATTAGCTAGTTGGTGAGGTAACGGCCCACCAAGGCGACGATCGGTAGCCGGACTGAGAGGTTGAACGGCCACATTGGGACTGAGACACGGCCCAGACTCCTACGGGAGGCAGCAGTGGGGAATATTGCACAATGGGGGAAACCCTGATGCAGCGACGCCGCGTGGAGGAAGAAGGTCTTCGGATTGTAAACTCCTGTTGTTGGGAAGATAATGACGGTACCAAC
>FQH3XDB01BP236|320|5
GACGAACGCTGGCGGCGCGCCTAACACATGCAAGTCGAACGGAGTAAATTTTCTCACTGAGTCTTTTGGGCGGTTAGCCGGGAGCGCTAACGATGCGCAAGCATCGTTCAGGCGATTCCAGTAAGCCTAACGGTGCTCGGAAGACCGAGTGAGGAAATTTACTTAGTGGCGAACGGGTGAGTAACGCGTGAGGAACCTGCCTCAAAGAGGGGGACAACAGTTGGAAACGACTGCTAATACCGCATAAGCCCACGGTCTCGCATGAGACAGAGGGAAAAGGATTTTATCCGCTTTTGAGATGGCCTCGCGTCCGATTAGCTAGTTGGTGAGGTAACGGCCCACCAAGGCGACGATCGGTAGCCGGACTGGAGAGGTTGAACGGCCACATTGGGACTGAGAC
>FQH3XDB01C4FHN|321|1
GACGAACGCTGGCGGCGCGCCTAACACATGCAAGTCGAACGGAGTAAATTTTCTCACTGAGTCTTTTGGGCGGTTAGCCGGGAGCGCTAACGATGCGCAAGCATCGTTCAGGCGATTCCAGTAAGCCTAACGGTGCTCGGAAGACCGAGTGAGGAAATTTACTTAGTGGCGAACGGGTGAGTAACGCGTGAGGAACCTGCCTCAAAGAGGGGGACAACAGTTGGAAACGACTGCTAATACCGCATAAGCCCACAGGCCGGCATCGGCCAGAGGGAAAAGGATTTATCCGCTTTGAGATGGCCTCGCGTCCGATTAGCTAGTTGGTGAGGTAACGGCCCACCAAGGCGACGATCGGTAGCCGGACTGAGAGGTTGAACGGCCACA
>FQH3XDB01AUPGU|322|3
AATGAACGTTGGCGGCGTGGATTAGGCATGCAAGTCGAGCGAGAACCCAGCAATGGGGGACAGCGGCGAAAGGGACAGTAATGTGTAGATCATCTGCCCTCAGGCCCGGGATAGCTGCGGGAAACTGCAGGTAATACCGGATAATATCTCCGGATCAAAGGTGTGGTTCCGCCTGGGGATGAGTCTACATCCTATTAGCTAGTTGGTGGGGTAATGGCCTACCAAGGCGATGATGGGTAGCGGGTGTGAGAGCACGACCCGCGTCATTGGGACTGAGACACTGCCCCAGACACCTACGGGTGCTGCAGTCGAGAATCTTCGGCAATGGGCGCAAGCCTGACCGAGCGACGCCGCGTGCGGGATGAAGGCCTTCGGG
>FQH3XDB01DSXLD|323|6
AATGAACGTTGGCGGCGTGGATTAGGCATGCAAGTCGAGCGAACGAGGGAGCTTGCTCCCGAGTTAGCGGCGAAAAGGGGATAGTAACGCGTAGTTACCAACCCTCAGGCCGGGGATAGCTTTTGGAAACGAAAGGTAATACCCGATAACATCTCCGGATCAAAGGTGAGATTCCGCCTGAGGACGGGACTGCGTCCTATTAGCTTGTGGTGAGGTAATGGCTCACCAAGGCGACGATGGGTAGCGGGCGTGAGAGCGTGACCCGCATCATTGGGACTGAGACACTGCCCAAACACCTACGGGTGGCTGCAGTCGAGAATCTTCGGCAATGGACGAAAGTCTGACCGAGCGACGCCGCGTGCGGGATGACGGTCTTCGGATTGTAAACCGCTGTCAAGTTGGAGGAAGTGACAGAGAGCCAATCTCTCTGTTTTGACCGAGA
>FQH3XDB01E19ZJ|324|17
AATGAACGTTGGCGGCGTGGATTAGGCATGCAAGTCGTGCGGCAAGGAAGACTTCGGTCTTCCCTAGAGCGGCGAAAGGGATAGGAACACATAGATACATGCCTCTTAGCCCGGGATAGCGTTTGGAAACGAACGATAATACCGGACGCCCTCTCCGGAGGAAAGGTGAGATTCCGCTAAGAGATTGGTCTATGTCCTATTAGCTAGTTGGTAAGGTAACGGCTTACCAAGGCGATGATGGTACGGGGTGTGAGAGCATGACCCCGATCACTGGGACTGAGACACTGCCCAGACGCCTACGGGCGGCTGCAGTCGAGAATCTTCGGCAATGGACGAAAGTCTGACCGAGCGACGCCGCGTGTATGACGAAGGTCTTCGGATTGTAAAGTACTGTCGATTAGG
>FQH3XDB01B32R8|325|1
AATGAACGTTGGCGGCGTGGATTAGGCATGCAAGTCATGCGGCAAGGAAGACTTCGGCCTTCCCTAGAGCGGCGAAAGGGATAGGAACACATAGATACATGCCTCTTAGCCCGGGATAGCGTTTGGAAACGAACGATAATACCGGATGCCCTCTCCGGAGGAAAGGTGAGATTCCGCTAAGAGATTGGTCTATGTCCTATTAGCTAGTTGGTAAGGTAACGGCTTACCAAGGCGATGATGGGTACGGGGTGTGAGAGCATGACCCCGATCACTGGGACTGAGACACTGCCCCAGACGCCTACGGGCGGCTGCAGTCGAGAATCTTCGGCAATGGACGAAAGTCTGACCGAGCGACGCCGCGTGTATGACGAAGGTCTTCGGATTGTAAAGTACTGTCGATTAGGCGGAA
>FQH3XDB01EMHYS|326|1
GACAAACGCTGGCGGCGTGCTTTAAACATGCAAGTCGAACGGAGGTTAGGTTTTCCACTAAGCATTCTTAATGGCTCCCATGAAACGAAAGTTGAAATGGGAAAAACCGAGCACCGACTAAAAAGAAATTTTGCAACGCAAAATGATTGAAAAAGTCGAGTGTGAGGGTGTTTAGTGGAGAACTTACCCTTAGTGGCGGACGGGTGAGTAACGCGTGAGTAATCTACCTCTTTCTGGGGGACAACAGTTGGAAACGACTGCTAATACCGCATAGGACCACAGGTACACAAGTACTAGGGGTGAAAGATTTATCGGATAGAGATGAGCTCGCGTAACATTAGGTTGTTGGTGAGGTAACGGCTCACCAAGCCGACGATGTTTAGCCGATCTGAGAGGATGATCGGCCACACTGGAACTGA
>FQH3XDB01AGBFC|327|1
GACGAACGCTGGCGGCGTGCTTCAAACATGCAAGTCGAACGAGTAAGTAGTTAGCACAAAAATTTCTAGGCAGCGTCTAAAGATTGTGAAACAATCTTTAAGGCAAACCAAAGTGCTCAACCCTAAAATAGAAAGAAGGCAATAGGGGGCAGAGAATAGAAATTTTGTGCTAACGACTTGCTAGTGGCGGACGGGTGAGTAACGCGTGAGCAATCTGGCTATTACAGGGGGATAACAGTTGGAAACGACTGCTAATACCGCATATGACCACAAGCCGACATCGGCAAGGGGGTGAAAAGGAGCAAATCCGGTAATAGATGAGCTTGCGTTTCATCAGCTAGTTGGTGAGGGTAACGGCCCACCAAGGCGACGACGAATAGCCGATCTGAGAGGATGACCGGCCACACTGGTATTGAGAACGGACCAGACTCCGTACGGGAGGCAGCAGTTAGGAATAGTTGGGCAAAGTGGAGGGAACTCTGACCCAGCAACG
>FQH3XDB01AL14F|328|1
GATGAACGCTGGCGGCATGCCTAAGACGTGCAAGTCGAACGAAGTGGCCCATTGAAGATTGAGTGCTTGCACAAGATTGGATTTGGATTCCCACTTAGTGGCAGACGGGTGAGTAACGCGTGGGTAATCTACCTCAGAGACTGGGACAACTATTGGAAACGATAGCTAATACCGGATGATTCATTTTTACATAAGTAGAAATGCTAAAAGGAGCTTCGGCTTCACTTTGAGATGAGCTTGCGGTGTATTAGCTAGTTGGTGGGGTAATGGCCTACCAAGGCAACGATGCATATCCGAGCTGAGAGGCTGATCGGACACATTGGAACTGAGAC
>FQH3XDB01AW75Q|329|79
AACGAACGCTGGCGGCGCGTCTTAAGCATGCAAGTCGGACGGCAAGAGAGGGCTTGCCCTCTCCTAGAGTGGCGGACTGGTGAGTAACGCGTGGGCGACGCACCCTTCCGGCGGGGACAGCTCCTGGAAACAGGAGGTAATACCCGGTACGTTCCGTGATGTCAGAGGTCACGGAGGAAAGGCTCCTTTGAGCCGCGGAAGGCGCGGCCCGCGTGCCATTAGCCTGCTGGTGAGGTAACGGCCCACCAGGGCGATGATGGCTACCCGGCCTGAGAGGGCGGACGGGCACATTGGGACTGAGATACGGCCCAGACTCCGTACGGGAGGCAGCAGCTAAGAATATTCCGCAA
>FQH3XDB01CAR5A|330|8
AACGAACGCTGGCGGCAGGTCTTAGGCATGCAAGTCGAACGGGTGAAGCAGGGCTCGCCCTGTGGAGCTAGTGGCGCACGAGTGAGTAACGCGTGGGAAACTGCCCATCACTGGGGAATAACGTTTGGAAACGAACGCTAATACCGCATACGCCGGAAACGGGAAAGATTTATCGGTGATGGATGTGCCCGCGTTGGATTAGCTTGTTGGTGGGGTAACGCCTACCAAGGCGATGATCCATAGCTGGTCTGAGAGGATGATCAGCCACGCTGGAACTGAGACACGGTCCAGACTCCTACGGGAGGCAGCAGCTAAGATATTGGGGCAATGGAGGAAACTCTGACCCCAGCCATGCCGCGTGAATGGAAGAAGGCCTTCGGGTTGTAAGTTCTTTAATCGTGAGATGATGACAGTAGCGAATAGAAAAAGCACGGCTAATCGTGCCAGCAG
>FQH3XDB01BHE8J|331|1
AACGAACGCTGGCGGCAGGTCTTAGGCATGCAAGTCGAACGGGTGAAGCAGGGCTTGCCCTGTGGAGCTAGTGGCGCACGAGTGAGTAACGCGTGGGAAACTGCCCATCACTGGGGAATAACGTTTGGAAACGAGCTAATACCGCATACGCCGGAACGGAAGATTATCGGTGATGGATGTGCCCGCGTTGGATTAGCTTGTTGGTGGGTAACGGCCTACCAAGGCGGATGATCCATAGCTGGTCTGAGAGGATGATCAGCCACGCTGGAACTGAGACACGGTTCCAGACTCCTACGGGAGGCAGCAGCTAAGAATATTGGGC
>FQH3XDB01AHXR3|332|1
AACGAACGCTGGCGGCAGGTCTTAGGCATGGCAAGTCGAACGGGTGAAGCAGGGGCTCGCCCTGTGGAGCTAGTGGCGCACGAGTGAGTAACGCGTGGGAAACTGCCCATCACTGGGGAATAACGTTTGGAAACGAACGCTAATACCGCATACGCCGGAAAACGGGAAAGATTTTATCGGTGATGGATGTGCCCGCGTTGGATTAGCTTGTTGGTGGGGTAACGGCCTACCAAGGCGATGATCCATAGCTGGTCTGAGAGGATGATCAGCCACGCTGGAACTGAGACACGGTCCAGACTCCTACGGGAGGCAGCAGCTAAGAATATTGGGGCAATGGAGGAAACTCTGACCCAGCCAT
>FQH3XDB01DBSNY|333|1
AACGAACGCTGGTGGAGTGTCTTATACATGCAAGTCGAGGGGAAACGGCAAAGTTAGCTTGCTAACTTTGGATGTCGACCGGCGCACGGGTGAGTAACGCGTATCCAACCTGCCCACCACTTGGGGATAACCTTGCGAAAGTAAGACTAATACCCAATGACATTGAGTGCAGACATCTAATCTCAATTAAAGATTCATCGGTGATGGATGGGGATGCATCCGATTAGCTTGTTGGCGGGTAACGGCCCACCAAGGCTACGATCGGTAGGGTTCTGAGAGGAACGGTCCCCCACATTGGAACTGAGACACGGTCC
>FQH3XDB01D882L|334|27
AACGAACGCTGGCGGCGCGTCTTAAGCATGCAAGTCGGACGGCAAGAGAGAGCTTGCTCTCTCCTAGAGTGGCGGACTGGTGAGGAACGCGTGGGTGACGCACCCTCCTGACGGGGACAGCTCCTAGAAATAGGAGATAATACCGGATACGCTGCATGTAAGTAGAGGACATGCAGGAAAGGAGCCTTTGCTCCGCAGGGGAACGCCCGCGTGCTATTAGCTGGACGGCGGGGTAACGGCCACCGTGGCGACGATAGCTACCCGGCCTAAGAGGGCAAACGGGCACATTGGGACTGAGATACGGCCCAGACTCCTACGGGAGGCAGCAGCTAAGATATTCCGCAATGGGGGAAACCCTGACGGAGCGACGCCGCGTGGACGATGAAGGCCGGAAGGTTGTAAAGTCCTTTA
>FQH3XDB01AFCI4|335|17
AACGAACGCTGGCGGCGCGTCTTAAGCATGCAAGTCGAGCGGCAAGATTGGGGCTTGCTCCAATCCCAGAGCGGCGGACTGGTGAGTAACACGTAGGCGACGTGCCCTTGGGACGGGGATAGCCTGTGGAAACACAGGGTAATACCGGATAAGGCCGTATGCGTTGGAGGCATACGGGGAAAGGAGCTATGGCTTCGCACAAGGAGCGACCTGCGGCCCATCAGCTAGTTGGCGGGTAAGGCCCACAAGGCAATGACGGGTATCCGGCCTGAGAGGGTGAACGGACACATTGGGACTGAGATACGGCCCAGACCTCCTACGGGAGGCAGCAGCTAAGATATTCCGCAATGCGCGAAGCGTGACGGAGCGACGCCGCGGTGGACGACGGAGGCCGGAAGG
>FQH3XDB01DSOJU|336|2
AACGAACGCTGGCAGCGTGGATTAGGCATGCAAGTCGAACGGGATCGGACGGGTAGCAATATTCGTCCGTGAGAGTGGCGGAAGGGCGAGGAACACGTGGGCAACCTGTCCTTGAGGTGGGGAAAACCGCTGGAAACGGCGGCTAATACCGCATAAGCGCACGAGATCGCATGATTTCGTGTGAAAAACTCCGGTGGTACAAGATGAACCCGCGTCTGATTAGCTAGTTGGTGAGGTAACGGCCCACCAAGGCGACGACCAGTAGCCGGCCTGAGAGGGTGAACGGCCACATTGGACTGAGACACGGCCCAAAACTCCTACGGGAGGCAGCGAGGTGGGGAATATTGC
>FQH3XDB01C6YGE|337|2
AACGAACGCTGGCAGCGTGGATTAGGCATGCAAGTCGAACGGGATCCGATGAATGATTAGCAATATGATTTTATTGGTGAGAGTGGCGGAAGGGCGAGGACAATATGGGTAATCTGCCTTTATGCTGGGGATAACCTCTCGAAAGAGAAGCTAAAACCGAATGTGGTGCGCGGGGACATCTTCGCGTAACGTAAAGGGGGGCTCGCAAGACCTCCTGCATAGAGATGAGCCCGTAGATCATCAGCTTGTTGGTGGGTAACGGCCTACCAAGGCGATGACGATTAGCTGGTCTGAGAGG
>FQH3XDB01E29L0|338|1
AACGAACGCTGGCAGCGTGGATTAGGCATGCAAGTCGAACGGGATCTTGCGGATGAGTAGCAATATGATTTCGCAGGTGAGAGTGGCGGAAGGGCGAGGACAATATGGGTAATCTGCCTTTATGCTGGGGATAACCTCTCGAAAGAGAAGCTAAAACCGAATGTGGTGCGTGGAGACATCTTCGCGTGACGTAAAGGGGGGATCGCAAGACCTCCTGCATAGAGATGAGCCCGTAGATCATCAGCTTGGTTGGTGGGGTAACCGGCCTACCAAGGCGATGACGATTAGCTGGTCTGAGAGGACGGCCAGCCGCACGTGGGACTGAGATACT
>FQH3XDB01BHQD6|339|2
AACGAACGCTGGCAGCGTGGATTAGGCATGCAAGTCGAACGGGATCCTGCGGAAGAGTAGCAATATGATTTCGCGGGTGAGAGTGGCGGAAGGGCGAGGACAATATGGGTAATCTGCCTTTATGCTGGGGATAACCTCTCGAAAGAGAAGCTAAAACCGAATGTGGTGCGCGGAGACATCTTCGCGTGACGTAAAGGGGGGATCGCAAGACCTCCTGCATAGAGATGAGCCCGTAGATCATCAGCTTGTTGGTGGGGTAACGGCCTACCAAGGCGATGACGATTAGCTGGTCTGAGAGGACGGCCAGCCGCACGTGGGA
>FQH3XDB01DG4NQ|340|3
AACGAACGCTGGCAGCGTGGATTAGGCATGCAAGTCGAACGGGATCCTGCGGAAGAGTAGCAATATGATTTCGCGGGTGAGAGTGGCGGAAGGGCGAGGACAATATGGGTAATCTGCCTTTATGCTGGGGATAACCTCTCGAAAGAGAAGCTAAAACCGAATGTGGTGCGCGGAGACATCTTCGCGTGACTAAAGGGGGGATCGCAAGACCTCCTGCATAGGGATGAGCCCGTAGATCATCAGCTTGTTGGTGGGTAACGGCCTACCAAGGCGATGACGATTAGCTGGTCTGAGAGGACGGCCAGCCGCACTGGGACTGAGATACTG
>FQH3XDB01BNQB7|341|8
AACGAACGCTGGCAGCGTGGATTAGGCATGCAAGTCGAACGGGATCCTGCGGATGAGTAGCAATATGATTTCGCAGGTGAGAGTGGCGGAAGGGCGAGGACAATATGGGTAATCTGCCTTTATGCTGGGGATAACCTCTCGAAAGAGAAGCTAAAACCGAATGTGGTGCGCGGGGACATCTTCGCGTGACTAAAGGGGGGATCGCAAGACCTCCTGCATAGAGATGAGCCCGTAGATCATCAGCTTGTTGGTGGGGTAACGGCCTACCAAGGCGATGACGATTAGCTGGTCTGAGAGGACGGCCAGCCGCACGTGGGACTGAGATACTG
>FQH3XDB01CGJL3|342|1
AACGAACGCTGGCAGCGTGGATTAGGCATGCAAGTCGAACGGGATCCTGCGGATGAGTAGCAATATGATTTCGCGGGTGAGAGTGGCGGAAGGGCGAGGACAATATGGGTAATCTGCCTTTATGCTGGGGATAACCTCTCGAAAGAGAAGCTAAAACCGAATGTGGTGCGCGGGGACATCTTCGCGTGACGTAAAGGGGGGATCGCAAGACCTCCTGCATAGAGATGAGCCCGTAGATCATCAGCTTGTTGGTGGGGTAACGCCTACCAAGGCGATGACGATTAGCTGGTCT
>FQH3XDB01D0D8U|343|3
AACGAACGCTGGCAGCGTGGATTAGGCATGCAAGTCGAACGGGATCCGGAAGGCAGCAATGTCGGCCGGTGAGAGTGGCGGAAGGGCGAGGAACACGTGGGCAACCTGTCCTCGAGGTGGGGAAAACCGCTGGAAACGGCGGCTAATACCGAATGCGGCGCGTCCGGGGCATCCCGGGCGCGCCAAAGGGGGCCGCAAGGCTCCCCTCGTCGAGGGGCTCGCGCACCATCAGCTTGTTGGCGGGTAACGGCCACCAAGGCTCACGGTTAGCTGGTCTGAGAGGATGGCCAGCCGCACTGGGACTGAGATACTGCCCCAGGACCTCCGTACGGGAGGCTGCAGTCGAGGATCATTCGCAAGTGGGGAAACCCTGACGATGCGACGCTGCGTGGA
>FQH3XDB01BIOJR|344|13
AACGAACGCTGGCAGCGTGGATTAGGCATGCAAGTCGAACGGGATCGGACGGGTAGCAATATTCGTCCGTGAGAGTGGCGGAAGGGCGAGGAACACGTGGGCAACCTGTCCTTGAGGTGGGGAAAACCGCTGGAAACGGCGGCTAATACCGAATGCGGCGCGGCCGGGGCATCCCGGACGCGCCAAAGGGGGCCGCAAGGCTCTCCCTCATCGAGGGGCTCGCGCACCATCAGCTTGTTGGCGGGTAACGGCCACCAAGGCTTACGGTTAGCTGGTCTGAGAGGATGGCCAGCCGCACTGGGACTGAGATACTGCCCCAGACTCCTACGGGAGGCTGCAGTCGAGGATCATTCGCAATGGGGGAAACCCTGACGATGCGACGCTGCGTGGAGGATGAAGGCCCTCGGGTCGTAAACTCCTGTCATGACGGAAAGGAGAGGGACCCAACACGTCCCTTATTGATGGTACGTCAAGAGGAAGAGACGGCTAACTCTGT
>FQH3XDB01BRXM8|345|1
AACGAACGCTGGCAGCGTGGATTAGGCATGCAAGTCGAACGGGATCTTGCGGATGAGTAGCAATATGATTTCGCAGGTGAGAGTGGCGGAAGGGCGAGGACAATATGGGTAATCTGCCTTTATGCTGGGGATAACCTCTCGAAAAGAGAAGCTAAAACCGAATGTGGTGCGTGGAGACATCTTCGCGTGACTAAAGGGGGGATCGCAAGACCTCCTGCATAGAGATGAGCCCGTAGATCATCAGCTTGTGGTGGGTAACGGCCTACAAGGCGATGACGATTAGCTGGTCTGAGAGGACGGCCAGCCGCACTGGGACTGAGATACTGCCCAGACCTCCGTACGGGAGGCTGCAGTCGAGGATC
>FQH3XDB01A2T74|346|2
AACGAACGCTGGCAGCGTGGATTAGGCATGCAAGTCGAACGGGATCTTGCGGATGAGTAGCAATATGATTTCGCAGGTGAGAGTGGCGGAAGGGCGAGGACAATATGGGTAATCTGCCTTTATGCTGGGGATAACCTCTCGAAAGAGAAGCTAAAACCGAATGTGGTGCGTGGAGACATCTTCGCGTGACGTAAAGGGGGGATCGCAAGACCTCCTGCATAGAGATGAGCCCGTAGATCATCAGCTTGTTGGTGGGTAACGGCCTACCGAAGGCGATGACGATTAGCTGGTCTGAGAGGACGGCCAGCCGCACTGGGACTGAGATACTGCCCC
>FQH3XDB01DNGG6|347|7
AACGAACGCTGGCAGCGTGGATTAGGCATGCAAGTCGAACGGGATCTTGCGGATGAGTAGCAATATGATTTCGCAGGTGAGAGTGGCGGAAGGGCGAGGACAATATGGGTAATCTGCCTTTATGCTGGGGATAACCTCTCGAAAGAGAAGCTAAAACCGAATGTGGTGCGTGGAGACATCTTCGCGTGACTAAAGGGGGGATCGCAAGACCTCCTGCATAGAGATGAGCCCGTAGATCATCAGCTTGTTGGTGGGTAACGGCCTACCAAGGCGATGACGATTAGCTGGTCTGAGAGGACGGCCAGCCGCACTGGGACTGAGATACTGCCCAGACTCCTACGGGAGGCTGCAGTCGAGGATCATTCGCAAGTGGACGGAAGTCTG
>FQH3XDB01DXUZ3|348|1
AACGAACGCTGGCAGCGTGGATTAGGCATGCAAGTCGAACGGGATCCGATGAATGATTAGCAATATGATTTTATTGGTGAGAGTGGCGGAAGGGCGAGGACAATATGGGTAATCTGCCTTTATGCTGGGGATAACCTCTCGAAAAGAGAAGCTAAAACCGAATGTGGTGCGCGGGGACATCTTCGCGTAACTAAAGGGGGGCTCGCAAGACCTCCTGCATAGAGATGAGCCCGTAGATCATCAGCTTGTGGTGGGGTAACGGCCTACCAAGGCGATGACGATTAGCTGGTCTGAGAGGACGGCCAGCCGCACTGGGACTGAGATACTGCCCAGACTCCGTACGGGAGGCTGCAGTCGAGGATCATTCGCAATGGACGGAA
>FQH3XDB01BPUR8|349|23
AACGAACGCTGGCAGCGTGGATTAGGCATGCGAGTCGAACGGGATCCGATGAATGATTAGCAATATGATTTTATTGGTGAGAGTGGCGGAAGGGCGAGGACAATATGGGTAATCTGCCTTTATGCTGGGGATAACCTCTCGAAAGAGAAGCTAAAACCGAATGTGGTGCGCGGGGACATCTTCGCGTAACTAAAGGGGGGCTCGCAAGACCTCCTGCATAGAGATGAGCCCGTAGATCATCAGCTTGTTGGTGGGTAACGGCCTACCAAGGCGATGACGATTAGCTGGTCTGAGAGGACGGCCAGCCGCACTGGGACTGAGACTACTGGCCCCAGTA
>FQH3XDB01DUXWP|350|22
AACGAACGCTGGCAGCGTGGATTAGGCATGCAAGTCGAACGGGATCCTGCGGATGAGTAGCAATATGATTTCGCAGGTGAGAGTGGCGGAAGGGCGAGGACAATATGGGTAATCTGCCTTTATGCTGGGGATAACCTCTCGAAAGAGAAGCTAAAACCGAATGTGGTGCGCGGGGACATCTTCGCGTGACTAAAGGGGGGATCGCAAGACCTCCTGCATAGAGATGAGCCCGTAGATCATCAGCTTGTTGGTGGGGTAACGGCCTACCAAGGCGATGACGATTAGCTGGTCTGAGAGGACGGCCAGCCGCACTGGGACTGAGATACTGCCCAGACCTCCGTACGGGAGGCTGCAGTCGAGGATCATTCGC
>FQH3XDB01A0JEM|351|3
AACGAACGCTGGCAGCGTGGATTAGGCATGCAAGTCGAACGGGGATCCTGCGGATGAGTAGCAATATGATTTCGCGGGTGAGAGTGGCGGAAGGGCGAGGACAATATGGGGTAATCTGCCTTTATGCTGGGGATAACCTCTCGAAAAGAGAAGCTAAAACCGAATGTGGTGCGCGGGGACATCTTCGCGTGACTAAAGGGGGGATCGCAAGACCTCCTGCATAGAGATGAGCCCGTAGATCATCAGCTTGTTGGTGGGGTAACGGCCTACCAAGGCGATGACGATTAGCTGGTCTGAGAGGACGGCCAGCCGCACTGGGACTGAGATACTGCCCAGACTCCGTACGGAGGCTGCAGTCGAGGATCATTCGCAATGGACGAAGTCTGACGA
>FQH3XDB01B06QZ|352|2
AACGAACGCTGGCAGCGTGGATTAGGCATGCAAGTCGAACGGGATCCTGCGGATGAGTAGCAATATGATTTCGCAGGTGAGAGTGGCGGAAGGGCGAGGACAATATGGGTAATCTGCCTTTATGCTGGGGATAACCTCTCGAAAGAGAAGCTAAAACCGAATGTGGTGCGCGGGGACATCTTCGCGTGACGTAAAGGGGGATCGCAAGACCTCCTGCATAGAGATGAGCCCGTAGATCATCAGCTTGTGGTGGGTAACGCCTACAGGCGATGACGATTAGCTGGTCT
>FQH3XDB01CO2OL|353|2
AACGAACGCTGGCAGCGTGGATTAGGCATGCAAGTCGAACGGGATCCTGCGGATGAGTAGCAATATGATTTCGCGGGTGAGAGTGGCGGAAGGGCGAGGACAATATGGGTAATCTGCCTTTATGCTGGGGATAACCTCTCGAAAGAGAAGCTAAAACCGAATGTGGTGCGCGGGGACATCTTCGCGTGACTAAAGGGGGGATCGCAAGCCTCCTGCATAGAGATGAGCCCGTAGATCATCAGCTTGTTGGTGGGTAACGCCTACCAAGGCGATGACGATTAGCTGGTCTGAGAGGACGGCCAGCCGCACTGGGACTGG
>FQH3XDB01AHASP|354|7
AACGAACGCTGGCAGCGTGGATTAGGCATGCAAGTCGAACGGGATCCTGCGGATGAGTAGCAATATGATTTCGCGGGTGAGAGTGGCGGAAGGGCGAGGACAATATGGGTAATCTGCCTTTATGCTGGGGATAACCTCTCGAAAGAGAAGCTAAAACCGAATGTGGTGCGCGGGGACATCTTCGCGTGACGTAAAGGGGGGATCGCAAGACCTCCTGCATAGAGATGAGCCCGTAGATCATCAGCTTGTTGGTGGGGTAACGCCTACCAAGGCGATGACGATTAG
>FQH3XDB01A1U58|355|1
AACGAACGCTGGCAGCGTGGATTAGGCATGCAAGTCGAACGGGATCCTGCGGATGAGTAGCAATATGATTTTCGCAGGTGAGAGTGGCGGAAGGGCGAGGACAATATGGGTAATCTGCCTTTATGCTGGGGATAACCTCTCGAAAGAGAAGCTAAAACCGAATGTGGTGCGCGGGGACATCTTCGCGTGACGTAAAGGGGGGATCGCAAGACCTCCTGCATAGAGATGACGCCCGTAGATCATCAGTCTTGGTTGGTGGGTAACGCCTACCGAAGGCGATGACGATTGCT
>FQH3XDB01DGIL3|356|13
AACGAACGCTGGCAGCGTGGATTAGGCATGCAAGTCGAACGGGATCCTGCGGAAGAGTAGCAATATGATTTCGCGGGTGAGAGTGGCGGAAGGGCGAGGACAATATGGGTAATCTGCCTTTATGCTGGGGATAACCTCTCGAAAGAGAAGCTAAAACCGAATGTGGTGCGCGGAGACATCTTCGCGTGACTAAAGGGGGGATCGCAAGACCTCCTGCATAGAGATGAGCCCGTAGATCATCAGCTTGTTGGTGGGTAACGCCTACCAAGGCGATGACGATTAGCTGGTCCGAGAGGACGGCCAGCCGCACGTGGGACTGAGATACTGCCCAG
>FQH3XDB01D9YK7|357|2
AACGAACGCTGGCAGCGTGGATTAGGCATGCAAGTCGAACGGGATCCTGCGGAAGAGTAGCAATATGATTTCGCGGGTGAGAGTGGCGGAAGGGCGAGGACAGTATGGGTAATCTGCCTTTATGCTGGGGATAACCTCTCGAAAGAGAAGCTAAAACCGAATGTGGTGCGCGGAGACATCTTCGCGTGACTAAAGGGGGGATCGCAAGACCTCCTGCATAGAGATGAGCCCGTAGATCATCAGCTTGTTGGTGGGTAACGGCCTACCAAGGCGATGACGATTAGCTGGTCTGAGAGGACGGCCAGCCGCACGTGGGACTGAGATACTGCCCAGACCTCCGTACGGGAGGCTGCAGTCGAGGATCA
>FQH3XDB01CEB7G|358|1
AACGAACGCTGGCAGCGTGGATTAGGCATGCAAGTCGAACGGGATCCTGCGGAAGAGTAGCAATATGATTTCGCGGGTGAGAGTGGCGGAAGGGCGAGGACAATATGGGTAATCTGCCTTTATGCTGGGGATAACCTCTCGAAAGAGAAGCTAAAACCGAATGTGGTGCGCGGAGACATCTTCGCGTGACTAAAGGGGGGATCGCAAGACCTCCTGCATAGAGATGAGCCCCGTAGATCATCAGCTTGTTGGTGGGTAACGCCTACAAGGCGATGACGATTAGCTGGTCTGAGAGGACGGCCAGCCG
>FQH3XDB01CW2G0|359|18
AACGAACGCTGGCAGCGTGGATTAGGCATGCAAGTCGAACGGGATCTTGCGGATGAGTAGCAATATGATTTCGCAGGTGAGAGTGGCGGAAGGGCGAGGACAATATGGGTAATCTGCCTTTATGCTGGGGATAACCTCTCGAAAGAGAAGCTAAAACCGAATGTGGTGCGTGGAGACATCTTCGCGTGACTAAAGGGGGGATCGCAAGACCTCCTGCATAGAGATGAGCCCGTAGATCATCAGCTTGTTGGTGGGGTAACGCCTACCAAGGCGATGACGATTAGCTGGTCTGAGAGGACGGCCAGCCGCACTGGGACTGAGATACTGCCCAGACCTCCTACGGGAGGCTGCAGTCGAGGATCATTCGCAATGGACGGAAGTCTGACGATGCGACGCTGCG
>FQH3XDB01A882E|360|36
AACGAACGCTGGCAGCGTGGATTAGGCATGCAAGTCGAACGGGATCCTGCGGATGAGTAGCAATATGATTTCGCAGGTGAGAGTGGCGGAAGGGCGAGGACAATATGGGTAATCTGCCTTTATGCTGGGGATAACCTCTCGAAAGAGAAGCTAAAACCGAATGTGGTGCGCGGGGACATCTTCGCGTGACTAAAGGGGGGATCGCAAGACCTCCTGCATAGAGATGAGCCCGTAGATCATCAGCTTGTTGGTGGGTAACGGCCTACCAAGGCGATGACGATTAGCTGGTCTGAGAGGACGGCCAGCCGCACTGGGACTGAGATACTGCCCAGACTCCTACGGGAGGCTGCAGTCGAGGATCATTCGCAAGTGGACGGAAGTCTGACGATGCGACG
>FQH3XDB01BCNQQ|361|14
AACGAACGCTGGCAGCGTGGATTAGGCATGCAAGTCGAACGGGATCCGATGAATGATTAGCAATATGATTTTATTGGTGAGAGTGGCGGAAGGGCGAGGACAATATGGGTAATCTGCCTTTATGCTGGGGATAACCTCTCGAAAGAGAAGCTAAAACCGAATGTGGTGCGCGGGGACATCTTCGCGTAACTAAAGGGGGGCTCGCAAGACCTCCTGCATAGAGATGAGCCCGTAGATCATCAGCTTGTTGGTGGGTAACGCCTACCAAGGCGATGACGATTAGCTGGTCTGAGAGGACGGCCAGCCGCACTGGGACTGAGATACTGCCCCAGACCTCCTACGGGGAGGCTGCAGTCGAGGATCATTCGCAATGGACGGAAGTCTGACGATGCGACGCTGCGT
>FQH3XDB01BQOFE|362|8
AACGAACGCTGGCAGCGTGGATTAGGCATGCAAGTCGAACGGGATCCTGCGGAAGAGTAGCAATATGATTTCGCAGGTGAGAGTGGCGGAAGGGCGAGGACAATATGGGTAATCTGCCTTTATGCTGGGGATAACCTCTCGAAAGAGAAGCTAAAACCGAATGTGGTGCGCGGAGACATCTTCGCGTGACTAAAGGGGGGATCGCAAGACCTCCTGCATAGAGATGAGCCCGTAGATCATCAGCTTGTTGGTGGGGTAACGGCCTACCAAGGCGATGACGATTAGCTGGTCTGAGAGGACGGCCAGCCGCACTGGGACTGAGATACTGCCCCAGACCTCCGTACGGGAGGCTGCAGTCGAGGATCATTCGCAAGTGGACGGAAGTCTGACGATGCGACGCTGCGTG
>FQH3XDB01BRQ28|363|1
AACGAACGCTGGCAGCGTGGATTAGGCATGCAAGTCGAACGGGATCCTGCGGAAGAGTAGCAATATGATTTCGCGGGCGAGAGTGGCGGAAGGGCGAGGACAATATGGGTAATCTGCCTTTATGCTGGGGATAACCTCTCGAAAGAGAAGCTAAAACCGAATGTGGTGCGCGGAGACATCTTCGCGTGACTAAAGGGGGGATCGCAAGACCTCCTGCATAGAGATGAGCCCGTAGATCATCAGCTTGTTGGTGGGGTAACGGCCTACCAAGGCGATGACGATTAGCTGGTCTGAGAGGACGGCCAGCCGCACTGGGACTGAGATACTGCCCAGACTCCTACGGGAGGCTGCAGTCGAGGATCATTCGCAATGGACGGAAGTCTGACGATGCGACGCTGCGTGAA
>FQH3XDB01E2JJP|364|19
AACGAACGCTGGCAGCGTGGATTAGGCATGCAAGTCGGACGGGATCGCCCCGGCAGCAATGTTGGGGCGTGAGAGTGGCGGATTGGCGAGGAACACGTGGGCAACCTGCCCCGGAGTCGGGGAAAACCTCTGGAAACGGAGGCTAATACCGGATGTGGCGCGGAGGCGGCATCGCCGCCGCGCCAAAGGGGGCCGCAAGGCTTCCGCTCCAGGAGGGGCCCGCGCACCATCAGCTCGTTGGCGGGTAACGGCCACCAAGGCTCACGGTTAGCTGGTCTGAGAGGATGACCAGCCGCACTGGGACTGAGATACTG
>FQH3XDB01D87NZ|365|61
AACGAACGCTGGTAGCGTGGATTAGGCATGCAAGTCGGACGGGATCCGGCGTGTAGCAATACAGGCCGGTGAGAGTGGCGGAAGGGCGAGGAACACGTGGGCAACCTGTCACGGAGGTGGGGAAAACCGCTGGAAACGGCGGCTAATACCGAATGCGGCGCCCGTCGGCATCGACGGAGCGCCAAAGGGGGCGAAAGCTCCCCTCCCCGAGGGGCCCGCGCACCATCAGCCTGCTGGCGGGTAACGGCCCACCAGGGCTTACGGTTAGCTGGTCTGAGAGGATGGCCAGCCGCACTGGGACTGAGATACTGCCCCAGACTCCTACGGGAGGCTGCAGTCGAGGATCATTTGCAATGGGCAAAGCCTGACAATGCGACGCTGCGTGGAGGATGAAGGCCCTCGGGTTGTAAACTCCTGTCAGTAAGGAACAGGCGAGGGGACCCAACGACGTCCTCAGTTGAGTGTACCTTAAGAGGAAGCGACGGCTAA
>FQH3XDB01D298M|366|2
AACGAACGCTGGCAGCGTGGATTAGGCATGCAAGTCGGACGGGATCGCCCCGGCAGCAATGTTGGGGCGTGAGAGTGGCGGATTGGCGAGGAACACGTGGGCAACCTGCCCCGGAGTCGGGGAAAACCTCTGGAAAACGGAGGCTAATACCGGATGTGGCGCGGAGGCGGCATCGCCGCCGCGCCAAAGGGGGCCGCAAGGCTTCCGCTCCAGGGAGGGGCCCCGCGCACCATCAGCTCGTTGGCGGGTAACGCCACCAAGGCTCACGGTTAGCTGGTCTGAGAGGATGACCAGCCGCACTGGGACTGAGATACTGCCCCAGACCTCCGTACGGGAGGCTGCAGTCGAGGATCATTTGCAAGTGGGCGAAAGCCTGAC
>FQH3XDB01ARFY3|367|3
AACGAACGCTGGCAGCGTGGATTAGGCATGCAAGTCGGACGGGATCGGACGGGCAGCAATGTTCGTCCGTGAGAGTGGCGGATTGGCGAGGAACACGTGGGCAACCTGCCCCGGAGTCGGGGAAAACCTCTGGAAACGGAGGCTAATACCGGATGTGGCGCGGAGGCGGCATCGCCGCCGCGCCAAAGGGGGCCGCAAGGCTCCCGCTCCAGGAGGGGCCCCGCGCACCATCAGCTCGTTGGCGGGTAACGCCACCAAGGCTCACGGTTAGCTGGTCT
>FQH3XDB01CYS9E|368|1
AACGAACGCTGGCAGCGTGGATTAGGCATGCAAGTCGGACGGGATCGGACGGGCAGCAATGTTCGTCCGTGAGAGTGGCGGATTGGCGAGGAACACGTGGGCAACCTGCCCCGGAGTCGGGGAAAACCTCTGGAAACGGAGGCTAATACCGGATGTGGCGCGGAGGCGGCATCGCCGCCGCGCCAAAGGGGCCGCAAGGCTCCCGCTCCGAGGGACGGGGCCCGCGCACCATCAGCTCGGTTGGCGGGTAACGCCACCAGGCTCACGGTT
>FQH3XDB01C9I1L|369|1
AACGAACGCTGGCAGCGTGGATTAGGCATGCAAGTCGGACGGGATCGGACGGGCAGCAATGTTCGTCCGTGAGAGTGGCGGATTGGCGAGGAACACGTGGGCAACCTGCCCCCGGAGTCGGGGAAAACCTCTGGAAACGGAGGCTAATACCGGATGTGGTTCTTCAACGCATGTTGAAGATACTAAAGCTTGAAATGGCGCTTTAAGAGGGGCTTGCGGCCTATCAGCTGTTGGTGAGGTAACGGCTCACCAAGGCAAAGACGGGTA
>FQH3XDB01DO8V7|370|3
AACGAACGCTGGCAGCGTGGATTAGGCATGCAAGTCGGACGGGATCGGACGGGTAGCAATATTCGTCCGTGAGAGTGGCGGATTGGCGAGGAACACGTGGGCAACCTGCCCCGGAGTCGGGGAAAACCTCTGGAAACGGAGGCTAATACCGGATGTGGCGCGGAGGCGGCATCGCCGCCGCGCCAAAGGGGGCCGCAAGGCTCCCGCTCCAGGAGGGGCCCGCGCACCATCAGCTCGTTGGCGGGGTAACGGCCCACCAAGGCTCACGGTTAGCTGGTCTGAGAGGATGGACCCAGCCGCACTGGGACTGAGATACC
>FQH3XDB01CIHN5|371|1
AACGAACGCTGGCAGCGTGGATTAGGCATGCAAGTCGGACGGGATCGGACGGGCAGCAATGTTCGTCCGTGAGAGTGGCGGATTGGCGAGGAACACGTGGGCAACCTGCCCCGGAGTCGGGGAAAACCTCTGGAAACGGAGGCTAATACCGGATGTGGTTCTTCAACGCATGTTGAAGATACTAAAGCTTGAAATGGCGCTTTAAGAGGGGCTTGCGGCCTATCAGCTTGTTGGTGAGGTAACGGCTCACCAAGGCAAAGACGGGTAGCGGGTCTGAGAGGACGATCCGCCACACTGGAACTGAGACACGGTCCAGACACCTACGGGTGGCAGCAGTTTCGAATCATTCACAATGGGGGCAACCCTGATGGTGCAACGCCGCGTGGGGATTGAAGGTCTTCGGATTGGTAAACCCCTGTCACACAGGACTAAACGCGAGGTTCATAGCCTCGCCTGATTAC
>FQH3XDB01DXMQQ|372|3
GATGAACGCTGGCGGCGTGCCTAATACATGCAAGTCGAACGCATCACATATGTGATGAGTGGCGAACGGGTGAGTAATACATAAGTAACCTGGCCTTTACAGGGGGATAACTATTGGAAACGATAGCTAAGACCGCATAGGTGTCATAACCGCATGGAGATGACATGAAATATGCCACGGCATAGGTAGAGGATGGACTTATGGCGCATTAGCTAGTTGGAGGGTAACGCCACAAGGCGACGATGCGTAGCCGACTGAGAGGTGACCGGCCACACGTGGGACTGAGAC
>FQH3XDB01CC3OM|373|16
AACGAACGCTGGCGGCAGGCTTAACACATGCAAGTTGAACGGGAGTTAGGATTGCTTGCAATACTAACGAGAGTGGCGCACGGGTGAGTAATACGTGGGAACATACCTATTGGTACGGAATAACGGTTGGAAACGACAGCTAATACCGTATATACCCTGAGGGGGAAAGATTTATCGCCGATAGCATGGCCCGCGGCAGATTAGGCAGTTGGTGGGTAAAGGCCTACCAAACCGACGATCTGTAGCTGGTCTGAGAGGACGATCAGCCACATTGGGACTGAGACACGGCCCAGACCTCCGTA
>FQH3XDB01CAQNQ|374|4
AACGAACGCTGGCGGCACGCTTAACACATGCAAGTCGAACGGATATTGAAAGATATTAGTGGCGGACGGGTGAGTAATATATAGGAATCCACCCATAGGTACGGGATAACAGGGGGAAACTTCTGCTAATACGGTATATACTCTACGGAGGAAAGAATTATCGCCTATGGAGGAGCTTATAACTGATTAGGTAGTTGGTGGGGTAAAAGGCCTACCAAGCCGATGATCAGTAGCTGGTCTGAGAGGACGAACAGCCACATTGGGACTGAGATACGGCCCAGACTCCTACGGGAGGCAGCAGTGAGGAATATTGGACAATGGGGGAACCCTGATCCAGCGATGCCGCGTGAATGAAGAAGGCCTTGGGTTGTAAAGTTCTTTTGATAGTGATGATAATGACAGTAGCTATAGAATAAGGTCCGGCTAACTTCGTGCCAGCAGCC
>FQH3XDB01CKQU2|375|2
AACGAACGCTGGCGGCACGCTTAACACATGCAAGTCGAACGGGACATGGGGCAACCTATGTTTAGTGGCGGACGGGTGAGTAACACGTGGGAACCTGCCTATTGATGGTGGATAACTCTGGGAAACTGGGGCTAATACAGCATAATCTCGAGAGAGGAAAGGCGAAAGTTGTCGATAGAGGGGCCTGCGTCTGATTAGGTAGTTGGTGGGGTAAAGGCCTACCAAGCCGATGATCAGTAGCTGGTCTGAGAGGATGATCAGCCACACTGGAACTGAGATACGGTCCAGACTCCTACGGGAGGCAGCAGTGGGGAATATTGGACAATGGGGGCAACCCTGATCCAGCGATGCCGCGTGAGTGAAGAAGGCCTTAGGGTTGTAAAGCTCTTTTGACGTAGGGGCGAGTGATGACGGTACCTGTAGAATAAGCACCGGCTAACTCTGTGCCAGCAGCC
>FQH3XDB01AP900|376|2
AACGAACGCTGGCGGCACGCTTAACACATGCAAGTCGGACGGTTTATGTTGGTTTACTGACATAGATAGTGGCGAACGGGTGAGTAGAATGCAGGAATCTGCCCATAGGACTGGGATAGCATTGGGAAACTGATGGTAATACTGGATAAGTTGGAGACAAGAAAGGGGAGACCCGCCGATGGAGGAGCCTGTACCTGATTAGATAGTTGGAGGTGTAAGAGACCCCCAAGTCGATGATCAGTAGCTGTTCTGAGAGGAAGATCAGCCACATTGGGGACTGAGATACGGCCCAAAACTCCTACGGGAGGCAGCAGTGGGGAATATTGGACAATGGGGGAACCCTGATCCAGCGATGCCGCGTGAGTGAAGAAGGCCCTAGGGTTGTAAAGCTCTTTTGCCGGGGAGATGATGACGGTACCCGGAGAATAAGCACCGGCTAACTCTGTGCCAGCAGC
>FQH3XDB01DONN3|377|27
AACGAACGCTGGCGGCACGCTTAACACATGCAAGTCGGACGGTGAATGTAGCTTGCTATATTCATAGTGGCGGACGGGTGAGTAATATGTAGGAATCTGCCCATTGGTCTGGGATAGCACCGGGAAACTGGTGGTAATACCGGATAAGTTGGTGACAAGAAAGGTGAAAATCGCCGATGGGGGAGCCTGCACCCGATTAGATAGTTGGTAGTGTAAAAGACTACCAAGTCGATGATCGGTAGCTGTTCTGAGAGGAAGATCAGCCACATTGGGACTGAGATACGGCCCAAACTCCTACGGGAGGCAGCAGTGGGAATATTGGACAATGGGGGAAACCCTGATCCAGCGATGCCGCGTGAGTGAAGAAGGCCCTCGGGTTGTAAAGC
>FQH3XDB01C42N0|378|2
AACGAACGCTGGCGGCACGCTTAACACATGCAAGTCGGACGGTGAATGTAGCTTGCTATATTCATAGTGGCGGACGGGGTGAGTAATATGTAGGAATCTGCCCATTGGGTCTGGGATAGCACCGGGAAACTGGTGGTAATACCGGATAAGTTGGTGACAAGAAAGGTGAAAATCGCCGATGGAGAGCCTGCACCCCGGATTAGATAGTTGGTAGTGTAAAAGACTACCAAGTCGATGATCGGTAGCTGTTCTGAGAGGAAGATCAGCC
>FQH3XDB01BT16X|379|2
AACGAACGCTGGCGGCACGCTTAACACATGCAAGTCGGACGGTGAATGTAGCTTGCTATATTCATAGTGGCGGACGGGTGAGTAATATGTAGGAATCTGCCCATTGGTCTGGGGATAGCACCCGGGGAAACTGGTGGTAATACCGGATAAGTTGGTGACAAGAAAGGTGAAAATCGCCGATGGAGAGCCTGCACCCGATTAGATAGTTGGTAGTGTAAAAGACTACCAAGTCGATGATCGGTAGCTGTTCTGAGAGGAAGATCAGCCACATTGGGACTGAGATACGGCCCAAACTCCTAC
>FQH3XDB01EIKLQ|380|3
AACGAACGCTGGCGGCACGCTTAACACATGCAAGTCGGACGGTTTATGTTGGTTTACTGACATGAACAGTGGCGAACGGGTGAGTAGAATGCAGGAATCTGCCCATAGGACTGGGATAGCATTGGGAAACTGATGGTAATACTGGATAAGTTGGAGACAAGAAAGGGGAGACCCGCCGATGGAGGAGCCTGTACCTGATTAGATAGTTGGAGGTGTAAGAGACCCCCAAGTCGATGATCAGTAGCTGTTCTGAGAGGAAGATCAGCCACATTGGGACTGAGATACGGCCCAAACTCCTACGGGAGCAGCAGTGGGGAATATTGGACAAGTGGGGGAACCCTGATCCAGCGATGCCGCGTGAGT
>FQH3XDB01EKQ04|381|2
AACGAACGCTGGCGGCACGCTTAACACATGCAAGTCGTACGGACGGAGCTTGAGGTAACTTGAGTGAAGTTAGTGGCGGACTGGTGAGTATAGTACAGGAATATGCCCTTTAGACTGGGATAGCTCTGGGAAACCGGAGGTAATACCGGATGAGGTCGAGAGATGAAAGCAGAGATGCGCTGAAGGAGTAGCCTGTATCTGATTAGGAAGTTGGCGGGGTAAAGGCCACCAATCCAGAGATCAGTAGCTGTTCTGAGAGGAAGATCAGCCACATTGGGACTGAGATACGGCCCAGACTCCTACGGGAGGCAGCAGTGGGGAATATGGACAATGGGGGAGACCCTGATCCAGCGATGCCGCGTGAGTGAAGAAGATTTT
>FQH3XDB01CEBPK|382|37
AACGAACGCTGGCGGCACGCTTAACACATGCAAGTCGTACGGATGGAACTTAGTTTACTAGGAGAAGTTAGTGGCGGACTGGTGAGTATAATATAGGAATGTGCCCTTGGGACTGGGATAGCTTTGGGAAACTGGAGGTAATACCGGATGAGATTGAGGAATGAAAGCAGAGATGCGCCTGAGGAGTAGCCTGTACCTGATTAGGAAGTTGGCGGGGTAAGAGCCCACCAATCCAGAGATCAGTAGCTGTTCTGAGAGGAAGATCAGCCACATCGGGACTGAGATACGGCCCAGACTCCTACGGGAGGCAGCAGTGGGGAATATTGGACAATGGGGAAACCCTGATCCAGCGATGCCGCGTGAGTGAAGAAGGTTTTCGGATTGTAGAGCTCTTTTCTTAGAGAAGATGATGACGGTATCTAAAGGAATAAGCACCGGCTAAACTCTGTGCCAGCAGCCG
>FQH3XDB01DLZSV|383|3
AACGAACGCTGGCGGCACGCTTAACACATGCAAGTCGTACGGATGGAACTTAGTTTACTAGGAGAAGTTAGTGGCGGACTGGTGAGTATAATATAGGAATATGCCCTTGGGACTGGGATAGCTTTGGGAAACTGGAGGTAATACCGGATGAGATTGAGGAATGAAAGCAGAGATGCGCCTGGGGAGTAGCCTGTACCTGATTAGGAAGTTGGCGGGGTAAGAGCCCACCAATCCAGAGATCAGTAGCTGTTCTGAGAGGAAGATCAGCCACATTGGGACTGAGATACGGCCCAGACTCCTACGGGAGGCAGCAGTGGGGAATATTGGACATGGGGAAACCTGATCCAGCGATGCCGCGTGAGTGAAGAAGGTTTTCGGATTGTAAAGCTTCTTTTCTTAGAGAAGATGATGACGGTATCTAAGGAATAAGCA
>FQH3XDB01DETR2|384|7
AACGAACGCTGGCGGCACGCTTAACACATGCAAGTCGTACGGATGGAGCTTAGTTTACTAGGTGAAGTTAGTGGCGGACTGGTGAGTATAATATAGGAATATACCCCTTGGGACTGGGATAGCTTTGGGAAACTGGAGGTAATACCGGATGATATTGAGGAATGAAAGCAGAGATGCGCCTGAGGATTAGCCTGTACCTGATTAGGAAGTTGGCGGGTAAAGCCCACCAATCCAGAGATCGGTAGCTGTTCTGAGAGGAAGATCAGCCACATTGGGACTGAGATACGGCCCAGACTCCGTACGGGAGGCAGCAGTGGGAAT
>FQH3XDB01BDDAS|385|1
AACGAACGCTGGCGGCACGCTTTAAGCATGCAAGTCGTGGGGGCAACAGTGTAGCAATACAGCTGGCGACCGGCGCACGGGTGAGTAACACGTAAGGATCTGCCTAAAGGTCACGAATAGCTAATGGAAACATTTGGTAATGCGTGATGGAACCGAGAGGTTAAAGCTAAGCGATTAGTGCCTTTAGATGAACTTGCGGTTCATTAGCTAGTTGGTGGGGTAAGAGCCTACCAAGGCGACGATGGATAGCCGGCCTGAGAGGGTGAACGGCCACAATGGAACTGAGACACGGTCCATACTCCTACGGGAGGCAGCAGCTAGGAATCTCCGCAATGGGCGCGAGCCTGACGGAGCGATGCCGCGTGAACG
>FQH3XDB01B68QI|386|38
AACGAACGCTGGCGGCACGCTTTAAGCATGCAAGTCGTGGGGCAACAGTGTAGCAATACAGCTGGCGACCAGCGCACGGGTGAGTAACACGTAAGGATCTGCCTAAAGGTCACGAATAGCTAATGGAAACATTTGGTAATGCGTGATGGAACCGAGAGGTTAAAGCTAAGCGATTAGTGCCTTTAGATGAACTTGCGGTTCATTAGCTAGTTGGTGGGGTAAGAGCCTACCAAGGCGACGATGGATAGCCGGCCTGAGAGGGTGAACGGCCACAATGGAACTGAGACACGGTCCATACTCCTACGGGAGGCAGCAGCTAGGATCTCCGCAATGGG
>FQH3XDB01BID4L|387|2
AACGAACGCTGGCGGCACGCTTTAAGCATGCAAGTCGTGGGGCAACAGTGTAGCAATACAGCTGGCGACCGGCGCACGGGTGAGTAACACGTAAGGATCTGCCTAAAGGTCACGAATAGCTAATGGAAACATTTGGTAATGCGTGATAGAACCGAGAGGTTAAAGCTAAGCGATTAGTGCCTTTAGATGAACTTGCGGTTCATTAGCTAGTTGGTGGGTAAAGGCCTACCAAGGCGACGATGGATAGCCGGCCTGAGAGGGTGAACGGCCACAATGGAACTGAGACACGGTCCATACTCCTACGGGAGCAGCAGCTAGGAATCTTCCGCAATGGGCGCGAGCCTGACGGAGCGATGCCGCGTGAA
>FQH3XDB01C5JDU|388|10
AACGAACGCTGGCGGCGCGTCTTAAGCATGCAAGTCGAGCGGCAAGATTGGGGCTTGCTCCAATCCTAGAGCGGCGGACTGGTGAGTAACACGTAGGCGACGTGCCCTTGTGACGGGGATAGCCTGTGGAAACACAGGGTAATACCGGATAAGGCCGTATGCGTTGGAGGCATACGGGGAAAGGAGCTATGGCTTCGCACAAGGAGCGACCTGCGGCCCATCAGCTAGTTGGCGGGGTAAGAGCCCACCAAGGCAATGACGGGTATCCGGCCTGAGAGGGTGAACGG
>FQH3XDB01BDSPV|389|131
AACGAACGCTGGCGGCGCGTCTTAAGCATGCAAGTCGGGCGGGATCCATGCGCTTGCGCATGGTGAGAGCGGCGGACTGGCGAGTAACACGTGGGCGACGCGCCCTCCGGACGGGAATAGCCTGTAGAAATACAGGGTAATGCCGGATGCGAACGCACGGGCTGGAGCCGTGCGTGGAAAGCCCCCAGGGGCGCCGGAGGAGCGGCCCGCGGCCCATCAGCTTGTAGGCGGTGCAAGGGACCACCTAGGCTACGACGGGTACCCGGCCTCAGAGGGCGGACGGGCGCATTGGGACTGAGATACGGCCCAGACTCCTACGGGAGGCAGCAGCTAAGAATATTCCGCAAGTGGGGGGAACCCTGACGGAGCGACGCCG
>FQH3XDB01BPZM8|390|24
AACGAACGCTGGCGGCAGGCCTAAAACATGCAAGTTGAGCGGGTGTAGCAATACATCAGCTGCGCACGGGTGAGTAATGTATGGGAACATGTCTTTTGGTGGGGGATAGCTTCTGGAAACGGGAGATAATACCGCATAAGCCCTGAGGGGAAAGATTTATTGCCGAAAGGTTGGCCCATATTGGATTAGCTAGTTGGTTAGGTAACGGCTGACCAAGGCGACGATCCATAGCTGGTTTGAGAGGATGATCAGCCACACTGGGACTGAGACACGGCCCAGACTCCTACGGGAGGCAGCAGTTAGGAATATTGGACAAGTGGGGGGAACCCTGATCCAGCCATGCCGCGTGAGTGACGAGGCCTTCGGTTGTAAGCTCTTTT
>FQH3XDB01CJY82|391|34
AACGAACGCTGGCGGCAGGCCTAAAACATGCAAGTTGAGCGGGTGTAGCAATACATCAGCAGCGCACGGGTGAGTAATGTATGGGAACATGTCTTTTGGTGGGGATAGCTTCTGGAAACGGGAGATAATACCGCATAAGCCCTGAGGGGGAAAGATTTATTGCCGAAAGGTTGGCCCATATTGGATTAGCTAGTTGGTTAGGTAACGGCTGACCAAGGCGACGATCCATAGCTGGTTTGAGAGGATGATCAGCCACACTGGGACTGAGACACGGCCCAGACTCCTACGGGAGCAGCAGTTAGGAATATTGGACAATGGGGGAACCCTGATCCAGCCATGCCGCGTGAGTGACGAAGGCCTTCGGGTTGTAAAGCTCTTTGTACGGGAAGATGGATGA
>FQH3XDB01A5S6C|392|1
AACGAACGCTGGCGGCAGGCCTAAAACATGCAAGTTGAACGCCATAGCAATATGGAGTAGCGCACGGGTGAGTAATACATGGGAACATGTCTTTCGGTGGGGGATATTCCTTGGAAACGAGGAGCAATACCGCATACGTCCTTTGGGAGAAAAGATTTATCCGGCCGATTGAGTGGCCCATGCTAGATTAGCTAGTTGGTAGGTAATGGCTGACCAAGGCAATTGATCTATAGCTGGTCTGAGAGGACGACCAGCCACATTGGGACTGAGACACGGCCCAAAA
>FQH3XDB01BJHJE|393|6
AACGAACGCTGGCGGCAGGCCTAAAACATGCAAGTTGAACGGTTATAGCGATATAGCAGTAGCGCACGGGTGAGTAATGTATGGGAACATGTCTTTTGGTGGGGATAGCTTCTGGAAACGGGAGATAATACCGCATAAGCCCTGAGGGGGAAAGATTTATTGCCGAAAGGTTGGCCCATATTAGATTAGCTAGTTGGTTAGGTAAAGGCTGACCAAGGCGATGATCTATAGCTGGTCTGAGAGGATGATCAGCCACACTGGGACTGAGACACGGCCCAGACTCCTACGGGAGGCAGCAGTTAGGAATATTGGACAATGGGGGAACCCTGATCCAGCCATGCCGCGTGAGTG
>FQH3XDB01DM5FY|394|3
AACGAATGCTGGCGGCAGGCCTAAAACATGCAAGTTGAGCGGGTGTAGCAATACATCAGCAGCGCACGGGTGAGTAATGTATGGGAACATGTCTTTTGGTGGGGGATAGCTTCTGGAAAACGGGGAGATAATACCGCATAAGCCCTGAGGGGAAAGATTTATTGCCGAAAGGTTGGCCCATATTGGATTAGCTAGTTGGTTAGGTAACGGCTGACCAAGGCGACGATCCATAGCTGGTTTGAGAGGATGATCAGCCACACTGGGACTGAGACACGGCCCAGACTCCTACGGGAGGCAGCAGTTAGGAATATTGG
>FQH3XDB01DTKLH|395|1
AACGAACGCTGGCGGCAGGCCTCAAACATGCAAGTCTAGGGGTGTAGCAATACATCACCGGCGGACGGGTGCGTAACGCGTGGGAATATACCTATCGATTCGGAACAACTGAGGGAAACTTCAGCTAATACCGAATACGTCCTTACGGAGAAAGATTTATCGTCGATAGATTAGCCCGCGTTCGAATTAGCCTAGTTGGTGGGGTAACCGGCCCACCAAGGCGACGATCGATAGCTGTTCTGAGAGGAAGATCAGCCACACTTGGGACTGAGACACGGCCCAGACTCCTACGGGAGGCAGCAGTTGGGAATCTTGGACAATGGGGGAAACCCTGATCCAGCCATGCCGCGGTAATCTCTCGACGTCG
>FQH3XDB01ES5IV|396|2
AACGAACGCTGGCGGCAGGCTTAACACATGCAAGTCAAGGGGTGTAGCAATACACAACCGGCGCACGGGTGAGTAACGCGTGGGAATATGTCCATTTGTGGGGGATAGCTTCTGGAAACGGAAGGTAATACCGCATAAGCCCTGAGGGGAAAGATTTATCGCGAATGGAGTGACCCGCGTTGGATTAGTTAGTTGGTTAGGTAAAGGCTGACCAAGGCGAAGATCCATAGCTGGTCTGAGAGGACGATCAGCCACATTGGGACTGAGACACGGCCCAGACTCCTACGGGAGGCAGCAGTAAGGAATATTGGACAAATGGGGGCAAACCCTGATCCAGCCATGCCGCGTGAGTGAAGAAGGCCTTCGGGTTGGTAAATACTCTTTAGATGGGAAGATGATGA
>FQH3XDB01DO1V9|397|1
AACGAACGCTGGCGGCAGGCTTAACACATGCAAGTCAAGGGGGTGTAGCAATACACAACCGGCGCACGGGTGAGTAACGCGTGGGAATATGTCCATTTGTGGGGGATAGCTTCTGGAAACGGAAGGTAATACCGCATAAGCCCTGAGGGGGAAAGATTTATCGCGAATGGAGTGGCCCGCGTTGGATTAGTTAGTTGGTTAGGTAAAGGCTGACCAAGGCGAAGATCCATAGCTGGTCTGAGAGGACGATCAGCCACATTGGGACTGAGACACGGCCCAGACTCCTACGAGAGGCAGCAGTGGGGAATAGTTGGGCAAGTGGGGGAAACCCCTG
>FQH3XDB01BL26T|398|1
AACGAACGCTGGCGGCAGGCTTAACACATGCAAGTCAAGGGGGTGTAGCAATACACAACCGGCGCACGGGTGAGTAACGCGTGGGAATATGTCCATTTGTGGGGGATAGCTTCTGGAAACGGAAGGTAATACCGCATAAGCCCTGAGGGGGAAAGATTTATCGCGAATGGGAGTGGCCCGCGTTGGATTAGTTAGTTGGTTAGGTAAAGGCTGACCAAGGCGAAGATCCATAGCTGGTCTGAGAGGACGATCAGCCACATTGGGACTGAGACACGGCCCAGACTCCTACGGGAGGCAGCAGTAAGGAATATTGGACAAGTGGGGCAACCCTGATCCAGCCATGCCGCGTGAGTGAAGAGGCCTTCGGGTTGTAAATCTTCTTTTAGATTGGGAAGATGATGACGGTACCATCGGAATAAGCACCGG
>FQH3XDB01B9Z5S|399|1
AACGAACGCTGGCGGCAGGCTTAACACATGCAAGTCAAGGGGGTGTAGCAATACACAACCGGCGCACGGGTGAGTAACGCGTGGGAATATGTCCATTTGTGGGGGATAGCTTCTGGAAACGGAAGGTAATACCGCATAAGCCCTGAAGGGGGAAAAGATTTATCGCGAATGGAGTGGCCCGCGTTGGATTAGTTAGTTGGTTAGGTAAAGGCTGACCAAGGCGAAGATCCATAGCTGGTCTGAGAGGACGATCAGCCACATTGGGACTGAGACACGGCCCAGA
>FQH3XDB01A1ITF|400|1
AACGAACGCTGGCGGCAGGCTTAACACATGCAAGTCAAGGGGTGTAGCAATACACAACCGGCGCACGGGTGAGTAACGCGTGGGAATATGTCCATTTGTGGGGATAGCTTCTGGAAACGGAAGGTAATACCGCATAAGCCCTGAGGGGAAAGATTTATCGCGAATGGAGTGGCCCGCGTTGGATTAGTAGTTGGTTAGTAAAGGCTGACCAAGGCGAAGATCCATAGCTGGTCTGAGAGGACGATCAGCCACATTGGGACTGAGACACGG
>FQH3XDB01CK2W7|401|1
AACGAACGCTGGCGGCAGGCTTAACACATGCAAGTCGAACGGAATACAGGGAGCTTGCTTTCTGTATCAGTGGCGCACGGGTGAGTAACGCGTAGGTATCTGTCTATGGGAGTTGGATAGCTTCTGGAAACGGAAGGTAAAACAGCATAAGCCCTGCGGGGGAAAGTGAGAAATCAGCCGATAGAGGAGCCTGCGTTAGATTAGGCAGTTGGTGGGTAACGGCCTACCAACCGACGATCTATAGCTGGTCTGAGAGGATGATCAGCCACATTGGGACTGAGACACGGCCCAAACTCCTACGGGAGGCAGCAGTGGGGAATATTGCACAATGGGGGAAACCCTGATGCAGCGACGCCGCGTGGAGGAAGAAGGTCTTCGGATTGTAAACTCCTGTTGTTGAGGAAGATAATGACGGTACTCAACAAGGAAGT
>FQH3XDB01BUA32|402|1
AACGAACGCTGGCGGCAGGCTTAACACATGCAAGTCGAACGGAATATGATGTGCTTGCACATTATATCAGTGGCGCACGGGTGAGTAACGCGTAGGAATCTGTCTATTGGACAGGGATAGCCTCTGGAAACGGAGGGTAAAACCTGGTAAGACCTGAGGGTGAGACGAGTAATCGGCCGATAGAGGAGCCTGCGTTAGATTAGGCAGGTTGGTGGGGTAACGGCCCTACCAAACCGACGATCTATAGCTGGTCTGAGAGGATGATCAGCCACATTGGGACTGAGACACGG
>FQH3XDB01CVBD4|403|1
AACGAACGCTGGCGGCAGGCTTAACACATGCAAGTCGAACGCTCGTAGCAATACGGGAGTGGCAGACGGGTGAGTAACGCGTGGGAACATACCCTCCGGTTCGGAATAGCTCAGGGAAACTTGGATTAATACCGGATACGGCTGAGAGGCGAAAGATTTATCGCCGGAGGATTGGCCCGCGTTCGATTAGCTAGTTGGTGAGGTAACGGCTCACCAAGGCGATGATCGATAGCTGGTCTGAGAGGATGATCAGCCACATTGGGACTGAGACACGGCCCAAAACTCCTACGGGAGCAGCAGTGGGGAATATTGGACAATGGGCGCAAGCCTGATCCAGCCATGCCGCGTGAGTGATGACGG
>FQH3XDB01EKFK2|404|1
AACGAACGCTGGCGGCAGGCTTAACACATGCAAGTCGAACGGAATACAGAGAGCTTGCTTTCTGTATCAGTGGCGCACGGGTGAGTAACGCGTAGGTATCTGTCTATGGGAGTTGGATAGCTTCTGGAAACGGAAGGTAAACAGCATGAGCCCTTCGGGGGAAAGTGAGGAATCAGCCGATAGAGGAGCCTGCGTTAAGATTAGGCCAGGTTGGTGGGGTAACGGCCTACCAAACCGACGATCTATAGCTGGTCTGAGAGGATGATCAGCCACATTGGGGACTGAGACACGGCCCAAAACTCCTACGGGAGCAGCAGTGGGGAATATTGGACAATGGGGGA
>FQH3XDB01CU2F0|405|8
AACGAACGCTGGCGGCAGGCTTAACACATGCAAGTCGAACGGAATACAGGGAGCTTGCTTTCTGTATCAGTGGCGCACGGGTGAGTAACGCGTAGGTATCTGTCTATGGGAGTTGGATAGCTTCTGGAAACGGAAGGTAAAACAGCATAAGCCCTGCGGGGGAAAGTGAGAAATCAGCCGATAGAGGAGCCTGCGTTAGATTAGGCAGGTTGGTGGGTAACGGCCTACCAACCGACGATCTATAGCTGGTCTGAGAGGATGATCAGCCACATTGGGACTGAGACACGGCCCAAACTCCTACGGGAGGCAGCGGTGGGAATATTGGACAAGTGGGGAGACCCTGGATCCAGCCATGGCCGCGTGAGTGAAGA
>FQH3XDB01CANY6|406|5
AACGAACGCTGGCGGCAGGCTTAACACATGCAAGTCGAACGGAATACAGGGAGCTTGCTTTCTGTATCAGTGGCGCACGGGTGAGTAACGCGTAGGTATCTGTCTATGGGAGTTGGATAGCTTCTGGAAACGGAAGGTAAAACAGCATAAGCCCTGCGGGGAAAGTGAGAAATCAGCCGATAGAGGAGCCTGCGTTAGATTAGGCAGTTGGTGGGTAACGGCCTACCAAACCGACGATCTATAGCTGGTCTGAGAGGATGATCAGCCACATTGGGACTGAGACACGGCCCAAAACTCCTACGGGAGGCAGCAGTGGGGAATATTGGACAATGGGGAGACCCTGATCCAGCCATGCCGCGTGAGTGAAGAAGGCCT
>FQH3XDB01CC3JE|407|5
AACGAACGCTGGCGGCAGGCTTAACACATGCAAGTCGAACGGAATACAGGGAGCTTGCTTTCTGTATCAGTGGCGCACGGGTGAGTAACGCGTAGGTATCTGTCTATGGGAGTTGGATAGCTTCTGGAAAACGGAAGGTAAAACAGCATAAGCCCTGCGGGGGAAAGTGAGAAATCAGCCGATAGAGGAGCCTGCGTTAGATTAGGCAGTTGGTGGGTAACGCCTACCAACCGACGATCTATAGCTGGTCTGAGAGGATGATCAGCCACATTGGGACTGAGACACGGCCCAAAACTCCTACGGGAGGCAGCGGTGGGGAATAGTTGGACAAGTGGGGAGACCCTGATCCAGCCATGCCGCGTGAGTGAAG
>FQH3XDB01D6XB8|408|1
AACGAACGCTGGCGGCAGGCTTAACACATGCAAGTCGAGCGCCCCGCAAGGGGAGCGGCAGACGGGTGAGTAACGCGTGGGAATCTACCTTTTGCTACGGAATAACTCAGGGAAACTTGTGCTAATACCGTATGTGCCCTTCGGGGGAAAGATTTATCGGCAAAGGATGAGCCCGCGTTGGATTAGCTAGTTGGTGGGTAAAGGCCCACCAAGGCGACGATCCATAGCTGGTCTGAGAGGATGATCAGCCACACTGGGACTGAGACACGGCCCCAGACTCCTACGGGAGGCAGCAGTGGGGAATATTGGACAATGGGCGCAAGCCTGATCCAGCCATGCCGCGTGAGTGATGAAGGCCCTAGGGTTGTAAAG
>FQH3XDB01AL3LY|409|1
AACGAACGCTGGCGGCAGGCTTAACACATGCAAGTCGAACGGAACATAAGGAGCTTGCTTCTTATGTCAGTGGCGCACGGGTGAGTAACGCGTAGGAATCTGTCTATTGGACTGGGATAGCTTCTGGAAACGGAAGGTAAAACCGGGTAAGACCTGAGGGTGAGACGAGTAATCGGCCGATAGAGGAGCCTGCGTTAGATTAGGCAGTTGGCGGGTAACGGCCACCAAACCGACGATCTATAGCTGGTCTGAGAGGATGATCAGCCACATTGGGACTGAGACACGGCCCAAACTCCTACGGGAGGCAGCAGTGGGGAATATTGGACAATGGGGGAGACCCTGATCCAGCCATGCCGCGTGAGTGATGACGG
>FQH3XDB01EZ6E6|410|2
AACGAACGCTGGCGGCAGGCTTAACACATGCAAGTCGAACGGAATACAGGGAGCTTGCTTTCTGTATCAGTGGCGCACGGGTGAGTAACGCGTAGGTATCTGTCTATGGGAGTTGGATAGCTTCTGGAAACGGAAGGTAAAACAGCATAAGCCCTGCGGGGAAAGTGAGAAATCAGCCGATAGAGGAGCCTGCGTTAGATTAGGCAGTTGGTGGGTAACGGCCTACCAACCGACGATCTATAGCTGGTCTGAGAGGATGATCAGCCACATTGGGACTGAGACACGGCCCAAACTCCTACGGGAGCAGCAGTGGGAAT
>FQH3XDB01B3MPT|411|2
AACGAACGCTGGCGGCAGGCTTAACACATGCAAGTCGAACGGAATACAGGGAGCTTGCTTTCTGTATCAGTGGCGCACGGGTGAGTAACGCGTAGGTATCTGTCTATGGGAGTTGGATAGCTTCTGGAAACGGAAGGTAAACAGCATAAGCCCTGCGGGGGAAAGTGAGAAATCAGCCGATAGAGGAGCCTGCGTTAGATTAGGCAGTTGGTGGGTAACGCCTACAACGACGATCTATAGCTGGTCTGAGAGGATGATCAGCCACATTGGGACTGAGACACGGCCCAAAA
>FQH3XDB01BK7I9|412|9
AACGAACGCTGGCGGCAGGCTTAACACATGCAAGTCGAACGGAATACAGGGAGCTTGCTTTCTGTATCAGTGGCGCACGGGTGAGTAACGCGTAGGTATCTGTCTATGGGAGTTGGATAGCTTCTGGAAACGGAAGGTAAAACAGCATAAGCCCTGCGGGGAAAGTGAGAAATCAGCCGATAGAGGAGCCTGCGTTAGATTAGGCAGTTGGTGGGTAACGCCTACCAACCGACGATCTATAGCTGGTCTGAGAGGATGATCAGCCACATTGGGACTGAGACACGGCCCAAAACTCCTACGGGAGGCAGCAGTGGGGAATATTGGACAATGGGGG
>FQH3XDB01C3WRU|413|5
AACGAACGCTGGCGGCAGGCTTAACACATGCAAGTCGAACGGAATACAGGGAGCTTGCTTTCTGTATCAGTGGCGCACGGGTGAGTAACGCGTAGGTATCTGTCTATGGGAGTTGGATAGCTTCTGGAAACGGAAGGTAAAACAGCATAAGCCCTGCGGGGGAAAGTGAGAAATCAGCCGATAGAGGAGCCTGCGTTAGATTAGGCAGTTGGTGGGTAACGCCTACCAACCGACGATCTATAGCTGGTCTGAGAGGATGATCAGCCACATTGGGACTGAGACACGGCCCAAACTCCGTACGGGAGGCAGCAGTGGGGAATA
>FQH3XDB01B61AK|414|1
AACGAACGCTGGCGGCAGGCTTAACACATGCAAGTCGAACGGAATACAGGGAGCTTGCTTTCTGTATCAGTGGCGCACGGGTGGGTAACGCGTAGGTATCTGTCTATGGGAGTTGGATAGCTTCTGGAAAACGGAAGGTAAAACAGCATAAGCCCTGCGGGGAAAGTGAGAAATCAGCCGATAGAGGAGCCTGCGTTAGATTAGGCAGTTGGTGGGTAACGCTACAAACCGACGATCTATAGCTGGTCTGAGAGGATGATCAGCCACATTGGGACTGAGACACGGCCCAAAA
>FQH3XDB01E0GVA|415|1
AACGAACGCTGGCGGCAGGCTTAACACATGCAAGTCGAGCGGGCCCTTCGGGGTCAGCGGCAGACGGGTGAGTAACACGTGGGAATCTACCCAACCCTACGGAACAACTCCGGGGAAACTGGAGCTAATACCGTATACGCCCTTCGGGGGAAAGATTTATCGGGGATGGATGAGCCCGCGTTGGATTAGCTAGTTGGTGGGGTAAAGGCCTACCAAGGCGACGATCCATAGCTGGTCTGAGAGGATGATCAGCCACATTGGGGACTGAGACACGGCCCAAACTCCTACGGGAGGCAGCAGTGGGGAATATTGGACAATGGGCGCAAGCCTGATCCAGCCATGCCGCGTGAGTGATGAAGGCCTTAGGGTGTAAAGCTCTTTCACCGATGAAGATAATCGACGGTAGTCGGAGAA
>FQH3XDB01AH0RS|416|3
AACGAACGCTGGCGGCAGGCTTAACACATGCAAGTTGAACGAGAGTTATCTTAGCTTGCTAAGATAATGAAAGTGGCGCACGGGTGAGTAAGACATGGGAACATACCCTGAAGTGGGGAATAACAGTTGGAAACGACTGCTAATACCGCATAATCCCTGCGGGGGAAAGATTAATCGCTTTAGGAATGGCCCATGGCAGATTAGGCAGTTGGTGGGTAAAGGCTCACCAAACCGACGATCTGTAGCTGGTCTGAGAGGACGATCAGCCACACTGGAACTGAGACACGGTCCAGACTCCTACGGGAGGCAGCAGTGAGGAATATTGGGCAATGGGGGGAACCCTGACCCAGCCATGCCGC
>FQH3XDB01ENKGE|417|2
AACGAACGCTGGCGGCAGGCTTAACACATGCAAGTTGAACGAGAGTTATCTTAGCTTGCTAAGATAATGAAAGTGGCGCACGGGTGAGTAAGACATGGGAACCATACCCTGAAGTGGGGAATAACAGTTGGAAACGACTGCTAATACCGCATAATCCCTGCGGGGGAAAGATTAATCGGCTTTAGGGAATGGCCCATGGCAGATTAGGCAGTTGGTGGGTAAAGCTCACCAACCGACGATCTGTAGCTGGTCTGAGAGGACGATCAGCCACACTGGAACTGAGACACGGTCCAGACTCCTAC
>FQH3XDB01DG6LB|418|17
AACGAACGCTGGCGGCAGGCTTAACACATGCAAGTTGAACGGGAACATACGATAGCTTGCTATAGTATGTGAGAGTGGCGCACGGGTGAGTAATACATGGGAACATACCTTAAGGTGGGGGATAACTTCTGGAAACGGATGCTAATACCGCATATACCCTGAGGGGGAAAGATTTATCGCTTTAAGATTGGCCCATGGCAGATTAGGTAGTTGGTGGGGTAAAGGCCTACCAAGCCGACGATCTGTAGCTGGTCTGAGAGGACGATCAGCCACATTGGGACTGAGACACGGCCCAGACTCCTACGGGAGGCAGCAGTGAGGAATATTGGGCAATGGGGGCAACCCTGACCCAGCCATGCCGCGTGAGTGAAGAAGGTTTCGAATTGTAAAGCTCTTTCGGATGTGACGATGATGACGGTAGCATC
>FQH3XDB01EK3YS|419|2
AACGAACGCTGGCGGCAGGCTTAACACATGCAAGTTGAACGGGAACATACGATAGCTTGCTATAGTATGTGAGAGTGGCGCACGGGTGAGTAATACATGGGGAACATACCTTAAGGTGGGGGATAACTTCTGGAAACGGATGCTAATACCGCATATACCCCTGAGGGGGAAAGATTTATCGCTTTTAAGGATTGGCCCATGGCAGATTAGGTAGTTGGTGGGGTAAAAGGCCTACCAAGCCGACGATCTGTAGCTGGTCTGAGAGGACGATCAGCCACATTGGGACTGAGACACGGCCCCAGACTCCTACGGGAGGCAGCAGTGAGGATATTGGGCAATGGGGGCAACCCTGACCCAGCCATGCCGCGTGAGTGAAGAAGGTTTTCGAATTGTAAAGCTCTTTCGGATGTGACGATGATGA
>FQH3XDB01BP6BQ|420|1
AACGAACGCTGGCGGCAGGCTTAACACATGCAAGTTGAACGGGAACATACGATAGCTTGCTATAGTATGTGAGAGTGGCGCACGGGGTGAGTAATACATGGGAACATACCTTAAAGTGGGGGATAACTTCTGGAAACGGACGCTAATACCGCATATACCCCTGAGGGGGAAAGATTTATCGCTTTAAGATTGGCCCATGGCAGATTAGGTAGTTGGTGGGGTAAAGGCCTACCAAGCCGACGATCTGTAGCTGGTCTGAGAGGACGATCAGCCACATTGGGACTGAGACACGGCCCAGACTCCTACGGAGGCAGCAGTGAGGAATAGTTGGGCAAGTCGGGGGCAACCCTGACCCCAGCCATGCCGCGTGAG
>FQH3XDB01BCSPW|421|5
AACGAACGCTGGCGGCAGGCTTAACACATGCAAGTTGAACGGGATCATACGGTAGCTTGCTATTGTATGTGAGAGTAGCGCACGGGTGAGTAATACATGGGAACATACCTATCTGTACGGAATAACTTCTGGAAACGGATGCTAATACCGTATACGCCCTGAGGGGGAAAGATTTATTGCAGATAGCATGGCCCATGGCAGATTAGGCAGTTGGTGGGGTAAAGGCCTACCAAACCGACGATCTGTAGCTGGTCTGAGAGGACGATCAGCCACATTGGGACTGAGACACGGCCCAGACTCCTACGGGAGCAGCAGTGAGGATATTGGGCAATGGGGCAA
>FQH3XDB01DV1YE|422|1
AACGAACGCTGGCGGCAGGCTTAACACATGCAAGTTGAACGGGATCATACGGTAGCTTGCTATTGTATGTGAGAGTAGCGCACGGGTGAGTAATACATGGGAACATACCTATCTGTACGGAATAACTTCTGGAAACGGATGCTAATACCGTATACGCCCTGAGGGGGAAAGATTTATTGCAGATAGCATGGCCCATGGCAGATTAGGCAGTTGTGGGTAAGGCTACAAACCGACGATCTGTAGCTGGTCTGAGAGGACGATCAGCCACATTGGGACTGAGACACGGCCCAGACTCCTACGGGAGGCAGCAGTGAGGAATATTGGGCAATGGGGGCAACCCTGACCCAGCCATGCCGCGTGAGTGAAGAAGGTTTTCGGATTGTAAAGCTCTTTCGGGTGTGACGATGATGACGGTAGCACCT
>FQH3XDB01D9CXJ|423|13
AACGAACGCTGGCGGCAGGCTTAACACATGCAAGTTGAACGGGATCATATGATAGCTTGCTATTGTATGTGAGAGTAGCGCACGGGTGAGTAATACATGGGAACATACCTATTTGTTCGGAATAACTTCTGGAAACGGATGCTAATACCGGATACGCCCTGAGGGGAAAGATTTATCGCAAATAGCATGGCCCATGGCAGATTAGGCAGTTGGTGGGTAAAGGCCTACCAAACCGACGATCTGTAGCTGGTCTGAGAGGACGATCAGCCACATTGGGACTGAGACACGGCCCAGACTCCTACGGGAGGCAGCAGTGAGGAATATTGGGCAATGGGGGCAACCCTGA
>FQH3XDB01A9IFH|424|4
AACGAACGCTGGCGGCAGGCTTAACACATGCAAGTTGAACGGGATTCAATTGGTGCTTGCACTGATTGATGAGAGTGGCGCACGGGTGAGTAATACGTGGGAACATATCTGACAGAGCGGAATAACGGTTGGAAACGACAGCTAATACCGCATACGCCCTGAGGGGAAAGATTTATCGCTGACAGGATGGCCCGCGGCAGATTAGGCAGTTGGTGGGTAAGGCCTACCAACCGACGATCTGTAGCTGGTCTGAGAGGACGATCAGCCACATTGGGACTGAGACACGGCCCAGACTCC
>FQH3XDB01BKRH6|425|1
AACGAACGCTGGCGGCAGGCTTAACACATGCAAGTTGAACGGGATTCAATTGGTGCTTGCACTGATTGATGAGAGTGGCGCACGGGTGAGTAATACGTGGGAACATATCTGACAGAGCGGAATAACGGTTGGAAACGACAGCTAATACCGCATACGCCCTGAGGGGGAAAGATTTATCGCTGACAGGATGGCCCCGCGGCAGATTAGGCAGTTGGTGGGGTAAAGGCCTACCAAACCGACGATCTGTAGCTGGTCTGAGAGGACGATCAGCCACATTGGGACTGAGACACGGCCCAGACTCCTACGGGAGGCAGCAGTGAGGAATATTGGGCAAGTGGGGGGAACCCTGACCCAGCCATGCCGCGTGAGTGAA
>FQH3XDB01BW4CF|426|32
AACGAACGCTGGCGGCAGGCTTAACACATGCAAGTTGAACGGGATTTATATAGTGCTTGCACTATATGATGAGAGTGGCGCACGGGTGAGTAACACATGGGAACATACCCTGAAGTTCGGAACAACAGTTGGAAACGACTGCTAATACCGGATAATACCTGAGGGAGAAAGATTTATCGCTTTAGGAATGGCCCATGGCAGATTAGGTAGTTGGTGGGGTAAAAGCTTACCAAGCCGACGATCTGTAGCTGGTCTGAGAGGACGATCAGCCACACTGGAACTGAGACACGGTCCAGACTCCTACGGGAGGCAGCAGTGAGGAATATTGGGGCAATGGAGGGAACTCTGACCCAGCCATGCCGCGTGAGTGAAGAAGGTTTTCGAATTGTAAAAGCTCTTTCGGATGTGAAGATGATGACGGTAGCATCTAAAGAAGCCCCGGCAAAACTTCGTGCCAGCAGCCG
>FQH3XDB01BMKV1|427|3
AACGAACGCTGGCGGCAGGCTTAACACATGCAAGTTGAACGGGATTTGATTGGTGCTTGCACTGATCAATGAGAGTGGCGCACGGGTGAGTAATACGTGGGAACATATCTGACAGAGCGGAATAACGGTTGGAAACGACAGCTAATACCGCATACGCCCTGAGGGGAAAGATTAATCGCTGACAGGATGGCCCGCGGCAGATTAGGCAGGTTGGTGGGTAAAGGCCTACCAAACCGACGATCTGTAGCTGGTCTGAGAGGACGATC
>FQH3XDB01AY866|428|3
AACGAACGCTGGCGGCAGGCTTAACACATGCAAGTTGAACGGGATTTGTGTGGTGCTTGCACCATATAATGAGAGTAGCGCACTGGTGAGTAACACGTGGGAACGTACCTTTTGGTGGGGGACAACATCTGGAAACGGATGCTAATACCGCATAAGACCTGAGGGTGAAAGATTTATCGCCGAAAGAACGGCCCGCGGAAGATTAGGCAGTTGGTGGGTAAAGGCCTACCAAACCTACGATCTATAGCTGGTCTGAGAGGACGATCAGCCACACTGGAACTGAGACACGGTCCAGACTCCTACGGGAGCAGCAGTGAGGAATATTGGGCAATGGAGGCAACTCTGACCCAGCCATGCCGCGTGAGTGAAGAGGTTTTCGGATTGTAAAGCTCTTCGGATGTGACGATGATGAC
>FQH3XDB01CRJBO|429|4
AACGAACGCTGGCGGCAGGCTTAACACATGCAAGTTGAACGGGAATGGTTAAGTAGCTTGCTATTTAATCATGAGAGTAGCGCACTGGTGAGTAACACGTGGGAATATACCTATTGGTGGGGGACAACAGTTGGAAACGACTGCTAATACCGCATAAGCCCTGAGGGGAAAGATTAATTGCCGATAGATTAGCCCGCGGAAGATTAGGTAGTTGGTGGGGTAAAAGGCCTACCAAGCCTACGATCTATAGCTGGTCTGAGAGGACGATCAGCCACATTGGAACTGAGACACGGTCCAGACTCCTACGGGAGGCAGCAGTGAGGAATATTGGGCAATGGAGGGAACTCTGACCCCAGCCATGCCGCGTGAGTGAGAAGGTCTTCGGATTGTAAAGCTCTTTTGTGCAGGAAGATGATGACAGTACTGA
>FQH3XDB01E0ODG|430|6
AACGAACGCTGGCGGCAGGCTTAACACATGCAAGTTGAACGGTATTGTGTAGTGCTTGCACTATATGAGAGAGTAGCGCACTGGTGAGTAACACGTGGGAACATGCCCTTAAGTAAGGAACAACAGTTGGAAACGACTGCTAATACCATATACGCCCTGAGGGGGAAAGATTTATCGCTTAAGGATTGGCCCGCGGCAGATTAGGTAGTTGGTGGGTAAAGGCCTACCAAGCCGACGATCTATAGCTGGTCTGAGAGGACGATCAGCCACATTGGAACTGAGACACGGTCCAGA
>FQH3XDB01BSL8S|431|1
AACGAACGCTGGCGGCAGGCTTAACACATGCAAGTTGAACGGTATTGTGTAGTGCTTGCACTATATGAGAGAGTAGCGCACTGGTGAGTAACACGTGGGAACATGCCCTTAAGTAAGGAACAACAGTTGGAAACGACTGCTAATACCATATACGCCCTGAGGGGGAAAGATTTATCGCTTAAGGGATTTGGGCCCCGCGGGCAGATTAGGTAGTTGGTGGGGTAAAGGCCTACCAAGCCGACGATCTATAGCTGGTCTGAGAGGACGATCAGCCACATTGGAACTGAGACACGGTCCAGACTCCTACGGGAGGCAGCAGTTGAGGAATATTGGGGCAATGGAGGGAACTCTGACCCAGCCATGCCGCGTGAGTGAAGAAGGTCTTCGGATTGTAAAGCTCTTTCGGATAAGCGATGATGACGG
>FQH3XDB01C6JCB|432|17
AACGAACGCTGGCGGCAGGCTTAACACATGCAAGTTGAACGGTATTTGCTTGTAGCTTGCTATGAGCAAAGAGAGTAGCGCACTGGTGAGTAACACGTGGGAACATGCCTTAAAGTGGGGGACAACAGCTGGAAACGGCTGCTAATACCGCATAAACCCTGAGGGGGAAAGATTTATTGCTTTAAGATTGGCCCCGCGGAAGATTAGGTAGTTGGTGGGGTAAAGGCCTACCAAGCCGACGATCTATAGCTGGTCTGAGAGGACGAACAGCCACATTGGAACTGAGACACGGTCCAGACTCCTACGGGAGCAGCAGTGAGGAATATTGGGGCAATGGAGGAAACTCTGACCCAGCCATGCCGCGTGAGTGAAGAAGGTTTTCGGATTGTAAAGCTCTTTCGGATGTGACGATGATGACGGTAACATCTAAAGAAGCCCCGGCAAACTTCGTGCCAGCAGCCGCGG
>FQH3XDB01CFO05|433|1
AACGAACGCTGGCGGCAGGCTTAACACATGCAAGTTGAACGGTATTTGCTTGTAGCTTGCTATGAGCAAAGAGAGTAGCGCACTGGTGAGTAACACGTGGGAACATGCCTTAAAGTGGGGGACAACAGCTGGAAACGGCTGCTAATACCGCATAAACCCCTGAGGGGGAAAGATTTATTGCTTTAAGATTGGCCCGCGGAAAGATTTAGGGTAGTTGGTGGGGTAAAAGGCCTACCAAGCCGACGATCTATAGCCTGGGTCTGAGAGGACGAACAGCCACATTGGAACTGAGACACGGTTCCAGACTCCTACGGGAGGCAGCAGTGAGGAAT
>FQH3XDB01C15VO|434|2
AACGAACGCTGGCGGCAGGCTTAACACATGCAAGTTGAACGGTATTTGCTTGTAGCTTGCTATGAGCAGAGAGAGTAGCGCACTGGTGAGTAACACGTGGGAACATACCTTGAAGTGGGGAACAACAGTTGGAAACGACTGCTAATACCGCATACGCCCTAAGGGGGAAAGATTTATCGCTTTGAGATTGGCCCGCGGAAAGATTAGGGTAGTTGGTGGGGTAAAAGGCCTACCAAGCCGACGATCTATAGCTGGTCTGAGAGGACGAACAGCCACATTGGAACTGAGACACGGTCCAGACCTCC
>FQH3XDB01CP6CI|435|1
AACGAACGCTGGCGGCAGGCTTAACACATGCAAGTTGAACGGTATTTGCTTGTAGCTTGCTATGAGCAGAGAGAGTAGCGCACTGGTGAGTAACACGTGGGAACATACCTTGAAGTGGGGAACAACAGTTGGAAACGACTGCTAATACCGCATACGCCTAAGGGGAAAGATTTATCGGCTTTGGGGATTGGCCCCGCGGAAAGATTAGGTAGTTGGTGGGGTAAAGCCTACCAAGCCGACGATCTATAGCTGGTCTGAGAGGACGAACAGCCACATTGGAACTGAGACACGGTCCAGACTCCTAGGGAGCAGCAGTGA
>FQH3XDB01EZNX5|436|9
AACGAACGCTGGCGGCAGGCTTAACACATGCAAGTTGAACGGTATTTGCTTGTAGCTTGCTATGAGCAGAGAGAGTAGCGCACTGGTGAGTAACACGTGGGAACATACCTTGAAGTGGGGAACAACAGTTGGAAACGACTGCTAATACCGCATACGCCCTAAGGGGGAAAGATTTATCGCTTTGAGATTGGCCCGCGGAAGATTAGGTAGTTGGTGGGGTAAAGCCTACCAAGCCGACGATCTATAGCTGGTCTGAGAGGACGAACAGCCACATTGGAACTGAGACACGGTCCAGACTCCTACGGGAGCAGCAGTGAGGAATAGTTGGGCAAATGGAAGGAAACTCTGACCCAGCCATGCCGCGTGAGTGAA
>FQH3XDB01BE35L|437|1
AACGAACGCTGGCGGCAGGCTTAACACATGCAAGTTGAACGGTATTTGCTTGTAGCTTGCTATGAGCAGAGAGAGTAGCGCACTGGTGAGTAACACGTGGGGACATACCTTGAAGTGGGGAACAACAGTTGGAAACGACTGCTAATACCGCATACGCCCGTAAGGGGAAAGATTTATCGCTTTGAGATTGGCCCGCGGAAGATTAGGTAGTTGGTGGGTAAGCCTACAGCCGACGATCTATAGCTGGTCTGAGAGGACGAAC
>FQH3XDB01B2W2X|438|2
AACGAACGCTGGCGGCAGGCTTAACACATGCAAGTTGAACGGTATTTGCTTGTAGCTTGCTATGAGCAAAGAGAGTAGCGCACTGGTGAGTAACACGTGGGAACATGCCTTAAAGTGGGGGACAACAGCTGGAAACGGCTGCTAATACCGCATAACGTATCGAGGAGGCATCTCCATGATACCAAAGATTATCGCTGAAAGATGGGCTCGCGTACGATTAGATAGTTGGTGAGGTAACGGCTCACCAAGTCGACGATCGTTAGCCGGACT
>FQH3XDB01C3LCI|439|20
AACGAACGCTGGCGGCAGGCTTAACACATGCAAGTTGAACGTGATTTGTTTGGTGCTTGCACCGAGCAATGAAAGTAGCGCACTGGTGAGTAACACGTGGGAACGTACCTTTGGTGGGGAACAACAGTTGGAAACGACTGCTAATACCGCATAAGCCCTGAGGGGAAAGATTTATCGCCGAAAGAACGGCCCGCGGAAGATTAGGTAGTTGGTGGGGTAACGGCCTACCAAGCCGACGATCTATAGCTGGTCTGAGAGGACGATCAGCCACATTGGAACTGAGACACGGTCCAAACTCCTACGGGAGGCAGCAGTGAGGAATATTGGGCAATGGAGGCAACTCTGACCCAGCCATGCCGCGTGAGTGAAGAAGGTTTTCGGATTGTAAAGCTCGTTTCGGGTGTGAC
>FQH3XDB01EWAEU|440|8
AACGAACGCTGGCGGCAGGCTTAACACATGCAAGTTGAGCGGGAATGATTGATAGCTTGCTATTGATTATGAGAGCAGCGCACTGGTGAGTAACACGTGGGAATATACCTATTGGTGGGGGACAACAATTGGAAACGATTGCTAATACCGCATAAGCCCTGAGGGGGAAAGATTTATTGCCGATAGATTAGCCCGCGGAAGATTAGGTAGTTGGTGGGTAACGGCCTACCAAGCCGACGATCTATAGCTGGTCTGAGAGGACGATCAGCCACATTGGAACTGAGACACGGTCCAGACTCCTACGGGAGCAGCAGTGAGGAATATTGGGCAATGGAGGCAACTCTGACCCAGCCATGCCGCGTGAGTGAA
>FQH3XDB01B9LUZ|441|3
AACGAACGCTGGCGGCAGGCTTAACACATGCAAGTTGAGCGGGAATGATTGATAGCTTGCTATTGATTATGAGAGCAGCGCACTGGTGAGTAACACGTGGGAATATACCTATTGGGTGGGGGACAACAATTGGAAACGATTGCTAATACCGCATAAGCCCTGGAGGGGGAAAGATTTATTGCCGATAGATTAGCCCGCGGAAGATTAGGTAGTTGGTGGGGTAACGGCCTACCAAGCCGACGATCTATAGCTGGTCTGAGAGGACGATCAGCCACATTGGAACTGAGACACGGTCCAGACTCCTACGGGAGCAGCAGTGAGGAATATTGGGGCAAT
>FQH3XDB01BBCDT|442|12
AACGAACGCTGGCGGCAGGCTTAACACATGCAAGTTGAGCGGGATTGATTGGTAGCTTGCTACTGATCATGAGAGCAGCGCACTGGTGAGTAACACGTGGGAATATACCTATTGGTGGGGGACAACAATTGGAAACGATTGCTAATACCGCATAAGCCCTGAGGGGAAAGATTTATTGCCGATAGATTAGCCCGCGGAAGATTAGGTAGTTGGTGGGTAACGGCCTACCAAGCCGACGATCTATAGCTGGTCTGAGAGGACGATCAGCCACATTGGAACTGAGACACGGTCCAGACTCCTACGGGAGGCAGCAGTGAGGAATATTGGGCAATGGAGGAAACTCTGACCCAGCCATGCCGCGTGAGTGAAGAGGTTTTCGGATTGTAAAGCTCTTTCGGACAGGAAGATAATGACGGTACTGTCTAAAGAAGCCCCGGCTAACTTCGTGCCAGCAGCCGCGG
>FQH3XDB01BGSTY|443|1
AACGAACGCTGGCGGCAGGCTTAACACATGCAAGTTGAGCGGGATTGATTGGTAGCTTGCTACTGATCATGAGAGCAGCGCACTGGTGAGTAACACGTGGGAATATACCTATTGGTGGGGACAACAATTGGAAACGATTGCTAATACCGCATAAGCCCTGAGGGGAAAGATTTATTGCCGATAGATTAGCCCGCGGAAGATTAGGTAGTTGGTGAGGTAACGGCCCACCAAGCCGACGATCAGTAGCCGGTCTGAGAGGATGAACGGCCACACTGGAACTGAGACACGGTCCAGACTCCTACGGAGGCAGCAGTGGGAATCTTCCGCAATGGGCGAAAGCCTGACGGAGCAACGCCGCGTGAGTGATGACGGCCTTCGGGTTGTAAAGCTCTGTGAGTCGGGACGAAT
>FQH3XDB01D099E|444|21
AACGAACGCTGGCGGCAGGCTTAACACATGCAAGTTGGACGGGAGTTGATTGAATGCTTGCATTTGATCAACGAGAGTAGCGCACGGGTGAGTAACACGTGGGAACATACCCACAGGAGGGGGATAACAGTTGGAAACGACTGATAATACCGCATAAGCCGTATGGAGACATACGGGAAAGGATGGAGACATCGCCTGAGGATTGGCCCCGCGGAAGATTAGGTAGTTGGTGGGGTAAAGGCCTACCAAGCCTACGATCTATAGCTGGTCTGAGAGGACGATCAGCCACATTGGGACTGAGACACGGCCCAAAACTCCTACGGGAGGCAGCAGTGAGGAATATTGGGCAAGTGGGGCAACCCTGACCCAGCCATGCCGCGTGAGTGAA
>FQH3XDB01ARGHR|445|1
AACGAACGCTGGCGGCAGGCTTAACACATGCAAGTTGGACGGGAGTTGATTGAATGCTTGCATTTGATCAACGAGAGTAGCGCACGGGTGAGTAACACGTGGGAACATACCCCACAGGAGGGGGATAACAGTTGGAAACGACTGATAATACCGCATAAGCCGTATGGAGGCATACGGGAAAGGATGGAGACATCGCCTGAGGATTGGCCCCGCGGAAAGATTAGGTAGTTGGTGGGGTAAAGGCCTACCAAGCCTACGATCTATAGCTGGTCTGAGAGGACGATCAGCCACATTGGGACTGAGACACGGCCCAAACTCCTACGGGAGGCAGCAGTGAGGAATATTGGGCAATGGGGGCAACCCTGACCCAGCCATGCCGCGTGAGTGAAG
>FQH3XDB01DMHLJ|446|1
AACGAACGCTGGCGGCAGGCTTAACACATGCAAGTTGGACGGGAGTTGATTGAATGCTTGCATTTGATCAACGAGAGTAGCGCACGGGTGAGTAACACGTGGGAACATACCCCACAGGAGGGGGATAACAGTTGGAAACGACTGATAATACCGCATAAGCCGTATGGAGACATACGGGAAAGGATGGAGACATCGCCTGAGGATTGGCCCGAGGAAGATTAGGTAGGTTGGGTAGGGGTAAACGGCCTACCAAGCCTACGATCTATA
>FQH3XDB01C4B0G|447|1
AACGAACGCTGGCGGCAGGCTTAACACATGCAAGTTGGACGGGAGTTGATTGAATGCTTGCATTTGATCAACGAGAGTAGCGCACGGGTGAGTAACACGTGAGCAACCTGCCTTTAAGAGAGGGATAGCTTCTGGAAACGGATGGTAATACCTCATAAAATATGTTTATCGCATGGTAGACATATCAAAGATTTATCGCTTAGAGATGGGCTCGCGTCTGATTAGATAGTTGGTGGGTAACGGCTCACCAAGTCGACGATCAGTAGCCGGACTGAGAGGTTGATCGGCCACATTGGGACTGAGACACGG
>FQH3XDB01APDR3|448|1
AACGAACGCTGGCGGCAGGCTTAACACATGCACGTTGAACGTGATTTGTTTGGTGCTTGCACCGAGCAATGAAAGTAGCGCACTGGTGAGTAACACGTGGGAACGTACCTTTTGGTGGGGAACAACAGTTGGAAACGACTGCTAATACCGCATAAGCCCTGAGGGGAAGATTATCGCCGAAGAACGGCCGCGGAAGATTAGGTAGTTGGTGGGTAACGGCCGTACCAAGCCGACGATCTATAGCTGGTCTGAGAGGACGATCAGCCACATTGGAACTGAGACACGGTCCAAACTCCTACGGGAGGCAGCAGTGAGGAATATT
>FQH3XDB01CA9JD|449|1
AACGAACGCTGGCGGCAGGCTTAACACATGCACGTTGAACGTGATTTGTTTGGTGCTTGCACCGAGCAATGAAAGTAGCGCACTGGTGAGTAACACGTGGGAACGTACCTTTTGGTGGGGAACAACAGTTGGAAACGACTGCTAATACCGCATAAGCCCTGAGGGGAAAGATTTATCGCCGAAAAGAACGGCCCGCGGAAAGATTTAGGTAGTTGGTGGGGTAAACGGCCTACCAAGCCGACGATCTATAGCTGGTCTGAGAGGACGATCAGCCACATTGGAACTGAGACACGGTCCAAAACTCCTACGGGAGGCAGCAGTGAGGAATATTGGGCAATGGAGGCAACTCTGACCCAGCCATGCCGCGTGAGTGAAGAAGGTTTTCGGATTGTAAAAGCTCTTTCGGGTGTGACGATGATGACGGTAGCACCTAAAGAAGCCCCGGCTAACTTCGTGCCAGCAGCCGCGGTAAATGTATACTCGGTCG
>FQH3XDB01EMV9H|450|2
AACGAACGCTGGCGGCAGGCTTAACACATGCAAGTCGAACGGAATACAGGGAGCTTGCTTTCTGTATCAGTGGCGCACGGGTGAGTAACGCGTAGGTATCTGTCTATGGGAGTTGGATAGCTTCTGGAAACGGAAGGTAAAACAGCATAAGCCCTGCGGGGGAAAGTGAGAAATCAGCCGATAGAGGAGCCTGCGTTAGATTAGGCAGTTGGTGGGTAACGGCCTACCAAACCGACGATCTATAGCTGGTCTGAGAGGATGATCAGCCACATTGGGACTGAGACACGGCCCAAACTCCTACGGGAGGCAGCAGTGGGGAATATTGGACAATGGGGGAGACCCTGATCCAGCCATGCCGCGTGAGTGAAGAAGGCCTTCGGGTTGTAAAGCTCTTTACATGGGAAGATAATGACGGT
>FQH3XDB01B0SNG|451|2
AACGAACGCTGGCGGCAGGCTTAACACATGCAAGTTGAACGGGAGTTAGGATTGCTTTGCAATACTAACGAGAGTGGCGCACGGGTGAGTAATACGTGGGAACATACCTATTGGTACGGAATAACGGTTGGAAACGACAGCTAATACCGTATATACCCTGAGGGGGAAAGATTTATCGCCGATAGCATGGCCCGCGGCAGATTAGGCAGTTGGTGGGGTAAAAGACCTACCAAAACCGACGATCTGTAGCTGGTCTGAGAGGACGATCAGCCACATTGGGACTGAGACACGGCCCAGACTCCTAC
>FQH3XDB01C19N9|452|10
AACGAACGCTGGCGGCAGGTTTGAGGCATGCAAGTCGAACGAGAAAGTGTAGCAATACACGAGTAGAGTGGCGCACGGGTGAGTAATATATGGGGATCTGCCCAGAAGTGGGGAATAGCAGTTGGAAACGACTGCTAATACCGCATAAGCCGGAAACGGGAAAGATTTATCGCTTTTGGATGAGCCCATATTGGATTAGCTAGTTGGTAGGGTAAAGGCCTACCAAGGCGATGATCCATAGCTGATTTGAGAGAATGATCAGCCACACTGGAACTGAGACACGGTCCAGACTCCTACGGGAGGCAGCAGCTAAGAATATTGGACAATGGGCGCAAGCCTGATCCAGCCATGCCGCGTGAGTGAAGAAGGCCTTGGGTTGTAAAGCTCTTTTAATAGGGAAGATGATGACGGTACCTATAGAATAAGCACCGGCTAACTTCGTGCCAG
>FQH3XDB01E3A2E|453|1
AACGAACGCTGGCGGCATGCCTAACACATGCAAGTCGAACGAGATCTTCGGATCTAGTGGCGCACGGGTGCGTAACGCGTGGGAATCTGCCCTTTGCTTCGGAATAACAGTGAGAAATTACTGCTAATACCGGATGATGTCTTCGGACCAAAGATTTATCGGCAAAGGATGAGCCCGCGTAGGATTAGGTAGTTGGTGGGGTAACTGGCCTACCAAGCCGACGATCCTTAGCTGGTCTGAGAGGATGATCAGCCACACTGGGACTGAGACACGGCCCTAGACTCCTACGGGAGGCAGCAGTGGGG
>FQH3XDB01D4TPO|454|1
AACGAACGCTGGCGGCATGCCTAACACATGCAAGTCGAACGGGATCCAGGCTTCGGCCTGGTGAGAGTGGCGGACGGGTGAGTAACGCGTGGGAACCTGCCCAATGGTCTGGGATAACCGCGGGAAACTGCGGCTAATACCGGATAAGCCCGAGAGGGGAAAGATTTATCGCCGATGGAGGGCCCGCGTCCGATTAGGTAGTTGGTGAGGTAACGGCTCACCAAGCCTACGATCGGTAGCTGGTCTGAGAGGATGATCAGCCACACTGGGACTGAGATACGGCCCAGACTCCTACGGAGGCAGCAGTGGGGAATATTGGACAATGGGGGCAACCCTGATCCAGCAATGCCGCGTGAGTGATGAAGGCCTTAGGGTTGTAAAGCTCTTTTGGCGGGACGATGATGACGGTACCGCA
>FQH3XDB01A4Z4V|455|1
AACGAACGCTGGCGGCATGCTTAACACATGCAAGTCGAACGATGATATATAATACTTGTATTATATAACATAGTGGCAAACGGGTGAGTAACGCGTGGGAATGTACCCTTCAGTAATGGATAACGATTGGAAACGATCGCTAATACAATATATAGTTGAGAGACGAAAGAGAGATCGCTGAGGGAGCGGCCCGCGTTAGATTAGGTAGTTGGTGGGGTAAAGCCTACCAAGCCGATGATCTATAGCTGGTCTGAGAGGATGATCAGCCACATTGGGACTGAGACACGGCCCAGACTCCTACGGGAGGCAGCAGTGGGGAATATTGGACAAGTGGGGAAACCCTGATCCAGCGATGCCGCGTGAG
>FQH3XDB01ASXKP|456|9
AACGAACGCTGGCGGCATGCTTAACACATGCAAGTCGAACGGAACATATAGAGCTTGCTTTATATGTCAGTGGCGCACGGGTGAGTAACGCGTGGGAATGTATCTTTTGTTTGGGATAGCTTCTGGAAACGGAAGGTAATACCGGATGAGCCCTGAGGGGGAAAGTAGTGATACGCAAAGAGAGCGGCCCGCGTTAGATTAGGCAGTTGGTGGGTAACGGCCTACCAAACCGACGATCTATAGCTGGTCTGAGAGGATGATCAGCCACATTGGGACTGAGACACGGCCCAAAACTCCTACGGGAGGCAGCAGTGGGGAATATTGGACAATGGGGGAAACCCTGATCCAGCAATGCCGCGTGGGTGAGAAGGCCTTCGGGTTGTAAAGCTCTTTTAGATGGAAGATAATGACGGTACCATCAGAATAAGCACCGGCTAACTTCGTGCCAGCAGCCGC
>FQH3XDB01CHUU1|457|1
AACGAACGCTGGCGGCATGCTTAACACATGCAAGTCGAACGGAACATATAGAGCTTGCTTTATATGTCAGTGGCGCACGGGTGAGTAACGCGTGGGAATGTATCTTTTGTTTGGGATAGCTTCTGGAAACGGAAGGTAATACCGGATGAGCCCTGAGGGGAAAGTAGTGATACGCAAAGAGAGCGGCCCGCGTTAGATTAGGCAGTTGTGGGTAACGCTACCAAACCGACGATCTATAGCTGGTCTGAGAGGATGATCAGCCACATTGGGACTGAGACACGGCCCAAACTCCTACGGAGGCAGCAGTGGGGAATATTGGACAATGGGGAAACCCTGATCCAGCAATGCCGCGTGAGTGAGAAGGCCTTCGGGTTGTAAAGCTCTTTTAGA
>FQH3XDB01EGZWE|458|1
AACGAACGCTGGCGGCATGCTTAACACATGCAAGTCGAACGGAACATAAGGTGCTTGCATCTTATGTCAGTGGCGCACGGGTGAGTAACGCGTGGGAATGTATCTTTTGTTGGGATAGCTTCTGGAAACGGAAGGTAATACCGGATGAGCCCTGAGGGGGAAAGTAGTGATACGCAAAGAGAGCGGCCCGCGTTAGATTAGGCAGTTGGTGGGGTAACGCCTACCAAACCGCGATCTATAGCTGGTCTGAGAGGATGATCAGCCACATTGGGACTGAGACACGGCCCAAAACTCCTACGGGAGGCAGCAGTGGGGAATATTGGACAATGGGGGAACCCTGATCCAGCAATGCCGCGTGAGTGAGAAGGCCTTGGGTTGTAAAGCTCTTTTAGATGGGAAGATAATGACGGTACCATCAGAATAA
>FQH3XDB01A0OWG|459|5
AACGAACGCTGGCGGCATGCTTAACACATGCAAGTCGAACGGAACATAAGGTGCTTGCATCTTATGTCAGTGGCGCACGGGTGAGTAACGCGTGGGAATGTATCTTTTTGTTTGGGATAGCTTCTGGAAACGGAAGGTAATACCGGATGAGCCCTGAGGGGGAAAGTAGTGATACGCAAAGAGAGCGGCCCGCGTTAGATTAGGCAGTTGGTGGGGTAACGGCCTACCAAACCGACGATCTATAGCTGGTCTGAGAGGATGATCAGCCACATTGGGACTGAGACACGGCCCAAACTCCTACGGGAGGCAGCAGTGGGGAATATTGGACAAGTGGGGGGAACCCTGATCCAGCAATGCCGCGTGAGTGAGAAGGCCTTCGGTATGTAAAGCTCTATCAGC
>FQH3XDB01CCDET|460|2
AACGAACGCTGGCGGCATGCTTAACACATGCAAGTCGAACGGAACATAAGGTGCTTGCATCTTATGTCAGTGGCGCACGGGTGAGTAACGCGTGGGAATGTATCTTTTTGCTTGGGATAGCTTCTGGAAACGGAAGGTAATACCGGATGAGCCCTGAGGGGGAAAGTAGTGATACGCAAAGAGAGCGGCCCGCGTTAGATTAGGCAGTTGGCGGGGTAACGCCTACCAAACCGACGATCTATAGCTGGTCTGAGAGGATGATCAGCCACATTGGGACTGAGACACGGCCCAAAACTCCTACGGGAGGCAGCAGTGGGGAATATTGGACAATGGGGGAAAACCCTGATCCAGCAATGCC
>FQH3XDB01A4RV9|461|1
AACGAACGCTGGCGGCATGCTTAACACATGCAAGTCGAACGGAACATATAGAGCTTGCTTTATATGTCAGTGGCGCACGGGTGAGTAACGCGTGGGAATGTATCTTTTGTTGGGATAGCTTCTGGAAACGGAGGTAATACCGGATGAGCCCTGAGGGGGAAAGTAGTGATACGCAAAGAGAGCGGCCCGCGTTAGATTAGGCAGTTGGTGGGTAACGCCTACCAAACCGACGATCTATAGCTGGTCTGAGAGGATGATCAGCCACATTGGGACTGAGACACGGCCCAAAACTCCTACGGGAGGCAGCAGTGGGAATATTGGACAATGGGGAAACCCTGATCCAGCAATGCCGCGTGGGTGAGAA
>FQH3XDB01B87VR|462|2
AACGAACGCTGGCGGCATGCTTAACACATGCAAGTCGAACGGAACATATAGAGCTTGCTTTATATGTCAGTGGCGCACGGGTGAGTAACGCGTGGGAATGTATCTTTTTGTTTGGGATAGCTTCTGGAAACGGAAGGTAATACCGGATGAGCCCTGAGGGGGAAAGTAGTGATACGCAAAGAGAGCGGCCCGCGTTAGATTAGGCAGTTGGTGGGGTAACGGCCTACCAAACCGCGATCTATAGCTGGTCTGAGAGGATGATCAGCCACATTGGGACTGAGACACGGCCCAAAACTCCTACGGGAGGCAGCAGTGGGGAATATTGGACAAGTGGGGAAACCCTGATCCAGCAATGCCGCGTGAGTGAGAAGG
>FQH3XDB01D5PZK|463|2
AACGAACGCTGGCGGCATGCTTAACACATGCAAGTCGAACGGAACATATAGAGCTTGCTTTATATGTCAGTGGCGCACGGGTGAGTAACGCGTGGGAATGTATCTTTTGTTTGGGATAGCTTCTGGAAAACGGAAGGTAATACCGGATGAGCCCTGAGGGGGAAAGTAGTGATACGCAAAGAGAGCGGCCCGCGTTAGATTAGGCAGTTGGTGGGTAACGCCTACCAAACCGACGATCTATAGCTGGTCTGAGGGGATGATCAGCCACATTGGGGACTGAGACACGGCCCCAAAACTCCTACGGGAGGCAGCAGTGGGGAATATTGGACAATGGGGGAAACCCTGATCCAGCAATGCCGCGTGAGTGAGAAGGCC
>FQH3XDB01DU24S|464|1
AACGAACGCTGGCGGCATGCTTAACACATGCAAGTCGAACGGAACATATAGAGCTTGCTTTATATGTCAGTGGCGCACGGGTGAGTAACGCGTGGGAATGTATCTTTTTGTTTGGGATAGCTTCTGGAAACGGAAGGTAATACCGGATGAGCCCCTGAGGGGGAAAGTAGTGATACGCAAAGAGAGCGGCCCGCGTTAGATTAGGCAGTTGGTGGGGTAACGGCCTACCAAACCGACGATCTATAGCTGGTCTGAGAGGATGATCAGCCACATTGGGACTGAGACACGGCCCAAAACTCCTACGGGAGGCAGCAGTGGGGAATATTGGACAATGGGGGAAACCCTGATCCAGCAATGCCGCGTGAGTGAGAAGG
>FQH3XDB01B7TCW|465|1
AACGAACGCTGGCGGCATGCTTAACACATGCAAGTCGAACGGAACATAAGGTGCTTGCATCTTATGTCAGTGGCGCACGGGTGAGTAACGCGTGGGAATGTATCTTTTGTTGGGATAGCTTCTGGAAACGGAAGGTAATACCGGATGAGCCCTGAGGGGGAAAGTAGTGATACGCAAAGAGAGCGGCCCGCGTTAGATTAGGCAGTTGGTGGGGTAACGGCCTACCAAACCGACGATCTATAGCTGGTCTGAGAGGATGATCAGCCACATTGGGACTGAGACACGGCCCAAAACTCCTACGGGAGGCAGCAGTGGGGAATATTGGGACAATGGGGGGAACCCTGATCCAGCAATGCCGCG
>FQH3XDB01DJRW1|466|1
AACGAACGCTGGCGGCATGCTTAACACATGCAAGTCGAACGGAACATATAGAGCTTGCTTTATATGTCAGTGGCGCACGGGTGAGTAACGCGTGGGAATGTATCTTTTTGTTTGGGATAGCTTCTGGAAACGGAAGGTAATACCGGATGAGCCCTGAGGGGAAGTAGTGATACGCAAAGAGAGCGGCCCGCGTTAGATTAGGCAGTTGGTGGGTAACGGCCTACCAAACCGACGATCTATAGCTGGTCTGAGAGGATGATCAGCCACATTGGGACTGAGACACGGCCCAAACTCCGTACGGG
>FQH3XDB01D8BDS|467|1
AACGAACGCTGGCGGCATGCTTAACACATGCAAGTCGAACGGAACATATAGAGCTTGCTTTATATGTCAGTGGCGCACGGGTGAGTAACGCGTGGGAATGTATCTTTTTGTTTGGGATAGCTTCTGGAAACGGAAGGTAATACCGGATGAGCCCTGAGGGGGAAATAGTGATACGCAAAGAGAGCGGCCCGCGTTAGATTAGGCAGTTGGTGGGGTAACGGCCTACCAACCGACGATCTATAGCTGGTCTGAGAGGATGATCAGCCTACATTGGGACTG
>FQH3XDB01BT7BX|468|3
AACGAACGCTGGCGGCATGCTTAACACATGCAAGTCGAACGGAACATAAGGTGCTTGCATCTTATGTCAGTGGCGCACGGGTGAGTAACGCGTGGGAATGTATCTTTTGTTGGGATAGCTTCTGGAAACGGAAGGTAATACCGGATGAGCCCTGAGGGGGAAAGTAGTGATACGCAAAGAGAGCGGCCCGCGTTAGATTAGGCAGTTGGTGGGTAACGGCCTACAAACCGCGATCTATAGCTGGTCTGAGAGGATGATCAGCCACATTGGGACTGAGACACGGCCCAAACTCCTACGGGAGCAGCAGTGGGAAT
>FQH3XDB01CR6JN|469|1
AACGAACGCTGGCGGCATGCTTAACACATGCAAGTCGAACGGAACATAAGGTGCTTGCATCTTATGTCAGTGGCGCACGGGTGAGTAACGCGTGGGAATGTATCTTTTGTTTGGGATAGCTTCTGGAACGGAAGGTAATACCGGATGAGCCCTGAGGGGAAGTAGTGATACGCAAGAGAGCGGCCGCGTTAGATTAGGCGAGTTGGTGGGGTAACGGCCTACCAACCGACGATCTATAGCTGGTCTGAGAGGATGATCAGCCACATTGGGACTGAGAC
>FQH3XDB01DDR41|470|1
AACGAACGCTGGCGGCATGCTTAACACATGCAAGTCGAACGGAACATAAGGTGCTTGCATCTTATGTCAGTGGCGCACGGGTGAGTAACGCGTGGGAATGTATCTTTTTGTTTGGGATAGCTTCTGGAAAACGGAAGGTAATACCGGATGAGCCCTGAGGGGGAAAGTAGTGATACGCAAAAGAGAGCGGCCCGCGTTAGATTAGGCAGTTGGTGGGGTAACGGCCTACCAAAACCGACGATCTATAGCTGGTCTGAGAGGATGATCAGCCACATTGGGACTGAGACACGGCCCAAACTCCTACGGGAGGCAGCAGTGGGGAATATTGGACAAGTGGGGGAACCCTGATCCAGCAATGCCGCGTGAGTGAGAAGG
>FQH3XDB01C7HQ7|471|1
AACGAACGCTGGCGGCATGCTTAACACATGCAAGTCGAACGGAACATATAGAGCTTGCTTTATATGTCAGTGGCGCACGGGTGAGTAACGCGTGGGAATGTATCTTTTTGTTTGGGATAGCTTCTGGAAACGGAAGGTAATACCGGATGAGCCCTGAGGGGGAAAGTAGTGATACGCAAAGAGAGCGGCCCGCGTTAGATTAGGCAGTTGGTGGGTAACGGCCTACCAAACCGACGATCTATAGCTGGTCTGAGAGGATGATCAGCCACATTGGGACTGAGACACGGCCCAAACTCC
>FQH3XDB01B4ZLM|472|1
AACGAACGCTGGCGGCATGGATTAGGCATGCAAGTCGAACGCCGGTAGCAATACCGGAGTGGCGCAAGGGTGAGGAACGCGTGAGTGATCTGCCCTCAAGTTGGGAATAGCTCCTGGAAACGGGAATTAATACCGAATGTGATCATAGAGCTGCATGGTTTTATGATTAAAGGCGAAAGTCGCTTGAGGAGGAGCTCGCGTCCCATTAGCTAGTTGGTGAAGTAACGGCCCACCAAGGCAATGATGGGTAGCTGGTCTGAGAGGACGACCAGCCACACTGGGACTGAGAACTACCCAGACTCCTACGGGAGGCTGCAGTCGAGAATTTTGGGCAATGGGCGAAAGCCTGACCCAGCAATGCCGCGTGCGGGATGAAGGTCTTCGGATTGTAAACCGC
>FQH3XDB01CQGN9|473|18
AACGAACGCTGGCGGCATGGATTAGGCATGCAAGTCGAACGGTGCAGCAATGCACAGTGGCGCAAGGGTGAGGAACGCGTGAGTAACCTGCCCTTAAGTTGGGAATAGCTCCTGGAAACGGGAATTAATACCGAATGTGATCGTTGGAAGACATCTTCTGGTGATTAAAGGCCGCAAGGTCGCTTAAGGAGGGGCTCGCGTCCCATTAGCTAGTTGGTGAGGTAACGGCCACCAAGGCGAAGATGGGTAGCTGGTCTGAGAGGACGATCAGCCACACTGGGACTGAGATACTGCCCAGACTCCTACGGGAGGCTGCAGTCGAGAATTTTGGGCAAGTGGGGCAA
>FQH3XDB01EFKHR|474|1
AACGAACGCTGGCGGCATGGATTAGGCATGCAAGTCGAACGGTGCAGCAATGCACAGTGGCGCAAGGGTGAGGAACGCGTGAGTAACCTGCCCTTAAGTTGGGAATAGCTCCTGGAAACGGGAATTAATACCGAATGTGATCGTTGGAAGACATCTTCTGGTGATTAAAGGCCGCAAGGTTCGCTTAAGGAGGGGCTCGCGTCCCATTAGCTAGTTGGTGAGGTAACGGCCACCAAGGCGAAGATGGGTAGCTGGTCTGAGAGGACGATCAGCCACACTGGGACTGAGATACTGCCCAGACTCCTAC
>FQH3XDB01ARMOV|475|1
AACGAACGCTGGCGGCATGGATTAGGCATGCAAGTCGAACGGTGCAGCAATGCACAGTGGCGCAAGGGGTGAGGAACGCGTGAGTAACCTGCCCCTTAAGTTGGGAATGCTCCTGGAAACGGGATTAATCCGAATGTGATCGTTGGAAGACATCTTCTGATGATTAAAGGCCGCAAGGTTCGCTTAAAGGAGGGGCTCGCGTCCCATTAGCCTAGTTGGTGAGGTAAACGGCCCACCAAGGCGAAGATGGGTAGCTGGGTCTGAG
>FQH3XDB01BF99S|476|7
AACGAACGCTGGCGGCATGGATTAGGCATGCAAGTCGAACGGTGTCAGCAATGACATAGTGGCGCAAGGGTGAGGAACGCGTGAGTGATCTGCCCTCAAGTTTGGAACAGCTCCTGGAAACGGGAATTAATACCGGATGTGATTAAGATGCTGCATGGTATCTTAATTAAAGCAGCAATGCGCTTGAGGAGGGGCTCGCGTCCATTAGTTAGTTGGTGAGGTAATGGCTCACCAAGACGATGATGGGTAGCTGGTCTGAGAGGACGACCAGCCA
>FQH3XDB01DOXKQ|477|1
AACGAACGCTGGCGGCATGGATTAGGCATGCAAGTCGAACGGTGTCAGCAATGACATAGTGGCGCAAGGGGTGAGGAACGCGTGAGTGATCTGCCCTCAAGTTTGGAACAGCTCCTGAAAAACGGGGAATTAATACCGGATGTGATTAAGATGCTGCATGGTATCTTAATTAAAGCAGCAATGCGCTTGAGGAGGGGCTCGCGTCCCATTAGTTAGTTGGTGAGGGTAATGGCTCACCAAGACGATGATGGGTAGCTGGGTCTGAGAGGACGACCAGCCACACTGGGACTGAGAACTGCCCAGGACTCCTACGGGAGGCTGCAGTCGAGAATTTTGGGCAATGGACGAAAGTCTGACCCAGCAATGC
>FQH3XDB01CW6ZY|478|6
AACGAACGCTGGCGGCATGGATTAGGCATGCAAGTTGAACGAACCACTTCGGTGGGGAGTAGCGGAAGGGTGAGGAACGCGTGAGTAATCTGCCCCCTAGTTGGGAATAACAGTTGGAAACGACTGCTAATACCGAATGTGGCTTCAATCCTGCATGGGATGGAGGCTAAAGATTTATCGCTAGGGGATGAGCTCGCGTCCCATTAGGTAGTTGGTGAGGTAACGGCCCACCAAGCCGACGATGGGTAGCTGGTCTGAGAGGATGGTCAGCCACACTGGGACTGAGACACTGCCCAGACTCCTACGGGAGCTGCAGTCGAGAATCTTGGGCAATGCGCGAAAGCGTGACCCAGCAATGCCGCGTGTGTGATGAACGG
>FQH3XDB01BEDKH|479|8
AACGAACGCTGGCGGCATGGATTAGGCATGCAAGTTGAACGAACTCCTTCGGGAGTGAGTAGCGAAAGGGTGAGGAACGCGTGAGTAATCTGCCCATAAGTTGGGAACAACAGTTGGAAACGACTGCTAATACCGAATGTGGCTTTTGGTCCGCATGGATCTTAGGCTAAAGATTTATCGCTTATGGATGAGCTCGCGTCCCATTAGCTTGTTAGTGAGGTAACGGCTCACCAAGGCTTCGATGGGTAGCTGGTCTGAGAGGATGGTCAGCCACACTGGGACTGAGACACTGCCCAGACTCCTACGGGAGGCTGCAGTCGAGAATCTTGGGCAATGCGCGAAAGCGTGACCCAGCAATGCCGCGTGCATGATGAAGGCCTTCGGGTCGTAAAAT
>FQH3XDB01BRQ6U|480|40
GAGAAACGCTGGCGGCGCACATAAGACATGCAAGTCGAACGGAGTTTAGTTGAATGTTTACATGAGACGAAGACTTAGTGGCGGACTGGTGAGTAACACGTGAGCAACCTGCCTCTAAGAGGGGAATAACAACGAGAAATCGTTGCTAATACCGCATAATGCAAAATCCACCGCATGATGGAATTGCCAAAGGAGAAATCCGCTTAGAGATGGGCTCGCGGCTGATTAGTTAGTTGGTGGGGTAATGGCCTACCAAGACGATGATCAGTAGCCGAGCTGAGAGGCTCTACGGCCACATTGGGACTGAGATACGGCCCAGACTCCTACGGGAGCAGCAGTCGGGAATATTGCACAATGGAGG
>FQH3XDB01B1PHV|481|1
AACGAACGCTGGCGGCGCGCCTTAAGCATGCAAGTCGAGCGGCAAGATTGGGGCTTGCTCCAATCCCAGAGCGGCGGACTGGTGAGTAACACGTAGGCGACGTGCCCTTGGGACGGGGATAGCCTGTGGAAACACAGGGTAATACCGGATAAGGCCGTATGCGTTGGAGGCAGTACGGGGAAAGGAGCCATGGCTTCGACCGAGGATCGACCTGCGGCCCATCAGCTAGTTGGCGGGGTAAGGCCCACAGGCAATGACGGGTATCCGG
>FQH3XDB01DV0JF|482|2
AACGAACGCTGGCGGCGCGTCTTAAGCATGCAAGTCGGGCGGCAAGATTGGAGCTTGCTCCAATCCCAGAGCGGCGGACTGGTGAGTAACACGTGGGCGACGCACCCCCGGGACGGGGACAGCCTGTGGAAACACAGGGGTAATACCGGATAAGGCCGCATGCGCCGGAGGCATGCGGGGAAAGGAGCTTCGGCTCCGGCCCGGGGAGCGGCCTGCGGCCCATCAGCTGGTTGGCGGGGTAAGGGCCCACCAAGGCGACGACGGGTATCCGGCCTGAGAGGGTGAGCGGACACATTGGGACTGAGATACGGCCCAGACTCCTACGGGAGGCAGCAGCTAAGGATATTCCGCAAGTGGGGGAACCCTGACGGAGCGACGCCGCG
>FQH3XDB01D8E82|483|9
AACGAACGCTGGCGGCGCGTCTTAAGCATGCAAGTCGGGCGGCAAGATTGGGGCTTGCTCCAATCCCAGAGCGGCGGACTGGTGAGTAACACGTAGGCGACGTGCCCTTGTGACGGGGATAGCCTGTGGAAACACAGGGGTAATACCGGATAAGGCCGTATGCGCTGGAGGCATACGGGGAAAGGAGCCATGGCTTCGCACAGGGAGCGACCTGCGGCCCATCAGCTAGTTGACGGGGCAAGGGCCCACCAAGGCAATGACGGGTATCCGGCCTGAGAGGGTGAACGGACACATTGGGACTGAGATACGGCCCAGACTCCTACGGGAGGCAGCAGCTAAGAATATTCCGCAATGCGCGAAAGCGTGACGGAGCGACGCCGCGTGGACGATGGAGGCCGGAAGGCTGTAAAGTCCTTTAAGGCAGAGGAATAAGGCGGGCAGGGAATGGCCTGCCGGTGACTGTAAGCTTT
>FQH3XDB01CZO39|484|141
AACGAACGCTGGCGGCGCGTCTTAAGCATGCAAGTCGAACGGGAACTAGGAGCTTGCTTCTAGCGAGAGTGGCGGACTGGTGAGTAACACGTGGGTGACGTACCCCTAAGGATGGGGATAGCCTGTAGAAATACGGGGTAATACCGAATACGCTCGTTGCAGTAAAGAGCGATGAGGAAAGATGCTACGGCATCACCTAAGGAACGGCCCGCGGACTATTAGCTAGTTGGTATGGTAACGGCATACCAAGGCGACGATAGTTATCCGGCCTAAGAGGGTGAACGGACACATTGGGACTGAGATACGGCCCAGACTCCTACGGGA
>FQH3XDB01AOT1G|485|1
AACGAACGCTGGCGGCGCGTCTTAAGCATGCAAGTCGGGCGGCAAGCCTCCTCCGGGAGGCCTAGAGCGGCGGACTGGTGAGCAACGCGTGGGCGACGTGCCCTCCGGACGGGGATAGCCTGTGGAAACACAGGGGTAATACCGGACAAGCCGCAGCGGGCTGGAGCCGCTGCGGGAAAGGCGCCACGGCGCCGCCGGAGGATCGGCCCGCGTCCCATCAGCTAGCAGGCGGGGTAAAGGCCCACCTGGGCGATGACGGGTATCCGGCCTGAGAGGGTGTACGGACACATTGGGACTGAGATACTGCCCAGACTCCTACGGGAGGCTGCAGTCGAGAATTTTGGGCAATGGGGGCAACCCTGACCCAGCAATGCCGCGTGCGGGATGAAGGTCTTCGGATTGTAAACCGCTGTCAAGAGGACGAATACAATTGACGGTACCTCTGGAGGAAGTCACGGCTAACGTACGTGCCAGCAGCCGCGGT
>FQH3XDB01D30NR|486|1
AACGAACGCTGGCGGCGCGTCTTAAGCATGCAAGTCGAACGGGAACTAGGAGCTTGCTTCTAGCGAGAGTGGCGGACTGGTGAGTAACACGTGGGTGACGTACCCTAAGGATGGGGATAGCCTGTAGAAATACGGGGTAATACCGAATACGCTCGTTGCAGTAAAGAGCGATGAGGAAAGATGCTACGGCATCACCTAAGGAACGGCCCGCGGACTATTAGCTAGTTGGTATGGTAACGGCATACCAAGGCGACGATAGTTATCCGGCCTAAGAGGGTGATCGGCCACACTGGGACTGAGACACGGCCCAGACTCCTACGGGAGACAGCAGTTAGGAATATTCGTCAATGGGGGAAAACCCTGAACGAGCAATGCCGCGTGAAGGATGACGGTCCTATGGATTGTAAACTTCTGTTGTTAGGGAAGACGACCTGTATAGGAAATGAT
>FQH3XDB01AO6KR|487|15
AACGAACGCTGGCGGCGCGTCTTAAGCATGCAAGTCGAACGGCAAGCTGCCCTCGGGCAGCCCAGAGTGGCGGACTGGTGAGTAACACGTAGGTGACGTACCCTTCGGACGGGGATAGCCTGTGGAAACACAGGGTAATACCGGATAAGGCCGTCTGCGCTGGAGGCAGGCGGGGAAAGGGAGCTTCGGCTCCGCCGGAGGATCGGCCTGCGCCCCATCAGCTTGACGGCGGGGTAAAGGCCCACCGTGGCGATGACGGGTACCGGACTGAGAGGTTGGCCGGGCACATTGGGACTGAGATACGGCCCAGACTCCTACGGGAGGCAGCAGCTAAGAATATTCCGCAAGTGGGGGGAACCCTGACGGAGCGACGCCGC
>FQH3XDB01DVL2X|488|7
AACGAACGCTGGCGGCGCGTCTTAAGCATGCAAGTCGAACGGCAAGCTGCCCTCGGGCAGCCCAGAGTGGCGGACTGGTGAGTAACACGTAGGTGACGTACCCTTCGGACGGGGATAGCCTGTGGAAACACAGGGTAATACCGGATAAGGCCGTCTGCGCTGGAGGCAGGCGGGAAAGGAGCTTCGGCTCCGCCGGAGGATCGGCCTGCGCCCCATCAGCTTGACGGCGGGTAAAGGCCCACCGTGGCGATGACGGGTACCCGGACTGAGAGGTTGGCCGGGCACATTGGGACTGAGATACGGCCCAGACTCCTACGGGAGCAGCAGCTAAGAATATTCCGCAAGTGGGGGGAACCCTGACGGAGCGACGCCGCGTGGACGATGAAGGCCGGAAGGTTGTAAAGTCCTTTGC
>FQH3XDB01EKISS|489|2
AACGAACGCTGGCGGCGCGTCTTAAGCATGCAAGTCGAACGGCAAGCTGCCCTCGGGCAGCCCAGAGTGGCGGACTGGTGAGTAACACGTAGGTGACGTACCCTTCGGACCGGGGATAGCCTGTGGAAACACAGGGTAATACCGGATAAGGCCGTCTGCGCTGGAGGCAGGCGGGAAAGGGAGCTTCGGCTCCGCCGGAGGATCGGCCTGCGCCCCATCAGCTTGACGGCGGGTAAAGCCCACCGTGGCGATGGACGGGTACCCGGACTGAGAGGTTGGCCGGGCACATTGGGACTGAGAT
>FQH3XDB01BJJ6O|490|6
AACGAACGCTGGCGGCGCGTCTTAAGCATGCAAGTCGAACGGCAAGCTGCCCTCGGGCAGCCCAGAGTGGCGGACTGGTGAGTAACACGTAGGTGACGTACCCTTCGGACGGGGATAGCCTGTGGAAAACACAGGGGTAATACCGGATAAAGGCCGTCTGCGCTGGAGGCAGGCGGGGAAAGGGAGCTTCGGCTCCGCCGGAGGATCGGCCTGCGCCCCATCAGCTTGACGGCGGGTAAAGGCCCACCGTGGCGATGACGGGTACCCGGACTGAGAGGTTGGCCGGGCACAGTTGGGACTGAGATACGGCCAGACTCCTACGGGAGG
>FQH3XDB01BR9VA|491|2
AACGAACGCTGGCGGCGCGTCTTAAGCATGCAAGTCGAACGGCAAGCTGCCCTCGGGCAGCCCAGAGTGGCGGACTGGTGAGTAACACGTAGGTGACGTACCCTTCGGACGGGGATAGCCTGTGGGAAAACACGAGGGGTTAAATACCGGATAAAGGCCGTCTGCGCTGGAGGCAGGCGGGGAAAGGGAGCTTCGGCTCCGGCCGGAGGATCGGCCTGCGCCCCATCAGCTTGACGGCGGGTAAAGGCCCACCGTGGCGATGACGGGTACCCGGACTGAGAGGTTGGCCGGGGCACATTGGGGACTGAGATACGGCCCAGGACTCC
>FQH3XDB01CVW3A|492|1
AACGAACGCTGGCGGCGCGTCTTAAGCATGCAAGTCGAACGGGAACTAGGAGCTTGCTTCTAGCGAGAGTGGCGGACTGGTGAGTAACACGTGGGTGACGTACCCTAAGGATGGGGATAGCCTGTAGAAATACGGGGTAATGCCGAATACGCTCGTTGCAGTAAAGAGCGATGAGGAAAAGATGCTACGGCATCACCTAAGGAACGGCCCGCGGACTATTAGCTAGTTGGTATAGGTAACGGCATACCAAGGCGACGATAGTTATCCGGCCTAAGAGGGTGAACGGACACAGTTGGGACTGAGATACGG
>FQH3XDB01DG64V|493|1
AACGAACGCTGGCGGCGCGTCTTAAGCATGCAAGTCGAACGGGAACTAGGAGCTTGCTTCTAGCGAGAGTGGCGGACTGGTGAGTAACACGTGGGTGACGTACCCTAAGGATGGGGATAGCCTGTAGAAATACGGGGTAATGCCGAATACGCTCGTTGCAGTAAAGAGCGATGAGGAAAGATGCTACGGCATCACCTAAGGAACGGCCCGCGGACTATTAGCTAGTTGGTATGGTAACGGCATACCAAGGCGACGATCGGTAGCCGGACTGAGAGGTTGAACGGCCACATTGGGACTGAGACACGGCCCCAGACTCCTACGGGAGGCAGCAGTGGGGAATATTGCACAATGGGGAAACCCTGATGCCAGCGACG
>FQH3XDB01AHCJ0|494|6
AACGAACGCTGGCGGCGCGTCTTAAGCATGCAAGTCGAGCGGCAAGATTGGAGCTTGCTCCAATCCCAGAGCGGCGGACTGGTGAGTAACACGTAGGCGACGTGCCCTTGGGACGGGGATAGCCTGTGGAAACACAGGGTAATACCGGATAAGGCCGTATGCGTTGGAGGCATACGGGGAAAGGAGCCATGGCTTCGCACAAGGAGCGACCTGCGGCCCATCAGCTAGTTGGCGGGTAAGGCCCACCAAGGCAATGACGGGTATCCGGCCTGAGAGGGTGAACGGACACATTGGGACTGAGATACGGCCCAGACTCCTACGGGAGGCAGCAGCTAAGAATATTCCGCAATGCGCGAAAGCGTGACGGAGCGACGCCGCGTGGACGAGT
>FQH3XDB01BX11V|495|3
AACGAACGCTGGCGGCGCGTCTTAAGCATGCAAGTCGAGCGGCAAGATTGGAGCTTGCTCCAATCCCAGAGCGGCGGACTGGTGAGTAACACGTAGGCGACGTGCCCTTGGGACGGGGATAGCCTGTGGAAACACAGGGGTAATACCGGATAAGGCCGTATGCGTTGGAGGCATACGGGGAAAGAGCCATGGCTTCGCACAAGGAGCGACCTGCGGCCCATCAGCTAGTTGGCGGGGTAAGGCCCACCAAGGCAATGACGGGTATCCGGCCTGAGAGGGTGAACGGACACATTGGGGACTGAGATACGGCCCAGACTCCTACGGGAGGCAGCAGCTAAGATATTCCGCAATGCGCGAAAGCGTGACGGAGCGACGCCGCGTGGACGATGGAGGCCGGAAGGCTGTAAAGTCCTTTTAAGGCAGAGGAATAAGGCAAGCAGGG
>FQH3XDB01C3IW0|496|3
AACGAACGCTGGCGGCGCGTCTTAAGCATGCAAGTCGAGCGGCAAGATTGGGGCTTACTCCAATCCTAGAGCGGCGGACTGGTGAGTAACACGTAGGCGACGTGCCCTTGGGACGGGGATAGCCTGTGGAAACACAGGGGTAATACCGGATAAGGCCGTATGCGTTGGAGGCATACGGGGAAAGAGCCATGGCTCCGACCGAGGATCGACCTGCGGCCCATCAGCTAGTTGGCGGGGCAAGGGCCCACCAAGGCAATGACGGGTATCCGGCCTGAGAGGGTGAAACGGACACATTGGGACTGAGATACGGCCCAGACTCCTAC
>FQH3XDB01AQTPL|497|18
AACGAACGCTGGCGGCGCGTCTTAAGCATGCAAGTCGAGCGGCAAGATTGGGGCTTGCTCCAATCCTAGAGCGGCGGACTGGTGAGTAACACGTAGGCGACGTGCCCTTGGGACGGGGATAGCCTGTGGAAACACAGGGTAATACCGGATAAGGCCGTATGCGTTGGAGGCATACGGGGAAAGGAGCCATGGCTTCGACCGAGGATCGACCTGCGGCCCATCAGCTAGTTGGCGGGTAAGGCCCACAAGGCAATGACGGGTATCCGGCTGGAGAGGGTGAACGGACACATTGGGACTGAGATACGGCCCAGACTCC
>FQH3XDB01A8PIY|498|4
AACGAACGCTGGCGGCGCGTCTTAAGCATGCAAGTCGAGCGGCAAGATTGGGGCTTGCTCCAATCCCAGAGCGGCGGACTGGTGAGTAACACGTAGGCGACGTGCCCTTGGGACGGGGATAGCCTGTGGAAACACAGGGTAATACCGGATAAGGCCGTATGCGCTGGAGGCAGTACGGGGAAAGGAGCCATGGCTTCGACCGAGGATCGACCTGCGGCCCATCAGCTAGTTGGCGGGTAAGGCCCACAAGGCAATGACGGGTATCCGGCCTGGAGAGGGTGAACGGACACATTGGGACTGAGATACGGCCCAGACTCC
>FQH3XDB01C4LP4|499|3
AACGAACGCTGGCGGCGCGTCTTAAGCATGCAAGTCGAGCGGCAAGATTGGGGCTTGCTCCAATCCCAGAGCGGCGGACTGGTGAGTAACACGTAGGCGACGTGCCCTTGGGACGGGGATAGCCTGTGGAAACACAGGGTAATACCGGATAAGGCCGTATGCGTTGGAGGCATACGGGGAAAGGAGCCATGGCTTCGCACAAGGAGCGACCTGCGGCCCATCAGCTGGTTGGCGGGGCAAGGGCCCACCAGGCAATGACGGGTATCCGGCCTGGAGAGGGTGAACCGGGACACGAGTTGGG
>FQH3XDB01CEGIR|500|1
AACGAACGCTGGCGGCGCGTCTTAAGCATGCAAGTCGAGCGGCAAGATTGGGGCTTGCTCCAATCCCAGAGCGGCGGACTGGTGAGTAACACGTAGGCGACGTGCCCTTGGGACGGGGATAGCCTGTGGAAACACAGGGTAATACCGGATAAGGCCGTATGCGTTGGAGGCAGTACGGGGAAAGGAGCTATGGCTTCGCACAAGGAGCGACCTGCGGCCCATCAGCTAGTGGCGGGTAAGGCCCACAAGGCAATGGACGGGTATCCGGCCTGG
>FQH3XDB01DDIUE|501|12
AACGAACGCTGGCGGCGCGTCTTAAGCATGCAAGTCGAGCGGCAAGATTGGGGCTTGCTCCAATCCCAGAGCGGCGGACTGGTGAGTAACACGTAGGCGACGTGCCCTTGGGACGGGGATAGCCTGTGGAAACACAGGGGTAATACCGGATAAGGCCGTATGCGTTGGAGGCATACGGGGAAAGGAGCTATGGCTTCGACCGAGGATCGACCTGCGGCCCATCAGCTAGTTGGCGGGTAAGGCCCACCAAGGCAATGACGGGTATCCGGCCTGAGAGGGTGAACGGACACATTGGGACTGAGATACGGCCCAGACTCCTACGGGAGCAGCAGCTAAGATATTCCGCAATGCGCGAAAGCGTGACGGAGCGAC
>FQH3XDB01BBPDA|502|4
AACGAACGCTGGCGGCGCGTCTTAAGCATGCAAGTCGAGCGGCAAGATTGGGGCTTGCTCCAATCCTAGAGCGGCGGACTGGTGAGTAACACGTAGGCGACGTGCCCTTGGGACGGGGATAGCCTGTGGAAAACACAGGGGTAATACCGGATAAGGCCGTATGCGTTGGAGGCATACGGGGAAAGAGCCATGGCTTCGACCGAGGATCGACCTGCGGCCATCAGCTAGTTGGCGGGTAAGGCCCACAAGGCAATGACGGGTATCCGGCCTGGAGAGGGTGAACGGACACATTGGGACT
>FQH3XDB01DWHP1|503|1
AACGAACGCTGGCGGCGCGTCTTAAGCATGCAAGTCGAGCGGCAAGATTGGGGCTTGCTCCAATCCCAGAGCGGCGGACTGGTGAGTAACACGTAGGCGACGTGCCCTTGGGGCGGGGATAGCCTGTGGAAACACAGGGTAATACCGGATAAGGCCGTATGCGTTGGAGGCATACGGGGAAAGGAGCCATGGCTCCGACCGAGGATCGACCTGCGGCCCATCAGCTGGTTGGCGGGGCAAGGGCCCACCAAGGCAATGACGGGTATCCGGCCTGAGAGGGTGAAACGGACACATTGGGACTGAGATACGGCCCAGACTCCTACGGGGA
>FQH3XDB01B8J9N|504|4
AACGAACGCTGGCGGCGCGTCTTAAGCATGCAAGTCGAGCGGCAAGATTGGGGCTTGCTCCAATCCCAGAGCGGCGGACTGGTGAGTAACACGTAGGCGACGTGCCCTTGTGACGGGGATAGCCTGTGGAAACACAGGGTAATACCGGATAAGGCCGTATGCGCTGGAGGCATACGGGGAAAGGAGCCATGGCTTCGCACAGGGAGCGACCTGCGGCCCATCAGCTAGTTGGCGGGTAAGGCCCACCAAGGCAACGACGGGTATCCGGCCTGAGAGGGTGAACGGACACATTGGGACTGAG
>FQH3XDB01B1XKI|505|1
AACGAACGCTGGCGGCGCGTCTTAAGCATGCAAGTCGAGCGGCAAGATTGGGGCTTGCTCCAATCCTAGAGCGGCGGACTGGTGAGTAACACGTAGGCGACGTGCCCTTGGGACGGGGATAGCCTGTGGAAACACAGGGTAATACCGGATAAGGCCGTATGCGTTGGAGGCATACGGGGAAAGAGCCATGGCTTCGGACCGAGGATCGACCTGCGGCCCATCAGCTAGTTGGCGGGTAAGGCCACAGCAATGACGGGTATCCGGCCTGAG
>FQH3XDB01E4AVM|506|2
AACGAACGCTGGCGGCGCGTCTTAAGCATGCAAGTCGAGCGGCAAGATTGGGGCTTGCTCCAATCCTAGAGCGGCGGACTGGTGAGTAACACGTAGGCGACGTGCCCTTGGGACGGGGATAGCCTGTGGAAACACAGGGGTAATACCGGATAAGGCCGTATGCGTTGGAGGCATACGGGGAAAGGAGCTATGGCTTCGACCGAGGAGCGACCTGCGGCCCATCAGCTAGTTGGCGGGTAAGGCCCACCAAGGCAATGACGGGTATCCGGCCTGAGAGGGTGAACGGACACATTGGGACTGAGATACGGCCCAGACTCCTACGGGAGCAGCAGCTAAGAATATTCCGCAATGCGCGAAAGCGTGACGGAGCGACGCCGCGTGGACGATGGAGGCCGGAAGGCTGTAAA
>FQH3XDB01AP987|507|1
AACGAACGCTGGCGGCGCGTCTTAAGCATGCAAGTCGAGCGGTAAGGCGGGAGCTTGCTCCTGCCCCAGAGCGGCGGACTGGTGAGTAACGCGTGGGTGACGTACCCTTTAGATGGGGATAGCCGGTAGAAATACCGGGTAATACCGAATAAGCTTGCTACTTTAATGTGAAGTAAGGAAAGGAGCGCATGCTCCGCTGAAGGACGGCCCGCGAACTATTAGCTTGGTTGGTGAGGTAAGGCCACGAAGGCGACGATAGTTA
>FQH3XDB01D6ZKJ|508|2
AACGAACGCTGGCGGCGCGTCTTAAGCATGCAAGTCGAGCGGTAAGGCGGGAGCTTGCTCCTGCCCCAGAGCGGCGGACTGGTGAGTAACGCGTGGGTGACGTACCCTTTAGATGGGGATAGCCGGTAGAAATACCGGGTAATACCGAATAAGCTTGCTTCTTTAATGTGAAGTAAGGAAAGGAGCGCATGCTCCGCTGAAGGAACGGCCCGCGAACTATTAGCTTGTTGGTGAGGTAAAAGGCCCACCAAGGCGACGATAGTTATCCGGCCTAAGAGGGTGAACGGACACATTGGGACTGAGATACGGCCCAGACTCCTACGGGAGCAGCAGCTAAGATA
>FQH3XDB01AFHJR|509|1
AACGAACGCTGGCGGCGCGTCTTAAGCATGCAAGTCGAGCGGTAAGGCGGGAGCTTGCTCCTGCCCCAGAGCGGCGGACTGGTGAGTAACGCGTGGGTGACGTACCCTTTAGATGGGGATAGCCGGTAGAAATACCCGGGGTAATACCGAATAAGCTTGCTTTTTTAAAGTGAAGTAAGGAAAGGAGCGCATGCTCCGCTGAAGGAACGGCCCGCGAACTATTAGCTAGTTGGTGAGGTAAAGGCCCACCAAGGCGACGATAGTTATCCGGCCCAAGAGGGGTGAACGG
>FQH3XDB01DTEWW|510|5
AACGAACGCTGGCGGCGCGTCTTAAGCATGCAAGTCGAGCGGTAAGGCGGGGCTTGCTCCTGCCCCAGAGCGGCGGACTGGTGAGTAACGCGTGGGTGACGTACCCTTTAGATGGGGATAGCCGGTAGAAATACCGGGTAATACCGAATAAGCTTGCTTTTTAAAGTGAAGTAAGGAAAGGAGCGCATGCTCCGCTGAAGGAACGGCCCGCGAACTATTAGCTAGTTGGTGAGGTAAAGGCCCACCAAGGCGACGATAGTTATCCGGCCTAAGAGGGTGAACGGACACATTGGGACTGAGATACGGCCCAGACTCCTACGGGAGCAGCAGCTAAGATATTCCGCAAAGTGGGGGAAAACCCTGACGGAGCGACGCCGCGTGGATGATGAAGGTCGGAAA
>FQH3XDB01DYGWG|511|8
AACGAACGCTGGCGGCGCGTCTTAAGCATGCAAGTCGAGCGGTAAGGCGGGAGCTTGCTCCTGCCCCAGAGCGGCGGACTGGTGAGTAACGCGTGGGTGACGTACCCTTTAGATGGGGATAGCCGGTAGAAATACCGGGTAATACCGAATAAGCTTGCTACTTTAATGTGAAGTAAGGAAAGGAGCGCATGCTCCGCTGAAGGAACGGCCCGCGAACTATTAGCTTGTTGGTGAGGTAAAGGCCCACCAAGGCGACGATAGTTATCCGGCCTAAGAGGGTGAACGGACACATTGGGACTGAGATACGGCCCAGACTCCTACGGGAGCAGCAGCTAAGAATATTCCGCAAGTGGGGGAAACCCTGACGGAGCGACGCCGCGTGGATGATGGAAGGTCGGAA
>FQH3XDB01CRHT8|512|7
AACGAACGCTGGCGGCGCGTCTTAAGCATGCAAGTCGAGCGGTAAGGCGGGAGCTTGCTCCTGCCCCAGAGCGGCGGACTGGTGAGTAACGCGTGGGTGACGTACCCTTTAGATGGGGATAGCCGGTAGAAATACCGGGTAATACCGAATAAGCTTGCTACTTTAATGTGAAGTAAGGAAAGGAGCGCATGCTCCGCTGAAGGAACGGCCCGCGAACTATTAGCTTGTTGGTGAGGTAAAGGCCCACCAAGGCGACGATAGTTATCCGGCCTAAGAGGGTGAACGGACACATTGGGACTGAGATACGGCCCAGACTCCTACGGGAGCAGCAGCTAAG
>FQH3XDB01E0WHM|513|1
AACGAACGCTGGCGGCGCGTCTTAAGCATGCAAGTCGAGCGGCAAGATTGGGGCTTGCCCCAATCCCAGAGCGGCGGACTGGTGAGTAACACGTAGGCGACGTGCCCTTGTGACGGGGATAGCCTGTGGAAACACAGGGTAATACCGGATAAGGCCGTATGCGTTGGAGGCATACGGGGAAAGGAGCCATGGCTTCGCACAAGGAGCGGCCTGCGGCCCATCAGCTAGTTGGCGGGGCAAGGGCCCACAAGGCAATGACGGGTATCCGGCCT
>FQH3XDB01AMY9K|514|1
AACGAACGCTGGCGGCGCGTCTTAAGCATGCAAGTCGAGCGGCAAGATTGGGGCTTGCTCCAATCCCAGAGCGGCGGACTGGTGAGTAACACGTAGGCGACGTGCCCTTGGGACGGGGATAGCCTGTGGAAACACAGGGTAATACCGGATAAGGCCGTATGCGTTGGAGGCATACGGGGAAAGGAGCTATGGCTTCGCACAAGGGAGCGACCTGCGGCCCATCAGCTAGTTGGCGGGTAAGGCCCACAGGCAATGACGGGTATCCGG
>FQH3XDB01AZL50|515|4
AACGAACGCTGGCGGCGCGTCTTAAGCATGCAAGTCGGACGGCAAGAGAGAGCTTGCTCTCTCCTAGAGTGGCGGACTGGTGAGGAACGCGTGGGTGACGCACCCTCCTGACGGGGACAGCTCCTAGAAATAGGAGATAATACCGGATACGCTGCATGTAAGTAGAGGACATGCAGGAAAGGAGCCTTTGCTCCGCAGGGGAACGGCCCGCGTGCTATTAGCTGGACGGCGGGGTAACGCCCACCGTGGCGACGATAGCTACCCGGCTAAGAGGGCAAACGGGCACATTGGGACTGAGATACGGCCCAGA
>FQH3XDB01AM18J|516|8
AACGAACGCTGGCGGCGCGTCTTAAGCATGCAAGTCGGACGGCAAGAGAGAGCTTGCTCTCTCCTAGAGTGGCGGACTGGTGAGGAACGCGTGGGTGACGCACCCTCCTGACGGGGACAGCTCCTAGAAATAGGAGATAATACCGGATACGCTGCATATAAGTAGAGGATATGCAGGAAAGGAGCTTTTGCTCCGCAGGGGGAGCGGCCCGCGTGCTATTAGCTTGACGGCGGGTAACGCCCACCGTGGCGACGATAGCTACCCGGCTAAGAGGGCAAACGGGCACATTGGGACTGAGATACGGCCCAGACTCCTACGGGAGCAGCAGCTAAGAT
>FQH3XDB01CHUXG|517|6
AACGAACGCTGGCGGCGCGTCTTAAGCATGCAAGTCGGACGGCAGGCTGCCCCCGGGCAGCCGAGAGTGGCGGACTGGTGAGTAACACGTAGGCGACGTGCCCTCCGGGCGGGGATAGCCTGTGGAAACACAGGGGTAATACCGGATACGGCCGCATGCGCTGGAGGCATGCGGGGAAAGAGCTTCGGCTCCGCCGGAGGATCGGCCTGCGTCCCATCAGCTAGACGGCGGGTAAGGGCCACCGTGGCGATGACGGGTACCCGGACTGAGAGGTTGGCCGGGCACATTGGGACTGAGATACGGCCCAGACTCCTACGGGAGGCAGCAGCTAAGAATATTCCGCAAGTGGGGGAACCCTGACGGAGCGACGCCGCGTGGACG
>FQH3XDB01BS4WX|518|1
AACGAACGCTGGCGGCGCGTCTTAAGCATGCAAGTCGGACGGCAGGCTGCCCCCGGGCAGCCGAGAGTGGCGGACTGGTGAGTAACACGTAGGCGACGTGCCCTCCGGGCGGGGATAGCCTGTGGAAACACAGGGGTAATACCGGATACGGCCGCATGCGCTGGAGGCATGCGGGGAAAGAGCTTCGGCTCCGCCGGAGGATCGGCCTGCGTCCCATCAGCTAGACGGCGGGTAAGGCCACCGTGGCGATGACGGGTACCCGGACTGAGAGGTTGGCCGGGCACATTGGGACTGAGATACGGCCCAGACTCCTACGGGAGGCAGCAGCTAAGAATATTCCGCAAGTCGGGGGGAACCCTGGACGGAGCGACGCCGCGTGGACGA
>FQH3XDB01ASAAU|519|1
AACGAACGCTGGCGGCGCGTCTTAAGCATGCAAGTCGGACGGCAGGCTGCCCCCGGGCAGCCGAGAGTGGCGGACTGGTGAGTAACACGTAGGCGACGTGCCCTCCGGGCGGGGATAGCCTGTGGAAACACAGGGGTAATACCGGATACGGCCGCATGCGCTGGAGGCATGCGGGAAAGAGCTTCGGCTCCGCCGGAGGATCGGCCTGCGTCCCATCAGCTAGACGGCGGGTAAGGCCCACCGTGGCGATGACGGGTACCGGACTGAGAGGTTGGCCGGGCACATTGGGACTGAGATACGGCCCAGACTCCTACGGGA
>FQH3XDB01CU4LH|520|1
AACGAACGCTGGCGGCGCGTCTTAAGCATGCAAGTCGGACGGCAGGCTGCCCCCGGGCAGCCGAGAGTGGCGGACTGGTGAGTAACACGTAGGCGACGTGCCCTCCGGGCGGGGATAGCCTGTGGAAACACAGGGGTAATACCGGGATACGGCCGCATGCGCTGGAGGCATACGGGGAAAGGAGCTTCGGCTCCGCCGGAGGATCGGCCTGCGTCCCATCAGCTAGACGGCGGGGTAAGGGCCCACCGTGGCGATGACGGGTACCCGGACTGAGAGGTTGGCCGGGCACATTGGGACTGAGATACGGCCCAGACTCCTACGGGAGGCAGCAGCTAAGAATATTCCGCAAGTGGGGGAACCCTGACGGAGCGACGACGCGTGGACGATGGAAGGCCGGAAGGTTGTAAA
>FQH3XDB01CUB46|521|1
AACGAACGCTGGCGGCGCGTCTTAAGCATGCAAGTCGGACGGCAAGAGAGAGCTTGCTCTCTCCTAGAGTGGCGGACTGGTGAGTAACGCGTGGGCGACGCACCCTTCCGGCGGGGACAGCTCCTGGAAACAGGAGGTAATACCCGGTACGCTCCGTGATGTCAGAGGTCACGGAGGAAAGGCTCCTTTGAGCCGCGGAAGGCGCGGCCCGCGTGCCATTAGCCTGCTGGTGAGGTAACGGCCCACCAGGGCGATGATGGCTACCCGGCCTGAGAGGGCGGACGGGCACATTGGGACTGAGATACGGCCCAGACTCCGTACGGGAGGCAGCAGCTAAGAA
>FQH3XDB01BGWDC|522|1
AACGAACGCTGGCGGCGCGTCTTAAGCATGCAAGTCGGACGGCAAGAGAGAGCTTGCTCTCTCCTAGAGTGGCGGACTGGTGAGTAACGCGTGGGCGACGCACCCTTCCGGCGGGGACAGCTCCTGGAAACAGGAGGTAATACCCCGGTACGCTCCGTGATGTCAGAGGTCACGGAGGAAAGGCTCCTTTGAGCCGCGGAAGGCGCGGCCCGCGTGCCATTAGCCTGCTGGTGAGGTAACGGCCCACCAGGGCGATGATGGCTACCCGGCCTGAGAGGGCGGACGGGCACATTGGGACTGAGATACGGCCCAGACTCCTACGGGAGGCAGCAGCTAAGATATTCCGCAAGTGGGGGGAACCCCTGACGGAGCGACGCCGCGTGGACG
>FQH3XDB01DVCQ7|523|37
AACGAACGCTGGCGGCGCGTCTTAAGCATGCAAGTCGGACGGCAAGAGAGGGCTTGCCCTCTCCTAGAGTGGCGGACTGGTGAGTAACGCGTGGGCGACGCACCCTTCCGGCGGGGACAGCTCCTGGAAACAGGAGGTAATACCCCGGTACGCTCCGTGATGTCAGAGGTCACGGAGGAAAGGCTCCTTTGAGCCGCGGAAGGCGCGGCCCGCGTGCCATTAGCCTGCTGGTGAGGTAACGGCCCACCAGGGCGATGATGGCTACCCGGCCTGAGAGGGCGGACGGGCACATTGGGACTGAGATACGGCCCAGACTCCTACGGGAGGCAGCAGCTAAGATATTCCGCAAGTGGGGGAACCCTGACGGAGCGACGCCGCG
>FQH3XDB01ERPG9|524|1
AACGAACGCTGGCGGCGCGTCTTAAGCATGCAAGTCGGACGGCAAGAGAGGGCTTGCCCTCTCCTAGAGTGGCGGACTGGTGAGTAACGCGTGGGCGACGCACCCTTCCGGCGGGGACAGCTCCTGGAAACAGGAGGTAATACCCGGTACGCTCCGTGATGTCAGAGGTCACGGAGGAAAGGCTCCTTTGAGCCGCGGAAGGCGCGGCCGCGTGCCATTAGCCTGCTGGTGAGGTAACGGCCACCAGGGCGATGATGGCTACCGGCCTGAGAGGGCGACGGGCACATTGGGACTGAGATACGGCCCAGACTCCTACGGAGGCAGCAGCTAAGATATTCGCAATGGGGGAACCCT
>FQH3XDB01EU80O|525|8
AACGAACGCTGGCGGCGCGTCTTAAGCATGCAAGTCGGACGGCAAGAGAGGGCTTGCCCTCTCCTAGAGTGGCGGACTGGTGAGTAACGCGTGGGCGACGCACCCTTCCGGCGGGGACAGCTCCTGGAAACAGGAGGTAATACCCCGGTACGCTCCGTGATGTCAGAGGTCACGGAGGAAAGGCTCCTTGAGCCGCGGAAGGCGCGGCCCGCGTGCCATTAGCCTGCTGGTGAGGTAACGGCCCACCAGGGCGATGATGGCTACCCGGCCTGAGAGGGCGGACGGGCACATTGGGACTGAGATACGGCCCAGACTCCTACGGGAGCAGCAGCTAAGATATTCCGCAAGTGGGGGAACCCTGGACGGAGCGACGCCGCG
>FQH3XDB01EGM1R|526|1
AACGAACGCTGGCGGCGCGTCTTAAGCATGCAAGTCGGACGGCAAGAGAGGGCTTGCCCTCTCCTAGAGTGGCGGACTGGTGAGTAACGCGTGGGCGACGCACCCTTCCGGCGGGGACAGCTCCTGGAAACCAGGAGGTAATACCCGGTACGCTCCGTGATGTCAGAGGTCACGGAGGAAAGGCTCCTTTGAGCCGCGGAAGGCGCGGCCCGCGTGCCATTAGCCTGCTCGGTGAGGTAACGGCCACCAGGGCGATGATGGCTA
>FQH3XDB01EUOZO|527|9
AACGAACGCTGGCGGCGCGTCTTAAGCATGCAAGTCGGACGGCAAGAGAGGGCTTGCCCTCTCCTAGAGTGGCGGACTGGTGAGTAACGCGTGGGCGACGCACCCTTCCGGCGGGGACAGCTCCTGGAAACAGGAGGTAATACCCGGTACGCTCCGTGATGTCAGAGGTCACGGAGGAAAGGCTCCTTGAGCCGCGGAAGGCGCGGCCCGCGTGCCATTAGCCTGCTGGTGAGGTAACGGCCCACCAGGGCGATGATGGCTACCCGGCCTGAGAGGGCGGACGGGCACATTGGGACTGAGATA
>FQH3XDB01C9RBQ|528|1
AACGAACGCTGGCGGCGCGTCTTAAGCATGCAAGTCGGACGGCAAGAGAGGGCTTGCCCTCTCCTAGAGTGGCGGACTGGTGAGTAACGCGTGGGCGACGCACCCTTCCGGCGGGGACAGCTCCTGGAAACAGGAGGTAATACCCGGTACGCTCCGTGATATTAGAAGTCACGGAGGAAAGGCTCCTTTGAGCCGCGGAAGGCGCGGCCCGCGTGCCATTAGCCGGCTGGTGAGGTAACGGCCCACCAGGGCGATGATGGCTACCCGGCCTGAG
>FQH3XDB01C9161|529|9
AACGAACGCTGGCGGCGCGTCTTAAGCATGCAAGTCGGACGGCAAGAGAGGGCTTGCCCTCTCCTAGAGTGGCGGACTGGTGAGTAACGCGTGGGCGACGCACCCTTCCGGCGGGGACAGCTCCTGGAAACAGGAGGTAATACCCCGGTACGCTCCGTGATATTAGAAGTTACGGAGGAAAGGCTCCTTTGAGCCGCGGAAGGCGCGGCCCGCGTGCCATTAGCCTGCTGGTGAGGTAACGGCCCACCAGGGCGATGATGGCTACCCGGCCTGAGAGGGCGGACGGGCACATTGGGACTGAGATACGGCCCAGACTCCTACGGGAGGCAGCAGCTAAGATATTCCGCAAGTGGGGGAACCCCTGACGGAGCGACGCCGCGTGGACGA
>FQH3XDB01COGL5|530|3
AACGAACGCTGGCGGCGCGTCTTAAGCATGCAAGTCGGACGGCAAGAGAGGGCTTGCCCTCTCCTAGAGTGGCGGACTGGTGAGTAACGCGTGGGCGACGCACCCTTCCGGCGGGGACAGCTCCTGGAAAACAGGAGGTAATACCCCGGTACGCTCCGTGATGTCAGAGGTCACGGAGGAAAGGCTCCTTTGAGCCGCGGAAGGCGCGGCCCGCGTGCCATTAGCCTGCTGGTGAGGTAACGGCCCACCAGGGCGATGATGGCTACCGGCCTGAGAGGGCGGACGGGCACGATTGGGACTGAGATACGGCCCAGACCTCCGTACGGGAGGCAGCAGCTAAGATA
>FQH3XDB01BKLO7|531|1
AACGAACGCTGGCGGCGCGTCTTAAGCATGCAAGTCGGACGGCAAGAGAGGGCTTGCCCTCTCCTAGAGTGGCGGACTGGTGAGTAACGCGTGGGCGACGCACCCGTTCCGGCGGGGACAGCTCCTGGAAACAGGAGGTAATACCCGGTACGCTCCGTTGATGTCAGAGGTCACGGAGGAAAGGCTCCTTTGAGCCGCGGAAGGCGCGGCCCGCGTGCCATTAGCCTGCTGGTGAGGTAACGGCCCACCAGGGCGATGATGGCTA
>FQH3XDB01ALPKP|532|24
AACGAACGCTGGCGGCGCGTCTTAAGCATGCAAGTCGGACGGCAAGAGAGAGCTTGCTCTCTCCTAGAGTGGCGGACTGGTGAGGAACGCGTGGGTGACGCACCCTCCTGACGGGGACAGCTCCTAGAAATAGGAGATAATACCGGATACGCTGCATGTAAGTAGAGGACATGCAGGAAAGGAGCCTTTGCTCCGCAGGGGGAACGCCCGCGTGCTATTAGCTAGACGGCGGGGTAACGGCCCACCGTGGCGACGATAGCTACCCGGCCTAAGAGGGCAAACGGGCACATTGGGACTGAGATACGGCCCAGACTCCTACGGGAGGCAGCAGCTAAGATATTCCGCAAGTGGGGGAACCCTGACGGAGCGACGCCGCGTGGACGATGAAGGCCGGAAGGTTGTAAAGTCCTTTTATGCGCGAGGAAGTAAGGGGAGCGAGAGAAACGCTCTCTGGTGACTGTAG
>FQH3XDB01DJAH2|533|12
AACGAACGCTGGCGGCGCGTCTTAAGCATGCAAGTCGGACGGCAAGAGAGAGCTTGCTCTCTCCTAGAGTGGCGGACTGGTGAGGAACGCGTGGGTGACGCACCCTCCTGACGGGGACAGCTCCTAGAAATAGGAGATAATACCGGATACGCTGCATATAAGTAGAGGATATGCAGGAAAGGAGCTTTTGCTCCGCAGGGGGAGCGGCCCGCGTGCTATTAGCTTGACGGCGGGTAACGGCCCACCGTGGCGACGATAGCTACCCGGCTAAGAGGGCAAAACGGGCACATTGGGACTGAG
>FQH3XDB01B8XE6|534|1
AACGAACGCTGGCGGCGCGTCTTAAGCATGCAAGTCGGACGGCAAGAGAGAGCTTGCTCTCTCCTAGAGTGGCGGACTGGTGAGGAACGCGTGGGTGACGCACCCTCCTGACGGGGACAGCTCCTAGAAATAGGAGATAATACCGGATACGCTGCATGTAAGTAGAGGACATGCAGGAAAGAGCTTTTGCTCCGCAGGGGAGCGGCCCGCGTGCTATTAGCTTGACGGCGGGGTAACGGCCCACCGTGGCGACGATAGCCTACCCGGCTAAGAGGGCAAACGGGCACGATTGGGACT
>FQH3XDB01AOR24|535|11
AACGAACGCTGGCGGCGCGTCTTAAGCATGCAAGTCGGACGGCAAGAGAGGGCTTGCCCTCTCCTAGAGTGGCGGACTGGTGAGTAACGCGTGGGCGACGCACCCTTCCGGCGGGGACAGCTCCTGGAAACAGGAGGTAATACCCCGGTACGCTCCGTGATATTAGAAGTCACGGAGGAAAGGCTCCTTTGAGCCGCGGAAGGCGCGGCCCGCGTGCCATTAGCCGGCTGGTGAGGTAACGGCCCACCAGGGCGATGATGGCTACCCGGCCTGAGAGGGCGGACGGGCACATTGGGACTGAGATACGGCCCAGACTCCTACGGGAGGCAGCAGCTAAGATATTCCGCAATGGGGGGAACT
>FQH3XDB01EGGTJ|536|10
AACGAACGCTGGCGGCGCGTCTTAAGCATGCAAGTCGGACGGCAAGAGAGGGCTTGTCCTCTCCTAGAGTGGCGGACTGGTGAGTAACGCGTGGGCGATGCACCCTTCCGACGGGGACAGCTCCTGGAAACAGGAGGTAATACCCGGTACGCTCCGTGATGTCAGAGGTTACGGAGGAAAGGCTCCTTTGAGCCGCGGAAGGCGCGGCCCGCGTGCCATTAGCCGGCTGGTGAGGTAACGGCCACCAGGGCGATGATGGCTACCCGGCCTGAGAGGGCGGACGGGCACATTGGGACTGAGATACGGCCCAGACTCCTACGGGAGGCAGCAGCTAAGAATATTCCGCAAGTGGGGGGAACCCCTGGACGGAGCGACGCCGCGTGGACGATGAAGGCCGGAAGGCTGTAAAGTCC
>FQH3XDB01D1I6L|537|9
AACGAACGCTGGCGGCGCGTCTTAAGCATGCAAGTCGGACGGCAAGAGAGGGCTTGCCCTCTCCTAGAGTGGCGGACTGGTGAGTAACGCGTGGGCGACGCACCCTTCCGGCGGGGACAGCTCCTGGAAAACAGGAGGTAATACCCGGTACGCTCCGTGATATTAGAAGTTACGGAGGAAAGGCTCCTTTGAGCCGCGGAAGGCGCGGCCCGCGTGCCATTAGCCTGCTGGTGAGGTAACGGCCCACCAGGGCGATGATGGCTACCCGGCCTGAGAGGGCGGACGGGCACATTGGGACTGAGATACGGCCCAGACTCCTACGGGAGCAGCAGCTAAGAATATTCCGCAAGG
>FQH3XDB01APEFI|538|1
AACGAACGCTGGCGGCGCGTCTTAAGCATGCAAGTCGGACGGCAAGAGAGGGCTTGCCCTCTCCTAGAGTGGCGGACTGGTGAGTAACGCGTGGGCGACGCATCCTTCCGGCGGGGACAGCTCCTGGAAACAGGAGGTAATACCCGGTACGCTCCGTGATATTAGAAGTCACGGAGGAAAGGCTCCTTTGAGCCGCGGAAGGCGCGGCCCGCGTGCCATTAGCCGGCTGGTGAGGTAACGGCCCACCAGGGCGATGATGGCTACCCGGCCTGAGAGGGCGGACGGGCACATTGGGACTGAGATACGGCCCAGACTCCTACGGGAGCAGCAGCTAAGAATATTCCGCAATGGGGGAACCCCTGACGGAGCGACGCCGCGTGGACGATGAAGGCCGGAAGGCTGTAAAGTCCTTTTAGGACCGAGGAATAAGGAAGGTGAGAGAAACGCCTTCCGGTGACTGTAGGTCGTGAATAAGCAACGGCTAATTACGTGCCAGCAG
>FQH3XDB01B6BCU|539|3
AACGAACGCTGGCGGCGCGTCTTAAGCATGCAAGTCGGACGGCAAGAGAGGGCTTGTCCTCTCCTAGAGTGGCGGACTGGTGAGTAACGCGTGGGCGATGCACCCTTCCGACGGGGACAGCTCCTGGAAACAGGAGGTAATACCCGGTACGCTCCGTGATGTCAGAGGTTACGGAGGAAAGGCTCCTTTGAGCCGCGGAAGGCGCGGCCCGCGTGCCATTAGCCGGCTGGTGAGGTAACGGCCCACCAGGGCGATGATGGCTACCCGGCCTGAGAGGGCGGACGGGCACATTGGGA
>FQH3XDB01DHXEA|540|3
AACGAACGCTGGCGGCGCGTCTTAAGCATGCAAGTCGGACGGCAAGAGAGGGCTTGCCCCTCTCCTAGAGTGGCGGACTGGTGAGTAACGCGTGGGCGACGCACCCTTCCGGCGGGGACAGCTCCTGGAAACAGGAGGTAATACCCCGGTACGTTCCGTGATGTCAGAGGTCACGGAGGAAAGGCTCCTTTGAGCCGCGGAAGGCGCGGCCCGCGTGCCATTAGCCTGCTGGTGAGGTAACGGCCCACCAGGGCGATGATGGCTACCCGGCCTGAGAGGGCGGACGGGCACATTGGGACTGAGATACGGCCCAGG
>FQH3XDB01BQWHX|541|27
AACGAACGCTGGCGGCGCGTCTTAAGCATGCAAGTCGGGCGGGAACCATGCGCTTGCGCATGGTGAGAGCGGCGGACTGGCGAGTAACACGTGGGCGACGCGCCCTCCGGACGGGAATAGCCTGTAGAAATACAGGGTAATGCCGGATGCGAACGCACGGGCTGGAGCCGTGCGTGGAAAGCCCCCACGGGGGCGCCGGAGGAGCGGCCCCGCGGCCCATCAGCTTGTAGGCGGTGCAAGGGACCACCTAGGCTACGACGGGTACCCGGCCTCAGAGGGCGGACGGGCGCATTGGGACTGAGATACGGCCCAGA
>FQH3XDB01CEW7C|542|14
AACGAACGCTGGCGGCGCGTCTTAAGCATGCAAGTCGGGCGGCAAGCCTCCTCCGGGAGGCCTAGAGCGGCGGACTGGTGAGCAACGCGTGGGCGACGTGCCCTCCGGACGGGGATAGCCTGTGGAAACACAGGGGTAATACCGGACAAGCCCCCGCGGGCTGGAGCCGCGGGGGGAAAGGCGCCACGGCGCCGCCGGAGGATCGGCCCGCGTCCCATCAGCTAGCAGGCGGGTAAAGGCCCACCTGGGCGATGACGGGTATCCGGCCTGAGAGGGTGTACGGACACATTGGGACTGAGATACGGCCCAGACTCCTACGGGAGGCAGCAGCTAAGAATATTCCGCAAGTGGGGGAACCCTGACGGAGCGACG
>FQH3XDB01D08NU|543|49
AACGAACGCTGGCGGCGCGTCTTAAGCATGCAAGTCGGGCGGCAAGCCTCCTCCGGGAGGCCTAGAGCGGCGGACTGGTGAGCAACGCGTGGGCGACGTGCCCTCCGGACGGGGATAGCCTGTGGAAACACAGGGGTAATACCGGACAAGCCGCAGTGGGCTGGAGCCGCTGCGGGAAAGGCGCCACGGCGCCGCCGGAGGATCGGCCCGCGTCCCATCAGCTAGCAGGCGGGTAAAGGCCCACCTGGGCGATGACGGGTATCCGGCCTGAGAGGGTGTACGGACACATTGGGACTGAGATACGGCCCAGACTCCGTACGGGAGGCAGCAGCTAAGAATATTCCGCAAGTGGGGGAACCCTGACGGAGCGACGCCGCGTGGACGACGGAGGCCGGAAGG
>FQH3XDB01ALQJV|544|4
AACGAACGCTGGCGGCGCGTCTTAAGCATGCAAGTCGGGCGGCAAGCCTCCTCCGGGAGGCCTAGAGCGGCGGACTGGTGAGCAACGCGTGGGCGACGTGCCCTCCGGACGGGGATAGCCTGTGGAAAACACAGGGGTAATACCGGACAAGCCGCAGCGGGCTGGAGCCGCTGCGGGAAAGGCGCCACGGCGCCGCCGGAGGATCGGCCCGCGTCCCATCAGCTAGCAGGCGGGGTAAAGGCCCACCTGGGCGATGACGGGTATCCGGCCTGAGAGGGTGTACGGACACATTGGGACTGAGATACGGCCCAGACTCCTACGGGAGGCAGCAGCTAAGAATATTGGGCAAGTGGGCGAAAAGCCTGACCCCAGCGACGCCGCGTG
>FQH3XDB01DGFAI|545|3
AACGAACGCTGGCGGCGCGTCTTAAGCATGCAAGTCGGGCGGCAAGCCTCCTCCGGGAGGCCTAGAGCGGCGGACTGGTGAGCAACGCGTGGGCGACGTGCCCTCCGGACGGGGATAGCCTGTGGAAAACACAGGGGTAATACCCGGACAAGCCCCCGCGGGCTGGAGCCGCGGGGGAAAGCGCCACGGCGCCGCCGGAGGATCGGCCCGCGTCCCATCAGCTAGCAGGCGGGTAAAGCCCACCTGGGCGATGACGGGTATCCGGCCTGAGAGGGTGTACGGACACATTGGGACTGAGATACGGCCCAG
>FQH3XDB01BS5I0|546|1
AACGAACGCTGGCGGCGCGTCTTAAGCATGCAAGTCGGGCGGCAAGCCCTCCTCGGAGGGCCTAGAGCGGCGGACTGGTGAGCAACGCGTGGGCGACGTGCCCTCCGGACGGGGATAGCCTGTGGAAACACAGGGTAATGCCGGACAAGCCCCCGCGGGCTGGAGCCGCGGGGGAAAGGCGCCACGGCGCCGCCGGAGGATCGGCCCGCGTCCCATCAGCTAGCAGGCGGGGTAAAGGCCCACCTGGGCGATGACGGGTATCCGGCCTGAGAGGGTGTACGGACACATTGGGGACTGAGATACGGCCCAGACTCCTACGGGAGGCAGCAGCTAAGATATCCGCAATGGGGGAAACCCCTGACGGAGCGACGCCGCGTGGACG
>FQH3XDB01CCTUP|547|1
AACGAACGCTGGCGGCGCGTCTTAAGCATGCAAGTCGGGCGGCAAGCCCTCCTCCGGGAGGCCTAGAGCGGCGGACTGGTGAGCAACGCGTGGGCGACGTGCCCTCCGGACGGGGATAGCCTGTGGAAACACAGGGTAATACCGGACAAGCCGCAGCGGGGCTGGAGCCGCTGCGGGAAAAGGCGCCACGGCGCCGCCGGAGGATCGGCCCGCGTCCCATCAGCTAGCAGGCGGGTAAAGCCCACCTGGGCGATGACGGGTATCCGGCCTGAGAGGGTGTACGGACACATTGGGACTGAGATACGGCCCAGACTCCTACGGGAGGCAGCAGCTAAGAATATTCCGCAAGTGGGGGGAAACCCTGACGGAGCGACGCCGCGTGGACGACGGAGG
>FQH3XDB01EVJCR|548|1
AACGAACGCTGGCGGCGCGTCTTAAGCATGCAAGTCGGGCGGCAAGCTTACTTCGGTAAACCCAGAGCGGCGGACTGGTGAGTAACACGTGGGCGACGCACCCCCGGGACGGGGACAGCCTGTGGAAACACAGGGGTAATACCGGATAAGGCCGCATGCGCCGGAGGCATGCGGGGAAAGGAGCCTCGGCTCCGCCCGGGGAGCGGCCTGCGGCCCATCAGCTGGTTGGCGGGTAAGGCCCACAAGGCAACGACGGTAGTCCGGCCGTGAGAGGGTGAGCGGACACATTGGGACTGAGATACGGCCCAGACTCCTACGGGAGGCAGCAGCTAAGGATATTCCGCAAGTGGGGGGAACCCTGACGGAGCGACGCCGCGTGGACGACGGAGGCCGGAA
>FQH3XDB01AYZU9|549|1
AACGAACGCTGGCGGCGCGTCTTAAGCATGCAAGTCGGGCGGCAAGCCTCCTCCGGGAGGCCTAGAGCGGCGGACTGGTGAGCAACGCGTGGGCGACGTGCCCTCCGGACGGGGATAGCCTGTGGAAACACAGGGGTAATACCGGACAAGCCGCAGCGGGCTGGAGCCGCTGCGGGAAAGGCGCCACGGCGCCGCCGGAGGATCGGCCCGCGTCCCATCAGCTAGCAGGCGGGTAAAGCCACTGGGCGATGACGGGTATCCGGCCTGAGAGGGTGTACGGGACACATTGGGACTGAGATACGGCCCAGACTCCTACGGGAGGCAGCAGCTAAGATATTCCGCAATGGGGGGAACCCTGACGGAGCGACGCCGCGTGGACGACGGAGGCCGGAAGGCTGTAAAGTCCTTTTGCGCGCGAGGAATAAGGGGAGGAGGGAATGCCTTCCCGGTGACTGTAGCG
>FQH3XDB01EJOOZ|550|1
AACGAACGCTGGCGGCGCGTCTTAAGCATGCAAGTCGGGCGGCAAGCCCTCCTCGGAGGGCCTAGAGCGGCGGACTGGTGAGCAACGCGTGGGCGACGTGCCCTCCGGACGGGGATAGCCTGTGGAAACACAGGGGTAATACCGGACAAGCCCCCGCGGGCTGGAGCCGCGGGGGAAAGCGCCACGGCGCCGCCGGAGGATCGGCCCGCGTCCCATCAGCTAGCAGGCGGGTAAAGCCACTGGGCGATGACGGGTATCCGGCCTGAGAGGGTGTACGGACACATTGGGGACTGA
>FQH3XDB01BPGA0|551|4
AACGAACGCTGGCGGCGCGTCTTAAGCATGCAAGTCGGGCGGCAAGCCTCCTCCGGGAGGCCTAGAGCGGCGGACTGGTGAGCAACGCGTGGGCGACGTGCCCTCCGGACGGGGATAGCCTGTGGAAACACAGGGGTAATACCGGGACAAGCCCCCGCGGGCTGGAGCCGCGGGGGAAAGCGCCACGGCGCCGCCGGAGGATCGGCCCGCGTCCCATCAGCTAGCAGGCGGGTAAAGCCCACTGGGCGATGACGGGTATCCGGCCTGAGAGGGTGTACGGACACATTTGGGACTGAGATACGGCCCAGACTCCTACGGGAGGCAGCAGCTAAGATATTCCGCAATGGGGGAACCCTGACGGAGCGACGCC
>FQH3XDB01B4GL4|552|3
AACGAACGCTGGCGGCGCGTCTTAAGCATGCAAGTCGGGCGGCAAGCCTCCTCCGGGAGGCCTAGAGCGGCGGACTGGTGAGCAACGCGTGGGCGACGTGCCCTCCGGACGGGGATAGCCTGTGGAAACACAGGGGTAATACCGGACAAGCCGCAGCGGGCTGGAGCCGCTGCGGGAAAGGCGCCACGGCGCCGCCGGAGGATCGGCCCGCGTCCCATCAGCTAGCAGGCGGGTAAAGGCCCACCTGGGCGATGACGGGTATCCGGCCTGAGAGGGTGGACGGCCACATTGGAACTGAGACACGGTCCAGACTCCTACGGGAGCAGCAGCTAAGATCTTCCACAATGGACGAAAGTCTGATGGAGCGACGCCGCGTGGACGACGAAGGCCGTGAGGTTGTAAAGTCC
>FQH3XDB01AVAV2|553|2
AACGAACGCTGGCGGCGCGTCTTAAGCATGCAAGTCGGGCGGCAAGCCTCCTCCGGGAGGCCTAGAGCGGCGGACTGGTGAGCAACGCGTGGGCGACGTGCCCTCCGGACGGGGATAGCCTGTGGAAACACAGGGGTAATACCGGACAAGCCGCAGCGGGCTGGAGCCGCTGCGGGAAAGGCGCCACGGCGCCGCCGGAGGATCGGCCCGCGTCCCATCAGCTAGCGAGGCGGGGTAAAGGCCCACCGTGGGCGATGACGGGTATCCGGCCTGA
>FQH3XDB01AP763|554|14
AACGAACGCTGGCGGCGCGTCTTAAGCATGCAAGTCGGGCGGCAAGATTGGGGCCTGCCCCAATCCCAGAGCGGCGGACTGGTGAGTAACACGTAGGCGACGTGCCCCTGTGACGGGGATAGCCTGTGGAAACACAGGGTAATACCGGATAAGGCCGTGTGCGCTGGAGGCATACGGGGAAAGGAGCCATGGCTTCGCACAGGGAGCGGCCTGCGGCCCATCAGCTGGTTGGCGGGTAAGGCCCACCAAGGCAATGACGGGTATCCGGCCTGAGAGGGTGAACGGACACATTGGGACTGAGATACGGCCCAGACTCCTACGGGAGGCAGCAGCTAAGATATTCCGCAATGCGCGAAAGCGTGACGGAGCGACGCCGCGTGGACGACGGAGGCCGGAAGGCTGTAAAGTCC
>FQH3XDB01CRAYF|555|1
AACGAACGCTGGCGGCGCGTCTTAAGCATGCAAGTCGGGCGGCAAGATTGGGGCTTGCTCCAATCCCAGAGCGGCGGACTGGTGAGTAACACGTAGGCGACGTGCCCTTGTGACGGGGATAGCCTGTGGAAACACAGGGTAATACCGGATAAGGCCGTATGCGCTGGAGGCATACGGGGAAAGGAGCCATGGCTTCGCACAGGGAGCGACCTGCGGCCCATCAGCTAGTTGGCGGGGCAAGGGCCCACCAAGGCAATGACGGGTATCCGGCCTGAGAGGGTGGACGGACACATTGGGACTGAGATACGGCCCAGACTCCTACGGGAGGCAGCAGCTAAGAATATCCGCAATGCGCGAAAGCGTGACGGAGCGACGCCGCGTGGACGAGTGG
>FQH3XDB01CXAPC|556|9
AACGAACGCTGGCGGCGCGTCTTAAGCATGCAAGTCGGGCGGCAAGATTGGGGCTTGCTCCAATCCCAGAGCGGCGGACTGGTGAGTAACACGTAGGCGACGTGCCCTTGTGACGGGGATAGCCTGTGGAAACACAGGGTAATACCGGATAAGGCCGTATGCGCTGGAGGCATACGGGGAAAGGAGCCATGGCTCCGCACAAGGAGCGGCCTGCGGCCCATCAGCTGGTTGGCGGGTAAGGCCCACCAAGGCAATGACGGGTATCCGGCCTGAGAGGGTGAACGGACACATTGGGACTGAGATACGGCCCAGACTCCTACGGGAGCAGCAGCTAAGATATTCCGCAATGCGCGAAAGCGTGACGGAGCGACGCCGCGTGGACGACGGAGGCCGGAAGGCT
>FQH3XDB01EL7KE|557|1
AACGAACGCTGGCGGCGCGTCTTAAGCATGCAAGTCGGGCGGCAAGATTGGGGCTTGCTCCAATCCCAGAGCGGCGGACTGGTGAGTAACACGTAGGCGACGTGCCCTTGTGACGGGGATAGCCTGTGGAAACACAGGGTAATACCGGATAAGGCCGTATGCGCTGGAGGCAGTACGGGGAAAGGAGCCATGGCTTCGCACAGGGAGCGACCTGCGGCCCATCAGCTAGTTGGCGGGCAAGGGCCCACAAGGCAATGACGGTA
>FQH3XDB01BR1H3|558|10
AACGAACGCTGGCGGCGCGTCTTAAGCATGCAAGTCGGGCGGGAACCATGCGCTTGCGCATGGTGAGAGCGGCGGACTGGCGAGTAACACGTGGGCGACGCGCCCTCCGGACGGGAATAGCCTGTAGAAATACAGGGTAATGCCGGATGCGAACGCACGGGCTGGAGCCGTGCGTGGAAAGCCCCCACGGGGGCGCCGGAGGAGCGGCCCGCGGCCCATCAGCTTGTAGGCGGTGCAAGGGACCACCTAGGCTACGGACGGGTACCCGGCCTC
>FQH3XDB01DNRJU|559|6
AACGAACGCTGGCGGCGCGTCTTAAGCATGCAAGTCGGGCGGGATCCATGCGCTTGCGCATGGTGAGAGCGGCGGACTGGCGAGTAACACGTGGGCGACGCGCCCTCCGGACGGGAATAGCCTGTAGAAATACAGGGGTAATGCCGGATGCGAACGCACGGGCTGGAGCCGTGCGTGGAAAGCCCCCACGGGGGCGCCGGAGGAGCGGCCCCGCGGCCCATCAGCTTGTAGGCGGTGCAAGGGACCACCTAGGCTACGACGGGTACCCGGCCTCAGAGGGCGGACGGGCGCATTGGGACTGAGATACGGCCCAGACTCCTACGGGGAGGCAGCAGCTAAGATATGCGCAATGGAGGAAACTCTGACGCAGTGACGCCGCGTATAGGAAGAAGGTTTTCGGATTGTAAACTATTGTCGTGAGG
>FQH3XDB01A5UZG|560|4
AACGAACGCTGGCGGCGCGTCTTAAGCATGCAAGTCGGGCGGGATCCATGCGCTTGCGCATGGTGAGAGCGGCGGACTGGCGAGTAACACGTGGGCGACGCGCCCTCCGGACGGGAATAGCCTGTAGAAATACAGGGGTAATGCCGGATGCGAACGCACGGGGCTGGAGCCGTGCGTGGAAAGCCCCCAGGGGGCGCCGGAGGAGCGGCCCGCGGCCCATCAGCTTGTAGGCGGTGCAAGGGGACCACCTAGGCTACGACGGGTACCCGGCCTCAGAGGGCGGACGGGCGCATTGGGACTGAGATACGGCCCCAGACTCCTACGGGAGGCAGCAGCTAAGAT
>FQH3XDB01DHM3T|561|2
AACGAACGCTGGCGGCGCGTCTTAAGCATGCAAGTCGGGCGGGATCCATGCGCTTGCGCATGGTGAGAGCGGCGGACTGGCGAGTAACACGTGGGCGACGCGCCCTCCGGACGGGAATAGCCTGTAGAAATACAGGGTAATGCCGGATGCGAACGCACGGGGCTGGAGCCGTGCGTGGAAAGCCCCCACGGGGGGCGCCCGGGAGGGAGCGGGCCCCGCGGGCCCCATCAGCTTGTAGGCGGTGCAAGGGGACCACCTAGGCTACGACGGGTACCCGGCCTCAGAGGGCGGACGGGCGCATTGGGACTGAGATACGGCCCAGACTCCTACGGGGAGCAGCAGCTAAGAATATTCCGCAAGTGGGGGGAACCCCTGACGGAGCGACGCCGCGT
>FQH3XDB01EE5TV|562|1
AACGAACGCTGGCGGCGCGTCTTAAGCATGCAAGTCGGGCGGGATCCATGCGCTTGCGCATGGTGAGAGCGGCGGACTGGCGAGTAACACGTGGGCGACGCGCCCTCCGGACGGGAATAGCCTGTAGAAATACAGGGTAATGCCGGATGCGAACGCACGGGCTGGAGCCGTGCGTGGAAACGCCCCCGACGGGGGGCGCCGGGAGGAGCCGGCCCGCGGCCCATCAGCTTGTAGGCGGTGCAAGGGACCACCTAGGCTACGACGGGTACCCGGCCTCAGGAGGGCGGACGGGCGCAGTTGGGACTGAGATACCGGCCCAGACCTCCGTACGGGAGGCAGCAGC
>FQH3XDB01EN99I|563|3
AACGAACGCTGGCGGCGCGTCTTAAGCATGCAAGTCGGGCGGGATCCATGCGCTTGCGCATGGTGAGAGCGGCGGACTGGCGAGTAACACGTGGGCGACGCGCCCTCCGGACGGGAATAGCCTGTGGAAAACACAGGGGTAATACCGGACAAGCCGCAGCGGGCTGGAGCCGCTGCGGGAAAGGCGCCACGGCGCCGCCGGAGGATCGGCCCCGCGTCCCATCAGCTAGCAGGCGGGTAAAGGCCCACCTGGGCGATGACGGGTATCCGGCCTGAGAGGGTGTACGGACACATTGGGACTGAGATACGGCCCAGACTCCTACGGGAGCAGCAGCTAAGATATCG
>FQH3XDB01C0US4|564|1
AACGAACGCTGGCGGCGCGTCTTAAGCATGCAAAGTCGAACGGGAACTAGGAGCTTGCTTCTAGCGAGAGTGGCGGACTGGTGAGTAACACGTGGGTGACGTACCCCTAAGGATGGGGATAGCCTGTAGAAATACGGGGTAATACCGAATACGCTCGTTGCAGTAAAGAGCGATGAGGAAAGATGCTACGGCATCACCTAAGGAACGGCCCGCGGACTATTAGCTAGTTGGTATGGTAACGGCATACCAAGGCGACGATAGTTATCC
>FQH3XDB01AOTDE|565|1
AACGAACGCTGGCGGCGCGTTTCAAGCATGCAAGTCGAGCGAATAGGCCTTCGGGCCTGGAAGCGGCGGACGGGTGAGTAACACGTGGGTAATCTGCCCTCCGGTGGGGAATAGCACATGGAAACATGTGATAATACCGCATAATACGGTCTGCCAGAAGGCAGGCTGGGAAAGGTCCTGCGGGACCGCCGGGGGATGAGCCCGCGGCCCATTAGCCAGTTGGCGGGTAACGGCCCACCAAAGCGACGATGGGTAGCCGGCCTGAGAGGGTGAACGGCCACATTGGGAATTGAGAACGGTCCAGACTCCTACGGGAGGCAGCAGCTAAGAATCTTCCGCAATGGGGGAAACCCTGACGGAGCGACGCCGCGTGAACGATGAAGGCCGTAAGGTTGTAAAGTTCTTTTCGGGGAGGAGGAATAAGGCGCTCAGGGAATGGGGTGCCGATGACGTGAAT
>FQH3XDB01B459T|566|29
AACGAACGCTGGCGGCGCGTTTTAAGCATGCAAGTCGAGCGGCAAGTGCAGCGATGCACCTAGAGCGGCGGACGGGTGAGTAACACGTGGATAATCTGCCCCGCAGTCGGGGATAGCCTGTGGAAACACAGGATAATACCGGATGAGCTTCGTGAGGCACAAGCCTTGCGAAGGAAAGGAGCCACGGCTCCGCTGAGGGATGAGTCCGCGGCCCATTAGCTGGATGGCGGGGTAAAGCCCACCATGGCGATGATGGGTAGCCGGCCTGAGGAGGGTGGACGGCCACATTGGAAACTGAGACACGGTCCAGACTCCTACGGGAGCAGCAGCTAAGATCTTCCACAATGGACGAAAGTCTGATGGAGCGACGCCGCGTGGACGACGAAGGCCGTGAGGTTGT
>FQH3XDB01B2VI2|567|1
ATTACCGCGGCTGCTGCCTCCCGTAGGAGTCTGGACCGTGTCTCAGTTCCAATGTGGCCGTCCACCCTCTCAGGCCGGCTACCCATCATCGCCATGGTGGGCCTTTACCCGCCATCCAGCTAATGGGCCGCGGACTCATCCCTCAGCGGAGCCGTGGCTCCTTTCCTTCCCAGGCCTTGTGGCCCGGGAAGCTCATCCGGTATTATCCTGTGTTTCCACAGGCTATCCCCGACTGCGGGGCAGATTATCCACGTGTTACTCACCCGTCCGCCGCTCTAGGTGCATCGCTGCACTTGCCGCTCGACTTGCATGCTTAAAACGCGCCGCCAGCGTTCGTT
>FQH3XDB01AV5Z8|568|2
AACGAACGCTGGCGGCGCGTTTTAAGCATGCAAGTCGAGCGGCAAGTGCAGCGATGCACCTAGAGCGGCGGACGGGTGAGTAACACGTGGATAATCTGCCCCGCAGTCGGGGATAGCCTGTGGAAACACAGGATAATACCGGATGAGCTTCGTGAGGCACAAGCCTTGCGAAGGAAGGAGCCACGGCTCCGCTGAGGGATGAGTCCGCGGCCCATTAGCTGGATGGCGGGGTAAAGGCCCACCATGGCGATGATGGGGTAGCCGGCCTGGAGGAGGGTGGACGGCCACATTGGAACTGAGACACGGTCCAGACCTCCGTACGGGGAGGCAGCAGCTAAGATCTCCACATGGACGAAAGTCTGAGTGGAGCGACGCCGCGTGGACGACGAGGCCGTGAGGTTGTAAGTCCGTTCGGAGGGACACC
>FQH3XDB01BW4JB|569|6
AACGAACGCTGGCGGCGCGTTTTAAGCATGCAAGTCGAGCGGCAAGTGCAGCGATGCACCTAGAGCGGCGGACGGGTGAGTAACACGTGGATAATCTGCCCCGCAGTCGGGGATAGCCTGTGGAAACACAGGATAATACCGGATGAGCTTCGTGAGGCACAAGCCTTGCGAAGGAAAGGAGCCACGGCTCCGCTGAGGGATGAGTCCGCGGCCCATTAGCTGGATGGCGGGTAAAGGCCCACCATGGCGATGGATGGGTAGCCGGCCTGGAGAGGGTGGACGGCCACATTGGAACTGAGACACGGTCCAGACCTCCGTACGGGAGCAGCAGCTAAG
>FQH3XDB01DHJSO|570|8
AACGAACGCTGGCGGCGCGTTTTAAGCATGCAAGTCGAGCGGCAAGTGCAGCGATGCACCTAGAGCGGCGGACGGGTGAGTAACACGTGGATAATCTGCCCCGCAGTCGGGGATAGCCTGTGGAAACACAGGATAATACCGGATGAGCTTCGTGAGGCACAAGCCTTGCGAAGGAAAGGAGCCACGGCTCCGCTGGGGATGAGTCCGCGGCCCATTAGCTGGATGGCGGGGTAAAGGCCCACCATGGCGATGATGGGTAGCCGGCCTGAGAGGGTGGACGGCCACATTGGAACTGAGACACGGTCCAGACCTCC
>FQH3XDB01A9OUF|571|1
AACGAACGCTGGCGGCGCGTTTTAAGCATGCAAGTCGAGCGGCAAGTGCAGCGATGCACCTAGAGCGGCGGACGGGTGAGTAACACGTGGATAATCTGCCCCGCAGTCGGGGATAGCCTGTGGAAAACACAGGATAATACCGGATGAGCTTCGTGGGGCACAAGCCTTGCGAAGGAAAGGAGCCACGGCTCCGCTGAGGGATGAGTCCGCGGCCCATTAGCTGGATGGCGGGTAAAGCCCACATGGCGATGATGGGGTAGCCGGCCTGGAGAGGGTGGACGGCCACATTGGAACTGAGACACGGTCCAGACCTCCGTACGGGA
>FQH3XDB01AY75Z|572|1
AACGAACGCTGGCGGCGTGCCTAACACATGCAAGTCGAACGAGAAAGCCCGCAAGGGTAAGTAGAGTGGCGAACGGGTGAGTAATGAATAGGAACATACCTATGGGTGGGGGATAACCAAGGGAAACTTTGGCTAATACCGCATGTGATCGCAAGATCAAAGGGGGCGTCGGGTAACCGAGCTTTCGCCGATAGAGTGGCCTATTTCCCATTAGCTAGTTGGTAGGTAAGCCTACCAAGGCTACGATGGGTAGCTGGTCTGAGAGGACGATCAGCCACACTGGAACTGAGACACGGTCCAGACTCCTACGGGAGCAGCAGTGGGGAATCTTGCACAAGTGGGCGAAAGCCTGATGCAGCAACGCGCGTGAGT
>FQH3XDB01D9V1Z|573|1
GAACGAACGCTGGCGGCGTGCCTAACACATGCAAGTCGAACGAAGGAATCGGGATTCGTCCGGTTCTTAGTGGCGGACGGGTGAGTAACGCGTGAGAAACCTGCCTTTCAGAGGGGGATAACGTTTGGAAACGAACGCTAATACCGCATAACGCAGCGGATTCGCATGAATCTGCTGCCAAAGGAGCGATCCGCTGAAAGATGGTCTCGCGTCCGATTAGCTAGTTGGTGAGATAACAGCCCACCAAGGCGACGATCGGTAGCCGGACTGAGAGGTCGAACGGCCACATTGGGACTGAGACACGGCCCCAGACTCCTACGGGAGGCAGCAGTGGGGGATATTGCACAATGGAGGAAACTCTGATGCAGCAACGCCGCGTGAGGGAAGAAGGATTTCGGTTTGTAAACCTCTGTCTTCGGTGAC
>FQH3XDB01EKDXR|574|26
GACGAACGCTGGCGGCGTGCCTAACACATGCAAGTCGAACGGAGTTAAGCCCTTCGGGACTTAACTTAGTGGCGAACGGGTGAGTAACGCGTGAGGAACCTGCCTTTCAGTGGGGGACAACAGTTGGAAACGACTGCTAATACCGCATGATACTTTTGGGAGACATCTCCTGGAAGTCAAAGCTTTATGTGCTGAAAGATGGCCTCGCGTCTGATTAGCTGGTTGGTGAGGTAACGGCTCACCAAGGCGACGATCAGTAGCCGGTCTGAGAGGATGAACGGCCACATTGGGACTGAGATACGGCCCAGACTCCTACGGGAGGCAGCAGTGGGGAATATTGGGGCAAATGGGGGAAACCCTGACCCAGCAACGCCGCGTGAAGGAAGAAGGCCTTCGGGTTGTAAACTTCTTTTACCAGGACGAAGGACGTGACGGTACCTGGAGAAAAAGCAAACGGTC
>FQH3XDB01AQ640|575|13
GACGAACGCTGGCGGCGTGCCTAACACATGCAAGTCGAACGGAGCAAGCCCCTTCGGGGACTTACTTAGTGGCGAACGGGTGAGTAACGCGTGAAGAACCTGCCTTTCAGTGGGGGACAACAGTTGGAAACGACTGCTAATACCGCATAATGCAGCGAGGGGGCATCCCATTGCTGCCAAAGATTATTGCTGAAAGATGGCTTCGCGTCTGATTAGCTAGTTGGTGAGGTAACGGCTCACCAAGGCGACGATCAGTAGCCGGTCTGAGAGGATGAACGGCCACATTGGGACTGAGATACGGCCCAGACTCCTACGGGAGGCAGCAGTGGGGAATATTGGGCAATGGGGGAAACCCTGACCCAGCAACGCCGCGTGAAGGAAGAAGGCCTTCGGGTTGTAAACTTCTTTAGTAGGACGAAGAACGTGACGGTACCTACAGAAAAGCAACGGCTAACTACGTGCCAGCAGCCG
>FQH3XDB01DE3WE|576|10
GACGAACGCTGGCGGCGTGCCTAACACATGCAAGTCGAACGGAGATAAGAATTTCGGTTTTATCTTAGTGGCGCACGGGTGAGTAACACGTGAATAACCTGACCCAAAGAGGGGATAACACCTGGAAACAGGTGCTAATACCGCATAAGACCACAATGCCGCATGGCGAAGGGGTCAAAGAAATTCGCTTTGGGAGGGGTTCGCGTCCCATTAGGTAGTAGGCGGGGTAACGGCCCACCTAGCCGACGATGGGTAGCCGAGCTGAGAGGCTGATCGGCCACACTGGAACTGAGACACGGTCCAGACTCCTACGGGAGGCAGCAGTGGGGAATATTGGGCAAGTGGACGCAAGCCTGACCCAGCAACGCCGCGTGAGGGAAGAAGGTTTTCGGATT
>FQH3XDB01B7PU2|577|1
AACGAACGCTGGCGGCGTGCTTAACACATGCAAGTCGAGTGCGCGGTTGTAGCAATACAACTGGCGCACGGCGCACGGGTGCGTAACACGTGGGTAACCTGCCCTTTGGTGGGGAATAACTCCGCGAAAGCGGAGCTAATTCCGCATAAGATCCGCAAGGATGAAAGCCGCAAGGCGCCGAAGGAGGGGCCCCGCGCCCCGATTAGCTAGTTGGTAGGGTAACGGCCTACCAAGGCTTTGATCGGTAGCTGGTCTGAGAGGATGATCAGCCACACTGGAACTGAGACACGGTCCAGACTCCTACGGGAGGCAGCAGTGGGGAATCTT
>FQH3XDB01B9X85|578|1
GAACGAACGCTGGCGGCGTGCTTCATACATGCAAGTCGAACGAGAACCTAACTTCGGTTGGGGGAAAGTGGCGGACGGGTGAGTAATATGTAGAGAATCTGCCCTAGAGCGGGGGACAACAGAGGGAAACTTCTGCTAATACCCCATATGAGCGTATCTGAAATGATATTCTTGAAAACTCCGGTGCTCTAGGATGAGTCTGCATCTGATTAGCTAGGTTGGGGTGTAATGGACCACCAAGGCGACGATCAGTAGCTGGTTTGAGAGGATGATCAGCCACAATGGGACTGAGACACGGCCCATACTCC
>FQH3XDB01AJ8P6|579|86
GACGAACGCTGGCGGCGTGCTTCATACATGCAAGTCGAACGAGAAGCTAGAGCTTGCTCTAGTGGAAAGTGGCGGACGGGTGAGTAATATGTAGGAAATCTGCCCTAGAGAGGGGGACAACAGAGGGAAACTTCTGCTAATACCCCATATGAGCTTGGTTGAAATACCAATCTTGAAAACTCCGGTGCTCTAGGATGAGCCTGCACCTGATTAGCTTGTTGGTGGTGTAATGGGACTACCAAGGCGACGATCAGTAGCTGGTTTTGAGAGGATGATCAGCCACAATGGGACTGAGACACGGCCCATACTCCTACGGGAGCAGCAGTA
>FQH3XDB01A6PW7|580|1
AACGAACGCTGGCGGCGTGGATAAGACATGCAAGTCGAACGGATCTGGTGCTGTAGCAATACAGTGCCGGGTCAGTGGCGCAAGGGTGCGTAACACGTGGGCAACCTGCCCTGGGGCCCGGGATAGCCCGCCGAAAGGCGGATTAATACCGGATGTGGCCAGCCAACGATTGTTGGCGACGCCAAAGGTGGCTGCAAAGCTGCCGCTCCTGGATGGGCCCGCGGCCTATCAGCTTGTTGGTGGGGTAACGGCTCACCAAGGCGATGACGGGTAGCTGGTCTGAGAGGACGACCAGTCACACTGGAACTGAGACACGGTCCA
>FQH3XDB01BOGJK|581|21
AACGAACGCTGGCGGCGTGGATAAGACATGCAAGTCGAACGAGAGAATTGCTAGCTTGCTAATAATTCTCTAGTGGCGCACGGGTGAGTAACACGTGAGTAACCTGCCCCCGAGAGCGGGGATAGCCCTGGGAAACTGGGATTAATACCGCATAGAATCGCAAGATTAAAGCAGCAATGCGCTTGGGGATGGGCTCGCGGCCTATTAGTTAGTTGGTGAGGTAACGGCTCACCAAGGCGATGACGGGTAGCCGGTCTGAGAGGATGTCCGGCCACACTGGAACTGAGACACGGTCCAGACACCTACGGGTGCAGCAGTCGAGAATCATTCACAATGGGGGAAACCCTGATGGTGCGACGCCGCGTGGGGAATGAAGGTCTTCGGATTGTAAACCCCTGTCATGT
>FQH3XDB01CLS5E|582|19
AACGAACGCTGGCGGCGTGGATAAGACATGCAAGTCGAACGAGAGGATTTCTAGCTTGCTAAAGATTCTCTAGTGGCGCACGGGTGAGTAACACGTGAGTAACCTGCCCCCCGAGAGAGGGATAGCCCCGGGAAACTGGGATTAATACCGCATAGTATCGCAAGATTAAAGCAGCAATGCGCTTGGGGATGGGCTCGCGGCCTATTAGTTAGTTGGTGAGGTAACGGCTCACCAAGGCGATGACGGGTAGCCGGTCTGAGAGGATGTCCGGCCACACTGGAACTGAGACACGGTCCAGACACCGTACGGGTGCAGCAGTCGAGAATCATTCACAAGTCGGGG
>FQH3XDB01EWXN3|583|1
AACGAACGCTGGCGGCGCGTCTTAAGCATGCAAGTCGAACGGGAACTAGGAGCTTGCTTCTAGCGAGAGTGGCGGACTGGTGAGTAACACGTGGGTGACGTACCCTAAGGATGGGGATAGCCTGTAGAAATACGGGGTAATACCGAATACGCTCGTTGCAGTAAAGAGCGATGAGGAAAGATGCTACGGCATCACCTAAGGAACGGCCCGCGGACTATTAGCTAGTTGGTATGGTAACGGCATACCAAGGCGACGATAGTTATCCGGCCTAAGAGGGTGAACGGACACATTGGGACTGAGATACGGCCCAGACTCCTACGGGAGGCAGCAGTCGGGAATATTGCGCAATGGAGGAAACTCTGACGCAGTGACGCCGCGTGCAGGAAGAAGGTTTTCGGATTG
>FQH3XDB01BN15C|584|1
GAACGAACGCTGGCGGCGGTGCTTCATACATGCAAGTCGAACGAGAATCTCTAGCTTGCTAGAGAGGAAAGTGGCGGACGGGTGAGTAATGTGTAGAGAATCTGCCCTAGAGGAGGGGGACAACAGAGGGAAACTCCTGCTAATACCCCATATGAGCGTATCTGAAATGGTATTCTTGAAAACTCCGGTGCTCTAGGATGAGTCTGCATCTGATTAGCTAGGTTGGGGGTGTAATGGACCACCAAGGCGACGATCAGTAGCTTGGTTTTGAGGAGGATGATCAGCCACAAGTGGGACTGAGACACGGCCCATA
>FQH3XDB01EQCHO|585|10
AACGAACGCTGGCGGTATGCTTAACACATGCAAGTCGTACGAGAAAGTTCTTCGGAATGAGTAAAGTGGCGTACGGGTGAGTAATGTATAGGAATCTACCCAGTAATGAGGAATAAGCACTGGAAACGGTGTCTAATACCACATACTACCGATTCGTCGGGAAAGATTTAGTCTTCGGATTAAGTTGTTATTGGATGAGCCTATATCTGATTAGCTAGTTGGTGGGTAAAGCCTACCAAGGCGATGATCAGTAGCTGATCTGAGAGGGTGATCAGCCACATTGGGACTGCGACACGGCCCAAACTCCTACGGGAGGCAGCAGTGGGGAATATTGGACAATGGGCGAAAGCCTGATCCAGCAATACCGCGTGAATGATG
>FQH3XDB01AF4HG|586|2
AACGAACGCTGGCGGTATGCTTAACACATGCAAGTCGTACGAGAAAGTTCTTCGGAATGAGTAAAGTGGCGTACGGGTGAGTAATGTATAGGAATCTACCCAGTAATGGGGAATAAGCACTGGAAACGGTGTCTAATACCACATACTACCGATTCGTCGGGAAAGATTTGGCCTTCGGGTTAAGTCGTTATTGGATGAGCCTATATCTGATTAGCTAGTTGGTGGGGTAAAGGCCTACCAAGGCGATGATCAGTAGCTGATCTGAGAGGGTGATCAGCCACATTGGGACTGCGACACGGCCCAAACTCCGTACGGGAGGCAGCAGGTGGGAAT
>FQH3XDB01C2937|587|1
AACGAACGCTGGCGGTATGCTTAACACATGCAAGTCGTACGAGAAAGTTCTTCGGAATGAGTAAAGTGGCGTACGGGTGAGTAATGTATAGGAATCTACCCAGTAATGAGGAATAAGCACTGGAAACGGTGTCTAATACCACATACTACCGATTCGTCGGGAAAGATTTTAGTCTTCGGATTAAGTTGTTATTGGATGAGCCTATATCTGATTAGCTAGTTGGTGGGTAAAGGCCTACCAAGGCGATGATCAGTAGCTGATCTGAGAGGGTGATCAGCCACATTGGGACTGCGACACGGCCCAAACTCCTACGGGAGCAGCAGTGGGGAATATTGGGACAATGGGCGAAAGCCTGATCCAGCAATACCGCGTGAATGATG
>FQH3XDB01C1DIS|588|2
AACGAACGCTGGCGGTATGCTTAACACATGCAAGTCGTACGAGAAAGTTCTTCGGAATGAGTAAAGTGGCGTACGGGTGAGTAATGTATAGGAATCTACCCAGTAATGAGGAATAAGCACTGGAAACGGTGTCTAATACCACATACTACCGATTCGTCGGGAAAGATTTAGTCTTCGGATTAAGTTGTTATTGGATGAGCCTATATCTGATTAGCTAGTTGGTGGGTAAAGGCCTACCAAGGCGATGATCAGTAGCTGATCTGAGAGGGTGATCAGCCACATTGGGACTGCGACACGGCCCAAACTCCTACGGGAGGCAGCAGTGGGGAATATTGGACAATGGGCGAAAGCCTGATCCAGCAATACCGCGTG
>FQH3XDB01EAHH9|589|1
AACGAACGCTGGCGGTATGCTTAACACATGCAAGTCGTACGAGAAAGTTCTTCGGAATGAGTAAAGTGGCGTACGGGTGAGTAATGTATAGGAATCTACCCAGTAATGAGGAATAAGCACTGGAAACGGTGTCTAATACCACATACTACCGATTCGTCGGGAAAAGATTTTGGGCCTTCGGGTTAAGTCGTTATTGGATGAGCCTATATCTGATTAGCTAGTTGGTGGGTAAAGCCTACCAAGGCGATGATCAGTAGCTGATCTGAGAGGGTGATCAGCCACATTGGGACTGCGACACGGCCCAAACTCCTACGGGAGGCAGCAGTGGGGAATATTGGACAATGGGCGAAAGCCTGATCCAGCAAT
>FQH3XDB01ADQJV|590|1
AACGAACGCTGGCGGTATGCTTAACACATGCAAGTCGTACGAGAAAGTTCTTCGGAATGAGTAAAGTGGCGTACGGGTGAGTAATGTATAGGAATCTACCCAGTAATGAGGAATAAGCACTGGAAACGGTGTCTAATACCACATACTACCGATTCGTCGGGAAAGATTAGTCTTCGGATTAAGTTGTTATTGGATGAGCCTATATCTGATTAGCTAGTTGGTGGGGTAAAGGCCTACCAAGGCGATGATCAGTAGCTGATCTGAGAGGGTGATCAGCCACATTGGGACTGCGACACGGCCAA
>FQH3XDB01CS3CE|591|1
AACGAACGCTGGCGGTATGCTTAACACATGCAAGTCGTACGAGAAAGTTCTTCGGAATGAGTAAAGTGGCGTACGGGTGAGTAATGTATAGGAATCTACCCAGTAATGAGGAATAAGCACTGGAAACGGTGTCTAATACCACATACTACCGATTCGTCGGGAAAGATTTAGTCTTCGGATTAAGTTGTTATTGGATGAGCCTATATCTGATTAGCTAGTTGGTGGGGTAAAGGCCTACCAAGGCGATGATCAGTAGCTGATCTGAGAGGGTGATCAGCCACATTGGGACTGCGACACGGCCCCAAAACTCCTACGGGAGGCAGCAGTGGGGAATATTGGACAATGGGCGAAAGCCTGATCCAGCAATACCGCGTGAATGATGAAGGCCTTC
>FQH3XDB01E2GKW|592|1
AACGAACGCTGGCGGTATGCTTAACACATGCAAGTCGTACGAGAAAGTTCTTCGGAATGAGTAAAGTGGCGTACGGGTGAGTAATGTATAGGAATCTACCCAGTAATGAGGAATAAGCACTGGAAACGGTGTCTAATACCACATACTACCGATTCGTCGGGAAAGATTTAGTCTTCGGATTAAGTTGTTATTGGATGAGCCTATATCTGATTAGCTAGTTGGTGGGGTAAAGGCCTACCAAGGCGATGATCAGTAGCTGATCTGAGAGGGTGATCAGCCACATTGGGACTGCGACACGGCCCAAACTCCTACGGGAGGCAGCAGTGGGGAATATTGGACAATGGGCGAAAGCCTGATCCAGCAATACCGCGTGAATGATG
>FQH3XDB01C8HRB|593|4
AACGAACGCTGGCGGTATGCTTAACACATGCAAGTCGTACGAGAAAGTTCTTCGGAATGAGTAAAGTGGCGTACGGGTGAGTAATGTATAGGAATCTACCCAGTAATGAGGAATAAGCACTGGAAACGGTGTCTAATACCACATACTACCGATTCGTCGGGAAAAGATTTTGGGCCTTCGGGTTAAGTCGTTATTGGATGAGCCTATATCTGATTAGCTAGTTGGTGGGTAAAGGCCTACCAAGGCGATGATCAGTAGCTGATCTGAGAGGGTGATCAGCCACATTGGGACTGCGACACGGCCCAAACTCCTACGGGAGGCAGCAGTGGGGAATATTGGACAATGGGCGAAAGCCTGATCCAGCAATACCGCGTGAATGATGAAGGCCTTCGGG
>FQH3XDB01BWX86|594|7
AACGAACGCTGGCGGTATGCTTAACACATGCAAGTCGTACGAGAAAGTTCTTCGGAATGAGTAAAGTGGCGTACGGGTGAGTAATGTATAGGAATCTACCCAGTAATGAGGAATAAGCACTGGAAACGGTGTCTAATACCACATACTACCGATTCGTCGGGAAAGATTTAGTCTTCGGATTAAGTTGTTATTGGATGAGCCTATATCTGATTAGCTAGTTGGTGGGTAAAGGCCTACCAAGGCGATGATCAGTAGCTGATCTGAGAGGGTGATCAGCCACATTGGGACTGCGACACGGCCCAAAACTCCTACGGGAGGCAGCAGTGGGAATATTGGACAATGGGCGAAAGCCTGATCCAGCAATACCGCGTGAATGATGAAGGCCTTCGGGTT
>FQH3XDB01BDQZL|595|1
AACGAACGCTGGCGGTATGCTTTAACCATGCAAGTCGAACGAGAAAGGGTAGCAATACCTGAGTAAAGTGGCAAACGGGTGAGTAACACACAGGAATCTACCCAGTAGAGAGGAATAAGCACTAGAAATGGTGTCTAATACCACATAATACGAGTTTAGAAATAAATTCGGAAAGGGCTAGTCTTCGGATTAGTTCGCTATTGGATGAGCCTATGTCGGATTAGTTAGTTGGTGGGGTAATGGCCTACCAAGACGATGATCCGTAGCTGATCTGAGAGGGTGATCAGCCACACTGGAACTGCGACACGGTCCAGACTCCTACGGGAGGCAGCAGGTGGAATACTTGGACAAGTGGGCGAAAGTCCTGATCCAGCAATACCGCGT
>FQH3XDB01BZRJE|596|20
AACGAACGCTGGCGGTGCGTCTTAAGCATGCAAGTCGAGCGGCAACCCGCCTTCGGGCGGGCCAGAGCGGCGGACTGGTGAGTAACACGTGGGCGACGCGCCCTCCGGATGGGAACAGCCTGCGGAAAACGCAGGGTAATGCCGAATAAGCTCCCGCAAGCCAGAGAGGCGGGAGGAAAGGGGCCTCGGCCCCGCCGGGGGAGCGGCCCGCGCGCCATTAGCTAGTTGGCGGGGCAAAAGCCCACCAAGGCGACGATGGCTATCCGGCCTGAGAGGGTGGACGGACACATTGGGACTGAGATACGGCCCAGACTCCTGCGGAGGCAGCAGCTAAGAATATTCCGCAAGTGGGGGAACCCTGACGGAGCGACACCGCGTGGACGACGAAGGCCGG
>FQH3XDB01CCHEW|597|4
AACGAACGCTGGCGGTGCGTCTTAAGCATGCAAGTCGAGCGGCAACCCGCCTTCGGGCGGGCCAGAGCGGCGGACTGGTGAGTAACACGTGGGCGACGCGCCCTCCGGATGGGAACAGCCTGCGGAAACGCAGGGTAATGCCGAATAAGCTCCCGCAAGCCAGAGAGGCGGGAGGAAAGGGGCCTCGGGCCCCGCCGGGGGAGCGGCCCGCGCGCCATTAGCTAGTTGGCGGGGCAAAAGCCCACCAAGGCGACGATGGCTATCCGGCCTGAGAGGGTGGACGGACACATTGGGGACTGAGATACGGCCCAGACTCCTGCGGAGGCAGCAGCTAAGAATATTCCGCAAGTGGGGGGAACCCCTGGACGGAGCGACACCGCGTGGACGACGAAGGCCGGAAGGTT
>FQH3XDB01ATA7T|598|1
AACGAACGCTGGCGGTCGCGTCTTAAGCATGCAAGTCGGAACGGCAAGAGAGAGCTTGCTCTCTCCTAGAGTGGCGGACTCGGTGAGGAACCGCGTGGGTGACGCACCCTCCTGACCGGGGACAGCTCCTAGAAATAGGAGATAATACCGGATACGCTGCATATAAGTAGAGGATATGCAGGAAAGGAGCTTTGCTCCGCAGGGGAGCGGCCCGCGTGCTATTAGCTTGACGGCGGGTAACGGCCCACCGTGGCGACGATAGCTACCCGGCCTAACGAGGGCAAACGGGCACATTGGGACT
>FQH3XDB01AOSOW|599|22
AACGAACGCTGGTAGCGTGGATTAGGCATGCAAGTCGAACGGGATCCGGAGGGTAGCAATATTCTTCGGTGAGAGTGGCGGACTGGGGAGGAGCACGTGAGCAACCTGCCCTTGGGTGGGGGAAAACCTCTGGAAACGGAGGCTAATACCGCATATGGCGCCGGGCGGCATCGTCCGAGCGCCAAAGGGGGCCGCAAGGCTCCCGCCCGTGGAGGGGCTCGCGCACCATCAGCTCGTTGGCGGGGTAACGGCCCACCAAGGCTCACGGTTAGCTGGTCTGAGAGGATGGCCAGCCCCACTGGGACTGAGATACTGCCCCAGACTCCTACGGGAGGCTGCAGTCGAGGATCATTTGCAATGGGCGAAAGCCTGACAATGCGACGCTGCGTGGAGGATGAAGGCCCTCGGGTCGTAAACTCCTGTCAGTGGGGAACAGGCGAGGTGA
>FQH3XDB01DJBYL|600|5
AACGAACGCTGGTAGCGTGGATTAGGCATGCAAGTCGAACGGGATCCGGAGGGTAGCAATATTCTTCGGTGAGAGTGGCGGACTGGGGAGGAGCACGTGAGCAACCTGCCCTTGGGTGGGGAAAACCTTTGGAAACGGAGGCTAATACCGCATATGGCGCCGGGCGGCATCGTCCGAGCGCCAAAGGGGGCCGCAAGGCTCCGCCGTGGAGGGGCTCGCGCACCATCAGCTCGTTGGCGGGGTAACGGCCACCAAGGCTCACGGTTAGCTGGTCTGAGAGGATGGCCAGCCCCACTGGGACTGAGATACTGCCCCAGACTCCTAC
>FQH3XDB01DJXV6|601|5
AACGAACGCTGGTAGCGTGGATTAGGCATGCAAGTCGAACGGGATCCGGAGGGTAGCAATATTCTTCGGTGAGAGTGGCGGACTGGGGAGGAGCACGTGAGCAACCTGCCCTTGGGTGGGGGAAAACCTCTGGAAACGGAGGCTAATACCGCATATGGCGCCGGGCGGCATCGTCCGAGCGCCAAAGGGGGCCGCAAGGCTCCCGCCGTGGAGGGGCTCGCGCACCATCAGCTCGTTGGCGGGTAACGCCCACCAAGGCTCACGGTTAGCTGGTCTGAGAGGATGGCCCAGGCCCCCA
>FQH3XDB01DYKE6|602|24
AACGAACGCTGGTAGCGTGGATTAGGCATGCAAGTCGAACGGGATCGGACGGGTAGCAATATTCGTCCGTGAGAGTGGCGGACTGGGGAGGAGCACGTGAGCAACCTGCCCTTGGGTGGGGGAAAACCTCTGGAAACGGAGGCTAATACCGCATATGGCGCCGGGCGGCATCGTCCGAGCGCCAAAGGGGGCCGCAAGGCTCCCGCCGTGGAGGGCTCGCGCACCATCAGCTCGTTGGCGGGGTAACGGCCCACCAAGGCTCACGGTTAGCTGGTCTGAGAGGATGGCCAGCCCCACTGGGACTGAGACACTGCCCAGACTCCTACGGGAGGCTGCAGTCGAGGATCATTTGCAATGGGC
>FQH3XDB01DI0UO|603|4
AACGAACGCTGGTAGCGTGGATTAGGCATGCAAGTCGAACGGGATCGGACGGGTAGCAATATTCGTCCGTGAGAGTGGCGGACTGGGGAGGAGCACGTGAGCAACCTGCCCTTGGGTGGGGGAAAAACCTCTGGAAACGGAGGCTAATACCGCATATGGCGCCGGGCGGCATCGTCCGAGCGCCAAAGGGGGCCGCAAGGCTCCGCCGTGGAGGGCTCGCGCACCATCAGCTCGTTGGCGGGGTAACGGCCCACCAAGGCTCACGGTTAGCTGGTCTGAGAGGATGGCCAGCCCCACTGGGACTGAGACACTGCCCCAGACTCCTACGGGAGGCTGCAGTCGAGGATCATTTGCAATGGGC
>FQH3XDB01EI0L1|604|1
AACGAACGCTGGTAGCGTGGATTAGGCATGCAAGTCGAACGGGATCGGACGGGTAGCAATATTCGTCCGTGAGAGTGGCGGACTGGGGAGGAGCACGTGAGCAACCTGCCCTTGGGTGGGGGAAAACCTCTGGAAACGGAGGCTAATACCGCATATGGCGCCGGGCGGCATCGTCCGAGCGCCAAAGGGGGCCGCAAGGCCTCCCGCCGTGGAGGGCTCGCGCACCATCAGCTCGTTGGCGGGTAACGCCACAAGGCTCGACGGTTAGCTGGTCTGAGAGGATGGCCCA
>FQH3XDB01C41BM|605|2
AACGAACGCTGGTAGCGTGGATTAGGCATGCAAGTCGGACGGGATCCGGCGTGTAGCAATACAGGCCGGTGAGAGTGGCGGAAGGGCGAGGAACACGTGGGCAACCTGTCACGGAGGTGGGGAAAACCGCTGGAAACGGCGGCTAATACCGAATGCGGCGCCCGTCGGCATCGACGGAGCGCCGAAAGGGGCGAAAGCTCCCCCTCCCGAGGGGCCCGCGCACCATCAGCCTGCTGGCGGGTAACGGCCACCAGGGCTTACGGTTAGCT
>FQH3XDB01B9I5Y|606|8
AACGAACGCTGGTAGCGTGGATTAGGCATGCAAGTCGGACGGGATCCGGCGTGTAGCAATACAGGCCGGTGAGAGTGGCGGAAGGGCGAGGAACACGTGGGCAACCTGTCACGGAGGTGGGGAAAACCGCTGGAAACGGCGGCTAATACCGAATGCGGCGCCCGTCGGCATCGACGGAGCGCCAAAGGGGGCGAAAGCCTCCCCCTCCCCGGAGGGGCCCGCGCACCATCAGCCTGCTGGCGGGTAACGGCCCACCAGGGCTTACGGTTAGCTGGTCTGAGAGGATGGCCAGCCGCACTGGGACTGAGATACTGCCCCAGACCTCCTACGGGAGGCTGCAGTCGAGGATCATTTTGCAATGGGCAAAGCCTGACAATGCGACGCTGCGTGG
>FQH3XDB01BOPJ7|607|5
AACGAACGCTGGTAGCGTGGATTAGGCATGCAAGTCGGACGGGATCCGGCGTGTAGCAATACAGGCCGGTGAGAGTGGCGGAAGGGCGAGGAACACGTGGGCAACCTGTCACGGAGGTGGGGAAAACCGCTGGAAAACGGCGGCTAATACCGAATGCGGCGCCCGTCGGCATCGACGGAGCGCCAAAGGGGGCGAAAGCTCCCCTCCCGAGGGGCCCGCGCACCATCAGCCTGCTGGCGGGTAACGGCCCACCAGGGCTTACGGTTAGCT
>FQH3XDB01DKOAO|608|1
AACGAACGCTGGTAGCGTGGATTAGGCATGCAAGTCGGACGGGATCCGGCGTGTAGCAATACAGGCCGGTGAGAGTGGCGGAAGGGCGAGGAACACGTGGGCAACCTGTCACGGAGGTGGGGAAAACCGCTGGAAACGGCGGCTAATACCGAATGCGGCGCCCGTCGGCATCGACGGAGCGCCGAAAGGGGGCAAAGCTCCCCTCCCGAGGGCCCCGCGCACCATCCAGCCTGCTGGCGGGTAACGGCCCACCAGGGCTTACGGTTAGCTGGTCTGAGAGGATGGGCCAGCCG
>FQH3XDB01DFF25|609|1
AACGAACGCTGGTAGCGTGGATTAGGCATGCAAGTCGGACGGGATCCGGCGTGTAGCAATACAGGCCGGTGAGAGTGGCGGAAGGGCGAGGAACACGTGGGCAACCTGTCACGGAGGTGGGGAAAACCGCTGGAAACGGCGGCTAATACCGAATGCGGCGCCCGTCGGCATCGACGGAGCGCCGAAAGGGGGCGAAAGCCTCCCCCGTCCCGGAGGGGCCCGCGCACCATCAGCCTGCTGGCGGGTAACGCCCACAGGGCTTACGGTTAGCTGGTCTGAGAGGATGGCCAGCCGCACTGGGACTGAGATACTGCCCCAGACCTCCGTACGGGGAGCTGCAGTCGAGGATCATTTGCAAGTGGGCGAAAGCCTGACAAT
>FQH3XDB01CANVV|610|1
AACGAACGCTGGTAGCGTGGATTAGGCATGCAAGTCGGACGGGATCCGGCGTGTAGCAATACAGGCCGGTGAGAGTGGCGGAAGGGCGAGGAACACGTGGGTGACGTACCCTAAGGATGGGGATAGCCTGTAGAAATACGGGGTAATACCGAATACGCTCGTTGCAGTAAAGAGCGATGAGGAAAGATGCTACGGCATCACCTAAGGAACGGCCCGCGGACTATTAGCTAGTTGGTATGGTAACGGCATACCAAGGCGACGATAGTTATCCGGCCTAAGAGGGTGAACGGACACATTGGGACTGAGATACGGCCCAGACTCCTACGGGAGGCAGCAGCTAAGAATATTTCGCAATGGGGGGAACCCTGACGGAGCGACGCCGCGTGGATGATGAAGGCCGGAAGGTTG
>FQH3XDB01CR8VC|611|1
AACGAACGCTGGTGGAGTGTCTTATACATGCAAGTCGAGCGAGGACGTAGCGATACGAGCCGAGCGGCGAATGGGTGAGTAACGCGTAAGCAACCTGCCCCGCACACCGGAACAACCGTGCCAACGCGCGGCTAATGCCGGGAGCCGTGGTTCCCCGCATGGGGGATTGACGAAAGATTTATCGGTGCGGGATGGGCTTGCGTCCGATTAGCTAGTTGGCGGGGCAACGGCCCACCAAGGCGACGATCGGTAGCCGAACTGAGAGGTTGAACGGCCACATTGGAACTGAGAAACGGTCCAGACTCCTACGGGAGGCAGCAGTGGGGAATAGTTGGGCAATGGGCGAAAGCCTGACCCAGCGACGCCGCGTGAGGG
>FQH3XDB01AI8AE|612|1
AACGAACGCTGGTGGAGTGTCTTATACATGCAAGTCGAGCGAGGACGTAGCGATACGAGCCGAGCGGCGAATGGGTGAGTAACGCGTAAGCAACCTGCCCGCACACCGGAACAACCGTGCCAACGCGCGGCTAATGCCGGGAGGCCGTGGTTCCCCGCCATGGGGGAGTTGACGAAAGATTATCGGTGCGGGAGTGGGCGTTGCGTCCGATTAGCTAGTTGGCGGGGCGAACGGCCCGACCAAGGCGACGATCGGTAGCCGGCCTGAGAGGGCGGATCGGCCACATTGGGACTGAGAGACGGCCCAGACTCCTACGGGAGGCAGCAGTAGGGAATATTGCGCAATGGGGGCAACCCTGGACGCAGCAACTCCACGTGTGGGATGAAGCATTTTCGGTGTGTAAACTTCTATCAGCAGGGAAGATAGTGACGGTACCTGACTAAGAAGCCCCGGCTAACTACGTGCCAGCAGCCGCGTAATCGTATGACGT
>FQH3XDB01A2T4D|613|18
AACGAACGCTGGTGGAGTGTCCTTATACATGCAAGTCGAGCGAGGACGCGGCAACGCGAGCCGAGCGGCGAATGGGTGAGTAACGCGTAAGCAACCTGCCCCGCATTCCGGAACAACCGTGCCAACGCGCGGCTAATGCCGGGAGCCGTGGCGCCCCGCATGGGGCGTTGACGAAAGATTCATCGATGCGGGATGGGCTTGCGTCCGATTAGCTAGTTGGCGGGGCGACGGCCCACCAAGGCGACGATCGGTAGCCGGCCTGAGAGGGCGATCGGCCACATTGGGACTGAGAGACGGCCCCAGACTCCTACGGGAGGCAGCAGTAGGGAATATTGCGCAAGTGGGGGCAACCCTGACGCAGCAACTCCACGTGTGGGATGAAGCATTTCGG
>FQH3XDB01CHS3G|614|1
AACGAACGCTGGTGGAGTGTCTTATACATGCAAGTCGAGCGAGGACGTAGCGATACGAGCCGAGCGGCGAATGGGTGAGTAACGCGTAAGCAACCTGCCCCGCACACCGGAACAACCGTGCCAACGCGCGGCTAATGCCGGGAGCCGTGGTTCCCCGCATGGGGGATTGACGAAAGATTTATCGGTGCGGGATGGGCTCGCGTCTGATTAGCTGGTTGGTGAGGTAACGGCCCACCAAGGCGACGATCAGTAGCCGTACTGAGAGGTAGAACGGCCACATTGGGGACTGAGACACGGCCCAGACTCCTACGGGAGGCAGCAGTGGGGAATATTGCACAATGGAGGAAACTCTGATGCAGCGATGCCGCGTGAGGGAAGAAGGTTTTCGGATTGTAAACCTC
>FQH3XDB01CML2Z|615|1
AACGAACGCTGGCCGGCACGCTTAACACATGCAAGTCGGACGGTGGTAGTCTTCGGGCTATCATAGTGGCGGACGGGTGAGTATAATGCAGGGATCTACCTATGGGTTTGGGATAGCATTGGGAAACTGATGGTAATACCGGATAAGTTGGAGACAAGAAAGGTGAGAACCGCCGATAGAGGAGCCTGTATCCGATTAGATAGTTGGAGGTGTAAAGGACCCCCAAGTCGATGATCGGTAGCTGTTCTGAGAGGAAGATCAGCCACATTGGGACTGAGATACGGCCCAAAACTCCTACGGGAGGCAGCAGTGGGGAATATTGGGACAATGGGGGCAACCCTGATCCAGCGATGCC
>FQH3XDB01EGM2J|616|3
AACGAACGTTAGCGGCGCGCCTAACACATGCAAGTCGAGCGAGAAAGGGAGCAATCCCCGGTACAGCGGCGCACGGGTGAGTAACACGTAGGTAATCTGCCTCTGAGTGGGGGATAACCTTCCGAAAGGAGGGCTAATACCGCATAAGACCACGACTTCGCGAGGAGAAGGGGTCAAAGCCGGCCACTATACATAAGCTGGCGCTTGGAGATGAGCCTGCGGCCCATCAGCTAGTTGGTAGGGTAATGGCCTACCAAGGCGAAGACGGGTAGCTGGTCTGAGAGGATGATCAGCCACACTGGAACTGAGACACGGTCCAGACTCCTACGGGAGGCAGCAGTGGGGAATCTTGCGCAATGGGCGAAAAGCCTGACGCAGCGACGCCGCGTGGGTGATGAAGGCCTTCGGGTGTAAAGCCCTGTGGAGGGGGAAGAATAAGTCTTGGCTAACATCCAGATGATGACGG
>FQH3XDB01CACZT|617|1
AACGAACGTTAGCGGCGCGCCTAAGACATGCAAGTCGAGCGAGAAAGGGATTCGTCCCCGGTACAGCGGCGCACGGGTGAGTAACACGTAGGTCATCAACCCCCGAGTGGTGGATAACTCTCCGAAAGGAGAGCTAATACAGCATGACGGCAGCAATGTCCAAAGCGGGCCTCTTCATGAAAGCTCGCGCTTGGGGATGAGCCTGCGGCCCATCAGGTAGTTGGCGGAGTAACAGCCCACCAAGCCAAAGACGGGTAGCTGGTCTGAGAGGATGAACAGCCACACTGGAACTGAGACACGGTCCAGACTCCTACGGGAGGCAGCAGTCGGGAATCTTCCGCAATGGGCGAAAGCCTGACGGAGCGACGCCGCGTGAGTGATGAAGGCCTTGGTGTAAAGCTCTGTGGGGAGAGAAGAATAAGTGCAGGCTAACACTCTGGCATGATGACGGTATCTCCTTAGCAAG
>FQH3XDB01DOWG2|618|1
AACGAACGCTGGCAGCGTGGATTAGGCATGCAAGTCGAACGGGATCGGACGGGTAGCAATATTCGTCCGTGAGAGTGGCGGAAGGGCGAGGAACACGTGGGCAACCTGTCCTTGAGGTGGGAAAACCGCTGGAAACGGCGGCTAATACCGCATAAGCGCACGAGATCGCATGATTTCGTGTGAAAAACTCCGGTGGTACAAGATGAACCCGCGTCTGATTAGCTAGTTGGTGAGGTAACGGCCCACCAAGGCGACGACCAGTAGCCGGCCTGAGAGGGTGAACGGCCACATTGGGACTGAGACACGGCCCAAAACTCCTACGGGAGGCAGCAGTGGGGAATATTGCACAATGGGGAAACCCTGATGCAGCGACGCCGCGTGAGTGAAGAAGTATT
>FQH3XDB01B28L0|619|1
GACGAACGCTGGCGGCGCGCCTAACACATGCAAGTCGAACGGAGTAAATTTCTCACTGAGTCTTTTGGGCGGTTAGCCGGGAGCGCTAACGATGCGCAAGCATCGTTCAGGCGATTCCAGTAAGCCTAACGGTGCTCGGAAGACCGAGTGAGGAAATTTACTTAGTGGCGAACGGGTGAGTAACGCGTGAGGAACCTGCCTCAAAGAGGGGGACAACAGTTGGAAACGACTGCTAATACCGGCATAAGCCCACGGTCTCGCATGAGACAGAGGGAAAAGGATTTTATCCGCTTTTGAGATGGCCTGCGTCCGATTAGCTAGTTGGTGAGGTAACGGCCACCAAGGCGACGATCGGGTAGCCGGACTGAGAGGTTGAACGGCCACA
>FQH3XDB01CKEZC|620|1
GACGAACGCTGGCGGCGTGCTTAATACATGCAAGTCGAACGGAGTTAGTTGCTACACTAAACAATTTTAGATAAGTTGTTTAGTGTAGCGATTGACTTAGTGGCGGACGGGTGAGTAACGCGTGAGTAATCTGCCTTATACAGGGGGACAACAGTTAGAAATAATTGCTAATACCGCATATGACCACAGCATCGCATGGTGCAGGGGTGAAAGGAGCAATCCGGTATAAGATGAGCTCGCGTATCATTAGCTTGTTGGTGGGGTAATGGCCTACCAAGGCGACGATGGTTAGCCGACTGAGAGGGTGATCGGCCACACTGGAACTGAGATACGGT
>FQH3XDB01CI804|621|1
AACGGACGCTGGCGGCAGGCTTAACACATGCAAGTCAAGGGGGTGTAGCAATACACAACCGGCGCACGGGTGAGTAACGCGTGGGAATATGTCCATTTGTGGGGGATAGCTTCTGGAAACGGAGGTAATACCGCATAAGCCCTGAGGGGGAAAGATTTATCGCGAATGGAGTGGCCCGCGTTGGATTAGTTAGTTGGTTAGGTAAAGGCTGACCAAGGCGAGGATCCATAGCTGGTCTGAGAGGACGATCAGCCACATTGGGACTGAGACACGGCCCCAGACTCCTACGGGAGGCAGCAGTAAGGAATATTGGACAATGGGGGCAACCCTGATCCAGCCATGCCGCGTGAGTGAAGAAGGCCTTCGGGTTGTAAAATCTCTTTTAGAG
>FQH3XDB01DPZP5|622|3
GACAAACGCTGGCGGCGTGCATAAAACATGCAAGTCGAACGGAGGATAGGTGTTGCACTAATTAATCATACAGTGGTTAATTAGTGCAATGCTTACCCTTAGTGGCGGACGGGTGAGTAACGCGTGAGTAATCTGGCTCTGTCTGGGGGATAACAGTTAGAAATGACTGCTAATACCGCATAAGACCACGGGCACGAAGGTGACTGAGGTAAAAGATTTATCGGATAGAGATGAGCTTGCGTTTTCATTAGCTAGTTGGTGGGTAAAGGCCTACCAAGGCGACGATGGATAGCCGATCTGAGAGGATGACCGGCCACACTGGAACTGAGAAACGGTCCAGACTCCTACGGGAGGCAGCAGTTGGGAATATTGGGGCAATGGAAGGAAACTCTGACCCAGCAACGCC
>FQH3XDB01CCMY2|623|1
GATGAACGCTGGCGGCATGCCTAAGACATGCAAGTCGAACGAGATGGCCCACAGATTCAAAAGGAAGATAGAGTGCTTGCACGAAGTTAGAATTTTGATGACGTGGAATTTCCATCTAGTGGCAAACGGGTGAGTAATATGTAGAGAATCTGCCCTAGAGAGGGGGACAACAGTTGGAAACGACTGCTAATACCCCATATGAGCGTATCTGAAATGATATTCTTGAAAACTCCGGTGCTCTAGGATGAGTCTGCATCTGATTAGCTAGTTGGGGGTGTAAATGGACCACCAAAGGCGACGATCAGTAGCTGGTTTGAGAGGATGATCAGCCACAATGGGACTGAGACACGGCCCATACTCCTACGGGAGGCAGCAGTAGGGAATTTTGCGCAAGTGGGGAAACCCTGACGCAGCAACGCCGCGTGATTG
>FQH3XDB01EAUB4|624|1
GATGAACGCTGGCGGCATGCCTAAGACATGCAAGTCGAACGAGATGGCCCACAGATTCAAAAGGAAGATAGAGTGCTTGCACGAAGTTAGAATTTTGATGACGTGGAATTTCCATCTAGTGGCAAACGGGTGAATAACACGTAAACAACCTGCCTTCAGGATGGGGACAACAGACGGAAACGACTGCTAATACCGAATACGTTCCTTGAGTCGCATGACTTAAGGAGAAGGGTGGCCTCTACTTGTAAGCTATCGCCTGAAAAGGGGTTTGCGTCTGATTAGGTAGTTGGTGAGGTAACGGCCCACCAAGCCGACGATCAGTAGCCGGTCTGAGAGGATGAACGGCCACACTGGAACTGAGACACGGTCCAGACTCCTACGGGAGGCAGCAGTGGGAATCTTCGCAATGGGCGAAAGCCGTGACGGAGCAACGCCGCGTGAGTGATGACGCTCGGTGTAAAGCTCTGTGATCGGGGACG
>FQH3XDB01EA5J3|625|2
GATGAACGCTGGCGGCATGCCTAAGACATGCAAGTCGAACGAAGGGACCCAATGAGATTTATAGAAATTTGGAGAGCTTGCTCAAAGAATGGAAATAAATTGATTTGGATTATCCCTTAGTGGCAAACGGGTGAGTAACACGTGGGTTACCTGCCTTCAAGATGGGGATAACAGTTGGAAACGATTGCTAATACCGAATGTGATCTACGGATTAAAGAAGCCTTTAAAAGCTTCGCTTGAAGATGGGCCTGCGGTGCATTAGCTAGTTGGTGAGATAACGGCCCACAAGGCGACGATGCATAGCCGAACTGAGAGGTTAATCGGCCACAGTTGGGACTGAGACA
>FQH3XDB01AD281|626|1
GATGAACGCTGGCGGCGTGCATAACACATTCAAGTCGAACGGTGAAGTGATTTCCACTATGTGGAAATGACTTCGTGAGTATGTTCTGAGGAACATCCTCAGCTGCAAAATATAGTAAAGGCTATATGATGTAGGCGAAGTTAATTCTGTATAGTGGAGGTCACTTCATAGTGGCGGACGGGTGAGTAACGCGTGAGCAACCTACCTGCTAAGGAGGGATAACACAGGGAAACTTGTGCTAATACCGCATGATGTATACTGATCGCATGATTGGTATACCAAAGGGGAAACCGACAGCAGATGGGCTCGCGTTGGATTAGATAGTTGGCAGGGTAACGGCCTACCAAGTACGACGATCAGTAGCCGGACTG
>FQH3XDB01DQQP5|627|1
GATGAACGCTGGCGGCGTGCATAACACATTCAAGTCGAACGGTGAAGTGATTTCCACTATGTGGAAATGACTTCGTGAGTATGTTCTGAGGAACATCCTCAGCTGCAAAATATAGTAAAGGCTATATGATGTAGGCGAAGTTAATTCTGTATAGTGGAGGTCACTTCATAGTGGCGGACGGGTGAGTAACGCGTGAGCAACCTACCTGCTAAGGAGGGATAACACAGGGAAACTTGTGCTAATACCGCATGATGTATACTGATCGCATGATTGGTATACCAAAAGGGAGACCGATAGCAGATGGGCTGCGTTGGATTAGATAGTTGGCAGGTAACGGCTACCAAGTCTGCGATCCATAGCCGAATGAGAGGTTGAACGGCACATTGGAACTGAGAAACGGTCCAGACT
>FQH3XDB01AGMDN|628|1
GATGAACGCTGGCGGCGTGCATAACACATTCAAGTCGAACGGTGAAGTGATTTCCACTATGTGGAAATGACTTCGTGAGTATGTTCTGAGGAACATCCTCAGCTGCAAAATATAGTAAAGGCTATATGATGTAGGCGAAGTTAATTCTGTATAGTGGAGGTCACTTCATAGTGGCGGACGGGTGAGTAACGCGTGAGCAACCTACCTGCTAAGGAGGGATAACACAGGGGAAACTTGTGCTAATACCGCATGATGTATACTGATCGCATGATTGGTATACCAAAGGGAAACCGATAGCAGATGGGCTCGCGTTGGATTAGATAGTTGGCAGGTACGCTACAGTCTGCGATCCATAGCCGAACTGAGAGGTTGAACGGCC
>FQH3XDB01DHS4G|629|1
GATGAACGCTGGCGGCGTGCATAACACATTCAAGTCGAACGGTGAAGTGATTTCCACTATGTGGAAATGGCTTCGTGAGTATGTTCTGAGGAACATCCTCGGCTACAAAATATAGTGTAGGCTGTATGATGTAGGCGAAGTTAATTCTGTATAGTGGAGGTCACTTCATAGTGGCGGACGGGTGAGTAACGCGTGAGCAACCTACCTGCTAAGGAGGGATAACACAGGGAAACTTGTGCTAATACCGCATGATGTATAATGGTCGCATGGCCTATATACCCAAAGGGAACCGATAGCAGATGGGCTCGCGTTGGATTAGATAGTTGGCAGGGTAACGCTACAGTCTGCGATCCATAGCCGAACTGAGAGGTTGAACGGCC
>FQH3XDB01D1Q7B|630|12
GATAAACGCTGGCGGCATGCCTAATACATGCAAGTCAAACGGGAACATAGCAATATGTTCTAGTGGCGAACGGGTGAGTAACACGTAGGCAACCTGTCCATAAGTCGAGGATAACAGTTGGAAACGACTGATAATACTGGATAGTATAAGAGTTTGCATGAATTCTTATTTAAAGATCCGTTTGGATCTCTTATGGAGAGGCCTGCGGTGCATTAGCTAGTTGGTGAGGTAACGGCCCACCAAGGCGACGATGCATAGCTGCGCTGAGAGGCGAAACAGCCACATTGGGACTGAGACACGGCCCAAAACTCCTACGGGAGGCAGCAGTAGGGAATTTTCGGCAAGTGGGGAAACCCTGACCGAGCAATGCCGCGTGAATGATGAAGGTCTTCGGATCGTAAAGTTCTGTTG
>FQH3XDB01A2U73|631|11
AATAAACGCTGGCGGCGTGTCTTAAGCATGCAAGTCGGACGGCAAGGGGGAGCTTGCTCCTCCCTAGAGTGGCGGACTGGTGAGTAACACGTAGGTGACGCGCCCTCCGGACGGGGACAGCTCCTGGAAACAGGAGGTAATACCGGATAAGGCCGCGCGGGCTGGAGCCGCGCGGGGAAAGGCGCTACGGCGCCGCCGGAGGAACGGCCTGCGACCTATCAGCTTGACGGCGGGTAAAGCCCACCGTGGCGATGACAGGTATCCGGCCTGAGAGGGTGAACGGACACATTGGGACTGAGATACGGCCCAGACCTCCGTACGGGAGGCAGCAGCTAAG
>FQH3XDB01A1Q0E|632|6
AATAAACGCTGGCGGCGTGTCTTAAGCATGCAAGTCGGACGGCAAGGGGGAGCTTGCTCCCCCCTAGAGTGGCGGACTGGTGAGTAACACGTAGGTGACGCGCCCTCCGGACGGGGACAGCTCCTGGAAACAGGAGGTAATACCGGATAAGGCCGCGCGGGCTGGAGCCGCGCGGGGAAAGGCGCTATGGCGCCGCCGGAGGAACGGCCTGCGACCTATCAGCTTGACGGCGGGTAAAGCCCACCGTGGCGATGACAGGTATCCGGCCTGAGAGGGTGAACGGACACATTGGGACTGAGATACGGCCCAGACTCCTACGGGA
>FQH3XDB01EHM7X|633|1
AATCAACGCTGGCGGCGTGCCTAACACATGCAAGTCGAACGCGAAAGTCCCGCAAGGGATCAGTAGAGTGGCAAACGGGTGAGTAACGCGTGGGTGACCTGCCTTCGAGCGGGGGATAACGTTCCGAAAGGGACGCTAATACCGCATAACATCCTGTCTTTCAAGAGGCGGAGATCAAAGCCGGGGATCGCAAGACCTGGCACTCGAAGAGGGGCCCGCGTCCGATTAGCTAGTTGGTGAGGTAATGGCTCACCAAGGCTCCGATCGGTATCCGGCCTGAGAGGGCGGACGGACACACTGGGACTGAGACACGGCCCAGACTCCTACGGGAGGCAGCAGTGGGGAATTGTTCGCAATGGGCGCAAGCCTGACGACGCAACGCCGCGTGGAGGATGAAGATCTTCGGGTCGTAAACTCCTTTCGATCGAGACGAACGGCTTCCGGATGAACAATCCGGGAGAGTGACGGTACCGAGAGAA
>FQH3XDB01C2DUZ|634|1
AATCAACGCTGGCGGCGTGCCTAACACATGCAAGTCGAACGCGAAAGTCCCGCAAGGGATAAGTAGAGTGGCAAACGGGTGAGTAACACGTGGGTGACCTGCCTTCGAGCGGGGGATAACGTCCCGAAAGGGACGCTAATACCGCATAACATCCTGCCTTTTAAGAGGTGGAGATCAAAGCCGGGGACCGCAAGGCCTGGCACTTGAAGAGGGGCCCGCGTCTGATTAGCTAGTTGGTGGGGTAATGGCCTACCAAGGCAACGATCAGTATCCGGCCTGAGAGGGCGGACGGACACACTGGGACTGAGACACGGCCCAGACTCCTACGGGAGGCAGCAGTGGGGAATTGTTCGCAATGGGCGCAAGCCTGACGACGCAACGCCGCGTGGAGGATGAAGACC
>FQH3XDB01B1G24|635|2
GATGAACGCTGGCGGCATGCCTAAGACATGCAAGTCGAACGAAGGGACCCAATGACATTTATTGAAGTTTGGAGAGCTTGCTCGAAGAATGGATTTAAATTGATTTGGATCATCCCTTAGTGGCAAACGGGTGAGTAACACGTGGGTTACCTGCCTCCAAGTCGGGGATAACAGTTGGAAACGATTGCTAATACCGGATGTGGTCTACGGATTAAAGAAGCCTTTAAAGCTTCGCTTGGAGATGGGGCCTGCGGTGCATTAGATAGTTGGTGGGGTAATGGCCTACCAAGTCGACGATGCATAGCCGAACTGAGAGGTTAATCGGACCACATTGGGACTGAGACAC
>FQH3XDB01AOKF4|636|2
GATGAACGCTGGCGGCATGCCTAAGACATGCAAGTCGTACGCGAGGGCCCAATGACGTTGAAGGAAGTGGAAAGTGCTTGCACTGGAAATGGAATTCAACCGATTTGGATTTTCCCTCGAGTGGCAAACGGGTGAGTAACACGTGGGTTACCTACCTCTATGTTGGGGATAACAGTTGGAAACGATTGCTAATACCGAATGTGCTCTTCGGAGTAAAGAAGCCCTTAAAGCTTCGCGTAGAGATGGGCCTGCGGCGCATTAGCTAGTTGGTGGGGTAATGGCCTACCAAGGCAACGATGCGTAGCTGAGCTGAGAGGTTGATCGGCCACACGTGGGACTGAGACACGGCCCAGACCTCCG
>FQH3XDB01AZY0J|637|1
GATGAACGCTGGCGGCATGCCTAAGACATGCAAGTCGAACGGAGGGACCCAATGACGTTTTATGAAGTTTTGCGAGCTTGCTCAAAAAATGGATTAAAATCGATTTGGATTATCCCTTAGTGGCAAACGGGTGAGTAACACGTGGGTTACCTGCCTCCAAGTCGGGGACAACAGTTGGAAAACGATTGCTAATACCGGATGTGGACTACGGTTTAAAGAAGCCTTTAAAGCTTCGCTTGGAGATGGGCCTGCGGTGCATTAGCTAGTTGGTGGGATAACGGCCTACCAAGGCGACGATGCATAGCCGAACTGAGA
>FQH3XDB01CZ9Y4|638|2
GATGAACGCTGGCGGCATGCCTAAGACATGCAAGTCGAACGAAGGGACCCAATGAGATTTATAGAAGTTTGGAGAGCTTGCTCAAAGAATGAAAATAATTGATTTGGATTATCCCTTAGTGGCAAACGGGTGAGTAACACGTGGGTTACCCTGCCTTCAAGATGGGGATAACAGTTGGAAACGATTGCTAATACCGAATGTGATCTACGGATTAAAGAAGCCTTTAAAGCTTCGCTTGAAGATGGGCCTGCGGTGCATTAGCTAGTTGGTGAGATAACGGCCCACAAGGCGACGATGCATAGCCGAACTGAGAGGTTAA
>FQH3XDB01CN2GY|639|3
GATGAACGCTGGCGGCATGCCTAAGACATGCAAGTCGAACGAAGGGACCCAATGAGATTTATAGAAGTTTGGAGAGCTTGCTCAAAGAATGGAAATAATTGATTTGGATTATCCCTTAGTGGCAAACGGGTGAGTAACACGTGGGTTACCTGCCTTCAAGATGGGGATAACAGTTGGAAACGATTGCTAATACCGAATGTGATCTACGGATTAAAGAGGCCTTTAAAAGCTTCGCTTGAAGATGGGCCTGCGGTGCATTAGCTAGTTGGTGAGATAACGGCCACCAAGGCGACGATGCATAGCCGAACTGAGAGGTTAATCGGCCA
>FQH3XDB01CE9MU|640|11
GATGAACGCTGGCGGCATGCCTAAGACATGCAAGTCGAACGAAGGGACCCAATGAGATTTATAGAAGTTTGGAGAGCTTGCTCAAAGAATGGAAATAAATTGATTTGGATTATCCCTTAGTGGCAAACGGGTGAGTAACACGTGGGTTACCTGCCTTCAAGATGGGGATAACAGTTGGAAACGATTGCTAATACCGAATGTGATCTACGGATTAAAGAAGCCTTTAAAGCTTCGCTTGAAGATGGGCCTGCGGTGCATTAGCTAGTTGGTGAGATAACGGCCCACCAAGGCGACGATGCATAGCCGAACTGAGAGGTTAATCGGGCCACATTGGGACTGAGACACGGCCCCAAAACTCCGTAACGGGAGACAGCAGTTAGGAATAT
>FQH3XDB01D78D5|641|1
GATGAACGCTGGCGGCATGCCTAAGACATGCAAGTCGAACGAAGGGACCCAATGAGATTTATAGAAGTTTGGAGAGCTTGCTCAAAGAATGGAAATAAATTGATTTGGATTATCCCCTTAGTGGCAAAACGGGTGAGTAACACGTGGGTTACCTGCCTTCAAGATGGGGATAACAGTTGGAAACGATTGCTAATACCGAATGTGATCTACGGATTAAAGAAGCCTTTAAAGCTTCGCTTGAAGATGGGCCTGCGGTGCATTAGCTAGTTGTGAGGTAACGCCACCAAGGCGACGATGCATAGCCGAACTGAGAGGTTAATCGGCCACATTGGGACT
>FQH3XDB01CFCZD|642|3
GATGAACGCTGGCGGCATGCCTAAGACATGCAAGTCGAACGAAGGGACCCAATGAGATTTATAGAAGTTTGGAGAGCTTGCTCAAAGAATGGAAATAAATTAATTTGGATTATCCCTTAGTGGCAAACGGGTGAGTAACACGTGGGTTACCTGCCTTCAGGATGGGGATAACAGTTGGAAACGATTGCTAATACCGAATGTGATCTACGGATTAAAGAAGCCTTTAAAGCTTCGCTTGAAGATGGGCTTGCGGTGCATTAGCTAGTTGGTGAGATAACGGCCCACCAAGGCGACGATGCATAGCCGAACTGAGAGGTTAATCGGCCAC
>FQH3XDB01BQ3N4|643|2
GATGAACGCTGGCGGCATGCCTAAGACATGCAAGTCGAACGGAGGGACCCAATGATATTTTATGAAGTTCTGCGAGCTTGCTCAAAGAACAGATTAAAATGGATTTGGATTATCCCTTAGTGGCAAAACGGGTGAGTAACACGTGGGTTACCTACCTCCAAGTCGGGGACAACAGTTGGAAACGACTGCTAATACCGGATGTGGACTACGGTTTAAAGAAGCCTTTAAAAGCTTCGCTTGGAGATGGGCCTGCGGTGCATTAGCTAGTTGGTGGGTAATGGCCTACCAAGGCGACGATGCATAGCCGAACTGAGAGGTTAATCGGCCACATTGGGACTGAGACACGGCCCAAAACTCCTACGGGAGACAGCAGTTAGGAATATT
>FQH3XDB01BXPEO|644|5
GATTAACGCTGGCGGCATGCCTAAGACATGCAAGTCGAACGGGAAGCCCCAATGAAAATGGAATGAAGTTGAAGAGCTTGCTCTGATATGGAATGAAGTTGGATTTGGATTCTGCTTCCAGTGGCGAACGGGTGAGTAACACGTGGGTTATCTGCCTTCAAGCTGGGGATAACGGTTAGAAATGATCGCTAATACCGAATGTGCTAGTAATAGTAAAGGCGCTCTTAAGCGTCACTTGAAGATGAGCCTGCGGCGTATTAGCTAGTTGGTGGGTAATGGCCTACCAAGGCGACGATGCGTAACCGGACTGAGAGGTTG
>FQH3XDB01CJS5W|645|2
GATGAACGCTGGCGGCATGCCTAAGACATGCAAGTCGAACGGAGCGGCCCAATGAAGTGTGATGAAGCTAAGAGAGCTTGCTCAATTAGTGGATTCACTCAGATTTGGATTCCCGCTTAGTGGCGAAAGGGTGAGTAACACGTAGGAATCTACCTTAGAGACTGGGACAACAGTTGGAAACGACTGCTAATACCGGATGATATATAAAATGATACGTTATTTATAGTAAAAGGAGCCTTTAAAGCTTCACTTTAAGATGAGCCTGCGGCGTATTAGCTAGTTGGTAGGTAATGGCCTACCAAGGCAACGATGCGTAGCCGAACTGAGAGGTTGATCGGCCACATTGGGACTGAGACACGGCCCAAACTCCTAGTGGGAGACAGCAGTTAGGAATATCGTCAAGTAGGAGGAAACTCTAGAACGAG
>FQH3XDB01DHX82|646|1
GATTAACGCTGGCGGCATCGCCTAAGACATGGCAAAGGTCGAACGGGGTATACCACTGATCTCTGAATGAAGTTGAAGAGCTTGCTCAGATATGGAGTGACGAAGGACGTGGATCCTATACCTAGTGGCGAACGGGTGAGTAACACGTGGGTTACCTGCCTCTAAGTTGGGGATAACGGTTAGAAATGATCGCTAATACCGAATGTGCTAGTAATAGTAAAGGCGCTTTCAAGCGTCGCTTAGAGATGGGCCTGCGGCGTATTAGCTAGTTGGTGAGGTAACGGCTCACCAAGGCGACGATGCGTAGCCGGACTGAGAGGTTAAAC
>FQH3XDB01C1PML|647|9
AATGAACGTTGGCGGCGTGGATTAGGCATGCAAGTCGTGCGGCAAGGAAGACTTCGGTCTTCCCTAGAGCGGCGAAAGGGATAGGAACACATAGATACATGCCTCTTAGCCCGGGATAGCGTTTGGAAACGAACGATAATACCGGACGCCCTCTCCGGAGGAAAGGTGAGATTCCGCTAAGAGATTGGTCTATGTCCTATTAGCTAGTTGGTAAGTAACGGCTTACCAAGGCGATGATGGGTACGGGGTGTGAGAGCATGACCCCGATCACTGGGACTGAGACACTGCCCAGACGCCTACGGGCGGCTGCAGTCGAGAATCTTCGGCAATGGACGAAAGTCTGACCGAGCGACGCCGCGTGTATGACGAAGGTCTTCGGATTGTAAAGTACTGTCGATTAGGC
>FQH3XDB01AMCKW|648|2
GATGAACGCTGGCGGCATGCCTAAGACATGCAAGTCGAACGGAGCGGCCCAATGAAATTTATTGATATTATGAAAGCTTGCTGATTAATTGATATAAGTGGATTTGGATTACCGCTTAGTGGCGAAAGGGTGAGTAACACGTAGGAATCTGCCTCAGAGACTGGGACAACAGTTGGAAACGACTGCTAATACCGGATGATATGTTAAATGATACGTTATTTAATATTAAAAGGAGCCTTTAAAAGCTTCACTTTGAGATGAGCCTGCGGCGTATTAGCTAGTTGGTAAGGTAATGGCTTACCAAGGCAACGATGCGTAGCCGAACTGAGAGGTTGATCGGCCACATTGGGGACTGAGACACGGCCCAAACTCCTATGGGAGACAGCAGTTAGG
>FQH3XDB01EQOV7|649|1
AATGAACGCTAGCAATAAGCCTAACACATGCAAGTTAAACAATATTTTTGCTAATTAAATATTGTAGCAAACGGGTGAGTAATACATAGGAAGCTGCCTTACAATTTAGTTAAACACTAAATAAATAGTTTTTATTTAAAGCTATAAAAGCAAAGTACATCCAAAATGTTTAAAAATTTAGGTTTCTGTTGTAAGATGCGCCTATGCAGGATTAGGTAGTTGGTGAGATAACGGCTCGCCAAGCCTTTGATCTTTAGCTGATCTGAGAGGATAACCAGCCACATTGGGACTGAGACACGGCCCAAACGTTTTCCAACGGCAGCAGTGAGGAATTTTGGACAATGGGCGAAAGCCTGATCCAGCTATGTTGCATGGGTGATGAAGGCTG
>FQH3XDB01BLTLE|650|7
GATGAACGCTAGCGGCAGGCTTAACACATGCAAGTCGAGGGGCAGCGCGTGGTGGCAACACTATGGCGGCGACCGGCGGAAGGGTGCGTAACGCGTGAGCGACATGCCCCGTTGCCCGGGGACAACCGGTGGAAACGCCGACTAATCCCCGATGGGACACGGAACCGCATGGTTCTGTGTTGAAAGTTTTGGCGGCGACGGATTGGCTCGCGTCCGATTAGCTAGTTGGCGGGTAACGGCCCACCAAGGCAACGATCGGTAGGGGTTCTGAGAGGAAGGTCCCCCC
>FQH3XDB01EMB6P|651|14
GATGAACGCTAGCTACAGGCTTAACACATGCAAGTCGAGGGGCAGCATTAGGTAGCGATACTGAGATGGCGACCGGCGCACGGGTGCGTAACGCGTATCCAACTTTCCCTATGCTCATGGATAGCCTTCCGAAAGGGAGATTAATACATGATGGTGTCTGAAATTCGCATGTAGATCGGACTAAAGATTTATCGGCATAGGATAGGGATGCGTTCCATTAGATAGTTGGCGGGTAACGCCCACCAAGTCTGCGATGGATAGGGGTTCTGAGAGG
>FQH3XDB01EFOW4|652|3
AATGAACGCTGGCGGCATGGATTAGGCATGCAAGTCGAACGGGGCGCAGCGATGCGCCCAGTGGCGAAAGGGTGAGTAGCACGTGAAGAACCTGCCCCCGGATCCGGGACAAGCGCTGGAAACGGCGTCTGATACGGGATGTGGCCGGGCCGCCGCATGGCGGCCGGGCTAAAGATTTCATCGTCCGGGGAGGGCTTCGCGCCCCATTAGCTGGTTGGCGGGGTGACGGCCCACCAAGGCTGCGACGGGTAGGTGGTCTGAGAGGATGGTCACCCACACTGGGACTGAGACACTGCCCAGACTCCTACGGGAGGCTGCAGTCGA
>FQH3XDB01DWZZP|653|82
GATGAACGCTGGCGGCGTGCCTAACACATTCAAGTCGAACGGAGCTGTAATACTTAATCCTTCGGGAAGCGGTAGAGCAGCTTAGTGGCGGACGGGTGAGTAACGCGTGAGCAACCTGCCCTTATGAACGGAACAACACAGGGAAACTTGTGCTAATACCGTATGACGTATAACCCGGGCATCCGGGAGATACCAAAGATTTATCGCATAAGGATGGACTCGCGTCCGATTAGATAGTTGGCGGGGTAAAGGCCACCAAGTCGACGATCGGTAGCCGGACTGAGAGGTTGAACGGCCACATTGGAACTGAGATACGGCCCAGACTCCTACGGGAGGCAGCAGTGGGGAATATTGGGGCAATGGGCGCAAGCCTGACCCAGCGACGCCGCGTGAGTGAAGAAGGTCTTCGGATT
>FQH3XDB01CISNN|654|3
GATGAACGCTGGCGGCGTGCTTAACACATGCAAGTCGAACGAAGCACTCTACTTGATTTCCTTCGGGATTGATTGTTCTGTGACTGAGTGGCGGACGGGTGAGTAACGCGTGGATAACCTGCCTCACACAGGGGGATAACAGTTGGAAACGGCTGCTAATACCGCATAAGCGCACAGTGCTGCATGGCACAGTGTGAAAAACTCCGGTGGTGTGAGATGGATCCGCGTCTGATTAGCTTGTTGGCGGGTAACGCCACCAAGGCGACGATCAGTAGCCGGCCTGAGAGGGCGACCGGCACATTGGGACTGAGACAC
>FQH3XDB01BS6GQ|655|54
GATGAACGCTGGCGGCGTGCTTAACACATGCAAGTCGAACGGAGATGTTTCGCTGAAGCGAGCTTGCTCAAATCATGAGACATCTTAGTGGCGGACGGGTGAGTAACGCGTGGGTAACCTGCCATATACAGGGGGATAACACTTAGAAATAGGTGCTAATACCGCATAAGCGCACGGTGTCGCATGACACAGTGTAGAAAAACTCCGGTGGTATATGATGGACCCGCGTCTGATTAGCTGGTTGGTGAGGTAACGGCTCACCAAGGCGACGATCAGTAGCCGGCCTGAGAGGGTGAACGGCCACAGTTGGGACTG
>FQH3XDB01C3ERB|656|36
GATGAACGCTGGCGGCGTGCTTAACACATGCAAGTCGAACGGACATCTGCGAAACCTAGTGAACGGATGTTAGTGGCGGACGGGTGAGTAACGCGTGGGCAACCTGCCCTGTACCGGGGGATAACACTTAGAAATAGGTGCTAATACCGCATAAGCGCACAGTTTCGCATGGAACGGTGTGAAAAACTCCGGTGGTACAGGATGGACCCGCGTCTGATTAGCTAGTTGGTGGGGTAAGGCCTACCAAGGCGACGATCAGTAGCCGGCCTGAGAGGGTGACCGGCCACATTGGGACTGAGACACGGCCC
>FQH3XDB01D4YUX|657|3
GAATGAACGCTGGCGGCGTGGCTTAAACACATGCAAGTCGAACGAAGCACTTTAATTTGATTTTCTTCGGAAATGAAGATTCTGTGACTGAGTGGCGGACGGGTGAGTAACGCGTGGGTAACCTGCCTCATACAGGGGGATAACAGTTGGAAACGACTGCTAATACCGCATAAGCGCACAGTACTGCATGGTACAGTGTGAAAAACTCCGGTGGTATGGGATGGACCCGCGTCTGATTAGCTGGTTGGCGGGGTAACGGCCCACCAAGGCGACGATCAGTAGCCGACCTGAGAGGGTGACCGGCCACATTGGGACTGAGACACGGCCCAAAACTTCCACGGGAGGCAGCAGT
>FQH3XDB01ESBJV|658|1
GAATGAACGCTGGCGGCGTGGCTTAAACACATGCAAGTCGAACGAAGCACTTTAATTTGATTTTCTTCGGAAATGAAGATTTTGTGACTGAGTGGCGGACGGGTGAGTAACGCGTGGGGTAACCTGCCTCATACAGGGGGATAACAGTTGGAAACGGCTGCTAATACCGCATAAGCGCACAGTGCTGCATGGTACAGTGTGAAAAACTCCGGTGGTATGGGGATGGACCCGCGTCTGATTAGCTGGTTGGCGGGGTAACGGCCCACCAAGGCGACGATCAGTAGCCGACCTGAGAGGGTGACCGGGCCACATTGGGACTGAGACACGGGCCCCAAAACTCCTACGGGAGGCAGCAGTGGGGAATATTGCACAATGGGGGAAACCCTGATGCAGCGACGCCGCGTGAGCGA
>FQH3XDB01DHLLJ|659|5
GAATGAACGCTGGCGGCGGTGCTTAACACATGCAAGTCGAACGAAGACATTCGAGTGCTTGCACTTGGATGGACTGAGTGGCGGACGGGTGAGTAACGCGTGGGTAACCTGCCTTATACAGGGGGATAACAGTTGGAAACGACTGCTAATACCGCATAAGCGCACAGCATCGCATGATGCAGTGTGAAAAAACTCCGGTGGTATAAGATGGACCCGCGTCTGATTAGCTGGTTGGTGAGGTAACGGCTCACCAAGGCGACGATCAGTAGCCGGCCTGAGAGGGTGAACGGCCACATTGGGACTGAGACACGGCCCAAAACTCCTACGGGAGCAGCAGTGGGGAATATT
>FQH3XDB01BLJVR|660|6
GATGAACGCTGGCGGCGTGCTTAACACATGCAAGTCGAACGAAGACATTCGAGTGCTTGCACTTGGATGGACTGAGTGGCGGACGGGTGAGTAACGCGTGGGTAACCTGCCTTATACAGGGGGATAACAGTTGGAAACGACTGCTAATACCGCATAAGCGCACAGCATCGCATGATGCAGTGTGAAAAAACTCCGGTGGTATAAGATGGACCCGCGTCTGATTAGCTGGTTGGTGAGGTAACGGCTCACCAAGGCGACGATCAGTAGCCGGCCTGAGAGGGTGAACGGCCACATTGGGACTGAGACACGG
>FQH3XDB01BYLF7|661|1
GAATGAACGCTGGCGGCGGTGACTTAACACATGCAAGTCGAACGAAGCGCTTTATTTGATTTTCTTCGGAAATGAAGATTTGTGACTGAGTGGCGGACGGGTGAGTAACGCGTGGGTAACCTGCCGTCATACAGGGGGATAACAGTTGGAAACGGCTGCTAATACCGCATAAGCGCACAGTACCGCATGGTACGGTGTGAAAAACTCCGGTGGTATGGGATGGACCCGCGTCTGATTAGCTGGTTGGCGGGGTAATGGCCCACCAAGGCGACGATCAGTAGCCGACCTGAGAGGGTGACCGGCCACATTGGGGACTGAGACAC
>FQH3XDB01COSY2|662|1
AATGAACGCTGGCGGTATGCTTAATACATGCAAGTCGAACGAGAAAGTCTAGCAATAGGCGAGTAAAGTGGCGTACGGGTGAGTAATATATAGGAATCTCCCCAGTAGTGAGGAATAAGCACTGGAAACGGTGTCTAATACCACATAATATCGTTTAGTTGAGTCTAATCGAGAAAGGATTAATCTTCGGGTTAATTTGCTATTGGATGGGCCTATATCGGATTAGCTAGTTGGTGGGGTAAAGGCCTACCAAGGCGATGATCCGTAGCTGATCTGAGAGGGTGATCAGCCACATTGGGGACTGCGACACGGCCCCAAAACTCCTACGGGAGGCAGCAGTAGGGAATATTGGACAAGTGGGGAAACCTGATCCAGCAATACCGCGTGAATGAT
>FQH3XDB01AQN4T|663|4
AATGAACGCTGGCGGTATGCTTAATACATGCAAGTCGAACGAGAAAGTCTAGCAATAGATGAGTAAAGTGGCGTACGGGTGAGTAATATATAGGAATCTACCCAGTAATGAGGAATAAGCACTGGAAACGGTGTCTAATACCACATACTACCATTTATTGGGGAAAGGGTTAATAGCAATATTAACTTGTTATTGGATGAGCCTATATCGGATTAGCTAGTTGGTGGGGTAAAGGCCTACCAAGGCAATGATCCGTAGCTGGTCTGAGAGGGTGATCAGCCACATTGGGACTGCGACACGGCCCAAACTCCGTACGGGAGGCAGCAGTAGGAATATTGGACAAGTGGGGAC
>FQH3XDB01CJ51S|664|1
AATGAACGCTGGCGGTATGCTTAATACATGCAAGTCGAACGAGAAAGTCTAGCAATAGATGAGTAAAGTGGCGTACGGGTGAGTAATATATAGGAATCTACCCAGTAATGAGGAATAAGCACTGGAAACGGTGTCTAATACCACATACTACCATTTATTTGGGGAAAGGGTTAATAGCAATATTAACTGTATGGATGAGCCTATATCGGATTAGCTAGTTGGTGGGGTAAAGCCTACAAGGCAATGATCCGTAGCT
>FQH3XDB01EHMB8|665|1
AATGAACGCTGGCGGTATGCTTAATACATGCAAGTCGAACGAGAAAGTTTAGCAATAAACGAGTAAAGTGGCGTACGGGTGAGTAATGTATAGGAATCTACCTAATAATATGGAATAAGCACTGGAAACGGTGTCTAATACCGTATACTACCATTTATTTGGGAAAGGGTTAATAGCAATATTAACTTGTTATTAGATGAGCCTATATCGGATTAGCTTGTTGGTGGGGTAAAGGCCTACCAAGGCGATGATCCGTAGCTGGTCTGAGAGGGTGATCAGCCACATTGGGGACTGCGACACGGCCC
>FQH3XDB01DLTAS|666|1
AATGAACGCTGGCGGTATGCTTAATACATGCAAGTCGAACGAGAAAGTCTAGCAATAGATGAGTAAAGTGGCGTACGGGGTGAGTAATATATAGGAATCTACCCAGTAATGAGGAATAAGCACTGGAAACGGTGTCTAATACCACATACTACCATTTATTTGGGAAAGGGTTAATAGCAATATTAACTTGTTATTGGATGAGCCTATATCGGATTAGCTAGTTGGTGGGGTAAAGGCCTACCAAGGCAATGATCCGTAGCTGGTCTGAGAGGGTGATCAGCCACATTGGGGACTGCGACACGGCCCAAACTCCGTACGGGGAGGCAGCAGTAGGGAATATGGACAATGGGCGAAAGCCTAATCCAGC
>FQH3XDB01ASTH2|667|1
ATACCGCGGCTGCTGGCACGAAGTTAGCCGGTGCTTTTTCTGTGGGTACCGTCATTATCTTCCCCACTAAAAGAACTTTACAATCCGAAGACCTTCATCATTCACGCGGTATTGCTGGATCAGGGTTTCCCCCATTGTCCAATATTCCCTACTGCTGCCTCCCGTAGGAGTTTGGGCCGTGTCGCAGTCCCAATGTGGCTGATCACCCTCTCAGATCAGCTACGGATCATCGCCTTGGTAGGCCTTTACCCCACCAACTAGCTAATCCGATATAGGCTCAGTCCAATAGCAAATTAACCCCGAAGATTTAATCCTTTCTCGATTAGACTCAACTAAACGATATTATGTGGTATTAGACACCGTTTCCAGTGCTTATTCCTCACTACTGGGGAGATTCCTATATATTACTCACCCGTACGCCACTTTACTCGCCTATTGCTAGACTTTCTCGTTCGACTTGCATGTATTAAGCATACCGCCAGCGTTCATT
>FQH3XDB01CXPBP|668|21
GATGAACGCTGGCGGCGTGCTTAACACATGCAAGTCGAACGAAGACATTCGAGTGCTTGCACTTGGATGGACTGAGTGGCGGACGGGTGAGTAACGCGTGGGTAACCTGCCCTTATACAGGGGGATAACAGTTGGAAACGACTGCTAATACCGCATAAGCGCACGGTATCGCATGATACAGTGTGAAAAACTCCGGTGGTATAAGATGGACCCGCGTCTGATTAGCTGGTTGGTGAGGTAACGGCTCACCAAGGCGACGATCAGTAGCCGGCCTGAGAGGGTGAACGGCCACATTGGGACTGAGACACGGCCCAAAACTCCTACGGGAGCAGCAGTGGGAATATGCACAATGGGGGAAACCCTGATGCAGCGACGCCGCGTGAGTGATGAAGTATTT
>FQH3XDB01BIRWC|669|1
GATGAACGCTGGCGGCATGCCTAAGACATGCAAGTCGTACGCGAAGGCCTAATGATTACTGTGGAAGATTGGAGAGCTTGCTCAAAGATTGGAAACAGTAAGATTTAGATTACCTTCGAGTGGCAAACGGGTGAGTAACACGTGGGTTACCTGCCTCTAGGTTGGGGATAACGGTTGGAAACGATCGATAATACCGAATGTGCTCTACGGAGTAAAGAAGTCCTTTAAAGCTTCGCCAAGAGATGGGCCTGCGGCGTATTAGCTAGGTTGGTGGGTAAGCCTACAAGGCGACGATACGTAGCCGAACTGA
>FQH3XDB01BX50A|670|4
GACGAACGCTGGCGGCGTGCCTAACACATGCAAGTCGAGCGGTTTTTAGAGGCAAGAATCGAGTGGCAAAAGAGGATTGGATTCTGTTATTTGCAACTGGGTTCTTGTTTCTGGAGATAGCGGCGGACGGGTGAGTAACGCGTGAGCAACCTGCCTTCTTGAGGGGGATAACGTCTGGAAACGGACGCTAATACCGCATAAAATACTTAAGTCGCATGGTTTAAGTATCAAAGGAGCAATCCGCAGGAAGATGGGCTCGCGTCCGATTAGCTAGTTGGAGGGTAAAGCCACAAGGCAACGATCGGTAGCCGGACTGAGAGGTTGAACGGCCACGATTGGGACTGAGACACGG
>FQH3XDB01A82HG|671|2
GACGAACGCTGGCGGCGTGCCTAACACATGCAAGTCGAGCGGTTTTTAGAGGCAAGAATCGAGTGGCAAAAGAGGAGTGGACTCTGTTATTTGCAACTGGGTTCTTGTTTCTGGAGATAGCGGCGGACGGGTGAGTAACGCGTGAGCAACCTGCCTTCTTGAGGGGGATAACGTCTGGAAACGGACGCTAATACCGCATAAAATACTTAAGTCGCATGGTTTAAGTATCAAAAGGAGCAATCCGCAGGAAGATGGGCTCGCGTCCGATTAGCTAGTTGGAGGGGTAAAGGCCCACCAAGGCAACGATCGGTAGCCGGACTGAGAGGTTGAACGGCCACATTGGGACTGAGACACGGCCCAGACTCCTAC
>FQH3XDB01EQUCN|672|113
GACTAACGCTGGCGGTGCGCCTAACACATGCAAGTCGTACGAGCCCTTCGGGGCGAGTGGCAGACGGGTGAGTAACGCGTAGGTAATGTGCCTCTTCGCTGGGGGTACCTATTCGAAAGGATAGCTAATACCGAATGAACTTGTTGAACGGCATCGTTTGACTCGTAAACCTCCGGGGCGAAGAGATCAGCTTGCGTACTATCAGCTAGTAGGTGGGGTAACGGCCTACCTAGGCGACGACGGTTAGCCGGTCTGAGAGGATGATCGGCCACATTGGAACTGAAACACGGTCCAGACTCCTACGGGAGGCAGCAGTGAGGAATCTTGCGCAATGGGCGAAAGCCTGACGCAGCGACACCGCGTGAGGGATGAAGGTCTTCGGATTGTAAACCTTTGTCAGTGGGGACGAGAACGGACGGTACCTACTGAGGAAGCATCGGCTTACGTACGTGCCAGCAGCCG
>FQH3XDB01E1UDF|673|49
GACTAACGCTGGCGGTGCGCCTAACACATGCAAGTCGTACGAGCCCTTCGGGGCGAGTGGCAGACGGGTGAGTAACGCGTAGGTAATGTGCCTCTTCGCTGGGGATACCTATTCGAAAGGATAGCTAATACCGAATGAACTTGTTGAACGGCATCGTTTGACTCGTAAACCTCCGGGGCGAAGAGATCAGCTTGCGTACTATCAGCTAGTAGGTGGGTAACGCCTACCTAGGCAACGACGGTTAGCCGGTCTGAGAGGATGATCGGCCACATTGGAACTGAAAC
>FQH3XDB01DAY0W|674|3
GACTAACGCTGGCGGTGCGCCTAACACATGCAAGTCGTACGAGCCCCTTCGGGGCGAGTGGCAGACGGGTGAGTAACGCGTAGGTAATGTGCCTCTTCGCTGGGGATACCTATTCGAAAGGATAGCTAATACCGAATGAACTTGTTGAACGGCATCGTTTGACTCGTAAACCTCCGGGGCGAAGAGATCAGCTTGCGTACTATCAGCTAGTAGGTGGGTAACGCCTACCTAGGCGACGACGGTTAGCCGGTCTGAGAGGATGATCGGCCACATTGGAACTGAAACACGGTCCAGACTCCTACGGGAGCAGCAGTGAGGAATCTTGCGCAAGTGGGACGAAAGCCTGACGCAGCGACACCGCGT
>FQH3XDB01BOALX|675|1
GACTAACGCTGGCGGTGCGCCTAACACATGCAAGTCGTACGAGCCCCTTCGGGGCGAGTGGCAGACGGGTGAGTAACGCGTAGGTAATGTGCCTCTTCGCTGGGGATACCTATTCGAAAGGATAGCTAATACCGAATGAACTTGTTGAACGGCATCGTTTGACTCGTAAAACCTCCGGGGCGAAAGAGATTCAGCTTGCGTACTATCAGCTAGTAGGTGGGTAACGCCTACCTAGGCGACGACGGTTAGCCGGTCTGAGAGGATGATCGGCCACATTGGAACTGAAACACGGTCCAGACTCCTACGGGAGGCAGCAGTGAGGAATCTTGCGCAATGGGCAAAGCCTGACGCAGCGA
>FQH3XDB01CW990|676|2
GACTAACGCTGGCGGTGCGCCTAACACATGCAAGTCGTACGAGCCCTTCGGGGCGAGTGGCAGACGGGTGAGTAACGCGTAGGTAATGTGCCTCTTCGCTGGGGATACCTATTCGAAAGGATAGCTAATACCGAATGAACTTGTTGAACGGCATCGTTTGACTCGTAAACCTCCGGGGCGAAGAGATCAGCTTGCGTACTATCAGCTAGTAGGTGGGGTAATGGCCTACCTAGGCGACGACGGTTAGCCGGTCTGAGAGGATGATCGGCCACATTGGAACTGAAACACGGTCCAGACTCCTACGGAGGCAGCAGTGAGGAATCTTGCGCAATGGG
>FQH3XDB01A86C6|677|2
GACTAACGCTGGCGGTGCGCCTAACACATGCAAGTCGTACGAGCCCTTCGGGGTGAGTGGCAGACGGGTGAGTAACGCGTAGGTAATGTGCCTCTTCGCTGGGGATACCTATTCGAAAGGATAGCTAATACCGAATGAACTTGTTGAACGGCATCGTTTGACTCGTAAACCTCCGGGGGCGAAGAGATCAGCTTGCGTACTATCAGCTAGTAGGTGGGTAACGCCTACCTAGGCAACGACGGTTAGCCGGTCTGAGAGGATGATCGGCCACATTGGAACTGAAACACGGTCCAGACTCCGTACGGGAGGCAGCAGTGAGG
>FQH3XDB01BWGDU|678|3
GACTAACGCTGGCGGTGCGCCTAACACATGCAAGTCGTACGAGCCCTTCGGGGTGAGTGGCAGACGGGTGAGTAACGCGTAGGTAATGTGCCTCTTCGCTGGGGATACCTATTCGAAAGGATAGCTAATACCGAATGAACTTGTTGAACGGCATCGTTTGACTCGTAAACCTCCGGGGCGAAGAGATCAGCTTGCGTACTATCAGCTAGTAGGTGGGTAACGGCCTACCTAGGCAACGACGGTTAGCCGGTCT
>FQH3XDB01C1HPJ|679|2
GACTAACGCTGGCGGTGCGCCTAACACATGCAAGTCGTACGAGCCCTTCGGGGCGAGTGGCAGACGGGTGAGTAACGCGTAGGTAATGTGCCTCTTCGCTGGGGATACCTATTCGAAAGGATAGCTAATACCGAATGAACTTGTTGAACGGCATCGTTTAACTCGTAAAACCTCCGGGGCGAAGAGATCAGCTTGCGTACTATCAGCTAGTAGGTAGGTAACGCCTACCTAGGCGACGACGGTTAGCCGGTCTGAGAGGATGATCGGCCACATTGGAACTGAAACACGGTCCAGACTCCTACGGAGGCAGCAGTGAGGAATTTTCGGC
>FQH3XDB01AWTLX|680|6
GACTAACGCTGGCGGTGCGCCTAACACATGCAAGTCGTACGAGCCCTTCGGGGCGAGTGGCAGACGGGTGAGTAACGCGTAGGTAATGTGCCTCTTCGCTGGGGATACCTATTCGAAAGGATAGCTAATACCGAATGAACTTGTTGAACGGCATCGTTTAACTCGTAAACCTCCGGGGGCAAGAGATCAGCTTGCGTACTATCAGCTAGTAGGTAGGTAACGGCCTACCTAGGCGACGACGGTTAGCCGGTCTGAGAGGA
>FQH3XDB01ASFP1|681|8
GACTAACGCTGGCGGTGCGCCTAACACATGCAAGTCGTACGAGCCCTTCGGGGCGAGTGGCAGACGGGTGAGTAACGCGTAGGTAATGTGCCTCTTCGCTGGGGATACCTATTCGAAAGGATAGCTAATACCGAATGAACTTGTTGAACGGCATCGTTTGACTCGTAAACCTCCGGGGCGAAGAGATCAGCTTGCGTACTATCAGCTAGTAGGTGGGGTAACGGCCTACCTAGGCGACGACGGTTAG
>FQH3XDB01B6H2J|682|1
GACTAACGCTGGCGGTGCGCCTAACACATGCAAGTCGTACGAGCCCTTCGGGGCGAGTGGCAGACGGGTGAGTAACGCGTAGGTAATGTGCCTCTTCGCTGGGGATACCTATTCGAAAGGATAGCTAATACCGAATGAACTTGTTGAACGGCATCGTTTGACTCGTAAAACCTCCGGGGCGAAGAGATCAGCTTGCGTACTATCAGCTAGTAGGTGGGGTAACGCCTACCTAGGCGACGACGGTTAGCCGGTCTGAGAGGATGATCGGCCACATTGGAACTGAAAACACGGTCCAGACTCCTACGGAGGCAGCAGTGAGGAATCTTGCGCAA
>FQH3XDB01CDKZ0|683|4
GACTAACGCTGGCGGTGCGCCTAACACATGCAAGTCGTACGAGCCCTCGGGGCGAGTGGCAGACGGGTGAGTAACGCGTAGGTAATGTGCCTCTTCGCTGGGGATACCTATTCGAAAGGATAGCTAATACCGAATGAACTTGTTGAACGGCATCGTTTGACTCGTAAACCTCCGGGGCGAAGAGATCAGCTTGCGTACTATCAGCTAGTAGGTGGGGTAACGCCTACCTAGGCAACGACGGTTAGCCGGTCTGAGAGGATGATCGGCCACATTGGAACTGAAACACGGTCCAGACTCCTACGGGAGGCAGCAGTGAGGAATCTTGCGCAAGTGGGCGAAAGCCTGACGCAGCGACACCGCGTGAGGGATG
>FQH3XDB01D3GPE|684|20
GACTAACGCTGGCGGTGCGCCTAACACATGCAAGTCGTACGAGCCCTTCGGGGTGAGTGGCAGACGGGTGAGTAACGCGTAGGTAATGTGCCTCTTCGCTGGGGATACCTATTCGAAAGGATAGCTAATACCGAATGAACTTGTTGAACGGCATCGTTTGACTCGTAAACCTCCGGGGCGAAGAGATCAGCTTGCGTACTATCAGCTAGTAGGTGGGTAACGGCCTACCTAGGCGACGACGGTTAGCCGGTCTGAGAGGATGATCGGCCACATTGGAACTGAAACACGGTCCAGACTCCTACGGGAGGCAGCAGTGAGGAATCTTGCGCAAGTGGGCGAAAAGCCTGACGCAGCGACACCGCGTGAGGGATGGAACGGTTCTTCGGATTGTAAACCTTGTCAGGTGGGAC
>FQH3XDB01ATT5Y|685|2
GACTAACGCTGGCGGTGCGCCTAACACATGCAAGTCGTACGAGCCCTTCGGGGCGAGTGGCAGACGGGTGAGTAACGCGTAGGTAATGTGCCTCTTCGCTGGGGATACCTATTCGAAAGGATAGCTAATACCGAATGAGCTTGTTGAACGGCATCGTTTGACTCGTAAACCTCCGGGGCGAAGAGATCAGCTTGCGTACTATCAGCTAGTAGGTGGGGTAACGGCCTACCTAGGCGACGACGGTTAGCCGGTCTGAGAGGATGATCGGCCACATTGGAACTGAAACACGGTCCAGACTCCTACGGGAGGCAGCAGTGAGGAATCTTGCGCAATGGGCGAAAGCCTGACGCAGCGACACCGCGTTGAGGGATGAAGGTCTTCGGATTGTAAA
>FQH3XDB01DUIR5|686|16
GACTAACGCTGGCGGTGCGCCTAACACATGCAAGTCGTACGAGCCCTTCGGGGCGAGTGGCAGACGGGTGAGTAACGCGTAGGTAATGTGCCTCTTCGCTGGGGATACCTATTCGAAAGGATAGCTAATACCGAATGAACTTGTTGAACGGCATCGTTTAACTCGTAAACCTCCGGGGCGAAGAGATCAGCTTGCGTACTATCAGCTAGTAGGTAGGTAACGCCTACCTAGGCGACGACGGTTAGCCGGTCTGAGAGGATGATCGGCCACATTGGAACTGAAACACGGTCCAGACTCCTACGGGAGGCAGCAGTGAGGAATCTTGCGCAATGGGCGAAAGCCTGACGCAGCGACACCGCGTGAGGGAT
>FQH3XDB01D7XSD|687|12
GACTAACGCTGGCGGTGCGCCTAACACATGCAAGTCGTACGAGCCCTTCGGGGCGAGTGGCAGACGGGTGAGTAACGCGTAGGTAATGTGCCTCTTCGCTGGGGATACCTATTCGAAAGGATAGCTAATACCGAATGAACTTGTTGAACGGCATCGTTTGACTCGTAAAACCTCCGGGGCGAAGAGATCAGCTTGCGTACTATCAGCTAGTAGGTGGGGTAACGCCTACCTAGGCGACGACGGTTAGCCGGTCTGAGAGGATGATCGGCCACATTGGAACTGAAACACGGTCCAGACTCCTACGGGAGGCAGCAGTGAGGAATCTTGCGCAATGGGCGAAAGCCTGACGCAGCGACACCGCGTGAGGGATGAAGGTCTTCGGATTGTAAACCTTTGTCAGTGGGGACAGAACGGACGGTACCTATGAGGAAGCATCGGCTT
>FQH3XDB01EUJRP|688|9
GACTAACGCTGGCGGTGCGCCTAACACATGCAAGTCGTACGAGCCCTTCGGGGCGAGTGGCAGACGGGTGAGTAACGCGTAGGTAATGTGCCTCTTCGCTGGGGATACCTATTCGAAAGGATAGCTAATACCGAATGAACTTGTTGAACGGCATCGTTTAACTCGTAAACCTCCGGGGGCGAAGAGATCAGCTTGCGTACTATCAGCTAGTAGGTAGGGTAACGGCCTACCTAGGCGACGACGGTTAGCCGGTCTGAGAGGATGATCGGCCACATTGGAACTGAAACACGGTCCAGACTCCTACGGGAGGCAGCAGTGAGGAATCTTGCGCAAGTGGGCGAAAGCCTGACGCAGCGACACCGCG
>FQH3XDB01BB91M|689|2
GACTAACGCTGGCGGTGCGCCTAACACATGCAAGTCGTACGAGCCCTTCGGGGCGAGTGGCAGACGGGTGAGTAACGCGTAGGTAATGTGCCTCTTCGCTGGGGATACCTATTCGAAAGGATAGCTAATACCGAATGAACTTGTTGAACGGCATCGTTTGACTCGTAAACCTCCGGGGCGGAAGAGATCAGCTTGCGTACTATCAGCTAGTAGGTGGGGTAACGGCCTACCTAGGCGACGACGGTTAGCCGGTCTGAGAGGATGATCGGCCACATTGGAACTGAAACACGGTCCAGACTCCTACGGGAGGCAGCAGTGAGGAATCTTGCGCAATGGGGCGAAAGCCTGACGCAGCGACACCGCGTGA
>FQH3XDB01B850G|690|1
GACTAACGCTGGCGGTGCGCCTAACACATGCAAGTCGTACGAGCCCTTCGGGGCGAGTGGCAGACGGGTGAGTAACGCGTAGGTAATGTGCCTCTTCGCTGGGGATACCTATTCGAAAGGATAGCTAATACCGAATGAACTTGTTGAACGGCGTCGTTTGACTCGTAAAACCTCCGGGGCGAAGAGATCAGCTTGCGTACTATCAGCTAGTAGGTGGGGTAACGCCTACCTAGGCAACGACGGTTAGCCGGTCTGAGAGGATGATCGGCCACATTGGAACTGAAACACGGTCCAGACTCCTACGGAGGCAGCAGTGAGGAATCTTGCGCAATGGGCGAAAAGCCTGACGCAGCGACACCGCGTGAGGGATGAAGGTCTTCGGATTGTAAACCTTGTCAGTGGGACGAGAAGGGACGGTACCTACTGAGGAAGCATCGGCTTAC
>FQH3XDB01C9QAG|691|2
GACTAACGCTGGCGGTGCGCCTAACACATGCAAGTCGTACGAGCCCTTCGGGGCGAGTGGCAGACGGGTGAGTAACGCGTAGGTAATGTGCCTCTTCGCTGGGGATACCTATTCGAAAGGATAGCTAATACCGAATGAACTTGTTGAACGGCGTCGTTTGACTCGTAAACCTCCGGGGCGAAGAAATCAGCTTGCGTACTATCAGCTAGTAGGTGGGGTAACGGCCTACCTAGGCAACGACGGTTAGCCGGTCTGAGAGGATGATCGGCCACATTGGAACTGAAACACGGTCCAGACTCCTACGGGAGGCAGCAGTGAGGAATCTTGCGCAATGGGCGAAAGCCTGACGCAGCGACACCGCGTGAGGGATGAAGGTCTTCGGATTGTAAACCTTTGTCAGTGGGACGAGAACGGACGGTACCTACTGAGGAAGCATCGGCTTACTACGTGCCAGCAGCCGCGTAATTACGATACGTCG
>FQH3XDB01DI3P9|692|1
GACTAACGCTGGCGGTGCGCCTAACACATGCAAGTCGTACGAGCCCTTCGGGGCGAGTGGCAGACGGGTGAGTAACGCGTAGGTAATGTGCCTCTTCGCTGGGGATACCTATTCGAAAGGATAGCTAATACCGAATGAGCTTGTTGAACGGCATCGTTTGACTCGTAAACCTCGGGGCGAAGAGATCAGCTTGCGTACTATCAGCTAGTAGGTGGGTAACGGCCTACCTAGGCGACGACGGTTAGCCGGTCTGAGAGGATGATCGGCCACATTGGAACTGAAACACGGTCCAGACTCCTACGGGAGGCAGCAGTGAGGAATCTTGCGCAATGGGCGAAAGCCTGACGCAGCGACACCGCGTGAGGGATGAAGGTCTTCGGATTGTAAACC
>FQH3XDB01COMS1|693|1
GACTAACGCTGGCGGTGCGCCTAACACATGCAAGTCGTACGAGCCCTTCGGGGTGAGTGGCAGACGGGTGAGTAACGCGTAGGTAATGTGCCTCTTCGGCTGGGGGATACCTATTCGAAAGGATAGCTAATACCGAATGAACTTGTTGAACGGCATCGTTTGACTCGTAAAACCTCCGGGGCGAAGAGATCAGCTTGCGTACTATCAGCTAGTAGGTGGGGTAACGCCTACCTAGGCAACGACGGTTAGCCGGTCTGAGAGGATGATCGGCCACATTGGAACTGAAACACGGTCCAGACTCCTACGGGAGGCAGCAGTGAGGAATCTTGCGCAATGGGCAAAGCCTGACGCAGCGACACCGCGTGAGGGATGAAGGTCTTCGGATT
>FQH3XDB01C73FR|694|2
GACTAACGCTGGCGGTGCGCCTAACACATGCAAGTCGTACGAGCCCTTCGGGGCGAGTGGCAGACGGGTGAGTAACGCGTAAGCAACCTGCCCCGCACACCGGAACAACCGTGCCAACGCGCGGCTAATGCCCGGGAGCCGTGGTTCCCCGCATGGGGGATTGACGAAAGATTTATCGGTGCGGGATGGGGCTTGCGTCCGATTAGCTAGTTGGCGGGGCAACGGCCCACCAAGGCGACGATCGGTAGCCGGCCTGAGAGGGCGATCGGCCACATTGGGACTGAGAGACGGCCCAGACTCCTACGGGAGGCAGCAGTAGGGAATATTGCGCAATGGGGGCAACCCTGACGCAGCAACTCCACGTGTGGGATGAAGCATTTCGGTGTGTAAACC
>FQH3XDB01DMGDT|695|5
GATGAACGCTGGCGGCATGCCTAAGACATGCAAGTCGTACGAGGCGGCCCACAGACTCAAACGGAAGTGAAGCGAGCTTGCTCAATTCATGGAAGGATGAAGACGTGGAATCTCCGCCTAGTGGCAAACGGGTGAGTAACACGTGGGTTACCTACCTCTTAGATGGGGATACCAATTGGAAACGATTGTTAATACCGAATGTGATCTACGGATTAAAGGAGCCTTTAAAGCTCCGCTAAGAGATGGGCCTGCGGTGCATTAGATAGTTGGTGGGTAACGCTACAAGTCGACGATGCATAGCTGAACTGAGAGGTTGATCGGCCACACTGGGACTGAGACACGGGCCCAGACCTCC
>FQH3XDB01E0DFY|696|1
GATGAACGCTGGCGGCATGCCTAAGACATGCAAGTCGTACGAGGCGGCCCACTGACGCAGACGGAAGTGAAGCGAGCTTGCTCAATTCATGGAAGTCTGAAGACGTGGATTCTCCGCCTAGTGGCAAACGGGTGAGTAACACGTGGGTTACCTACCTCTTAGATGGGGATACCAATTGGAAACGATTGTTAATACCGAATGTGATCTACGGATTAAAGGAGCCTTTAAAGCTCCGCTAAGAGATGGGCCTGCGGTGCATTAGATAGTTGGTGGGTAACGGCCTACCAAGTCGACGATGCATAGCTGAACTGAGAGGTTGATCGGCCACACTGGGACTGAGACACGGCCCAGACTCCGTACGGGAGACAGCAGTTAGGAATATCGTCAAGTGGGGGAAACCCTGAACGAGCAATGCCGCGTGAACGATGACGGTCCTCTGGGATTGGTAAAGTTC
>FQH3XDB01D7L8N|697|2
GATGAACGCTGGCGGCATGCCTAAGACATGCAAGTCGAACGAGGCGGCCCACTGACGTAAATGGAAATTGGAGTGCTTGCACAAAGATGGAAATTTACAGACGTGGATTCTCCGCCTAGTGGCGCAAGGGTGAGTAACACGTGGGTAATCTACCTTAGAGACTGGGATAACAGTTGGAAACGACTGCTAATACCGGATGATATTTAAGAAGATACGTTTCTAAATTAAAGGAGCCTTTTAAAAGCTTCACTTTAAGATGAGCCTGCGGCGTATTAGCTAGTTGTGGGTAAAGCCTACAAGGCAACGATGCGTA
>FQH3XDB01C6T08|698|1
GATGAACGCTGGCGGCATGCCTAAGACATGCAAGTCGAACGAGGCGGCCCACTGACGTAAATGGAAATTGGAGTGCTTGCACAAAGATGGAAATTTACAGACGTGGATTCTCCGCCTAGTGGCGCAAGGGTGAGTAACACGTGGGTAATCTACCTTAGAGACTGGGGATAACAGTTGGAAACGCTGCTAATACCGGATGATATTAAGAAGATACGTTTCTAAATTAAAGGAGCCCTTTTAAAAGCTTCACTTTAAGATGAGCCTGCGGCGTATTAGCTAGTTGGTGGGTAAAGCCTACCAAGGCAACGATGCGTA
>FQH3XDB01BSS7T|699|7
GATGAACGCTGGCGGCATGCCTAAGACATGCAAGTCGTACGAAGCGGCCCACTGACTCATTTCGAAACATGGAGAGCTTGCTCAAAGTGTGGACTTATGATGATGTGGATTTTCCGCTTAGTGGCAAACGGGTGAGTAACACGTGGGTTACCTACCTCTTAGATGGGGATACCAATTGGAAACGATTGTTAATACCGAATGTGATCTACGGATTAAAGGAGCCTTTAAAGCTTCGCTAAGAGATGGGCCTGCGGTGCATTAGATAGTTGGTGGGTAATGGCCTACAAGTCAACGATGCATAGCTGAACTGAGAGGTTG
>FQH3XDB01B3NCB|700|1
GATGAACGCTGGCGGCATGCCTAAGACATGCAAGTCGTACGAAGCGGCCCACTGACTCATTTCGAAACATGGAGAGCTTGCTCAAAGTGTGGACTTATGATGATGTGGATTTTCCGCTTAGTGGCAAACGGGTGAGTAACACGTGGGTTACCTACCTCTTAGATGGGGATACCAATTGGAAACGATTGTTAATACCGAATGTGATCTACGGATTAAAGGAGCCTTTAAAGCTTCGCTAAGAGATGGGCCTGCGGTGCATTAGATAGTTGGTGGGGTAATGGCCTACCAAGTCAACGATGCATAGCTGAACTGAGAGGTTGATCGGCCAC
>FQH3XDB01BI4TP|701|1
GATTAACGCTGGCGGCATGCCTAAGACATGCAAGTCGAACGGAGCGCCCCACTGAAGTTGGATGAAGTTGAGAAGCTTGCTTCAAGATGGATGAAGACGGATGTGGATTCTGCGCTCAGTGGCGAACGGGTGAGTAACACGTGGGTAATCTGCCTTCAAGTTGGGGATAACGGTTAGAAATGATCGCTAATACCGAATGTGCTAGAAATAGTAAAGGCGCTCTAAAGCGTCGCTTGAAGATGAGCCTGCGGTGTATTAGCTAGTTGGTGGGGTAATGGCCTACCAAGGCAACGATGCATAGCCGGACTGAGAGGTTGAACGGCCACACTGGGACTGAGACACGGCCCAGACTCCTACGGGAGACAGCAGTTAGGAATATTCGGCAATGGGGAAACCCTGACCGAGCAATGCCGCGTGTGCGATGAAGGTCCTTGGATTG
>FQH3XDB01D92H2|702|1
GATGAACGCTGGCGGCATGCCTAAGACATGCAAGTCGAACGGGATGGCCCCATTGATTACAACTGAAAACTGGAGTGCTTGCACAAAAGCTGGATGGTGTATGATTTGGATTTTCCATCCAGTGGCAAACGGGTGAGTAACACGTGGGTTACCTGCCTCTAAGTTGGGGATAACAGTTGGAAACGATTGCTAATACCGAATGTGCTCTACGGAGTAAAGATGCCTTTAAAGCATCGCTTAGAGATGGGCCTGCGGCGTATTAGTTAGTTGGCGGGGTAATGGCCTACAAGACGACGATGCGTAGCCGAACTGAG
>FQH3XDB01CTWDE|703|3
GATGAACGCTGGCGGCATGCCTAAGACATGCAAGTCGAACGGGATGGCCCCATTGATTACAACTGAAAGCTGGAGTGCTTGCACAAAAGTCGGATGGTGTATGATTTGGATTTTCCATCCAGTGGCAAACGGGTGAGTAACACGTGGGTTACCTACCTCTAAGTTGGGGATAACAGTTGGAAACGATTGCTAATACCGAATGTGCTCTACGGAGTAAAGATGCCTTTAAAGCATCGCTTAGAGATGGGCCTGCGGCGTATTAGTTAGTTGGTGGGGTAATGGCCTACCGAAGACGACGATGCGTAGCCGAACTGAGAGGTTAATCGGCCA
>FQH3XDB01EH3U3|704|1
GATTAACGCTGGCGGCATGCCTAAGACATGCAAGTCGAACGGAGTGCCCCACTGAAATATGAATGAAACTTGAGAGCTTGCTCAAAGGTGGAATGATTGTGGATGTGGATTCTGCACTCAGTGGCGAACGGGTGAGTAACACGTGGGTTACCTGCCTCTAAGTTGGGGACAACAGTTGGAAACGACTGCTAATACCGAATGTGATAGTAATATTAAAGGAGCCTTAAAGCTTCGCTTAGAGATGGGCCTGCGGCGTATTAGCTAGTTGGTGGGGTAATGGCCTACCAAGGCAACGATGCGTAGCCGGACTGAGAGGTTGATCGGCCACACTGGGACTGAGACACGGCCCAGACTCCTACGGGAGACAGCAGTTAGGAATATTCGG
>FQH3XDB01A0W9U|705|1
GATTAACGCTGGCGGCATGCCTAAGACATGCAAGTCGAACGGAACACCCCACTGAAATTTGAATGAAGTCTAAGAGCTTGCTCAAAGATGGAATGATAGTGGATGTGGATTCTGTGTTCAGTGGCGAACGGGTGAGTAACACGTGGGTTACCTGCCTCTAAGTTGGGGACAACAGTTGGAAACGACTGCTAATACCGAATGTGATAGAAATATTAAAGGAGCCTTAAAGCTTCGCTTAGAGATGGGCCTGCGGCGTATTAGCTAGTTGGTGGGGTAACGCCTACCAAGGCATCGATGCGTAGCCGGACTGAGAGGCTGATCGGACACACTGGGACTGAGACACGGCCCAGACTCCGTACGGGAGACAGCAGTTAGGAATATTCGTCAAGTGGGG
>FQH3XDB01AXO09|706|55
ATTGAACGCTGGCGGCATGCCTTACACATGCAAGTCGAACGGCAGCGCGGGAGCTTGCTCCTGGCGGCGAGTGGCGAACGGGTGAGTAATACATCGGAACGTGTCCGTTTGTGGGGGACAACCAGCCGAAAGGTTGGCTAATACCGCATAAGACCTGAGGGTGAAAGCCGGGGACCGCAAGGCCTGGCGCAGACGGAGCGGCCGATGATTGATTAGCTAGTTGGCGGGGTAAAGGCCCACCAAGGCGACGATCAATAGCTGGTCTGAGAGGACGACCAGCCACACTGGAACTGAGACACGGTCCAGACTCCTACGGGAGGCAGCAGTGGGGAATTTTGGACAATGGGGGCAACCCTGATCCAGCCATGCCGCGT
>FQH3XDB01CTKJ6|707|11
ATTGAACGCTGGCGGCATGCCTTACACATGCAAGTCGAACGGCAGCGCGGGAGCTTGCTCCTGGCGGCGAGTGGCGAACGGGTGAGTAATACATCGGAACGTGTCCGTTTGTGGGGGACAACCAGCCGAAAGGTTGGCTAATACCGCATAAGACCTGAGGGTGAAAGCCGGGGACCGCAAGGCCTGGCGCAGACGGAGCGGCCGATGATTGATTAGCTTGTTGCGGGTAAGGCCCACCAAGGCGACGATCAATAGCTGGTCTGAGAGGACGACCAGCCACACTGGAACTGAGACACGGTCCAGACTCCTACGGGAGGCAGCAGTGGGGAATTTTGGACAATGGGGGCAACCCTGATCCAGCCATGCCGCGTGCGGGAAGAAGGCC
>FQH3XDB01DFUJV|708|1
GATGAACGCTGGCGGCATGCCTAAGACATGCAAGTCGAACGCGAAGGCCCACTGACTTCTGTTGAAAACTAGAGAGCTTGCTCAAAGGTTGGAAACAGTTCGACGTGGATTTTCCTTCGAGTGGCAAACGGGTGAGTAACACGTGGGTTACCTGCCTCTTAGTTGGGGATAACGATTGGAAACGATCGATAATACCGAATGTACTCTACGGAGTAAAGAAGCCCTTAAAGCTTCGCTAAGAGATGGGCCTGCGGCGTATTAGCTAGTTGGTGGGGTAATGGCCTACCAAGGCAACGATACGTAGCCGAACTGAGAGGTTAATCGGCCACACTGGGACTGAGACACGGCCCAGACCTCCGTACGGGAGACAGCAGTTAGGAATATTCGTCAAGTGGGGGAAACCCTGAACGAGCAATGCCGCGTGAGTGATGAAGTCTTGGATTGTAAACT
>FQH3XDB01EJB7Q|709|3
GATTAACGCTGGCGGCATGCCTAAGACATGCAAGTCGAACGGAGTGCCCCACTGATACTTGAATGAAATTTGAGAGCTTGCTCGAAAATGGAATGACGGTGGATGTGGATTCTGCACTCAGTGGCGAACGGGTGAGTAACACGTGGGTTACCTGCCTCTAAGTTGGGGACAACAGTTGGAAACGACTGCTAATACCGAATGTGATAGTAATATTAAAGGAGCCTTTAAAAGCTTCGCTTAGAGATGGGCCTGCGGCGTATTAGCTTGTTGGTGGGTAATGGCCTACCAAGGCAACGATGCGTAGCCGGACTGAGAGGTTGATCGGCCACACTGGGACTGA
>FQH3XDB01BW2BT|710|2
AGCGAACGCTGGCGGCAGGCCTAACACATGCAAGTCGAGCGGCCATAGCAATATGGCAGCGGCAGACGGGAGAGTAACACGTGGGAACGTACCCTTCGGTTCGGAATAACTCAGGGAAACTTGAGCTAATACCGGATACGTCCGTAAGGAGAAAGATTTATCGCCGAGGGATCGGCCCGCGTCCGATTAGCTAGTTGGTGGGGTAATGGCCCACCAAGGCGACGATCGGTAGCTGGTCTGAGAGGATGATCAGCCTCACTGGGACTGAGACACGGCCCAGACTCCTACGGGAGGCAGCAGTGGGGAATATTGGACAATGGGCGCAAGCCTGATCCAGCCATGCCGCGTGGGTGATGAAGGCCCTAGGGTTGTAAA
>FQH3XDB01B3LNP|711|1
AGCGAACGCTGGCGGCAGGCCTAATACATGCAAGTCGAACGCACCTTCGGGTGAGTGGCGGACGGGTGAGTAACACGTGGGAACGTGCCCTTTGGTTCGGAATAATCCGGGGAAACCTGGCCTAATACCGGATGTGCCCCTTCGGGGGAAAGATTTATCGCCATTGGAGCGGCCCGCGTCTGATTAGCTAGTTGGTGAGGTAAAGGCTCACCAAGGCTACGATCAGTAGCTGGTCTGAGAGGATGATCAGCCACACTGGGACTGAGACACGGCCCAGACTCCTACGGGAGGCAGCAGTAGGGAATCTTGCGCAATGGGCGAAAGCCTGACGCAGCCATGCCGCGTGAATGATGAAGGTCTTAGGATTGTAAAATTCTTTCGAGCGGGGAAGATAATGACTGTACCCGCAGAAGAAGCTCCGGCTAACTTCGTGCCAGCAG
>FQH3XDB01CZV0I|712|114
AGTGAACGCTGGCGGCGTGGCTAAGACATGCAAGTTGAGCGAGTAGATACCGCGAGGTATTGAGGAGCAGCGAACGGGTGAGTAAGACGTAAGTAACCTACCCTTAAGACGAGGATAGACTATCGAAAGGTAGAGTAATACTGGATGGAGGATATCTCTGCATGGGGATAGACCGAAAGGCAAGGAAATGCGCTAAAGGAGGGGCTTGCGGCCTATCAGCTAGTTGGTGGGTAAGAGCCGACCAAGGCGAAGACGGGTAGCCGGTCT
>FQH3XDB01DQ90S|713|1
AGCGAACGTTAGCGGCAGGCTTAACACATGCAAGTCGAGCGAGAAAGGGGGCAACCCCTGAGTAAAGCGGCGGACGGGTGCGTAACACGTGGATAATCTGCCCTCTAGTGGAGAACAACAGTCCGAAAGGATTGCTAATGCTGCATGTCACAAGTGAGTCTCCGGGCTTGCTTGGAAAAAGAGGGCGCTCGCAAGAGGCTCTCGCTAGAGGATGAGTCCGCGGCCCATCAGCTAGTTGGCGGGGTAATGGCCCACAAGGCTTTGACGGGTAGCTGGTCTGAGAGGATGACCAGCCACACTGGGACTGAGAC
>FQH3XDB01BBNIV|714|1
AGCGAACGTTAGCGGCAGGCTTAACACATGCAAGTCGAGCGAGAAAGAGGGGCAACCCTTGAGTAAAGCGGCGGACGGGTGCGTAACACGTGGATAATCTGCCCTCTAGTGGGGAACAACAGTCCGAAAGGATTGCTAATGCCGCATGTCACGGCTAGGACCCTGGTCCTGGTCGGCAAAGAGGGCGCCCGCAAGGGGCTCTCGCTAGAGGATGAGTCCGCGGCCCATCAGCTTGTTGGCGGGGTAATGGCCCACCAAGGCTTTTGACGGGTAGCGGGTCTGAGAGGATGACCCGCCACACTGGGACTGAGACACGGCCCCAGACTCCTACGGGAGGCAGCAGTGGGGAATATTGGGGCAATGGGCGAAAGCCTGACCCAGCCATGCCGCGTGAGTGATGAAGGCCTTCGGGTCGTAAAGCTCTGTGGGAAGGGACGAAAAAACGGACCTAATACGTCCGGGCTTGACGGTACTCTTAGCAAGCACCGGCTAACTCCGTGCCAGCAGCCG
>FQH3XDB01C0RXM|715|1
AGCGAACGTTAGCGGCGCGCCTAACACATGCAAGTCGCACGAGAAGAGGGCAACCTCTGAAAGTGGCGCACGGGTGCGTAACACGTAGATAACCTGCCCTTCGTTGGGGGATAACGTTCCGAAAGGAGCGCTAATACCGCGTACAGCGTTTGAGGGTTTGCCCTTGGACGGCAAAGGCTGGGCTTCGCAAGAACCAGTCGACGAAGGAGGGGTCTGCGGCCCATCAGCTAGTTGGTGAGGTAATGGCTCACCAAGGCAAAGACGGGTAGCTGGTCTGAGAGGATGATCAGCCACACTGGTACTGGAACACGGACCAGACTCCTACGGGAGGCAGCAGTGGGGAATCTTGGTCAATGGGGCGAAAGCCTGAACCAGCGACGCCGCGTGAGTGATGAAGGCCTTCGGGTTGTAAAGCTCTGTGGGCGAGACGAAGCCTACGTTCTAACCGGACGTAGTTGACGGTATCGCCTTAGCAA
>FQH3XDB01DK43N|716|1
AGCGAACGTTAGCGGCGGGCCTAACACATGCAAGTCGAGCGAGAAAGTGGCTTCGGCCATCAGTAAAGCGGCGGACGGGTGAGTAACACGTAGGTAATCTTCCCTTGAGCGGGGGACAACGAGGCGAAAGCCTCGCTAATACCGCATAAGTTCGTAGAGACTCCGGTCTTTACGAGGAAAGCTAGGTAGCAATATCCGGCACTCGAGGAGGAGCCTGCGTCGCATCAGCTAGTTGGTGGGGTAACGGCCTACCAAGGCGAAGACGCGTAGCTGGTCTGAGAGGATGATCAGCCACACTGAAACTGAGACACGGTTCAGACTCCTACGGGAGGCAGCAGTGGGGAATATTGGGACAATGGGCGCAAGCCTGATCCAGCCACGCCGCGTGAGTGATGAAGGCCTTCGGGTCGTAAAGCTCTGTGGGGAGGGACGAACCGCCGTTGGTTAACTACCAACGGCATGACGGT
>FQH3XDB01DWUG0|717|29
AGCGAACGTTGGCGATGCGTCTTAAGCATGCAAGTCGAGCGGGCTTATTCGGGCAACTGGATAAGTTAGCGGCGAACTGGTGAGTAACACGTAGGTAATCTGCCGTAGAGTGGGGATAACCCATGGAAACATGGACTAATACCGCATATACTCTTGACACATAAGTGTAGTAGAGGAAAGGAGCAATCCGCTTTACGATGAGCCTGCGGCCTATTAGCCTGTTGGTGAGATAAAAGCCCACCAAAGCTACGATAGGTAGCCGACCTGAGAGGGTGACCGGCCACATTGGGACTGAGATACGGCCCAGACTCCTACGGGAGCAGCAGCTGAGAATCTTCCACAATGGACGAAAGTCTGATGGAGCGACATCGCGTGAGGGATGAATGCC
>FQH3XDB01BFVP1|718|2
AGCGAACGTTGGCGATGCGTCTTAAGCATGCAAGTCGAGCGGGCTTATTTGGGTAACTGGATAAGTTAGCGGCGAACTGGTGAGTAACACGTAGGTAATCTGCCGTAGAGTGGGGGATAACCCATGGAAACATGGACTAATACCGCATATACTCTTGACACATAAGTGTAGTAGAGGAAAGGAGTAATCCGCTTTACGATGAGCCTGCGGCCTATTAGCCTGTTGGTGAGGTAAAAGCTTACCAAAGCTACGATAGGTAGCCGACCTGAGAGGGTGACCGGCCACATTGGGACTGAGATACGGCCCAGACTCCTACGGGAGGCAGCAGCTGAGAATCTTCCACAATGGACGAAAGTCTGATGGAGCGACATCGCGTGAGGGATGAAGGCCTTCGGGGTTGTAAACCCGTTGGAAATTATCGAAGAATGAGTGACAGTAGATAATGTAAGCCTCGGCTAACTACGTGCCAGCAGCCG
>FQH3XDB01C3G34|719|1
GATGAACGCTGGCGGCATGCCTAAGACATGCAAGTCGAACGAAGGGAACCAAGGAAGGAATATTGAAGTTGGAGAGCTTGCTCAAAGATGGAAATAAACTGGACATGGATTTTCCCTTAGTGGCAAACGGGTGAGTAACACGTGGGTAACCTGCCTTCAAGATGGGGATAACAGTTGGAAACGATTGCTAATACCGAATGTGATCTATGGATTAAAGAAGCTCCAAAGCTTCGCTTGAAGATGGGCCTGCGGTGCATTAGCTAGGTTGGTAAGGTATGGCTTACCAAGGCGACGATGCATACGCGAACTGAGAGGTTAATCGGCCA
>FQH3XDB01D7O5I|720|6
GATGAACGCTGGCGGCATGCCTAAGACATGCAAGTCGAACGGAGGGACCCAATGACGTTTTATGAAGTTTTGCGAGCTTGCTCAAGAAATGGATTAAAATTGATTTGGATTATCCCTTAGTGGCAAACGGGTGAGTAACACGTGGGTTACCTACCTCCAAGTCGGGGACAACAGTTGGAAACGATTGCTAATACCGGATGTGGACTACGGTTTAAAGAAGCCTTTAAAGCTTCGCTTGGAGATGGGCCTGCGGTGCATTAGCTAGTTGGTGGGGTAATGGCCTACCAAGGCGACGATGCATAGCCGAACTGAGAGGTTAATCGGCCACATTGGGACTGAGACACGGCCCAAAACTCCTAACGGGAGACAGCAGTTAGGAATATTCGTCAAGTGGGGAAACCCTGAACGAGCAATGCCGCGTGAGTGATGACGGT
>FQH3XDB01AUBLV|721|1
GATGAACGCTGGCGGCATGCCTAAGACATGCAAGTCGAACGGAGGGACCCAATGACGTTTATGAAGTTTTGCGAGCTTGCTCAAAAATGGATTAAATTGATTTGGATTATCCCTTAGTGGCAAACGGGTGAGTAACACGTGGGTTACCTACCTCCAAGTCGGGGACAACAGTTGGAAACGATTGCTAATACCGGATGTGGACTACGGTTTAAAGAGCCTTTAAAAGCTTCGCTTGGAGATGGGCCTGCGGTGCATTAGCTAGTTGGTGGGATAACGGCCTACCAAGGCGACGATGCATAGCCGAACTGAGAGGTTAATCGGCCACATTGGGACTGAGACA
>FQH3XDB01DPE5S|722|4
GATGAACGCTGGCGGCATGCCTAAGACATGCAAGTCGAACGAGAGGGCCCATTGATAGTTATTGAAGTTATGCGAGCTTGCTCAATTAATGGATTTAACTTGATTTGGATTTTCCCTCTAGTGGCAAACGGGTGAGTAACACGTGGGTTACCTACCTCTAAGTTGGGGACAACAGTTGGAAACGACTGCTAATACCGAATGTGATCTACGGATTAAAGGAGCCTTTAAAGCTTCGCTTAGAGATGGGCCTGCGGTGCATTAGCTAGTTGGTGGGTAATGGCCTACCAAGGCGACGATGCATAGCCGAACTGAGAGGTTAATCGGCCACACTGGGGACTGAGACACGGCCCAGACTCC
>FQH3XDB01CBNH6|723|1
GATGAACGCTGGCGGCATGCCTAAGACATGCAAGTCGAACGAGAGGGCCCATTGATAGTTATTGAAGTTATGCGAGCTTGCTCAATTAATGGATTTAACTTGATTTGGATTTTCCCTCTAGTGGCAAACGGGTGAGTAACACGTGGGTTACCTACCTCTAAGTTGGGGACAACAGTTGGAAACGACTGCTAATACCGAATGTGATCTACGGATTAAAGGGAGCCCTTTTAAAAGCTTCGCTTAGAGATGGGCCTGCGGTGCATTAGCTAGTTGGTGGGTAATGGCCTACCAAGGCGACGATGCAT
>FQH3XDB01EVYKK|724|34
AGTGAACGCTGGCGACGTGGTTAAGACATGCAAGTCGAGCGGTATTGAAGTTGTAGCAATACAATGGAGAGAGAGCGGCGAACGGGTGCGTAACACGTGAGAAATCTGCCTTGAAGTTTGGAATAGCCCGGGGAAACTCGGATTAATGCCGGATGTGGTGGCGAGCGGCATCGCTTGTCAACTAAAGTTTGAGACGACGCTTCAAGAGGGGTCTCGCGGCCTATCAGCTTGTTGGTGAGGTAACGGCTCACCAAGGCGAAGACGGGTAGCTGGTCTGAGAGGATGATCAGCCACACTGGAACTGAGACACGGTCCAGACACCTACGGGTGGCAGCAGTTTCGAATCATTCACAATGGGGGCAACCCTGATGGTGCGACGTCGCGTGGGGGATGAAGGTCTTCGGATTGTAAACCTCTGTCAAGGGGGATAATGTATGGTTGTAGCCATATTTGAATTAACCTCTAGAGGAAGCAGTGGCT
>FQH3XDB01BN5PM|725|384
AGTGAACGCTGGCGACGTGGTTAAGACATGCAAGTCGAGCGGTATTGAAGTTGTAGCAATACAATGGAGAGAGAGCGGCGAACGGGTGCGTAACACGTGAGAAATCTGCCTTGAAGTTTGGAATAGCCCGGGGAAACTCGGATTAATGCCGGATGTGGTGGCGAGCGGCATCGCTTGTCAACTAAAGTTTGAGACGACGCTTCAAGAGGGTCTCGCGGCCTATCAGCTTGTTGGTGAGGTAACGGCTCACCAGGCGAAGACGGGTAGCTGGGTCTGAGAGGATGATCAGCCA
>FQH3XDB01E0FWV|726|2
AGTGAACGCTGGCGACGTGGTTAAGACATGCAAGTCGAGCGGTATTGAAGTTGTAGCAATACAATGGAGAGAGAGCGGCGAACGGGTGCGTAACACGTGAGAAATCTGCCTTGAAGTTTGGAATAGCCCGGGGAAACTCGGATTAATGCCGGATGTGGTGGCGAGCGGCATCGCTTGTCAACTAAAGTTTGAGACGACGCTTCAAGAGGGTCTCGCGGCCTATCAGCTTGTTGGTGAGGTAACGGCTCACCGAAGGCGAAGGACGGGTAGCTGGTCTGAGAGGATGATCAGCC
>FQH3XDB01D9KAI|727|1
AGTGAACGCTGGCGACGTGGTTAAGACATGCAAGTCGAGCGGTATTGAAGTTGTAGCAATACAATGGAGAGAGAGCGGCGAACGGGTGCGTAACACGTGAGAAATCTGCCTTGGAAGTTTGGAATAGCCCGGGAAACTCGGATTAATGCCGGATGTGGTGGCGGAGCGGCCATCGGCTTAGGTCAACTAAAGTTTGAGACGACGCTTCAAGAGGGTCTCGCGGCCTATCAGCTTGTGGTGAGGTAACGGCTCACCAAGGCGAAGACGGGTTAGCTGGTCT
>FQH3XDB01C8BEO|728|9
AGTGAACGCTGGCGACGTGGTTAAGACATGCAAGTCGAGCGGTATTGAAGTTGTAGCAATACAATGGAGAGAGAGCGGCGAACGGGTGCGTAACACGTGAGAAATCTGCCTTGAAGTTTGGAATAGCCCGGGGAAACTCGGATTAATGCCGGATGTGGTGGCGAGCGGCATCGCTTGTCAACTAAAGTTTGAGACGACGCTTCAAGAGGGTCTCGCGGCCTATCAGCTTGTTGGTGAGGTAACGGCTCACCAAGGCGAAGACGGGTAGCTGGTCTGAGAGGATGATCAGCCACACTGGAACTGAGACACGGTCCAGACACCTACGGGTGGCAGCAGTTTCGAATCATTCACAAGTGGGGCAACCCTGATGGTGCGACGTCGCGGTGGGGATGAAGGTCTTCGGATTGTAAACCTCTGTCGAAGGGG
>FQH3XDB01B6ZYA|729|21
AGTGAACGCTGGCGGCGTGCCTAATACATGCAAGTCGAACGATGAAGCTTCTAGCTTACTAGAAGTGGATTAGTGGCGCACGGGTGAGTAAGGTATAGTTAATCTGCCTTTTGCTGGGGGACAACACTTAGAAATGAGTGCTAATACCCCATACTCCATTATGACACAAGTTATAATGGGAAAAGTTTTTCGGCAAAAGATGAGACTATATAGTATCAGCTTGTTGGTGAGGTAATGGCTTACCAAGGCTATGACGCTTAACTGGTCTGAGAGGATGATCAGTCACATTGGAACTGAGACACGGTCCAAAACTCCTACGGGAGGCAGCAGTAGGGAATATTGCGCAATGGGCGAAAGCCTGACGCAGCAACGCCGCGTGGAGGATGACACTTTTCGGAGCGTAAACTCCTTTTCTTTGGGAAGTAATTTTGACGGTACCAAGGATAAGCACGGCTAACTCCGTGCCAGCAG
>FQH3XDB01D7G2S|730|4
AGTGAACGCTGGCGGCGTGCCTAATACATGCAAGTCGAACGATGAAGCTTCTAGCTTGCTAGAAGTGGATTAGTGGCGCACGGGTGAGTAAGGTATAGTTAATCTGCCCTTTGCTGGGGGACAACACTTAGAAATGAGTGCTAATACCCCATACTCCGCTACAACACAAGTTGTGGTGGGAAAAGTTTTTCGGCAAAAGGATGAGACTATATAGTATCAGCTTGTTGGTGAGGTAATGGCTTACCAAGGCTATGACGCTTAACTGGTCTGAGAGGATGATCAGTCACATTGGAACTGAGACACGGTCCAAAACTCCTACGGGAGCAGCAGTAGGGAATATTGCGCAAATGGGCGAAAGCCTGACGCAGCAACGCCGCGTGGAGGATGACACTTTTCGGAGCGTAAACTCCTTTTCTTGGGAAGTATTTTGACGTACAAGGATAAGCACCGGCTAACTCCGTGCCAGCAGCC
>FQH3XDB01A3U9F|731|9
AGTGAACGCTGGCGGCGTGCCTAATACATGCAAGTCGAACGATGAAGCTTTTAGCTTGCTAGAAGTGGATTAGTGGCGCACGGGTGAGTAAGGTATAGTTAATCTGCCTTTTGCTGGGGGACAACATTTAGAAATGAGTGCTAATACCCCATACTCCGCATTTGCACAAGCAAATGTGGGAAAGTTTTTCGGCAAAAGATGAGACTATATAGTATCAGCTTGTTGGTGAGGTAATGGCTTACCAAGGCTATGACGCTTAACTGATCTGAGAGGATGATCAGTCACATTGGAACTGAGACACGGGTCCAAAACTCCTACGGGAGGCAGCAGTAGGGAA
>FQH3XDB01CWAKC|732|2
AGTGAACGCTGGCGGCGTGCCTAATACATGCAAGTCGAACGATGAAGCTTTTAGCTTGCTAGAAGTGGATTAGTGGCGCACGGGTGAGTAAGATATAGTTAATCTGCCTCGCACTGGGGGACAACATTTAGAAATGAGTGCTAATACCCCATACTCCACCATAACACAAGTTATGGTGGGAAAGTTTTTCGGTGCGAGATGAGACTATACAGTATCAGCTAGTTGGTGAGGTAATGGCTTACCAAGGCTATGACGCTTAACTGGTCTGAGAGGATGATCAGTCACATTGGAACTGAGACACGGTCCAAACTCC
>FQH3XDB01A0DDO|733|2
AGTGAACGCTGGCGGCGTGCCTAATACATGCAAGTCGAACGATGAAGCCTTTAGCTTGCTAGAGGTGGATTAGTGGCGCACGGGTGAGTAACGCATAGGTAATATGCCCTTTGGTCTGGGGATAGCCACTGGAAACGGTGATTAATACTAGATACTCCCTACGGGGGAAAGAATTTCGCCAAAGGATTAGCCTATGTCCTATCAGCTTGTTGGTGAGGTAATGGCTCACCAAGGCTATGACGGGTATCCGGCCTGAGAGGGTGAACGGACACACTGGAACTGAGACACGGTCCAGACTCCTACGGGAGGCAGCAGTAGGGAATATTGCTC
>FQH3XDB01BCH4M|734|1
AGTGAACGCTGGCGGCGTGCCTAATACATGCAAGTCGAACGATGAAGCTTCTAGCTTGCTAGAAGTGGATTAGTGGCGCACGGGTGAGTAATGCATAGGTTATGTGCCCTTTAGTCTAGGATAGCCACTGGAAACGGTGATTAATACTGGATACTCCCTACGGGGGAAAGAATTTCGCTAAAGGATCAGCCTATGTCCTATCAGCTTGTTGGTGAGGTAATGGCTCACCAAGGCTATGACGGGTATCCGGCCTGAGAGGGTGAACGGACACACTGGAACTGAGACACGGTCCAGACTCCTACGGGAGGCAGCAGTAGGGAATATTGCTCAATGGGGGAAACCCTGAAGCAGCAACGCCGCGTGGAGG
>FQH3XDB01DZREH|735|4
AGTGAACGCTGGCGGCGTGGCTAAGACATGCAAGTCGAGCGGGAATATTTGATGTAGCAATACATTGGATACGAGAGCGGCGAACGGGTGAGTAATACATAAGCAATCTGCCTTTAAATTGGGGATAGCCCAGGGAAACTTGGATTAATACCGAATGTGGTGCACCACTGCATGGTGGTGCTACTAAAGCTTGTGATGGCGTTTAAAGAGGAGCTTATGGCCTATCAGCTTGTTGGTAGAGGTAACGGCCTACCAAGGCAAAGACGGGTAGCGGGTCTGAGAGGACGATCCGCCACACTGGAACTGAGACACGGTCCAGACACCTACGGGTGGCAGCAGTTTCGAATCATTCACAATGGGGAAACCCTGATGGTGCGACGCCGCGTGGGGGATGAAGG
>FQH3XDB01CFGWI|736|3
AGTGAACGCTGGCGGCGTGGCTAAGACATGCAAGTCGAGCGAGAATATGCTTAGGCATAGGGAAGCGGCGAACGGGTGAGTAACACGTAAGTAACCTACCCTTAAGACAGGGATAGCTCAGCGAAAGTTGAGGTAATACCGGATAAAGACCCTAATTGCATGATTAGGGATCCAAAGGGGCGGGAAGACGCTTAAGGAGGGGCTTGCGGCCTATCAGCTAGTTGGAGGTGTAAAGGACCCCCAAGGCGAAGACGGGTAGCCGGTCTGAGAGGACGGACGGCCACATTGGAACTGAGACACGGTCCAAACATCTACGGATGGCAGCAGTTTCGAATTATTCACAATGGGCGAAAGCCTGATGGTGCGACGTCGCGTGGAGGAAGACGG
>FQH3XDB01AGWXB|737|2
AGTGAACGCTGGCGGCGTGGCTAAGACATGCAAGTCGAGCGAGAATATGCTTCGGCATAGGGAAGCGGCGAACGGGTGAGTAACACGTAAGTAACCTACCCTTAAGACAGGGATAGCTCAGCGAAAGTTGAGGTAATACCGGATAAAGACCCTAATTGCATGATTAGGGATCCAAAGGAATGAGAGATCGCTTAAGGAGGGGCTTGCGGCCTATCAGCTAGTTGGAGGTGTAAAGGACCCCCAAGGCGAAGACGGGTAGCCGGTCTGAGAGGACGGACGGCCACATTGGAACTGAGACACGGTCCAAACATCTACGGATGGCAGCAGTTTCGAATTATCACAATGGGCGAAAGCCTGATGGTGCGACGTCGCGTGGAGGAAGAAGGTCTTCGGATTGTAAACTCCTGTCAGTTAGGGAGC
>FQH3XDB01CCQF3|738|2
AGTGAACGCTGGCGGCGTGGCTAAGACATGCAAGTCGGGCGAGAATGTGCCTTCGGGCACAGGGGAAGCGGCAAACGGGTGAGTAACACGTAAGTAACCCTGCCCTTGAGACTGGGATAGCTCAGCGAAAGTTGAGGTAATACCGGATGACAACCCTTAATGCATGTTAAGGGATTCAAAGGAATGAGAAGTCGCTCAAGGAGGGGCTTGCGGCCTATCAGCTAGTTGGTGATGTAACGGACCACCAAGGCAAAGCGGGTAGCCGGTCTG
>FQH3XDB01EGZUL|739|12
AGTGAACGCTGGCGGCGTGGCTAAGACATGCAAGTTGAGCGAGTAGATACCGCGGGGTATTGAGGAGCAGCGAACGGGTGAGTAAGACGTAAGTAACCTACCCTATAGACGAGGATAGGCTATCGAAAGGTAGTGTAATACTGGATGGAGGATATATCTGCATAGATATAGACCGAAAGGCAAGAGAATGCGCTAAAGGAGGGGCTTGCGGCCTATCAGCTAGTTGGTTGGGTAAGAGCCGACCAAGGCGAAGACGGGTAGCCGGTCTGAGAGGACGGACGGCCACATTGGAACTGAGACACGGTCCAAACATCTACGGATGGCAGCAGTTTCGAATTATTCACAATGGGCGAAAGCCTGATGGTGCGACGTCGCGTGGAGGAAGAAGGTCCTAGGATTGTAAACTCCTGTCATTAGGGA
>FQH3XDB01AGXX9|740|17
AGTGAACGCTGGCGGCGTGGCTAAGACATGCAAGTTGAGCGAGTAGATACCGCGAGGTATTGAGGAGCAGCGAACGGGTGAGTAAGACGTGAGTAACCTACCCTATAGACGAGGATAGGCTATCGAAAGGTAGTGTAATACTGGATGGAGGATATATCTGCATAGGTATAGACCGAAAGGCAAGAGAATGCGCTAAAGGAGGGGCTTGCGGCCTATCAGCTAGTTGGTTGGGTAAGAGCCGACCAAGGCGAAGACGGGTAGCCGGTCTGAGAGGACGGACGGCCACATTGGAACTGAGACACGGTCCAAAACATCTACGGATGGCAGCAGTTTTCGAATTATTCACAATGGGCGAAGCCTGATGGTGCGACGTCGCGTGGAGGAAGAAGGTCCTAGGATTGTAAACTCCTGTCATTAGGGA
>FQH3XDB01C2TRD|741|56
AGTGAACGCTGGCGGCGTGGCTAAGACATGCAAGTTGAGCGAGTAGATACCGCGAGGTATTGAGGAGCAGCGAACGGGTGAGTAAGACGTAAGTAACCTACCCTTAAGACGAGGATAGACTATCGAAAGGTAGAGTAATACTGGATGAAGGATATTTCCGCATGGGGATAGACCGAAAGGCTAGAGAGAGCGCTAAAGGAGGGGCTTGCGGCCTATCAGCTAGTTGGTTGGGTAAGAGCCAACCAAGGCGAGGACGGGTAGCCGGTCTGAGAGGACGGACGGCCACATTGGAACTGAGACACGGTCCAAACATCTACGGATGGCAGCAGTTTCGAATTATTCACAATGGGCGAAAGCCTGATGGTGCGACGTCGCGTGGAGG
>FQH3XDB01AESCI|742|2
AGTGAACGCTGGCGGCGTGGCTAAGACATGCAAGTTGAGCGAGTAGATACCGTGAGGTATTGAGGAGCAGCGAACGGGTGAGTAAGACGTAAGTAACCTACCCTTAAGACGAGGATAGACTATCGAAAGGTAGAGTAATACTGGATGAAGGATATTCCGCATGGGGATAGACCGAAAGGCTAGAGAGAGCGCTAAAGGAGGGCTTGCGGCCTATCAGCTAGTTGGTTGGGTAAGAGCCAACCAAGGCGAAGACGGGTAGCCGGTCTGAGAGGACGGACGGCCACATTGGAACTGAGACACGGTCCAAACATCTACGGATGGCAGCAGTTTCGAATTATTCACAATGGGCCGAAAGCCTGATGGTGCGAACGTCGCGTGGAAGGAAG
>FQH3XDB01AQCGD|743|1
AGTGAACGCTGGCGGCGTGGCTAAGACATGCAAGTTGAGCGAGTAGATACCGCGAGGTATTGAGGAGCAGCGAACGGGTGAGTAAGACGTAAGTAACCTACCCTTAAGACGAGGATAGACTATCGAAAGGTGAGTAATACTGGATGAAGGATATTTCCCGGCAATGGGGGATAGACCGAAAGGCTAGAGAGAGCGCTAAAGGAGGGGCTTGCGGCCTATCAGCTAGTGGTTGGGTAAGAGCCAACCAAGGCGAAGACGGGTTAGCCGGTCTGAGAGGACGGA
>FQH3XDB01B61PF|744|78
GGTGAACGCTGGCGGCGTGGCTAAGACATGCAAGTTGAGCGAGTAGATACCGTGAGGTATTGAGGAGCAGCGAACGGGTGAGTAAGACGTAAGTAACCTACCCTTAAGACGAGGATAGACTATCGAAAGGTAGAGTAATACTGGATGAAGGATATTTCCGCATGGGGATAGACCGAAAGGCTAGAGAGAGCGCTAAAGGAGGGGCTTGCGGCCTATCAGCTAGTTGGTTGGGTAAGAGCCAACCAAGGCGAAGACGGGTAGCCGGTCTGAGAGGACGGACGGCCACATTGGAACTGAGACACGGTCCAAAACATCTACGGATGGCAGCAGTTTCGAATTATTCACAATGGGCAAAGCCTGATGGTGCGACGTCGCGTGGAGGAAGAAGGTTCTAGGATTGTAAACTCCTGTCATT
>FQH3XDB01AXTRK|745|16
AGTGAACGCTGGCGGCGTGGCTAAGACATGCAAGTTGAGCGAGTAGATACCGTGAGGTATTGAGGAGCAGCGAACGGGTGAGTAAGACGTAAGTAACCTACCCTTAAGACGAGGATAGACTATCGAAAGGTAGAGTAATACTGGATGAAGGATATTTCCGCATGGGGATAGACCGAAAGGCTAGAGAGAGCGCTAAAGGAGGGCTTGCGGCCTATCAGCTAGTTGGTTGGTAAGAGCCAACCAAGGCGAAGACGGGTAGCCGGTCTGAGAGGACGGACGGCCACATTGGAACTGAGACACGGTCCAAACATCTACGGATGGCAGCAGTTTCGAATTATTCACAATGGGCGAAAGCCTGATGGTGCGACGTCGCGTGGAGGAAGAAGGTTCTAGGATTGTAAACTCCTGTCATTAGGGAGCAAAAGGCTGTGGAGAGCAGCGGATTGAGTAAACCTAAGAGGAAGCAGTGGCAAACTCCGTGCCAGC
>FQH3XDB01DPFQY|746|1
AGTGAACGCTGGCGGCGTGGCTAAGACATGCAAGTTGAGCGAGTAGATACCGTGAGGTATTGAGGAGCAGCGAACGGGTGGGTAAGACGTAAGTAACCTACCCTTAAGACGAGGATAGACTATCGAAAGGTAGAGTAATACTGGATGAAGGATATTTCCGCATGGGGATAGACCGAAAGGCTAGAGAGAGCGCTAAAGGAGGGGCTTGCGGCCTATCAGCTAGTTGGTGGGTAAGAGCCAAACCAAGGCGAAGACCGGGTAGCCGGTCTGAGAGGACGGACGGCCACATTGGAACTGAGACACGGTCCAAAC
>FQH3XDB01BNS3B|747|4
AGTGAACGCTGGCGGCGTGGCTAAGACATGCAAGTTGAGCGAGTAAATACCGCAAGGTATTGAGGAGCAGCGAACGGGTGAGTAAGACGTAAGTAACCTACCCTATAGACGAGGATAGGCTATCGAAAGGTAGTGTAATACTGGATGGAGGATATATCTGCATAGGTATAGACCGAAAGGCAAGAGAATGCGCTAAAGGAGGGCTTGCGGCCTATCAGCTAGTTGGTTGGGTAAGAGCCGACCAAGGCGAAGACGGGTAGCCGGTCT
>FQH3XDB01AZZ4D|748|2
AGTGAACGCTGGCGGCGTGGTTAAGACATGCAAGTCGAACGAGATTTGGCTGACCGTAAGGAAGGTCAATGAAAGTGGCAAACGGGTGCGTAACACGTAAACAACTTGCCCTTTAGTTGGGGATAGCTCGCTGAAAAGGTGAATTAATACCGAATGTGGTTGTTTTTCGCATGAAAAACATACTAAAGCTTGAAATGGCGCTAAAGGAGAGGTTTGCGGCCTATCAGCTAGTTGGTGAGGTAATGGCTCACCAAGGCTAAGCGGTAGCTGGTCTGAGAGGATGATCAGCC
>FQH3XDB01BSTQ5|749|6
AGTGAACGCTGGCGGCGTGGTTAAGACATGCAAGTCGAGCGAGAACAAAAGCGTAGCAATACGTGGATGAGAAAGCGGCGAACGGGTGCGTAACACGTAAGCAACCTGCCCTAAAGACGGGGATAGCTCGGGGAAACTCGAATTAATACCGGATGTGGTCCTTAAACACATGTAAGAGGAACTAAAGCTTGAGAAGGCGCTTTAGGAGGGCTTGCGGCCTATCAGCTAGTTGGTAAGGTAACGGCTTACCAAGGCAAAGACGGGTAGCGGGTCTGAGAGGACGATCCGCCACACTGGAACTGAGACACGGTCCAGACACCTACGGGTGGCAGCAGTTTCGAATCATTCACAATGGGGGCAACCCTGATGGTGCAACGCCGCGTGGGGACGAAGGTCTTCGGATTGTAAACCCCTGTCACACAGGACTAACCATAAGGTTCATAGCCTTATCTGAATTAACTGTG
>FQH3XDB01CJ7LZ|750|5
AGTGAACGCTGGCGGCGTGGTTAAGACATGCAAGTCGAGCGAGAACAAAAGCGTAGCAATACGTGGATGAGAAAGCGGCGAACGGGTGCGTAACACGTAAGCAACCTGCCCCTAAAGACGGGGATAGCTCGGGGAAACTCGAATTAATACCGGATGTGGTGCTTTAACGCATGTTAAAAGCTACTAAAGCTTGAGAAGGCGCTTTAGGAGGGGCTTGCGGCCTATCAGCTTGTTGGTGAGGTAAAGGCTCACCAAGGCAAAGACGGGTAGCGGGTCTGAGAGGACGATCCGCCACACTGGAACTGAGACACGGTCCAGACACCTACGGGTGGCAGCAGTTTCGAATCATTCACAAG
>FQH3XDB01AIZDS|751|8
AGTGAACGCTGGCGGCGTGGTTAAGACATGCAAGTCGAGCGAGAACAAAAGCGTAGCAATACGTGGATGAGAAAGCGGCGAACGGGTGCGTAACACGTAAGCAACCTGCCCTAAAGATTGGGATAGCTCGGGGAAACTCGAATTAATACCGGATGTGGTCCTTTGACGCATGTTGAAGGCACTAAAGCTTGAGAAGGCGCTTTAGGAGGGGCTTGCGGCCTATCAGCTTGTTGGTGAGGTAACGGCTCACCAAGGCAAAGACGGGTAGCGGGTCTGAGAGGACGATCCGCCACACTGGAACTGAGACACGGTCCAGACACCTACGGGTGGCAGCAGTTTCGAATCATTCACAATGGGGGCAACCCTGATGGTGCAACGCCGCGTGGGGATGAAGGTCTTCGGATTGTAAACCCCTGTCACACAGGACTAACCGTAAGGTTCATAGCCTTACCTGAATTAACTGTGAGAGGAAGTAGTGGCTAACTCCGTGCCAGCA
>FQH3XDB01BSNZR|752|10
AGTGAACGCTGGCGGCGTGGTTAAGACATGCAAGTCGAGCGAGAACAAAAGCGTAGCAATACGTGGATGAGAAAGCGGCGAACGGGTGCGTAACACGTAAGCAACCTGCCTTAAAGACGGGGATAGCTCGGGGAAACTCGAATTAATACCGGATGTGGTTCTTCAACGCATGTTGAAGATACTAAAGCTTGAAATGGCGCTTTAAGAGGGGCTTGCGGCCTATCAGCTTGTGGTGAGGTAACGGCTCACCAAGGCAAAGACGGGTAGCGGGTCTGAGAGGACCGATCCGCCACACTGGAACTGAGACACGGTCCAGACACCTACGGGTGGCAGCAGTTTCGAATCATTCAC
>FQH3XDB01A04IB|753|1
AGTGAACGCTGGCGGCGTGGTTAAGACATGCAAGTCGAGCGGGATTTATTGCTACAAGTCTTCGGATGCGTAGTGATGATGAGAGCGGCAAACGGGTGAGTAACACGTAAACAACTTGCCCTTTAGTTGGGGATAGCTCGCTGAAAGGCGGATTAATACCGAATGTGATTTTTTCTGCATGGGAAAAATATTAAAGCTTGTAACGGCGCTAAAGGAGAGGTTTGCGGTCTATCAGCTTGTTGGTAAGGTAACGGCTTACCAAGGCAAAAGACGGATAGCTGGTCTGAGAGGATGATCAGCCACATTGGAACTGAGACACGGTCCAG
>FQH3XDB01DS24Q|754|1
AGTGAACGCTGGCGGTAGGCCTAACACATGCAAGTCGAACGGCAGCACAGGAGAGCTTGCTCTCTGGGTGGCGAGTGGCGGACGGGTGAGGAATGCATCGGAATCTACCTTTTCGTGGGGGATAACGTAGGGAAACTTACGCTAATACCGCATACGACCTTCGGGTGAAAGCAGGGGACCTTCGGGCCTTGCGCGGATAGATGAGCCGATGTCGGATTAGCTAGTTGGCGGGTAAAGGCCCACCAAGGCGACGATCCGTAGCTGGTCTGAGAGGATGATCAGCCACACTGGAACTGAGACACGGTCCAGACTCCTACGGGGAGGCAGCAGTGGGGAAT
>FQH3XDB01CFW9I|755|1
GACAAACGCTGGCGGCGTGCTTTAAACATGCAAGTCGAACGGAGGTTAGGTTTTCCACAGAGTGTTCTCGACTACGAAAGTAGAGTATAGTGCACTGTGTGGAGAACTTACCCTTAGTGGCGGACGGGTGAGTAACGCGTGAGTAATCTACCTCTATCTGGGGGACAACAGTTGGAAAACGACTGCTAATACCGCATAGGACCACAGGTACACATGTACTAGGGGTGAAAAGATTTATCGAATAGAGATGAGCTCGCGTAGCATTAGTTAGTTGGTGAGGTAACGGCCCACCAAGACCGTGATGCTTAGCCGATCTGAGAGGATGACTCGGCCACACTGGAACTGAGATACGGTCCAGACCTCCTACGGGA
>FQH3XDB01BVBTI|756|1
GACAAACGCTGGCGGCGTGCTTTAAACATGCAAGTCGAACGGAGGTTAGGTTTTCCACAGAGTGTTCTCGACTACGAAAGTAGAGTATAGTGCACTGTGTGGAGAACTTACCCTTAGTGGCGGACGGGTGAGTAACGCGTGAGTAATCTACCTCTATCTGGGGGGACAACAGTTGGAAACGCTGCTAATACCGCATAGGACCACAGGTTACACATGTACTAGGGGTGAAAGATTTATCCGAATAGAGATGAGCTCGCGTAGCATTAGTTAGTTGGTGAGGTAACGGCCCACCAAGACCGTGATGCTTAGCCGATCTGAGAGGATCGATCGGCCACACTGGAACTGAGATACGGTCCAGACTCCTACGGGAGGCAGCAGTTAGGAATA
>FQH3XDB01D9HIF|757|1
AGTTAACGCTGGCATCGTGCATAACACATGCAAGTCGAACGGGACTTACTTGAGTAGCAATACTTAGGTAAGTTTAGTGGCAGACGGGTGAGTAATACATAAGGAATTTACCTCCGAGTGGGGAATAACAGTCCGAAAGGATTGCTAATACCCCATAACATAATGTCGTGGCATCACTTCATTATCAAAGATTTATCGCTTGGAGAGAATCTTATGGTCTATCAGCTTGTTGGCAGTGTAACGGACTACCAAGGCGACGACGGATAGCCGGCCTGAAAGGGCGACCGGCCACAAGGGCACTGAGACACGGGCCC
>FQH3XDB01EKSZW|758|3
AGTTAACGCTGGCATCGTGCATAACACATGCAAGTCGAACGGGACTTGCTTTGGTAGCAATACCGAAGTAAGTTTAGTGGCAGACGGGTGAGTAATACATAAGGAATTCACCTCCGAGTGGGGAATAACAGTCCGAAAGGATTGCTAATACCCCATAACATAATCTAGTGGCATCACTTGATTATCAAAAGATTTATCGCTTGGAGAGAATCTTATGGTCTATCAGCTTGTTGGTAGGGTAACGGCCTACCAAGGCGACGACGGATAGCCGGCCTGAAAGGGCGACCGGCCACAAGGGCACTGAGACACGGGGCCCTACTCCTACGGGAGGCAGCAGTGGGGAATTTTGGACAATGGGCGAAAGCCTGATCCAGCAACGATGCGTGGAGGATGAAGGTTCTCGGGATTGTAAACTCCTTTTGCAGGGGACGAAAAATGACGGTACCCTGCGAATAAGCCACGGCTAACTACGTGCCAGCAGCC
>FQH3XDB01ERLPS|759|27
AGTTAACGCTGGCATCGTGCATAACACATGCAAGTCGAACGGGACTTTGCTTTGTAGCAATACAGAGTAAAGTTTAGTGGCAGACGGGGTGAGTAATGTATAAGAAACCTACCTCTGAGTGGGGAATAACAGTCCGAAAGGATTGCTAATACCCCATAACATCTTTAGGCGGCATCGTTTAGAGATCAAAGATTTATCGCTTGGAGACGGTCTTATATTCTATCAGCTAGTTGGTGGGGTAACGGCCTACCAAGGCTACGACGGATAGCCGGCCTGAAAGGGCGACCGGCCACAAGGGCACTGAGACACGGGCCCTACTCCTACCGGGAGGCCAGCAGTGGGGAATTTTGGACAATGGGCGAAAGCCTGATCCAGCAACGATGCGTGGAG
>FQH3XDB01ENJL3|760|2
GACGAACGCTGGCGGCGCGCCTAACACATGCAAGTCGAACGGAGTAAATTTTCTCACTGAGTTTTCTGAGACCTGACCAATGAGCGTCATTGATGCGGAGCATCAATTAGGCTCATTCGAGTAAGACTAACAGACTGAGAAGACCGAGTGAGGAAATTTACTTAGTGGCGAACGGGTGAGTAACGCGTGAGGAACCTGCCTCAAAGAGGGGGACAACAGTTGGAAACGACTGCTAATACCGCATAAGCCCACGGGTCGGCATCGGTCTGAGGGAAAAGGAGAGATCCGCTTTGAGATGGCCTCGCGTCCGATTAGCTAGTTGGTGAGGTAACGGCCCACCAAGGCGACGATCGGTAGCCGGACTGAGAGGTTGAACGGCCACATTGGGACTGAGACACGGCCCAG
>FQH3XDB01BZH6O|761|12
GACGAACGCTGGCGGCGCGCCTAACACATGCAAGTCGAACGGAGTAAATTTTCTCACTGAGTTTTCTGAGACCTGACCAATGAGCGTCATTGATGCGGAGCATCAATTAGGCTCATTCAAGTAAGACTAACAGACTGAGAAGACCGAGTGAGGAAATTTACTTAGTGGCGAACGGGTGAGTAACGCGTGAGGAACCTGTCTCAAAAGAGGGGGACAACAGTTGGAAACGACTGCTAATACCGCATAAGCCCACGGGTCGGCATCGGTCTGAGGGAAAAGGAGAAATCCGCTTTGAGATGGCCTCGCGTCCGATTAGCTAGTTGGTGAGGTAACGGCCCACCAAGGCGACGATCGGTAGCCGGACTGAGAGGTTGAACGGCCACATTGGGACTGAGACACGGCCCAGACTCCTACGGGAGGCAGCAGTGGGGAATATTGCAC
>FQH3XDB01CGL1Q|762|1
GACGAACGCTGGCGGCGCGCCTAACACATGCAAGTCGAACGGAGTAAATTTTCTCACTGAGTTTTCTGAGACCTGACCAATGAGCGTCATTGATGCGGAGCATCAATTAGGCTCATTCAAGTAAGACTAACAGACTGAGAAGACCGAGTGAGGAAATTTACTTAGTGGCGAACGGGTGAGTAACGCGTGAGGAACCTGTCTCAAAGAGGGGGACAACAGTTGGAAACGACTGCTAATACCGCATAAGCCCACGGGTCGGCATCGGTCTGGAGGGAAAAAGGAGAAAATCCGCTTTGAGATGGCCTCGCGTCCGATTAGCTAGTTGGTGAGGTAACGGCCACAAGGCGACGATCGGTAGCCGGACTGAGA
>FQH3XDB01BKO88|763|1
GACGAACGCTGGCGGCGCGCTTAACACATGCAAGTCGAACGGAGACACTCTGTTCACTGAGCGGTGGAGGCGAACGACAGAAACGTTAAGGTAAGAAGCGTTGCGCTTCTACTGTTGAGTGAATGGAGTGTCTTAGTGGCGGACGGGTGAGTAACACGTGAGGAACCTGCCTTGCAGTGGGGAATAACAGTCCGAAAGGACTGCTAATACCGCATAACGTATCGAGACCGCATGGTCATGATGCCAAAAGATTTTATCGCTGTAAGATGGCCTCGCGTCTGATTAGATAGTTGGTGAGGTAACGGCCCACCAAGTCGACGATCAGTAGCCGGACTGAGAGGTTGATCGGCCACATTGGGACTGAGACACGGCCCAGACTCCTACGGGAGGCAGCAGTGGGGATATTGCACAATGGCGCAAGCCTGATGCAGCGACGCCGC
>FQH3XDB01BAU6A|764|4
GATGAACGCTGGCGGCATGCCTAAGACATGCAAGTCGTACGAAGGGGCCCAATGAAAAGAATTGAAGTTTGAAGTGCTTGCACTAAGAATGGATTGAATTGGATTTGGATTTTCCCCTTAGTGGCAAACGGGTGAGTAACACGTGGGTTACCTGCCTCCAAGATGGGGATAACAGTTGGAAACGACTGATAATACCGAATGTGCTCTATGGAGTAAAGAAGCCTTTAAAGCTTCGCTTGGAGATGGGCCTGCGGCGTATTAGCTAGTTGGTGGGGTAATGGCCTACAGGCAACGATGCGTAGCCGAACTGAGAGGTTGATCGGCCAC
>FQH3XDB01ENS6H|765|1
GATGAACGCTGGCGGCATGCCTAAGACATGCAAGTCGTACGAAGGGGCCCAATGAAAAGAATTGAAGTTTGAAGTGCTTGCACTAAGAATGGATTGAATTGGATTTGGATTTTCCCCTTAGTGGCAAACGGGTGAGTAACACGTGGGTTACCCGCTCCAAGATAGGGATAACAGTTGGAAACGACTGATAATACCGAATGTGCTCTATGGAAGTAAAGAAGCCTTTAAAGCTTCGCTTGGAGATGGGCCTGCGGCGTATTAGCTAGTTGGTGGGGTAATGGCCTACCAAGGCAACGATGCGTAGCCGAACTGAGAGGTTGATCGGCCACACTGGGACTGAGACACGGCCCA
>FQH3XDB01E4KXR|766|1
GATGAACGCTGGCGGCATGCCTAAGACATGCAAGTCGTACGAAGGGGCCCAATGAAAGGATTGAAGTTTGGAGTGCTTGCACAAAGAATGAATTTTCTTGGGATTTGGATTTTCCCCCTTAGTGGCAAACGGGTGAGTAACACGTGGGTTACCTACCTCTAAGATGGGGATAACAGTTGGAAACGACTGATAATACCGAATGTGCTCTACGGAGTAAAGAAGCCCTTAAAGCTTCGCTTAGGAGATGGGCCTGCGGCGTATTAGCTAGTTGGTGGGGTAATGGCCTACCAAGGCGACGATGCGTA
>FQH3XDB01ETDWA|767|1
GATGAACGCTGGCGGCATGCCTAAGACATGCAAGTCGAACGGGATGGCCCATTGATTACAACTGAAAACTGGAGTGCTTGCACAAAAGCTGGATGGTGTATGATTTGGATTTCCATCCAGTGGCAAAACGGGTGAGTAACACGTGGGTTACCTGGCCTCTAAGTTGGGGATAACAGTTGGAACGATTGCTAATACCGAATGTGCTCTACGGAGTAAAGATGCCTTTAAAGCATCGCTTAGAGATGGGCCTGCGGCGTATTAGTTAGTTGGTGGGGTAATGGCCTACCAAGACGACGATGCGTAGCCGAACTGA
>FQH3XDB01A8BMK|768|11
GATTAACGCCGGCGGCATGCCTAATACATGCAAGTCAAACGGGATTATAGCTTGCTATAATCTAGTGGCGAACGGGTGAGTAACACGTGGGCAACCTGTCCTAAAGTCGAGGATAACAGTTGGAAACGACTGCTAATACTGGATAGTATATATTATCGCATGGTGATATATTTAAAGATGCGTTTGCATCACTTTTAGGAGGGGCCTGCGGTGCATTAGCTAGTTGGTGAGGTAACGGCTCACCAAGGCGACGATGCATAGCTGCGCTGAGAGGCGAAACAGCCACATTGGGACTGAGACACGGCCCAAAACTCCTACGGGAGGCAGCAGTAGGGAATTTTCGGCAATGGAGGAAACTCTGACCGAGCAATGCCGCGTGAACGATGAAGGTCTTCGGATTGTAAAGTTCTGTTGTTAAGGAGAACTGTACGAATAGGAAATGATTCGTATTTGACGGTACTTAACCAG
>FQH3XDB01EL7VH|769|7
GATTAACGCTGGCGGCATGCCTAATACATGCAAGTTGAACGGGAAGTAGCAATACTTCCAGTAGCGAACGGGTGAGTAATACATAAGTAACCTGCCTCCTTGATGGGGATAACTGATCGAAAGATTAGCTAATACCGAATGAACTGATTGATATGCATATATTAATGAGGAAAGATAAGATCGCAAGGAGATGGGCTTATGGCGCATTAGCTAGTTGGTGGGTAAGGCCTACCAAGGCAACGATGCGTAGCCGACCTGAGAGGGTGAACGGCCACACTGGAACTGAGACACGGTCCAGACTCCTACGGAGGCAGCAGTAGGGAATTTTCGGCAATGGGCGAAAGCCTGACCGAGCAATGCCGCGTGAACGAAGAAGGTCTTCGGATTGTAAAGTTCTTTGCAAGGGAAGAAAAGGCTGTGAAGAGCAGCGAATTGACGGTACTTGAGAATAAGCCACGGCTAACTACGTGCCAGCAGCC
>FQH3XDB01AW0ER|770|4
GATTAACGCTGGCGGCATGCCTAATACATGCAAGTTGAACGGGAAGTAGCAATACTTCTAGTAGCGAACGGGTGAGTAATACATAAGTAACCTGCCTTCTTGATGGGGATAACTGATCGAAAGATTAGCTAATACCGAATGAACTGATTGATATGCATATATTAATGAGGAAAGATGAGATCGCAAGGAGATGGGCTTATGGCGCATTAGCTAGTTGGTGGGTAAGGGCCTACCAAGGCGACGATGCGTAGCCGACCTGAGAGGGTGAACGGCCACACTGGAACTGAGACACGGTCCAGACTCCTACGGGAGGCAGCAGTAGGGAATTTTCGGCAATGGGCGAAAGCCTGACCGAGCAATGCCGCGTGAATGATGAAGGTCTTCGGATTGTAAAGTCTTTTGCTAAGGGAGAAAAGGCTGTGAAGAGCAGCGAATTGACGGTACCTTGAGAATAAGCCACGGCTAACTACGTGCCAGC
>FQH3XDB01E4UQQ|771|1
GATTAACGCTGGCGGCATGCCTAATACATGCAAGTTGAACGGGAAGTAGCAATACTTCTAGTAGCGAACGGGTGAGTAATACATGAGTAACCTGCCTTTTTGATGGGGATAACTGATCGAAAGATTAGCTAATACCGAATAAAGAGATTGAGATGCATATCTTAATAAGGAAAGCTTAGAAGCGCAAGAAGATGGGCTCATGGCGCATTAGCTAGTTGGTGGGGTAAGGGCCTACCAAGGCAACGATGCGTAGCCGACCTGAGAGGGTGAACGGCCACACTGGAACTGAGACACGGTCCAGACTCCTACGGGAGGCAGCAGTAGGGAATTTTCGGCAATGGGCGAAAGCCTGACCGAGCAT
>FQH3XDB01EYAL4|772|1
GATTAACGCTGGCGGCATGCCTAATACATGCAAGTTGAACGGGAAGTAGCAATACTTCTAGTAGCGAACGGGTGAGTAATACATGAGTAACCTGCCTCTTTGATGGGGATAACTGATCGAAAGATTAGCTAATACCGAATAAACTGATTGATATGCATATGTTAATGAGGAAAGATAAGATCGCAAAGAGATGGGCTCATGGCGCATTAGCTAGTTGGTGGGTAAGGGCCTACCAAGGCAACGATGCGTAGCCGACCTGAGAGGGTGAACGGCCACACTGGAACTGAGACACGGTCCAGACTCCTACGGGAGGCAGCAGTAGGGAATTTTCGGCAATGGGCGGAAGCCTGACCGAGCAATGCCGCGTGAACGATGAAGGTCTTCGGATTGTAAAGTTCTTTGCAAAGGAAGAAAAGCTGTGAAGAGCAGCAT
>FQH3XDB01B7ALS|773|7
GATTAACGCTGGCGGCATGCCTAATACATGCAAGTCAAACGGGGTTATAGCTTGCTATAATCAAGTGGCGAACGGGTGAGTAACACGTAGGCAACCTGTCCTAAAGACGAGGATAACAGTTGGAAACGACTGCTAATACTGGATAGTATATAGAATTGCATGATTTTATATTTAAAGATGCGTTTGCATCACTTTAGGAGGGCCTGCGGCGCATTAGCTAGTTGGTGAGGTAACGGCTCACCAAGGCGACGATGCGTAGCTGCGCTGAGAGGCGAAACAGCCACATTGGGACTGAGACACGGCCCAAAACTCCTACGGGAGGCAGCAGTAGGGAATTTTCGGCAATGGGGGAAACCCTGACCGAGCAATGCCGCGTGAATGATGAAGGTCTTCGGATTGTAAAAGTTCTATTGTTAAGGAAGAATTGTACAAATAGGAAAATGATTTGTATTTGACGGTACTTAACTAGAAAAGCCCCGGCTAAACTATGTGCCAGCA
>FQH3XDB01ASL8O|774|48
GATTAACGCTGGCGGCATGCCTAATACATGCAAGTCAAACGGGATTATAGCTTGCTATAATCTAGTGGCGAACGGGTGAGTAACACGTGGGCAACCTGTCCTAAAGTCGAGGATAACAGTTGGAAACGGCTGCTAATACTGGATAGTATATATTATCGCATGATGATATATTTAAAGATGCGCTTGCATCACTTTAAGAGGGGCCTGCGGTGCATTAGCTAGTTGGTGAGGTAACGGCCCACCAAGGTGACGATGCATAGCTGCGCTGAGAGGCGAAAACAGCCACATTGGGGACTGAGACACGGCCCCAAAACTCCTACGGGGAGGCAGCAGTAGGGAATTTTCGGCAATGGAGGAAACTCTGACCGAGCAATGCCGCGTGAACGATGAAGGTCTTCGGATTGTAAAGTTCTGTTGTTAAGGAAGAACTATACGAATAGGAAATGATTCGTATTTGACGGTACTTAACTAGAAA
>FQH3XDB01B9TA0|775|1
GATTAACGCTGGCGGCATGCCTAATACATGCAAGTCAAACGGGATTATAGCTTGCTATAATCTAGTGGCGAACGGGTGAGTAACACGTGGGCAACCTGTCCTAAAGACGAGGATAACAGTTGGAAACGACTGCTAATACTGGATAGTATATATAACCGCATGGAGATATATTTAAAGATGCGTTTGCATCACTTTAGGAGGGGCCCGCGGTGCATTAGCTAGTTGGTGAGGTAACGGCTCACCAAGGCAACGATGGCAGTAGCTGCGCTGAGAGGCGAAACAGCCACATTGGGACTGAGACACGGCCCAAAACTCCTACGGGAGGCAGCAGTAGGGAATTTTCGGCAATGGAGGAAACTCTGACCGAGCAATGCCGCGTGAATGATGAAGGTCTTCGGATTGTAAAGTTCTGTTGTTAAGGAAGAATGTATGAATAGG
>FQH3XDB01AZGOE|776|1
GATTAACGCTGGCGGCATGCCTAATACATGCAAGTTGAACGGGAAGTAGCAATACTTCCAGTAGCGAACGGGTGAGTAATACATAAGTAACCTGCCTCTTTGATGGGGATAACTGATCGAAAGATTAGCTAATACCGAATGAACTGATTGATATGCATATATTAATGAGGAAAGATAAGATCGCAAGGAGATGGGCTTATGGCGCATTAGCTAGTTGGTGGGTAAGGGCCTACCAAGGCAACGATGCGTAGCCGACCTGAGAGGGTGAACGGCCACACTGGAACTGAGACACGGTCCAGACTCCTACGGGAGGCAGCAGTAGGGAATTTTCGGCAATGGGCGAAAGCCTGACCGAGCAATGCCGCGTGAACGAG
>FQH3XDB01CYW69|777|1
GATTAACGCTGGCGGCATGCCTAATACATGCAAGTTGAACGAGAAGTAGCAATACTTCTAGTAGCGAACGGGTGAGTAATACATAAGTAACCTGCCTTCTTGATGGGGATAACTGATCGAAAGATTGGCTAATACCGAATGAACTGATTGATATGCATATATTAATGAGGAAAAGATGAGATCGCAAGGAGATGGGCTTATGGCGCATTAGCTAGTTGGAGGGGTAATGGCCCGCCAAGGCGACGATGCGTAGCCGACCTGAGAGGGTGAACGGCCACACTGGAACTGAGACACGGTCCAGACTCCTACGGGAGGCAGCAGTAGGGAATTTTCGGCAATGGGCGGAAGCCTGACCGAGCAATGCCGCGTGAATGATGAAGGTCTTCGGATTGTAAAAGTTCTTTGCAAGGG
>FQH3XDB01DJLLK|778|7
GATTAACGCTGGCGGCATGCCTAATACATGCAAGTTGAACGAGAAGTAGCAATACTTCTAGTAGCGAACGGGTGAGTAATACATAAGTAACCTGCCTTCTTGATGGGGATAACTGATCGAAAGATTGGCTAATACCGAATGAACTGATTGATATGCATATATTAATGAGGAAAGATGAGATCGCAAGGAGATGGGCTTATGGGCGCATTAGCTAGTTGGAGGGTAATGGCCCGCCAAGGCGACGATGCGTAGCCGACCTGAGAGGTGAACGGCCACACTGGAACTGAGACACGGTCCAGACTCC
>FQH3XDB01BAAFH|779|1
GATTAACGCTGGCGGCATGCCTAATACATGCAAGTTGAACGAGAAGTAGCAATACTTCTAGTAGCGAACGGGTGAGTAATACATAAGTAACCTGCCTTCTTGATGGGGATAACTGATCGAAAGATTGGCTAATACCGAATGAACTGATTGATATGCATATAGTTAATGACGGAAAGATGAGATCGCAAGGAGATGAGCTTATGGCGCATTAGCTAGTTGGAGGGGTAATGGCCCGCCAAGGCGACGATGCGTAGCCGACCTGAGAGGGTGAACGGCCACACTGGAACTGAGACACGGTCCAG
>FQH3XDB01CMMRV|780|1
GATTAACGCTGGCGGCATGCCTAATACATGCAAGTTGAACGAGAAGTAGCAATACTTCTAGTAGCGAACGGGTGAGTAATACATAAGTAACCTGCCTTCTTGATGGGGATAACTGATCGAAAGATTGGCTAATACCGAATGAACTGATTGATATGCATATATTAATGAAGGAAAGATGAGATCGCAAGGAGATGGGCTTATGGCGCATTAGCTAGTTGGAGGGTAATGGCCCGCCAAGGCGACGATGCGTAGCCGACCTGAGAGGTGAACGGCCACACTGGAACTGAGACACGGTCCAGACTCCGTA
>FQH3XDB01C8OQI|781|1
GATTAACGCTGGCGGCATGCCTAATACATGCAAGTTGAACGAGAAGTAGCAATACTTCTAGTAGCGAACGGGTGAGTAATACATAAGTAACCTGCCTTCTTGATGGGGATAACTGATCGAAAGATTGGCTAATACCGAATGAACTGATTGATATGCATATATTAATGAGGAAAGATGAGATCGCAAGGAGATTGGGGCCTTAATGGGCGCATTAGCTAGTTGGAGGGGTAATGGCCCGCCAAGGCGACGATGCGTAGCCGACCTGAGAGGGTGAACGGCCACACTGGAACTGAGACACGGTCCAGACTCCTACGGGAGGCAGCAGTAGGGAATTTTCGGCAATGGCGAAGCCTGACCGAGCAATGCCGCGTGAATGATGAAGGTCTTCGGATTGTAAA
>FQH3XDB01D5N7B|782|1
GATTAACGCTGGCGGCATGCCTAATACATGCAAGTTGAACGGGAAGTAGCAATACTTCTAGTAGCGAACGGGTGAGTAATACATAAGTAACCTGCCTCTTTGATGGGGATAACTGATCGAAAGATTAGCTAATACCGAATGAACTGATTGATATGCATATATTAATGAGGAAAGATAAGATCGCAAAGAGATGGGCTTATGGCGCATTAGCTAGTTGTGGGTAAGGGCCTACCAAGGCAACGATGCGTAGCCGACCTGAGAGGGTGAACGGCACACTGGAACTGAGACACGGTCCAGACTCCTACGGAGGCAGCAGTAGGGAATTTTCGGCAATGGGCGAAAAGCCTGACCGAGCAATGCCGCGTGAACGATGAAGGTCTTCGGATTGTAAAAGTTCTTTTGCAAGGG
>FQH3XDB01DUPJZ|783|8
GATTAACGCTGGCGGCATGCCTAATACATGCAAGTTGAACGGGAAGTAGCAATACTTCTAGTAGCGAACGGGTGAGTAATACATGAGTAACCTGCCTCTTTGATGGGGATAACTGATCGAAAGATTAGCTAATACCGAATAAACTGATTGATATGCATATGTTAATGAGGAAAGATAAGATCGCAAAGAGATGGGCTCATGGCGCATTAGCTAGTTGGTGGGTAAGGGCCTACCAAGGCAACGATGCGTAGCCGACCTGAGAGGGTGAACGGCCACACTGGAACTGAGACACGGTCCAGACTCCTACGGGAGGCAGCAGTAGGAATTTTCGGCAAGTGGGC
>FQH3XDB01AQK3R|784|1
GATTAACGCTGGCGGCATTGCCTAATACATGCAAGTCAAACGGGATTATAGCTTGCTATAAATCTAGTGGCGAACGGGTGAGTAACACGTGGGCAACCTGTCCTAAAGTCGAGGATAACAGTTGGAAACGACTGCTAATACTGGATAGTATATATTATCGCATGGTGATATATTTAAAGATGCGTTTGCATCACTTTAGGAGGGCCTGCGGTGCATTAGCTAGTTGGTGAGGTAACGGCTCACCAAGGCGACGATGCATAGCTGCGCTGAGAGGCGAAACAGCCACATTGGGACTGAGACAC
>FQH3XDB01C4GD1|785|216
ATTGAACGCTGGCGGCATGCTTTACACATGCAAGTCGAACGGCAGCACAGGGAGCTTGCTCCCGGGTGGCGAGTGGCGCACGGGTGAGTAATACATCGGAACGTGTCCTGTTGTGGGGGATAACTGCTCGAAAGGTGGCTAATACCGCATGAGACCTGAGGGTGAAAGCGGGGGATCGCAAGACCTCGCGCAACTGGAGCGGCCGATGCCCGATTAGCTAGTTGGTGAGGTAAAGGCTCACCAAGGCGACGATCGGTAGCTGGTCTGAGAGGACGACCAGCCACACTGGGACTGAGACACGGCCCAGACTCC
>FQH3XDB01CO3VD|786|8
GATTAACGCTGGCGGCATGCCTAAGACATGCAAGTCGAACGGAGCGCCCCATTGACACTGGAATGAAGTTGAAGAGCTTGCTCAGATATGGAATGAAAGTTGATTTGGATTCTGCGCTCAGTGGCGCACGGGTGAGTAACACGTGGGTTATCTGCCTTTAAGTTGGGGATAACAGTTAGAAATGACTGCTAATACCGAATGTGCTAGTAATAGTAAAGGAGCCTTTAAAGCTTCGCTTAAAGATGAGCCTGCGGCGTATTAGCTAGTTGGTGGGGTAATGGCCTACCGAAGGCGACGATGCGTAGCCGGATGAGAGGTTGATCGGCCACACT
>FQH3XDB01CNKI7|787|2
GATTAACGCTGGCGGCATGCCTAAGACATGCAAGTCGAACGGAGCGCCCCATTGACACTGGAATGAAGTTGAAGAGCTTGCTCAGATATGGAATGAAGGTTGATTTGGATTCTGCGCTCAGTGGCGCACGGGTGAGTAACACGTGGGTTATCTGCCTTTAAGTTGGGGATAACAGTTAGAAATGACTGCTAATACCGAATGTGCTAGTAATAGTAAAGGAGCCCTTAAAAGCTTCGCTTAAAGATGAGCCTGCGGCGTATTAGCTTGTTGGTGGGGTAATGGCCTACCAAGGCGACGATGCGTAGCCGGACTGAGAGGTTGATCGGCCACACTGGGACTGAGACACGGCCCAGACCTCCGTACGGGAGACAGCAGTT
>FQH3XDB01DHVTM|788|2
GATTAACGCTGGCGGCATGCCTAAGACATGCAAGTCGAACGGAGCGCCCCATTGAAACTGGAATGAAGTTGAAGAGCTTGCTCAGATATGGAATGAAAGTCGATTTGGATTCTGCGCTCAGTGGCGCACGGGTGAGTAACACGTGGGTTATCTGCCTTTAAGCTGGGGATAACAGTTAGAAATGACTGCTAATACCGAATGTGCTAGTAATAGTAAAGAAGCCCTTAAAGCTTCACTTAAAGATGAGCCTGCGGCGTATTAGCTTGTTGGTGGGGTAATGGCCTACCAAGGCAACGATGCGTAGCCGGACTGAGAGGTTGATCGGCCACACTGGGACTGAGACACGGCCCAGACTCCTACGGGAGACAGCAGTTAGGAATATTCGGCAATGGGCGAAAGCCTGACCGAGCAATGCCGCGTGTGAGATGAAGGTCCTTTGGATTGTAAACTCACTTTTATTTGGGAAGAACTGTAAGTATAGGAAAG
>FQH3XDB01EWXCO|789|1
GATTAACGCTGGCGGCATGCCTAAGACATGCAAGTCGAACGGAGCGCCCCATTGACACTGGAATGAAGTTGAAGAGCTTGCTCAGATATGGAATGATAGTTGATTTGGATTTTGCGCTCAGTGGCGCACGGGTGAGTAACACGTGGGTTATCTGCCTTTAAGTTGGGGATAACAGTTAGAAATGACTGCTAATACCGAATGTGCTAGTAATAGTAAAGGAGCCCTTAAAGCTTCGCTTAAAGATGAGCCTGCGGCGTATTAGCTTGTTGGTGGGGTAATGGCCTACCAAGGCGACGATGCGTAGCCGGACTGAGAGGTTGATCGGCCACACTGGGGACTGAGACACGGCCCAGACTCCTACGGGAGACAGCAGTTAGGAATATTCGGCAATGGGCGAAAGCCTGACCGAGCAATGCCGCGTGTGAGATGAAGGTCTTGGATTGTAAATCA
>FQH3XDB01B2EQR|790|2
GATAAACGCTGGCGGCATGCCTAAGACATGCAAGTCGAACGAAGTGCCCCATTGACGTTGGAATGAAATTGATGAGCTTGCTCTGATATGGAATGAAAACTGATTTGGATTCTGCACTTAGTGGCGAACGGGTGAGTAACACGTGGGTTATCTGCCTTCAAAAGTTGGGGATAACAGTTAGAAATGACTGCTAATACCGAATGTGCTAGTAATAGTAAAGGAGCCCTTAAAGCTTCGCTTGAAGATGAGCCTGCGGTGTATTAGCTAGTTGGTGGGTAATGGCCTACCAAGGCGACGATGCATAGCCGGATGA
>FQH3XDB01DGIK6|791|6
GATAAACGCTGGCGGCATGCCTAAGACATGCAAGTCGAACGAAGTGCCCCATTGACGTTGGAATGAAATTGATGAGCTTGCTCTGATATGGAATGAAAACTGATTTGGATTCTGCACTTAGTGGCGAACGGGTGAGTAACACGTGGGTTATCTGCCTTCAAGTTGGGGATAACAGTTAGAAATGACTGCTAATACCGAATGTGCTAGTAATAGTAAAGGAGCCCTTAAAGCTTCGCTTGAAGATGAGCCTGCGGTGTATTAGCTAGTTGGTGGGTAATGGCCTACCAAGGCGACGATGCATAGCCGGACTGAGAGGTTGATCGGCCACACTGGGACTGAGAC
>FQH3XDB01DVOKO|792|1
GATAAACGCTGGCGGCATGCCTAAGACATGCAAGTCGAACGAAGTGCCCCATTGACGTTGGAATGAAATTGATGAGCTTGCTCTGATATGGAATGAAAACTGATTTGGATTCTGCACTTAGTGGCGAACGGGTGAGTAACACGTGGGTTATCTGCCTTCAAGTTGGGGATAACAGTTAGAAATGACTGCTAATACCGAATGTGCTAGTAATAGTAAAGGAGCCCTTAAAGCTTCGCTTGAAGATGAGCCTGCGGTGTATTAGCTAGTTGGTGGGGTAATGGCCTACCAAGGCGACGATGCATAGCCGGACTGAGAGGTTGATCGGCCACACTGGGACTGAGACACGGCCCAGACTCCTACGGGGAGACAGCAGTTAGGAATATTCGGCAATGGGCGAAAGCCTGACCGAGCAATGCCGCGTG
>FQH3XDB01DPJQW|793|2
GATTAACGCTGGCGGCATGCCTAAGACATGCAAGTCGAACGGGGTATACCATTGAGATTGGAATGAAGTTGAAGAGCTTGCTCAGATATGGAATGAAAGTGGATTTGGATTCTATACCTAGTGGCGAACGGGTGAGTAACACGTGGGTTACCTGCCTCTAAGTTGGGGATAACGGTTAGAAATGATCGCTAATACCGAATGTGCTAGTAATAGTAAAAGGTGCTTTCAAGCATCGCTTAGAGATGGGCCTGCGGCGTATTAGCTTGTTGGTGAGGTAACGGCTCACCAAGGCACGATGCGTAGCCGGACTGAGAGTTAAAC
>FQH3XDB01B4999|794|2
GATGAACGCTGGCGGCATGCCTAAGACATGCAAGTCGAACGAGGTGGCCCATTGATTTTTATTGAAAGCGGAGTGCTTGCACAAAGTTGGATTTAATTTGATTTGGATTCTCCACCTAGTGGCGAAAAGGGTGAGTAACACGTAGGAATCTACCTTTAAGACTGGGATAACAATTGGAAACGATTGCTAATACCGGATGACATATTAAAATGATACGTTATTAATATTAAAAAGGAGCCTTTAAAAGCTTCACTTAAAGATGAGCCTGCGGCGTATTAGCTAGTTGGTAAGGTAACGGCTTAACCAAGGCGACGATGCGTAGCCGACCTGAGAGGGTGATCGGCCACAGTTGGGACTGAGA
>FQH3XDB01CUYXN|795|1
GATGAACGCTGGCGGCATGCCTAAGACATGCAAGTCGTACGAAGGGGCCCAATGAAAAGAATTGAAATTTGGAGTGCTTGCACAAAAAATGGACTTTCTTGGATTTGGATTTTCCCCTTAGTGGCAAACGGGTGAGTAACACGTGGGTTACCTGCCTCCAAGATGGGGATAACAGTTGGAAACGACTGATAATACCGAATATGCTCTACGGAGTAAAGAAGCCTTTAAAGCTTCGCTTGGAGATGGGCCTGCGGCGTATTAGCTAGTTGGTGGGTAATGGCCTACCAAGGCAACGATGCGTAGCCGAACTGAGAGGTTGATCGGCCACACTGGGACTGAGACACGGCCCA
>FQH3XDB01EP936|796|1
GATGAACGCTGGCGGCATGCCTAAGACATGCAAGTCGAACGGGATGGCCCATTGATTATAATTGAAAATTGGAGTGCTTGCACAAAAATTGGAAGTTATATGATTTGGATTTTCCATCCAGTGGCAAACGGGTGAGTAACACGTGGGTTACCTACCTCTAAGTTGGGGATAACAATTGGAAACGATTGCTAATACCGAATGTGCTCTACGGAGTAAAGATGCCCTTAAAGCATCACTTAGAGATGGGCCTGCGGCGTATTAGCTAGTTGGTGGGTAACGCTACCAAGGCGACGATGCGTAGCCGAACTGAGAGGTCAA
>FQH3XDB01EUVR7|797|1
GATTAACGCTGGCGGCATGCCTAAGACATGCAAGTCGAACGGAGCGCCCCATTGAAACTGGAATGAAGTTGAAGAGCTTGCTCAGATATGGAATGAAAGTCGATTTGGATTCTGCGCTCAGTGGCGCACGGGTGAGTAACACGTGGGTTATCTGCCTTTAAGCTGGGGATAACAGTTAGAAATGACTGCTAATACCGAATGTGCTAGTAATAGTAAAGAAGCCCTTAAAGCTTCACTTAAAGATGAGCCTGCGGCGTATTAGCTAGTTGGTGAGATAACGGCCCACCAAGGCGACGATGCATAGCCGAACTGAGAGGTTAATCGGCCACATTGGGACTGAGACACGGCCAACTCCTACGGGAGACAGCAGTTAGGAATATTCGTC
>FQH3XDB01E3ZMJ|798|7
GATTAACGCTGGCGGCATGCCTAAGACATGCAAGTCGAACGGAGCGCCCCATTGAAACTGGAATGAAGTTGAAGAGCTTGCTCAGATATGGAATGAAAGTCGATTTGGATTCTGCGCTCAGTGGCGCACGGGTGAGTAACACGTGGGTTATCTGCCTTTAAGCTGGGGATAACAGTTAGAAATGACTGCTAATACCGAATGTGCTAGTAATAGTAAAGAAGCCCTTAAAGCTTCACTTAAAGATGAGCCTGCGGCGTATTAGCTTGTTGGTGGGGTAATGGCCTACCAAGGGCAACGATGCGTAGCCGGGACTGAGAGGTTGATCGGCCACACTGGGACTGAGACCACGGCCCCAGACTCCTACGGGA
>FQH3XDB01ER9OF|799|2
GATTAACGCTGGCGGCATGCCTAAGACATGCAAGTCGAACGGAGCGCCCCATTGAAACTGGAATGAAGTTGAAGAGCTTGCTCAGATATGGAATGAAAGTCGATTTGGATTCTGCGCTCAGTGGCGCACGGGTGAGTAACACGTGGGTTATCTGCCTTTAAGCTGGGGATAACAGTTAGAAATGACTGCTAATACCGAATGTGCTAGTAATAGTAAAGAAGCCCTTAAAAGCTTCACTTAAAGATGAGCCTGCGGCGTATTAGGCTTTGTTGGTGGGGTAATGGCCTACCAAGGCAACGATGCGTAGCCGGACTGAGAGGTTGATCGGCCACACTGGGACTGAGACACGGCCCAGACTCCTACGGGAGACAGCAGTTAGG
>FQH3XDB01ENPB0|800|1
GATTAACGCTGGCGGCATGCCTAAGACATGCAAGTCGAACGGAGCGCCCCATTGAAACTGGAATGAAGTTGAAGAGCTTGCTCAGATATGGAATGAAAGTCGATTTGGATTCTGCGCTCAGTGGCGCACGGGTGAGTAACACGTGGGTTATCTGCCTTTAAGCTGGGGATAACAGTTAGAAATGACTGCTAATACCGAATGTGCTAGTAATAGTAAAGAAGCCCTTAAAGCTTCACTTAAAGATGAGCCTGCGGCGTATTAGCTTGTTGGTGGGGTAATCGGCCTACCAAGGCAACGATTGCGTAGCCGGACT
>FQH3XDB01EF7R7|801|10
GATTAACGCTTGCGGCATGCCTAAGACATGCAAGTCGAACGAGAAGGCCTATGGATTTAATATTGAAGTCGGAGTGCTTGCACAATGATGAATTTATTATTGAAGTAGATTACCTTCTAGTGGCAAACGGGTGAGTAACACGTGGGTTACCTGCCCTCAAGTTGGGGATATCATCTGGAAACGGATAGTAATACCGAATGTGATCTACGGATTAAAGGAGCCTTTAAAGCTTCGCTTGAGGATGGGCCTGCGGTGCATTAGCTAGTTGGTGAGATAAAAGCCCACAAGGCGACGATGCATAGCCGAACTGAGAGGTTGATCGGCCACACTGGGACTGAGACACGGCCCAGACTCCTACGGGAGACAGCAGTTAGGAATATTCGGTCAAGTGGGGAAACCCTGAACGAGCAATGCCGCGTGAACGATGAAGGCCCTCCGGGTTGTAAAGTTC
>FQH3XDB01EWPQZ|802|5
GATGAACGCTGGCGGCATGCCTAAGACATGCAAGTCGAACGGAGCGGCCCAATGATGTTTATTGAAGCTATGAGAGCTTGCTCGATTAGTGGATTTAAATTGATTTGGATTCCCGCTTAGTGGCGAAAGGGTGAGTAACACGTAGGAATCTGCCTCAGAGACTGGGACAACAGTTGGAAACGACTGCTAATACCGGATGATATATTAAACGATACGTTGTTTAATATTAAAAGGAGCCTTTAAAGCTTCACTTTGAGATGAGCCTGCGGCGTATTAGCTAGTTGGTGAGGTAATGGCTCACCAAGGCAACGATGCGTAGCCGAACTGAGAGGTTGATCGGCCACATTGGGACTGAGACACGGCCCAAACTCCTATGGGAGACAGCAGTTAGGAATATTCGTCAATGGGGAAACCCTGAACGAGCAATGCCGCGTGAGTGATGAAGGTCTTGATGTAAACT
>FQH3XDB01BIYWV|803|4
GATTAACGCTTGCGGCATGCCTAAGACATGCAAGTCGAACGAGAAGGCCTACGGATTTAATATTGAAGTTGGAGTGCTTGCACAATGATGGATTTATTATTGAAGTAGATTACCTTCTAGTGGCAAACGGGTGAGTAACACGTGGGTTACCTGCCCTCAAGTTGGGGATATCATCTGGAAACGGATAGTAATACCGAATGTGATCTACGGATTAAAGGAGCCTTTAAAAGCTTCGCTTGAGGATGGGCCTGCGGTGCATTAGCTAGTTGGTGAGATAAAAGCCCACCAAGGCGACGATGCATAGCCGAACTGAGAGGTTGATCGGCCACACTGGGACTGAGACACGGCCCAGACTCCTACGGGAGACAGCAGTTAGGAATATT
>FQH3XDB01CCH1W|804|5
GATGAACGCTGGCGGCATGCCTAAGACATGCAAGTCGAACGGGATGGCCCAATGATAATGATTGAAAACTTGAGTGCTTGCACAAGAGTCGGATATCATTTGATTTGGATTTTCCATCCAGTGGCAAACGGGTGAGTAACACGTGGGTTACCTACCTCTAAGTTGGGGATAACAGTTGGAAACGATTGCTAATACCGAATGTGCTCTACGGAGTAAAGATGCCTTTAAAGCATCGCTTAGAGATGGGGCCTGCGGCGTATTAGCTAGTTGGTGGGGTAATGGCCTACCAAGGCGACGATGCGTAGCCGAACTGAGAGGTTAATCGGCCACACTGGGACTGAGACACGGCCCAGACTCCTACGGGAGACAGCAGTTAGGAATATTCGTCAAGTGGGGGGAACCCTGAACGAGCAATGCCGCGTGAGTGAT
>FQH3XDB01EN6LC|805|2
GATGAACGCTGGCGGCATGCCTAAGACATGCAAGTCGAACGGGATGGCCCATTGATTATAATTGAAAATTGGAGTGCTTGCACAAAGATTGGAAGTTATATGATTTGGATTTTCCATCCAGTGGCAAACGGGTGAGTAACACGTGGGTTACCTACCTCTAAGTTGGGGATAACAATTGGAAACGATTGCTAATACCGAATGTGCTCTACGGAGTAAAGATGCCCTTAAAGCATCGCTTAGAGATGGGCCTGCGGCGTATTAGCTAGTTGGTGGGTAACGGCCTACCAAGGCGACGATGCGTAGCCGAACTGAGAGGTCAATCGGCCACACTGGGGACTGAGACACGGCCCAGACTCCTACGGGGAGACAGCAGTTAGGAATATTCGTCAATGGGGAAACCCTGAACGAGCAATGCCGCGTGAGTGATGAA
>FQH3XDB01C9J5T|806|1
GATGAACGCTGGCGGCATGCCTAAGACATGCAAGTCGAACGAGGTGGCCCATTGATTTTTATTGAAAGTGGAGTGCTTGCACAAAGCCGGATTTAATTTGATTTGGATTCTCCACCTAGTGGCGAAAGGGTGAGTAACACGTAGGAATCTACCTTTGAGACTGGGATAACAATTGGAAACGATTGCTAATACCGGATGATATATTAAATGATACGTTATTAATATTAAAAAGGAGCCTTTAAAAGCTTCACTTAAAGATGAGCCTGCGGCGTATTAGCTAGTTGGTGAGGTAACTGCTCACCAAGGCGACGATGCGTAGCCGACCTGAGAGGGTGATCGGCCACATTGGGGACTGAGACACGGCCCAAAACTCCTATGGGAGACAGCAGTTAGGAATATTCGACAATGGAGGAAA
>FQH3XDB01B0QB6|807|1
GATGAACGCTGGCGGCATGCCTAAGACATGCAAGTCGAACGAAGTGGCCCATGATTTTTATTGAAAGTCGGAGTGCTTGCACAAAGACCGGAAATAATTTGATTGGATTCTCCACTTAGTGGCGAAAGGGTGAGTAACACGTAGGAATCTACCTTTAAGACTGGGATAACAGTTGGAAACGACTGCTAATACCGGATATGATATTAAATGATACGTTATTAATATTGAAAAGGAGCCTTTAAAAGCTTCACTTAAAGATGAGCCTGCGGCGTATTAGCTAGTTGGTAGGTAACGGCCTACCAAGGCAACGATGCGTAGCCGACCTGAGAGGGTGATCGGCCACATTGGGACTGAGACACGGCCCAAA
>FQH3XDB01AL0RB|808|1
GATGAACGCTGGCGGCATGCCTAAGACATGCAAGTCGAACGAGAGGGCCCATTGATTAAGATTGAAAGATTGAGTGCTTGCACGATTTCCGGATTTCTTATGATTTGGAATTTCCCTCTAGTGGCAAACGGGTGAGTAATACGTGGGTTACCTACCTCTAAGCTGGGGATAACAGTTGGAAACGACTGATAATACCGAATGTGCTCTACGGAGTAAAGAAGCCCTTAAAGCTTCACTTAGAGATGGGCCTGCGGCGTATTAGCTAGTTGGTGGGTAACGGCCTACCAAGGCGACGATGCGTAGCCGAACTGAGAGGTTAATCGGCCACACTGGGACTGAGACACGGCCCAGACCTCCTACGGGAGACAGCAGTTAGGAATATTCG
>FQH3XDB01A0PG6|809|1
GATGAACGCTGGCGGCATGCCTAAGACATGCAAGTCGTACGAGGGGACCCATTGATTTTTATTGAAAGCTGAGTGCTTGCACAAAGCTGGATTTAATTTGATATGGATTTTTCCCCTAGTGGCAAACGGGTGAGTAACACGTGGGTTACCTACCTCTAAGTTGGGGATACCAATTGGAAACGATTGTTAATACCGAATGTGCTCTACGGAGTAAAGAAGCCTTTAAAGCTTCGCTTGGAGATGGGCCTGCGGCGCATTAGCTTGTTGGTGGGGTAATGGCCTACCAAGGCAACGATGCGTA
>FQH3XDB01EIK70|810|8
GATGAACGCTGGCGGCATGCCTAAGACATGCAAGTCGAACGAGATGGCCCATTGATTAAGATTGAAAGTTGAGTGCTTGCACAATTCCGGATTTCTTATGAAGTGGATTTTCCATCGAGTGGCAAACGGGTGAATAACACGTGGGTTACCTACCTCTAAGTTGGGGATAACAGTTGGAAACGACTGATAATACCGAATATGCTCTCCGGAGTAAAGAAGCCTTTAAAGCTTCGCTAAGAGATGGGCCTGCGGCGTATTAGCTAGTTGGTGGGTAACGCCTACCAAGGCAACGATGCGTAGCCGAACTGAGAGGTTGATCGGCC
>FQH3XDB01B0FID|811|16
GATGAACGCTGGCGGCATGCCTAAGACATGCAAGTCGAACGAGAAGGCCCAATGACTTTAATTGAAAGTTTGAGTGCTTGCACAAAGACTGGATTTTATGGGGATTTGGATTTTCCTTCTAGTGGCAAACGGGTGAGTAACACGTGGGTTACCTACCTCTAAGTTGGGGATAACAGTTGGAAACGATTGCTAATACCGAATATCTTCTACGGAACAAAGAAGCTTCAAAGCTTCGCTTAGAGATGGGCCTGCGGCGTATTAGCTAGTTGGTGGGTAATGGCCTACCAAGGCGACGATGCGTAGCCGGACTGAGAGGTTAATCGGCCACACTGGGACTGAG
>FQH3XDB01ASU5Y|812|1
GATGAACGCTGGCGGCATGCCTAAGACATGCAAGTCGAACGGGATGGCCCATTGATAATAATTGAAAGTTTGCGTGCTTGCACAAAAACCGGATTTTATTGATTTGGATTTTCCATCCAGTGGCAAACGGGTGAGTAACACGTGGGTTACCTACCTCTAAGTTGGGGATAACAGTTGGAAACGATTGCTAATACCGAATGTTCTCTACGGAGTAAAGATGCCTTTAAAGCATCGCTTAGAGATGGGCCTGCGGCGTATTAGCTAGTTGGTGGGGTAATGGCCTACCAAGGCGACGATGCGTAGCCGAACTGAGAGGTTAATCGGCCACACTGGGACTGAGACACGGCCCAGACTCCTACGGGGAGA
>FQH3XDB01AJ3JV|813|3
GATGAACGCTGGCGGCATGCCTAAGACATGCAAGTCGAACGGGATGGCCCATTGATAATAATTGAAAGTTTGCGTGCTTGCACAAAAACCGGATTTTATTTGATTTGGATTTTCCATCCAGTGGCAAACGGGTGAGTAACACGTGGGTTACCTACCTCTAAGTTGGGGATAACAGTTGGAAACGATTGCTAATACCGAATGTTCTCTACGGAGTAAAGATGCCTTTAAAGCATCGCTTAGAGATGGGCCTGCGGCGTATTAGCTAGTTGGTGGGGTAATGGCCTACCAAGGCGACGATGCGTAGCCGAACTGAGAGGTTAATCGGCCACACGTGGGACTGAGACACGGCCCAGACCTCCGTACGGGA
>FQH3XDB01AX2A3|814|1
GATGAACGCTGGCGGCATGCCTAAGACATGCAAGTCGAACGGGATGGCCCAATGATTACAATTGAAAGTTTGCGTGCTTGCACAAGAGCCGGATATTGTATGATTTGGATTTTCCATCCAGTGGCAAACGGGTGAGTAACACGTGGGTTACCTGCCTCTAAGTTGGGGATAACAGTTGGAAACGATTGCTAATACCGAATGTGCTCTACGGAGTAAAGTTGCCCTTAAAGCAACGCTTAGAGATGGGCCTGCGGCGTATTAGCTAGTTGGTGGGGTAACGGCCTACCAAGGCGACGATGCGTAGCCGAACTGAGAGGTTAATCGGCCA
>FQH3XDB01CJCNU|815|1
GATGAACGCTGGCGGCATGCCTAAGACATGCAAGTCGAACGAAGGGACCCAATGACATTTATTGAAATTTGGAGAGCTTGCTCAAAGAATGGATTTAAATTGATTTGGATTATCCCTTAGTGGCAAACGGGTGAGTAACACGTGGGTTACCTGCCTCCAAGTCGGGGATAACAGTTGGAAACGATTGCTAATACCGGATGTGGTCTACGGATTAAAGAAGCCTTTAAAGCTTCGCTTGGAGATGGGCCTGCGGTGCATTAGATAGTTGGTGGGGTAATGGCCTACCAAGTCGACGATGCATAGCCGAACTGAGAGGTTAATCGGCCACATTGGGACTGAGACACGGTCCCAAACTCCTACGGGAGACAGCAGTTAGGAATATTCGTCAA
>FQH3XDB01EFGJQ|816|3
ATTGAACGCTAGCGGCATGCTTTACACATGCAAGTCGAACGGCAGCATGGGGGCAACCCTGATGGCGAGTGGCGAACGGGTGAGTAATATATCGGAACGTACCCGAGAGTGGGGGACAACCAGTCGAAAGATTGGCTAATACCGCATACGATCTACGGATGAAAGTGGGGGACCTTCGGGCCTCACGCTCCTGGAGCGGCCGATAGCTGATTAGCTAGTTGGTGGGGTAATGGCCTACCAAGGCGATGATCAGTAGCTGGTCTGAGAGGACGATCAGCCACACTGGGACTGAGACACGGCCCAGACTCCTACGGGAGGCAGCAGTGGGGAATTTTGGACAATGGGGGAACCCTGATCCAGCAATGCCGCGTGAGTGAAGAAGGCCTTCGGGTTGTAAAGCTCTTTGTCAGGAAGAAAGGACTGGGTAATACCCTGGTTCATGACGGTACCTGAAGAATAAGCACCGGCTAACTACGTGCCA
>FQH3XDB01AEF97|817|60
ATTGAACGCTGGCGGAACGCTTTACACATGCAAGTCGAACGGCAGCGGAGGTTAGCTTGCTAACCTGCCGGCGAGTGGCGAACGGGTGAGTAAGACATCGGAACGTGCCTAGATGAGGGGGACAACCAGTCGAAAGATTGGCTAATACCGCATAATATCTGAGGATGAAAGCAGGGGACCGTAAGGCCTTGCGCATTTAGAGCGGCCGATGACTGATTAGCTAGTTGGTGAGGTAAAAGGCTCACCAAGGCGACGATCAGTAGCTGGTCTGAGAGGACGACCAGCCACACTGGGACTGAGACACGGCCCAGACTCCTACGGGAGGCAGCAGTGGGGAATTTTGGACAATGGGCGCAAGCCTGATCCAGCTATTCCGCGTGTGGGATGACGGCCCTCGGTTGTAAACCACTTTTGTAGAGGACGAAAAGTCGCTTACGAATAATGAGCGA
>FQH3XDB01B51GS|818|2
ATTGAACGCTGGCGGAACGCTTTACACATGCAAGTCGAACGGTAGCGGAGGTTAGCTTGCTAACCTGCCGGCGAGTGGCGAACGGGTGAGTAAGACATCGGAACGTGCCTAGATGAGGGGACAACCAGTCGAAAGATTGGCTAATACCGCATAATATCTGAGGATGAAAGCAGGGGACCGCAAGGCCTTGCGCATTTAGAGCGGCCGATGACTGATTAGCTAGTTGGTGAGGTAAAGGCTCACCAAGGCGACGATCAGTAGCTGGTCTGAGAGGACGACCAGCCACACTGGGACTGAGACACGGCCCAGACTCCTACGGGAGGCAGCAGTGGGGAATTTTGGACAATGGGCGCAAGCCTGATCCAGCTATTCCGCGTGTGGGATGACGGCCCTCGGGGTTGTAAACCACTTTTGGTAGAGGACGAAAAGTCGCTTACGAACAATGAGCGATGCTGACGG
>FQH3XDB01EU3WI|819|1
ATTGAACGCTGGCGGAACGCTTTACACATGCAAGTCGAACGGCAGCGGAGGTTAGCTTGCTAACCTGCCGGCGAGTGGCGAACGGGTGAGTAAGACATCGGAACGTGCCTAGATGAGGGGGACAACCAGTCGAAAGATTGGCTAATACCGCATAATATCTGAGGATGAAAGCAGGGGACCGCAAGGGCCCTTGCGCATTTAGAGCGGCCGATGACTGATTAGCTAGTTGGTGAGGTAAAGGCTCACCAAGGCGACGATCAGTAGCTGG
>FQH3XDB01EW7L8|820|1
ATTGAACGCTGGCGGAACGCTTTACACATGCAAGTCGAACGGCAGCGGAGGTTAGCTTGCTAACCTGCCGGCGAGTGGCGAACGGGTGAGTAAGACATCGGAACGTGCCTAGATGAGGGGGACAACCAGTCGAAAGATTGGCTAATACCGCATAATATCTGAGGATGAAAGCAGGGGGACCTAAGGCCTTGCGCATTTAGAGCGGCCGATGACTGATTAGCTAGTTGGTGAGGTAAAGGCTCACCAAGGCGACGATCAGTAGCTGGTCTGAGAGGACGACCAGCCACACGTGGGACTGAGA
>FQH3XDB01EYXQE|821|1
ATTGAACGCTGGCGGAACGCTTTACACATGCAAGTCGAACGGCAGCGGAGGTTAGCTTGCTAACCTGCCGGCGAGTGGCGAACGGGTGAGTAAGACATCGGAACGTGCCTAGGATGAGGGGGACAACCAGTCGAAAGATTGGCTAATACCGCATAATATCTGAGGATGAAAGCAGGGGACCGTAAGGCCTTGCGCATTTAGAGCGGCGATGACTGATTAGCTAGTTGGTGAGGTAAAGGCTTCACCGAAGGCGACGATCAGTAGCTGGTCTGAGAGGACGACCAGGCCACACCTGGACTGAGCACGGCCCAGACCTCCGTACGGGAGG
>FQH3XDB01BY0GJ|822|4
ATTGAACGCTGGCGGAACGCTTTACGCATGCAAGTCGAACGGCAGCGGGGGTTAGCTTGCTAACCTGCCGGCGAGTGGCGAACGGGTGAGTAAGACATCGGAACGTGCCTAGATGAGGGGGACAACCAGTCGAAAGATTGGCTAATACCGCATAATATCTGAGGATGAAAGCAGGGGACCGGAAGGCCTTGCGCATTTAGAGCGGCCGATGACTGATTAGCTAGTTGGTGAGGTAAAGGCTCACCAAGGCGACGATCAGTAGCTGGTCTGAGAGGTCGACCAGCCACACTGGGACTGAGACACGGCCCAGACTCCTACGGGAGGCAGCAGTGGGAATTTTGGACAAGTGGGCGCAAGCCTGATCCAGCTATTCCGG
>FQH3XDB01EGJ2P|823|3
ATTGAACGCTGGCGGAACGCTTTACACATGCAAGTCGAACGGCAGCGGAGGTTAGCTTGCTAACCTGCCGGCGAGTGGCGAACGGGTGAGTAAGACATCGGAACGTGCCTAGATGAGGGGGACAACCAGTCGAAAGATTGGCTAATACCGCATAATATCTGAGGATGAAAGCAGGGGACCGCAAGGCCTTGCGCATTTAGAGCGGCCGATGACTGATTAGCTAGTTGGTGAGGTAAAGGCTCACCAAGGCGACGATCAGTAGCCTGGTCTGAAGAGGCGACCA
>FQH3XDB01EOAF1|824|1
ATTGAACGCTGGCGGAACGCTTTACACATGCAAGTCGAACGGCAGCGGAGGTTAGCTTGCTAACCTGCCGGCGAGTGGCGAACGGGTGAGTAAGCATCGGAACGTGCCTAGATGAGGGGGACAACCAGTCGAAAGATTGGCTAATACCGCATAATATCTGAGGATGAAAAGCAGGGGACCGCAAGGCCTTGCGCATTTAGAGCGGCCGATGACTGATTAGCTAGTTGGTGAGGTTAAAAGGCTCACCAAGGCGACGGATCAGTAGCTGGTCTGGAGAGGACGACCAGCC
>FQH3XDB01CJ759|825|2
ATTGAACGCTGGCGGAACGCTTTACACATGCAAGTCGAACGGTAACAGTATTAAAAGCTTGCTTTTAATAGCTGACGAGTGGCGAACGGGTGAGTAATACATCGGAACGTATCCGCTCGTGGGGGACAACTTCCCGAAAGGGGGGCTAATACCGCATGAGATCCGAGGATGAAAGAGGGGGACCCGCAAGGGGCCTCTTGCGAGCGGAGCGGCCGATGACTGATTAGCTAGTTGGTGAGGTAAAAGGCTCACCAAGGCGACGATCAGTAGCTGGTCTGAGAGGACGACCAGCCACACTGGGACTGAGACACGGCCCAGACC
>FQH3XDB01BOTCE|826|7
ATTGAACGCTGGCGGCAGGCCTAACACATGCAAGTCGAGCGGTAACACAGGGAGCTTGCTCCTGGGTGACGAGCGGCGGACGGGTGAGTAATGTCTGGGAAACTGCCTGATGGAGGGGGATAACTACTGGAAACGGTAGCTAATACCGCATAACGTCGCAAGACCAAAGAGGGGGGACCTTCGGGCCTCTTGCCATCAGATGTGCCCAGATGGGATTAGCTAGTAGGTGGGGTAACGGCTCACCTAGGCGACGATCCCTAGCTGGTCTGAGAGGATGACCAGCCACACTGGAACCTGAGACACGGTCCAGACTCCTACGGGAGCAGCAGTGGGAATATT
>FQH3XDB01D7HDG|827|1
ATTGAACGCTGGCGGCAGGCCTAACACATGCAAGTCGAGCGGTAGCACAGAGAGCTTGCTCTCGGGTGACGAGCGGCGGACGGGTGAGTAATGTCTGGGAAACTGCCTGATGGAGGGGGATAACTACTGGAAACGGTAGCTAATACCGCATAACGTCGCAAGACCAAAGGAGGGGGACCTTCGGGGCCTCTTGCCATCGGATGTGCCCAGATGGGATTAGCTAGTAGGTGGGGTAACGGCTCACCTAGGCGACGATCCCTAGCTGGTCTGAGAGGATGACCAGCCACACTGGAACTGAGACACGGTCCAGACTCCTACGGGAGCAGCAGTGGGAATATGCACAATGGGCGCAAGCCTGATGCAGCCATGCCGCGTGTATG
>FQH3XDB01EH586|828|2
ATTGAACGCTGGCGGCAGGCCTAGCACATGCAAGTCGAGCGGATGACGGGAGCTTGCTCCTTGATTCAGCGGCGGACGGGTGAGTAATGCCTAGGAATCTGCCTGGTAGTGGGGGACAACGTTTCGAAAGGAACGCTAATACCGCATACGTCCTACGGGAGAAAGCAGGGGACCTTCGGGCCTTGCGCTATCAGATGAGCCTAGGTCGGATTAGCTAGTTGGTGGGGTAATGGGCTCACCAAGGCGACGATCCGTAACTGGTCTGAGAAGATGATCAGTCACACTGGAACTGAGACACGGTCCAGACTCCTACGGGAGGCAGCAGTGGGGAATATTGGACAATGGGCGAAAGCCTGATCCAGCCATGCCGCGTGTGTGAAGAAGGTCTT
>FQH3XDB01CC2I5|829|1
ATTGAACGCTGGCGGCAGGCTTAACACATGCAAGTCGAACGGTAACGGGAAGAAGCTTGCTTCTTTGCCGACGAGTGGCGGACGGGTGAGTAATGCTTGGGGATCTGGCTTATGGCGGGGGATAACGACGGGAAACTGTCGCTAATACCGCGTAGTGTCGGGAGACGAAAGTGCGGGACCTTAGGGCCGCATGCCATGAGATGAGCCCAAGTGGGATTAGGTAGTTGGTGGGGTAAAGGCCTACCAAGCCGTCGATCTCTAGCTGGTCTGAGAGGATGACCAGCCACACCGGGACTGAGACACGGCCCGGACTCCTACGGGAGGCAGCAGTGGGGAATATTGCGCAAGTGGGGGCAACCCTGACGCAGCCATGCCGCGTGAATG
>FQH3XDB01DL76H|830|2
ATTGAACGCTGGCGGCAGGCTTAACACATGCAAGTCGAACGGTAACAGGAGGAAGCTTGCTTTCTTGCTGACGAGTGGCGGACGGGTGAGTAATGCTTGGGAATCTGGCTTATGGAGGGGGATAACTGCGGGAAACTGCAGCTAATACCGCGTAGTATCGAAAGATGAAAGTGCGGGATCGTAAGGCCGCATGCCATGAGATGAGCCCAAGTGGGATTAGGTAGTTGGTGGGGTAAAGGCCTACCAAGCCTGCGATCTCTAGCTGGTCTGAGAGGATGACCAGCCACACCGGGACTGAGACACGGCCCGGACTCCTACGGGAGGCAGCAGTGGGGAATATTGCGCAATGGGGGCAACCCTGACGCAGCCATGCCGCGTGAATGAAGAA
>FQH3XDB01CUOR3|831|1
ATTGAACGCTGGCGGCAGGCTTAACACATGCAAGTCGAACGGTAACAGGGATTAGCTTGCTAATCTGCTGACGAGTGGCGGACGGGTGAGTAATGCTTGGGAATCTGGCTTATGGAGGGGGATAACTACGGGAAACTGTAGCTAATACCGCGTAATATCTTTGGATTAAAGGGTGGGACTTTCGGGCCACCTGCCATAAGATGAGCCCAAGTGGGATTAGGTAGTTGGTTAGGTAAAGGCTGACCAAGCCGACGATCTCTAGCTGGTCTGAGAGGATGACCAGCCACACTGGAACTGAGACACGGTCCAGACTCCTACGGGAGGCAGCAGTGGGGAATATTGCACAATGGGGGAAACCCTGATGCAGCCATGCCGCGTGAATGAA
>FQH3XDB01AU7IO|832|4
ATTGAACGCTGGCGGCAGGCTTAACACATGCAAGTCGAACGGTAACAGGAAGAAACTTGTTTCTTTGCTGACGAGTGGCGGACGGGTGAGTAATGCTTGGGAATCTGGCTTATGGAGGGGGATAACAACGGGGAAACTGTTGCTAATACCGCGTAGTATCGGAAGATGAAAGTGTGGGACCTTCGGGCCACATGCCATGAGATGAGCCCAAGTGGGATTAGGTAGTTGGTGGGGTAAAGGCCTACCAAGCCTGCGATCTCTAGCTGGTCTGAGAGGATGACCAGCCACACCGGGACTGAGACACGGCCCGGACTCCTACGGGAGGCAGCAGTGGGGAATATTGCGCAAGTGGGGGCAACCCTGACGCAGCCATGCCC
>FQH3XDB01DBRYZ|833|1
ATTGAACGCTGGCGGCAGGCTTAACACATGCAAGTCGAACGGTAACAGGAAGAAACTTGTTTCTTTGCTGACGAGTGGCGGACGGGTGAGTAATGCTTGGGAATCTGGCTTATGGAGGGGGATAACAACGGGAAACTGTTGCTAATACCGCGTGGTATCGAGAGATGAGAGTGTGGGACCTTCGGGCCACATGCCATGAGATGAGCCCAAGTGGGATTAGGTAGCTGGTGGGGTAAAGGCCTACCAAGCCTGCGATCTCTAGCTGGTCTGAGAGGATGACCAGCCACACCGGGACTGAGACCACGGCCCGGACTCCGTACGGGAGCAGCAG
>FQH3XDB01DPRML|834|15
ATTGAACGCTGGCGGCAGGCTTAACACATGCAAGTCGAACGGTAACAGGAAGAAACTTGTTTCTTTGCTGACGAGTGGCGGACGGGTGAGTAATGCTTGGGAATCTGGCTTATGGAGGGGGATAACTGCGGGAAACTGCAGCTAATACCGCGTAGAATCGGAAGATGAAAGTGTGGGACCTTCGGGCCACATGCCATGAGATGAGCCCAAGTGGGATTAGGTAGTTGGTGGGGTAAAGGCCTACCAAGCCTGCGATCTCTAGCTGGTCTGAGAGGATGACCAGCCACACCGGGACTGAGACACGGCCCGGACTCCTACGGGAGGCAGCAGTGGGGAATATTGCGCAATGGGGCAACCCTGACGCAGCCATGCCGCGTGAATGAAGAAGGCCTTCGGGTTGTAAAGTTCTTCGGTGGTGAGGAAGGCGTGGTGTTTAATAGACGATCATGATTGACGTT
>FQH3XDB01DRPH4|835|1
ATTGAACGCTGGCGGCAGGCTTAACACATGCAAGTCGAACGGTAACGGGTAAGTACTTGTACTTATGCCGACGAGTGGCGGACGGGTGAGTAATGCTTGGGAATCTGGCTTATGGCGGGGGATAACGACGGGAAACTGTCGCTAATACCGCGTAGTGTCGGGAGATGAAAGTGCGGGGACCTTATGGCCGCATGCCATGAGATGAGCCCAAGTGGGATTAGGTAGTTGGTGGGGTAAAGGCCTACCAAGCCGTCGATCTCTAGCTGGTCTGAGAGGATGACCAGCCACACCGGGGACTGAGACACGGCCCGGACTCCTACGGGAGGCAGCAGTGGGGAATATTGCGCAATGGGGGCAACCCGTGACGCAGCCATGCCGCGTGAA
>FQH3XDB01BV6DB|836|2
ATTGAACGCTGGCGGCAGGCTTAACACATGCAAGTCGAGCGGGGAGAAGGTAGCTTGCTACTGGAACCTAGCGGCGGACGGGTGAGTAATGCTTAGGAATCTGCCTATTAGTGGGGGACAACGTTTCGAAAGGGACGCTAATACCGCATACGCCCTACGGGGGAAAGCAGGGGATCTTCGGACCTTGCGCTAATAGATGAGCCTAAGTCGGATTAGCTAGTTGGTGGGGTAAAGCCTACCAAGGCGACGATCTGTAGCGGGTCTGAGAGGATGATCCGCCACACTGGGACTGAGACACGGCCCAGACTCCTACGGGAGGCAGCAGTGGGGAATATTGGACAATGGGCGAAGCCTGATCCAGCCATGCCGCGTGTGTGAAGAAGGT
>FQH3XDB01BH0Z2|837|1
ATTGAACGCTGGCGGCAGGCTTAACACATGCAAGTCGAGCGGAGGAAGGTAGCTTGCTACTGGACTTAGCGGCGGACGGGTGAGTAAAGCTTAGGAATCTGCCTATTAGTGGGGGACAACGTTTCGAAAGGGACGCTAATACCGCATACGCCCTACGGGGGAAAGCAGGGGATCGTAAGACCTTGTGCTAATAGATGAGCCTAAGTCGGATTAGCTAGTTGGTAGGGTAAAGGCCTACCAAGGCGACGATCTGTAGCGGGTCTGAGAGGATGATCCGCCACACTGGGACTGAGACACGGCCCAGACTCCTACGGGAGGCAGCAGTGGGGAATATTGGACAATGGGCGAAAGCCTGATCCAGCCATGCCGCGTGTGTGAAGAAGGCCTTATGGTTGT
>FQH3XDB01DJ7FZ|838|1
ATTGAACGCTGGCGGCATGCCTTACACATGCAAGTCGAACGGCAGCGCGGGAGCAATCCTGGCGGCGAGTGGCGAACGGGTGAGTAATACATCGGAACGTGCCCAATCGTGGGGGATAACGCAGCGAAAGCTGTGCTAATACCGCATACGATCTACGGATGAAAGCAGGGGATCGCAAGACCTTGCGCGAATGGAGCGGCCGATGGCAGATTAGGTAGTTGGTGAGGTAAAGGCTCACCAAGCCTTCGATCTGTAGCTGGTCTGAGAGGACGACCAGCCACACTGGGACTGAGACACGGCCCA
>FQH3XDB01BJJQ3|839|7
ATTGAACGCTGGCGGCATGCCTTACACATGCAAGTCGAACGGCAGCGCGGGAGCTTGCTCCTGGCGGCGAGTGGCGAACGGGTGAGTAATACATCGGAACGTGTCCGTTTGTGGGGGACAACCAGCCGAAAGGTTGGCTAATACCGCATAAGACCTGAGGGTGAAAGCCGGGGACCGCAAGGCCTGGCGCAGACGGAGCGGCCGATGATTGATTAGCTTGTTGGCGGGTAAAGGCCACCAAGGCGACGATCAATAGCTGGTCTGAGAGGACGACCAGCCACACTGGAACTGAGACACGGTCCAGACTCCTACGGGAGGCAGCAGTGGGGAATTTTGGGACAATGGGGGCAA
>FQH3XDB01DI9WZ|840|6
ATTGAACGCTGGCGGCATGCCTTACACATGCAAGTCGAACGGCAGCGCGGGAGCTTGCTCCTGGCGGCGAGTGGCGAACGGGTGAGTAATACATCGGAACGTGTCCGTTTGTGGGGGACAACCAGCCGAAAGGTTGGCTAATACCGCATAAGACCTGAGGGTGAAAGCCGGGGATCGCAAGACCTGGCGCAGACGGAGCGGCCGATGATTGATTAGCTGGTTGGCGGGGTAAAGGCCCACCAAGGCGACGATCAATAGCTGGTCTGAGAGGACGACCAGCCACACTGGAACTGAGACACGGTCCAGACTCCTACGGGAGGCAGCAGTGGGGAATTTTGGACAATGGGGGCAACCCTGATCCAGCCATGCCGCGTGCGGGAAGAAGGCCTTCGGGTTGTAAACCGC
>FQH3XDB01DV0JS|841|2
ATTGAACGCTGGCGGCATGCCTTACACATGCAAGTCGAACGGCAGCGCGGGAGCTTGCTCCTGGCGGCGAGTGGCGAACGGGTGAGTAATACATCGGAACGTGTCCGTTTGTGGGGGACAACCAGCCGAAAGGTTGGCTAATACCGCATAAGACCTGAGGGTGAAAGCCGGGGACCGCAAGGCCTGGCGCAGACGGAGCGGCCGATGATTGATTAGCTTGTTGGTGGGGTAACGGCTCACCAAGTCGACGATCAGTAGGGGTCCTGAGAGGGAGATCCCCCACATTGGAACTGAGACACGGTCCAAACTCCTACGGGAGGCAGCAGTGAGGAATATTGGGTCAAAGTGGGGCGAGAGCCTGAACCAGCCAAGTCGCGTGAAGGAAGACGGTTCTATGGATTGTAAACTTCTTTGCCGA
>FQH3XDB01BZ706|842|1
ATTGAACGCTGGCGGCATGCCTTACACATGCAAGTCGAACGGCAGCACGGGAGCAATCCTGGTGGCGAGTGGCGAACGGGTGAGTAATATATCGGAACGTGCCCAGTTGTGGGGGATAGCCCGGCGAAAGCCGGATTAATACCGCATACGACCTGAGGGTGAAAGCGGGGGATCGCAAGACCTCGCGCAATTGGAGCGGCCGATATCAGATTAGCTAGTTGGTGGGGTAAAGGCCTACCAAGGCGACGATCTGTAGTCTGGTCTGAGAGGACGACCAGCCACACGTGGGACTGAGACACGGCCCAGACCTCCGTACGGGA
>FQH3XDB01DV75M|843|1
ATTGAACGCTGGCGGCATGCCTTACACATGCAAGTCGAACGGCAGCGCGGAGCTTGCTCCTGGCGGCGAGTGGCGAACGGTGAGTAATACATCGGAACGTGTCCGTTTGTGGGGACAACCAGCCGAAAGGTTGGCTAATACCGCATAAGACCTGAGGGTGAAAGCCGGGATCGCAAGACCTGGCGCAGACGGAGCGGCCGATGATTGATTAGCTGGTTGCGGGTAAAGGCCACCAAGGCGACGATCAATAGCTGGTCTGAGAGGACGACCAGCCACACTGGAACTGAGACACGGTCCAGACTCCTACGGAGGCAGCAGTGGGAATTTTGGACAATGGGGCAA
>FQH3XDB01AROJO|844|1
ATTGAACGCTGGCGGCATGCCTTACACATGCAAGTCGAACGGCAGCGCGGGAGCTTGCTCCTGGCGGCGAGTGGCGAACGGGTGAGTAATACATCGGAACGTGTCCGTTTGTGGGGACAACCGAGCCGAAAGGTTGGCTAATACCGCATAAGACCTGAGGTGAAAGCCGGGGATCGCAAGACCTGGCGCAGACGGAGCGGCCGATGATTGATTAGCTAGTTGGCGGGTAAAGGCCCACCAAGGCGACGGATTAATAGGCTGGTCTGAGAGGACGACCAGCCACACTGGAACTGAGACACGGTCCAGACTCCTAC
>FQH3XDB01AM4ZM|845|4
ATTGAACGCTGGCGGCATGCCTTACACATGCAAGTCGAACGGCAGCGCGGGAGCTTGCTCCTGGCGGCGAGTGGCGAACGGGTGAGTAATACATCGGAACGTGTCCGTTTGTGGGGGACAACCAGCCGAAAGGTTGGCTAATACCGCATAAGACCTGAGGGTGAAAGCCGGGGACCGCAAGGCCTGGCGCAGACGGAGCGGCCGATGATTGATTAGCTTGGTTGGCGGGGTAAAGGCCCACCAAGGCGACGATCAATAGCTGGTCTGAGAGGACGACCAGCCACACTGGAACT
>FQH3XDB01AL48C|846|13
ATTGAACGCTGGCGGCATGCCTTACACATGCAAGTCGAACGGCAGCGCGGGAGCTTGCTCCTGGCGGCGAGTGGCGAACGGGTGAGTAATACATCGGAACGTGTCCGTTTGTGGGGGACAACCAGCCGAAAGGTTGGCTAATACCGCATAAGACCTGAGGGTGAAAGCCGGGGACCGCAAGGCCTGGCGCAGACGGAGCGGCCGATGATTGATTAGCTTGTTGGCGGGGTAAAGGCCCACCAAGGCGACGATCAATAGCTGGTCTGAGAGGACGACCAGCCACACTGGAACTGAGACACGGTCCAGACTCCTACGGGAGGCAGCAGTGGGGAATTTTGGACAATGGGGGCAACCCTGATCCAGCCATGCCGCGTGCGGGAAGAAGGGCCTTCGGGTTGTAAACCGCTTTGTCAGGGCGAAAAGGACGGGTTAAGAGCTAGTTCTGCTGACGGTACCTGAAGAA
>FQH3XDB01CWJQ1|847|1
ATTGAACGCTGGCGGCATGCCTTACACATGCAAGTCGAACGGCAGCGCGGGAGCTTGCTCCTGGCGGCGAGTGGCGAACGGGTGAGTAATACATCGGAACGTGTCCGTTTGTGGGGGACAACCAGCCGGAAGGTTGGCTAATACCGCATAAGACCTGAGGGTGAAAGCCGGGGATCGCAAGACCTGGCGCAGACGGAGCGGCCGATGATTGATTAGCTGGTTGGCGGGGTAAAGGCCCACCAAGGCGACGATCAATAGCTGGTCTGAGAGGACGACCAGCCACACTGGAACTGAGACACGGTCCCAGACTCCTACGGGAGGCAGCAGTGGGGAATTTTGGACAATGGGGGCAACCCTGATCCAGCCATGCCGCGTGCGGGAAGAAGGCCTTCGGGTTGTAAACCGCTTTTGTCAGGGACGAAAGGGACGGTTAAGAGCTAGTTCTGCTGACGGTACCTGAA
>FQH3XDB01EASKU|848|2
ATTGAACGCTGGCGGCATGCCTTACACATGCAAGTCGAACGGCAGCGCGGGAGCTTGCTCCTGGCGGCGAGTGGCGAACGGGTGAGTAATACATCGGAACGTGTCCGTTTGTGGGGGACAACCAGCCGAAAGGTTGGCTAATACCGCATAAGACCTGAGGGTGAAAGCCGGGGATCGCAAGACCTGGCGCAGACGGAGCGGCCGATGATTGATTAGCTAGTTGCGGGTAAAGGCCCACCAAGGCGACGATCAATAGCTGGTCTGAGAGGACGACCAGCCACACTGGAACTGAGACACGGTCCAGACTCCTACGGGAGGCAGCAGTGGGGAATATTGCACAATGGAGGAAACTCTGATGCAGCGACGCCGCGTGAGTGAAGAAGTATTTCGGTATGTAAAGCTCTATC
>FQH3XDB01BVVAK|849|5
ATTGAACGCTGGCGGCATGCCTTACACATGCAAGTCGAACGGCAGCGCGGGAGCTTGCTCCTGGCGGCGAGTGGCGAACGGGTGAGTAATACATCGGAACGTGTCCGTTTGTGGGGGACAACCAGCCGAAAGGTTGGCTAATACCGCATAAGACCTGAGGGTGAAAGCCGGGGACCGCAAGGCCTGGCGCAGACGGAGCGGCCGATGATTGATTAGCTGGTTGGCGGGGTAAAGGCCCACCAAGGCGACGATCAATAGCTGGTCTGAGAGGACGACCAGCCACACTGGAACTGAGACACGGTCCAGACTCCTACGGGAGGCAGCAGTAGGGAATTTTGCGCAATGGGCGAAAGCCTGACGCAGCAACGCCGCGTGAATGATGAAGCCCTTCGGGGTGTAAGTTCTGTCAGTAGGGACGAAACTTGACGGTACCTACAGAGGAAGCACCGGCTAACTCCGTGCCAGCAGCCGCGGTAA
>FQH3XDB01DCUXS|850|1
ATTGAACGCTGGCGGCATGCCTTACACATGCAAGTCGAACGGCAGCGGCGGGAGCTTGCTCCTGGCGGCGAGTGGCGAACGGGTGAGTAATACATCGGAACGTGTCCGTTTGTGGGGACAACCAGCCGAAAGGTTGGCTAATACCGCATAAGACCTGAGGGTGAAAGCCGGGGACTGCAAGGCCTGGCGCAGACGGAGCGGCCGATGATTGATTAGCTTGTTGGCGGGTAAGCCACCAAGGCGACGATCAATAGCTGGTCTGAGAGGACGACCAGCCACACTGGAACTGAGACACGGTCCAGACTCCTACGGGAGGCCAGCAGTGGGAATTTGGACAA
>FQH3XDB01EOQLT|851|36
ATTGAACGCTGGCGGCATGCTTTACACATGCAAGTCGAACGGCAGCATGGAGAGCTTGCTCTCTGATGGCGAGTGGCGAACGGGTGAGTAATACATCGGAACGTGTCCTGTTGTGGGGGATAACTACTCGAAAGAGTAGCTAATACCGCATAAGACCTGAGGGTGAAAGCGGGGGACCGAAAGGCCTCGCGCAGTTGGAGCGGCCGATGACTGATTAGCTAGTTGGTGGGTAAAGGCCTACCAAGGCGACGATCAGTAGCTGGTCTGAGAGGACGACCAGCCACATTGGGACTGAGACACGGCCCAAAACTCCTACGGGAGGCAGCAGTGGGGAATTTTGGACAATGGGGGCAACCCTGATCCAGCCATGCCGCGTGCGGGAAGAAGGCCTTCGGGTTGTAAACCGCTTTGTTAGGAACGAAAGGACTTGACGAATAATCAGAGCTGCTGACGGTACCTAAGAATAAGCACCGGCTAACTACG
>FQH3XDB01BVKI7|852|18
ATTGAACGCTGGCGGCATGCTTTACACATGCAAGTCGAACGGCAGCGCGGGCTTCGGCCTGGCGGCGAGTGGCGGACGGGTGAGTAAGACATCGGAACGTGCCATAAGGAGGGGGACAACCAGTCGAAAGATTGGCTAATACCGCATAATATCCACGGATCAAAGCAGGGGACCTACGGGCCCTGCGCCATATGAGCGGCCGATGGCTGATTAGCTAGTTGGTGGGTAAAGGCCTACCAAGGCGATGATCAGTAGCTGGTCTGAGAGGACGATCAGCCACATTGGGACTGAGACACGGCCCAAACTCCTACGGGAGGCAGCAGTGGGAATTTTGGACAAGTGGGGAACCTGATCCAGCAATGCCGCGTGAGTG
>FQH3XDB01EO77O|853|4
ATTGAACGCTGGCGGCATGCTTTACACATGCAAGTCGAACGGCAGCGCGGGCTTCGGCCTGGCGGCGAGTGGCGGACGGGTGAGTAAGACATCGGAACGTGCCATAAGGAGGGGGACAACCAGTCGAAAGATTGGCCTAATACCGCATAATATCCACGGATCAAAGCAGGGGACCAACGGGCCCTGCGCCATATGAGCGGCCGATGGCTGATTAGCTAGTTGGTGGGGTAAAGGCCTACCAAGGCGATGATCAGTAGCTGGTCTGAGAGGACGATCAGCCACATTGGGACTGAGACACGGCCCAAAACTCCTACGGGAGCAGCAGTGGGGAATTTTGGACAAGTGGGGGAACCCTGATCCAGCAA
>FQH3XDB01BVE1M|854|1
ATTGAACGCTGGCGGCATGCTTTACACATGCAAGTCGAACGGCAGCGCGGGCTTCGGCCTGGCGGCGAGTGGCGGACGGGTGAGTAAGACATCGGAACGTGCCATAAGGAGGGGGACAACCAGTCGAAAGATTGGCTAATACCGCATAATATCCACGGATCAAAGCAGGGGACCTACGGGCCCTGCGCCATATGGAGCGGCCGATGGCCTGATTAGCTAGTTGGTGGGGTAAAGGCCTACCAAGGCGATGATCAGTAGCTGGTCTGAGAGGACGATCAGCCACATTGGGACTGAGACACGGCCCCAAACTCCTAC
>FQH3XDB01CM8GJ|855|13
ATTGAACGCTGGCGGCATGCTTTACACATGCAAGTCGAACGGCAGCACAGGGAGCTTGCTCCCGGGTGGCGAGTGGCGCACGGGTGAGTAATACATCGGAACGTGTCCTGTTGTGGGGATAACTGCTCGAAAGGTGGCTAATACCGCATGAGACCTGAGGGTGAAAGCGGGGGATCGCAAGACCTCGCGCAACTGGAGCGGCCGATGCCCGATTAGCTAGTTGGTGAGGTAAAGGCTCACCAAGGCGACGATCGGTAGCTGGTCTGAGAGGACGACCAGCCACACTGGGACTGAGACACGGCCCAGACTCCTACGGGAGGCAGCAGTGGGGAATTTTGGACAATGGGGCAACCCTGATCCAGCCATGCCGCGTGCAGGATGAAGGCCTTCGGGTTGTAAACTGCTTTGTCAGGGACGAAAAGGATCGTGCTAAGTACCATGGTCTGC
>FQH3XDB01DDNOG|856|1
ATTGAACGCTGGCGGCATGCTTTACACATGCAAGTCGAACGGCAGCACAGGGAGCTTTGCTCCCGGGTGGCGAGTGGCGCACGGGTGAGTAATACATCGGAACGTGTCCTGGTTGTGGGGGATAACTGCTCGAAAGGGTGGCTAATACCGCATGAGACCTGAGGGTGAAAGCGGGGGATCGGCAAGACCTCGCGCAAACTGGAGCGGCCGATGCCCCGATTAGCTAGTTGGTGAGGTAAAGGCTCACCAAGGCGACGATCGGTAGCTGGTCTGAGAGGACGACCAGCCACACTGGGACTGAGACACGGCCCCAGACTCCTACGGGAGCAGCAGTGGGGAATTTTGGACAATGGGGGCAA
>FQH3XDB01E2N7V|857|11
ATTGAACGCTGGCGGCATGCTTTACACATGCAAGTCGAACGGCAGCATGGGGGCAACCCTGATGGCGAGTGGCGAACGGGTGAGTAATATATCGGAACGTACCCGAGAGTGGGGGACAACCAGTCGAAAGATTGGCTAATACCGCATACGATCTACGGATGAAAGTGGGGGACCTTCGGGCCTCACGCTCCTGGAGCGGCCGATAGCTGATTAGCTAGTTGGTGGGTAATGGCCTACCAAGGCGATGATCAGTAGCTGGTCTGAGAGGACGATCAGCCACACTGGGACTGAGACACGGCCCAGACTCCTACGGGAGG
>FQH3XDB01CVSAO|858|7
ATTGAACGCTGGCGGCATGCTTTACACATGCAAGTCGAACGGCAGCATGGAGAGCTTGCTCTCTGATGGCGAGTGGCGAACGGGTGAGTAATACATCGGAACGTGTCCTGTTGTGGGGGATAACTACTCGAAAGAGTAGCTAATACCGCATAAGACCTGAGGGTGAAAGCGGGGACCGAAAGGCCTCGCGCAGTTGGAGCGGCCGATGACTGATTAGCTAGTTGGTGGGTAAAGGCCTACCAAGGCGACGATCAGTAGCTGGTCTGAGAGGACGACCAGCCACATTGGGACTGAGACACGGCCCAAACTCCTACGGGAGGCAGCAGTGGGAATTTGGACAATGGGGCAACCTGATCCAGCCATGCCGCGTGC
>FQH3XDB01DQ1PE|859|3
ATTGAACGCTGGCGGCATGCTTTACACATGCAAGTCGAACGGCAGCGGGGAACTAAGCTTGCTTAGTTCTGCCGGCGAGTGGCGAACGGGTGAGTACAACATCGGAACGTGTCCGTTTGTGGGGGACAACTGCTCGAAAGGGTAGCTAATACCGCATAAGACCTGAGGGTGAAAGTGGGGATCGCAAGACCTCACGCGAACGGAGCGGCCGATGACTGATTAGGTAGTTGGTGAGGTAAAGGCCCACCAAGCCGACGATCAGTAGCTGGTCTGAGAGGACGACCAGCCACACTGGAACTGAGACACGGTCCAGACTCCTACGGAGGCAGCAGTGGGGAATTTTGGACAATGGGGAAACCCTGATCCAGCCATGCCGCGTGCGGGAAGAAGGCCTC
>FQH3XDB01DAWTR|860|1
ATTGAACGCTGGCGGCATGCTTTACACATGCAAGTCGAACGGCAGCATGGAGAGCTTGCTCTCTGATGGCGAGTGGCGAACGGGTGAGTAATACATCGGAACGTGTCCTGTTGTGGGGGATAACTACTCGAAAGAGTAGCTAATACCGCATAAGACCTGAGGTGAAAGCGGGGACCAAAGGCCTCGCGCAGTTGAGCGCCGATGACTGATTAGCTAGTTGGTGGGTAAAGGCCTACCAAGGCGACGATCAGTAGCTGGGTCTGAGAGGACGACCAGCCACATTGGGACT
>FQH3XDB01BDFC4|861|8
ATTGAACGCTGGCGGCATGCTTTACACATGCAAGTCGAACGGCAGCGGGGAACTAAGCTTGCTTAGTTCTGCCGGCGAGTGGCGAACGGGTGAGTACAACATCGGAACGTGTCCGTTTGTGGGGACAACTGCTCGAAAGGGTAGCTAATACCGCATAAGACCTGAGGGTGAAAGTGGGGATCGCAAGACCTCACGCGAACGGAGCGGCCGATGACTGATTAGGTAGTTGGTGAGGTAAAGGCCCACCAAGCCGACGATCAGTAGCTGGTCTGAGAGGACGACCAGCCACACTGGAACTGAGACACGGTCCAGACTCCTACGGGAGGCAGCAGTGGGGAATTTTGGACAATGGGGGAAACCCTGATCCAGCCATGCCGCGTGCGGGAAGAAGGCCTTCGGGTTGTAAACCGCTTTGTCAGGGACGAAAGTTACTATTGAACAAATAGTGATGCTGACGGTACCTGAAGAATAAGCACCGGCTAACTACGTGCCAGCAGCCG
>FQH3XDB01CJHRX|862|1
ATTGAACGCTGGCGGCATGCTTTACACATGCAAGTCGAACGGTAACGCGGGGGCAACCCTGGCGACGAGTGGCGAACGGGTGAGTAATACATCGGAACATGTCCTGGAGTGGGGGATAGCTCGGCGAAAGCCGGATTAATACCGCATAAGCTCTGAGGAGGAAAGCGGGGGACCTTCGGGCCTCGCGCTGCAGGAGTGGCCGATGTCGGATTAGCTAGTTGGTGAGGTAAAGGCTCACCAAGGCGACGATCCGTAGCTGGTCTGAGAGGATGATCAGCCACACTGGGACTGAGACACGGCCCAGACTCCTACGGGAGGCAGCAGTGGGGAATTTTGGACAATGGGGGCAACCCTGATCCAGCCATGCCGCGTGAGTGAAGAAGGCCTCGGTTGTAAGC
>FQH3XDB01DMXSZ|863|2
ATTGAACGCTGGCGGCATGCTTTACACATGCAAGTCGAACGGCAACGGGGAACTAAGCTTGCTTAGTTCTGCCGGCGAGTGGCGAACGGGTGAGTACAACATCGGAACGTGTCCGTTTGTGGGGGACAACTGCTCGAAAGGGTAGCTAATACCGCATAAGACCTGAGGGTGAAAGTGGGGGATCGCAAGACCTCACGCGAACGGAGCGGCCGATGACTGATTAGGTAGTTGGTGAGGTAAAGGCCCACCAAGCCGACGATCAGTAGCTGGTCTGAGAGGACGACCAGCCACACTGGAACTGAGACACGGTCCAGACTCCTACGGGAGGCAGCAGTGGGGAATTTTGGACAATGGGGAAACCCTGATCCAGCCATGCCGCGTGCGGGAAGAAGGCCTTCGGGTTGTAAACCGCTTTAGTCAGGACGAAAAGTTACTATTAGAACAAATAGTGATGCTGACGGTACCTGAAGAATAAGCACCGGCTAACTACGTGCCAGCAGCCGACG
>FQH3XDB01AYBMC|864|2
GTTGAACGCTGGCGGCGTGCCTAACACATGCAAGTCGAACGCGAAAGGGGCTTCGGCCCTGAGTAAAGTGGCGCACGGGTGAGTACCGCGTGGATAATCTGCCTTCAAGATGGGGATAACAGTTGGAAACGACTGCTAATACCGAATACGCTCACAATACGAATTTTGTGGGGAAAAGACGGCCTCTGAATATGCTGTTGCTTGAAGATGAGTCCGCGTCCCATTAGCTAGTTGCGGGTAAAGCCCACCAAGGCGACGATGGGTAGCCGATCTGAGAGGATGATCGGCCA
>FQH3XDB01BQ5MD|865|2
ATTGAACGCTGGCGGCGTGCCTAACACATGCAAGTCGTACGCGAAAGGGTCTTCGGACCCGAGTAAAGTGGCGCACGGGTGAGTAACGCGTGGATAATCTACCGGGAAGTGGGGAATAACGACTGGAAACGGTCGCTAATACCGCATAAGTTCTGCAAAGAAGAAAGATGGCCTCTGCATATGCTATCGCTTTTCGATGAGTCCGCGTCCCATTAGCTAGTTGGTGAGGTAAAGGCCCACCAAGGCGACGATGGGTAGCTGGTCTAAGAGGATGATCAGTCACATTGGAACTGGAACACGGTCCAAAACTCCTACGGGAGGCAGCAGTGGGGAATATTGCGCAATGGGGCGAAAGCCTGACGCAGCGACGCCGCGTGAGGGATGAAGGTCTTCGGATCGGTAAACCT
>FQH3XDB01DBL4Y|866|1
ATTGAACGCTGGCGGCGTGCCTAACACATGCAAGTCGTACGCGAAAGGGGCTTCGGCCCCGAGTAAAGTGGCGCACGGGTGAGTAACGCGTGGATAATCTACCGGGAAGTGGGGAATAACGACTGGAAACGGTCGCTAATACCGCATGAGCTTCGTATAAGATATGAAGGAAAGATGGCCTCTGCATATGCTATCGCTTTTCGATGAGTCCGCGTCCCATTAGCTAGTTGGTGAGGTAAAGGCCCACCAAGGCTACGATGGGTAGCTGGTCTAAGAGGATGATCAGTCACATTGGAACTGGAACACGGTCCAAACTCCTACGGGAGGCAGCAGTGGGGAATATTGCGCAATGGGCGAAAGCCTGACGCAGCGACGCCGCGTGA
>FQH3XDB01CZISY|867|2
ATTGAACGCTGGCGGCGTGCCTAACACATGCAAGTCGTACGCGAAAGGGTCTTCGGATCCGAGTAAAGTGGCGCACGGGTGAGTAACGCGTGGATAATCTACCGGGAAGTGGGGAATAACGACTGGAAACGGTCGCTAATACCGCATAAGTTCTGCAAAGAAGAAAGATGGCCTCTGCATATGCTGTCGCTTTTCGATGAGTCCGCGTCCCATTAGCTAGTTGGTGAGGTAAAGGCCCACCAAGGCGACGATGGGTAGCTGGTCTAAGAGGATGATCAGTCACATTGGAACTGGAACACGGTCCAAACTCCTACGGGAGCAGCAGTGGGGAATATTGCGCAATGGGCGAAAGCCTGACGCAGCGACG
>FQH3XDB01BKLQL|868|3
ATTGAACGCTGGCGGCGTGCCTAACACATGCAAGTCGTACGTGAAAGGGACTTCGGTCCCGAGTAAAGTGGCGCACGGGTGAGTAACACGTGGATAATCTACCGGGAAGTGGGGAATAACGACTGGAAACGGTCGCTAATACCGCATACGTTCTGCAAAGAAGAAAGATGGCCTCTGCATATGCTATCGCTTTTCGATGAGTCCGCGTCCCATTAGCTAGTTGGTGAGGTAAAGGCCCACCAAGGCGACGATGGGTAGCTGGTCTAAGAGGATGATCAGTCACATTGGAACTGGAACACGGTCCAAAACTCCTACGGGAGGCAGCAGTGGGGAATATTGCGCAATGGGCGAAAGCCTGACGCAGCGACGCCGCGTGAGGGATGAAGGTCTTCGGATCGGTAAACCTCTGTCAAG
>FQH3XDB01A2ZTP|869|1
ATTGAACGCTGGCGGCGTGCTTAACACATGCAAGTCGAACGAGAAAGTTCCTTCGGGAATGAGTAGAGTGGCGCACGGGTGAGTAACGCGTGGATAATCTACCGGGGAGTGGGGAATAACAGTTGGAAACGGCTGCTAATACCGCATACGCTGCATATATATCTATGCAGGAAAGGGGCCTCTGCATATGCTTCCGCTTTTCGATGAGTCCGCGTCCCATTAGCTTGTTGGCGGGTAACGCCACCAAGGCGACGATGGGTAGCTGGTCTGAGAGGATGACCAGCCACACTGGGACTGGAACACGGCCCA
>FQH3XDB01BUDG9|870|2
ATTGAACGCTGGCGGCGTGCTTAACACATGCAAGTCGAACGCGAAAGTTCCTTCGGGGACGAGTAGAGTGGCGCACGGGTGAGTAACGCGTGGACAATCTGCCTTTCTGACGGGGATAACAGTTGGAAACGACTGCTAATACCGGATACGCTCATGTTGAACTATGTGAGGAAAGACGGCCTCTGTCTGCAAGCTGTCGCAGAAAGATGAGTCCGCGTCCCATTAGCTTGTTGGTGGGGTAACGGCCCACCAAGGCGACGATGGGTAGCCGATTTGAGAGGATGATCGGCCACACTGGAACTGAAACACGGTCCAGACTCCTACGGGAGGCAGCAGTGGGGAATATTGCGCAATGGGCGAAAGCCTGACGCAGCGACGCCGCGTGAGCGAAGAAGTATTTCGGTATGTAAAGCTCTATCAGCAGGGAAGAATAATGACGGTACCTGAC
>FQH3XDB01AMFDR|871|2
ATTGAACGCTGGCGGCGTGCTTAACACATGCAAGTCGAACGCGAAAGTTCCTTCGGGGACGAGTAGAGTGGCGCACGGGTGAGTAACGCGTGGACAATCTGCCTTTCTGACGGGGATAACAGTTGGAAACGACTGCTAATACCGGATACGCTCATGTTGAACTATGTGAGGAAAGACGGCCTCTGTCTGCAAGCTGTCGCAGAAAGATGAGTCCGCGTCCCATTAGCTTGTTGGTGGGGTAACGGCCCACCAAGGCGACGATGGGTAGCCGATTTTGAGAGGATGATCGGCCACACTGGAACTGAAACACGGTCCAGACTCCTACGGGAGGCAGCAGTGGGGAATATTGCGCAATGGGCGAAAGCCTGACGCAGCGACGCCGCGTGAGCGAAGAAGTATTTCGGTATGTAAAGCTCTATCAGCAGGGAAGAATAATGACGGTACCTGACTAAGAAGCACCGGCTAAATACGTGCCAGCAC
>FQH3XDB01ELLOC|872|1
ATTGAACGCTGGCGGCGTGCTTAACACATGCAAGTCGAACGCGAAAGTTCCTTCGGGGACGAGTAGAGTGGCGCACGGGTGAGTAACGCGTGGACAATCTGCCTTTCTGACGGGGATAACCAGTTGGAAACGACTGCCTAATACCGGATACGGCTCATGTTGAACTATGTGAGGAAAGACGGCCTCTGCCTGCAAGCTGTCGCAGAAAGCTGAGTCCGCGTCCCATTAGCTTGTTGGCGGGGTAACGGCCCACCAAGGCGACGATGGGTAGCCGATTTGAGAGGATGATCGGCCACACTGGAACTGAAACA
>FQH3XDB01EGT78|873|1
ATTGAACGCTGGCGGCGTGCTTAACACATGCAAGTCGAGCGGTAACAGGTGTAGCAATACATGCTGACGAGCGGCGAACGGGTGAGTAATGCTTCGGAATCTACCCATTGGCGGGGAATAACTAGCCGAAAGGTTAGCTAATACCGCATAAGCCCTACGGGGGAAAGCAGGGGATCGCAAGACCTTGCACCGATGGATGAGCTGAAGTCGGATTAGCTAGTTGGTAGGGTAACGGCCTACCAAGGCGACGATCCGTAGCTGGTCTGAGAGGACGACCAGCCACACTGGGACTGAGACACGGCCCAGACTCCTACGGGAGGCAGCAGTGGGGAATATTGGACAATGGGGGAAACCCTGATCCAGCGACGCCGCGTGTGTGAAGAAGGCCTGCGGGTTGTAAA
>FQH3XDB01AI2LM|874|1
ATTGAACGCTGGCGGCGTGCTTAACACATGCAAGTCGTACGTGAAAGGGGCTTCGGTCCCGAGTAAAGTGGCGCACGGGTGAGTAACGCGTGGACAATCTACCGGGAAGTGAGGAATAACGGCTGGAAACGGCCGCTAATACCGCATACGTTCTGTGATTTAGTTTATGGAAGAAAGGGGGCCTCTGCATATGCTTCCGCTTTTCGATGAGTCCGCGTCCCATTAGCTAGTTGGTGAGGTAACGGCCCACCAAGGCGACGATGGGTAGCTGTTCTGAGAGGATGATCAGCCACATTGGAACTGGAACACGGTCCAAAACTCCTACGGGAGGCAGCAGTGGGGAATATTGCGCAATGGGCGAAAGCCTGACGCAGCGACGCCGCGTG
>FQH3XDB01AMLYJ|875|1
GATGAACGCTGGCGGCATGCCTAAGACATGCAAGTCGAACGAGATGGCCCATTGACTAAGATTGAAGGTTGAGTGCTTGCACAATTCTGGATTTCTTATGAAGTGGATTTTCCATCGAGTGGCAAACGGGTGAATAACACGTGGGTTACCTACCTCTAAGTTGGGGATAACAGTTGGAAACGACTGATAATACCGAATATGCTCTCCGGAGTAAAGAAGCCCTTAAAGCTTCGCTAAGAGATGGGCCTGCGGCGTATTAGCTAGTGGTGGGTAACGCTACCAAGGCAACGATGCGTAGCCGAACTGAGAGGTTGATCGGCCA
>FQH3XDB01D7J7C|876|1
GATAAACGCTGGCGGCATGCCTAAGACATGCAAGTCGAACGGGACGCCCCATTGAGAACGGAAATAAGTTGGAGAGCTTGCTCAAAGATGGAAATAAGTAGGATTTGGATTCTGCGTCCAGTGGCGAACGGGTGAGTAACACGTGGGTTACCTGCCTTCAAGCTGGGGATAACAGTTAGAAATGATTGCTAATACCGAATGTGCTAGAATAGTAAGAGCCTTTAAAGCTTCACTTGAAGATGGGCCTGCGGCGTATTAGATAGTTGGTGGGTAACGCCTACCAAGTCAACGATGCGTAGCCGGA
>FQH3XDB01C3P3S|877|7
GATAAACGCTGGCGGCATGCCTAAGACATGCAAGTCGAACGGGACGCCCCATTGAGAACGGAAATAAGTTGGAGAGCTTGCTCAAAGATGGAAATAAGTAGGATTTGGATTCTGCGTCCAGTGGCGAACGGGTGAGTAACACGTGGGTTACCTGCCTTCAAGCTGGGGATAACAGTTAGAAATGATTGCTAATACCGAATGTGCTAGAAATAGTAAAGAAGCCTTTAAAGCTTCACTTGAAGATGGGCCTGCGGCGTATTAGATAGTTGGTGGGGTAACGCCTACCAAGTCAACGATGCGTAGCCGGACTGAGAGGTTGA
>FQH3XDB01D0FON|878|1
GATAAACGCTGGCGGCATGCCTAAGACATGCAAGTCGAACGGGACGCCCCATTGAGAACGGAAATAAGTTGGAGAGCTTGCTCAAAGATGGAAATATGTAGGATTTGGATTCTGCGTCCAGTGGCGAACGGGTGAGTAACACGTGGGTTACCTGCCTTCAAGCTGGGGATAACAGTTAGAAATGATTGCTAATACCGAATGTGCTAGAAATAGTAAAGGAGCCTTTAAAGCTTCACTTGAAGATGGGGCCTGGCGGCGTGTTAGATAGTTGGTGGGTAACGCCTACCAGTCGACGATGCGTAGCCGGACT
>FQH3XDB01B0EJ6|879|1
GATAAACGCTGGCGGCATGCCTAAGACATGCAAGTCAAACGGGACGCCCCATTGAGAACGGAAATAAGTTGGAGAGCTTGCTCAAAGATGGAAATATGTAGGATTTGGTTCTGCGTCCAGTGGCGAACGGGTGAGTAACACGTGGGTTACCTGCCTTCAAGCTGGGGATAACAGTTAGAAATGATTGCTAATACCGAATGTGCTAGAAATGTAAAGGAGCCTTTAAAGCTTCACTTGAAGATGGGCCTGCGGCTATTAGATAGTTGGTGGGGTAACGCCTACCAAGTCGACGATGCGTAGCCGGACTGA
>FQH3XDB01CZZ8T|880|2
GATAAACGCTGGCGGCATGCCTAAGACATGCAAGTCAAACGGGACGCCCCATTGAGAACGGAAATAAGTTGGAGAGCTTGCTCAAAGATGGAAATATGTAGGATTTGGTTTCTGCGTCCAGTGGCGAACGGGTGAGTAACACGTGGGTTACCTGCCTTCAAGCTGGGGATAACAGTTAGAAATGATTGCTAATACCGAATGTGCTAGAAATAGTAAAGGAGCCTTTAAAAGCTTCACTTGAAGATGGGCCTGCGGCGTATTAGATAGTTGGTGGGTAACGCCTACCAAGTCGACGATGCGTAGCCGGA
>FQH3XDB01C72QA|881|8
GATGAACGCTGGCGGCATGGCCTAAGACATGCAAGTCGAACGGAGGGGCCCATTGATAGTTAAAGAAAGTGGAGTGCTTGCACAAAACTGGAATTAGTTAGATTTGGAATTTCCCCTCAGTGGCAGACGGGTGAGTAACACGTGGGTAATCTACCTTCGAGACTGGGATAACGTTTAGAAATGAACGCTAATACCGGATAAACCATATTTAGATAACTAAGTATGTTAAAAGGGGCTTCGGCCTCACTTGAAGATGAGCTTGCGGTGTATTAGCTAGTTGGTGAGGTAATGGCTCACCAAGGCAACGATGCCATATCCGAGCTGAGAGGCCTGAACGGACACATTGGGACTGAG
>FQH3XDB01CC8LG|882|2
GATTAACGCTGGCGGCATGCCTAAGACATGCAAGTCGAACGGAGCGCCCCATTGATATCTGATTGAAATTGAAGAGCTTGCTCAGATATGGATTGACGAAGGATTTGGATTCTGCGCTCAGTGGCGAACGGGTGAGTAACACGTGGGTTATCTGCCTCTAAGTTGGGGATAACAGTTAGAAATGACTGCTAATACCGAATGTGCTAGTAATAGTAAAGGAGCCCTTAAAAGCTTCGCTTAGAGATGAGCCTGCGGTGTATTAGCTAGTTGGTGGGGTAATGGCCTACCAAGGCAACGATGCATAGCCGGACTGAGAGGTTCGAAACGGCCACACTGGGACTGAGACACGGCCCAGACTCCTACGGGAGA
>FQH3XDB01BFQDE|883|4
GATTAACGCTGGCGGCATGCCTAAGACATGCAAGTCGAACGGAGCGCCCCATTGATATCTGATTGAAATTGAAGAGCTTGCTCAGATATGGATTGACGAAGGATTTGGATTCTGCGCTCAGTGGCGAACGGGTGAGTAACACGTGGGTTATCTGCCTCTAAGTTGGGGATAACAGTTAGAAATGACTGCTAATACCGAATGTGCTAGTAATAGTAAAGGAGCCCTTAAAAGCTTCGCTTAGAGATGAGCCTGCGGTGTATTAGCTAGTTGGTGGGGTAATGGCCTACCAAGGCAACGATGCATAGCCGGACTGAGAGGTTGAACGGCCACACTGGGACTGAGACACGGCCCAGACTCCTACGGGAGACAGCAGTTAGGAATATCGGCAATGGGACGAAAGCCTGACCGAGCAATGCCGCGTGTGAGATGAAGGTCCT
>FQH3XDB01DIKCS|884|2
GATTAACGCTGGCGGCATGCCTAAGACATGCAAGTCGAACGGGGTATACCATTGATCTCTGAATGAAGTTGAAGAGCTTGCTCAGATATGGAATGACGAAGGATGTGGATTCTATACCTAGTGGCGAACGGGTGAGTAACACGTGGGTTACCTGCCTCTAAGTTGGGGATAACGGTTAGAAATGATCGCTAATACCGAATGTGCTAGTAATAGTAAAGGCGCTTTCAAGCGTCGCTTAGAGATGGGCCTGCGGCGTATTAGCTAGTTGGTGAGGTAACGGCTCACCAAGGCGACGATGCGTAGCCGGACTGAGAGGTTAAACGGCCACATTGGGACTGAGACACGGGCCCAAAACTCCTACGGGAGACAGCAGTTAGGAATATTCGGCAATGGGCGAAAGCCTGACCGAGCAATGCCGCGTGTGAG
>FQH3XDB01BYQSI|885|1
GATGAACGCTGGCGGCATGCCTAAGACATGCAAGTCGAACGAAGGGACCCAATGAGATTTATAGAAGTTTGGAGAGCTTGCTCAAAAAATGGAAATAAATTGATTTGGATTATCCCTTAGTGGCAAACGGGTGAGTAACACGTGGGTTACCTGCCTTCAAGATGGGGATAACAGTTGGAAACGATTGCTAATACCGAATGTGATCTACGGATTAAAGAAGCCTTTAAAAGCTTCGCTTGAAGATGGGCCTGCGGTGCATTAGCTAGTTGGTGAGATAACGGCCCACAAGGCGACGATGCATAGCCGAATGAGA
>FQH3XDB01CNI82|886|6
GATGAACGCTGGCGGCATGCCTAAGACATGCAAGTCGAACGAAGGGACCCAATGAGATTTATAGAAGTTTGGAGAGCTTGCTCAAAGAATGAAAATAAATTGATTTGGATTATCCCTTAGTGGCAAACGGGTGAGTAACACGTGGGTTACCTGCCTTCAAGATGGGGATAACAGTTGGAAACGATTGCTAATACCGAATGTGATCTACGGATTAAAGAAGCCTTTAAAGCTTCGCTTGAAGATGGGCCTGCGGTGCATTAGCTAGTTGGTGAGATAACGGCCCACAAGGCGACGATGCATAGCCGAACTG
>FQH3XDB01D9H6H|887|1
GATGAACGCTGGCGGCATGCCTAAGACATGCAAGTCGAACGAAATGGCCCAGAAATATTTATTGAAGTTTGAAGTGCTTGCACTGATTTCTGATTTATTTGGCTCTGGATTTTCCATTTAGTGGCAAACGGGTGAGTAACACGTGGGTTACCTGCCTCTAAGTTGGGGATAACAGTTGGAAACGACTGATAATACCGAATGTGCTCTACGGAGTAAAGAAGCCCTTAAAGCTTCGCTTAGAGATGGGCCTGCGGCGTATTAGCTAGTTGGTGAGATAACGGCCACCAAGGCGACGATGCGTAGCCGAACTGAAGAGGTTAATCGGCCACACTGGGACTGAGACACGG
>FQH3XDB01D2PQ7|888|2
GATGAACGCTGGCGGCATGCCTAAGACATGCAAGTCGTACGCGAGGGCCCAATGATATTAAAGGAAGTGGAAAGTGCTTGCACTGGAAATGGAATTTAGTTGATTTGGATTTTCCCTCGAGTGGCAAACGGGTGAGTAACACGTGGGTTACCTACCTCTATGTTGGGGATAACAGTTGGAAACGATTGCTAATACCGAATGTGCTCTTCGGAGTAAAGAAGCCTTTAAAAGCTTCGCGTAGAGATGGGCCTGCGGCGCATTAGCTAGTTGGTGGGGTAATGGCCTACCAAGGCAACGATGCGTAGCTGAGCTGAGAGGTTGATCGGCCACACTGGGACTGAGACACGGCCCAGACTCC
>FQH3XDB01CRAEF|889|1
GACGAACGCTGGCGGCGCGCCTAACACATGCAAGTCGAACGGAGTAAATTTTCTCACTGAGTTTTCTGAGACCTGACCAATGAGCGCATTGATGCGAAGCATCAATTAGGCTCATTCAAGTAAGACTAACAGACTGAGAAGACCGAGTGAGGGAAATTTACTTAGTGGCGAACGGGTGAGTAACGCGTGAGGAACCTGCCTCAAAGAGGGGGACAACAGTTGGAAACGACTGCTAATACCGCATAAGCCCACGGACCGGCATCGGTCTGAGGGAAAAGAGAATCCGCTTTGAGATGGCCTCGCGTCCGATTAGCTAGTTGGTGAGGTAACGGCCCACAAGGCGACGATCGGTAGCCGGACTGAGAGGTTGAACGGCCACATTGGGACTGAGACACGGCCC
>FQH3XDB01EK0B2|890|26
GATGAACGCTGGCGGCATGCCTAAGACATGCAAGTCGAACGGGATGGCCCATTGATTACAACTGAAAGCTGGAGTGCTTGCACAAAAGTCGGATGGTGTATGATTTGGATTTTCCATCCAGTGGCAAACGGGTGAGTAACACGTGGGTTACCTACCTCTAAGTTGGGGATAACAGTTGGAAACGATTGCTAATACCGAATGTGCTCTACGGAGTAAAGATGCCTTTAAAGCATCGCTTAGAGATGGGCCTGCGGCGTATTAGTTAGTTGGTGGGGTAATGGCCTACAAGACGACGATGCGTAGCCGAACTGAGAGGTTAATCGGGCCACACTGGGACTGAGACACGGCCCAGGACTCCG
>FQH3XDB01D1R0L|891|36
GATGAACGCTGGCGGCATGCCTAAGACATGCAAGTCGAACGGGATGGCCCATTGATTACAACTGAAAACTGGAGTGCTTGCACAAAAGCTGGATGGTGTATGATTTGGATTTTCCATCCAGTGGCAAACGGGTGAGTAACACGTGGGTTACCTGCCTCTAAGTTGGGGATAACAGTTGGAAACGATTGCTAATACCGAATGTGCTCTACGGAGTAAAGATGCCTTAAAGCATCGCTTAGAGATGGGCCTGCGGCGTATTAGTTAGTTGGTGGGGTAATGGCCTACCAAGACGACGATGCGTAGCCGAACTGAGAGGTTAA
>FQH3XDB01EIVRE|892|1
GATGAACGCTGGCGGCATGCCTAAGACATGCAAGTCGAACGAAGTGGCCCTTTGACTTATATTGAAACTTTGAGTGCTTGCACGATTTGTTGATTTATAATGATTAGGATTTTCCACTTAGTGGCAGACGGGTGAGTAACACGTGGGTAACCTACCTCAGAGACTGGGATAACTATTGGAAACGATAGCTAATACCGGATGATTCATAACTACATAAGTAGATATGCTAAAAGGAGCTTCGGCTTCACTTTGAGATGGGCTTGCGGTGTATTAGCTAGTTGGTGGGTAATGGCCTACAAGGCAACGATGCATATCCGAGCTGAGAGGCTGATCGGACACACTGGAACTGAGATATGGTCCA
>FQH3XDB01CUQIE|893|1
GATGAACGCTGGCGGCATGCCTAAGACATGCAAGTCGAACGAAGTGGCCCTTTGACTTATATTGAAACTTTGAGTGCTTGCACGATTTGTTGATTTATAATGATTAGGATTTTCCACTTAGTGGCAGACGGGTGAGTAACACGTGGGTAACCTACCTCAGAGACTGGGATAACTATTGGAAACGATAGCTAATACCGGATGATTCATAACTACATAAGTAGATATGACTAAAAAGGAGCTTCGGCTTCACTTTGAGATGGGCTTGCGGTGTATTAGCTAGTTGGTGGGTAATGGCCTACCAAGGCAACGATGCATATCCGAGCTGAGAGGCTGATCGGACACACTGGAACTGAGATAC
>FQH3XDB01AM1AG|894|1
GATGAACGCTGGCGGCATGCCTAAGACATGCAAGTCGAACGAGGCGGCCCACTGATTTGAAAGGAAGTTGGAGTGCTTGCACAAAGATGAAATTTTGATGACGTGGAACTTCCGCCTAGTGGCGCAAGGGTGAGTAACACGTGGGTAATCTACCTTAGAGACTGGGATAACAGTTGGAAACGACTGCTAATACCGGATGATATGTAAGAAGATACGTTTTCTTATATTAAAAGGAGTACCTTTAAAAGCTTCACTTTTGAGATGAGCCTGCGGCGTATTAGCTAGTTGGTGGGGTAAAGGCCTACCAAGGCAACGATGCGTAGCCGACTGAGAGGGT
>FQH3XDB01A4ILS|895|2
GATGAACGCTGGCGGCATGCCTAAGACATGCAAGTCGAACGAGGCGGCCCACTGATTTGAAAGGAAGTTGGAGTGCTTGCACAAAGATGAAATTTTGATGAAGTGGAACTTCCGCCTAGTGGCGCAAGGGTGAGTAACACGTGGGTAATCTACCTTAGAGACTGGGATAACAGTTGGAAACGACTGCTAATACCGGATGATATGTAAGAAGATACGTTTTCTTATATTAAAAGGAGCCTTTTAAAAGCTTCACTTTAAGATGAGCCTGCGGCGTATTAGCTAGTTGGTGGGGTAAAGGCCTACCAAGGCAACGATGCGTAGCCGACCTGAGAGGGTGATCGGCCACACTGGGACTGAGACACGGCCCAGACTCCTACGGGAGACAGCAGTTAGGAATATTCGTCAAGTGGGGGAAACCCTGAACGA
>FQH3XDB01CUAOM|896|2
GATGAACGCTGGCGGCATGCCTAAGACATGCAAGTCGAACGAGGCGGCCCACTGATTTGAAAGGAAGTTGGAGTGCTTGCACAAAGATGAAATTTCAATGAAGTGGAACTTCCGCCTAGTGGCGCAAGGGTGAGTAACACGTGGGTAATCTACCTTAGAGACTGGGATAACAGTTGGAAACGACTGCTAATACCGGATGATATGTAAGAAGATACGTTTTCTTATATTAAAAGGAGCCTTTAAAAGCTTCACTTTAAGATGAGCCTGCGGCGTATTAGCTAGTTGGTGGGTAAAGGCCTACCAAGGCAACGATGCGTAGCCGACCTGAGAGGTGATCGGCCACACTGGGACTGAGACA
>FQH3XDB01BLBQD|897|7
GATGAACGCTGGCGGCGTGCTTAACACATGCAAGTCAAAACGGTAGTGGTGTTTAGCACTAAGCGTATTCTGTTAGGATATGTTTAGTGCTGAGCATTACTGCAGTGGCGGACGGGTGAGTAACGCATGAACAACCTGCCTTAGAGAGGGGGATAACACTTAGAAATAGGTGCTAATACCGCATAAGACCACGGTGTCGCATGACACAGGGGTAAAAGAGTAATCCACTTTAAGAGGGGTTCGCGTATCATTAGCTAGTTGGTGAGGTAAAGGCCCACCAAGGCGACGATGGTTAGCCGACCTGAGAGGGTGATCGGGCCA
>FQH3XDB01BNEK8|898|1
GATGAACGCTGGCGGCATGCCTAAGACATGCAAGTCGAACGAAGTGGCCCATTGATGCAGAGTGCTTGCACGAAGTTGATTTGGATTTCCACTTAGTGGCGCAAGGGTGAGTAACACGTGGGTTATCTACCTCAGAGACTGGAATAACAATTGGAAACGATTGCTAATGCCGGATGATATGTAAAACGATACGTTGTTTATATTAAAAGGAGCCTTTAAAAGCTTCACTTTGAGATGAGCCTGCGGCGTATTAGCTAGTTGGTAAGGTATGGCTTACCAAGGCGACGATGCGTAGCC
>FQH3XDB01A705H|899|1
GATGAACGCTGGCGGCATGCCTAAGACATGCAAGTCGAACGAAGTGGCCCATTGATGCGGAGTGCTTGCACAAAGTTGATTTGGATTTCCACTTAGTGGCGCAAGGGTGAGTAACACGTGGGTTATCTACCTCAGAGACTGGAATAACAATTGGAAACGATTGCTAATGCCGGATGATATATAAGGCGATACGTTACCTTATATTAAAAGGAGCCTTTAAAAGCTTCACTTTGAGATGAGCCTGCGGCGTATTAGCTAGTTGGTAAGGTAATGGCTTACCAAGGCAACGATGCGTAGCCGGCCTGAGAGGGTGATCGGCCACATTGGGACTGAGACAC
>FQH3XDB01BD8RY|900|10
GATGAACGCTGGCGGCATGCCTAAGACATGCAAGTCGTACGAAGTGACCCTTTGAAATGGAGTGCTTGCACAAAGTAGATAAGGATTTCACTTAGTGGCGCAAGGGTGAGTAACACGTGGGTAATCTGCCTTTAAGACTGGGATAACAATTGGAAACGATTGCTAATACCGGATAACATATATAATGGTAAACTTTATATATTAAAAGGAGCCTTTAAAGCTTCACTTAAAGATGAGCCTGCGGCGCATTAGCTAGTTGGTGGGTAATGGCCTACCAAGGCAACGATGCGTAGCCGAACTGAGAGGTTGATCGGCCACATT
>FQH3XDB01AXF31|901|2
GATGAACGCTGGCGGCATGCCTAAGACATGCAAGTTGAACGGAGCGAACTTATGAAGATGGAGTGCTTGCACAAAGTTGGAATTTGACCTCGCTTAGTGGCGAAAGGGTGAGTAACACGTGGGAATCTGCCTTTGAGACTGGGACAACAGTTGGAAACGACTGCTAATACCGGATGATATATAAGATGATACGTTATCTTATATTAAAAGAAGCCTTTAAAGCTTCACTTAAAGATGAGCCTGCGGCGTATTAGCTAGTTGGTAAGGTAATGGCTTACCAAGGCAACGATGCGTAGCCGAACTGAGAGGTTGATCGGCCACGATTGGGACTGAGACACGGGCCCAAACTCCTAATGGGAGACAGCAGTTAGGAAT
>FQH3XDB01AHXZM|902|1
GATGAACGCTGGCGGCATGCCTAAGACATGCAAGTCGAACGAAGTGGCCCATTGAAAGTGGAGTGCTTGCACAAAACTGGATATGGGGTTCCCACTTAGTGGCAGACGGGTGAGTAACACGTGGGTAACCTACCCTAAAGACTGGGATAACTATTGGAAACGATAGCTAATACCGGATAATATATATTTAGATAACTAGATATATTAAAAGGAGCGTTTGCTTCACTTTAGGATGGGCTTGCGGTGTATTAGCTAGTTGGTGGGGTAATGGCCTACCAAGGCAACGATGCATATCCGAGCTGAGAGGCTGATCGGACACACTGGGACTGAGACACGGCCCAGACTCCTACGGGAGACAGCAGTTAGGAATATTCGTCAATGGGGAAACCCTGAACGAGCAATGCCGCGTGAGTGATGAAGG
>FQH3XDB01A4LX2|903|3
GATGAACGCTGGCGGCATGCCTAAGACATGCAAGTCGAACGAGGAGACCTAATGACGATTATTGAAACTGGAGTGCTTGCACAAAAGCGGATTTAATCTGATTTAGATCATCTCCTAGTGGCAAACGGGTGAGTAACACGTGGGTTACCTGCCTCTAAGTTGGGGATAACAGTTGGAAACGATTGCTAATACCGAATGTGCTCTACGGAGTAAAGAAGCCCTTAAAGCTTCGCTTAGAGATGGGCCTGCGGCGTATTAGATAGTTGGTGGGGTAATGGCCTACCAAGTCGACGATGCGTAGCCGAACTGAGAGGTTAATCGGCCACACTGGGACTGAGACACGGCCCAGACTCCGTACGGGAGACAGCAGTTAGGAAT
>FQH3XDB01ERYMF|904|10
GATGAACGCTGGCGGCATGCCTAAGACATGCAAGTCGAACGGAGCGGCCCATTGAAATGGAGTGCTTGCACAAAGTGGATTTGGATTCCCGCTTAGTGGCGAAAGGGTGAGTAACACGTGGGTTATCTACCTCAGAGACTGGAATAACAATTGGAAACGATTGCTAATGCCGGATGATATATAAAACGATACGTTGTTTTATATTAAAAAGGAGCCTTTAAAAGCTTCACTTTGAGATGAGCCCGCGGCGTATTAGCTAGTTGGTGAGGTAATGGCCCACCAAGGCAACGATGCGTAGCCGGCCTGAGA
>FQH3XDB01CW1BE|905|1
GATGAACGCTGGCGGCATGCCGTAAGACATGCAAGTCGGACGAGACGGCCCAATGAAAACGGAGTGCTTGCACAAAGTTGGATTTGGTTTCCCGTCTAGTGGCGCAAGGGTGAGTAACACGTGGGTAATCTGCCTTCGAGTCTGGAATAACAGTTAGAAATGATTGCTAATGCCGGATGATATTTAGATAGATACGTCTAACTAAATTAAAAGGAGCCTTTAAAAGCTTCGCTTGAAGATGAGCCTGCGCCGTATTAGCTAGTTGGTGGGGTAAAGCCTACCAAGGCAACGATGCGTAGCCGACTGAG
>FQH3XDB01A9E4D|906|1
GATGAACGCTGGCGGCATGCCTAAGACATGCAAGTCGTACGAAGTGGCCCAATGAGATTTTGTGCTTGCACAAAATTGATTTGGATTCCCACTTAGTGGCGCAAGGGTGAGTAACACGTGGGTTATCTACCTCAGAGACTGGGATAACAATTGGAAACGATTGCTAATACCGGATGATATATAAGATGATACGTTGTCTTATATTAAAAAGGAGCCTTTTAAAAGCTTCACTTTGAGATGAGCCTGCGGCGTATTAGCTAGTTGGTGGGGTAATGGCCTACCAAGGCAACGATGCGTAGCCGAACTGAGAGGTTGATCGGCCACACTGGGACTGAGACACGGCCCAGACTCCTACGGGAGACAGCAGTTAGGAATA
>FQH3XDB01EXA5D|907|2
GATGAACGCTGGCGGCATGCCTAAGACATGCAAGTCGTACGAAGTGGCCCATTGATGATGGAGTGCTTGCACAAGATTTGATTTGGATTCCCACTTAGTGGCGCAAGGGTGAGTAACGCGTAGGTTATCTGCCTTCGAGTTTGGAATAACAATTAGAAATGATTGCTAATGCCGGATTATATTTGAGATGATACGTTGTCTTAAAATTAAAAGGAGCCTTTAAAGCTTCGCTTGAAGATGAGCCTGCGTCGTATTAGCTAGTTGGTAGGGTAATGGCCTACCAAGGCAACGATGCGTAGCCGACC
>FQH3XDB01DJ38W|908|2
GATGAACGCTGGCGGCATGCCTAAGACATGCAAGTCGTACGCGAAGGCCTAATGATTATTGTAGAAGATTGGAGAGCTTGCTCAAAGATTGGAAACAATATGATTTAGATCACCTTCGAGTGGCAAACGGGTGAGTAACACGTGGGTTACCTGCCTCTAGGTTGGGGATAACGGTTGGAAACGATCGATAATACCGAATATACTCTACGGAGCAAAGAAGCCTTTAAAGCTTCGCCAAGAGATGGGCCTGCGGCGTATTAGCTAGTTGGTGGGTAATGGCCTACCAAGGCAACGATACGTAGCCGAACTGAGAGGTTAATCGGGCCACACTGGGACTGAGACACGGCCCAGACTCCTACGGGAGACAGCAGTTAGGAATATTCGTCAATGGGGAAACCCTGAACGAGCAATGCCGCGTGAGTGATGA
>FQH3XDB01DAAZ2|909|1
GATGAACGCTGGCGGCATGCCTAAGACATGCAAGTCGTACGCGAAGGCCTAATGATTATTGTGAAAGATTGGAGAGCTTGCTCAAAGATTGGAAACAATATGATTTAGATCACCTTCGAGTGGCAAACGGGTGAGTAACACGTGGGTTACCTGCCTCTAGGTTGGGGATAACGGTTGGAAACGATCGATAATACCGAATATACTCTAAGGAGCAAAAGAAGCCTTTAAAGCTTCGCCAAGAGATGGGCCTGCGGCGTATTAGCTAGTTGGTGGGTAATGGCCTACCAAGGCAACGATACGTAGCCGAACTGAGAGGTTAATCGGCCACACTGGGACTGAGACACGGCCCAG
>FQH3XDB01DJHK8|910|1
GATGAACGCTGGCGGCATGCCTAAGACATGCAAGTCGAACGAAGCGGCCCGATGACATGGAGTGCTTGCACAAAGTAGATTCGGATTTCCGCTTAGTGGCGCAAGGGTGAGTAACACGTGGGTAATCTACCTCAGAGACTGGGATAACAGTTGGAAACGATTGCTAATACCAGATGATATGTAAGAAGATACGTTTTCTTAAATTAAAAGGGAGCCTTTAAAGCTTCGCTTTGAGATGAGCCTGCGGCGTATTAGCTAGTTGGTAGGGTAATGGCCTACCAAGGCAACGATGCGTAGCCGACTGAGAGGGTGATCGGCCACATTGGGACTGAGACACGGCCCAAACTCCGTAACGGGAGACAGCAGTTA
>FQH3XDB01EDZK2|911|2
GATGAACGCTGGCGGCATGCCTAAGACATGCAAGTCGAACGAAGCGGCCCGATGACATGGAGTGCTTGCACAAAATAGATTCGGATTTCCGCTTAGTGGCGCAAGGGTGAGTAACACGTGGGTAATCTACCTCAGAGACTGGGATAACAGTTGGAAACGATTGCTAATACCAGATGATATGTAAGAAGATACGTTTTCTTAAATTAAAAGGAAGCCTTTAAAGCTTCGCCTTGAGATGAGCCTGCGGCGTATTAGCTAGTTGGTAGGGTAATGGCCTACCAAGGCAACGATGCGTAGCCGACCTGAGAGGTGATCGGCCACATTGGGACTGAGACACGGCCCAAACTCCTACGGGAGACAGCAG
>FQH3XDB01DVFEI|912|1
GATGAACGCTGGCGGCATGCCTAAGACATGCAAGTCGAACGAAGTGGTCCAATGAAGATGGAGTGCTTGCACGAAATCAGATTTGGGTTGCCACTTAGTGGCGAAAGGGTGAGTAACACGTAGGTTATCTACCTTTAAGACTGGAATAACAATTAGAAATGATTGCTAATACCGGATGATATATAGAGTGATACGTCACTTTGATTAAAAGGAGCCCTTTAAAAGCTTCACTTAAAGATGAGCCTGCGACGTATTAGCTAGTTGGTGGGTAAAGGCTTACCAAGGCGACGATGCGTAGCCGACCTGAGAGGTGATCGGCC
>FQH3XDB01D08KC|913|1
GATGAACGCTGGCGGCATGCCTAAGACATGCAAGTCGAACGGAGCAGCCTAATGAAGATTGAGTGCTTGCACAAGATTGGATTTAGTTCTGCTTAGTGGCGAAAGGGTGAGTAACACGTGGGAATCTACCTTCAAGACTGGGACAACAGTTGGAAACGACTGCTAATACCGGATGATATATTAAATGATAAGTTATTTAATAGTAAAGGAGCCCTTTAAAGCTTCACTTGAAGATGAGCCTGCGGCGTATTAGCTAGTTGGTGAGGTAACCTGCTCACCAAGGCAACGATGCGTAGCCGAACTGAGAGGTTGATCGGCCACATTTGGGGACTGAGACACGGCCCAAAATCCTATGGGAGACAGCAGTTAGGAATATTCGTCAATGGGGGAAACCCTGAACGAGCAATGCCGCGTGTGTGATGAAGGT
>FQH3XDB01A1UQU|914|3
GATGAACGCTGGCGGCATGCCTAAGACATGCAAGTCGTACGAAGCGGCCCTTTGAAATGGAGTGCTTGCACAATATGGATAAGGATTCCCGCTTAGTGGCGCAAGGGTGAGTAACACGTGGGTTATCTGCCTCGAAGACTGGGATAACAATTGGAAACGATTGCTAATACCGGATGATATGTAGAATGATACGTTGTTCTATAGTAAAAGGAGCCTTTAAAGCTTCACTTCGAGATGAGCCTGCGGCGCATTAGCTAGTTGGTAAGGTAATGGCTTACCAAGGCAACGATGCGTAGCCGAACTGAGAGGTTGATCGGCCACATTGGGACTGAGACACGGCCCAAAC
>FQH3XDB01BNMO6|915|22
GATGAACGCTGGTGGCATGCCTAAGACATGCAAGTCGTACGAAGTACCCCAAAGAAGTTTTGAGTGCTTGCACAAGAGACGGACTTGGATCAGTACTTAGTGGCAGACGGGTGAGTAACACGTGGGTAATCTACCTCGAAGACTGGGATAACAATTAGAAATGGTTGCTAATACCGGATAATTCGTAATCGGAAAACTGATTATGCTAAAAGGAGCTTCGGCTTCACTTCGAGATGAGCTTGCGGTGTATTAGCTAGTTGGTAGGGCAATGGCCTACCAAGGCAACGATGCATAGCCGAACTGAGAGGTTGATCGGCCACATTGGGACTGAGACAGGCCCAGACTCC
>FQH3XDB01ASGRC|916|1
GATGAACGCTGGCGGCATGCCTAAGACATGCAAGTCGTACGAAGTGGCCCTTTGAAATGGAGTGCTTGCACAAAGTAGATAAGGATTTCCACTTAGTGGCGCAAGGGTGAGTAACACGTGGGTTATCTGCCTTCAAGACTGGGATAACAATTGGAAACGATTGCTAATACCGGATGATATGTAAAATGATACGTTATTTATATTAAAAGGAGCCTTTAAAAGCTTCACTTGAAGATGAGCCTGCGGCGCATTAGCTAGTTGGTAAGGTAATGGCTTACCAAGGCAACGATGCGTAGCCGAACTGAGAGGTTGATCGGCCACATTGGGGACTGAGACACGGCCCAAAACTCCTACGGGAGACAGCAGTTAGGAATATTCGTCAATGGGGAAACCCTGAACGAGCAATGCCGCGTGAATGATGACGGTCCTCTGGATTGTAAAATTCTGTTGTTGGGAAGAA
>FQH3XDB01CQ73R|917|1
GATGAACGCTGGCGGCATGCCTAAGACATGCAAGTCGAACGAAGTGACCCAATGAAGTGGAGTGCTTGCACAAAGCAGATTTGGATTCTCACTTAGTGGCGCAAGGGTGAGTAACACGTGGGTAATCTACCTCAGAGACTGGGATAACAATTGGAAACGATTGCTAATACCGGATGATATATAAGATGATACGTTGTCTTATATTAAAAGGAGCCTTTAAAAGCTTCACTTTGAGATGAGCCTGCGGCGTATTAGCTAGTTGGTAAGGTAATGGCTTACCAAGGCAACGATGCGTAGCCGGCCTGAGAGGGTGATCGGCCACATTGGGACTGAGACACGGCCC
>FQH3XDB01D1NNC|918|2
GATAAACGCTGGCGGCGCACATAAGACATGCAAGTCGAACGGAAGTCGTTGTAATGAAACAGAAATGGACAGAGAACTTGTTCAAAGAAAGTGGAAGTAGACTTACAACAATGGCTTTAGTGGCGGACTGGTGAGTAACGCGTAAGGAACCTGCCTTCTAGAGGGGAATAACAATGAGAAATCATTGCTAATACCGCATATGCCGAGAGAATCACATGATACAATCGGGGAAAAGAGAAATCTGCTAGAAGATGGCCTTGCGTCTGATTAGATAGTTGGTGGGGTAACGGCCTACCAAGTCGACGATCAGTAGCCGGACTGAGAGGTTGAACGGCCACATTGGGGACTGAGATACGGCCCAGACTTCCTACGGGGAGGCAGCAGTTCGGGAATATTGCGCAATGGAGGAAACTCTGACGCAGTT
>FQH3XDB01D5PHW|919|1
GACGAACGCTGGCGGCGTGCTTCAAACATGCAAGTCGAACGGAGAAGTTGTCAGCACAAAGGTATTTTAGGAAGCGTTTGAAATTTTCGGAGAAAATTTCTCCCCGCACCAGCAAGGGAAGCAAACCTTGAGGAAGATACTTTTGTGCTGATAATTTCTTAGTGGCGGACTGGTGAGTAACGCGTGAGTAATCTGCCTATCACAGGGGGATAACAGTTGGAAACGACTGCTAATACCGCATAAGACCACATGATGGCATCATCGAGGGGTCAAAGGGCTACTGGTGATAGATGAGCTTGCGTATCATTAGCTAGTTGGTGAGGTAAGCCCACAGGCGACGATGATTAGCCGATCTGAGAGGATCGACCGGCCACACTGGAACTGAGATACGGTCCAGA
>FQH3XDB01B7S3R|920|1
GACGAACGCTGGCGGCGCGCCTAACACATGCAAGTCGAACGGAGTAAATTTTCTCACTGAGTTTTCTGAGACCTGACCAATGAGCGTCATTGATGCGAAGCATCAATTAGGCTCATTCAAGTAAGACTAACAGACTGAGAAGACCGAGTGAGGAAATTTACTTAGTGGCGAACGGGTGAGTAACGCGTGAGGAACCTGCCTCAAAGAGGGGGACAACAGTTGGAAACGACTGCTAATACCGCATAAGCCCACGGACCGGCATCGGTCTGAGGGAAAAGGATTTTATCCGCTTTTGAGATGGCCTCGCGTCCGATTAGCTAGTTGGTGAGGTAACGGCCCACCAAGGCGACGATCGGTAGCCGGACTGAGAGGTTGAACGGCCACATTGGGACTGAGACACGGCCCAGACTCCTACGGGAGGCAGCAGTGGGGAATAGTTGCACGAAGTGGGGGGAACCCTGATGCAGCGAC
>FQH3XDB01C2YR6|921|8
GACGAACGCTGGCGGCATGCCTAACACATGCAAGTCGAACGGAGATAACCGCTGATTTGACGGTAGCTTGCTACAAGATTTTCTTGGTTATCTTAGTGGCGGACGGGCGAGTAACGCGTGAGTAACCTGCCCTTAAGTGGGGAACAACAGTTGGAAACGACTGCTAATACCGCATAAAGTCATGTAGCCGCATGGTTGTATGACCAAAGGATTTATTCGCTTAAAGGATGGACTCGCGTCCGATTAGCTAGTTGGTGAGGTAACGGCCCACCAAGGCGACGATCGGTAGCCGAACTGAGAGGTTGATCGGCCGCATTGGGACTGAGACACGGCCCAGACTCCTACGGGAGGCAGCAGTGGGGATATTGCACAATGGGGGAAACCCTGATGCAGCAATGCCGCGT
>FQH3XDB01BX0SZ|922|24
GACGAACGCTGGCGGCGCGCCTAACACATGCAAGTCGAACGGAATGAGAGGGAGCTTGCTTCTTCTTGTTTAGTGGCGAACGGGTGAGTAACGCGTGAGGAACCTGCCTCAAAGAGGGGGACAACAGTTGGAAACGACTGCTAATACCGCATAAGCCCACAGACCGGCATCGGTCAGGGGGAAAAGATTTATCCGCTTTGAGATGGCCTCGCGTCCGATTAGCTAGTTGGTGAGGTAACGGCCCACCAAGGCGACGATCGGTAGCCGGACTGAGAGGTTGAACGGCCACATTGGGACTGAGACACGGCCCAGACTCCTACGGGAGGCAGCAGTGGGGAATATTGCACAATGGGGGAACCCTGATGCAGCGACGCCGCGTGGAGGAAGAAGGTC
>FQH3XDB01BYKA6|923|48
GACGAACGCTGGCGGCGCGCCTAACACATGCAAGTCGAACGGAGTTGAGAGGAGCTTGCTTTTCTTGACTTAGTGGCGAACGGGTGAGTAACGCGTGAGGAACCTGCCTCAAAGAGGGGGACAACAGTTGGAAACGACTGCTAATACCGCATAAGCCCACAGGTCGGCATCGACCAGAGGGAAAAGGATTTTTATCCGCTTTGAGATGGCCTCGCGTCCGATTAGCTAGTTGGTGAGGTAATGGCCCACCAAGGCGACGATCGGTAGCCGGACTGAGAGGTTGAACGGCCACATT
>FQH3XDB01DHYKR|924|1
GACAAACGCTGGCGGCGTGCTTAACACATGCAAGTCGAACGAAGCATAGGAGCTTGCTCCTATGACTGAGTGGCGGACGGGTGAGTAACACGTGAGCAACCTGCCCTATACACCGGGATAACACAGAGAAATTTGTGCTAATACCGGATAAGACCACGACGGGGCATCCCGATGCGGTCAAAGCTTTAGCGGTATAGGAGGGGCTCGCGTCCCATTAGTTAGTTGGTGAGGTAACGGCCCACCAAGACAGCGATGGGTAGCCGACCTGAGAGGGTGTACGGCCACACTGGAACTGAGACACGGTCCAGACTCCTACGGGAGGCAGCAGTGGGGAATATTGGGCAAGTGGAGGCAACTCTGACCCAGCAACGCCGCGTGAATGATGAAGGT
>FQH3XDB01CL761|925|61
GACGAACGCTGGCGGCGCGCCTAACACATGCAAGTCGAACGAGCGATGGAGAGCTTGCTCTCCAAAGCGAGTGGCGAACGGGTGAGTAACGCGTGAGGAACCTGCCTCAAAGAGGGGGACAACAGTTGGAAACGACTGCTAATACCGCATAAGCCCACGGCTCGGCATCGAGCAGAGGGAAAAGGAGCAATCCGCTTTGAGATGGCCTCGCGTCCGATTAGCTAGTTGGTGAGGTAACGGCCACCAAGGCGACGATCGGTAGCCGGACTGAGAGGTTGAACGGCCACATTGGGACTGAGACACGGCCCAGACTCCTACGGGAGGCAGCAGGTGGGGAATATTGCACGAAGTGGGGGAAAACCCTGATGCAGCGACGCCGCG
>FQH3XDB01B0VKV|926|41
GACGAACGCTGGCGGCGCGCCTAACACATGCAAGTCGAACGAGCGATGGAGAGCTTGCTCTCCAAAGCGAGTGGCGAACGGGTGAGTAACGCGTGAGGAACCTGCCTCAAAGAGGGGGACAACAGTTGGAAACGACTGCTAATACCGCATAAGCCCACAGCTCGGCATCGAGCAGAGGGAAAAGAGCAATCCGCTTTGAGATGGCCTCGCGTCCGATTAGCTAGTTGGTGAGGTAACGGCCCACCAAGGCGACGATCGGTAGCCGGACTGAGAGGTTGAACGGCCACATTGGGACTGAGACACGGGCCCCAGGACTCCTACGGGAGCAGCAGTGGGGAATATT
>FQH3XDB01C5U0J|927|99
GACGAACGCTGGCGGCGCGCCTAACACATGCAAGTCGAACGAGCGATGGAGAGCTTGCTTTCCAAAGCGAGTGGCGAACGGGTGAGTAACGCGTGAGGAACCTGCCTCAAAGAGGGGGACAACAGTTGGAAACGACTGCTAATACCGCATAAGCCCACGGGTCGGCATCGACCAGAGGGAAAAGGAGCAATCCGCTTTGAGATGGCCTCGCGTCCGATTAGCTAGTTGGTGAGGTAACGGCCCACCAAGGCGACGATCGGTAGCCGGACTGAGAGGTTGAACGGCCACATTGGGACTGAGACACGGCCCCAGACTCCTACGGGAGGCAGCAGTGGGGAATATTGCACAATGGGGGAAACCCTGATGCAGCGACGCCGCGTGGAGG
>FQH3XDB01BFKPJ|928|1
GACGAACGCTGGCGGCACGCTTAACACATGCAAGTCGAACGGTGAAGAGAAGCTTGCTTCTTGGATCAGTGGCGGACGGGTGAGTAACACGTGAGCAACCTGCCTCAAAGAGGGGGACAACAGTTGGAAACGACTGCTAATACCGCATAAGCCCCACAGCTCGGCATCGAGCAGAGGGAAAAGAGTAATCCGCTTTGAGATGGCCTCGCGTCCGATTAGCTAGTTGGTGAGGTAACGGCCACAAGGCGACGATCGGTAGCCGGACTGAGAGGTTGAACGGCCACAGTTGGGACTGAGACACGGCCCAGACCT
>FQH3XDB01BOAQX|929|8
GACGAACGCTGGCGGCACGCTTAACACATGCAAGTCGAACGGAGTTAATTGGAGCTTGCTCTGATTAACTTAGTGGCGGACGGGTGAGTAACACGTGAGCAACCTGCCTTTAAGAGAGGAATAACAGTCTGAAAAGACTGCTAATACCTCATAAGCCCACGACCCGGCATCGGGGTTGAGGGAAAAGGAGCGATCCGCTTTGAGATGGCCTCGCGTCCGATTAGCTAGTTGGTGAGGTAACGGCCCACCAAGGCGACGATCGGTAGCCGGACTGAGAGGTTGAACGGCCACATTGGGACTGAGACACGGCCCAGACTCCTACGGGAGGCAGCAGTGGGGAATATTGCACAAGTGGGGGAAACCCTGATGCAGCGACGCCGCGTGGAGGAAGAAGGTC
>FQH3XDB01BV9V6|930|1
GACGAACGCTGGCGGCACGCTTAACACATGCAAGTCGAACGGAGTTTACTGAAGCTTGCTTTGGTAAACTTAGTGGCGGACGGGTGAGTAACACGTGAGCAACCTGCCTTTAAGAGGGGGACAACAGTTGGAAACGACTGCTAATACCGCATAAGCCCACGGCTCGGCATCGAGCAGAGGGAAAAGGAGCAATCCGCTTTGAGATGGCCTCGCGTCCGATTAGCTAGTTGGTGAGGTAATGGCCCACCAAGGCGACGATCGGTAGCCGGACTGAGAGGTTGAACGGCCACATTGGGACTGAGACACGGCCCAGACTCCTACGGGAGGCAGCAGGTGGGGAATATTGCACAATGGGGAAACCCTGATGCAGCGACGCCGC
>FQH3XDB01C1W37|931|1
GACGAACGCTGGCGGCATGCCTAACACATGCAAGTCGAACGGAAAGTAACGCTGAAACGATGATAGCTTGCTATAGGAATTTCTTGTTGCTTTTAGTGGCGGACGGGCGAGTAACGCGTGAGTAACCTGCCCTTCAGTGGGGAACAACAGTTGGAAACGACTGCTAATACCGCATAATGTCATTTTCCCGCATGAGAGAATGACCAAAGGATTTATTCGCTGAAGGATGGACTCGCGTCCGATTAGCTAGTTGGTGGGGTAACGGCCCACCAAGGCGACGATCGGTAGCCGAACTGAGAGGTTGATCGGCCGCATTGGGACTGAGACACGGCCCCAGACTCCTACGGGAGGCAGCAGTGGGGGATATTGCACAAGTGGGGGAAACCCTGATGCAG
>FQH3XDB01B2TR1|932|30
GACGAACGCTGGCGGCGCGCCTAACACATGCAAGTCGAACGAGCGATGGAGAGCTTGCTTTCCAGAGCGAGTGGCGAACGGGTGAGTAACGCGTGAGGAACCTGCCTCAAAGAGGGGGACAACAGTTGGAAACGACTGCTAATACCGCATAAGCCCACGGGTCGGCATCGACCAGAGGGAAAAGGAGTGATCCGCTTTGAGATGGCCTCGCGTCCGATTAGCTAGTTGGTGAGGTAACGGCCACCAAGGCGACGATCGGTAGCCGGACTGAGAGGTTGAACGGCCACATTGGGACTGAGACACGGCCCAGACTCCTACGGGAGGCAGCAGTGGGGAATATTGCACAATGGGGAAACCCTGATGCAGCGACGCCGCGTGGAGGAA
>FQH3XDB01BLUFB|933|22
GACGAACGCTGGCGGCGCGCCTAACACATGCAAGTCGAACGGAGTTAAGGAGAGCTTGCTTTTCTTAACTTAGTGGCGAACGGGTGAGTAACGCGTGAGGAACCTGCCTCAAAGAGGGGGACAACAGTTGGAAACGACTGCTAATACCGCATAAGGCCCACAGGTCGGCATCGACCAGAGGGAAAAGGAGAAATCCGCTTTGAGATGGCCTCGCGTCCGATTAGCTGGTTGGTGAGGTAACGGCCCACCAAGGCGACGATCGGTAGCCGGACTGAGAGGTTGAACGGCCACATTGGGACTGAGACACGGCCCAGACTCCTAC
>FQH3XDB01A7EQV|934|37
GACGAACGCTGGCGGCGCGCCTAACACATGCAAGTCGAACGAGCGATGGAGAGCTTGCTTTCCAGAGCGAGTGGCGAACGGGTGAGTAACGCGTGAGGAACCTGCCTCAAAGAGGGGGACAACAGTTGGAAACGACTGCTAATACCGCATAAGCCCACGGGTCGGCATCGATCTGAGGGAAAAGAGCAATCCGCTTTGAGATGGCCTCGCGTCCGATTAGCTAGTTGGTGAGGTAACGGCCCACCAAGGCGACGATCGGTAGCCGGACTGAGAGGTTGAACGGCCACATTGGGACTGAGACACGGCCCAGACTCCTACGGGA
>FQH3XDB01A7VR2|935|3
GACGAACGCTGGCGGCGCGCCTAACACATGCAAGTCGAACGAGAGAGAGGGAGCTTGCTTCCTCGATCGAGTGGCGAACGGGTGAGTAACGCGTGAGGAACCTGCCTCAAAGAGGGGGACAACAGTTGGAAACGACTGCTAATACCGCATAAGCCCACGGCTCGGCATCGAGCAGAGGGAAAAGGAGTGATCCGCTTTGAGATGGCCTCGCGTCCGATTAGCTGGTTGGTGAGGTAATGGCCCACCAAGGCGACGATCGGTAGCCGGACTGAGAGGTTGAACGGCCACATTGGGACTGAGACACGGCCCAGGACTCCTACGGGAGGCAGCAGTGGGGAATATTGGCACAATGGGGGAAACCCTGATGCAGCGACGCCGCGTGGAGGAGAAGGTCTTCGGATGTAAACT
>FQH3XDB01ESQCQ|936|3
GACGAACGCTGGCGGCGCGCCTAACACATGCAAGTCGAACGAAATGAGAGGGAGCTTGCTTCTTCTTATTTAGTGGCGAACGGGTGAGTAACGCGTGAGGAACCTGCCTCAAAGAGGGGGACAACAGTTGGAAACGACTGCTAATACCGCATAAGCCCACGGACCGGCATCGGTCTGAGGGAAAAGGAGAGATCCGCTTTGAGATGGCCTCGCGTCCGATTAGCTAGTTGGTGAGGTAACGGCCCACCAAGGCGACGATCGGTAGCCGGACTGAGAGGTTGAACGGCCACATTGGGACTGAGACACGGCCCAGACTCCTACGGGAGGCAGCAGTGGGGAATATTGCACAATGGGGGAACCCTGATGCAGCGACGCCGCGTGGAGGAAGAAGGTCTTCGGA
>FQH3XDB01APUFP|937|4
GACGAACGCTGGCGGCGCGCCTAACACATGCAAGTCGAACGAGAAAGGAAGAGCTTGCTCTTCTGATCGAGTGGCGAACGGGTGAGTAACGCGTGAGGAACCTGCCTCAAAGAGGGGGACAACAGTTGGAAACGACTGCTAATACCGCATAAGCCCACGGGCCGGCATCGGTCTGAGGGAAAAGGAGCAATCCGCTTTGAGATGGCCTCGCGTCCGATTAGCTGGTTGGTGAGGTAACGGCCACCAAGGCGACGATCGGTAGCCGGACTGAGAGGTTGAACGGCCACATTGGGACTGAGACACGGCCCAGACTCCTACGGGAGGCAGCAGTGGGGAATATTGCACAAGTGGGGGAAACCCTGATGCAGCGACGCCGCGTGGAGGAAGAAGGTCTTCGGATTGTAACTCTGTGTTGAGGAAGAT
>FQH3XDB01BWATB|938|1
GACGAACGCTGGCGGCGCGCCTAACACATGCAAGTCGAACGAGAGAGAGGAAGCTTGCTCCCTCAATCGAGTGGCGAACGGGTGAGTAACGCGTGAGGAACCTGCCTCAAAGAGGGGGACAACAGTTGGAAACGACTGCTAATACCGCATAAGCCCACGGCTCGGCATCGAGCAGAGGGAAAAGGAGCAATCCGCTTTGAGATGGCCTCGCGTCCGATTAGCTAGTTGGTGAGGTAATGGCCCACCAAGGCGACGATCGGTAGCCGGACTGAGAGGTTGAACGGCCACATTGGGACTGAGACACGGCCCAGACTCCTACGGGAGGCAGCAGTGGGGAATATTGCACAATGGGGGAAACCCTGATGCAGCGACGCCGCGTGGAGGAAGAAGGTCTTCGGATTGTAAACTCCTGTTGTTGGGGAAGATAATGACGGTACCCAACAAGGAAGTGACGGCTAACTACGTGCCAGCAG
>FQH3XDB01EA1CY|939|2
GACGAACGCTGGCGGCGCGCCTAACACATGCAAGTCGAACGAGAGAGAGGGAGCTTGCTTCCTCGATCGAGTGGCGAACGGGTGAGTAACGCGTGAGGAACCTGCCTCAAAGAGGGGGACAACAGTTGGAAACGACTGCTAATACCGCATAAGCCCACGGCTCGGCATCGAGCAGAGGGAAAAGGAGTGATCCGCTTTGAGATGGCCTCGCGTCCGATTAGCTGGTTGGTGAGGGTAATGGCCCACCAAGGCGACGATCGGTAGCCGGACTGAGAGGTTGAACGGCCACATTGGGACTGAGACACGGCCCAGACTCCGTACGGGAGGCAGCA
>FQH3XDB01EV9T0|940|28
GACGAACGCTGGCGGCGCGCCTAACACATGCAAGTCGAACGAGAGAGGAAGAGCTTGCTCTTCTAATCGAGTGGCGAACGGGTGAGTAACGCGTGAGGAACCTGCCTCAAAGAGGGGGACAACAGTTGGAAACGACTGCTAATACCGCATAAGCCCACGGTTCGGCATCGAGCTGAGGGAAAAGAGCAATCCGCTTTGAGATGGCCTCGCGTCCGATTAGCTAGTTGGTGAGGTAACGGCCCACCAAGGCGACGATCGGTAGCCGGACTGAGAGGTTGAACGGCCACATTGGGACTGAGACACGGCCCCAGACTCCTACGGGAGGCAGCAGTGGGGAATATTGCACAATGGGGGAAACCCTGATGCAGCGACGCCGCGTGGAGGAAGAAGGTCTTCGGGATTGGTAAACTCCTGTTGTTGAGGAAGATAATGACGGTACTCAAC
>FQH3XDB01B2D6I|941|11
GACGAACGCTGGCGGCGCGCCTAACACATGCAAGTCGAACGAGAGAGAGGGAGCTTGCTTCCTCAATCGAGTGGCGAACGGGTGAGTAACGCGTGAGGAACCTGCCTCAAAGAGGGGGACAACAGTTGGAAACGACTGCTAATACCGCATAAGCCCACGGCCCGGCATCGGGTTGAGGGAAAAGGAGCAATCCGCTTTGAGATGGCCTCGCGTCCGATTAGCTGGTTGGTGAGGTAATGGCCCACCAAGGCGACGATCGGTAGCCGGACTGAGAGGTTGAACGGCCACATTGGGACTGAGACACGGCCCAGACTCCTACGGGAGGCAGCAGTGGGGAATATTGGCACAATGGGGGAAACCCTGATGCAGCGACGCCGCGTGGAGGAAGAAGGTCTTCGGATTG
>FQH3XDB01A0254|942|28
GACGAACGCTGGCGGCGCGCCTAACACATGCAAGTCGAACGAGAGACAGAGAGCTTGCTTTCTGGATCGAGTGGCGAACGGGTGAGTAACGCGTGAGGAACCTGCCTCAAAGAGGGGGACAACAGTTGGAAACGACTGCTAATACCGCATAAGCCCACGACCCGGCATCGGGTTGAGGGAAAAGGAGCAATCCGCTTTGAGATGGCCTCGCGTCCGATTAGCTAGTTGGTGAGGTAATGGGCCCACCAAGGCGACGATCGGTAGCCGGACTGAGAGGTTGAACGGCCACATTGGGACTGAGACACGGCCCAGACTCCTACGGGAGGCAGCAGTGGGGAATATTGCACAATGGGGGAAACCCTGATGCAGCGACGCCGCGTGGAGGAAGAAGGTCTTCGGATTGTAAACTCCTGTTGTTGAGGAAGATAATGACGGTACTCAACAAGGAAGTGACGGCTAACTACGTGCCAGCAGCC
>FQH3XDB01CI9ZN|943|6
GACGAACGCTGGCGGCGCGCCTAACACATGCAAGTCGAACGAGAGACAGAGAGCTTGCTCTTTGAATCGAGTGGCGAACGGGTGAGTAACGCGTGAGGAACCTGCCTCAAAGAGGGGGACAACAGTTGGAAACGACTGCTAATACCGCATAAGCCCCACGACCCGGCATCGGGGTTGAGGGAAAAGGAGCAATCCGCTTTGAGATGGCCTCGCGTCCGATTAGCTAGTTGGTGAGGTAACGGCCCACCAAGGCGACGATCGGTAGCCGGACTGAGAGGTTGAACGGCCACATTGGGACTGAGACACGGCCCAGACTCCTACGGGAGGCAGCAGTGGGGAATATTGCACAATGGGGAAACCCTGATGCAGCGACGCCGCGTGGAGGAAGAAGGTCTTCGGATTGTAAA
>FQH3XDB01DTDVW|944|22
GACGAACGCTGGCGGCGCGCCTAACACATGCAAGTCGAACGAGAGACGGAGAGCTTGCTCTCTGAATCGAGTGGCGAACGGGTGAGTAACGCGTGAGGAACCTGCCTCAAAGAGGGGGACAACAGTTGGAAACGACTGCTAATACCGCATAAGCCCACGACCCGGCATCGGGTTGAGGGAAAAGGAGCAATCCGCTTTGAGATGGCCTCGCGTCCGATTAGCTAGTTGGTGAGGTAACGGCCCACCAAGGCGACGATCGGTAGCCGGACTGAGAGGTTGAACGGCCACATTGGGACTGAGACACGGCCCAGACTCCTACGGGAGGCAGCAGTGGGGAATATTGCACAATGGGGGAAACCCTGATGCAGCGACGCCGCGTGGAGGAAGAAGGTCTTCGGATTGAAACTCTGTTGTTGAGGAAGATAAT
>FQH3XDB01DNBJB|945|10
GACGAACGCTGGCGGCGCGCCTAACACATGCAAGTCGAACGAGAGACAGAGAGCTTGCTTTCTGAATCGAGTGGCGAACGGGTGAGTAACGCGTGAGGAACCTGCCTCAAAGAGGGGGACAACAGTTGGAAACGACTGCTAATACCGCATAAGCCCACAGCTCGGCATCGAGCAGAGGGAAAAGGAGCAATCCGCTTTGAGATGGCCTCGCGTCCGATTAGCTGGTTGGTGAGGTAACGGCCCACCAAGGCGACGATCGGTAGCCGGACTGAGAGGTTGAACGGCCACATTGGGACTGAGACACGGCCCAGACTCCTACGGGAGCAGCAGTGGGGAATATTGCACGAATGGGG
>FQH3XDB01ELJTR|946|4
GACGAACGCTGGCGGCGCGCCTAACACATGCAAGTCGAACGAGAGAGAAGGAGCTTGCTTCTTCGATCGAGTGGCGAACGGGTGAGTAACGCGTGAGGAACCTGCCTCAAAGAGGGGGACAACAGTTGGAAACGACTGCTAATACCGCATAAGCCCACGGCTCGGCATCGAGCTGAGGGAAAAGGAGCAATCCGCTTTGAGATGGCCTCGCGTCCGATTAGCTAGTTGGTGAGGTAACGGCCCACCAAGGCGACGATCGGTAGCCGGACTGAGAGGTTGAACGGCCACATTGGGACTGAGACACGGCCCAGACTCCTACGGGAGGCAGCAGTGGGGAATATTGCACAAGTGGGGAAACCCTGATGCAGCGACGCCG
>FQH3XDB01B6X5I|947|1
GACGAACGCTGGCGGCGCGCCTAACACATGCAAGTCGAACGAGAGAGGAAGAGCTTGCTCTTCTAATCGAGTGGCGAACGGGTGAGTAACGCGTGAGGAACCTGCCTCAAAGAGGGGGACAACAGTTGGAAACGACTGCTAATACCGCATAAGCCCACGGGATCGCATGATTCTGAGGGAAAAGGAGTGATCCGCTTTGAGATGGCCTCGCGTCCGATTAGCTAGTTGGTGAGGTAACGGCCACCAAGGCGACGATCGGTAGCCGGACTGAGAGGTTGAACGGCCA
>FQH3XDB01BNA8Z|948|6
GACGAACGCTGGCGGCGCGCCTAACACATGCAAGTCGAACGAGAGAGGAGAAGCTTGCTTCTCCAATCGAGTGGCGAACGGGTGAGTAACGCGTGAGGAACCTGCCTCAAAGAGGGGGACAACAGTTGGAAACGACTGCTAATACCGCATAAGCCCACGGCTCGGCACCGAGCAGAGGGAAAAGGAGCAATCCGCTTTGAGATGGCCTCGCGTCCGATTAGCTAGTTGGTGAGGTAATGGGCCCACCAAGGCGACGATCGGTAGCCGGACTGAGAGGTTGAACGGCCACATTGGGACTGAGACACGGCCCAGACTCCTACGGGAGGCAGCAGTGGGGAATATTGCACAATGGGGGAAACCTGATGCAGCGACGCCGCGTGGAGGAAGAAGGTC
>FQH3XDB01DQSXV|949|73
GACGAACGCTGGCGGCGCGCCTAACACATGCAAGTCGAACGAGAGAGAGGGAGCTTGCTTCCTTGATCGAGTGGCGAACGGGTGAGTAACGCGTGAGGAACCTGCCTCAAAGAGGGGGACAACAGTTGGAAACGACTGCTAATACCGCATAAGCCCACGACCCGGCATCGGGTTGAGGGAAAAGGAGCAATCCGCTTTGAGATGGCCTCGCGTCCGATTAGCTAGTTGGTGAGGTAATGGCCCACCAAGGCGACGATCGGTAGCCGGACTGAGAGGTTGAACGGCCACATTGGGACTGAGACACGGCCCCAGACTCCTACGGGAGGCAGCAGTGGGGAATATTGCACAATGGGGGAAACCCTGATGCAGCGACGCCGCGTGGAGGAAGAAGGTCTTCGGGATTGTAAAACTCCTGTTGTTGAGGAAGA
>FQH3XDB01CQXSD|950|8
GACGAACGCTGGCGGCGCGCCTAACACATGCAAGTCGAACGAGAGAGAAGGAGCTTGCTTCTTCAATCGAGTGGCGAACGGGTGAGTAACGCGTGAGGAACCTGCCTCAAAGAGGGGGACAACAGTTGGAAACGACTGCTAATACCGCATAAGCCCACGGACCGGCATCGGTCTGAGGGAAAAGGAGCAATCCGCTTTGAGATGGCCTCGCGTCCGATTAGCTAGTTGGTGAGGTAACGGCCACCAAGGCGACGATCGGTAGCCGGACTGAGAGGTTGAACGGCCACATTGGGACTGAGACACGGGCCCAGACCTCCTACGGGAGGCAGCAGTGGGGAATATTGCACAAAGTGGGGGGAAAACCCTGATGCAGCGACGCCGCGTGGAGG
>FQH3XDB01DAKT5|951|2
GACGAACGCTGGCGGCGCGCCTAACACATGCAAGTCGAACGAGAGAGAGGGAGCTTGCTTCCTCAATCGAGTGGCGAACGGGTGAGTAACGCGTGAGGAACCTGCCTCAAAGAGGGGGACAACAGTTGGAAACGACTGCTAATACCGCATAAGCCCCACGGTACCGCATGGTACAGAGGGAAAAGGAGTGATCCGCTTTGAGATGGCCTCGCGTCCGATTAGCTGGTTGGTGAGGTAACGGCCCACCAAGGCGACGATCGGTAGCCGGACTGAGAGGTTGAACGGCCACATTGGGACTGAGACACGGCCCAGACTCCTACGGGAGGCAGCAGTGGGGAATATTGCACAAGTGGGGGAACCCTGATGCAGCGACGCC
>FQH3XDB01EYNNO|952|9
GACGAACGCTGGCGGCGCGCCTAACACATGCAAGTCGAACGAGAGAGAGGGAGCTTGCTTCCTTGATCGAGTGGCGAACGGGTGAGTAACGCGTGAGGAACCTGCCTCAAAGAGGGGGACAACAGTTGGAAACGACTGCTAATACCGCATAAGCCCACAGCTCGGCATCGAGCAGAGGGAAAAGGAGCAATCCGCTTTGAGATGGCCTCGCGTCCGATTAGCTAGTTGGTGAGGTAACGGCCCACCAAGGCGACGATCGGTAGCCGGACTGAGAGGTTGAACGGCCACATTGGGACTGAGACACGGCCCAGACTCCTACGGGAGGCAGCAGTGGGGAATAGTTGCACGAATGGGGGAAACCCTGATGCAGCGACGCCGCGT
>FQH3XDB01EDQ6U|953|1
GACGAACGCTGGCGGCGCGCCTAACACATGCAAGTCGAACGAGAGAGAGGGAGCTTGCTCCCTTGATCGAGTGGCGAACGGGTGAGTAACGCGTGAGGAACCTGCCTCAAAGAGGGGGACAACAGTTGGAAACGACTGCTAATACCGCATAAGCCCACAGGTCGGCATCGACCAGAGGGAACAGGAGTGATCCGCTTTGAGATGGCCTCGCGTCCGATTAGCTAGTTGGTGAGGTAACGGCCCACCAAGGCGACGATCGGTAGCCGGACTGAGAGGTTGAACGGCCACATTGGGACTGAGACACGGCCCAGACTCCTACGGGAGGCCAGCAGTGGGGAATATT
>FQH3XDB01A0ATY|954|6
GACGAACGCTGGCGGCGCGCCTAACACATGCAAGTCGAACGAGAGAGGAAGAGCTTGCTCTTCTAATCGAGTGGCGAACGGGTGAGTAACGCGTGAGGAACCTGCCTCAAAGAGGGGGACAACAGTTGGAAACGACTGCTAATACCGCATAAGCCCACGACCCGGCATCGGGTTGAGGGAAAAGGAGTAATCCGCTTTGAGATGGCCTCGCGTCCGATTAGCTAGTTGGTGAGGTAACGGCCCACCAAGGCGACGATCGGTAGCCGGACTGAGAGGTTGAACGGCCACATTGGGACTGAGACACGGCCCAGACTCCTACGGGAGCAGCAGTGGGAATATG
>FQH3XDB01ERKQ1|955|2
GACGAGCGCTGGCGGCGCGCCTAACACATGCAAGTCGAACGAGAGAGGAAGAGCTTGCTCTTCTAATCGAGTGGCGAACGGGTGAGTAACGCGTGAGGAACCTGCCTCAAAGAGGGGGACAACAGTTGGAAACGACTGCTAATACCGCATAAGCCCACGGGATCGCATGATTCTGAAGGAAAAGGAGCAATCCGCTTTGAGATGGCCTCGCGTCCGATTAGCTAGTTGGTGAGGTAATGGCCCACCAAGGCGACGATCGGTAGCCGGACTGAGAGGTTGAACGGCCACATTGGGACTGAGACACGGCCCAGACTCCTACGGGAGGCAGCAGTGGGGAATATTGCACAATGGGGAAACCCTGATGCAGCGACGCCGCGTGGAGGAAGAAGGTCTTCGGGATTGTAAAACTCCTGTTGTTGAGGAAGATAATGACGGTACTCAACAAGGAAGTGACGGCT
>FQH3XDB01D87XR|956|18
GACGAACGCTGGCGGCGCGCCTAACACATGCAAGTCGAACGAGAGAGGAAGAGCTTGCTCTTCTAATCGAGTGGCGAACGGGTGAGTAACGCGTGAGGAACCTGCCTCAAAGAGGGGGACAACAGTTGGAAACGACTGCTAATACCGCATAAGCCCACGGTTCGGCATCGAGCTGAGGGAAAAGAGCAATCCGCTTTGAGATGGCCTCGCGTCCGATTAGCTAGTTGGTGAGGTAACGGCCCACCAAGGCGACGATCGGTAGCCGGACTGAGAGGTTGAACGGCCACATTGGGACTGAGACACGGCCCAGACTCCTACGGGAGCAGCAGTGGGGAATATTGCACAATGGACGGAAGTCTGACCGAGCAACGCCGCGTGAATGATGAAGTA
>FQH3XDB01AJDC6|957|3
GACGAACGCTGGCGGCGCGCCTAACACATGCAAGTCGAACGAGAGAGGAAGAGCTTGCTCTTCTGATCGAGTGGCGAACGGGTGAGTAACGCGTGAGGAACCTGCCTCAAAGAGGGGGACAACAGTTGGAAACGACTGCTAATACCGCATAAGCCCACGGACCGGCATCGGTTTGAGGGAAAAGGAGCAATCCGCTTTGAGATGGCCTCGCGTCCGATTAGCTAGTTGGTGAGGTAACGGGCCCGACCCAAGGCGACGATCGGTAGCCGGACTGAGAGGTTGAACGGCCACATTGGGACTGAGACACGGCCCAGACTCCTAC
>FQH3XDB01D37DG|958|10
GACGAACGCTGGCGGCGCGCCTAACACATGCAAGTCGAACGAGCGAGGAGGAGCTTGCTTCTCCGAGCGAGTGGCGAACGGGTGAGTAACGCGTGAGGAACCTGCCTCAAAGAGGGGGACAACAGTTGGAAACGACTGCTAATACCGCATAAGCCCACGACCCGGCATCGGGTTGAGGGAAAAGGAGCAATCCGCTTTGAGATGGCCTCGCGTCCGATTAGCTAGTTGGTGAGGTAACGGCCCACCAAGGCGACGATCGGTAGCCGGACTGAGAGGTTGAACGGCCACATTGGGACTGAGACACGGCCCAGACTCCTACGGGAGGCAGCAGTGGGGAATATTGCACAATGGGGGAAACCCTGATGCAGCGACGCCGCGTGGAGGAAGAAGGTCTTCGGATTGTAAACTCTGTTGTTGGGAAGATAATGACGGTACCCAACAAGGAAGTGACGGCTAACTACGTGCCAGCAACCGCGGTAATCGTATGACGTCG
>FQH3XDB01AWWTC|959|11
GACGAACGCTGGCGGCGCGCCTAACACATGCAAGTCGAACGAGAGAGGAGAAGCTTGCTTTTCCAATCGAGTGGCGAACGGGTGAGTAACGCGTGAGGAACCTGCCTCAAAGAGGGGGACAACAGTTGGAAACGACTGCTAATACCGCATAAGCCCACGGACCGGCATCGGTCTGAGGGAAAAGGAGCAATCCGCTTTGAGATGGCCTCGCGTCCGATTAGCTAGTTGGTGAGGTAACGGCCCACCAAGGCGACGATCGGTAGCCGGACTGAGAGGTTGAACGGCCACATTGGGACTGAGACACGGGCCCAGACTCCTACGGGAGGCAGCAGTGGGGAATATTGCACAATGGGGGAAACCCTGATGCAGCGACGCCGCGTGGAGGAA
>FQH3XDB01CS7EF|960|6
GACGAACGCTGGCGGCGCGCCTAACACATGCAAGTCGAACGAGAGAGGAGAAGCTTGCTTCTCCAATCGAGTGGCGAACGGGTGAGTAACGCGTGAGGAACCTGCCTCAAGGAGGGGGACAACAGTTGGAAACGACTGCTAATACCGCATAAGCCCACGGAACCGCATGGTTCAGAGGGAAAAGGAGCAATCCGCTTTGAGATGGCCTCGCGTCCGATTAGCTAGTTGGTGAGGTAACGGCCCACCAAGGCGACGATCGGTAGCCGGACTGAGAGGTTGAACGGCCACATTGGGACTGAGACACGGCCCAGACTCCTACGGGAGGCAGCAGTGGGGAATATTGCACAATGGGGAAACCCTGACTGCAGCGACGCCGCGTGGAGGAGAAGGTC
>FQH3XDB01A5ZI5|961|2
GACGAACGCTGGCGGCGCGCCTAACACATGCAAGTCGAGCGAGAGAGGAAGAGCTTGCTCTTCTAATCGAGTGGCGAACGGGTGAGTAACGCGTGAGGAACCTGCCTCAAAGAGGGGACAACAGTTGGAAACGACTGCTAATACCGCATAAGCCCACGGGTCGGCATCGACCAGAGGGAAAAGGAGTGATCCGCTTTGAGATGGCCTCGCGTCCGATTAGCTAGTTGGTGAGGTAACGGCCCACCAAGGCGACGATCGGTAGCCGGACTGAGAGGTTGAACGGCCACATTGGGACTGAGACACGGCCCAGACTCCTACGGGAGGCAGCAGTGGGGAATATTGCACAATGGGGAAACCCTGATGCAGCGACGCCGCGTGGAGGAAGAAGGTCTTCGGATTGTAAACTCCTGTTGTTGAGAAGATAATGAACGGTACTCAACAAGGAAGTGACGGCTAACTACGTGCCAGCA
>FQH3XDB01BONVK|962|24
GACGAACGCTGGCGGCGCGCCTAACACATGCAAGTCGAACGAGAGATGAGGAGCTTGCTCTTCAAATCGAGTGGCGAACGGGTGAGTAACGCGTGAGGAACCTGCCTCAAAGAGGGGGACAACAGTTGGAAACGACTGCTAATACCGCATAAGCCCACAGCTCGGCATCGAGCAGAGGGAAAAGAGCAATCCGCTTTGAGATGGCCTCGCGTCCGATTAGCTAGTTGGTGAGGTAACGGCCCACCAAGGCGACGATCGGTAGCCGGACTGAGAGGTTGAACGGCCACATTGGGACTGAGACACGGCCCAGACTCCTACGGGAGGCAGCAGTGGGGAATACTTGC
>FQH3XDB01BWAXE|963|2
GACGAACGCTGGCGGCGCGCCTAACACATGCAAGTCGAACGAGAGACAGAGAGCTTGCTCTTTGAATCGAGTGGCGAACGGGTGAGTAACGCGTGAGGAACCTGCCTCAAAGAGGGGGACAACAGTTGGAAACGACTGCTAATACCGCATAAGCCCACGGATTGGCATCAATCTGAGGGAAAAGGAGCAATCCGCTTTGAGATGGCCTCGCGTCCGATTAGCTAGTTGGTGAGGTAACGGCCCACCAAGGCGACGATCGGTAGCCGGACTGAGAGGTTGAACGGCCACATTGGGACTGAGACACGGCCCAGACTCC
>FQH3XDB01EZNZX|964|10
GACGAACGCTGGCGGCGCGCCTAACACATGCAAGTCGAACGAGAGATGAGGAGCTTGCTCTTCAAATCGAGTGGCGAACGGGTGAGTAACGCGTGAGGAACCTGCCTCAAAGAGGGGGACAACAGTTGGAAACGACTGCTAATACCGCATAAGCCCACGGGTCGGCATCGACCTGAGGGAAAAGGAGCGATCCGCTTTGAGATGGCCTCGCGTCCGATTAGCTAGTTGGTGAGGTAACGGCCCACCAAGGCGACGATCGGTAGCCGGACTGAGAGGTTGAACGGCCACATTGGGACTGAGACACGGCCCAGACTCCTACGGGAGGCAGCAGTGGGGAATATTGCACAAGTGGGGAAACCCTGATGCAGCGACG
>FQH3XDB01EGA8A|965|7
GACGAACGCTGGCGGCGCGCCTAACACATGCAAGTCGAACGAGAGACAGAGAGCTTGCTTTCTGAATCGAGTGGCGAACGGGTGAGTAACGCGTGAGGAACCTGCCTCAAAGAGGGGGACAACAGTTGGAAACGACTGCTAATACCGCATAAGCCCACGGCTCGGCATCGAGCAGAGGGAAAAGGAGCAATCCGCTTTGAGATGGCCTCGCGTCCGATTAGCTAGTTGGTGAGGTAACGGCCCACCAAGGCGACGATCGGTAGCCGGACTGAGAGGTTGAACGGCCACATTGGGACTGAGACACGGCCCAGACTCCTACGGGAGGCAGCAGTGGGGAATATTGCACAAGTGGGGAAACCCTGATGCAGCGACG
>FQH3XDB01CDYRQ|966|3
GACGAACGCTGGCGGCGCGCCTAACACATGCAAGTCGAACGAGAGACAGAGAGCTTGCTCTCTGAATCGAGTGGCGAACGGGTGAGTAACGCGTGAGGAACCTGCCTCAAAGAGGGGGACAACAGTTGGAAACGACTGCTAATACCGCATAAGCCCACGGGTCGGCATCGATCTGAGGGAAAAGGAGCAAATCCGCTTTGAGATGGCCTCGCGTCCGATTAGCTAGTTGGTGAGGTAATGGCCCACCAAGGCGACGATCGGTAGCCGGACTGAGAGGTTGAACGGCCACATTGGGACTGAGACACAGCCCCAGACTCCTACGGGA
>FQH3XDB01DO363|967|1
GACGAACGCTGGCGGCGCGCCTAACACATGCAAGTCGAACGAGAGACAGAGAGCTTGCTCTCTGAATCGAGTGGCGAACGGGTGAGTAACGCGTGAGGAACCTGCCTCAAAGAGGGGGACAACAGTTGGAAACGGTCGCTAATACCGCATAACACATTTTGAGGGCATCCTTGAAATGTCAAAGGAGCAATCCGCTGAAAGATGAGCTCGCGTCTGATTAGATAGTTGGTGAGGTAACGGCCCACAAGTCTGCGATCAGTAGCCGGACTGAGAGGTTGAACGGCCACATTGGGACTGAGACACGGCCCAGACTCCTACGGGAGCAGCAGTGGGAAT
>FQH3XDB01BBF72|968|5
GACGAACGCTGGCGGCGCGCCTAACACATGCAAGTCGAACGAGAGAGGAGAAGCTTGCTTCTCCAATCGAGTGGCGAACGGGTGAGTAACGCGTGAGGAACCTGCCTCAAAGAGGGGGACAACAGTTGGAAACGACTGCTAATACCGCATAAGCCCACAGCTCGGCATCGAGCAGAGGGAAAAGGAGTAATCCGCTTTGAGATGGCCTCGCGTCCGATTAGCTAGTTGGTGAGGTAATGGCCCACCAAGGCGACGATCGGTAGCCGGACTGAGAGGTTGAACGGCCACATTGGGACTGAGACACGGCCC
>FQH3XDB01ALXHM|969|1
GACGAACGCTGGCGGCGCGCCTAACACATGCAAGTCGAACGAGAGATGAGGAGCTTGCTCTTCAAATCGAGTGGCGAACGGGTGAGTAACGCGTGAGGAACCTGCCTCAAAGGAGGGGGACAACAGTTGGAAACGACTGCTAATACCGCATAAGCCCACGACCCGGCATCGGGTTGAGGGAAAAGGAGCAATCCGCTTTGAGATGGCCTCGCGTCCGATTAGCTAGTTGGTGAGGTAACGGCCACCAAGGCGACGATCGGTAGCCGGACTGAGAGGTTGAACGGCCACATTGGGACTGAGACACGGCCCAGACTCCGTACGGGAGGCAGCAGTGGGGAATATTGCACGAAGTGGGGGAAACCCT
>FQH3XDB01EL8PJ|970|76
GACGAACGCTGGCGGCGCGCCTAACACATGCAAGTCGAACGAGCGAGAGAGAGCTTGCTTTCTCGAGCGAGTGGCGAACGGGTGAGTAACGCGTGAGGAACCTGCCTCAAAGAGGGGGACAACAGTTGGAAACGACTGCTAATACCGCATAAGCCCACGGGTCGGCATCGATCTGAGGGAAAAGGAGCAATCCGCTTTGAGATGGCCTCGCGTCCGATTAGCTGGTTGGTGAGGTAACGGCCCACCAAGGCGACGATCGGTAGCCGGACTGAGAGGTTGAACGGCCACATTGGGACTGAGACACGGCCCCAGACTCCTACGGGAGGCAGCAGTGGGGAATATTGCACAATGGGGGAAACCCTGATGCAGCGACGCCGCGTGG
>FQH3XDB01EZ266|971|7
GACGAACGCTGGCGGCGCGCCTAACACATGCAAGTCGAACGAGCGACAGAGAGCTTGCTCTCTGAAGCGAGTGGCGAACGGGTGAGTAACGCGTGAGGAACCTGCCTCAAAGAGGGGGACAACAGTTGGAAACGACTGCTAATACCGCATAAGCCCACAGGTCGGCATCGACCAGAGGGAAAAGGAGCAATCCGCTTTGAGATGGCCTCGCGTCCGATTAGCTAGTTGGTGAGGTAACGGCCACCAAGGCGACGATCGGTAGCCGGACTGAGAGGTTGAACGGCCACATTGGGACTGAGACACGGCCCAGACTCCTACGGGAGCAGCAGTGGGGAATATTGCACAATGGGGAAACCCTGATGCAGCGACGCC
>FQH3XDB01BT427|972|1
GACGAACGCTGGCGGCGCGCCTAACACATGCAAGTCGAACGAGCGAAGAGGAGCTTGCTTCTCTGAGCGAGTGGCGAACGGGTGAGTAACGCGTGAGGAACCTGCCTCAAAGAGGGGGACAACAGTTGGAAACGACTGCTAATACCGCATAAGCCCACGGATCGGCATCGATCTGAGGGAAAGGGAGCAATCCGCTTTGAGATGGCCTCGCGTCCGATTAGCTGGTTGGTGAGGTAACGGCCCACCAAGGCGACGATCGGTAGCCGGACTGAGAGGTTGAACGGCCACATTGGGACTGAGACACGGCCCAGACCTCCGTACGGGAGGCAGCAGGTGGGAATAGTTGCACAAGTCGGGGAAACCTGATGCAGCGAC
>FQH3XDB01CB1XU|973|2
GACGAACGCTGGCGGCGCGCCTAACACATGCAAGTCGAACGAGTGACAGAGAGCTTGCTCTCTGAAGCGAGTGGCGAACGGGTGAGTAACGCGTGAGGAACCTGCCTCAAAGAGGGGGACAACAGTTGGAAACGACTGCTAATACCGCATAAGCCCACAGCTCGGCATCGAGCAGAGGGAAAAGAGCAATCCGCTTTGAGATGGCCTCGCGTCCGATTAGCTAGTTGGTGAGGTACGCCACAGGCGACGATCGGTAGCCGGACT
>FQH3XDB01B9Z30|974|56
GACGAACGCTGGCGGCGCGCCTAACACATGCAAGTCGAACGAGTGACAGAGAGCTTGCTCTCTGAAGCGAGTGGCGAACGGGTGAGTAACGCGTGAGGAACCTGCCTCAAAGAGGGGGACAACAGTTGGAAACGACTGCTAATACCGCATAAGCCCACAGCTCGGCATCGAGCAGAGGGAAAAGGAGCAATCCGCTTTGAGATGGCCTCGCGTCCGATTAGCTAGTTGGTGAGGTAACGGCCCACCAAGGCAACGATCGGTAGCCGGACTGAGAGGTTGAACGGCCACATTGGGACTGAGACACGGCCCAGACTCCTACGGGAGCAGCAGTGGGGAATATTGCACAATGGGGGAAACCCTGATGCAGCGACGCCGCGTGGAGGAAGAAGGTCTTCGGATTGTAAACTCCTGTTGTTGAGGAAGATAATGACGGTACTCAACAAGGAAGTGAACGGCTAAACTCGTGCCAGCAG
>FQH3XDB01EFQRG|975|6
GACGAACGCTGGCGGCGCGCCTAACACATGCAAGTCGAACGAGCGACAGAGAGCTTGCTCTCTGAAGCGAGTGGCGAACGGGTGAGTAACGCGTGAGGAACCTGCCTCAAAGAGGGGGACAACAGTTGGAAACGACTGCTAATACCGCATAAGCCCACGGCTCGGCATCGAGCAGAGGGAAAAGGAGCAATCCGCTTTGAGATGGCCTCGCGTCCGATTAGCTAGTTGGTGAGGTAATGGCCCACCAAGGCAACGATCGGTAGCCGGACTGAGAGGTTGAACGGCCACATTGGGACTGAGACACGGCCCAGACTCCTACGGGAGGCAGCAGTGGGGAATATTGCACAATGGGGAAACCCTGATGCAGCGACGCCGCGTGGAGGAAGAAGGTCTTCGGATTGTAAACTCCTGTTGTTGGGAAGATAATGACGGTACCAACAAGGAAGTGACGGCTAACTACGTGCCAGCAG
>FQH3XDB01BDQ95|976|1
GACGAACGCTGGCGGCGCGCCTAACACATGCAAGTCGAACGAGCGACAGAGAGCTTGCTCTCTGAAGCGAGTGGCGAACGGGTGAGTAACGCGTGAGGAACCTGCCTCAAAGGAGGGGGGACAACAGTTGGAAACGACTGCTAATACCGCATAAGCCCACAGCTCGGCATCGAGCAGAGGGAAAGAGCAATCCGCTTTGAGATGGCCTCGCGTCCGATTAGCTAGTTGGTGAGGTAACGGCCCACCAAGGCGACGATCCGGTAGCCGGATGAGAGGTTGAACGGCCATATTGGGACTGAGACACGGCCCAGACTCCACGGGAGCAGCAGTGGGAA
>FQH3XDB01C595D|977|5
GACGAACGCTGGCGGCGCGCCTAACACATGCAAGTCGAACGAGCGACAGAGAGCTTGCTCTTTGAAGCGAGTGGCGAACGGGTGAGTAACGCGTGAGGAACCTGCCTCAAAGAGGGGGACAACAGTTGGAAACGACTGCTAATACCGCATAAGCCCACGGGTCGGCATCGATCTGAGGGAAAAGGAGTAATCCGCTTTGAGATGGCCTCGCGTCCGATTAGCTAGTTGGTGAGGTAATGGGCCCACCAAGGCGACGATCGGTAGCCGGACTGAGAGGTTGAACGGCCACATTGGGACTGAGACACGGCCCCAGACTCCTACGGGAGGCAGCAGTGGGGAATATTGCACAATGGGGGAAACCCTGATGCAGCGACGCCGCGTGGAGGAAGAAGGTCTTCGGGATTGTAAACTCCTGTTGTTGAGGAAGATAATGACGGTACTCAACAAGGAAGTG
>FQH3XDB01BT9E9|978|14
GACGAACGCTGGCGGCGCGCCTAACACATGCAAGTCGAACGAGCGAGAGAAAGCTTGCTTTCTTGAGCGAGTGGCGAACGGGTGAGTAACGCGTGAGGAACCTGCCTCAAAGAGGGGGACAACAGTTGGAAACGACTGCTAATACCGCATAAGCCCACAGGTCGGCATCGACCAGAGGGAAAAGGAGTGATCCGCTTTGAGATGGCCTCGCGTCCGATTAGCTAGTTGGTGAGGTAACGGCCCACCAAGGCGACGATCGGTAGCCGGACTGAGAGGTTGAACGGCCACATTGGGACTGAGACACGGCCCCAGACTCCTACGGGAGCAGCAGTGGGGAATATTGCACAATGGGG
>FQH3XDB01A0SFP|979|27
GACGAACGCTGGCGGCGCGCCTAACACATGCAAGTCGAACGAGCGAGAGAGAGCTTGCTTTCTTGAGCGAGTGGCGAACGGGTGAGTAACGCGTGAGGAACCTGCCTCAAAGAGGGGGACAACAGTTGGAAACGACTGCTAATACCGCATAAGCCCACGGGTCGGCATCGACCAGAGGGAAAAGGAGCAATCCGCTTTGAGATGGCCTCGCGTCCGATTAGCTAGTTGGTGAGGTAACGGCCCACCAAGGCGACGATCGGTAGCCGGACTGAGAGGTTGAACGGCCACATTGGGACTGAGACACGGCCCCAGACTCCTACGGGAGGCAGCAGTGGGGAATATTGGCACAATGGGGGAAACCCTGATGCAGCGACGCCGCGTGGAGGAA
>FQH3XDB01EGXGZ|980|1
GACGAACGCTGGCGGCGCGCCTAACACATGCAAGTCGAACGAGCGAGAGAGAGCTTGCTTTCTCGAGCGAGTGGCGAACGGGTGAGTAACGCGTGAGGAACCTGCCTCAAAGAGGGGGACAACAGTCGGAAACGACTGCTAATACCGCATAAAGGCCCCACGGATCGGCATCGATCTGAGGGAAAAGGAGCAATCCGCTTTGAGATGGCCTACGCGTCCGATTAGCTGGTTGGTGAGGTAACGGCCCACCAAGGCGACGATCGGTAGCCGGACTGAGAGGTTGAACGGCCACATTGGGACTGAGACACGGCCCCAGACTCCTACGGGGAGGCAACAGTGGGGAATATTGCACAATGGGGGAAACCCTGATGGCAGCGACGCCGCGTGGAGGAAGAAGGTCTTCGGATTGTAAACTCCTGTTGTTGAGGAAGAT
>FQH3XDB01CAQQM|981|1
GACGAACGCTGGCGGCGCGCCTAACACATGCAAGTCGAACGAGCGAGAGAGAGCTTGCTTTCTCGAGCGAGTGGCGAACGGGTGAGTAACGCGTGAGGAACCTGCCTCAAAGAGGGGGACAACAGTTGGAAACGACTGCTAATACCGCATAAGCCCACGGTACCGCATGGTACAGGGGGAAAAGGAGCAATCCGCTTTGAGATGGCCTCGCGTCCGATTAGCTAGTTGGTGAGGTAACGGCCCACCAAGGCGACGATCGGTAGCCGGACTGAGAGGTTGAACGGCCACATTGGGACTGAGACACGGCCCAGACTCCTACGGGAGGCAGCAGTGGGGAATATTGCACAATGGGGGAAACCCTGATGCAGCGACGCCGCGTGGAGGAAGAAGGTCTTCGGATTGGTAAAACTCCTGTTGTTGAGGAAGA
>FQH3XDB01EWVW7|982|6
GACGAACGCTGGCGGCGCGCCTAACACATGCAAGTCGAACGAGCGAGAGAGAGCTTGCTTTCTTGAGCGAGTGGCGAACGGGTGAGTAACGCGTGAGGAACCTGCCTCAAAGAGGGGGACAACAGTTGGAAACGACTGCTAATACCGCATAAGCCCACGGATCGGCATCGATCTGAGGGAAAAGGAGCGATCCGCTTTGAGATGGCCTCGCGTCCGATTAGCTGGTTGGTGAGGTAATGGCCCACCGAAGGCGACGATCGGTAGCCGGACTGAGAGGTTGAACGGCCACATTGGGACTGAGACACGG
>FQH3XDB01CEURU|983|14
GACGAACGCTGGCGGCGCGCCTAACACATGCAAGTCGAACGAGCGATGGGGAGCTTGCTCTCCAAAGCGAGTGGCGAACGGGTGAGTAACGCGTGAGGAACCTGCCTCAAAGAGGGGGACAACAGTTGGAAACGACTGCTAATACCGCATAAGCCCACGGGTCGGCATCGATCTGAGGGAAAAGAGCAATCCGCTTTGAGATGGCCTCGCGTCCGATTAGCTAGTTGGTGAGGTAATGGCCCACCAAGGCGACGATCGGTAGCCGGACTGAGAGGTTGAACGGCCACATTGGGACTGAGACACGGCCCAGACTCCTACGGGAGCAGCAGTGGGGAATATTGCACAATGGGGGAAACCCTGATGCAGCGACGCCGCGTGG
>FQH3XDB01BVEL4|984|2
GACGAACGCTGGCGGCGCGCCTAACACATGCAAGTCGAACGAGCGATGGGGAGCTTGCTCTCCAAAGCGAGTGGCGAACGGGTGAGTAACGCGTGAGGAACCTGCCTCAAAGAGGGGGACAACAGTTGGAAACGACTGCTAATACCGCATAAGCCCACGGGTCGGCATCGATCTGAGGGAAAGGAGCAATCCGCTTTGAGATGGCCTCGCGCCCGATTAGCTAGTTGGTGAGGTAACGCCACCAGGCGACGATCGGTAGCCGGACT
>FQH3XDB01EUSCA|985|28
GACGAACGCTGGCGGCGCGCCTAACACATGCAAGTCGAACGAGCGATGGAGAGCTTGCTTTCCAGAGCGAGTGGCGAACGGGTGAGTAACGCGTGAGGAACCTGCCTCAAAGAGGGGGACAACAGTTGGAAACGACTGCTAATACCGCATAAGCCCACGGGTCGGCATCGATCTGAGGGAAAAGAGCAATCCGCTTTGAGATGGCCTCGCGTCCGATTAGCTGGTTGGTGAGGTAATGGCCCACCAAGGCGACGATCGGTAGCCGGACTGAGAGGTTGAACGGCCACATTGGGACTGAGACACGGCCCA
>FQH3XDB01DYLVK|986|3
GACGAACGCTGGCGGCGCGCCTAACACATGCAAGTCGAACGAGCGATGGAGAGCTTGCTTTCCAGAGCGAGTGGCGAACGGGTGAGTAACGCGTGAGGAACCTGCCTCAAAGAGGGGACAACAGTTGGAAACGACTGCTAATACCGCATAAGCCCACAGTACCGCATGGTACAGAGGGAAAAGGAGCAATCCGCTTTGAGATGGCCTCGCGTCCGATTAGCTAGTTGGTGAGGTAATGGCCCACAAGGCAACGATCGGTAGCCGGACTGAGAGGTTGAACGGCCACATTGGGACTGAGACACGGCCCAGACTCCTACGGGAGCAGCAGTGGGATATTGCACAATGGGGAAACCCTGATGCAGCGACGCCGCGTGGAGGAAGAAGGTC
>FQH3XDB01EJZ4P|987|1
GACGAACGCTGGCGGCGCGCCTAACACATGCAAGTCGAACGAGCGATGGAGAGCTTGCTTTCCAGAGCGAGTGGCGAACGGGTGAGTAACGCGTGAGGAACCTGCCTCAAAGAGGGGACGAACAGTTGGAACGACTGCTAATACCGCATAAGCCACGGTCGGCATCGATCTGAGGGAAAGGAGCAATCCGCTTGAGATGGCCTCGCGTCCGATTAGCTAGTTGGTGAGGTAACGGCCACCAAGGCGACGATCGGTAGCCGGACTGAGAGGTTGAACGGCCACATTGGGACTGAGACACGGCCCAGACTCCTACGGGAGGCAGCAGTGGGGAATATTGCACAATGGGGGAAACCCTGATGCAGCGACGCCGCGTGGAG
>FQH3XDB01D0MBT|988|1
GACGAACGCTGGCGGCGCGCCTAACACATGCAAGTCGAACGAGCGATGGAGAGCTTGCTTTCCAGAGCGAGTGGCGAACGGGTGAGTAACGCGTGAGGAACCTGCCTCAAAGAGGGGGACAACAGTTGGAAACGACTGCTAATACCGCATAAGCCCACGGCCCGGCATCGGGTTGAGGGAAAAGGAGCAATCCGCTTTGAGATGGCCTCGCGTCCGATTAGCTGGTTGGTGAGGTAATGGCCCACCAAGCGACGATCGGTAGCCGGACTGAGAGGTTGAACGGCCACATTGGGACTGAGACACGGCCCAGACTCCTACGGGAGCAGCAGTGGGGAATATTGCACAATGGGGAAACCTGATGCAGCGACGCCG
>FQH3XDB01CDB06|989|9
GACGAACGCTGGCGGCGCGCCTAACACATGCAAGTCGAACGAGCGATGGAGAGCTTGCTTTCCAGAGCGAGTGGCGAACGGGTGAGTAACGCGTGAGGAACCTGCCTCAAAGAGGGGGACAACAGTTGGAAACGACTGCTAATACCGCATAAGCCCACGGGTCGGCATCGATCTGAGGGAAAAGGAGCGATCCGCTTTGAGATGGCCTCGCGTCCGATTAGCTGGTTGGTGAGGTAACGGCCACCAAGGCGACGATCGGTAGCCGGACTGAGAGGTTGAACGGCCACATTGGGACTGAGACACGGCCCAGACTCCTACGGG
>FQH3XDB01EYYJW|990|6
GACGAACGCTGGCGGCGCGCCTAACACATGCAAGTCGAACGAGCGATGGGGAGCTTGCTCTCCAAAGCGAGTGGCGAACGGGTGAGTAACGCGTGAGGAACCTGCCTCAAAGAGGGGGACAACAGTTGGAAACGACTGCTAATACCGCATAAGCCCACAGCTCGGCATCGAGCAGAGGGAAAAGAGCAATCCGCTTTGAGATGGCCTCGCGTCCGATTAGCTAGTTGGTGAGGTAATGGCCCACCAAGGCGACGATCGGTAGCCGGACTGAGAGGTTGAACGGCCACATTGGGACTGAGACACGGCCCAGACTCCTACGGGAGGCAGCAGTGGGGAATATTGCACAATGGGGGAAAACCCTGATGCAGCGACGCCGCGTGGAGGAAGAAGGTCTTCGGATTGAAACT
>FQH3XDB01CGKXJ|991|5
GACGAACGCTGGCGGCGCGCCTAACACATGCAAGTCGAACGAGCGATGGGGAGCTTGCTCTCCAAAGCGAGTGGCGAACGGGTGAGTAACGCGTGAGGAACCTGCCTCAAAGAGGGGGACAACAGTTGGAAACGACTGCTAATACCGCATAAGCCCACGGGTCGGCATCGACCAGAGGGAAAAGGAGTGATCCGCTTTGAGATGGCCTCGCGTCCGATTAGCTGGTTGGTAGACGGTAACGGCCCACCAAGGCGACGATCGGTAGCCGGACTGAGAGGTTGAACGGCCACATTGGGGACTGA
>FQH3XDB01AFQEG|992|6
GACGAACGCTGGCGGCGCGCCTAACACATGCAAGTCGAACGAGCGATGGGGAGCTTGCTTTCCAGAGCGAGTGGCGAACGGGTGAGTAACGCGTGAGGAACCTGCCTCAAAGAGGGGGACAACAGTTGGAAACGACTGCTAATACCGCATAAGCCCACGACCTGGCATCAGGTTGAGGGAAAAGGAGCAATCCGCTTTGAGATGGCCTCGCGTCCGATTAGCTAGTTGGTGAGGTAATGGGCCCACCAAGGCGACGATCGGTAGCCGGACTGAGAGGTTGAACGGCCACATTGGGACTGAGACACGGCCCCAGACTCCTACGGGAGGCAGCAGTGGGGAATATTGCACAATGGGGGAAACCCTGATGCAGCGACGCCGCGTGGAGGAAGAGGTCTTCGGATTGTAACTCCTGTTGTGAGGAAGATAATGACGGTACTCAACAAGGAAGTGACGGCTAACTACGTGCCCAGCAGTCCGCGGTAA
>FQH3XDB01CZBHS|993|16
GACGAACGCTGGCGGCGCGCCTAACACATGCAAGTCGAACGAGCGACAGAGAGCTTGCTCTCTGAAGCGAGTGGCGAACGGGTGAGTAACGCGTGAGGAACCTGCCTCAAAGAGGGGGACAACAGTTGGAAACGACTGCTAATACCGCATAAGCCCACAGCTCGGCATCGAGCAGAGGGAAAAGAGCAATCCGCTTTGAGATGGCCTCGCGTCCGATTAGCTAGTTGGTGAGGTAACGGCCCACCAAGGCGACGATCGGTAGCCGGACTGAGAGGTTGAACGGCCACATTGGGACTGAGACA
>FQH3XDB01BDAOY|994|2
GACGAACGCTGGCGGCGCGCCTAACACATGCAAGTCGAACGAGCGAGGAGGAGCTTGCTTCTCCGAGCGAGTGGCGAACGGGTGAGTAACGCGTGAGGAACCTGCCTCAAAGAGGGGGACAACAGTTGGAAACGACTGCTAATACCGCATAAGCCCACGGGTCGGCATCGATCTGAGGGAAAAGGAGTGATCCGCTTTGAGATGGCCTCGCGTCCGATTAGCTAGTTGGTGAGGTAATGGCCCACCAAGGCGACGATCGGTAGCCGGACTGAGAGGTTGAACGGCCACATTGGGACTGAGACACGGCCCAGACTCCTACGGGAGGCAGCAGTGGGAATATTGCACAATGGGGGAAACCCTGATGCAGCGACGCCGCGTGGAGGGAAGGAAAGGGTACGTTCC
>FQH3XDB01CUESQ|995|1
GACGAACGCTGGCGGCGCGCCTAACACATGCAAGTCGAACGAGCGAGGAGAAGCTTGCTTCTCTGAGCGAGTGGCGAACGGGTGAGTAACGCGTGAGGAACCTGCCTCAAAGAGGGGGACAACAGTTGGAAACGACTGCTAATACCGCATAAGCCCACGGATCCGCATGGATTTGAGGGAAAAGGAGCAATCCGCTTTGAGATGGCCTCGCGTCCGATTAGCTAGTTGGTGAGGTAACGGCCCACCAAGGCGACGATCGGTAGCCGGACTGAGAGGTTGAACGGCCACATTGGGACTGAGACACGGCCCAGACTCCTACGGGAGGCAGCAGTGGGGAATATTGCACAATGGGGGAACCCTGATGCAGCGACGCCGCGTGGAGGAAGAAGGTCTTCGGATTGTAAACTCCTGTTG
>FQH3XDB01AZPHV|996|5
GACGAACGCTGGCGGCGCGCCTAACACATGCAAGTCGAACGAGTGATGAAGAGCTTGCTCTTCAAAGCGAGTGGCGAACGGGTGAGTAACGCGTGAGGAACCTGCCTCAAAGAGGGGGACAACAGTTGGAAACGACTGCTAATACCGCATAAGCCCACGGGTCGGCATCGACCTGAGGGAAAGGAGCAATCCGCTTTGAGATGGCCTCGCGTCCGATTAGCTAGTTGGTGAGGTAACGGCCACCAAGGCGACGATCGGTAGCCGGACTGAGAGGTTGAACGGCCACATTGGGACTGAGACACGGCCCAGACTCCGTACGGGAGC
>FQH3XDB01B8EWX|997|11
GACGAACGCTGGCGGCGCGCCTAACACATGCAAGTCGAACGAGCGATGAGGAGCTTGCTTCTCAAAGCGAGTGGCGAACGGGTGAGTAACGCGTGAGGAACCTGCCTCAAAGAGGGGGACAACAGTTGGAAACGACTGCTAATACCGCATAAGCCCACGGGTCGGCATCGACCAGAGGGAAAAGGAGCAATCCGCTTTGAGATGGCCTCGCGTCCGATTAGCTAGTTGGTGAGGTAACGGCCCACCAAGGCGACGATCGGTAGCCGGACTGAGAGGTTGAACGGCCACATTGGGACTGAGACACGGCCCAGACTCCTACGGGAGGCAGCAGTGGGGAATATTGCACAATGGGGAAACCCTGATGCAGCGACGCCGCGTGGAGGAAGAAGGTCTTCGGATTGTAAACTCCTGTTGTTGGGGAAGATAATGACGGTACCAACAAGGAAGTGACGGCTAACTACGTGCCAGCAGGCCGCGGTAATTACGA
>FQH3XDB01CZOKT|998|10
GACGAACGCTGGCGGCGCGCCTAACACATGCAAGTCGAACGAGCGAAGAGGAGCTTGCTTCTCTGAGCGAGTGGCGAACGGGTGAGTAACGCGTGAGGAACCTGCCTCAAAGAGGGGGACAACAGTTGGAAACGACTGCTAATACCGCATAAGCCCACGACCCGGCATCGGGTTGAGGGAAAAGGAGCAATCCGCTTTGAGATGGCCTCGCGTCCGATTAGCTGGTTGGTGAGGTAACGGCCCACCAAGGCGACGATCGGTAGCCGGACTGAGAGGTTGAACGGCCACATTGGGACTGAGACACGGCCCAGACTCCTACGGGAGGCAGCAGTGGGGAATATTGCACAATGGGGGAAACCCTGATGCAGCGACGCCGCGTGGAGGAAGAAGGTCTTCGGGATTGTAAAACTCCTGTTGTT
>FQH3XDB01ETNFP|999|110
GACGAACGCTGGCGGCGCGCCTAACACATGCAAGTCGAACGAGCGAAGAGGAGCTTGCTTCTCTGAGCGAGTGGCGAACGGGTGAGTAACGCGTGAGGAACCTGCCTCAAAGAGGGGGACAACAGTTGGAAACGACTGCTAATACCGCATAAGCCCACGGGTCGGCATCGATCTGAGGGAAAAGGAGCGATCCGCTTTGAGATGGCCTCGCGTCCGATTAGCTAGTTGGTGAGGTAACGGCCCACCAAGGCGACGATCGGTAGCCGGACTGAGAGGTTGAACGGCCACATTGGGACTGAGACACGGCCCCAGACTCCTACGGGAGGCAGCAGTGGGGAATATTGCACAATGGGGAAACCCTGATGCAGCGACGCCGCGTGGAGGAAGAAGGTCTTCGGATTGTAAACTCTGTTGTGGGAAGATAATGACGTACCAAC
>FQH3XDB01A3E99|1000|61
GACGAACGCTGGCGGCGCGCCTAACACATGCAAGTCGAACGAGCGAAGAGGAGCTTGCTTCTCTGAGCGAGTGGCGAACGGGTGAGTAACGCGTGAGGAACCTGCCTCAAAGAGGGGGACAACAGTTGGAAACGACTGCTAATACCGCATAAGCCCACGGATCGGCATCGATCTGAGGGAAAGGAGCAATCCGCTTTGAGATGGCCTCGCGTCCGATTAGCTGGTTGGTGAGGTAACGGCCCACCAAGGCGACGATCGGTAGCCGGACTGAGAGGTTGAACGGCCACATTGGGACTGAGACACGGCCCCAGACTCCTACGGGAGGCAGCAGTGGGAATATTGCACAATGGGGGAAACCCTGATGCAGCGACGCCGCGTGGAGGAAGAAGGTCTTCGGATTGTAAACTCCTGTTGTTGAGGAAGATAACTGACGGTACTCAACAAGGAAGTGACGGCTAACTACGTGCCAGCAGCCG
>FQH3XDB01AKXK8|1001|29
GACGAACGCTGGCGGCGCGCCTAACACATGCAAGTCGAACGAGCGAAGAGGAGCTTGCTTCTCTGAGCGAGTGGCGAACGGGTGAGTAACGCGTGAGGAACCTGCCTCAAAGAGGGGGACAACAGTTGGAAACGACTGCTAATACCGCATAAGCCCACGGGTCGGCATCGACCTGAGGGAAAAGGAGCAATCCGCTTTGAGATGGCCTCGCGTCCGATTAGCTAGTTGGTGAGGTAACGGCCCACCAAGGCGACGATCGGTAGCCGGACTGAGAGGTTGAACGGCCACATTGGGACTGAGACACGGCCCCAGACTCCTACGGGAGGCAGCAGTGGGGAATATTGCACAATGGGGGAAACCCTGATGCAGCGACGCCGCGTGGAGGAAGAAGGTCTTCGGGATTGG
>FQH3XDB01BB0G1|1002|5
GACGAACGCTGGCGGCGCGCCTAACACATGCAAGTCGAACGAGCGAAGAGGAGCTTGCTTCTCTGAGCGAGTGGCGAACGGGTGAGTAACGCGTGAGGAACCTGCCTCAAAGAGGGGGGCAACAGTTGGAAACGACTGCTAATACCGCATAAGCCCACGACCCGGCATCGGGTTGAGGGAAAAGGAGCAATCCGCTTTGAGATGGCCTCGCGTCCGATTAGCTAGTTGGTGAGGTAACGGCCCACCAAGGCAACGATCGGTAGCCGGCCTGAGAGGGCGATCGGCCACATTGGGGACTGAGAGACGGCCCCAGACTCCTACGGGAGGCAGCAGTCGGGAATATTGCGCAATGGAGGAAACTCTGACGCAGTGACGCCGCGTATAGGAAGAAGGTTTTCGGATTGTAAACTATTGTCGTTAGGGAAGAGAAGG
>FQH3XDB01DU4MN|1003|2
GACGAACGCTGGCGGCGCGCCTAACACATGCAAGTCGAACGAGTGATGGAAAGCTTGCTTTCCAAAGCGAGTGGCGAACGGGTGAGTAACGCGTGAGGAACCTGCCTCAAAGAGGGGGACAACAGTTGGAAACGACTGCTAATACCGCATAAGCCCACGGGTCGGCATCGATCTGAGGGAAAAGGAGCAATCTGCTTTGAGATGGCCTCGCGTCCGATTAGCTGGTTGGTGAGGTAACGGCCCACCAAGGCGACGATCGGTAGCCGGACTGAGAGGTTGAACGGGCCACATTGGGACTGAGACACGGCCCAGGACTCCTACGGGAGGCAGCAGTGGGGAATATTGCACAATGGGGGAAACCCTGATGCAGCGACGCCGCGTGG
>FQH3XDB01CIJP4|1004|1
GACGAACGCTGGCGGCGCGCCTAACACATGCAAGTCGAACGGGATTGGGAGGAGCTTGCTCTTCTTAATCTAGTGGCGAACGGGTGAGTAACGCGTGAGTAACCTGCCCTAGAGTGGGGGACAACAGTTGGAAACGACTGCTAATACCGCATAAGCCCACGGCACCGCATGGTGCTGCGGGAAAAGGATTTATTCGCTTTAGGATGGACTCGCGTCCAATTAGCTAGTTGGTGAGGTAACGGCCCACCAAGGCGACGATTGGTAGCCGGACTGAGAGGTTGAACGGCCACATTGGGACTGAGACACGGCCCAGACTCCTACGGGAGGCAGCAGTGGGGGATATTGCACAATGGGGGAACCCTGATGCAGCGACGCCGCGTGG
>FQH3XDB01EZSE5|1005|4
GACGAACGCTGGCGGCGCGCCTAACACATGCAAGTCGAACGGAGAATATCCTTCGGGATATTCTGAGTGGCGGACGGGTGAGTAACGCGTGAGCAATCTGCCTTTCAGAGGGGGATAATGTCTGGAAACGGACACTAATACCGCATAAGATGTTAATAACGCATGTTGTAGACATCAAAGGAGAAATCCGCTGAGAGATGAGCTCGCGTCCAATTAGGTAGATGGTGAGGTAACGGCCACCATGCCGACGATTGGTAGCCGGACTGAGAGGTTGAACGGCCACATTGGGACTGAGACACGGCCCAGACTCCTA
>FQH3XDB01BSJ5Y|1006|1
GACGAACGCTGGCGGCGCGCCTAACACATGCAAGTCGAACGGAGCATTGAGAGCTTGCTTTTAATGCTTAGTGGCGAACGGGTGAGTAACGCGTGAGTAACCTGCCCTAGAGTGGGGACAACAGTTGGAAACGACTGCTAATACCGCATAAGCCCACGGCCCGGCATCGGGCTGAAGGGAAAAGGATTTATTCGCTTTAGGATGGACTCGCGTCCAATTAGCTAGTTGGTGAGGTAACGGCCCACCAAGGCGACGATTGGTAGCCGGACTGAGAGGTTGAACGGCCACATTGGGACTGAGACACGGCCCAGACTCCTACGGAGGCAGCAGTGGGGGATATTGCACAATGGGGGAAACCCTGATGCAGCGACGCCGC
>FQH3XDB01CRLU6|1007|9
GACGAACGCTGGCGGCGCGCCTAACACATGCAAGTCGAACGGAGCTTTGAGAGCTTGCTTTTAAAGCTTAGTGGCGAACGGGTGAGTAACGCGTGAGTAACCTGCCCTAGAGTGGGGGACAACAGTTGGAAACGACTGCTAATACCGCATAAGCCCACGACCCGGCATCGGGTTGAGGGAAAAGGATTTATTCGCTTTAGGATGGACTCGCGTCCAATTAGCTAGTTGGTGAGGTAACGGCCACCAAGGCGACGATTGGTAGCCGGACTGAGAGGTTGAACGGCCACATTGGGACTGAGGACACGGCCCAGACTCCTACGGAGGCAGCAGTGGGGG
>FQH3XDB01CQJK6|1008|1
GACGAACGCTGGCGGCGCGCCTAACACATGCAAGTCGAACGGAGCTTGAAGAAGCTTGCTTTTTTAAGCTTAGTGGCGAACGGGTGAGTAACGCGTGAGTAACCTGCCCTAGAGTGGGGGACAACAGTTGGAAACGACTGCTAATACCGCATAAGCCCACGGATTCGCATGGATCTGAGGGAAAAGGATTTATTCGCTTTAGGATGGACTCGCGTCCAATTAGCTAGTTGGTGAGGTAACGGCCCACCAAGGCGACGATTGGTAGCCGGACTGAGAGGTTGAACGGCCACATTGGGACTGAGACACGGCCCAGACTCCTACGGGAGGCAGCAGTGGGGATATTGCACAAGTGGGGAAACCCTGATGCAGCGACGCCGCGTGGAGGAA
>FQH3XDB01DTXT5|1009|5
GACGAACGCTGGCGGCGCGCCTAACACATGCAAGTCGAACGGAGCTTTGAGAGCTTGCTTTTAAAGCTTAGTGGCGAACGGGTGAGTAACGCGTGAGTAACCTGCCCTAGAGTGGGGACAACAGTTGGAAACGACTGCTAATACCGCATAAGCCCACGGGTCGGCATCGATCTGAGGGAAAAGGATTTATTCGCTTTAGGATGGACTCGCGTCCAATTAGCTAGTTGGTGAGGTAACGGCCCACCAAGGCGACGATTGGTAGCCGGACTGAGAGGTTGAACGGCCACATTGGGACTGAGACACGGCCCAGACTCCTACGGGAGGCAGCAGTGGGGGATATTGCACAATGGGGG
>FQH3XDB01DGKR5|1010|1
GACGAACGCTGGCGGCGCGCCTAACACATGCAAGTCGAACGGAGCTTGAGAGAGCTTGCTTTTTTAAGCTTAGTGGCGAACGGGTGAGTAACGCGTGAGTAACCTGCCCTAGAGTGGGGGACAACAGTTGGAAACGACTGCTAATACCGCATAAGCCCACGGTACCGCATGGCACTGAGGGAAAAGGATTTATTCGCTTTAGGATGGACTCGCGTCCAATTAGCTAGTTGGTGAGGTAACGGCCCACCAAGGCGACGATTGGTAGCCGGACTGAGAGGTTGAACGGCCACATTGGGGACTGAGACACGGCCCAGACTCCTACGGGAGGCAGCAGTGGGGGATATTGCACAATGGGGGAAACCCTGATGCAGCGACGCC
>FQH3XDB01BU9D2|1011|1
GACGAACGCTGGCGGCGCGCCTAACACATGCAAGTCGAACGGAGTAAAGAAAGCTTGCTTTCTTTGCTTAGTGGCGAACGGGTGAGTAACACGTGAGCAATCTGCCTTCGAGTGGGGGACAACAGTTGGAAACGACTGCTAATACCGCATAAGACCACAGTCCCACATGGGGACAGCGGTCAAAAGGATTTATTCGCTTGAGGATGAGCTCGCGTCCGATTAGATAGTTGGTGAGGTAACGGCCCACCAAGTCGACGATCGGTAGCCGGACTGAGAGGTTGAACGGCCACATTGGGACTGAGACACGGCCCAGACCTCCTACGGGAGGCAGCAGTGGGGAATATTGCACAATGGGGGAAACCCTGATGCAGCGACGCCGCGTGAGCGAAGAAGATCT
>FQH3XDB01B5506|1012|1
GACGAACGCTGGCGGCGCGCCTAACACATGCAAGTCGAACGGAGTTAAGAGGAGCTTGCTTTTCTTAACTTAGTGGCGAACGGGTGAGTAACGCGTGAGTAACCTGCCCTAGAGTGGGGACAACAGTTGGAAACGACTGCTAATACCGCATAAGCCCACGGCTCGGCATCGAGCTGAGGGAAAAGATTATCGCTTTAGGATGGACTCGCGTCCAATTAGCTAGTTGGTGAGGTAACGGCCACCAAGGCGACGATTGGTAGCCGGACTGAGAGGTTGAACGGCCACATTGGGACTGAGACACGGCCCAGACTCCTAC
>FQH3XDB01D9CRC|1013|1
GACGAACGCTGGCGGCGCGCCTAACACATGCAAGTCGAACGGAGTTAAGAGGAGCTTGCTTTTCTTAACTTAGTGGCGAAACGGGTGAGTAACGCGTGAGTAACCTGCCCTAGAGTGGGGGACAACAGTTGGAAACGACTGCTAATACCGCATAAGCCCACGGTCTCGCATGGGACTGAGGGAAAAGGATTTATTCGCTTTAGGATGGACTCGCGTCCAATTAGCTAGTTGGTGAGGTAACGGCCCACCAAGGCGACGATTGGTAGCCGGACTGAGAGGTTGAACGGCCACATTGGGACTGAGACACGGCCCCAGACTCCTACGGGAGGCAGCAGTGGGGATATTGCACAATGGGGGGAACCCTGATGC
>FQH3XDB01DM7L7|1014|1
GACGAACGCTGGCGGCGCGCCTAACACATGCAAGTCGAACGGAGTTGAGAGGAGCTTGCTTTTCTTAACTTAGTGGCGAACGGGTGAGTAACGCGTGAGTAACCTGCCCTAGAGTGGGGGACAACAGTTGGAAACGACTGCTAATACCGCATAAGCCCACGACCCGGCATCGGGGTTGAGGGAAAAGGATTTATTCGCTTTAGGATGGACTCGCGTCCAATTAGCTAGTTGGTGAGGTAACGGCCCACCAAGGCGACGATTGGTAGCCGGACTGAGAGGTTGAACGGCCACATTGGGACTGAGACACGGCCCAGACTCCTACGGGAGGCAGCAGTGGGGGATATTGCACAATGGGGGAAACCCTGATGCAGCGACGCCGCGTGAGCGAAGAAGTATTTCGGTATGTAAAGCTCTATCAGCGAAGGGAAGAAAAGTGACGGTACCTGACTAAG
>FQH3XDB01EOI18|1015|1
GACGAACGCTGGCGGCGCGCCTAACACATGCAAGTCGAACGGAGTTGAGGAGAGCTTGCTTTCCTTAACTTAGTGGCGAACGGGTGAGTAACGCGTGAGTAACCTGCCCTAGAGTGGGGGACAACAGTTGGAAACGACTGCTAATACCGCATAAGCCCACGGATTCGCATGGATCTGAGGGAAAAGGATTTATTCGCTTTAGGATGGACTCGCGTCCAATTAGCTAGTTGGTGAGGTAACGGCCCACCAAGGCGACGATTGGTAGCCGGACTGAGAGGTTGAACGGCCACATTGGGACTGAGACACGGCCCAGACTCCTACGGGAGGCAGCAGTGGGGGATATTGCACAATGGGGGAAACCCTGATGCAGCGACGCCGCGTGGA
>FQH3XDB01D3OXE|1016|1
GACGAACGCTGGCGGCGCGCCTAACACATGCAAGTCGAACGGAGTTGCATCGACAGAAGCCTTCGGGTGGAAGATGATGTAACTTAGTGGCGGACGGGTGAGTAACACGTGAGCAACCTGCCTATGAGAGGGGGATAACGTTCTGAAAAAGAACGCTAATACCGCATAACGTGCAAGAGAGACATCTCTCTTGCACCAAAGGAGCAATCCGCTGATAGATGGGCTCGCGTCCGATTAGGTAGTTGGTAGGTAACGCCTACCAGCCGACGATCGGTAGCCGGACTGGAG
>FQH3XDB01ET774|1017|1
GACGAACGCTGGCGGCGCGCCTAACACATGCAAGTCGAACGGGGCATTGAGAGCTTGCTTTTAATGCTTAGTGGCGAACGGGTGAGTAACGCGTGAGTAACCTGCCCTGGAGTGGGGGACAACAGTTGGAAACGACTGCTAATACCGCATAAGCCCACGACCCGGCATCGGGTTGAGGGAAAAGGAGCAATCCGCTTTGAGATGGCCTCGCGTCCGATTAGCTAGTTGGTGAGGTAATGGCCCACCAAGGCGACGATCGGTAGCCGGACTGAGAGGTTGAACGGCCACATTGGGACTGAGACACGGCCCCAGACTCCTACGGGAGGCAGCAGTGGGGAATATTGCACAATGGGGGAAACCCTGATGCAGCGACGCCGCGTGGAGGAAGAAGGGTCTTCGGGATTGGTAAAACTCCTGTTGTTGGGGAAGATAATGACGGTACCAACAAGGAAGTGACGGCTAACTACGTGCCAGCAGCCGCGG
>FQH3XDB01CPS26|1018|7
GACGAACGCTGGCGGCGCGCCTAACACATGCAAGTCGAACGGAATGAGAGGGAGCTTGCTTCTTCTTGTTTAGTGGCGAACGGGTGAGTAACGCGTGAGGAACCTGCCTCAAAGAGGGGGACAACAGTTGGAAACGACTGCTAATACCGCATAAGCCCACGGACCGGCATCGGTCTGAGGGAAAAGGATTTTATCCGCTTTGAGATGGCCTCGCGTCCGATTAGCTAGTTGGTGAGGTAACGGCCCACCAAGGCGACGATCGGTAGCCGGACTGAGAGGTTGAACGGCCACATTGGGACTGAGACACGGCCCAGACTCCTACGGGAGGCAGCAGTGGGGAATATTGCACAATGGGGGAACCCTGATGCAGCGACGCCGCGTGGAGGAAGAAGGTCTTC
>FQH3XDB01A544S|1019|186
GACGAACGCTGGCGGCGCGCCTAACACATGCAAGTCGAACGGAATGAGAGGGAGCTTGCTTCTTCTTATTTAGTGGCGAACGGGTGAGTAACGCGTGAGGAACCTGCCTCAAAGAGGGGGGACAACAGTTGGAAACGACTGCTAATACCGCATAAGCCCACGGACCGGCATCGGTCTGAGGGAAAAGGAGAGATCCGCTTTGAGATGGCCTCGCGTCCGATTAGCTAGTTGGTGAGGTAACGGCCCACCAAGGCGACGATCGGTAGCCGGACTGAGAGGTTGAACGGCCACATTGGACTGAGACACGGCCCAGACTCCTACGGGAGGCAGCAGTGGGGAATATTGCACGAATGGGGGGAACCCTGATGCAGCGACG
>FQH3XDB01B1Q2E|1020|3
GACGAACGCTGGCGGCGCGCCTAACACATGCAAGTCGAACGGAATGAGAGGGAGCTTGCTTCTTCTTATTTAGTGGCGAACGGGTGAGTAACGCGTGAGGAACCTGCCTCAAAGAGGGGGACAACAGTTGGAAACGACTGCTAATACCGCATAAGCCCACGGATCGGCATCGGTCTGAGGGAAAAGGAGAGATTCCGCTTTGAGATGGCCTCGCGTCCGATTAGCTAGTTGGTGAGGTAACGGCCCACCAAGGCGACGATCGGTGGCCGGACTGAGAGGTTGAACGGCCACATTGGGACTGAGACACGGCCCCAGACTCCTACGGGAGCAGCAGTGGGGAATATTGCACAATGGGGGGAACCCTGATGCAGCGACGCCGCGTGGAGGAAGAAGGTCTTCGGGATTGAAACTCCTGTTGTTGAGGAAGATAATGACGGTACT
>FQH3XDB01EHAYR|1021|13
GACGAACGCTGGCGGCGCGCCTAACATATGCAAGTCGAACGGAATGAGAGGGAGCTTGCTTCTTCTTATTTAGTGGCGAACGGGTGAGTAACGCGTGAGGAACCTGCCTCAAAGAGGGGGACAACAGTTGGAAACGACTGCTAATACCGCATAAGCCCACGGACCGGCATCGGTCTGAGGGAAAAGGATTTATCCGCTTTGAGATGGCCTCGCGTCCGATTAGCTAGTTGGTGAGGTAACGGCCCACCAAGGCGACGATCGGTAGCCGGACTGAGAGGTTGAACGGCCACATTGGGACTGAGACACGGCCCAGACTCCTACGGGAGGCAGCAGTGGGAATATTGCACAATGGGGGAACCCTGATGCAGCGACGCCGCGTGGAGGAAGAAGGTCTTCGGATTGTAAACTCCTGTTGTTGAGGAAGATAATGACGGTACTCAACAAGGAAGTGACGGCTAACTACGTGCC
>FQH3XDB01CCA8K|1022|2
GACGAACGCTGGCGGCGCGCCTAACACATGCAAGTCGAACGGAATGAGAGGGAGCTTGCTTCTTCTTGTTAGTGGCGAACGGGTGAGTAACGCGTGAGGAACCTGCCTCAAAGAGGGGGACAACAGTTGGAAACGACTGCTAATACCGCATAAGCCCACAGACCGGCATCGGTCAGGGGAAAGATTTATCCGCTTTGAGATGGCCTCGCGTCCGATTAGCTAGTTGGTGAGGTAACGGCCACCAAGGCGACGATCGGTAGCCGGACTGAGAGGTTGAACGGCCACATTGGGACTGAGACACGGCCCAGACTCCTACGGGAGGCAGCAGTGGGGAATATTGCACAATGGGGGAACCCTGATGCAGCGACGCCGCGTGGAGGAAGAAGGTCTTCGGGATTGTAAACTCCTGT
>FQH3XDB01EIW0K|1023|87
GACGAACGCTGGCGGCGCGCCTAACACATGCAAGTCGAACGGAATGAGAGGGAGCTTGCTTCTTCTTGTTTAGTGGCGAACGGGTGAGTAACGCGTGAGGAACCTGCCTCAAAGAGGGGGACAACAGTTGGAAACGACTGCTAATACCGCATAAGCCCCACGGACCGGCATCGGTCTGAGGGAAAAGGAGAAATCCGCTTTGAGATGGCCTCGCGTCCGATTAGCTAGTTGGTGAGGTAACGGCCCACCAAGGCGACGATCGGTAGCCGGACTGAGAGGTTGAACGGCCACATTGGGACTGAGACACGGCCCAGACTCCTACGGGAGGCAGCAGTGGGGAATATTGCACAAGTGGGGGGAACCCTGATGCAGCGACGCCGCGTGGAGGAAGAAGGTCTTCGGATTGGTAAACTCCTGTTGTTGAGGAAGATAATGACGGTACTCAACAAGGAAGTGACGGCTAACTACGTGCCAGCAG
>FQH3XDB01AUW6E|1024|1
GACGAACGCTGGCGGCGCGCCTAACACATGCAAGTCGAACGGAATGAGAGGGAGCTTGCTTCTTCTTGTTTAGTGGCGAACGGGTGAGTAACGCGTGAGGAACCTGCCTCAAAGAGGGGGACAACAGTTGGAAACGACTGCTAATACCGCATAAGCCCCACGGACCGGCATCGGTCTGGAAGGGAAAAGGAGAGATCCGCTTTGAGATGGCCTCGCGTCCGATTAGCTAGTTGGTGAGGTAACGGCCCACCAAGGCGACGATCGGTAGCCGGACTGAGAGGTTGAACGGCCACATTGGGACTGAGACACGGCCCAGACTCCGTACGGGAGGCAGC
>FQH3XDB01DLK1N|1025|9
GACGAACGCTGGCGGCGCGCCTAACACATGCAAGTCGAACGGAATGAGAGGGAGCTTGCTTCTTCTTGTTTAGTGGCGAACGGGTGAGTAACGCGTGAGGAACCTGCCTCAAAGAGGGGGACAACAGTTGGAAACGACTGCTAATACCGCATAAGCCCACAGGTCGGCATCGACCAGGGGGAAAAGGATCATATCCGCTTTGAGATGGCCTCGCGTCCGATTAGCTGGTTGGTGAGGTAACGGCCCACCAAGGCGACGATCGGTAGCCGGACTGAGAGGTTGAACGGGCCACATTGGGACTGAGACACGGCCCCAGACTCCTACGGGAGGCAGCAGTGGGGGATATTGCACAATGGGGGAAACCCTGATGCAGCGACGCCGCGTGGAGGAAGAACGGGTCTTCGGGATTGGTAAAACTCCTGTTGTTGAGGAAGATAATGACGGTACTCAACAAGGAAGT
>FQH3XDB01EDWLO|1026|1
GACGAACGCTGGCGGCGCGCCTAACACATGCAAGTCGAACGGAATGAGAGGGAGCTTGCTTCTTCTTGTTTAGTGGCGAACGGGTGAGTAACGCGTGAGGAACCTGCCTCAAAGAGGGGGACAACAGTTGGAAACGACTGCTAATACCGCATAAGCCCACGGACCGGCATCGGTTTGAGGGAAAAGGAGAAATCCGCTTTGAGATGGCCTCGCGTCCGATTAGCTAGTTGGTGAGGTAACGGCCTACCAAGTCGACGATCAGTAGCCGAACTGAGAGGTTGATCGGCCACATTGGGACTGAGACACGGCCCCAGACTCCTACGGGAGGCAGCAGTGGGGGATATTGCGCAATGGGGGCAACCCTGACGCAGCAACGCCGCGTGAAGGAAGAAGGTTTTCGGATCGTAAACTTCTTAAGTGGGGAAGATAGTGACGGTACCCACAGAATAAGCCACGGCTAACTACGTGCCAGCAGCC
>FQH3XDB01C4ITR|1027|12
[truncated: 1,200,045 more chars]
